# Trends in the Population Prevalence of People Who Inject Drugs in US Metropolitan Areas 1992–2007

**DOI:** 10.1371/journal.pone.0064789

**Published:** 2013-06-05

**Authors:** Barbara Tempalski, Enrique R. Pouget, Charles M. Cleland, Joanne E. Brady, Hannah L. F. Cooper, H. Irene Hall, Amy Lansky, Brooke S. West, Samuel R. Friedman

**Affiliations:** 1 Institute for AIDS Research, National Development and Research Institutes, Inc. (NDRI), New York, New York, United States of America; 2 College of Nursing, New York University (NYU), New York, New York, United States of America; 3 Department of Epidemiology, Columbia University, New York, New York, United States of America; 4 Behavioral Sciences and Health Education, Rollins School of Public Health at Emory University, Atlanta, Georgia, United States of America; 5 Centers for Disease Control, Division of HIV/AIDS Prevention, National Center for HIV/AIDS, Viral Hepatitis, STD, and TB Prevention, Atlanta, Georgia, United States of America; 6 Centers for Disease Control^,^ Division of HIV/AIDS Prevention, Surveillance, Epidemiology and Laboratory Science, Atlanta, Georgia, United States of America; 7 Department of Epidemiology, Johns Hopkins Bloomberg School of Public Health, Baltimore, Maryland, United States of America; Centers for Disease Control and Prevention, United States of America

## Abstract

**Background:**

People who inject drugs (PWID) have increased risk of morbidity and mortality. We update and present estimates and trends of the prevalence of current PWID and PWID subpopulations in 96 US metropolitan statistical areas (MSAs) for 1992–2007. Current estimates of PWID and PWID subpopulations will help target services and help to understand long-term health trends among PWID populations.

**Methodology:**

We calculated the number of PWID in the US annually from 1992–2007 and apportioned estimates to MSAs using multiplier methods. We used four types of data indicating drug injection to allocate national annual totals to MSAs, creating four distinct series of component estimates of PWID in each MSA and year. The four component estimates are averaged to create the best estimate of PWID for each MSA and year. We estimated PWID prevalence rates for three subpopulations defined by gender, age, and race/ethnicity. We evaluated trends using multi-level polynomial models.

**Results:**

PWID per 10,000 persons aged 15–64 years varied across MSAs from 31 to 345 in 1992 (median 104.4) to 34 to 324 in 2007 (median 91.5). Trend analysis indicates that this rate declined during the early period and then was relatively stable in 2002–2007. Overall prevalence rates for non-Hispanic black PWID increased in 2005 as compared to other racial/ethnic groups. Hispanic prevalence, in contrast, declined across time. Importantly, results show a worrisome trend in young PWID prevalence since HAART was initiated – the mean prevalence was 90 to 100 per 10,000 youth in 1992–1996, but increased to >120 PWID per 10,000 youth in 2006–2007.

**Conclusions:**

Overall, PWID rates remained constant since 2002, but increased for two subpopulations: non-Hispanic black PWID and young PWID. Estimates of PWID are important for planning and evaluating public health programs to reduce harm among PWID and for understanding related trends in social and health outcomes.

## Introduction

Injection drug use continues to account for a substantial proportion of new Human immunodeficiency virus (HIV) diagnoses in the United States, and is the third most frequently reported risk factor for HIV infection, after male-to-male sexual contact and high-risk heterosexual contact [1], [2]. Overall, people who inject drugs (PWID) represented 9% of new HIV infections in 2009 and 17% of those living with HIV in 2008 [2], [3]. Yet, the most alarming feature of HIV among PWID in the U.S. is racial/ethnic disparities. Disparities have been apparent in HIV among PWID since early in the epidemic [4], [5], [6], [7] and still are very marked [1], [8], [9], [10]. More than 50% of PWID living with a diagnosis of HIV infection at the end of 2009 were non-Hispanic black, 27% were Hispanic, and 21% were non-Hispanic white [1], [2], [10]. Non-Hispanic blacks who inject drugs are ten times as likely to be diagnosed with HIV as non-Hispanic white injectors [8], [9].

PWID are at high risk for HIV, hepatitis B and C, and many socially related problems. PWID experience high rates of morbidity and mortality, often from drug overdose, endocarditis, cellulitis, and abscesses [11], [12], [13], [14], [15] – in addition to, increased rates of exposure to violence and injury [16], [17], [18]. Further, PWID experience poor health outcomes due to either lack of or delayed access to effective treatment, continuation of illicit drug use, and depression and negative life events [19], [20], [21], [22], [23], [24].

Drug use patterns and prevalence reflect notable differences in the U.S. [25], [26], [27]. U.S. figures, as reported by SAMHSA [27], for 2002–05 show injection was the primary route of administration for a higher proportion of heroin users (42%) than for methamphetamine (9%), stimulant (5%) or cocaine users (3%).

Although difficult to ascertain, geographic-specific data over time on PWID prevalence rates are important, as they may help policy makers allocate resources and establish public policy to reduce harm among PWID and PWID subpopulations [28], [29], [30]. Additionally, such data could provide a foundation for the design, implementation and evaluation of structural interventions and service coverage, such as the expansion of Opioid Treatment Programs (OTPs) and Medication-Assisted Treatment (MAT) facilities in areas of need [31], [32], [33]. Trend data on PWID prevalence may help forecast which metropolitan areas may be at greater risk for outbreaks of drug injecting and blood-borne infections associated with drug injection. Data on PWID populations also allows study of patterns of change of PWID prevalence in relation to social, economic and political predictors in metropolitan areas [32], [33].

Drug use–particularly illicit and injection drug use –carries a heavy stigma. PWID are near the bottom in terms of social tolerance in the hierarchy of client groups [34], [35], and most are reluctant to divulge any illegal drug use or needle use. Thus, despite the need for data on numbers of PWID, several factors make it difficult to assess the actual number of PWID over time and across U.S. geographic areas [36], [37], [38], [39], [40], [41], [42].

### Historical Variation in PWID Prevalence and Subpopulation Prevalence Rates Across Large U.S. Metropolitan Statistical Areas (MSA)

Brady and colleagues (2008) [43] created estimates of PWID population prevalence for the same 96 Metropolitan Statistical Areas (MSAs) that are studied here for 1992–2002. These data indicated an overall decreasing trend in prevalence across the 96 MSAs until 2000, after which there was a slight increase; they also identified substantial variation across MSAs in these trends [43].

Analyses have also discovered substantial variation in PWID trends across subpopulations over time. Cooper and colleagues (2008) [44] reported that non-Hispanic black PWID prevalence declined from a median of 279 injectors per 10,000 adults in 1992 to 156 injectors per 10,000 adults in 2002. PWID prevalence for non-Hispanic white adults remained relatively flat over time (median values ranged between 86 and 97 injectors per 10,000 adults). Hispanic PWID prevalence rate also declined significantly (1992 mean = 192, median = 133; 2002 mean = 144, median = 93) [45].

Chatterjee and colleagues (2011) [46] found the mean proportion of young PWID (ages 15–29) across MSAs increased by almost 20%, from a mean of 103 per 10,000 in 1996 to 122 per 10,000 in 2002. Young PWID increased significantly in nearly thirty-four separate MSAs, and declined significantly in approximately ten.

In this paper, we revise and extend the PWID estimates for the period 1992–2007 for both the overall PWID population and for specific subpopulations of PWIDs. PWID subpopulations are defined by: (1) sex (male and female); (2) age (youth 15–29 years and older 30–64 years); and (3) race/ethnicity (non-Hispanic white, non-Hispanic black, and Hispanic). Our estimates capture current injectors as opposed to ever-injected (i.e., since 1977). Measures and estimates of current injectors provide valuable information for needs assessment, public health program planning, program evaluation, and development of public policy to reduce harm among PWID. Current estimates among PWID are of particular importance for linking PWID to services and care.

In the following sections, we describe our method of estimating PWID prevalence rates for adults living in large MSAs each year between 1992 and 2007 both overall and for specific subpopulations; validate these estimates; and report temporal and spatial variations in these estimates. Lastly, we close with a discussion of the PWID estimation methods and limitations, and discuss possible causes and consequences of the observed trends in PWID and PWID subpopulations.

## Data and Methods Overview

In this analysis, we update and amend previously published methods of estimating current PWID prevalence among adult residents of large U.S. MSAs [43], [47]. For each MSA, four component sets of estimates are calculated. Each of these allocates a proportion of the national total of PWID to an MSA. The first set of estimates represents the MSAs proportion of the national total of PWID based on the proportion of HIV Counseling and Testing (Centers for Disease Control and Prevention [CDC] HIV Counseling and Testing Services database [CTS] [48]) events in which PWID were clients. The second uses the MSAs proportion of the national total of PWID in drug abuse treatment in the Uniform Facility Data Set (UFDS) [49], [50], [51] and Treatment Entry Data System (TEDS) from the Substance Abuse and Mental Health Service Administration (SAMHSA) [52]. These estimates both depend on the extent to which services are provided for PWID–which can vary by budgetary, siting and other decisions made by funders or service providers. They thus need to be balanced by estimates that might be higher where under-servicing is particularly high. We accomplish this by using a third component estimate reflecting: (1) the extent to which HIV-positive PWID develop new AIDS diagnoses (data from CDC’s National HIV Surveillance System on AIDS diagnoses among PWID) [53]; and (2) adjusting this with our previous estimates of HIV prevalence among PWID in the MSA [54]. A fourth estimate is based on extrapolation of estimates from earlier years to more recent years [55], [56], creating four distinct series of estimates of the number of injectors in each MSA and year. The final estimates are the result of smoothing each U.S. apportioned data series over time using loess regression and taking the mean value of the four series as a best estimate of PWID for that MSA and year.

**Figure 1:**
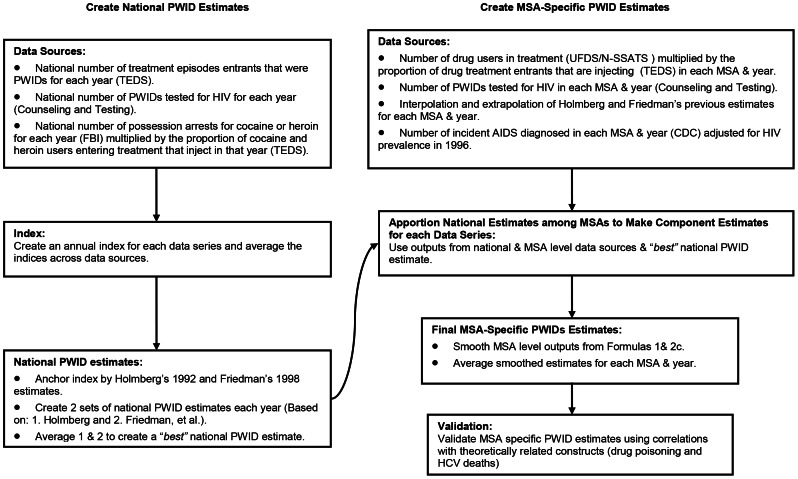
Flowchart of Methods for Estimating People Who Inject Drugs in 96 Metropolitan Areas from 1992–2007.

In order to compute MSA-level estimates from the national estimates of PWID for each year, we used multiplier methods to allocate the data. In the past, such methods have been widely used to estimate the numbers of global or local problem drug-using populations [37], [56], [57]. National estimates were attributed to MSAs using the four data series discussed above (Figure 1). As in Friedman et al (2004) [55] and Brady et al (2008) [43], we hypothesized that apportioning the national number of PWID to each MSA using different data sources helps to balance biases and offset potential limitations of individual datasets. For example, increases in drug treatment funding and treatment slots for PWID could make it appear as if the number of PWID was increasing. By combining our series measures, some of which are either not subject to service bias or inversely related to services, we mitigate such service bias effects.

The resulting estimates were smoothed and averaged to create a PWID-specific estimate for each MSA and year. These MSA estimates were validated using yearly cross-sectional correlations with variables theoretically related to drug injection (i.e., unemployment, HCV mortality, and drug-poisoning mortality). Concordance between these constructs and our estimates lends credence to the validity of our estimates [43]. Our methods and steps used to create national and MSA-specific PWID prevalence estimates from 1992 to 2007 are described below and are outlined in Figure 1.

**Figure 2:**
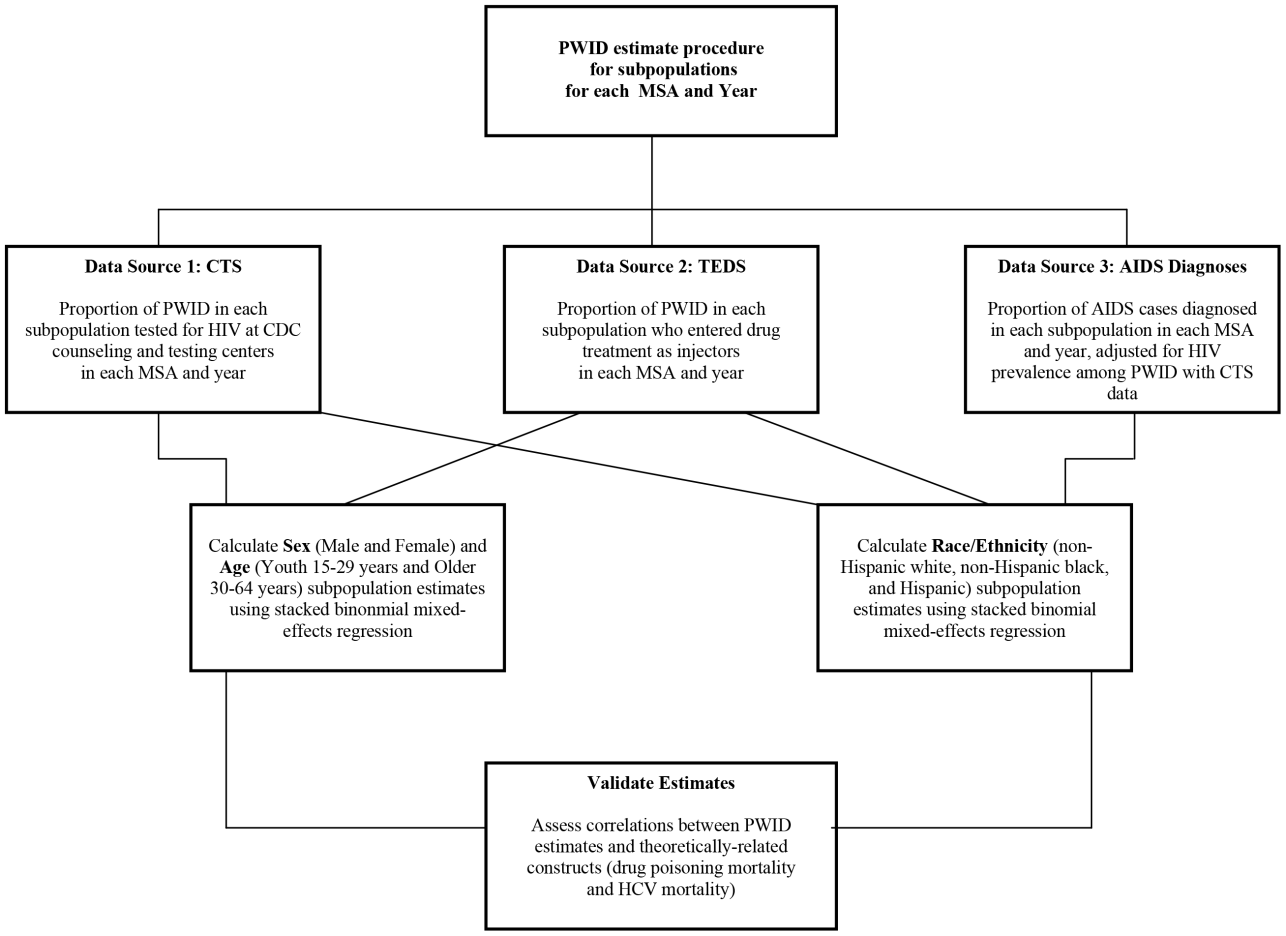
Flowchart of Methods for Estimating People Who Inject Drugs Prevalence for Subpopulations in 96 MSAs, 1992–2007.

Lastly, we estimate PWID prevalence rates for three sets of subpopulations: (1) sex (male and female); (2) age (youth ages 15–29 years, older ages 30–64); and (3) race/ethnicity (non-Hispanic white, non-Hispanic black, and Hispanic). Our subpopulation estimates use a different set of procedures than that of the overall PWID estimates. Here, to estimate subpopulation prevalence we multiplied yearly estimates of the proportion of PWID who were in the subpopulation in each MSA by yearly estimates of the total number of PWID in each MSA, and divided by their respective populations. The proportions of PWID who were in each subpopulation were estimated by combining three data series based on indicators of PWID use of health services and AIDS surveillance data. The PWID subpopulations are combined by using predicted values from a binomial mixed-effects regression to produce MSA-specific rates. The PWID subpopulation estimates are also validated using yearly cross-sectional correlations with variables theoretically related to drug injection. Figure 2 includes a flow chart of procedures for estimating PWID subpopulations prevalence.

### Metropolitan Statistical Areas: Unit of Analysis

This analysis estimates the annual number of current PWID in 96 MSAs for each year from 1992 to 2007. The June 30, 1993 Office of Management and Budget [47] MSA definitions, which were based on the application of 1993 metropolitan area standards to 1990 census data, were used in these analyses, except in New England, where we instead used New England County Metropolitan Areas (NECMA) definitions [47], [58].

MSAs are defined to represent a central population in a large city and surrounding urban and suburban areas which have strong economic and social ties [47], [58]. The MSA is a particularly useful geographic unit for studying PWID and HIV epidemiology among PWID for several reasons. Drug distribution networks often parallel networks of distribution of legal commodities [55], [59]. In addition, social networks of drug users often include individuals from inside and outside central cities, but include individuals from multiple MSAs less often [60], [61]. Most importantly, MSAs are large enough to have discrete HIV outbreaks and unique data on drug use and HIV while still being small enough to be sensitive to relatively small differences in HIV outbreaks and small changes in social and economic contexts and in population characteristics over time.

### Step 1: Estimating the Number of PWID in the United States Annually from 1992 to 2007

We analyzed three data series (described below) to estimate the number of PWID nationwide for each year of the study period. Our first step was to create an index for each data series by dividing the number of PWID in the U.S. for that year and the data series by the average number of PWID over the study period for that series. Indices serve as a way to compare values of a variable over time by relating each value in a time series to a reference or standard value. For each year, index values were averaged across data sources to create an overall index for each year. The first set of national PWID estimates was the ratio of the overall index in a given year to that in 1992 multiplied by Holmberg’s 1992 estimate of the number of PWID [56]. The second set of estimates was the ratio of the overall index in a given year to that in 1998, multiplied by Friedman’s 1998 estimates [55]. The final set of national estimates was created by averaging the first and second set of approximations (Figure 1).

To create the national estimates for PWID for 1992–2007, we utilized three different data series to estimate the number of PWID nationwide from 1992–2007: (1) data from the CDC’s CTS which monitors the utilization of counseling and testing services in CDC-supported sites; [48], [62]; and (2) the SAMHSA’s TEDS [52] to determine the number of injectors entering treatment nationwide each year of the study period; and (3) the Uniform Crime Reporting Program County-Level Detailed Arrest and Offense time series data, produced and distributed by Inter-university Consortium for Political and Social Research (ICPSR) [63], to calculate the number of arrests between 1992 and 2007 for possession of opium, cocaine, and their derivatives (i.e., “hard” drug arrests). These data include arrests of non-injecting users and of PWID.

In 2005–07, CTS data reported lower national testing numbers which may be attributable to fewer health departments reporting test level data for those years based on a new reporting form starting in 2005. In 1999–2004 approximately 48–50 health departments were reporting test level data whereas those numbers have been decreasing 2005 (43), 2006 (34), 2007 (31). To correct for this, we predict US CTS data for year 2005–2007 using a quadratic polynomial model based on year and using years 1992–2004.

In creating U.S annual PWID estimates, we utilized the method of Brady et al [43] by first creating index values [64] for each of the three data series described above. We use the average value for the 16 years of our study period as our reference value for each data series. For example, the index value for TEDS, in a given year, is the number of PWID entering treatment in that year divided by the average number of PWID entering treatment 1992–2007, multiplied by 100. The indices for the three series were averaged across data sources to create an overall index for each year which was less sensitive to year-to-year fluctuation from each data source.

**Figure 3:**
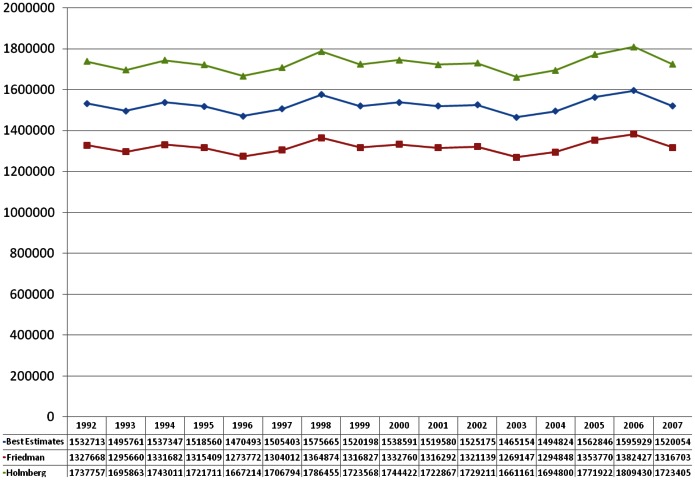
National Estimates of People Who Inject Drugs, by Index, 1992–2007.

We then multiplied the index by (1) Holmberg’s 1992 PWID estimate [56] and by (2) Friedman et al’s 1998 PWID estimate [55], thus creating two sets of national PWID estimates for each year of the study period. The final set of national estimates was created by averaging the Holmberg-based and Friedman-based ratios to calculate a “best” national PWID estimate (Figure 3).

### Step 2: Calculating MSA-Specific Estimates of PWID: 1992 to 2007

As in Brady et al. (2008) [43] and Friedman et al. (2004) [55], multiplier techniques were utilized to allocate national estimates of PWID to each MSA. To estimate the prevalence of PWID in each MSA, we calculated the number of PWID in each MSA and year from each of four data series as described in Table 1. We refer to these data series as the “component series”; the number of PWID from a given component series, MSA and year as “component series counts”; and MSA-level estimates derived from a given component series and the national estimates as its “component estimate.” Each component estimate required a unique methodological approach.

**Table 1:** Description of the four databases utilized to estimate People Who Inject Drugs.

Database	Description and Characteristics
**Counseling & Testing Services**	Individual reports on injecting at CDC HIV testing sites. Data are a count of tests not unique individuals, and thus can count an individual more than once a year
**Drug Treatment Services**	(1) UFDS/N-SSATS collects facility-level data annually from all privately and publicly funded substance abuse treatment facilities in the country, as well as from state-administered facilities; data reflect program services on October 1 of each year.
	(2) TEDS - The Substance Abuse and Mental Health Services Administration, Treatment Episode Data Set (TEDS) records data on admissions to public and private drug treatment programs that receive state funds, certificates, or licenses. An individual who enters drug treatment twice or more in a year is counted as two or more independent cases, which inflates the annual treatment entries.
**AIDS Diagnoses**	AIDS case data give the number of individuals diagnosed with AIDS as reported to CDC by state and local health departments through the CDC’s National HIV Surveillance System [51]. These data report yearly number of incident AIDS diagnoses with transmission categories noted as injection drug use or both male-to-male sexual contact and injection drug use.
**Estimates from previously published data**	This series utilizes previously published estimates by Holmberg from 1992 [44] and by Friedman for 1998 [43] for PWID per population aged 15–64 years.

1 These time series estimates of PWID are partially derived from past studies, their methods are described in detail elsewhere (see Holmberg 1993; Friedman et al 2004; or Brady et al, 2008).

#### CTS data components estimates

We use the following formula to apportion the national PWID estimate for each year to each MSA for the CTS and drug treatment-based data series independently.

Formula 1: Calculating the database-specific PWID in each MSA and year.
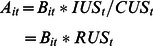
(1)where

A*_it_* – Component estimate for an MSA i in year t;

B*_it_* – Component series count for an MSA i in year t per 10,000 population 15–64 years;

IUS*_t_* – Estimated number of PWID for the entire U.S. in year t;

CUS*_t_* – Component series count for the entire U.S. in year t;

RUS*_t_* – Ratio of the estimated number of U.S. injectors to the component series count for U.S. in year t.

For the first component estimate using CTS data [48] we applied Formula 1 to allocate the number of people tested for HIV who identified injection drug use as a risk factor in the CDC’s HIV CTS data. These testing data also included men who have sex with men (MSM) and who also reported injecting drugs. For each year we allocated our national PWID estimate to MSAs by taking the number of PWID tested for HIV per population aged 15–64 years in the MSA times 10,000 and multiplying this number by the ratio of the national number of PWID to the number of PWID tested in the U.S.

#### Drug treatment data component estimates

Identical methods were used to create estimates based on drug treatment. Our second component estimate uses the drug treatment data (SAMSHA’s UFDS/N-SSATS [49], [50], [51] and TEDS [52]); we utilize Formula 1, and for each year, our national PWID estimates were allocated by taking the number of drug users (UFDS/N-SSATS) multiplied by the proportion of drug users entering treatment who injected in that MSA (TEDS) and multiplying this number by the ratio of the national number of PWID to the number of drug users in treatment in the U.S. (UFDS/N-SSATS).

#### AIDS diagnoses adjusted for HIV prevalence

Our third component series was created using data on the yearly number of incident AIDS case diagnoses [53], [65] with transmission categories noted as injection drug use or both male-to-male sexual contact and injection drug use. We have adapted these data to use time-varying estimates of HIV prevalence rates in each MSA and in the US as a whole (Appendix S1 describes in detail our methods for estimating time-varying HIV prevalence rates in each MSA and in the US as a whole among PWID for years 1992–2008). We base our AIDS component estimates on the assumption that there is a constant proportionality of HIV prevalence rate five years before to the percent of PWID who are HIV-positive and who develop AIDS. We use a five-year lag because an untreated person takes approximately 5–10 years from HIV infection to progress to AIDS infection [65], [66], [67], and because using a lag larger than five would cut into the number of years for which we can make an adjustment. The assumption of constant proportionality is counterfactual but benign for this purpose. It is benign to the extent that a large part of the variation of HIV infection and progression to AIDS is due to poor access to Highly Active Antiretroviral Treatment (HAART) and utilization of HIV counseling and testing or drug treatment services for PWID–which implies that errors in this adjustment should counter-balance errors in estimates due to changing service-levels and service encounter biases. Formula 2a thus assumes that each MSA’s share of the total number of injection-related AIDS cases diagnosed nationwide in year t equals that MSA’s share of the total number of injection-related HIV cases nationwide in year t −5.
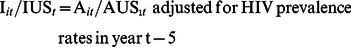
(2a)


Inserting the adjustment into equation 2a, we get:
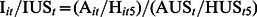
(2b)where

I*_it_* – Estimated number of PWID in the MSA i in year t;

IUS*_t_* – Estimated number of PWID in the U.S. in year t;

A*_it_* – Number of AIDS cases diagnosed in PWID in the MSA i in year t;

AUS*_t_* – Number of AIDS cases diagnosed in PWID in the U.S. in year t;

H*_it5_*– Estimated number of HIV cases among PWID in the MSA i in year t−5;

HUS*_t5_*– Estimated number of HIV cases among PWID in the U.S. in year t−5.

Solving equation 2b for the number of PWID in an MSA in a given year t gives us equation 2c.
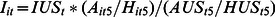
(2c)


Lastly, to complete the AIDS adjusted component estimate, for years 1992–1996, we use data calculated from Brady et al (2008) [39].

#### Previous Estimate Component Series

Our fourth and final component estimate updates and extends the 1992–2002 Holmberg-Friedman PWID estimation series for years 2003–2007. We predict data for year 2003–2007 using a polynomial regression model using quadratic and cubic measure of time based on year and using years 1992–2002. The Holmberg-Friedman PWID 19922002 estimate series were calculated by Brady and colleagues 43 by interpolating yearly MSA-specific PWID estimates based on Holmbergs 1992 56 and Friedmans 1998 55 estimates, and then extrapolating the number of PWID in each year from 19992002 by using the yearly change in PWID between 1992 and 1998 (see Brady et al (2008) for a detailed discussion of the Holmberg-Friedman PWID estimate series calculation).

#### Excluding outliers, imputing missing values, and combining component estimates to create overall metropolitan statistical area–specific people who inject drug estimates

We correct for missing values in each data series when calculating the PWID component estimates. Missing values occurred for a variety of reasons, although most arose because the source database obtained by the project lacked data on either CTS HIV testing or drug treatment entry data for specific MSAs and years under study. Thus, missing data were considered under the following circumstances:

1. In some instances, missingness arose simply because data were not collected in some of the MSAs and for years during the study period (e.g., Little Rock, AR; Honolulu, HI and San Juan, PR MSAs do not report CTS data to the CDC; Hartford, CT; New Haven, CT; Phoenix-Mesa, AZ and Tucson, AZ drug treatment centers do not report mode-of-administration, and Gary, IN and San Juan, PR do not report drug treatment admissions to SAMSHA);
2. Data were set to missing if there were unexpectedly high or low values (i.e., more than twice, or less than half, the magnitude in values of both of the adjacent years), suggesting a possible error in the data source. This accounted for 78 observations in CDC’s CTS data and five observations in the drug treatment data set of the overall 1,536 observations; and
3. Data were set to missing based on the exclusion criteria. We applied plausible bounds to the four component series and any values falling outside set bounds were set to missing and imputed. We excluded component estimates that fell outside the plausible bounds for a value. Component estimates were bounded between 15 and 696 PWID per 10,000 population aged 15–64 years. These lower and upper bounds were set by taking 1/2 of the minimum and twice the maximum number of PWID per 10,000 population aged 15–64 years from any MSA estimate from Holmberg’s 1992 and Friedman’s 1998 PWID estimates for MSAs. The CTS and drug treatment component series had only minimal data that fell outside the plausible bounds; and no values from the AIDS component estimate or the component estimates interpolated and extrapolated from estimates for 1992 and 1998) fell outside the plausible range. These exclusion criteria resulted in the exclusion of 127 component estimates: Seventy-nine drug treatment-based values were excluded because of low values; forty-eight low values based on counseling and testing were excluded. These 127 values were treated as missing data.

When data were missing for any of these reasons, we imputed a value for this component equal to the average of the values for the non-missing components. For this, single linear regression imputation was used to estimate the predicted value of these observations. For each component series, missing values were predicted by the other component series separately. The missing values were replaced with imputed predicted values for that MSA based on regression models results for each component series with non-missing.

#### Smoothing and averaging component estimates to create final PWID estimates

The values of each of the component estimates for each MSA across time were smoothed using loess regression, which fits curves to noisy data and smoothes data in a manner similar to computing a weighted moving average on time series data.

The predicted values of the component estimates resulting from the loess regressions for each MSA and year were averaged to create our final estimates (Appendix S2). We chose a smoothing coefficient, between 0.4 and 1 by intervals of 0.1, for each MSA based on the smallest AICC1 criterion [68]. Statistical analyses were performed by using SAS software, version 9.2 [69].

### Calculating MSA-Specific PWID Subpopulation Prevalence: 1992 to 2007

The following sections describe our methods for calculating the proportion of PWID in three subpopulations across MSAs and year. We stratify each three subpopulation into specific groups: (1) sex (male and female); (2) age (youth 15–29 years and older 30–64 years); (3) and race/ethnicity (non-Hispanic white, non-Hispanic black, and Hispanic). Figure 2 describes our methods and procedures for calculating the subpopulation prevalence estimates. We further assess the reliability and validity of both the overall PWID estimates and PWID subpopulation estimates, and discuss trend results for both.

Our estimates of MSA-specific subpopulation prevalence for PWID are built on previous methods and estimates by Cooper et al, (2008) [44], Pouget et al (2012) [45], and Chatterjee et al, (2011) [46] although no previous prevalence estimates exist for men and women who inject drugs. The subpopulation prevalence estimates utilize our current PWID estimates as described above. The proportions of PWID who were in each subpopulation were estimated by combining three data series: (1) CDC CTS [48]; (2) SAMSHA TEDS [52]; and (3) CDC’s data on PWID diagnosed with AIDS [53]. The CTS data had missing values for four of the 96 MSAs for the entire study period (Honolulu, HI, Little Rock–North Little Rock, AR, San Juan-Caguas-Arecibo, PR, and Springfield, MA). We also excluded existing data for one to six years from 14 MSAs in CTS in order to avoid proportion estimates that could be unreliable due to small total numbers of PWID (fewer than 20). In addition, data on proportions of each subpopulation were available from at least 86 MSAs in each year from the TEDS treatment data series. Lastly, AIDS data were not reported according to racial/ethnic category in some states. This included two MSAs (Cincinnati, OH–KY–IN and Louisville, KY–IN) within our study boundaries.

To reduce potential bias due to variation among MSAs in relative HIV prevalence, we adjusted the proportion of PWID who were in each subpopulation from the AIDS data (P*_AIDS_*) for HIV prevalence among PWID calculated from CTS data (see Appendix S1 for adjustment formula). Database-specific estimates were combined by using predicted values from a binomial mixed-effects regression to produce MSA-specific rates.

To estimate subpopulation PWID prevalence we multiplied yearly estimates of the proportion of PWID who were in the subpopulation (averaging proportions from three data series) in each MSA by yearly estimates of the total number of PWID in each MSA, and divided by their respective populations. The age range used for population subpopulation denominators was 15–64, except for estimates of young PWID, where the 15–29 age range is applied. The calculation is summarized below.
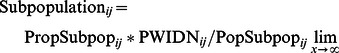
(3)where

Subpopulation *_ij_* = Estimated prevalence of PWID among subpopulation residents in study year i, MSA j in database k.

PropSubpop*_ij_* = Estimated proportion of PWID who were in the subpopulation in study year i, MSA j in database k.

PWIDN*_ij_* = Estimated total number of PWID, aged 15–64, in study year i, MSA j in database k.

PopSubpop*_ij_* = Number of residents aged 15–64 who were in the subpopulation, in study year i, MSA j and database k.

#### Imputations to estimate missing HIV test results data for subpopulations estimates

Missing data for calculating subpopulations estimates were imputed separately. CTS data on the proportion testing positive for HIV among PWID subpopulations were incomplete due to inconsistent reporting, removal by the CDC of testing results from small cells (fewer than 5 positive results) to protect participant confidentiality, and exclusion of data on the proportion positive that would have been based on fewer than 20 PWID.

Missing CTS data for computing the proportion testing positive for HIV among PWID subpopulations were imputed in two steps. First, in a binomial mixed-effects regression, values were imputed as a function of: (a) cubic polynomial terms for time; (b) the proportion of total PWID testing positive for HIV; and (c) the proportion of PWID tested who were in each subpopulation.

Mixed-effects regressions were performed using the SAS procedure PROC GLIMMIX [69]. Intercepts and time parameters were set to vary randomly using residual pseudo-likelihood estimation. Next, in MSAs where PWID subpopulation HIV data were missing, we imputed missing values using predicted values from a mixed-effects Poisson regression using a cubic polynomial model of time. We imputed values to fill in missing CTS HIV data for total PWID in a parallel manner, using year, the proportion of all CTS clients testing positive for HIV, and the proportion of CTS clients tested who were PWID as predictors for the mixed-effects binomial regression. Thirteen MSAs had missing data for all study years; as a result we replaced missing cells with the average values as predicted by the binomial regressions.

#### Averaged proportions of PWID in each subpopulation

We created a complete data set of the proportions of PWID who were in each subpopulation from the three data sources–CTS, TEDS and AIDS (adjusted for HIV prevalence)–using predicted values from a binomial mixed-effects regression, with one exception: in the San Juan-Caguas-Arecibo, PR MSA we assume that all of the PWID were Hispanic because CTS data were not available, TEDS data were not collected by race/ethnicity, and AIDS data indicated that few PWID with AIDS were non-Hispanic).

Data were stacked so that each MSA and year was represented by as many observations as there were data sources (1, 2, or all 3 sources). The model used all the available data to estimate parameters, even where some data were missing, under the assumption that the data were missing at random, conditional on the observed data. Dummy codes were used to compare data sources. We used a cubic polynomial model of time to maintain consistency with procedures used to produce the overall PWID estimates. Intercepts and time parameters were set to vary randomly using residual pseudo-likelihood estimation. The resulting complete sets of proportions were averaged to create single yearly estimates of the proportion of PWID who were in each subpopulation. These proportions were then multiplied by estimates of the number of total PWID to produce estimates of the PWID subpopulations. The resulting numbers of PWID in each subpopulation were divided by each subpopulation, respectively, to produce subpopulation prevalence rates.

### Testing Reliability of PWID and PWID Subpopulation Estimates

To test the reliability of our overall PWID estimates (i.e., the extent to which our component estimates seem to be capturing the same underlying construct), we examined the correlation between each of our component estimates for each year. These correlations describe the extent to which our component estimates produce similar results.

To assess the reliability of our subpopulation estimates were compared the proportions of PWID in each subpopulation across sources of data. We used CTS, TEDS, and AIDS data for racial/ethnic subpopulation estimates. For estimating sex and age PWID subpopulations we used CTS and TEDS data only because AIDS data showed less consistency and produced weak correlations with the subpopulation proportions in CTS and TEDS data. As such, we considered the AIDS data less reliable as a measure for these specific subpopulations estimates. By definition, young PWID have less time to become HIV-infected and progress to AIDS, and thus we expect low correlations because AIDS data among youth are sparse, reflecting less time-at-risk to develop AIDS among youth. Additionally, HIV is generally found to be lower among females, which reflect less stable numbers of AIDS diagnosis [1], [70], [71].

### Testing Validity of PWID and PWID Subpopulation Estimates

To assess the validity of the overall PWID prevalence estimates, and the PWID subpopulation estimates we compared these with mortality related to drug-poisoning and HCV mortality. A minimum of three deaths was used as a criterion for calculating subpopulation mortality rates. We do not present HCV mortality analyses for young PWID since they would have had less time to develop fatal liver disease caused by HCV acquired by injecting drugs. Additionally, numerous studies have found that economic conditions are associated with rates of substance use and/or HIV prevalence [32], [43], [54], [55], [61]. Thus, we further utilize a number of socioeconomic metropolitan area characteristics, including income inequality, related to the subsequent prevalence of drug injection in the population.

#### Identifying drug-poisoning mortality indicators

We include drug-poisoning mortality as a key validator due to the high rates of overdose deaths that occur among drug-using populations. For example, in the U.S., drug overdose is the second leading cause of unintentional injury death behind motor vehicle crashes, but is the leading cause of injury death among persons 35 to 54 years of age [44]. We refer to drug-poisoning mortality as those deaths that are directly caused by an accidental or unintentional drug-poisoning death. Since injection drug use cannot be determined in the multiple causes of death files [72], [73], [74], [75], for both ICD-9 [72] and ICD-10 [73], [74], [75] coding, we included a range of substances that correspond to injection practices. For the ICD-9, we had included a more expansive list of substances; however, for ICD-10 coding, we decided to adapt our coding scheme to include only opioids (heroin, methadone, opium, other opioids, and other synthetic narcotics), cocaine and psychostimulants. The motivation for this change was both a desire to simplify the coding scheme and to reduce inconsistencies in case definitions inherent in trying to identify overdose deaths related to injection drug use. Drug-poisoning deaths were included if the MSA in which the death occurred matched one of our study MSAs. Our algorithm for drug-related mortality is adapted from the European Monitoring Centre for Drugs and Drug Addiction (EMCDDA) ICD-9 and ICD-10 coding scheme [76], [77].

#### Identifying HCV virus mortality indicators

HCV virus (HCV) was selected as a validator because it is a blood-borne infection most commonly transmitted in the U.S. via PWID [78]. The HCV was first identified in 1989 and widespread screening and testing of the virus began in 1993. Thus the ICD-9 coding conventions used to identify HCV might result in an underestimation of the contribution of HCV to mortality in the early years of the study period [79], [80], [81], [82]. We utilize the ICD-10 coding [73], [74], [75] scheme to identify HCV mortality, defined as deaths recorded as either acute (B171) or chronic (B182) HCV [78]; accordingly the correlations values only reflect data from 1999 to 2007.

### Studying Temporal Trends

We analyze trends over time for overall PWID prevalence rates and subpopulation prevalence rates by utilizing mixed-effects polynomial models with PROC MIXED using linear, quadratic and cubic terms for time [68]. We centered time at the mid-point of the study period, year 2000. Nested models were compared using likelihood ratio tests [69].

#### Sample and its implications for statistical analyses

This is a study of 96 MSAs that are the largest metropolitan areas in the U.S. Therefore, it was a study of a population rather than of a sample. This means that there was no sampling error (although there was measurement error). Statistical analyses are primarily descriptive rather than inferential. Thus, the use of p values in this analysis is a guide to the relative “importance”, and not the significance of possible associations. Some researchers conducting studies with similar populations use P values as a heuristic device to avoid over-interpreting model parameters [83], [84], [85] (We refer to these as pseudo-Ps.) Other analysts would consider the population to be a random sample of “possible universes”; under this interpretation, pseudo-Ps have a probabilistic interpretation.

## Results

### Reliability and Validity of the PWID and PWID Subpopulation Prevalence Estimates Reliability and Validity of the PWID Prevalence Estimates

**Figure 4:**
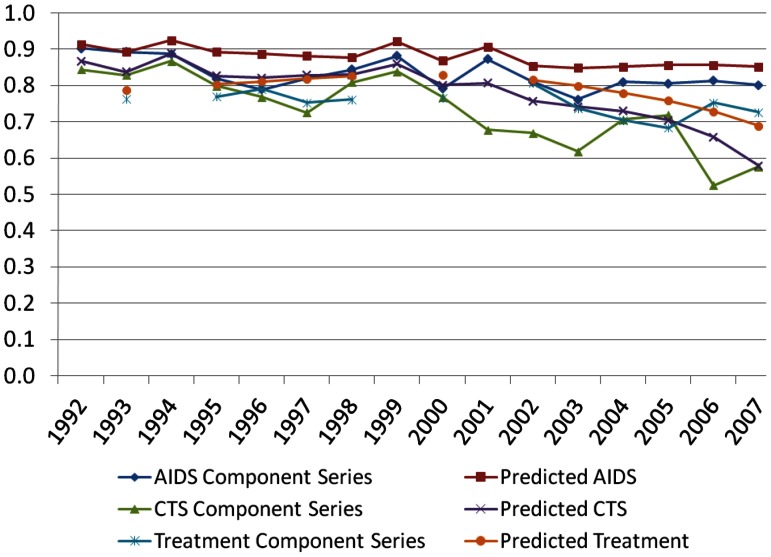
Intercorrelations between Component Series Estimates, 1992–2007.

We assess the reliability of our PWID estimates by comparing each component series estimate across data sources. Figure 4 presents the correlations across MSAs of the component series estimates by year for the overall PWID prevalence estimates. In general, the intercorrelations were moderately strong and mostly stable over time, which is expected because the component estimates aim to measure the same underlying construct. As expected CTS and TEDS component series correlate more strongly with each other than with AIDS component series, since counseling and testing, and drug treatment represent services that can help to prevent HIV infection or delay progression to AIDS for those who are infected. These was an overall declining correlation between the drug treatment counseling and testing and AIDS-based component estimates, and to some extent, between the counseling and testing and AIDS-based component estimates which may suggest that the performance of the AIDS adjustment formula that relates HIV prevalence to AIDS cases deteriorates over time, or that our estimates are affected by service bias to a greater extent over time.

Convergent validity was assessed by evaluating if the PWID prevalence estimates correlated with theoretically relevant predictors, including unemployment, drug-poisoning mortality and mortality from HCV. Table 2 displays the cross-sectional correlations between PWID prevalence and theoretical constructs. Correlations between PWID prevalence and unemployment varied little over time ranging from 0.42 in 1992 to 0.35 in 2003 where correlations declined starting 2004. Correlations between PWID prevalence and drug- poisoning mortality held steady over time ranging from 0.51 in 1992 to 0.40 in 2003– correlations declined starting 2004. Correlations between PWID prevalence and HCV mortality were consistent across years, ranging from 0.48 in 1999 to 0.43 in 2007.

**Table 2:** PWID Validity: Pearson correlations between PWID prevalence estimates and theoretically related constructs over time.

Year	Unemployment Rate	Unemployment with 2 year lag	HCV Deaths^2^	Drug-Poisoning Deaths^3^
**1992**	0.42**	–	–	0.51**
**1993**	0.54**	–	–	0.61**
**1994**	0.50**	0.44**	–	0.51**
**1995**	0.57**	0.54**	–	0.58**
**1996**	0.56**	0.55**	–	0.63**
**1997**	0.54**	0.57**	–	0.60**
**1998**	0.54**	0.55**	–	0.54**
**1999** 1	0.50**	0.53**	0.48**	0.44**
**2000**	0.47**	0.51**	0.46**	0.40**
**2001**	0.43**	0.47**	0.52**	0.39**
**2002**	0.38**	0.43**	0.51**	0.34**
**2003**	0.35**	0.37**	0.51**	0.40**
**2004**	0.28**	0.35**	0.45**	0.25*
**2005**	0.23*	0.33**	0.41**	0.24*
**2006**	0.21*	0.28*	0.40**	0.27*
**2007**	0.18	0.23*	0.43**	0.29*

** pseudo- p<0.001;

* pseudo -p<0.05.

1 ICD-10 implemented in 1999:

2 ICD-10 codes for HCV are more accurate so earlier data are not reported;

3 2001 California MSAS set to missing due to inaccurate reporting.

NOTE: Construct variables are calculated as per 10, 000 population.

Additionally, correlations of the estimate of the prevalence of PWID with theoretically related indicators, such as percent living in poverty and income inequality were analyzed. The correlations between percent in poverty in 1990 and our PWID estimates per 10,000 population in 1992; between poverty in 2000 and our PWID estimates per 10,000 population in 2000 were 0.33 and 0.36 respectively; and finally between income inequality 1990 and 2000 and our 1992 and 2000 PWID estimates were 0.24 and 0.23, respectively (each of these is significant at p = 0.05 level). This suggests that for the given years our estimates were similarly accurate to those estimates presented by Brady and colleagues (2008) [43].

### Reliability and Validity of the PWID of Subpopulation Estimates

Pearson correlations values for the subpopulation estimates are presented in Table 3. Reliability analysis on the subpopulation proportions among the data series showed good consistency for most comparisons as well. CTS and TEDS proportions were moderately correlated for young PWID and for male PWID, and correlated somewhat less for female PWID. For all three racial/ethnic groups, CTS and TEDS proportion data correlated more strongly with each other than with AIDS proportions.

**Table 3:** PWID Subpopulation Reliability: Pearson correlations between proportions estimated among CTS, TEDS, and AIDS data adjusted for HIV prevalence based on CTS test results.

	Non-Hispanic White			Non-Hispanic Black			Hispanic			Female	Young(15–29)
Year	C-T^1^	C-A^2^	T-A^3^	C-T^1^	C-A^2^	T-A^3^	C-T^1^	C-A^2^	T-A^3^	C-T^1^	C-T^1^
**1992**	0.68	0.51	0.35	0.76	0.60	0.56	0.92	0.72	0.69	0.36	0.49
**1993**	0.59	0.45	0.33	0.70	0.56	0.61	0.92	0.83	0.79	0.51	0.54
**1994**	0.64	0.62	0.47	0.73	0.61	0.57	0.92	0.89	0.85	0.40	0.27
**1995**	0.69	0.55	0.44	0.84	0.64	0.61	0.90	0.90	0.81	0.36	0.35
**1996**	0.71	0.58	0.36	0.81	0.61	0.52	0.91	0.78	0.85	0.14	0.31
**1997**	0.74	0.51	0.29	0.82	0.54	0.57	0.91	0.84	0.87	0.31	0.46
**1998**	0.77	0.56	0.37	0.83	0.55	0.49	0.91	0.82	0.88	0.26	0.64
**1999**	0.68	0.51	0.38	0.74	0.57	0.46	0.89	0.77	0.79	0.21	0.62
**2000**	0.60	0.49	0.36	0.77	0.51	0.47	0.89	0.82	0.82	0.20	0.63
**2001**	0.62	0.43	0.27	0.72	0.61	0.45	0.90	0.79	0.69	0.15	0.52
**2002**	0.62	0.44	0.22	0.72	0.46	0.21	0.89	0.80	0.74	0.44	0.55
**2003**	0.68	0.49	0.32	0.69	0.51	0.41	0.88	0.75	0.76	0.55	0.51
**2004**	0.74	0.46	0.39	0.80	0.60	0.49	0.92	0.85	0.82	0.49	0.45
**2005**	0.74	0.29	0.24	0.75	0.45	0.48	0.93	0.76	0.73	0.39	0.42
**2006**	0.67	0.41	0.23	0.58	0.38	0.39	0.88	0.77	0.73	0.25	0.43
**2007**	0.69	0.32	0.22	0.60	0.49	0.59	0.91	0.77	0.69	0.16	0.48

1 CTS-TEDS;

2 CTS-AIDS;

3 TEDS-AIDS.

Female and male PWID proportions in CTS and TEDS data may not as accurately represent the proportion of females and males among PWID in the MSA populations relative to proportions for the other subpopulations. Weak correlations found for female PWID subpopulation may reflect the fact that female PWID are much more likely to be stigmatized by society than their male counterparts because drug use violates social norms of behavior as it diverges from the traditional expectations of women as nurturers of families [86, 87). As such, female PWID may be more inclined to conceal injecting behavior and hesitant to utilize counseling and testing or drug treatment services [88], [89], [90], [91], [92].

Subpopulation validity was assessed by comparing subpopulation PWID prevalence rates with rates of drug-poisoning deaths and HCV deaths series showed moderate to strong associations for most comparisons (Table 4). Correlations with HCV deaths begin in 1999, when ICD-10 implemented explicit coding for HCV. Across subpopulations, correlations between PWID rates and drug-poisoning deaths were higher in earlier years. Correlations averaged 0.52 for drug-poisoning deaths from 1992–1999, and 0.35 for 2000–2007. Correlations with HCV deaths were 0.30 or above for all subpopulations in all years, averaging 0.50 across subpopulations. Correlations with PWID subpopulations and drug-poisoning deaths generally were reduced over time. Some values were below 0.20 for Hispanic and male subpopulations in some years. We conjecture that the downward trend in these correlations may reflect an increase in the contribution of non-injecting opioid analgesic users to drug-poisoning deaths over the study period [93].

**Table 4:** PWID Subpopulation Validity: Pearson correlations between subpopulation PWID prevalence estimates and theoretically related constructs over time.

	Non-Hispanic White		Non-Hispanic Black		Hispanic		Male		Female		Young (15–29)	Old (30–64)	
Year	DP^1^	HCV^2^	DP^1^	HCV^2^	DP^1^	HCV^2^	DP^1^	HCV^2^	DP^1^	HCV^2^	DP^1^	DP^1^	HCV^2^
**1992**	0.65	–	0.51	–	0.48	–	0.45	–	0.44	–	0.34	0.53	–
**1993**	0.71	–	0.60	–	0.48	–	0.54	–	0.54	–	0.41	0.61	–
**1994**	0.62	–	0.61	–	0.51	–	0.46	–	0.29	–	0.47	0.46	–
**1995**	0.72	–	0.63	–	0.63	–	0.55	–	0.42	–	0.40	0.54	–
**1996**	0.71	–	0.70	–	0.38	–	0.55	–	0.64	–	0.33	0.60	–
**1997**	0.70	–	0.72	–	0.64	–	0.56	–	0.46	–	0.55	0.55	–
**1998**	0.67	–	0.50	–	0.33	–	0.48	–	0.53	–	0.47	0.53	–
**1999**	0.58	0.56	0.46	0.66	0.33	0.66	0.35	0.43	0.49	0.45	0.44	0.44	0.48
**2000**	0.54	0.53	0.60	0.60	0.39	0.61	0.32	0.36	0.42	0.45	0.33	0.40	0.49
**2001**	0.40	0.58	0.23	0.56	0.27	0.80	0.21	0.47	0.31	0.35	0.42	0.22	0.55
**2002**	0.45	0.56	0.51	0.70	0.28	0.53	0.26	0.41	0.42	0.57	0.27	0.38	0.53
**2003**	0.51	0.60	0.46	0.41	0.38	0.64	0.36	0.45	0.34	0.38	0.42	0.41	0.57
**2004**	0.43	0.43	0.40	0.62	−0.04	0.33	0.15	0.42	0.33	0.38	0.36	0.28	0.49
**2005**	0.34	0.50	0.61	0.60	0.23	0.54	0.19	0.33	0.29	0.42	0.36	0.23	0.44
**2006**	0.40	0.48	0.46	0.52	0.22	0.47	0.22	0.33	0.37	0.37	0.36	0.30	0.52
**2007**	0.43	0.55	0.53	0.50	0.25	0.57	0.24	0.32	0.36	0.52	0.34	0.30	0.52

1 Drug Poisoning Deaths;

2 HCV Deaths (beginning in 1999).

### Trends in PWID Prevalence and PWID Subpopulation Prevalence Estimates

Appendix S2 shows the estimates of the number of PWID per 10,000 population aged 15–64 years for each of the 96 largest MSAs in the US for each year from 1992 to 2007. Mean PWID prevalence rates and standard deviations are presented in Table 5. Overall, the number of PWID per 10,000 persons aged 15–64 years varied from 31 to 345 across MSAs, median 104.4 (mean 127.4; standard deviation 66.7; percentile range 76–162) in 1992 and from 34 to 324 across MSAs, median 91.5 (mean 103.6; standard deviation 56.4; percentile range 61–125 ) in 2007 indicating an overall decline in PWID prevalence across MSAs.

**Table 5:** Mean PWID and subpopulation PWID prevalence rates, 1992–2007.

	PWID	Non-Hispanic White	Non-Hispanic Black	Hispanic	Male	Female	Young (15–29)	Old (30–64)
Year	Mean (SD)	Mean (SD)	Mean (SD)	Mean (SD)	Mean (SD)	Mean (SD)	Mean (SD)	Mean (SD)
**1992**	127.38 (66.73)	96.51 (62.85)	337.03 (191.43)	183.74 (184.07)	170.47 (92.46)	85.28 (47.63)	101.64 (62.00)	142.47 (73.64)
**1993**	119.61 (64.68)	90.89 (60.33)	310.38 (192.55)	169.86 (173.09)	158.56 (85.24)	81.77 (48.72)	93.58 (55.89)	133.78 (73.68)
**1994**	121.63 (60.95)	92.30 (57.02)	300.98 (164.69)	166.51 (176.26)	160.84 (82.43)	83.93 (45.44)	94.91 (54.14)	135.35 (68.59)
**1995**	114.87 (59.83)	87.35 (54.32)	276.32 (167.84)	156.31 (169.20)	150.77 (78.23)	80.67 (46.12)	90.57 (50.92)	127.44 (69.49)
**1996**	112.61 (57.99)	85.96 (52.10)	259.71 (159.26)	149.84 (166.52)	147.19 (75.69)	80.01 (45.05)	90.50 (49.50)	124.38 (68.17)
**1997**	110.49 (56.89)	84.82 (50.67)	244.05 (154.40)	144.44 (165.07)	143.91 (74.22)	79.31 (44.41)	91.32 (49.15)	121.32 (67.60)
**1998**	108.95 (56.47)	84.32 (50.30)	230.87 (153.89)	139.94 (163.30)	141.49 (73.70)	78.90 (44.26)	93.35 (50.17)	118.67 (67.54)
**1999**	110.80 (56.23)	87.09 (52.12)	223.61 (147.42)	137.84 (163.44)	143.42 (73.56)	80.86 (44.38)	99.35 (53.69)	119.12 (66.76)
**2000**	106.71 (56.02)	84.57 (50.75)	210.75 (162.29)	132.05 (157.20)	137.87 (73.58)	78.32 (43.94)	100.38 (54.76)	113.10 (66.90)
**2001**	109.95 (57.16)	88.82 (54.78)	209.87 (168.06)	131.53 (155.33)	141.48 (74.50)	81.30 (45.53)	108.82 (59.72)	114.26 (67.77)
**2002**	105.02 (55.64)	85.31 (51.14)	198.49 (177.16)	126.82 (151.29)	135.20 (73.67)	77.59 (43.33)	108.30 (58.75)	107.09 (65.82)
**2003**	104.56 (55.70)	86.15 (51.44)	195.92 (185.83)	125.01 (148.72)	134.55 (74.15)	77.17 (42.96)	111.53 (60.07)	104.38 (65.58)
**2004**	104.36 (55.83)	87.26 (51.82)	194.94 (192.54)	123.61 (145.84)	134.16 (74.72)	76.79 (42.56)	113.96 (61.00)	101.88 (65.64)
**2005**	104.08 (55.78)	88.50 (52.33)	196.03 (198.56)	121.52 (140.53)	133.67 (74.85)	76.14 (42.04)	115.19 (61.29)	99.10 (65.47)
**2006**	103.89 (55.96)	89.71 (53.02)	200.18 (208.74)	119.13 (133.70)	133.33 (75.22)	75.36 (41.62)	124.62 (66.06)	96.20 (65.34)
**2007**	103.65 (56.36)	90.88 (54.18)	209.69 (229.77)	117.31 (127.69)	132.94 (75.82)	74.30 (41.33)	126.67 (68.20)	92.89 (65.24)

**Figure 5:**
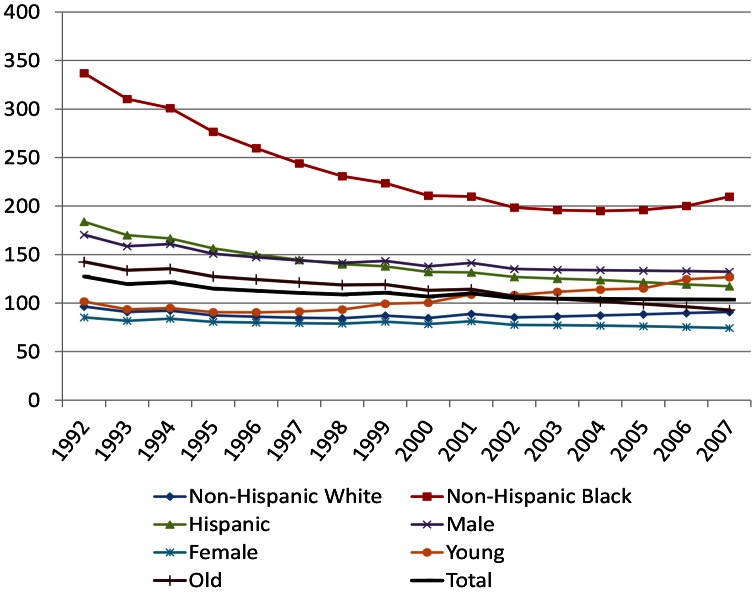
People Who Inject Drugs Prevalence Rates per 10,000 population: PWID Total and Subpopulations, 1992–2007.

Figure 5 shows the overall trajectory of the PWID prevalence rates based on the multilevel model. Trend analysis of the overall results is consistent with a decline in the early study period, followed by an increase in 2000–02, and then remaining stable thereafter over time. On average there has been very little change since 2002 (mean 105.0) to 2007 (mean 103.6). Overall, across the 96 MSAs the mean PWID prevalence mostly decreased during our study period, as did the dispersion of estimates over time.

Trend analysis results for the overall PWID prevalence are in Table 6. The linear model indicates the average number of PWID across MSAs was about 110 PWID per 10,000 population age 15–64 years in year 2000 where each year from 2000, the average number of PWID across MSAs declined slightly by about 1.4 per 10,000 population. Quadratic and cubic terms are also presented. Overall linear and quadratic terms for PWID were significant; the somewhat smaller linear decline was offset over time by the quadratic term. Although the cubic term in Model 3 was not significant, it significantly improved the fit of the model. All models indicate a decline in PWID prevalence, increasing in years 2000–02; thereafter, the trend indicates our PWID prevalence rates stabilize after 2002 and vary little through the end of the study period.

**Table 6:** PWID prevalence trend analysis with linear, quadratic and cubic models of time.

	Model 1	Model 2	Model 3
**Intercept**	109.58**	107.08**	107.27**
**Linear (years since 2000**	−1.37**	−1.25**	−0.89**
**Quadratic (years since** **2000 squared)**	–	0.20**	0.10**
**Cubic (years since 2000 cubed)**	–	–	−0.01

** pseudo-p<0.001;

* pseudo-p<0.05.

**Note:** To minimize potential multicollinearity time is centered at the year 2000. Model 3 is the best model by likelihood ratio test.

We calculated percent change values of the earliest (1992–1994) and latest (2005–2007) 3 years of data. MSAs that experienced increased rates in PWID prevalence reside mainly in the northeast (Baltimore, MD; Springfield, MA; Boston, MA–NH; Providence–Fall River–Warwick, RI–MA; Harrisburg–Lebanon–Carlisle, PA). Sarasota–Bradenton, FL; Stockton–Lodi, CA; Youngstown–Warren, OH; Salt Lake City–Ogden, UT; Tulsa, OK MSAs also experienced increases in PWID prevalence rates over time.

Table 7 shows the results of test for trends over time for each subpopulation and are also graphically displayed in Figure 5. The intercept column represents the modeled subpopulation PWID prevalence rate in the year 2000. All intercepts were significantly different from zero. For non-Hispanic white, only the quadratic term for time was significant, in the positive direction, reflecting a small increase since 2000 (7.5% in total, or an average of 1.1% per year). For the non-Hispanic black subpopulation, the linear and quadratic terms were significant; the strong linear decline seen in the early 1990s was offset over time by the smaller quadratic term, reflecting an increase in each year since 2004 (0.6% in 2005, 2.1% in 2006 and 4.8% in 2007). For the Hispanic subpopulation, linear and quadratic terms were also significant; the somewhat smaller linear decline was also offset over time by the quadratic term, averaging a yearly decline of 4.7% in 1993–1997, 2.6% in 1998–2002, and 1.5% in 2003–2007. For the male subgroup, linear and quadratic terms were also significant, with a more modest linear decline offset by the quadratic term, reflecting an average yearly decline of 2.6% in 1992–2000, and 0.5% in 2001–2007. For the female subpopulation, no time effects were significant.

**Table 7:** PWID subpopulations trend analysis with linear, quadratic and cubic models of time.

	Intercept	Linear	Quadratic	Cubic
**Non-Hispanic White**	85.17	0.32	0.13**	−0.01
**Non-Hispanic Black**	211.80	−8.38**	1.01**	0.02
**Hispanic**	132.73	−3.06**	0.24**	−0.02
**Male**	138.46	−1.41**	0.17**	−0.02
**Female**	78.90	−0.28	−0.01	−0.01
**Young (15–29)**	100.64	3.52**	0.18**	−0.03*
**Old (30–64)**	113.25	−2.68**	0.04	−0.01

** pseudo-p<0.001;

* pseudo- p<0.05.

Overall, the number of PWID per 10,000 persons aged 15–64 years varied from 31 to 345 across MSAs in 1992 (mean 127.4) and from 34 to 324 across MSAs in 2007 (mean 103.7). Trend analysis indicates that declines during the early period (1992–2002) leveled off, and rates were relatively stable in 2003–2007. Mean rates for non-Hispanic black PWID were the highest of all racial/ethnic groups in 1992 (337.0). After an early steep decline in the mean, non-Hispanic black PWID prevalence leveled off in 2000, and on average rates increased after 2005. Rates for non-Hispanic whites increased on average since 2003, although the rate was only about half that of non-Hispanic blacks. Hispanic PWID prevalence, in contrast, has consistently declined, although the decline has slowed across time. Mean male PWID prevalence shows a decline that decreased over time. Mean female prevalence shows no clear trend over time, although mean values since 2000 are somewhat lower than those before 2000.

Most notably, our results indicate an overall increasing trend in young PWID. For the young subpopulation, linear, quadratic and cubic terms were significant; positive linear and quadratic increases were offset with a negative cubic term over time, reflecting a general increase since 1996. After decreasing on average by 11.0% from 1992–1996 (2.8% per year), the average rate of young PWID increased by 40.0% from 1996–2006 (3.1% per year). In contrast, for older PWID, only the linear term was significant, reflecting a continuous linear decline.

## Discussion

Our national estimates indicate that PWID prevalence remained relatively stable from 1992 to 2007 nationwide. The overall MSA-level population prevalence of PWID decreased through the 1990s (as reported in Brady (2008) [43]). Trend analysis showed an overall decline in the early study period, followed by an increase in 2000–02; thereafter PWID prevalence rates remained stable. Trends among subpopulation prevalence rates varied over time. Importantly, results show a worrisome trend in young PWID prevalence since HAART was initiated – the average increase across MSAs for 1996 to 2007 was 35%.

Our validation test showed relatively high to modest correlations between each series of MSA-level component estimates across years, suggesting that the component estimates measure the same underlying construct and that our data series support each other (Figure 4). Multiple sources of data on PWID showed consistency, and comparisons with data on drug-poisoning mortality and HCV mortality supported the validity of the estimates.

PWID prevalence among MSA residents aged 30 and older closely matched PWID prevalence overall. However, prevalence was slightly higher among older MSA residents from 1992–2002, and lower from 2003–2007, reflecting a decreasing average age of older PWID. Overall, declining PWID prevalence rates among those 30 and above may stem from increases in overdose and/or HCV mortality and from age-associated mortality in aging cohorts of PWIDs. Another explanation for the downward trend in PWID prevalence in metropolitan areas is that drug users have been employing alternative modes of drug administration, or discontinue drug use in general. There are numerous reasons to choose modes of administration other than injection and discontinue use, including fear of HIV or HCV.

This might be balanced in some MSAs due to declines in HIV-related mortality due to improved treatments and decreases in HIV-associated mortality in MSAs where HIV among PWID is prevalent. Drug treatment programs or other processes may have led some PWIDs to stop drug use or at least injecting. In addition, the rising use of methamphetamine and prescription drugs, which are often administered by means other than injection, may mark a shift away from injection drug use. Increases in non-injection opioid use may have influenced changes in PWID prevalence in contradictory ways, perhaps lead some youth not to inject their drugs but leading others to start injecting [8].

Prevalence rates for young PWID are consistent with previously published estimates [46], and confirm and extend the previously observed increase that began in 1996. There are at least two potential explanations for this increase. First, young MSA residents may have avoided injecting in the early 1990s due to the well-publicized risks of HIV/AIDS, and morbidity and mortality associated with injecting drugs. Potentially, this reluctance may have been reduced over time since the HAART era due to HIV treatment optimism, similar to explanations some have hypothesized for increased sexual risk behavior among MSM [94], [95].

Moreover, recent increases in non-medical use of prescription opioids may have led some new opioid users to inject prescription opioids or transition to heroin or methamphetamine injecting [96], [97]. A recent study by Jones (In Press) [98], found that over 77% of people using both prescription opioid and heroin in the past year report initiating a prescription opioid prior to heroin initiation. Among the three age groups examined in the study, 18–25 year olds had the highest rate of past year heroin use – of those in this age category 12.3% also used heroin in the past year, a 95% increase compared to 2002–2004. Additional studies by Church et al., (2011) [99] and Stanley et al., (2012) [100] reportedly linked new HCV infections in young adults to the use of prescription opioids and transition to heroin or methamphetamine injection.

If young PWID engage in high levels of risk behavior, this could lead to widespread increases in HIV transmission that could parallel recent increases in HIV incidence among young MSM. Young MSM increasingly assume the heaviest burden of HIV infection, presenting more than half of new infection in 2010 [1], [2]. A critical component of risk among young MSM is the intersection of the HIV epidemic, substance use (which includes both injection drug use risk and non-injection risks), and unprotected sexual activity with multiple partners [101], [102], [103]. The increasing practice of injecting methamphetamine among MSM leads to greater risk of HIV, STIs and hepatitis, offering new challenges for prevention and reducing harm among MSM injectors [102], [103], [104].

An early steep decline in non-Hispanic black PWID prevalence leveled off, and on average, rates increased since 2005. Mean rates for non-Hispanic black PWID were the highest in 1992 (mean 337.0), and lowest in 2004 (mean 195.0). Although representing less than 14% of the total population in the US, non-Hispanic blacks are disproportionate affected by HIV–accounting for 44% of HIV infection for 2010 [1], [2]. Recent increases in non-Hispanic black PWID prevalence warrant further investigation, given that the average annual rate of new HIV infection diagnosis per 100,000 population during 2004–2007 was 11.0 for non-Hispanic black PWID, 4.9 per 100,000 for Hispanic PWID, and 0.9 per 100,000 for non-Hispanic white PWID [1], [10]. As such, HIV infection among PWID subpopulations continues to be a public health issue – where non-Hispanic blacks are disproportionately affected by HIV [1], [2].

Knowledge of these findings can assist research that investigates the causes and consequences of increasing PWID prevalence among subpopulations, including how risk exposures differ among subpopulations. For example, young injectors may be less aware of the dangers of injecting drugs and how to reduce their risk, and as such, more likely to share syringes and drug preparation equipment increasing their risk of HIV, hepatitis B and C, as well as fatal overdose and homicide [96], [97], [98], [100], [105], [106], [107]. In contrast, non-Hispanic black injectors are more likely than other PWID subpopulations to experience drug-related arrests and incarceration [108], [109]. Fear of arrest has been associated with higher rates of risk behaviors, with difficulty in using prevention programs, and injection-related infections [110], [111], [112], [113], [114].

Resource availability and the allocation of resources can vary greatly, affecting where and when prevention and treatment services are implemented. Access to a broad range of comprehensive health services, medical care and drug treatment services, such as those offered at MAT facilities, are fundamental to achieving and reducing harm among PWID [115], [116]. Effectiveness of MATs may have played a role in the overall decreases in PWID rates we observed and decreasing HIV transmission rates among PWID. Provisions of MAT for PWID and PWID subpopulations remain central to efforts for the prevention and management of HIV infections and health care among PWID; however, access and coverage of MAT might not be sufficient in some U.S. localities, further impacting PWID rates and HIV and HCV transmission, particularly among young PWID and non-Hispanic black PWID.

One of the most practical applications of our PWID estimates in terms of targeting primary and secondary HIV prevention initiatives would be the computation of population-based HIV and HVC transmission rates among PWID and subpopulations. Furthermore, trend data on PWID prevalence and subpopulations may provide a foundation for the design, implementation and evaluation of structural interventions and service coverage, such as the expansion of OTPs and MAT facilities in areas of need [31], [32], [117]. Efforts to characterize PWID populations and PWID subpopulations have been complicated by prejudices about the stigmatizing nature of injection drug use [118], [119]. Prejudice about drug use, and in particular injection drug use, makes it difficult to assess the actual numbers of PWID over time and across geographic areas [36], [37], [38], [39], [40], [41], [42]. Our estimates on overall PWID population prevalence and PWID subpopulation prevalence have attempted to correct for this inability to measure and estimate PWID populations.

### Data Source Biases

Data sources were chosen so that biases of the different data sets, in theory, would cancel one another out [43], [55]. For example, the CTS data and the TEDS data both reflect health service provision: if funding for health service declines, so will the number of PWID noted for that data set. In contrast, a high component estimate based on PWID being diagnosed with AIDS, in conjunction with low service-based component estimates, may reflect inadequate services for PWID, such as having poor access to HAART. It is important that our various component estimates are not subject to the same biases, so that when they are averaged, even if one component estimate declines because of funding cuts, another component estimate will be unaffected by this event or will be affected in the opposite way.

### Limitations

Despite the fact that using different data sources helps to balance biases, each data series has strengths and weaknesses and are subject to several limitations. These limitations have been discussed in detail elsewhere [43], [44], [45], [46], [55], and are briefly reviewed here.

First, with regard to TEDS and UFDS/N-SSATS, during our study period SAMSHA eliminated questions from UFDS about the number of PWID in treatment programs after 1998. We therefore multiplied the proportion of drug users entering treatment who inject drugs (from TEDS) in each MSA and year by the total number of drug users in treatment as reported by both UFDS/N-SSATS. Second, these data sets differ in what they count: TEDS counts each admission in a given year, so an individual who enters drug treatment twice or more in a year is counted as two or more independent cases. In contrast, UFDS/N-SSATS client count numbers are from a point-prevalence survey–those in treatment on one specific day. It gives a snapshot of the substance abuse treatment system on a given day. Consequently, if PWID differ from non-PWID in the ratio of admissions to those remaining in treatment, our estimates will be biased.

Additionally, UFDS/N-SSATS data sets were not available for 1992, 1994, 1999, and 2001. We did not impute UFDS/N-SSATS for the years 1992, 1994, 1999, and 2001 because we did not believe there was a need to estimate the variance of the individual series, as the treatment component estimate would be smoothed and averaged with the other component estimates. As such, our final PWID estimates are calculated without using UFDS/N-SSATS data in the above years.

The counseling and testing data series has several limitations. First, data only exist where there are publicly funded CDC HIV counseling and testing sites. Since CTS systems only collect data from subsets of United States locations where publicly funded CDC HIV counseling and testing take place our estimates may be biased to the extent that the demographic distributions of PWIDs using these sites differ from those at sites that were not included. For example, the HIV CTS data as a whole are considered to only represent approximately 10–15% of all HIV tests in the U.S. [48], [120]. Therefore, HIV testing of PWID occurring at a health maintenance organization or at a blood donation center is not included in these data and may lead to an underestimation of the number of PWID. Second, reluctance to identify and report illicit and otherwise stigmatized behaviors may lead to misclassification and underreporting of PWID in the counseling and testing data.

Additionally, the counseling and testing data classify injectors as anyone injecting since 1978, although definitions used to classify injectors may vary by health department e.g., some people were classified as PWID if they reported injecting in their lifetimes, while others were classified as an PWID only if they reported injecting in the past 30 days, or since the last HIV test [120], [121]. This means we may not be capturing recent injectors in this PWID prevalence measure and that comparability across health department jurisdictions may be limited.

Included in our PWID estimate model was an adjustment for time to the advent of highly active antiretroviral therapies HAART in 1996. Since then, HAART has become the standard of care for the treatment of HIV-infected individuals. As HAART has become more widely used, AIDS researchers have seen an increase in the time from HIV infection to progression of AIDS, and an increase in survival time from a diagnosis of AIDS to death [122], [123], [124], [125]. Thus, as survival time increases prevalence rates in the research estimates we needed to adjust for this in our equation. During our study period, access to HAART changed over time and took time to reach PWID–thus, we assume year of 1996 as a uniform time for which PWID had access to HAART. To adjust for this in our AIDS component estimation series, we assume that PWID in all MSAs have equal access to HAART, starting 1996.

The component series based on the interpolation and extrapolation of Holmberg and Friedman’s estimates assumes a linear change on the log scale for each MSA. The weakness in this assumption is that the changes in number of PWID may not have changed linearly (on the log scale) over time in each MSA. Additionally, this component series is further subject to all the limitations, biases, and weaknesses that Holmberg and Friedman highlighted in their papers.

Finally, our PWID estimates do not extend beyond 2007. Completion of our estimates is based on the availability and release of secondary data; as such data for producing PWID estimates beyond 2007 were not readily available. In this paper, we presented estimates of the prevalence of people who injected in the last year. As such, these estimates do not provide information about the frequency of injecting or about risk behaviors among injectors.

### Conclusions

With proper acknowledgement of their limitations, these data can be used for structural analyses of the correlates and predictors of the population density of PWID and subpopulations in metropolitan areas. Specifically, the correlations found between the PWID estimates and theoretically relevant variables suggest that further research should be done to determine whether socioeconomic and health policy affect the population density of PWID.

These PWID prevalence and subpopulation prevalence estimates can help local agencies plan local drug-related interventions and policies. For example, where both our broader estimates and local data suggest that PWID prevalence is steady or rising, local health departments working together with syringe exchange programs might intensify outreach efforts targeting this population. Our estimates can also be used to measure the extent to which health systems and social service systems are providing services to PWID and PWID subpopulations.

For example, before now no adequate data have existed on patterns of female PWID prevalence over time within geographic areas. The lack of such prevalence data makes it difficult to assess whether at a metropolitan area has gender disparities in access to essential services [126], [127]. Thus the sex-specific PWID prevalence estimates reported here can inform planning efforts for health services for female PWIDs, and help design comprehensive health programs for female PWID. In addition, in the U.S., overdose is the most frequent cause of mortality among PWID [128], [129], [130] and the second leading cause of accidental injury death overall [130]. Our PWID prevalence estimates and subpopulation estimates can deepen our understanding of the causes of determinants and changes over time in overdoses deaths and HCV mortality among PWID populations. Finally, with more young people injecting, drug-related morbidity, including overdose, HIV and HCV infection, is likely to increase in this age group. Funding for harm reduction and other health-related services targeting young PWID should be increased.

In summary, it is clearly of very high importance to conduct research to understand what it is about some, but not other, MSAs that leads to increasing PWID prevalence, and to monitor future changes in injection by youth and, in general, changes in PWID prevalence and subpopulation prevalence rates. Our estimates can be used to inform public health officials about population vulnerability to future blood-borne epidemics, and will help local policy makers differentiate why PWID prevalence and subpopulation prevalence varies over time. Additionally, our estimates may be used in research into how socioeconomic conditions, public health programs, policies, and other socio-political factors shape the prevalence of PWID in a population.

## Supporting Information

Appendix S1 **A1. AIDS cases as adjusted for HIV prevalence** Figure A1: Estimation of Annual U.S. HIV Prevalence Rates among People Who Inject Drugs, Observed and Predicted Values A2. Adjustment of AIDS diagnoses for the proportion of PWID testing positive for HIV.
(DOC)

Appendix S2 **The Estimates of the Number of PWID per 10,000 population aged 15–64 years for each of the 96 largest MSAs in the US for each year from 1992 to 2007.**
(PDF)
