# Supplementary material for: Trends in the Population Prevalence of People Who Inject Drugs in US Metropolitan Areas 1992–2007
Source: PLoS One. 2013 Jun 5;8(6):e64789. doi: 10.1371/journal.pone.0064789 (PMC3673953; doi:10.1371/journal.pone.0064789)
Supplement: Appendix S1 — A1. AIDS cases as adjusted for HIV prevalence Figure A1: Estimation of Annual U.S. HIV Prevalence Rates among People Who Inject Drugs, Observed and Predicted Values A2. Adjustment of AIDS diagnoses for the proportion of PWID testing positive for HIV. (DOC) [file pone.0064789.s001.doc]

**Appendix A**

**A1.** **AIDS cases as adjusted for HIV prevalence**

We create time-varying estimates of HIV prevalence rates in the U.S. and in each MSA by calculating values of I*_it_* and adjusting for HIV seroprevalence as described below. Time-varying HIV prevalence rates among PWID, for the U.S. and MSAs, and year were estimated using previously published data and then lagged 5 years.

I*_it_*= IUS*_t_* * (A*_it5_* / H*_it5_*) / (AUS*_t5_*/ HUS*_t5_*)

where

I*_it_* – Estimated number of PWID in the MSA i in year t;

IUS*_t_* – Estimated number of PWID in the U.S. in year t;

A*_it_* – Number of AIDS cases diagnosed in PWID in the MSA i in year t;

AUS*_t_* – Number of AIDS cases diagnosed in PWID in the U.S. in year t;

H*_it5_* – Estimated number of HIV cases among PWID in the MSA i in year t -5;

HUS*_t5_* – Estimated number of HIV cases among PWID in the U.S. in year t -5

*HUS_t5_* – *Estimated number of HIV cases among PWID in the US in year t -5.* Given that no standard national HIV prevalence estimates exist for PWID over time [1, 2, 3, 4], we developed methods to estimate U.S. HIV prevalence rates for PWID for 1992-2008. Our annual U.S. HIV prevalence estimates for PWID are derived from a review of published literature and conference abstracts, as well as web-based searches and inquiries of researchers [5, 6, 7, 8]. To be eligible, HIV prevalence rates from studies had to have been conducted during 1992–2010 and only included those where HIV transmission risk category was injection drug use. We derive two sets of national HIV prevalence estimates and average these for a final annual U.S. HIV estimate from: (1) previously-published HIV prevalence rates for PWID in 96 MSAs (1992-2002) by Tempalski and colleagues [5]; and (2) prevalence estimates taken from Karon et al (1996) [6] and as reported by Morbidity and Mortality Weekly Report [7, 8]. For each set of estimates, we use generalized linear regression modeling (GLM) to predict HIV prevalence estimates in years where data were missing. The model corrects for missing data and includes linear and quadratic effects of time, measured as years since 1992.

We first extend previously published estimates of HIV prevalence rates [5] for years 1992-2002 to include years 2004-2008. The annual point estimates were obtained by multiplying the HIV prevalence rate by the total number of PWID for each MSA and year for 1992-2002. We then calculated an annual total by summing each value by year and dividing by the annual number of U.S. PWID. Using results from the GLM, we use years 1992-2002 to predict years 2003-2008, the resulting predicted values for 2003-2008 replace missing values for these years.

Our second set of national HIV prevalence estimates are derived from national point estimates from previously published data sources [6, 7, 8]. The three different data sources and methods used in estimating PWID infected with HIV in the U.S. in years 1992 (16%); 2006 (16.3%) and 2008, (17.2%) are consistent across studies. Using the same methodological approach as above, we utilize GLM to predict where specific years of national HIV prevalence rates were missing.

Figure S1 depicts both sets of national HIV prevalence estimates for PWID: (1) published prevalence estimates; (2) predicted values over time; and (3) the final averaged estimate. The mean U.S. HIV prevalence rate over time was 12.4; standard deviation 1.45; median 12.0; percentile range 11-13. Over all, the mean national HIV prevalence rate among PWID declined from 15.0% in 1992 to 10.7% in 2008. We further time lag these national estimates to correspond to our equation in *year t -5.*

**Figure S1: Estimation of Annual U.S. HIV Prevalence Rates among People Who Inject Drugs, Observed and Predicted Values**

*H_it5_ -* *Estimated number of HIV cases among PWID in the MSA i in year t -5.* For MSA-level HIV prevalence data, we utilize published HIV prevalence rates among PWID as estimated by Tempalski and colleagues (2009) [5]. Tempalski and colleagues created longitudinal PWID-HIV prevalence estimates in 96 MSAs during 1992–2002. Using data derived from a number of independent data sources, Tempalski et al (2009) [5] developed two sets of estimates based on independent methodologies: (1) calculating CTS-based Method (CBM) using regression adjustments to CDC CTS; and (2) calculating the PLWA-based Method (PBM) by taking the ratio of the number of injectors living with HIV to the numbers of injectors living in the MSA. Lastly, the mean of CBM and PBM was used to calculate over all HIV prevalence rates for 1992–2002.[[1]](#footnote-2)

Finally, for the purpose of our AIDS cases adjustment formula, we employ these longitudinal PWID-HIV prevalence estimates lagged at *year t -5.*

**A2. Adjustment of AIDS diagnoses for the proportion of PWID testing positive for HIV**

To reduce potential bias due to variation among MSAs in relative HIV prevalence, we adjusted the proportion of PWID who were in each subpopulation from the AIDS data (P*ijAIDS_adj*) for HIV prevalence among PWID calculated from CTS data using the following formula:

P*ijAIDS_adj* = (Subpop*_ijAIDS_* / Subpop*_ij_*) / (T*_ijAIDS_* / T*_ij_*)

where

Subpop*_ijAIDS_* = Number of PWID AIDS cases in each subpopulation in study year i, MSA j

Subpop*_ij_* = Proportion of PWID in each subpopulation testing positive for HIV in CTS data in study year i, MSA j

T*_ijAIDS_* = Number of total AIDS cases of PWID in study year i, MSA j

T*_ij_* = Proportion of PWID testing positive for HIV in CTS data in study year i, MSA j

This formula assumes that the yearly HIV proportions of PWID subpopulations and of total PWID who test positive for HIV in CTS data in each MSA reflect HIV prevalence in their respective underlying populations. It is important to note that CTS data represent the number of tests and positive results, not the number of individuals testing positive.

**Reference:**

- 1. Hall I. Personal Communication 2011.
  2. Lansky A. Personal Communication 2011.
  3. Mathers B. Personal Communication 2011.
  4. Holtgrave D. Personal Communication 2011
  5. Tempalski B, Lieb S, Cleland CM, Cooper H, Brady JE, et al. (2009) HIV prevalence rates among injection drug users in 96 large US metropolitan areas, 1992-2002. J Urban Health 86:132-154.
  6. Karon JM, Rosenberg PS, McQuillan G, Khare M, Gwinn M, et al. (1996) Prevalence of HIV infection in the United States, 1984 to 1992. JAMA 276:126-131
  7. U.S. Department of Health and Human Services, Division of HIV/AIDS Prevention, National Center for HIV/AIDS, Viral Hepatitis, STD, and TB Prevention, Centers for Disease Control and Prevention (2011) HIV Surveillance — United States, 1981-2008. MMWR Morb Mortal Wkly Rep. 60:689-693.
  8. U.S. Department of Health and Human Services, Division of HIV/AIDS Prevention, National Center for HIV/AIDS, Viral Hepatitis, STD, and TB Prevention, Centers for Disease Control and Prevention (2008) HIV Prevalence Estimates — United States, 2006. MMWR Morb Mortal Wkly Rep. 57:1073-1075.

1. For a complete overview see Tempalski et al., (2009) HIV prevalence rates among injection drug users in 96 large US metropolitan areas, (1992-2002). *Journal of Urban Health* 86:132-54. [↑](#footnote-ref-2)
