# Supplementary material for: Trends in the Population Prevalence of People Who Inject Drugs in US Metropolitan Areas 1992–2007
Source: PLoS One. 2013 Jun 5;8(6):e64789. doi: 10.1371/journal.pone.0064789 (PMC3673953; doi:10.1371/journal.pone.0064789)
Supplement: Appendix S2 — The Estimates of the Number of PWID per 10,000 population aged 15–64 years for each of the 96 largest MSAs in the US for each year from 1992 to 2007. (PDF) [file pone.0064789.s002.pdf]

Metropolitan Statistical Area	PWID Population	Year	Number	Min	Max	Rank	Rate	Min	Max	Rank
Akron, OH	Total	1992	2,755	1,862	3,589	92	62.20	42.03	81.01	89
		1993	2,386	1,457	3,548	93	53.68	32.79	79.84	92
		1994	2,639	1,771	3,511	93	59.14	39.68	78.67	89
		1995	2,316	1,497	3,481	94	51.58	33.34	77.50	92
		1996	2,292	1,524	3,462	93	50.61	33.64	76.44	91
		1997	2,256	1,544	3,423	93	49.67	34.00	75.38	91
		1998	2,221	1,564	3,392	93	48.78	34.35	74.51	93
		1999	2,389	1,566	3,364	93	52.31	34.28	73.64	93
		2000	2,183	1,571	3,397	94	47.53	34.21	73.96	95
		2001	2,371	1,578	3,433	94	51.30	34.14	74.27	93
		2002	2,202	1,592	3,488	95	47.39	34.27	75.08	95
		2003	2,213	1,606	3,540	96	47.44	34.41	75.88	95
		2004	2,228	1,618	3,596	96	47.55	34.55	76.76	95
		2005	2,244	1,632	3,655	98	47.68	34.68	77.65	95
		2006	2,260	1,645	3,717	98	47.88	34.84	78.74	95
		2007	2,277	1,657	3,778	99	48.10	35.01	79.82	95
	Non-Hispanic White	1992	1,793	1,212	2,336	87	45.81	30.96	59.67	81
		1993	1,463	894	2,176	92	37.35	22.81	55.55	85
		1994	1,550	1,040	2,062	93	39.49	26.50	52.53	85
		1995	1,325	857	1,991	94	33.64	21.75	50.54	88
		1996	1,298	863	1,961	95	32.77	21.78	49.49	88
		1997	1,284	879	1,948	94	32.39	22.17	49.16	88
		1998	1,284	904	1,962	94	32.40	22.81	49.48	88
		1999	1,416	928	1,994	93	35.71	23.40	50.27	91
		2000	1,334	960	2,075	94	33.53	24.13	52.18	89
		2001	1,497	996	2,167	93	37.49	24.95	54.29	91
		2002	1,437	1,039	2,276	95	35.89	25.96	56.85	91
		2003	1,489	1,080	2,382	95	37.14	26.94	59.41	89
		2004	1,541	1,119	2,487	95	38.37	27.87	61.94	89
		2005	1,588	1,155	2,586	96	39.47	28.71	64.28	89
		2006	1,626	1,183	2,674	96	40.43	29.42	66.48	87
		2007	1,655	1,204	2,746	93	41.14	29.94	68.27	88

Metropolitan Statistical Area	PWID Population	Year	Number	Min	Max	Rank	Rate	Min	Max	Rank
Akron, OH	Non-Hispanic Black	1992	859	580	1,118	82	200.48	135.47	261.11	76
		1993	814	497	1,211	84	186.59	113.96	277.50	73
		1994	951	638	1,264	78	214.04	143.60	284.70	67
		1995	854	552	1,283	75	188.13	121.62	282.67	66
		1996	844	561	1,274	74	181.75	120.83	274.51	65
		1997	811	555	1,231	73	171.80	117.59	260.73	64
		1998	765	539	1,169	75	159.88	112.59	244.23	67
		1999	776	509	1,093	74	159.48	104.51	224.53	69
		2000	660	475	1,027	77	132.88	95.64	206.76	72
		2001	660	439	955	77	130.64	86.94	189.16	71
		2002	560	405	887	82	109.12	78.91	172.86	73
		2003	514	373	822	84	98.58	71.51	157.68	75
		2004	474	344	765	85	89.55	65.06	144.57	80
		2005	442	321	719	87	82.03	59.66	133.58	81
		2006	418	304	688	88	76.31	55.53	125.47	83
		2007	406	295	673	88	72.78	52.96	120.76	85
	Hispanic	1992	17	12	22	97	62.42	42.18	81.30	76
		1993	18	11	27	95	64.13	39.17	95.38	69
		1994	24	16	31	93	79.54	53.36	105.80	63
		1995	22	15	34	94	72.46	46.84	108.87	60
		1996	23	15	35	94	70.49	46.86	106.47	61
		1997	22	15	34	95	66.13	45.26	100.36	63
		1998	21	15	32	96	59.91	42.19	91.51	66
		1999	21	14	29	97	57.02	37.37	80.28	67
		2000	17	12	27	98	44.78	32.23	69.68	71
		2001	17	11	24	97	41.28	27.47	59.77	75
		2002	14	10	22	98	32.73	23.67	51.85	80
		2003	13	9	20	99	28.45	20.64	45.50	82
		2004	12	8	19	100	25.34	18.41	40.90	87
		2005	11	8	18	100	22.74	16.54	37.02	88
		2006	11	8	18	100	21.50	15.64	35.35	91
		2007	11	8	18	101	21.34	15.53	35.42	92

Metropolitan Statistical Area	PWID Population	Year	Number	Min	Max	Rank	Rate	Min	Max	Rank
Akron, OH	Male	1992	1,746	1,180	2,274	94	80.76	54.57	105.19	91
		1993	1,520	928	2,260	94	69.97	42.74	104.06	92
		1994	1,680	1,127	2,234	93	76.99	51.65	102.41	89
		1995	1,465	947	2,201	94	66.69	43.11	100.20	92
		1996	1,435	954	2,168	94	64.76	43.05	97.82	92
		1997	1,394	954	2,116	94	62.75	42.95	95.23	92
		1998	1,353	953	2,067	94	60.73	42.77	92.77	92
		1999	1,434	940	2,019	95	64.14	42.03	90.29	93
		2000	1,292	930	2,011	96	57.47	41.36	89.42	95
		2001	1,388	923	2,009	96	61.29	40.79	88.74	96
		2002	1,279	925	2,026	98	56.18	40.63	88.99	95
		2003	1,283	931	2,053	99	56.07	40.68	89.69	95
		2004	1,298	943	2,096	99	56.49	41.04	91.20	95
		2005	1,326	964	2,159	99	57.36	41.72	93.41	95
		2006	1,366	994	2,246	99	58.87	42.84	96.81	96
		2007	1,421	1,034	2,357	99	61.07	44.45	101.35	95
	Female	1992	1,039	702	1,353	86	45.79	30.94	59.64	82
		1993	890	544	1,324	91	39.17	23.92	58.25	84
		1994	988	663	1,314	91	43.31	29.05	57.61	85
		1995	880	569	1,322	92	38.34	24.78	57.60	87
		1996	890	591	1,344	93	38.46	25.57	58.09	87
		1997	899	615	1,365	93	38.77	26.54	58.84	87
		1998	911	641	1,391	93	39.18	27.59	59.84	88
		1999	1,007	660	1,418	91	43.20	28.31	60.81	88
		2000	942	678	1,466	94	40.21	28.94	62.56	87
		2001	1,042	694	1,509	92	44.20	29.42	64.00	87
		2002	978	707	1,549	95	41.27	29.85	65.38	85
		2003	982	713	1,571	95	41.32	29.98	66.10	85
		2004	975	709	1,575	96	40.87	29.69	65.98	85
		2005	954	694	1,554	96	39.83	28.97	64.87	87
		2006	915	666	1,504	96	38.11	27.73	62.66	89
		2007	856	623	1,420	97	35.56	25.88	59.00	92

Metropolitan Statistical Area	PWID Population	Year	Number	Min	Max	Rank	Rate	Min	Max	Rank
Akron, OH	Young (15-29)	1992	561	379	730	96	37.50	25.34	48.84	94
		1993	423	258	628	98	28.84	17.62	42.89	98
		1994	430	288	572	98	29.78	19.98	39.62	97
		1995	365	236	549	99	25.49	16.48	38.30	98
		1996	366	243	553	98	25.54	16.98	38.57	98
		1997	380	260	576	98	26.71	18.28	40.53	99
		1998	407	286	621	99	28.85	20.32	44.08	99
		1999	487	319	686	98	34.85	22.84	49.07	96
		2000	503	362	783	98	36.25	26.09	56.41	97
		2001	622	414	901	97	44.89	29.88	65.01	92
		2002	658	476	1,042	98	47.38	34.27	75.06	88
		2003	747	542	1,195	96	53.32	38.68	85.29	86
		2004	839	610	1,355	96	59.36	43.13	95.84	82
		2005	929	675	1,512	96	64.90	47.20	105.69	80
		2006	1,007	733	1,656	94	70.01	50.95	115.12	76
		2007	1,069	778	1,774	93	73.69	53.63	122.28	71
	Old (30-64)	1992	2,249	1,519	2,929	89	76.62	51.77	99.79	86
		1993	1,993	1,217	2,965	91	66.92	40.87	99.52	87
		1994	2,235	1,500	2,973	92	74.02	49.66	98.46	85
		1995	1,973	1,275	2,964	89	64.50	41.70	96.91	85
		1996	1,951	1,297	2,946	89	63.02	41.90	95.19	86
		1997	1,906	1,305	2,893	89	61.12	41.83	92.75	86
		1998	1,852	1,304	2,829	89	58.93	41.50	90.01	86
		1999	1,954	1,280	2,750	91	61.62	40.38	86.75	86
		2000	1,736	1,249	2,701	91	54.16	38.98	84.27	88
		2001	1,818	1,210	2,632	95	56.15	37.37	81.31	87
		2002	1,611	1,165	2,551	94	49.43	35.75	78.30	89
		2003	1,527	1,108	2,443	95	46.78	33.93	74.83	92
		2004	1,432	1,040	2,312	95	43.79	31.81	70.69	93
		2005	1,327	965	2,161	97	40.51	29.46	65.97	94
		2006	1,214	883	1,996	98	36.98	26.91	60.80	94
		2007	1,098	799	1,822	98	33.45	24.34	55.50	94

Metropolitan Statistical Area	PWID Population	Year	Number	Min	Max	Rank	Rate	Min	Max	Rank
Albany--Schenectady--Troy, NY	Total	1992	3,829	2,594	4,474	84	66.55	45.08	77.75	83
		1993	3,376	2,355	4,392	85	58.66	40.92	76.32	83
		1994	3,419	2,139	4,331	86	59.38	37.15	75.22	86
		1995	3,174	1,919	4,259	86	55.23	33.39	74.11	85
		1996	3,092	1,734	4,210	88	53.98	30.28	73.50	86
		1997	3,021	1,554	4,169	88	52.83	27.17	72.89	86
		1998	3,089	1,641	4,297	88	53.86	28.60	74.91	84
		1999	2,891	1,727	4,425	89	50.25	30.03	76.92	94
		2000	3,430	1,976	4,733	86	59.28	34.15	81.80	77
		2001	3,157	2,174	5,061	89	54.06	37.24	86.68	91
		2002	4,131	2,026	6,493	85	69.99	34.33	109.99	67
		2003	4,461	1,893	7,197	83	74.57	31.65	120.31	65
		2004	4,643	1,765	7,387	83	76.76	29.18	122.13	64
		2005	4,619	1,643	6,742	83	75.63	26.90	110.40	64
		2006	4,570	1,529	6,984	83	74.05	24.77	113.15	66
		2007	4,476	1,406	7,343	83	72.15	22.66	118.36	68
	Non-Hispanic White	1992	1,805	1,223	2,109	86	34.31	23.24	40.09	94
		1993	1,671	1,165	2,173	87	31.86	22.23	41.45	89
		1994	1,735	1,085	2,197	86	33.19	20.76	42.04	95
		1995	1,621	980	2,175	87	31.19	18.86	41.86	91
		1996	1,568	879	2,135	88	30.39	17.05	41.38	92
		1997	1,505	774	2,076	89	29.33	15.08	40.46	93
		1998	1,500	796	2,086	89	29.25	15.53	40.68	94
		1999	1,361	813	2,084	94	26.57	15.87	40.66	97
		2000	1,565	901	2,159	92	30.46	17.55	42.04	95
		2001	1,398	963	2,241	95	27.07	18.65	43.41	96
		2002	1,787	876	2,808	91	34.41	16.88	54.07	93
		2003	1,904	808	3,072	88	36.34	15.42	58.63	92
		2004	1,984	754	3,157	86	37.60	14.29	59.82	92
		2005	2,011	715	2,936	85	37.87	13.47	55.29	92
		2006	2,068	692	3,160	85	38.71	12.95	59.16	92
		2007	2,147	674	3,522	86	40.12	12.60	65.81	89

Metropolitan Statistical Area	PWID Population	Year	Number	Min	Max	Rank	Rate	Min	Max	Rank
Albany--Schenectady--Troy, NY	Non-Hispanic Black	1992	1,278	866	1,494	65	458.96	310.93	536.25	21
		1993	1,078	752	1,403	71	373.59	260.58	486.01	31
		1994	1,048	656	1,328	69	351.16	219.71	444.78	31
		1995	937	566	1,257	71	304.80	184.26	409.01	34
		1996	880	494	1,199	71	278.65	156.33	379.46	37
		1997	831	427	1,147	71	256.01	131.68	353.22	37
		1998	821	436	1,143	72	246.05	130.66	342.21	36
		1999	743	444	1,137	75	216.81	129.55	331.86	44
		2000	852	491	1,175	67	242.47	139.70	334.58	28
		2001	756	521	1,212	72	208.52	143.62	334.30	37
		2002	952	467	1,497	65	253.31	124.25	398.11	19
		2003	987	419	1,592	64	253.95	107.79	409.74	18
		2004	983	373	1,563	65	246.39	93.65	391.99	18
		2005	932	331	1,360	66	228.75	81.36	333.95	21
		2006	876	293	1,338	68	208.57	69.78	318.73	28
		2007	813	255	1,334	74	189.63	59.56	311.08	38
	Hispanic	1992	586	397	685	48	533.74	361.59	623.62	6
		1993	491	342	638	49	427.52	298.20	556.17	8
		1994	488	305	618	53	406.95	254.61	515.45	8
		1995	457	276	613	54	365.11	220.72	489.95	9
		1996	459	258	626	54	353.49	198.32	481.37	9
		1997	471	242	650	56	347.53	178.76	479.49	11
		1998	511	272	711	55	362.79	192.66	504.58	9
		1999	511	305	782	55	349.09	208.59	534.34	9
		2000	648	373	894	53	420.93	242.52	580.85	6
		2001	635	437	1,018	55	394.99	272.05	633.26	8
		2002	878	431	1,379	49	517.88	254.02	813.91	5
		2003	989	420	1,596	48	553.77	235.04	893.48	5
		2004	1,058	402	1,683	49	564.36	214.51	897.86	3
		2005	1,060	377	1,547	51	545.13	193.90	795.82	3
		2006	1,032	345	1,576	51	503.18	168.35	768.93	2
		2007	967	304	1,586	52	457.81	143.80	751.02	3

Metropolitan Statistical Area	PWID Population	Year	Number	Min	Max	Rank	Rate	Min	Max	Rank
Albany--Schenectady--Troy, NY	Male	1992	2,638	1,787	3,083	83	92.79	62.86	108.42	83
		1993	2,351	1,640	3,058	83	82.65	57.65	107.52	84
		1994	2,398	1,501	3,038	85	84.27	52.72	106.73	86
		1995	2,236	1,352	3,001	85	78.73	47.60	105.65	83
		1996	2,184	1,225	2,974	85	77.14	43.28	105.05	83
		1997	2,135	1,098	2,946	86	75.53	38.85	104.20	83
		1998	2,180	1,158	3,033	86	76.86	40.82	106.90	81
		1999	2,035	1,216	3,115	86	71.55	42.75	109.52	87
		2000	2,406	1,386	3,320	85	84.10	48.46	116.05	69
		2001	2,204	1,518	3,534	86	76.30	52.55	122.32	81
		2002	2,870	1,408	4,510	84	98.20	48.17	154.33	63
		2003	3,082	1,308	4,973	78	103.95	44.12	167.72	58
		2004	3,192	1,213	5,078	78	106.41	40.45	169.29	58
		2005	3,159	1,124	4,612	80	104.30	37.10	152.27	57
		2006	3,113	1,042	4,758	78	101.67	34.01	155.36	58
		2007	3,040	955	4,987	79	98.70	31.00	161.92	61
	Female	1992	1,164	788	1,360	85	39.98	27.09	46.72	86
		1993	1,025	715	1,333	86	35.22	24.56	45.82	87
		1994	1,040	650	1,317	89	35.70	22.34	45.22	91
		1995	968	585	1,299	88	33.32	20.14	44.71	91
		1996	947	532	1,290	89	32.71	18.35	44.54	91
		1997	931	479	1,285	92	32.20	16.56	44.43	92
		1998	958	509	1,333	91	33.05	17.55	45.97	92
		1999	903	539	1,382	95	31.04	18.55	47.51	96
		2000	1,078	621	1,488	89	36.84	21.23	50.84	91
		2001	998	687	1,600	96	33.83	23.30	54.24	94
		2002	1,313	644	2,063	87	44.04	21.60	69.22	79
		2003	1,423	604	2,295	86	47.15	20.01	76.07	75
		2004	1,484	564	2,361	86	48.66	18.49	77.41	74
		2005	1,476	525	2,154	86	47.94	17.05	69.99	77
		2006	1,457	487	2,226	87	46.85	15.67	71.59	76
		2007	1,420	446	2,329	87	45.45	14.28	74.56	79

Metropolitan Statistical Area	PWID Population	Year	Number	Min	Max	Rank	Rate	Min	Max	Rank
Albany--Schenectady--Troy, NY	Young (15-29)	1992	816	553	953	87	41.73	28.27	48.75	88
		1993	778	543	1,012	86	40.91	28.53	53.22	84
		1994	842	527	1,067	85	45.34	28.37	57.43	82
		1995	827	500	1,110	84	45.50	27.51	61.06	80
		1996	845	474	1,151	86	47.51	26.66	64.71	76
		1997	859	442	1,185	87	49.07	25.24	67.71	75
		1998	906	481	1,260	87	52.25	27.75	72.67	74
		1999	870	520	1,331	89	50.63	30.26	77.51	80
		2000	1,052	606	1,452	87	61.53	35.45	84.91	71
		2001	983	677	1,575	89	56.68	39.04	90.87	80
		2002	1,299	637	2,042	86	73.66	36.13	115.76	67
		2003	1,412	599	2,279	83	77.72	32.99	125.40	64
		2004	1,475	561	2,347	83	79.18	30.09	125.97	64
		2005	1,469	523	2,145	84	76.90	27.35	112.26	69
		2006	1,453	486	2,221	84	74.17	24.82	113.34	69
		2007	1,422	447	2,332	84	71.66	22.51	117.55	73
	Old (30-64)	1992	3,059	2,073	3,575	83	80.54	54.56	94.10	81
		1993	2,636	1,839	3,430	84	68.42	47.72	89.01	86
		1994	2,618	1,638	3,316	86	67.12	41.99	85.02	89
		1995	2,390	1,445	3,208	86	60.85	36.79	81.66	88
		1996	2,298	1,289	3,129	87	58.18	32.64	79.23	89
		1997	2,222	1,143	3,066	87	55.98	28.79	77.23	91
		1998	2,252	1,196	3,132	87	56.27	29.88	78.27	89
		1999	2,091	1,249	3,200	87	51.82	30.96	79.32	92
		2000	2,463	1,419	3,398	87	60.41	34.80	83.36	78
		2001	2,248	1,549	3,605	87	54.76	37.72	87.80	89
		2002	2,915	1,430	4,581	81	70.42	34.54	110.68	64
		2003	3,111	1,320	5,019	79	74.69	31.70	120.50	60
		2004	3,190	1,213	5,075	79	76.22	28.97	121.26	55
		2005	3,111	1,107	4,542	79	74.15	26.37	108.25	57
		2006	2,999	1,003	4,583	79	71.20	23.82	108.80	59
		2007	2,836	891	4,652	80	67.20	21.11	110.25	60

Metropolitan Statistical Area	PWID Population	Year	Number	Min	Max	Rank	Rate	Min	Max	Rank
Albuquerque, NM	Total	1992	10,123	5,575	17,982	44	246.38	135.70	437.68	6
		1993	9,203	5,536	16,737	44	218.91	131.68	398.11	8
		1994	9,415	5,557	15,540	44	216.64	127.86	357.57	10
		1995	8,743	5,551	14,186	46	195.38	124.05	317.02	10
		1996	8,422	5,489	12,774	46	184.35	120.16	279.63	13
		1997	7,999	5,368	11,184	47	173.25	116.28	242.24	13
		1998	7,735	5,276	10,160	51	165.33	112.77	217.17	13
		1999	7,271	5,151	9,057	57	154.21	109.26	192.10	18
		2000	7,561	5,172	9,456	50	158.17	108.21	197.81	14
		2001	7,600	5,190	9,859	54	156.89	107.15	203.53	17
		2002	7,932	5,225	10,663	49	160.11	105.47	215.23	11
		2003	8,152	5,232	11,437	49	161.76	103.80	226.93	13
		2004	8,321	5,244	11,908	49	161.77	101.95	231.51	13
		2005	8,513	5,269	12,428	50	161.72	100.10	236.10	11
		2006	8,677	5,297	12,825	48	160.99	98.28	237.97	11
		2007	8,802	5,298	13,172	48	160.26	96.46	239.83	10
	Non-Hispanic White	1992	4,547	2,504	8,077	52	204.04	112.38	362.46	8
		1993	4,626	2,782	8,412	48	204.45	122.98	371.81	5
		1994	4,971	2,934	8,204	46	213.63	126.09	352.60	4
		1995	4,645	2,949	7,536	48	195.25	123.97	316.82	5
		1996	4,366	2,846	6,623	48	182.22	118.77	276.40	5
		1997	3,959	2,657	5,535	51	165.34	110.96	231.17	5
		1998	3,606	2,459	4,736	57	150.80	102.86	198.08	9
		1999	3,182	2,254	3,963	72	134.13	95.04	167.08	17
		2000	3,134	2,144	3,920	66	132.64	90.74	165.89	15
		2001	3,056	2,087	3,964	73	129.40	88.37	167.85	17
		2002	3,212	2,116	4,317	68	134.41	88.54	180.68	13
		2003	3,489	2,239	4,895	65	144.99	93.04	203.40	9
		2004	3,964	2,498	5,674	56	162.54	102.43	232.61	8
		2005	4,718	2,920	6,888	44	190.82	118.10	278.58	7
		2006	5,727	3,496	8,465	39	228.27	139.34	337.40	5
		2007	6,874	4,137	10,287	29	271.93	163.67	406.96	3

Metropolitan Statistical Area	PWID Population	Year	Number	Min	Max	Rank	Rate	Min	Max	Rank
Albuquerque, NM	Non-Hispanic Black	1992	337	186	599	97	347.79	191.55	617.82	41
		1993	283	170	514	97	280.72	168.85	510.51	51
		1994	267	158	441	97	257.09	151.74	424.33	56
		1995	230	146	373	97	213.19	135.35	345.91	53
		1996	207	135	314	97	185.22	120.73	280.95	63
		1997	185	124	259	97	161.66	108.49	226.03	69
		1998	171	117	225	98	147.37	100.52	193.58	72
		1999	155	110	194	98	131.52	93.18	163.83	77
		2000	159	109	199	98	134.10	91.74	167.71	71
		2001	160	109	208	97	131.92	90.09	171.13	69
		2002	171	113	230	97	135.37	89.18	181.97	66
		2003	185	118	259	96	141.59	90.86	198.64	57
		2004	203	128	290	96	149.05	93.93	213.31	54
		2005	230	142	336	95	161.11	99.72	235.21	45
		2006	269	164	397	95	178.35	108.87	263.62	42
		2007	324	195	485	93	205.43	123.65	307.44	33
	Hispanic	1992	5,454	3,004	9,688	13	356.08	196.11	632.54	14
		1993	5,088	3,060	9,253	15	322.33	193.88	586.18	13
		1994	5,216	3,078	8,609	13	318.02	187.70	524.89	12
		1995	4,764	3,025	7,731	15	279.55	177.49	453.59	14
		1996	4,451	2,901	6,751	15	252.18	164.37	382.50	15
		1997	4,058	2,723	5,673	16	225.06	151.04	314.66	17
		1998	3,744	2,554	4,918	18	202.41	138.06	265.87	19
		1999	3,357	2,378	4,181	19	177.55	125.80	221.17	20
		2000	3,347	2,290	4,187	22	171.76	117.50	214.81	19
		2001	3,267	2,231	4,238	20	163.18	111.44	211.67	21
		2002	3,376	2,224	4,538	20	163.42	107.66	219.68	19
		2003	3,527	2,263	4,947	20	166.51	106.85	233.59	19
		2004	3,775	2,379	5,402	18	173.74	109.49	248.64	18
		2005	4,189	2,593	6,116	17	186.98	115.73	272.99	18
		2006	4,777	2,916	7,061	15	206.88	126.28	305.79	17
		2007	5,539	3,334	8,289	13	233.03	140.26	348.74	16

Metropolitan Statistical Area	PWID Population	Year	Number	Min	Max	Rank	Rate	Min	Max	Rank
Albuquerque, NM	Male	1992	6,625	3,649	11,769	44	326.07	179.58	579.23	7
		1993	5,933	3,568	10,789	44	285.30	171.61	518.85	9
		1994	5,999	3,540	9,901	45	278.66	164.47	459.93	12
		1995	5,522	3,506	8,960	44	249.10	158.16	404.19	13
		1996	5,288	3,447	8,021	45	233.61	152.27	354.34	13
		1997	5,003	3,357	6,994	47	218.90	146.91	306.06	14
		1998	4,826	3,291	6,339	54	208.36	142.12	273.69	18
		1999	4,529	3,209	5,641	59	194.20	137.59	241.91	21
		2000	4,703	3,217	5,882	52	198.87	136.05	248.71	19
		2001	4,719	3,223	6,121	54	196.62	134.28	255.06	18
		2002	4,911	3,235	6,601	48	199.86	131.66	268.66	16
		2003	5,024	3,224	7,048	48	200.81	128.87	281.72	16
		2004	5,092	3,209	7,287	49	199.24	125.56	285.13	16
		2005	5,156	3,191	7,527	49	196.91	121.88	287.48	16
		2006	5,180	3,162	7,657	49	193.39	118.05	285.85	16
		2007	5,155	3,103	7,714	49	188.76	113.61	282.49	16
	Female	1992	3,510	1,933	6,236	42	169.03	93.09	300.27	7
		1993	3,381	2,034	6,149	40	159.14	95.73	289.42	7
		1994	3,613	2,133	5,964	42	164.73	97.23	271.89	5
		1995	3,463	2,199	5,619	40	153.38	97.38	248.87	7
		1996	3,409	2,222	5,170	40	147.90	96.40	224.34	8
		1997	3,281	2,202	4,587	43	140.72	94.44	196.75	11
		1998	3,193	2,178	4,194	43	135.16	92.19	177.54	12
		1999	3,004	2,129	3,742	52	126.09	89.34	157.06	11
		2000	3,113	2,130	3,894	45	128.90	88.19	161.22	11
		2001	3,110	2,124	4,034	49	127.23	86.89	165.05	12
		2002	3,219	2,121	4,327	45	128.92	84.92	173.29	9
		2003	3,279	2,105	4,601	45	129.21	82.91	181.26	9
		2004	3,320	2,092	4,751	45	128.29	80.85	183.60	9
		2005	3,376	2,090	4,929	45	127.61	78.98	186.31	9
		2006	3,432	2,095	5,072	43	126.60	77.28	187.12	8
		2007	3,488	2,099	5,220	41	126.31	76.02	189.02	11

Metropolitan Statistical Area	PWID Population	Year	Number	Min	Max	Rank	Rate	Min	Max	Rank
Albuquerque, NM	Young (15-29)	1992	3,462	1,906	6,149	37	254.75	140.30	452.54	3
		1993	3,130	1,883	5,692	34	229.17	137.85	416.77	5
		1994	3,217	1,898	5,309	36	230.86	136.26	381.04	3
		1995	3,023	1,919	4,904	34	212.62	135.00	345.00	2
		1996	2,960	1,929	4,489	33	204.77	133.47	310.60	2
		1997	2,862	1,921	4,001	34	195.74	131.37	273.68	4
		1998	2,814	1,919	3,696	36	189.46	129.23	248.86	5
		1999	2,679	1,898	3,338	45	179.34	127.07	223.40	11
		2000	2,804	1,918	3,507	41	185.90	127.18	232.50	8
		2001	2,811	1,919	3,646	45	183.59	125.38	238.15	12
		2002	2,891	1,904	3,886	44	183.51	120.89	246.68	14
		2003	2,884	1,851	4,045	45	179.15	114.96	251.32	16
		2004	2,804	1,767	4,013	47	170.01	107.14	243.30	17
		2005	2,673	1,654	3,902	55	157.49	97.48	229.93	19
		2006	2,469	1,507	3,649	62	141.99	86.68	209.88	26
		2007	2,196	1,322	3,286	65	124.01	74.64	185.58	33
	Old (30-64)	1992	6,620	3,646	11,761	51	240.77	132.61	427.71	14
		1993	6,152	3,700	11,188	47	216.74	130.37	394.15	15
		1994	6,381	3,766	10,532	51	216.11	127.55	356.69	16
		1995	5,971	3,791	9,688	51	195.56	124.16	317.31	16
		1996	5,767	3,759	8,748	52	184.68	120.37	280.12	20
		1997	5,471	3,672	7,650	54	173.42	116.39	242.47	19
		1998	5,268	3,593	6,920	57	164.99	112.54	216.72	20
		1999	4,921	3,486	6,129	63	152.78	108.25	190.32	24
		2000	5,078	3,474	6,352	58	155.22	106.19	194.13	18
		2001	5,067	3,460	6,573	58	152.93	104.44	198.39	23
		2002	5,255	3,462	7,064	54	155.52	102.45	209.05	16
		2003	5,381	3,453	7,549	49	156.86	100.66	220.06	13
		2004	5,492	3,461	7,860	48	157.20	99.07	224.97	12
		2005	5,647	3,495	8,244	47	158.32	97.99	231.14	9
		2006	5,818	3,551	8,599	44	159.35	97.27	235.54	9
		2007	5,999	3,611	8,978	44	161.21	97.03	241.26	9

Metropolitan Statistical Area	PWID Population	Year	Number	Min	Max	Rank	Rate	Min	Max	Rank
Allentown--Bethlehem--Easton, PA	Total	1992	6,095	5,683	6,713	68	154.97	144.51	170.68	30
		1993	5,622	3,145	6,750	70	142.32	79.62	170.87	33
		1994	6,792	6,375	7,207	63	171.03	160.54	181.48	20
		1995	6,066	2,935	7,901	68	152.29	73.68	198.34	26
		1996	6,175	2,829	8,140	66	154.12	70.62	203.19	23
		1997	6,225	2,723	8,148	65	154.51	67.58	202.23	20
		1998	6,252	2,618	8,016	65	154.12	64.55	197.59	18
		1999	7,508	7,038	7,775	56	183.49	172.02	190.01	10
		2000	6,287	2,406	8,039	65	152.47	58.34	194.96	18
		2001	7,763	7,193	8,406	52	186.05	172.40	201.47	8
		2002	6,527	2,208	8,704	64	154.25	52.19	205.70	15
		2003	6,661	2,115	9,041	59	154.66	49.12	209.93	15
		2004	6,753	2,030	9,397	57	153.84	46.25	214.08	15
		2005	6,662	1,947	9,768	61	148.84	43.50	218.22	16
		2006	6,538	1,857	10,141	65	143.45	40.74	222.52	19
		2007	6,350	1,759	10,473	70	137.51	38.10	226.81	21
	Non-Hispanic White	1992	3,321	3,097	3,657	67	92.14	85.92	101.48	40
		1993	3,047	1,705	3,658	68	84.60	47.33	101.57	41
		1994	3,655	3,431	3,878	62	101.49	95.27	107.70	36
		1995	3,239	1,567	4,219	65	90.19	43.64	117.47	41
		1996	3,271	1,499	4,312	65	91.16	41.77	120.18	43
		1997	3,273	1,432	4,284	65	91.27	39.92	119.46	40
		1998	3,267	1,368	4,189	64	91.06	38.14	116.74	38
		1999	3,907	3,663	4,046	57	108.70	101.90	112.56	28
		2000	3,268	1,250	4,178	63	90.77	34.73	116.07	42
		2001	4,044	3,747	4,379	56	111.73	103.53	120.99	27
		2002	3,422	1,158	4,563	63	93.84	31.75	125.14	36
		2003	3,531	1,121	4,793	61	95.91	30.46	130.18	36
		2004	3,639	1,094	5,064	63	97.81	29.41	136.11	35
		2005	3,668	1,072	5,378	64	97.57	28.51	143.05	35
		2006	3,697	1,050	5,736	64	97.70	27.75	151.55	38
		2007	3,707	1,027	6,114	64	97.70	27.07	161.15	38

Metropolitan Statistical Area	PWID Population	Year	Number	Min	Max	Rank	Rate	Min	Max	Rank
Allentown--Bethlehem--Easton, PA	Non-Hispanic Black	1992	776	724	855	84	948.59	884.54	1044.72	2
		1993	708	396	850	85	824.42	461.21	989.80	3
		1994	833	782	884	82	922.81	866.25	979.20	1
		1995	717	347	934	85	756.13	365.82	984.77	3
		1996	696	319	918	84	700.25	320.85	923.19	3
		1997	666	291	871	84	638.84	279.42	836.15	4
		1998	631	264	810	82	577.09	241.68	739.86	4
		1999	715	671	741	77	621.62	582.74	643.71	3
		2000	566	217	724	82	471.64	180.47	603.07	6
		2001	664	615	719	76	527.21	488.54	570.92	4
		2002	534	181	712	83	399.68	135.24	532.99	8
		2003	527	167	715	83	366.62	116.43	497.63	9
		2004	524	158	730	82	335.06	100.74	466.26	12
		2005	517	151	757	82	305.96	89.41	448.59	14
		2006	518	147	803	82	282.49	80.23	438.20	15
		2007	527	146	870	83	269.36	74.63	444.27	17
	Hispanic	1992	1,975	1,842	2,175	34	1034.67	964.80	1139.51	1
		1993	1,817	1,017	2,182	34	893.29	499.74	1072.49	1
		1994	2,198	2,063	2,332	32	1015.68	953.43	1077.74	1
		1995	1,972	954	2,568	32	854.52	413.42	1112.91	1
		1996	2,021	926	2,664	32	814.51	373.20	1073.82	1
		1997	2,053	898	2,687	32	775.23	339.08	1014.67	3
		1998	2,079	871	2,665	32	738.33	309.21	946.58	3
		1999	2,516	2,358	2,605	28	838.85	786.40	868.67	1
		2000	2,120	811	2,711	32	667.19	255.30	853.11	3
		2001	2,629	2,437	2,847	28	787.71	729.94	853.01	1
		2002	2,215	749	2,953	33	625.35	211.59	833.91	3
		2003	2,256	717	3,063	32	596.77	189.53	810.02	3
		2004	2,275	684	3,165	32	562.57	169.13	782.84	4
		2005	2,221	649	3,256	32	512.38	149.74	751.25	5
		2006	2,145	609	3,327	33	458.22	130.15	710.79	6
		2007	2,039	565	3,363	34	407.14	112.80	671.52	7

Metropolitan Statistical Area	PWID Population	Year	Number	Min	Max	Rank	Rate	Min	Max	Rank
Allentown--Bethlehem--Easton, PA	Male	1992	4,226	3,941	4,655	66	216.94	202.29	238.92	27
		1993	3,897	2,180	4,679	68	199.01	111.34	238.94	32
		1994	4,706	4,417	4,993	60	238.94	224.29	253.54	19
		1995	4,202	2,033	5,473	64	212.59	102.85	276.87	24
		1996	4,277	1,959	5,638	62	215.10	98.56	283.58	18
		1997	4,312	1,886	5,644	61	215.57	94.29	282.15	17
		1998	4,333	1,815	5,555	59	215.11	90.09	275.78	14
		1999	5,208	4,882	5,393	52	256.13	240.12	265.24	8
		2000	4,367	1,671	5,584	58	213.04	81.52	272.41	13
		2001	5,402	5,006	5,850	48	260.32	241.23	281.90	8
		2002	4,554	1,541	6,073	54	216.08	73.11	288.15	13
		2003	4,663	1,481	6,329	52	217.14	68.96	294.74	12
		2004	4,747	1,427	6,605	52	216.64	65.13	301.46	12
		2005	4,706	1,375	6,899	54	210.46	61.50	308.57	12
		2006	4,644	1,319	7,204	55	203.83	57.89	316.18	14
		2007	4,541	1,258	7,490	55	196.54	54.45	324.16	14
	Female	1992	1,915	1,785	2,109	68	96.46	89.95	106.24	36
		1993	1,759	984	2,111	73	88.27	49.38	105.98	34
		1994	2,119	1,989	2,248	67	105.87	99.38	112.33	28
		1995	1,890	915	2,462	74	94.20	45.58	122.69	29
		1996	1,924	881	2,536	73	95.32	43.67	125.66	28
		1997	1,940	849	2,539	75	95.62	41.83	125.16	27
		1998	1,949	816	2,499	76	95.43	39.97	122.34	24
		1999	2,341	2,194	2,424	65	113.71	106.60	117.75	15
		2000	1,959	750	2,505	75	94.47	36.15	120.79	25
		2001	2,415	2,238	2,615	65	115.14	106.69	124.68	15
		2002	2,024	685	2,699	74	95.29	32.24	127.07	23
		2003	2,055	653	2,789	75	95.15	30.22	129.16	24
		2004	2,068	622	2,877	74	94.05	28.27	130.87	23
		2005	2,019	590	2,961	76	90.14	26.34	132.16	23
		2006	1,956	555	3,033	78	85.81	24.37	133.10	27
		2007	1,868	518	3,081	79	80.97	22.43	133.55	29

Metropolitan Statistical Area	PWID Population	Year	Number	Min	Max	Rank	Rate	Min	Max	Rank
Allentown--Bethlehem--Easton, PA	Young (15-29)	1992	2,071	1,931	2,281	53	168.53	157.15	185.61	13
		1993	1,760	985	2,113	56	145.03	81.14	174.13	17
		1994	2,030	1,906	2,154	51	169.13	158.76	179.46	12
		1995	1,788	865	2,329	56	150.52	72.82	196.03	13
		1996	1,844	845	2,431	58	156.07	71.51	205.75	12
		1997	1,924	842	2,519	56	163.49	71.51	213.99	9
		1998	2,031	850	2,604	56	173.05	72.47	221.86	8
		1999	2,585	2,423	2,676	48	221.89	208.02	229.78	3
		2000	2,300	880	2,941	49	198.72	76.04	254.10	5
		2001	3,010	2,789	3,259	43	257.63	238.74	278.99	4
		2002	2,662	901	3,550	47	223.85	75.74	298.51	5
		2003	2,824	897	3,833	46	231.22	73.43	313.85	5
		2004	2,931	881	4,078	44	232.49	69.90	323.52	6
		2005	2,903	848	4,256	46	223.60	65.35	327.84	8
		2006	2,793	793	4,333	52	209.52	59.51	325.01	11
		2007	2,584	716	4,261	56	191.37	53.02	315.63	13
	Old (30-64)	1992	4,090	3,814	4,504	72	151.23	141.02	166.56	40
		1993	3,896	2,180	4,678	75	142.35	79.64	170.91	37
		1994	4,781	4,488	5,074	64	172.57	161.99	183.12	26
		1995	4,285	2,073	5,581	67	153.30	74.17	199.65	31
		1996	4,333	1,985	5,713	66	153.40	70.29	202.23	29
		1997	4,303	1,882	5,633	66	150.89	66.00	197.50	26
		1998	4,227	1,770	5,420	68	146.62	61.41	187.98	27
		1999	4,938	4,629	5,113	62	168.70	158.15	174.70	18
		2000	4,007	1,533	5,124	69	135.10	51.69	172.74	31
		2001	4,789	4,438	5,186	62	159.42	147.73	172.64	17
		2002	3,906	1,322	5,209	68	128.41	43.45	171.23	27
		2003	3,889	1,235	5,279	67	126.06	40.03	171.11	27
		2004	3,885	1,168	5,406	68	124.15	37.32	172.76	27
		2005	3,826	1,118	5,610	67	120.41	35.19	176.54	25
		2006	3,812	1,083	5,913	67	118.21	33.58	183.38	25
		2007	3,827	1,060	6,311	64	117.11	32.44	193.15	21

Metropolitan Statistical Area	PWID Population	Year	Number	Min	Max	Rank	Rate	Min	Max	Rank
Ann Arbor, MI	Total	1992	1,413	1,235	1,504	99	39.48	34.51	42.04	99
		1993	1,340	1,248	1,447	99	37.19	34.63	40.14	99
		1994	1,294	1,218	1,396	100	35.47	33.39	38.25	100
		1995	1,285	1,091	1,398	100	34.57	29.35	37.61	100
		1996	1,267	978	1,443	100	33.39	25.78	38.03	100
		1997	1,254	897	1,480	100	32.44	23.21	38.28	100
		1998	1,247	847	1,514	100	31.74	21.55	38.54	100
		1999	1,208	988	1,430	100	30.06	24.58	35.59	100
		2000	1,366	1,174	1,550	100	33.24	28.58	37.74	99
		2001	1,336	1,141	1,532	100	31.89	27.22	36.55	100
		2002	1,489	1,108	1,634	100	34.86	25.93	38.26	99
		2003	1,542	1,069	1,770	100	35.63	24.70	40.89	99
		2004	1,565	1,034	1,789	100	35.62	23.53	40.72	100
		2005	1,577	998	1,937	100	35.41	22.41	43.50	100
		2006	1,571	959	2,080	100	34.96	21.34	46.29	100
		2007	1,554	914	2,238	100	34.47	20.28	49.63	100
	Non-Hispanic White	1992	908	794	967	99	29.21	25.54	31.11	98
		1993	846	788	913	99	27.11	25.24	29.26	98
		1994	811	763	875	99	25.76	24.25	27.79	99
		1995	807	686	878	99	25.29	21.47	27.51	99
		1996	804	621	916	99	24.77	19.13	28.21	99
		1997	808	578	953	100	24.52	17.55	28.94	100
		1998	818	555	993	100	24.49	16.62	29.73	100
		1999	806	659	955	100	23.71	19.39	28.08	100
		2000	927	797	1,053	99	26.77	23.01	30.39	96
		2001	920	785	1,055	99	26.08	22.27	29.90	97
		2002	1,035	770	1,136	99	28.87	21.48	31.69	96
		2003	1,077	747	1,236	99	29.69	20.58	34.07	96
		2004	1,090	720	1,246	99	29.64	19.58	33.89	96
		2005	1,086	687	1,334	99	29.21	18.49	35.89	99
		2006	1,058	646	1,401	99	28.28	17.27	37.45	100
		2007	1,010	594	1,455	99	26.96	15.86	38.83	100

Metropolitan Statistical Area	PWID Population	Year	Number	Min	Max	Rank	Rate	Min	Max	Rank
Ann Arbor, MI	Non-Hispanic Black	1992	447	391	476	92	178.61	156.15	190.20	82
		1993	430	400	464	93	167.74	156.19	181.05	79
		1994	411	386	443	93	155.82	146.68	168.07	83
		1995	396	336	431	94	145.49	123.52	158.28	80
		1996	374	288	425	94	133.39	102.98	151.91	81
		1997	349	250	412	94	120.52	86.24	142.23	81
		1998	326	221	396	94	109.59	74.39	133.04	86
		1999	295	241	349	94	96.10	78.59	113.79	88
		2000	312	268	354	93	98.72	84.88	112.08	85
		2001	287	245	329	94	90.00	76.84	103.17	86
		2002	304	226	334	93	93.78	69.76	102.93	83
		2003	306	212	351	93	93.54	64.85	107.34	80
		2004	308	203	352	93	92.91	61.39	106.22	75
		2005	317	201	389	93	93.85	59.41	115.31	74
		2006	334	204	442	92	97.21	59.35	128.71	74
		2007	362	213	522	92	105.37	61.99	151.74	71
	Hispanic	1992	16	14	17	99	18.23	15.94	19.42	100
		1993	17	16	18	97	18.76	17.47	20.25	97
		1994	18	17	19	98	18.78	17.68	20.25	98
		1995	18	15	20	97	18.56	15.75	20.19	95
		1996	18	14	21	98	17.57	13.56	20.01	95
		1997	18	13	21	98	16.49	11.80	19.46	94
		1998	17	12	21	99	15.40	10.45	18.69	96
		1999	16	13	19	99	13.72	11.22	16.25	98
		2000	18	15	20	97	14.18	12.19	16.09	95
		2001	16	14	19	98	12.64	10.79	14.49	98
		2002	17	13	19	97	12.98	9.65	14.24	96
		2003	18	12	20	97	12.64	8.77	14.51	99
		2004	17	12	20	97	12.28	8.11	14.04	100
		2005	18	11	22	97	12.17	7.71	14.96	100
		2006	18	11	24	99	12.19	7.44	16.15	99
		2007	19	11	27	99	12.54	7.38	18.06	98

Metropolitan Statistical Area	PWID Population	Year	Number	Min	Max	Rank	Rate	Min	Max	Rank
Ann Arbor, MI	Male	1992	849	742	904	99	47.09	41.16	50.14	99
		1993	820	764	885	99	45.13	42.02	48.70	99
		1994	800	753	863	100	43.45	40.90	46.87	100
		1995	799	678	869	100	42.53	36.11	46.27	100
		1996	788	609	898	100	41.10	31.73	46.81	100
		1997	779	557	919	100	39.84	28.51	47.02	100
		1998	772	524	937	100	38.86	26.38	47.18	100
		1999	745	609	882	100	36.63	29.95	43.37	100
		2000	839	722	953	100	40.37	34.71	45.83	100
		2001	821	701	941	100	38.73	33.06	44.39	100
		2002	917	682	1,006	100	42.38	31.52	46.51	100
		2003	957	663	1,098	100	43.63	30.25	50.07	100
		2004	984	650	1,125	100	44.16	29.18	50.49	100
		2005	1,011	640	1,243	100	44.71	28.30	54.93	100
		2006	1,035	632	1,371	100	45.33	27.67	60.02	99
		2007	1,060	623	1,526	100	46.31	27.25	66.69	99
	Female	1992	581	508	619	97	32.73	28.62	34.86	92
		1993	547	509	591	98	30.62	28.51	33.05	94
		1994	527	496	568	99	29.17	27.46	31.46	98
		1995	524	445	570	99	28.53	24.22	31.03	95
		1996	520	401	592	99	27.70	21.38	31.54	97
		1997	517	370	611	99	27.08	19.38	31.96	98
		1998	518	351	628	100	26.65	18.09	32.35	98
		1999	503	412	596	100	25.36	20.74	30.02	99
		2000	570	490	647	100	28.08	24.14	31.88	98
		2001	555	474	637	100	26.83	22.90	30.75	99
		2002	613	456	673	100	29.09	21.64	31.93	98
		2003	624	433	716	100	29.22	20.26	33.54	98
		2004	616	407	704	100	28.45	18.80	32.53	98
		2005	597	378	734	100	27.26	17.26	33.49	100
		2006	564	345	747	100	25.54	15.59	33.82	100
		2007	521	306	750	100	23.45	13.80	33.77	100

Metropolitan Statistical Area	PWID Population	Year	Number	Min	Max	Rank	Rate	Min	Max	Rank
Ann Arbor, MI	Young (15-29)	1992	438	383	467	99	32.06	28.03	34.14	97
		1993	396	369	428	99	29.41	27.39	31.74	97
		1994	376	354	406	99	28.14	26.49	30.35	98
		1995	378	321	411	97	28.12	23.88	30.59	97
		1996	385	297	439	97	28.35	21.88	32.28	97
		1997	401	287	474	97	29.27	20.95	34.54	96
		1998	425	288	516	96	30.90	20.97	37.51	96
		1999	441	361	522	100	31.72	25.94	37.55	98
		2000	535	460	607	97	38.13	32.78	43.28	93
		2001	560	478	642	99	39.24	33.50	44.99	98
		2002	663	493	727	97	45.68	33.98	50.14	91
		2003	722	500	828	98	49.07	34.02	56.31	89
		2004	760	502	869	99	50.80	33.56	58.07	93
		2005	784	496	963	99	51.59	32.66	63.39	95
		2006	785	479	1,039	100	51.02	31.15	67.56	96
		2007	765	450	1,101	100	49.54	29.15	71.34	96
	Old (30-64)	1992	1,005	879	1,070	99	45.46	39.74	48.41	98
		1993	973	906	1,050	99	43.11	40.15	46.53	97
		1994	947	891	1,021	100	40.98	38.58	44.20	100
		1995	939	797	1,022	100	39.59	33.62	43.07	99
		1996	918	709	1,045	100	37.70	29.10	42.93	99
		1997	893	639	1,054	100	35.81	25.62	42.26	99
		1998	867	589	1,053	100	33.95	23.05	41.22	99
		1999	813	665	963	100	30.96	25.31	36.65	100
		2000	885	761	1,005	100	32.72	28.13	37.14	98
		2001	828	707	949	99	29.97	25.59	34.35	99
		2002	879	654	964	99	31.15	23.17	34.19	98
		2003	864	599	991	99	30.22	20.95	34.68	99
		2004	831	549	950	99	28.69	18.95	32.80	99
		2005	796	504	978	99	27.12	17.17	33.32	99
		2006	757	462	1,003	100	25.63	15.65	33.94	100
		2007	723	425	1,041	100	24.37	14.34	35.09	100

Metropolitan Statistical Area	PWID Population	Year	Number	Min	Max	Rank	Rate	Min	Max	Rank
Atlanta, GA	Total	1992	21,482	16,827	24,457	22	97.27	76.19	110.74	56
		1993	19,846	16,300	24,447	20	87.00	71.46	107.17	59
		1994	20,499	15,836	24,421	19	86.55	66.86	103.11	65
		1995	18,901	15,275	24,234	21	77.04	62.26	98.78	69
		1996	18,320	14,637	23,910	21	72.20	57.68	94.23	71
		1997	17,433	13,934	22,450	22	66.44	53.11	85.56	76
		1998	16,502	13,462	20,604	24	60.81	49.61	75.93	78
		1999	16,139	12,929	18,822	26	57.56	46.11	67.13	84
		2000	15,019	12,981	17,772	27	51.79	44.76	61.28	88
		2001	15,287	13,023	17,711	27	50.96	43.41	59.04	94
		2002	15,209	13,778	18,559	26	49.39	44.74	60.26	93
		2003	15,930	13,502	19,737	23	50.48	42.78	62.54	93
		2004	16,859	12,786	20,916	20	51.96	39.41	64.47	94
		2005	17,958	12,111	22,345	19	53.82	36.30	66.97	91
		2006	19,287	11,516	23,879	18	55.93	33.40	69.25	85
		2007	20,564	10,807	25,232	17	58.07	30.52	71.25	81
	Non-Hispanic White	1992	9,041	7,082	10,293	25	58.53	45.85	66.64	66
		1993	8,184	6,722	10,082	25	52.03	42.74	64.09	64
		1994	8,366	6,463	9,967	25	52.01	40.18	61.96	72
		1995	7,700	6,223	9,873	25	47.02	38.00	60.29	72
		1996	7,506	5,997	9,797	25	45.15	36.07	58.92	75
		1997	7,227	5,776	9,307	26	42.89	34.28	55.23	77
		1998	6,954	5,673	8,683	27	40.66	33.17	50.76	79
		1999	6,938	5,558	8,091	28	40.11	32.13	46.78	81
		2000	6,600	5,705	7,810	29	37.60	32.50	44.50	84
		2001	6,874	5,856	7,964	31	38.42	32.73	44.51	88
		2002	6,997	6,339	8,539	27	38.62	34.99	47.13	84
		2003	7,489	6,348	9,279	26	40.86	34.63	50.63	85
		2004	8,083	6,130	10,028	25	43.54	33.02	54.01	85
		2005	8,753	5,903	10,891	22	46.55	31.40	57.93	81
		2006	9,520	5,684	11,787	22	50.01	29.86	61.91	79
		2007	10,234	5,378	12,557	21	53.24	27.98	65.33	77

Metropolitan Statistical Area	PWID Population	Year	Number	Min	Max	Rank	Rate	Min	Max	Rank
Atlanta, GA	Non-Hispanic Black	1992	8,662	6,785	9,862	12	156.16	122.32	177.78	87
		1993	8,745	7,183	10,772	12	149.95	123.16	184.72	85
		1994	9,507	7,345	11,326	11	154.08	119.03	183.56	84
		1995	8,969	7,248	11,500	11	138.07	111.58	177.03	84
		1996	8,702	6,953	11,357	11	127.26	101.68	166.10	82
		1997	8,149	6,513	10,494	11	113.43	90.66	146.08	86
		1998	7,494	6,113	9,356	11	99.27	80.98	123.95	87
		1999	7,058	5,654	8,231	12	89.12	71.39	103.93	89
		2000	6,299	5,444	7,453	14	76.12	65.79	90.07	92
		2001	6,156	5,244	7,132	14	70.89	60.39	82.13	92
		2002	5,922	5,365	7,227	14	65.51	59.35	79.95	92
		2003	6,079	5,153	7,532	14	64.74	54.88	80.22	92
		2004	6,431	4,877	7,979	14	65.47	49.66	81.23	89
		2005	7,020	4,734	8,735	14	68.33	46.08	85.03	89
		2006	7,949	4,746	9,841	12	73.11	43.65	90.52	85
		2007	9,193	4,831	11,280	11	80.98	42.56	99.36	81
	Hispanic	1992	228	178	259	65	39.64	31.05	45.13	91
		1993	209	172	257	65	31.27	25.68	38.52	93
		1994	219	169	261	67	28.14	21.74	33.52	94
		1995	208	168	267	65	22.39	18.09	28.71	94
		1996	211	169	276	64	19.19	15.33	25.04	94
		1997	214	171	275	64	16.26	13.00	20.94	95
		1998	217	177	272	65	14.41	11.76	18.00	97
		1999	231	185	269	65	13.19	10.57	15.39	99
		2000	235	203	278	65	11.72	10.13	13.87	99
		2001	262	223	304	65	12.11	10.31	14.03	99
		2002	288	261	351	64	12.46	11.28	15.20	99
		2003	333	283	413	63	13.64	11.56	16.90	96
		2004	391	296	484	61	15.07	11.43	18.70	96
		2005	460	310	573	60	16.75	11.29	20.84	96
		2006	547	326	677	58	18.60	11.11	23.03	94
		2007	644	338	790	58	20.60	10.82	25.27	93

Metropolitan Statistical Area	PWID Population	Year	Number	Min	Max	Rank	Rate	Min	Max	Rank
Atlanta, GA	Male	1992	14,919	11,686	16,985	18	137.34	107.58	156.36	53
		1993	13,689	11,243	16,863	19	121.80	100.04	150.03	55
		1994	14,022	10,833	16,705	18	119.98	92.69	142.94	59
		1995	12,808	10,351	16,422	19	105.67	85.40	135.48	65
		1996	12,292	9,821	16,042	20	97.94	78.25	127.82	67
		1997	11,579	9,255	14,912	22	89.06	71.19	114.69	76
		1998	10,856	8,856	13,554	23	80.62	65.77	100.66	77
		1999	10,525	8,432	12,275	24	75.51	60.49	88.06	84
		2000	9,726	8,406	11,509	25	67.28	58.15	79.61	86
		2001	9,852	8,392	11,414	27	65.84	56.09	76.28	92
		2002	9,781	8,861	11,936	24	63.75	57.75	77.79	88
		2003	10,259	8,695	12,711	23	65.33	55.38	80.95	89
		2004	10,915	8,278	13,542	19	67.47	51.17	83.71	87
		2005	11,739	7,916	14,607	18	70.79	47.74	88.09	81
		2006	12,788	7,635	15,833	18	74.56	44.52	92.31	76
		2007	13,896	7,303	17,050	16	79.04	41.54	96.98	74
	Female	1992	6,280	4,920	7,150	25	55.96	43.84	63.71	66
		1993	6,142	5,045	7,566	24	53.08	43.59	65.38	68
		1994	6,652	5,139	7,925	21	55.44	42.83	66.05	72
		1995	6,376	5,153	8,175	22	51.37	41.52	65.87	71
		1996	6,375	5,093	8,320	22	49.71	39.71	64.87	74
		1997	6,212	4,965	7,999	22	46.93	37.51	60.43	78
		1998	5,981	4,879	7,468	22	43.75	35.69	54.62	79
		1999	5,913	4,737	6,896	22	41.94	33.60	48.92	89
		2000	5,531	4,781	6,545	25	38.03	32.87	45.00	89
		2001	5,628	4,795	6,521	24	37.44	31.89	43.37	89
		2002	5,569	5,046	6,796	25	36.04	32.65	43.98	92
		2003	5,774	4,894	7,154	23	36.41	30.87	45.12	92
		2004	6,021	4,566	7,470	23	37.01	28.07	45.92	92
		2005	6,292	4,243	7,829	23	37.48	25.28	46.64	92
		2006	6,601	3,942	8,173	21	38.09	22.74	47.16	91
		2007	6,849	3,600	8,404	21	38.41	20.19	47.13	88

Metropolitan Statistical Area	PWID Population	Year	Number	Min	Max	Rank	Rate	Min	Max	Rank
Atlanta, GA	Young (15-29)	1992	5,833	4,569	6,641	19	77.34	60.58	88.05	57
		1993	4,848	3,982	5,972	22	63.39	52.07	78.09	61
		1994	4,636	3,581	5,523	22	59.53	45.99	70.92	68
		1995	4,068	3,287	5,216	24	51.12	41.31	65.54	71
		1996	3,850	3,076	5,024	25	47.15	37.67	61.54	78
		1997	3,659	2,925	4,712	28	43.51	34.77	56.02	82
		1998	3,528	2,878	4,405	31	40.65	33.16	50.75	86
		1999	3,571	2,860	4,164	31	39.94	32.00	46.58	92
		2000	3,481	3,009	4,120	33	37.83	32.70	44.76	94
		2001	3,745	3,190	4,338	32	40.27	34.30	46.65	97
		2002	3,956	3,584	4,828	32	42.31	38.33	51.63	96
		2003	4,407	3,735	5,460	28	46.72	39.60	57.88	95
		2004	4,951	3,755	6,143	23	51.32	38.92	63.68	92
		2005	5,571	3,757	6,932	19	56.95	38.41	70.86	89
		2006	6,272	3,745	7,766	17	61.90	36.96	76.63	86
		2007	6,941	3,648	8,516	16	66.99	35.21	82.20	80
	Old (30-64)	1992	15,725	12,317	17,903	20	108.12	84.69	123.10	56
		1993	15,238	12,516	18,771	19	100.49	82.54	123.79	57
		1994	16,196	12,512	19,295	17	101.88	78.70	121.37	60
		1995	15,168	12,258	19,448	18	91.51	73.95	117.32	64
		1996	14,791	11,817	19,304	19	85.94	68.66	112.16	67
		1997	14,053	11,232	18,097	20	78.83	63.00	101.51	71
		1998	13,199	10,767	16,480	22	71.51	58.34	89.29	73
		1999	12,740	10,206	14,858	23	66.71	53.44	77.80	79
		2000	11,650	10,069	13,785	25	58.84	50.86	69.63	82
		2001	11,616	9,895	13,457	25	56.12	47.81	65.02	88
		2002	11,303	10,240	13,793	24	52.71	47.75	64.32	86
		2003	11,590	9,824	14,360	21	52.38	44.40	64.90	84
		2004	12,049	9,138	14,948	21	52.85	40.08	65.57	80
		2005	12,687	8,556	15,786	21	53.80	36.28	66.94	77
		2006	13,591	8,115	16,827	19	55.82	33.33	69.11	74
		2007	14,611	7,679	17,928	18	58.33	30.65	71.57	67

Metropolitan Statistical Area	PWID Population	Year	Number	Min	Max	Rank	Rate	Min	Max	Rank
Austin--San Marcos, TX	Total	1992	15,264	13,758	16,376	33	238.41	214.89	255.77	9
		1993	13,957	7,920	18,086	31	209.35	118.80	271.29	11
		1994	16,680	13,141	20,537	26	239.87	188.98	295.35	5
		1995	14,699	7,332	22,199	29	201.88	100.70	304.89	8
		1996	14,440	6,988	21,953	29	190.66	92.27	289.85	8
		1997	13,689	6,625	19,846	30	174.17	84.29	252.51	11
		1998	12,937	6,380	17,387	31	157.92	77.88	212.24	17
		1999	14,234	11,605	16,182	32	165.89	135.26	188.60	15
		2000	11,845	6,395	16,692	34	131.05	70.75	184.68	31
		2001	13,166	11,187	17,088	33	139.26	118.32	180.75	22
		2002	10,986	6,078	17,367	39	113.80	62.96	179.90	36
		2003	10,569	5,595	17,632	40	107.33	56.82	179.05	39
		2004	10,118	5,060	17,973	40	100.36	50.19	178.27	44
		2005	9,716	4,552	18,435	43	93.54	43.82	177.49	48
		2006	9,470	4,037	19,146	42	87.74	37.40	177.38	55
		2007	9,233	3,483	19,904	46	82.23	31.02	177.27	59
	Non-Hispanic White	1992	7,509	6,768	8,056	29	173.42	156.31	186.06	13
		1993	6,731	3,820	8,722	29	150.70	85.52	195.29	16
		1994	7,994	6,297	9,842	26	173.07	136.35	213.10	12
		1995	7,077	3,530	10,688	26	147.87	73.76	223.32	15
		1996	7,044	3,409	10,708	27	142.89	69.15	217.23	15
		1997	6,802	3,292	9,862	29	134.28	64.99	194.68	15
		1998	6,569	3,240	8,829	31	125.55	61.92	168.74	19
		1999	7,391	6,026	8,402	25	136.15	111.01	154.79	15
		2000	6,279	3,390	8,849	32	111.06	59.96	156.50	23
		2001	7,101	6,033	9,216	28	120.99	102.80	157.04	21
		2002	5,997	3,318	9,480	34	101.13	55.95	159.88	31
		2003	5,799	3,070	9,674	36	96.67	51.17	161.27	34
		2004	5,532	2,766	9,826	39	90.70	45.36	161.10	37
		2005	5,237	2,453	9,937	41	84.00	39.35	159.37	44
		2006	4,969	2,118	10,046	42	77.33	32.96	156.34	48
		2007	4,644	1,752	10,011	54	70.01	26.41	150.92	60

Metropolitan Statistical Area	PWID Population	Year	Number	Min	Max	Rank	Rate	Min	Max	Rank
Austin--San Marcos, TX	Non-Hispanic Black	1992	3,507	3,161	3,762	28	657.84	592.94	705.77	7
		1993	3,023	1,715	3,917	31	552.16	313.34	715.53	9
		1994	3,376	2,660	4,157	28	596.77	470.15	734.79	6
		1995	2,762	1,378	4,171	33	469.51	234.20	709.08	12
		1996	2,509	1,214	3,815	35	415.95	201.29	632.33	12
		1997	2,199	1,064	3,188	39	355.63	172.11	515.59	14
		1998	1,927	950	2,590	44	302.62	149.25	406.72	19
		1999	1,979	1,614	2,250	43	300.32	244.86	341.44	19
		2000	1,554	839	2,190	54	226.04	122.04	318.54	33
		2001	1,653	1,405	2,146	52	233.34	198.27	302.87	25
		2002	1,345	744	2,127	58	186.16	102.99	294.30	39
		2003	1,290	683	2,152	59	175.99	93.17	293.61	40
		2004	1,263	632	2,244	60	168.96	84.50	300.12	43
		2005	1,278	599	2,425	60	166.28	77.90	315.51	42
		2006	1,357	578	2,743	57	167.57	71.43	338.77	45
		2007	1,491	562	3,214	54	177.97	67.14	383.66	44
	Hispanic	1992	3,506	3,160	3,761	23	262.94	237.00	282.10	22
		1993	3,392	1,925	4,396	21	238.05	135.09	308.48	25
		1994	4,185	3,297	5,153	18	275.13	216.75	338.76	16
		1995	3,728	1,860	5,631	19	228.25	113.86	344.72	21
		1996	3,642	1,763	5,537	19	208.82	101.05	317.45	20
		1997	3,389	1,640	4,913	23	182.74	88.44	264.93	25
		1998	3,114	1,536	4,185	24	157.51	77.68	211.69	31
		1999	3,312	2,701	3,766	20	156.64	127.72	178.09	24
		2000	2,660	1,436	3,748	25	116.35	62.82	163.96	36
		2001	2,858	2,428	3,709	26	117.65	99.96	152.70	36
		2002	2,320	1,283	3,667	29	91.59	50.67	144.79	45
		2003	2,193	1,161	3,659	33	83.50	44.20	139.30	46
		2004	2,095	1,048	3,722	34	76.86	38.44	136.52	48
		2005	2,047	959	3,885	35	71.83	33.65	136.29	54
		2006	2,080	887	4,206	34	69.44	29.60	140.39	55
		2007	2,174	820	4,686	33	68.77	25.94	148.25	56

Metropolitan Statistical Area	PWID Population	Year	Number	Min	Max	Rank	Rate	Min	Max	Rank
Austin--San Marcos, TX	Male	1992	10,840	9,771	11,630	29	333.18	300.31	357.45	6
		1993	9,839	5,583	12,750	29	290.12	164.64	375.96	8
		1994	11,630	9,162	14,320	25	327.83	258.28	403.65	2
		1995	10,108	5,042	15,266	28	271.87	135.61	410.59	8
		1996	9,773	4,730	14,857	28	252.25	122.07	383.48	9
		1997	9,107	4,407	13,203	31	226.03	109.39	327.70	11
		1998	8,459	4,172	11,368	31	201.01	99.14	270.15	19
		1999	9,155	7,465	10,409	32	207.25	168.98	235.63	18
		2000	7,512	4,056	10,586	35	161.14	87.00	227.08	35
		2001	8,262	7,020	10,724	33	169.28	143.83	219.71	33
		2002	6,857	3,793	10,840	39	137.35	75.98	217.13	41
		2003	6,602	3,495	11,015	40	129.55	68.58	216.13	41
		2004	6,374	3,188	11,322	40	122.05	61.04	216.81	44
		2005	6,224	2,916	11,809	40	115.48	54.10	219.11	52
		2006	6,222	2,652	12,580	39	111.08	47.35	224.58	54
		2007	6,275	2,367	13,528	42	107.66	40.61	232.09	55
	Female	1992	4,476	4,034	4,802	36	142.13	128.11	152.49	15
		1993	4,155	2,358	5,384	34	126.84	71.98	164.37	17
		1994	5,090	4,010	6,268	29	149.45	117.74	184.02	11
		1995	4,631	2,310	6,995	31	129.99	64.84	196.31	14
		1996	4,718	2,283	7,173	31	127.54	61.72	193.89	13
		1997	4,647	2,249	6,737	32	121.31	58.71	175.87	13
		1998	4,560	2,249	6,128	33	114.45	56.44	153.81	15
		1999	5,194	4,235	5,905	31	124.79	101.74	141.87	12
		2000	4,454	2,405	6,277	34	101.76	54.94	143.41	19
		2001	5,067	4,305	6,576	31	110.79	94.14	143.80	17
		2002	4,290	2,373	6,782	35	92.03	50.91	145.49	27
		2003	4,144	2,194	6,914	36	87.23	46.18	145.52	29
		2004	3,935	1,968	6,989	41	80.97	40.49	143.82	33
		2005	3,692	1,729	7,004	42	73.88	34.61	140.18	41
		2006	3,454	1,472	6,984	41	66.53	28.36	134.50	47
		2007	3,165	1,194	6,824	48	58.62	22.11	126.38	55

Metropolitan Statistical Area	PWID Population	Year	Number	Min	Max	Rank	Rate	Min	Max	Rank
Austin--San Marcos, TX	Young (15-29)	1992	5,056	4,557	5,424	23	198.66	179.06	213.13	9
		1993	4,671	2,651	6,054	24	180.21	102.27	233.53	9
		1994	5,602	4,414	6,898	18	210.30	165.68	258.93	5
		1995	4,926	2,457	7,439	20	178.39	88.98	269.41	7
		1996	4,806	2,326	7,307	20	168.17	81.38	255.65	8
		1997	4,511	2,183	6,540	22	152.09	73.60	220.49	13
		1998	4,214	2,078	5,663	23	136.23	67.19	183.09	20
		1999	4,580	3,734	5,207	21	141.37	115.26	160.72	18
		2000	3,768	2,034	5,310	29	110.86	59.85	156.22	37
		2001	4,151	3,527	5,388	29	118.45	100.64	153.74	36
		2002	3,446	1,907	5,448	34	97.70	54.05	154.46	51
		2003	3,317	1,756	5,533	37	93.65	49.58	156.24	54
		2004	3,198	1,599	5,680	40	89.57	44.80	159.11	56
		2005	3,118	1,461	5,916	42	85.99	40.28	163.15	58
		2006	3,115	1,328	6,297	44	83.76	35.70	169.34	62
		2007	3,144	1,186	6,779	44	82.30	31.05	177.43	58
	Old (30-64)	1992	10,251	9,240	10,998	35	265.72	239.50	285.08	9
		1993	9,265	5,258	12,006	35	227.39	129.04	294.67	12
		1994	11,019	8,681	13,567	32	256.87	202.37	316.28	6
		1995	9,718	4,847	14,676	31	215.01	107.25	324.72	9
		1996	9,597	4,644	14,590	31	203.51	98.49	309.38	11
		1997	9,173	4,439	13,299	32	187.47	90.73	271.78	15
		1998	8,756	4,318	11,768	33	171.71	84.69	230.78	18
		1999	9,733	7,936	11,066	33	182.26	148.60	207.21	12
		2000	8,178	4,415	11,524	35	145.01	78.29	204.35	24
		2001	9,161	7,784	11,890	34	153.97	130.83	199.85	21
		2002	7,684	4,251	12,148	37	125.42	69.39	198.28	29
		2003	7,405	3,920	12,354	37	117.43	62.16	195.91	29
		2004	7,071	3,537	12,561	37	108.59	54.31	192.89	32
		2005	6,735	3,155	12,780	38	99.62	46.67	189.03	40
		2006	6,466	2,756	13,071	37	91.38	38.95	184.74	42
		2007	6,149	2,320	13,256	39	83.01	31.31	178.94	46

Metropolitan Statistical Area	PWID Population	Year	Number	Min	Max	Rank	Rate	Min	Max	Rank
Bakersfield, CA	Total	1992	11,714	9,434	16,192	40	317.40	255.62	438.73	2
		1993	10,954	9,111	16,164	39	293.71	244.29	433.38	2
		1994	11,593	8,848	16,419	40	303.54	231.65	429.89	1
		1995	10,741	8,400	16,394	40	279.36	218.49	426.40	2
		1996	10,611	7,978	16,595	40	273.26	205.46	427.36	2
		1997	10,597	7,631	16,985	38	267.23	192.43	428.32	2
		1998	10,799	7,275	18,231	38	266.28	179.39	449.56	2
		1999	12,101	6,938	19,636	34	290.14	166.36	470.80	1
		2000	11,213	6,544	21,127	36	264.41	154.30	498.18	3
		2001	12,937	6,168	22,790	34	298.35	142.25	525.56	2
		2002	11,689	5,868	24,219	36	261.98	131.53	542.83	3
		2003	11,953	5,561	25,782	36	259.68	120.80	560.10	3
		2004	12,196	5,243	27,235	35	256.71	110.36	573.23	3
		2005	12,489	4,917	28,856	33	253.78	99.91	586.35	3
		2006	12,782	4,576	30,543	33	251.14	89.91	600.13	4
		2007	12,966	4,168	32,021	34	248.58	79.91	613.91	4
	Non-Hispanic White	1992	6,844	5,512	9,461	34	303.94	244.78	420.13	1
		1993	5,922	4,926	8,738	36	266.10	221.33	392.65	3
		1994	5,949	4,540	8,426	39	268.18	204.67	379.82	1
		1995	5,365	4,196	8,189	36	245.12	191.71	374.14	3
		1996	5,273	3,965	8,247	36	243.43	183.03	380.70	3
		1997	5,329	3,837	8,541	35	245.51	176.79	393.50	2
		1998	5,558	3,745	9,384	35	255.55	172.16	431.44	1
		1999	6,413	3,677	10,406	32	292.73	167.84	475.00	1
		2000	6,123	3,573	11,536	34	279.69	163.23	526.98	1
		2001	7,254	3,458	12,777	27	329.23	156.98	579.96	1
		2002	6,680	3,354	13,841	29	300.33	150.78	622.30	1
		2003	6,888	3,204	14,857	27	306.49	142.58	661.06	1
		2004	6,987	3,004	15,602	28	307.68	132.27	687.04	1
		2005	6,983	2,749	16,134	29	305.81	120.40	706.57	1
		2006	6,812	2,439	16,277	31	297.50	106.51	710.90	1
		2007	6,383	2,052	15,765	35	279.26	89.77	689.68	2

Metropolitan Statistical Area	PWID Population	Year	Number	Min	Max	Rank	Rate	Min	Max	Rank
Bakersfield, CA	Non-Hispanic Black	1992	1,200	966	1,659	71	585.86	471.84	809.83	11
		1993	994	827	1,466	72	475.31	395.34	701.35	14
		1994	952	726	1,348	77	427.86	326.53	605.97	18
		1995	814	637	1,242	80	359.58	281.23	548.85	23
		1996	756	568	1,182	79	328.67	247.12	514.01	23
		1997	721	520	1,156	79	305.02	219.64	488.89	23
		1998	713	480	1,204	78	291.32	196.26	491.82	21
		1999	784	450	1,273	73	306.75	175.88	497.74	18
		2000	721	421	1,359	73	278.79	162.70	525.28	18
		2001	833	397	1,467	68	312.61	149.05	550.68	15
		2002	757	380	1,569	71	273.75	137.44	567.21	15
		2003	782	364	1,687	71	275.36	128.10	593.91	16
		2004	808	347	1,805	69	277.69	119.38	620.09	15
		2005	838	330	1,937	69	278.84	109.78	644.23	15
		2006	869	311	2,077	70	274.03	98.10	654.83	16
		2007	893	287	2,206	69	273.17	87.81	674.64	16
	Hispanic	1992	3,248	2,616	4,490	25	301.67	242.96	416.99	16
		1993	3,246	2,700	4,789	24	287.10	238.80	423.64	17
		1994	3,473	2,651	4,919	24	287.91	219.72	407.76	15
		1995	3,114	2,435	4,753	26	248.79	194.58	379.74	18
		1996	2,876	2,162	4,498	26	220.72	165.96	345.19	19
		1997	2,618	1,885	4,196	28	191.41	137.83	306.80	21
		1998	2,393	1,612	4,041	28	166.34	112.06	280.82	25
		1999	2,395	1,373	3,886	29	157.60	90.36	255.73	23
		2000	1,997	1,165	3,762	34	126.29	73.70	237.95	34
		2001	2,118	1,010	3,731	34	128.56	61.30	226.46	31
		2002	1,823	915	3,777	36	105.14	52.79	217.85	39
		2003	1,866	868	4,025	35	101.69	47.31	219.34	41
		2004	2,031	873	4,535	35	104.44	44.90	233.22	37
		2005	2,387	940	5,515	29	114.72	45.17	265.06	33
		2006	3,034	1,086	7,251	24	136.97	49.04	327.31	23
		2007	4,111	1,322	10,153	16	176.52	56.74	435.94	20

Metropolitan Statistical Area	PWID Population	Year	Number	Min	Max	Rank	Rate	Min	Max	Rank
Bakersfield, CA	Male	1992	6,796	5,473	9,394	42	354.78	285.73	490.41	3
		1993	6,151	5,116	9,076	42	317.85	264.38	469.01	5
		1994	6,367	4,859	9,018	43	319.36	243.73	452.31	3
		1995	5,828	4,558	8,895	43	290.36	227.09	443.19	5
		1996	5,739	4,315	8,975	43	283.15	212.89	442.82	5
		1997	5,757	4,145	9,227	43	277.87	200.09	445.37	7
		1998	5,928	3,994	10,008	42	279.20	188.10	471.37	7
		1999	6,742	3,866	10,940	39	307.61	176.37	499.14	4
		2000	6,358	3,711	11,980	39	285.47	166.60	537.87	6
		2001	7,475	3,564	13,167	35	327.74	156.26	577.32	4
		2002	6,879	3,454	14,254	38	292.95	147.08	607.01	6
		2003	7,155	3,328	15,432	38	295.49	137.46	637.33	5
		2004	7,404	3,183	16,534	38	296.42	127.43	661.92	5
		2005	7,660	3,016	17,699	38	295.77	116.44	683.36	5
		2006	7,882	2,822	18,834	37	292.74	104.80	699.53	6
		2007	7,990	2,568	19,732	36	288.84	92.85	713.32	7
	Female	1992	4,984	4,014	6,889	32	280.75	226.11	388.08	1
		1993	4,859	4,042	7,170	29	270.79	225.23	399.56	1
		1994	5,283	4,032	7,482	28	289.36	220.83	409.82	1
		1995	4,966	3,884	7,580	27	270.25	211.36	412.49	1
		1996	4,928	3,705	7,706	27	265.46	199.59	415.15	1
		1997	4,900	3,529	7,854	28	258.78	186.34	414.76	1
		1998	4,938	3,327	8,337	28	255.58	172.18	431.49	1
		1999	5,442	3,120	8,830	27	275.00	157.68	446.23	1
		2000	4,939	2,882	9,305	28	245.28	143.14	462.13	1
		2001	5,566	2,654	9,805	26	270.79	129.11	477.00	1
		2002	4,907	2,464	10,168	28	232.21	116.58	481.14	3
		2003	4,901	2,280	10,570	29	224.64	104.50	484.51	3
		2004	4,896	2,105	10,932	29	217.27	93.41	485.17	3
		2005	4,930	1,941	11,391	29	211.49	83.26	488.63	3
		2006	4,995	1,788	11,936	29	208.38	74.60	497.95	3
		2007	5,059	1,626	12,493	28	206.51	66.38	510.00	3

Metropolitan Statistical Area	PWID Population	Year	Number	Min	Max	Rank	Rate	Min	Max	Rank
Bakersfield, CA	Young (15-29)	1992	3,927	3,163	5,429	33	296.62	238.89	410.01	2
		1993	3,265	2,716	4,818	32	248.19	206.44	366.22	1
		1994	3,143	2,399	4,452	37	236.01	180.12	334.26	2
		1995	2,712	2,121	4,140	38	203.82	159.41	311.10	4
		1996	2,554	1,920	3,994	39	190.58	143.29	298.05	5
		1997	2,483	1,788	3,979	43	181.25	130.51	290.50	7
		1998	2,511	1,691	4,239	42	178.71	120.40	301.71	7
		1999	2,839	1,628	4,606	41	195.53	112.11	317.27	7
		2000	2,690	1,570	5,069	44	181.51	105.92	341.98	12
		2001	3,208	1,530	5,652	37	209.89	100.08	369.73	8
		2002	3,019	1,516	6,256	42	189.46	95.12	392.57	9
		2003	3,231	1,503	6,968	39	193.84	90.18	418.10	12
		2004	3,455	1,485	7,714	38	197.13	84.75	440.19	13
		2005	3,703	1,458	8,556	35	200.49	78.93	463.21	13
		2006	3,953	1,415	9,445	35	204.34	73.15	488.29	12
		2007	4,156	1,336	10,263	32	208.30	66.96	514.43	12
	Old (30-64)	1992	7,911	6,371	10,936	42	334.28	269.22	462.06	2
		1993	7,768	6,461	11,462	40	321.79	267.65	474.82	2
		1994	8,515	6,498	12,060	40	342.31	261.24	484.81	1
		1995	8,086	6,324	12,342	40	321.66	251.57	490.97	2
		1996	8,122	6,107	12,703	38	319.38	240.14	499.49	2
		1997	8,193	5,899	13,131	36	315.64	227.28	505.90	3
		1998	8,384	5,648	14,155	35	316.33	213.11	534.05	3
		1999	9,388	5,383	15,233	34	345.27	197.96	560.25	2
		2000	8,652	5,049	16,302	33	313.63	183.03	590.93	3
		2001	9,887	4,714	17,417	29	352.15	167.90	620.32	2
		2002	8,812	4,424	18,260	32	307.26	154.26	636.66	4
		2003	8,858	4,121	19,106	31	301.66	140.33	650.65	4
		2004	8,853	3,806	19,769	29	295.24	126.93	659.28	4
		2005	8,851	3,484	20,449	28	287.91	113.35	665.20	4
		2006	8,814	3,155	21,062	28	279.35	100.01	667.54	4
		2007	8,671	2,787	21,413	28	269.21	86.54	664.85	4

Metropolitan Statistical Area	PWID Population	Year	Number	Min	Max	Rank	Rate	Min	Max	Rank
Baltimore, MD	Total	1992	30,776	19,280	40,482	7	188.62	118.16	248.10	18
		1993	34,566	20,301	42,602	6	211.33	124.12	260.46	10
		1994	34,566	21,762	44,825	6	210.13	132.29	272.49	13
		1995	37,446	23,109	47,026	6	226.56	139.82	284.52	6
		1996	39,323	25,482	49,297	5	236.27	153.11	296.19	5
		1997	41,613	29,258	51,501	5	248.76	174.90	307.87	4
		1998	44,091	32,859	53,683	5	262.55	195.66	319.66	3
		1999	47,873	36,195	56,124	4	282.73	213.76	331.45	2
		2000	49,971	39,559	57,809	5	292.36	231.44	338.22	1
		2001	53,777	42,836	59,601	4	311.28	247.95	344.99	1
		2002	56,158	46,030	63,228	4	321.17	263.25	361.60	1
		2003	58,353	47,090	67,836	4	329.95	266.26	383.57	1
		2004	59,271	44,419	72,607	4	332.14	248.91	406.87	1
		2005	59,435	38,593	77,677	4	330.23	214.43	431.59	1
		2006	59,508	32,362	83,014	4	327.98	178.36	457.53	1
		2007	59,113	25,142	88,079	4	324.57	138.05	483.61	1
	Non-Hispanic White	1992	7,727	4,840	10,163	27	67.34	42.19	88.58	57
		1993	9,108	5,349	11,225	22	79.77	46.85	98.31	47
		1994	9,555	6,015	12,390	22	83.72	52.71	108.56	46
		1995	10,845	6,693	13,620	15	95.13	58.71	119.47	37
		1996	11,908	7,717	14,929	13	104.41	67.66	130.90	29
		1997	13,140	9,239	16,263	9	115.41	81.15	142.84	21
		1998	14,470	10,784	17,618	7	127.31	94.88	155.00	17
		1999	16,265	12,297	19,068	5	142.77	107.94	167.37	12
		2000	17,500	13,854	20,245	5	152.90	121.04	176.88	6
		2001	19,320	15,389	21,412	5	168.02	133.84	186.22	7
		2002	20,589	16,876	23,181	5	178.10	145.98	200.52	5
		2003	21,709	17,519	25,238	5	186.99	150.90	217.38	5
		2004	22,242	16,668	27,246	5	191.35	143.41	234.41	6
		2005	22,351	14,514	29,211	5	192.22	124.81	251.21	6
		2006	22,274	12,113	31,072	4	191.84	104.33	267.62	7
		2007	21,864	9,299	32,577	4	189.13	80.44	281.81	7

Metropolitan Statistical Area	PWID Population	Year	Number	Min	Max	Rank	Rate	Min	Max	Rank
Baltimore, MD	Non-Hispanic Black	1992	20,385	12,770	26,813	4	483.93	303.16	636.53	17
		1993	22,802	13,392	28,103	2	534.38	313.84	658.61	11
		1994	22,560	14,204	29,256	2	521.36	328.24	676.09	8
		1995	24,048	14,840	30,199	2	548.23	338.33	688.48	8
		1996	24,731	16,026	31,004	2	554.99	359.65	695.75	6
		1997	25,535	17,954	31,602	2	565.52	397.61	699.89	5
		1998	26,330	19,622	32,058	2	576.98	429.99	702.49	5
		1999	27,788	21,009	32,577	1	600.64	454.12	704.15	4
		2000	28,207	22,330	32,632	1	602.99	477.34	697.56	3
		2001	29,594	23,573	32,799	1	622.60	495.93	690.03	3
		2002	30,269	24,810	34,080	1	625.14	512.40	703.85	3
		2003	31,021	25,033	36,062	1	628.74	507.38	730.92	4
		2004	31,359	23,502	38,415	1	624.72	468.18	765.28	4
		2005	31,639	20,545	41,350	1	619.83	402.48	810.06	4
		2006	32,255	17,541	44,995	1	619.83	337.08	864.66	4
		2007	33,016	14,042	49,194	1	626.85	266.61	934.02	4
	Hispanic	1992	172	108	227	71	74.67	46.77	98.21	68
		1993	174	102	215	68	71.29	41.87	87.86	65
		1994	165	104	214	72	63.84	40.19	82.79	76
		1995	176	109	222	69	65.38	40.35	82.11	66
		1996	190	123	238	66	66.98	43.41	83.97	64
		1997	213	150	264	65	70.97	49.90	87.83	58
		1998	246	184	300	63	77.64	57.86	94.53	54
		1999	298	225	349	62	88.44	66.87	103.68	51
		2000	351	278	406	61	98.04	77.61	113.42	43
		2001	432	344	479	58	112.94	89.96	125.17	37
		2002	518	425	584	56	126.93	104.04	142.91	31
		2003	619	500	720	55	142.45	114.96	165.60	22
		2004	720	540	882	53	155.47	116.51	190.45	20
		2005	820	532	1,071	52	166.12	107.87	217.11	19
		2006	920	500	1,283	52	174.38	94.83	243.26	19
		2007	1,007	428	1,500	49	179.97	76.55	268.16	19

Metropolitan Statistical Area	PWID Population	Year	Number	Min	Max	Rank	Rate	Min	Max	Rank
Baltimore, MD	Male	1992	18,827	11,794	24,764	7	235.66	147.63	309.97	23
		1993	21,182	12,440	26,106	8	264.83	155.54	326.40	11
		1994	21,219	13,359	27,517	6	263.93	166.16	342.26	15
		1995	23,024	14,209	28,914	6	285.21	176.01	358.17	7
		1996	24,208	15,687	30,348	6	298.05	193.15	373.65	4
		1997	25,637	18,025	31,728	5	314.39	221.05	389.09	4
		1998	27,165	20,245	33,074	5	332.44	247.75	404.76	2
		1999	29,471	22,282	34,550	5	358.06	270.71	419.77	1
		2000	30,706	24,307	35,522	5	369.84	292.77	427.85	1
		2001	32,943	26,241	36,511	5	392.66	312.77	435.18	1
		2002	34,247	28,070	38,558	5	403.35	330.61	454.13	1
		2003	35,368	28,541	41,116	4	412.02	332.49	478.98	1
		2004	35,639	26,708	43,657	4	411.78	308.60	504.43	1
		2005	35,378	22,972	46,236	4	405.31	263.18	529.71	1
		2006	34,981	19,024	48,799	4	397.22	216.02	554.12	1
		2007	34,224	14,556	50,994	4	386.90	164.56	576.48	2
	Female	1992	11,808	7,397	15,532	5	141.80	88.83	186.52	16
		1993	13,310	7,817	16,404	5	159.24	93.52	196.27	6
		1994	13,354	8,408	17,318	5	158.79	99.97	205.91	7
		1995	14,514	8,957	18,227	5	171.65	105.93	215.56	5
		1996	15,291	9,909	19,170	4	179.44	116.28	224.96	4
		1997	16,236	11,416	20,094	4	189.37	133.15	234.37	4
		1998	17,266	12,867	21,022	4	200.24	149.23	243.80	4
		1999	18,822	14,231	22,066	3	216.29	163.53	253.57	3
		2000	19,735	15,623	22,830	4	224.52	177.74	259.74	3
		2001	21,346	17,003	23,658	3	240.21	191.34	266.22	3
		2002	22,420	18,377	25,243	3	249.26	204.30	280.64	1
		2003	23,451	18,925	27,262	3	257.67	207.93	299.54	1
		2004	24,001	17,987	29,401	2	261.16	195.72	319.91	1
		2005	24,277	15,764	31,728	2	261.91	170.06	342.29	1
		2006	24,548	13,350	34,244	1	262.90	142.97	366.74	1
		2007	24,661	10,489	36,744	1	263.28	111.98	392.28	1

Metropolitan Statistical Area	PWID Population	Year	Number	Min	Max	Rank	Rate	Min	Max	Rank
Baltimore, MD	Young (15-29)	1992	7,774	4,870	10,225	12	148.67	93.13	195.55	16
		1993	8,455	4,966	10,420	7	166.08	97.54	204.69	12
		1994	8,323	5,240	10,793	7	167.38	105.38	217.06	14
		1995	9,003	5,556	11,307	6	184.28	113.72	231.42	6
		1996	9,552	6,190	11,975	6	197.45	127.95	247.53	3
		1997	10,307	7,246	12,755	5	213.97	150.44	264.81	2
		1998	11,208	8,353	13,646	5	233.15	173.75	283.87	1
		1999	12,540	9,481	14,701	5	259.34	196.08	304.04	1
		2000	13,508	10,694	15,627	5	277.34	219.55	320.84	1
		2001	14,988	11,939	16,612	4	304.73	242.73	337.73	1
		2002	16,085	13,184	18,110	4	322.27	264.15	362.85	1
		2003	17,082	13,785	19,858	4	335.31	270.59	389.80	1
		2004	17,597	13,187	21,556	4	338.22	253.47	414.32	1
		2005	17,721	11,507	23,160	4	333.43	216.51	435.77	1
		2006	17,606	9,575	24,561	4	323.87	176.13	451.80	1
		2007	17,108	7,277	25,492	3	310.55	132.08	462.73	1
	Old (30-64)	1992	23,449	14,690	30,844	7	211.49	132.49	278.18	18
		1993	26,333	15,465	32,455	6	233.75	137.28	288.09	9
		1994	26,315	16,567	34,124	6	229.27	144.34	297.32	12
		1995	28,471	17,570	35,754	6	244.54	150.91	307.09	7
		1996	29,836	19,334	37,403	5	252.72	163.77	316.82	7
		1997	31,474	22,129	38,952	5	264.24	185.79	327.03	4
		1998	33,200	24,742	40,423	5	276.98	206.42	337.23	4
		1999	35,831	27,091	42,007	4	296.19	223.94	347.24	4
		2000	37,112	29,379	42,933	4	303.67	240.39	351.30	4
		2001	39,555	31,508	43,839	3	320.09	254.97	354.76	4
		2002	40,828	33,464	45,968	4	326.77	267.84	367.91	2
		2003	41,843	33,766	48,643	3	332.32	268.17	386.32	2
		2004	41,819	31,341	51,229	3	330.79	247.90	405.21	2
		2005	41,149	26,720	53,779	3	324.44	210.67	424.01	2
		2006	40,290	21,911	56,204	3	317.05	172.42	442.28	3
		2007	38,966	16,573	58,059	3	306.73	130.46	457.03	3

Metropolitan Statistical Area	PWID Population	Year	Number	Min	Max	Rank	Rate	Min	Max	Rank
Bergen--Passaic, NJ	Total	1992	8,576	6,873	10,099	53	98.44	78.89	115.93	55
		1993	8,255	6,733	9,590	47	94.65	77.21	109.97	53
		1994	8,051	6,613	9,086	55	92.19	75.72	104.04	58
		1995	7,756	6,513	8,625	49	88.46	74.29	98.37	56
		1996	7,573	6,449	8,268	51	85.85	73.11	93.73	59
		1997	7,436	6,404	8,307	53	83.60	72.00	93.40	61
		1998	7,301	6,176	8,547	53	81.55	68.99	95.47	59
		1999	7,259	5,733	8,977	58	80.77	63.79	99.88	64
		2000	7,000	5,271	9,327	56	77.47	58.33	103.23	64
		2001	6,901	5,026	9,288	61	75.94	55.31	102.21	66
		2002	6,630	5,057	8,779	60	72.72	55.46	96.28	66
		2003	6,418	5,369	7,983	63	70.25	58.77	87.38	68
		2004	6,380	5,441	7,329	65	69.70	59.44	80.07	68
		2005	6,378	5,152	7,205	68	69.58	56.20	78.59	70
		2006	6,393	4,879	8,196	68	69.59	53.11	89.22	69
		2007	6,432	4,612	9,239	66	69.79	50.05	100.24	69
	Non-Hispanic White	1992	5,083	4,074	5,986	47	81.44	65.27	95.91	47
		1993	4,533	3,698	5,267	49	73.67	60.09	85.59	54
		1994	4,160	3,416	4,694	57	68.48	56.25	77.28	54
		1995	3,835	3,220	4,264	54	63.78	53.57	70.93	56
		1996	3,645	3,104	3,979	59	61.19	52.11	66.81	58
		1997	3,540	3,048	3,954	58	59.83	51.53	66.84	60
		1998	3,486	2,949	4,081	61	59.38	50.23	69.52	61
		1999	3,516	2,777	4,348	64	60.53	47.80	74.85	62
		2000	3,471	2,613	4,625	58	60.29	45.40	80.33	62
		2001	3,524	2,566	4,742	64	61.83	45.03	83.21	62
		2002	3,498	2,668	4,631	62	62.08	47.35	82.19	63
		2003	3,502	2,930	4,356	63	62.91	52.64	78.26	64
		2004	3,596	3,066	4,131	64	65.35	55.74	75.08	62
		2005	3,702	2,991	4,182	63	68.06	54.98	76.88	57
		2006	3,804	2,904	4,878	61	70.75	54.00	90.71	56
		2007	3,902	2,798	5,604	62	73.23	52.51	105.18	54

Metropolitan Statistical Area	PWID Population	Year	Number	Min	Max	Rank	Rate	Min	Max	Rank
Bergen--Passaic, NJ	Non-Hispanic Black	1992	2,207	1,768	2,599	51	304.23	243.81	358.28	48
		1993	2,119	1,729	2,462	48	291.92	238.11	339.15	46
		1994	2,052	1,685	2,315	52	281.38	231.10	317.53	47
		1995	1,955	1,642	2,174	48	267.23	224.42	297.17	44
		1996	1,884	1,605	2,057	49	256.03	218.03	279.52	44
		1997	1,825	1,572	2,039	48	246.46	212.25	275.33	42
		1998	1,769	1,496	2,071	49	237.87	201.24	278.50	37
		1999	1,741	1,375	2,153	49	234.16	184.92	289.57	35
		2000	1,668	1,256	2,222	52	223.46	168.25	297.74	34
		2001	1,643	1,197	2,211	53	218.66	159.25	294.29	28
		2002	1,588	1,211	2,103	51	210.81	160.79	279.12	32
		2003	1,560	1,306	1,941	52	207.58	173.68	258.21	31
		2004	1,590	1,356	1,827	48	212.01	180.81	243.56	26
		2005	1,649	1,332	1,862	44	220.25	177.91	248.78	23
		2006	1,735	1,324	2,224	42	231.78	176.91	297.18	21
		2007	1,857	1,332	2,667	40	247.95	177.80	356.13	20
	Hispanic	1992	1,507	1,208	1,775	37	126.13	101.08	148.54	52
		1993	1,522	1,241	1,768	36	121.54	99.13	141.20	47
		1994	1,548	1,272	1,747	37	118.62	97.42	133.86	47
		1995	1,548	1,300	1,721	37	113.59	95.39	126.31	46
		1996	1,562	1,330	1,705	37	109.38	93.14	119.41	45
		1997	1,578	1,359	1,763	38	105.53	90.89	117.90	41
		1998	1,590	1,345	1,862	38	102.05	86.34	119.48	42
		1999	1,619	1,279	2,003	38	100.41	79.30	124.17	42
		2000	1,597	1,203	2,128	38	95.67	72.03	127.47	46
		2001	1,609	1,172	2,166	38	92.75	67.55	124.83	43
		2002	1,581	1,206	2,094	38	88.21	67.28	116.80	46
		2003	1,567	1,311	1,949	38	84.74	70.90	105.41	45
		2004	1,599	1,363	1,836	38	84.09	71.71	96.60	45
		2005	1,646	1,329	1,859	38	84.32	68.11	95.24	45
		2006	1,706	1,302	2,187	38	85.24	65.06	109.29	44
		2007	1,785	1,280	2,564	36	86.92	62.33	124.85	44

Metropolitan Statistical Area	PWID Population	Year	Number	Min	Max	Rank	Rate	Min	Max	Rank
Bergen--Passaic, NJ	Male	1992	5,672	4,545	6,679	53	132.85	106.47	156.46	55
		1993	5,423	4,423	6,300	47	126.87	103.49	147.40	54
		1994	5,271	4,329	5,949	54	123.10	101.11	138.92	55
		1995	5,076	4,262	5,644	48	118.01	99.10	131.23	56
		1996	4,966	4,229	5,421	51	114.75	97.72	125.28	56
		1997	4,894	4,215	5,468	49	112.17	96.60	125.31	56
		1998	4,831	4,087	5,656	53	109.97	93.03	128.75	56
		1999	4,832	3,816	5,976	55	109.51	86.48	135.42	61
		2000	4,690	3,531	6,249	53	105.72	79.60	140.86	58
		2001	4,652	3,388	6,261	57	104.13	75.84	140.15	61
		2002	4,495	3,428	5,951	55	100.18	76.41	132.64	61
		2003	4,370	3,657	5,436	56	97.12	81.26	120.80	63
		2004	4,358	3,716	5,006	58	96.54	82.33	110.90	61
		2005	4,362	3,523	4,927	58	96.36	77.84	108.85	61
		2006	4,366	3,332	5,597	58	96.17	73.40	123.31	63
		2007	4,374	3,137	6,283	58	95.90	68.77	137.74	62
	Female	1992	2,909	2,331	3,426	51	65.48	52.48	77.12	56
		1993	2,841	2,317	3,300	47	63.88	52.10	74.21	54
		1994	2,796	2,296	3,155	55	62.82	51.59	70.89	61
		1995	2,706	2,273	3,010	53	60.59	50.89	67.38	59
		1996	2,645	2,252	2,888	53	58.86	50.12	64.26	60
		1997	2,591	2,231	2,894	55	57.18	49.24	63.88	61
		1998	2,531	2,141	2,963	58	55.50	46.95	64.98	67
		1999	2,498	1,973	3,089	61	54.60	43.12	67.52	68
		2000	2,387	1,797	3,180	61	51.90	39.07	69.15	68
		2001	2,329	1,696	3,135	67	50.42	36.72	67.86	75
		2002	2,214	1,689	2,931	66	47.81	36.46	63.30	75
		2003	2,120	1,774	2,637	68	45.73	38.26	56.89	78
		2004	2,086	1,779	2,396	73	44.96	38.34	51.65	81
		2005	2,067	1,669	2,334	74	44.52	35.97	50.29	81
		2006	2,056	1,570	2,637	76	44.25	33.78	56.74	80
		2007	2,059	1,477	2,958	76	44.24	31.72	63.54	80

Metropolitan Statistical Area	PWID Population	Year	Number	Min	Max	Rank	Rate	Min	Max	Rank
Bergen--Passaic, NJ	Young (15-29)	1992	2,486	1,992	2,927	45	93.59	75.01	110.22	47
		1993	2,344	1,912	2,724	43	90.58	73.89	105.24	46
		1994	2,269	1,864	2,561	46	89.70	73.67	101.23	45
		1995	2,193	1,841	2,438	46	88.15	74.03	98.03	49
		1996	2,166	1,844	2,364	47	88.07	75.00	96.15	51
		1997	2,164	1,864	2,418	52	88.53	76.24	98.90	48
		1998	2,172	1,837	2,543	51	89.03	75.32	104.24	47
		1999	2,211	1,746	2,734	55	91.03	71.89	112.57	46
		2000	2,182	1,643	2,908	52	89.69	67.53	119.51	51
		2001	2,197	1,600	2,957	59	90.92	66.22	122.37	52
		2002	2,146	1,637	2,842	60	89.24	68.07	118.16	53
		2003	2,100	1,757	2,612	65	87.33	73.06	108.63	58
		2004	2,092	1,784	2,403	66	86.76	73.99	99.67	58
		2005	2,076	1,677	2,344	71	85.33	68.93	96.39	59
		2006	2,039	1,557	2,615	72	83.31	63.58	106.81	63
		2007	1,983	1,422	2,848	72	80.03	57.39	114.95	63
	Old (30-64)	1992	6,182	4,954	7,280	56	102.07	81.80	120.21	63
		1993	5,954	4,857	6,917	51	97.08	79.19	112.79	61
		1994	5,805	4,768	6,551	58	93.58	76.86	105.60	67
		1995	5,585	4,690	6,211	54	88.93	74.68	98.89	67
		1996	5,441	4,634	5,941	55	85.53	72.83	93.38	68
		1997	5,325	4,586	5,949	56	82.57	71.11	92.25	67
		1998	5,206	4,404	6,095	59	79.93	67.62	93.58	66
		1999	5,148	4,066	6,367	58	78.50	61.99	97.07	71
		2000	4,933	3,714	6,572	59	74.71	56.25	99.54	63
		2001	4,826	3,515	6,496	61	72.35	52.69	97.37	72
		2002	4,598	3,507	6,089	60	68.50	52.25	90.70	66
		2003	4,411	3,691	5,487	61	65.53	54.83	81.52	67
		2004	4,344	3,705	4,991	61	64.43	54.95	74.02	67
		2005	4,301	3,474	4,858	60	63.85	51.58	72.13	68
		2006	4,266	3,256	5,469	57	63.30	48.31	81.16	63
		2007	4,243	3,043	6,094	57	62.96	45.15	90.43	64

Metropolitan Statistical Area	PWID Population	Year	Number	Min	Max	Rank	Rate	Min	Max	Rank
Birmingham, AL	Total	1992	3,138	2,370	3,723	90	55.36	41.82	65.69	93
		1993	2,739	1,410	3,670	88	47.73	24.58	63.97	94
		1994	3,226	2,531	3,617	88	55.53	43.58	62.26	92
		1995	2,779	1,329	3,625	89	47.39	22.67	61.83	94
		1996	2,800	1,292	3,726	89	47.25	21.81	62.89	94
		1997	2,814	1,257	3,817	89	47.14	21.05	63.94	94
		1998	2,851	1,235	3,945	89	47.18	20.44	65.27	95
		1999	3,433	2,932	4,057	86	56.35	48.12	66.59	86
		2000	2,953	1,371	4,202	89	48.09	22.33	68.45	94
		2001	3,526	3,048	4,348	86	57.02	49.29	70.31	85
		2002	3,104	1,696	4,507	89	49.87	27.24	72.40	92
		2003	3,225	1,980	4,689	89	51.23	31.46	74.50	92
		2004	3,340	2,261	4,869	89	52.56	35.58	76.62	91
		2005	3,459	2,545	5,054	89	53.89	39.65	78.74	90
		2006	3,589	2,837	5,264	89	55.19	43.63	80.95	88
		2007	3,699	2,861	5,449	89	56.46	43.67	83.17	85
	Non-Hispanic White	1992	1,998	1,509	2,370	84	49.84	37.65	59.13	76
		1993	1,745	899	2,339	84	43.27	22.28	57.99	79
		1994	2,082	1,633	2,334	81	51.33	40.28	57.55	75
		1995	1,829	875	2,386	84	44.99	21.52	58.70	76
		1996	1,886	870	2,510	83	46.19	21.32	61.47	72
		1997	1,938	865	2,628	83	47.43	21.18	64.34	71
		1998	2,000	866	2,766	81	48.65	21.08	67.30	72
		1999	2,437	2,081	2,880	78	59.22	50.57	69.98	63
		2000	2,103	977	2,993	83	51.05	23.71	72.66	71
		2001	2,491	2,153	3,072	79	60.29	52.11	74.33	64
		2002	2,143	1,171	3,111	84	51.77	28.28	75.16	72
		2003	2,131	1,309	3,099	84	51.18	31.43	74.42	71
		2004	2,053	1,390	2,993	85	49.09	33.24	71.56	74
		2005	1,896	1,395	2,770	91	45.16	33.23	65.99	84
		2006	1,650	1,304	2,420	94	39.03	30.85	57.25	89
		2007	1,305	1,009	1,922	97	30.77	23.80	45.32	98

Metropolitan Statistical Area	PWID Population	Year	Number	Min	Max	Rank	Rate	Min	Max	Rank
Birmingham, AL	Non-Hispanic Black	1992	1,145	865	1,358	72	72.47	54.74	85.98	100
		1993	939	483	1,258	78	58.13	29.94	77.92	100
		1994	1,033	811	1,159	72	62.54	49.08	70.12	100
		1995	831	397	1,084	78	49.32	23.59	64.35	100
		1996	783	361	1,042	78	45.66	21.08	60.77	100
		1997	741	331	1,006	77	42.58	19.01	57.76	100
		1998	716	310	991	77	40.40	17.51	55.90	100
		1999	836	714	988	68	46.55	39.75	55.01	100
		2000	713	331	1,014	74	39.18	18.19	55.76	100
		2001	867	749	1,069	66	47.11	40.72	58.08	100
		2002	801	437	1,162	69	43.06	23.52	62.51	99
		2003	902	554	1,311	65	47.68	29.28	69.33	97
		2004	1,046	708	1,525	63	54.50	36.90	79.45	93
		2005	1,250	920	1,827	61	64.04	47.12	93.57	91
		2006	1,530	1,210	2,245	52	76.83	60.74	112.70	82
		2007	1,878	1,453	2,767	39	93.24	72.12	137.35	78
	Hispanic	1992	6	4	7	101	17.42	13.16	20.67	101
		1993	5	3	7	101	13.44	6.92	18.01	101
		1994	6	5	7	101	13.62	10.69	15.27	101
		1995	5	3	7	101	10.16	4.86	13.25	101
		1996	5	3	7	101	8.61	3.97	11.46	101
		1997	5	2	7	101	7.40	3.31	10.04	101
		1998	6	2	8	101	6.33	2.74	8.75	101
		1999	7	6	8	101	6.49	5.54	7.67	101
		2000	6	3	8	101	5.01	2.33	7.13	101
		2001	7	6	9	101	5.72	4.94	7.05	101
		2002	7	4	10	101	4.83	2.64	7.02	101
		2003	7	4	11	101	4.92	3.02	7.16	101
		2004	8	5	12	101	5.11	3.46	7.45	101
		2005	9	7	13	101	5.38	3.96	7.86	101
		2006	10	8	15	101	5.83	4.61	8.55	101
		2007	12	9	18	100	6.54	5.06	9.63	101

Metropolitan Statistical Area	PWID Population	Year	Number	Min	Max	Rank	Rate	Min	Max	Rank
Birmingham, AL	Male	1992	2,085	1,575	2,474	89	76.63	57.89	90.92	92
		1993	1,803	928	2,417	89	65.37	33.66	87.61	95
		1994	2,088	1,639	2,342	88	74.72	58.63	83.77	91
		1995	1,758	841	2,294	89	62.23	29.76	81.19	95
		1996	1,722	795	2,292	89	60.28	27.82	80.23	95
		1997	1,676	748	2,273	89	58.21	25.99	78.95	95
		1998	1,641	711	2,270	89	56.23	24.36	77.79	96
		1999	1,908	1,629	2,254	88	64.78	55.32	76.55	91
		2000	1,587	737	2,259	89	53.45	24.82	76.07	96
		2001	1,843	1,593	2,272	88	61.56	53.21	75.90	95
		2002	1,589	868	2,306	91	52.62	28.74	76.39	96
		2003	1,633	1,003	2,374	91	53.36	32.77	77.59	96
		2004	1,695	1,148	2,471	91	54.89	37.16	80.01	96
		2005	1,786	1,314	2,610	91	57.22	42.10	83.61	96
		2006	1,917	1,515	2,812	89	60.60	47.91	88.90	95
		2007	2,078	1,607	3,061	89	65.18	50.42	96.02	91
	Female	1992	1,035	782	1,228	87	35.12	26.53	41.67	91
		1993	925	476	1,239	88	31.03	15.98	41.59	93
		1994	1,131	887	1,268	87	37.53	29.45	42.08	86
		1995	1,022	489	1,333	87	33.63	16.08	43.88	89
		1996	1,086	501	1,446	87	35.39	16.34	47.11	89
		1997	1,154	515	1,565	87	37.34	16.67	50.65	88
		1998	1,234	534	1,707	87	39.46	17.10	54.59	87
		1999	1,560	1,332	1,843	85	49.55	42.31	58.55	78
		2000	1,399	649	1,990	86	44.13	20.49	62.80	78
		2001	1,725	1,491	2,127	82	54.06	46.73	66.66	68
		2002	1,551	847	2,251	84	48.37	26.42	70.23	73
		2003	1,623	996	2,359	83	50.17	30.81	72.95	73
		2004	1,667	1,128	2,429	82	51.01	34.54	74.36	71
		2005	1,678	1,235	2,452	83	50.91	37.46	74.38	68
		2006	1,654	1,308	2,427	83	49.54	39.16	72.67	72
		2007	1,572	1,216	2,316	84	46.75	36.16	68.87	77

Metropolitan Statistical Area	PWID Population	Year	Number	Min	Max	Rank	Rate	Min	Max	Rank
Birmingham, AL	Young (15-29)	1992	942	712	1,118	83	50.23	37.94	59.60	78
		1993	794	409	1,064	85	42.23	21.74	56.59	80
		1994	941	738	1,055	82	49.90	39.16	55.94	78
		1995	844	403	1,101	83	44.58	21.32	58.16	82
		1996	906	418	1,206	82	47.60	21.97	63.35	75
		1997	984	439	1,334	83	51.52	23.00	69.87	71
		1998	1,082	469	1,497	84	56.50	24.48	78.17	67
		1999	1,409	1,204	1,665	78	73.77	63.00	87.17	63
		2000	1,297	602	1,846	81	68.28	31.71	97.18	66
		2001	1,632	1,410	2,012	78	86.79	75.02	107.01	59
		2002	1,482	809	2,151	78	79.44	43.40	115.33	64
		2003	1,548	951	2,251	79	82.16	50.46	119.47	62
		2004	1,562	1,057	2,277	79	82.37	55.76	120.07	62
		2005	1,513	1,113	2,211	82	79.23	58.30	115.77	67
		2006	1,392	1,101	2,042	86	71.95	56.88	105.55	74
		2007	1,183	915	1,743	88	60.90	47.11	89.71	87
	Old (30-64)	1992	2,240	1,693	2,658	91	59.10	44.65	70.12	94
		1993	1,965	1,012	2,634	92	50.94	26.23	68.27	94
		1994	2,299	1,804	2,577	91	58.60	45.99	65.71	92
		1995	1,947	931	2,541	91	49.04	23.46	63.98	94
		1996	1,913	883	2,546	91	47.56	21.95	63.30	95
		1997	1,860	831	2,523	91	45.82	20.46	62.15	95
		1998	1,811	785	2,506	91	43.88	19.01	60.70	96
		1999	2,087	1,782	2,466	88	49.91	42.62	58.97	94
		2000	1,715	797	2,441	93	40.46	18.79	57.58	96
		2001	1,963	1,696	2,420	89	45.59	39.41	56.22	94
		2002	1,667	911	2,420	93	38.24	20.89	55.51	96
		2003	1,691	1,039	2,459	92	38.34	23.55	55.75	96
		2004	1,740	1,178	2,536	92	39.02	26.41	56.88	96
		2005	1,827	1,344	2,669	91	40.52	29.82	59.21	93
		2006	1,968	1,556	2,887	88	43.09	34.07	63.21	91
		2007	2,156	1,668	3,176	87	46.78	36.19	68.92	84

Metropolitan Statistical Area	PWID Population	Year	Number	Min	Max	Rank	Rate	Min	Max	Rank
Boston, MA--NH	Total	1992	25,960	24,404	28,135	10	67.99	63.91	73.68	82
		1993	39,976	26,731	73,030	5	104.61	69.95	191.11	49
		1994	32,072	29,292	34,665	7	83.55	76.31	90.31	67
		1995	46,991	32,024	81,754	4	121.31	82.67	211.04	39
		1996	50,239	34,840	85,951	3	128.63	89.20	220.06	36
		1997	53,166	37,811	89,998	3	134.60	95.73	227.86	31
		1998	55,841	40,878	94,005	3	139.98	102.47	235.65	28
		1999	45,208	44,067	46,499	5	112.04	109.22	115.24	42
		2000	61,357	44,208	102,495	3	150.43	108.39	251.29	19
		2001	50,522	44,093	54,021	5	122.29	106.72	130.76	35
		2002	66,127	45,676	102,646	3	158.75	109.65	246.42	13
		2003	68,653	47,567	102,157	3	163.96	113.61	243.98	11
		2004	70,763	48,197	101,084	3	168.30	114.63	240.41	9
		2005	72,472	47,109	99,649	3	171.48	111.47	235.78	8
		2006	74,336	45,953	98,329	2	174.75	108.03	231.16	8
		2007	76,040	44,235	96,701	2	177.52	103.27	225.75	8
	Non-Hispanic White	1992	16,743	15,740	18,146	6	49.86	46.87	54.04	75
		1993	25,741	17,212	47,025	3	77.00	51.48	140.66	49
		1994	20,515	18,736	22,173	5	61.39	56.07	66.35	61
		1995	29,740	20,268	51,741	2	88.70	60.45	154.31	43
		1996	31,364	21,750	53,659	1	93.29	64.70	159.61	39
		1997	32,671	23,236	55,305	1	96.68	68.76	163.67	34
		1998	33,740	24,699	56,799	1	99.43	72.79	167.39	34
		1999	26,856	26,178	27,623	2	78.75	76.77	81.00	51
		2000	35,878	25,850	59,932	1	104.70	75.44	174.90	29
		2001	29,148	25,439	31,167	2	84.49	73.74	90.35	42
		2002	37,784	26,099	58,650	1	109.21	75.44	169.53	26
		2003	39,046	27,054	58,101	1	112.83	78.18	167.89	24
		2004	40,311	27,456	57,584	1	116.53	79.37	166.45	22
		2005	41,652	27,075	57,271	1	120.33	78.22	165.45	19
		2006	43,443	26,856	57,466	1	125.27	77.44	165.70	18
		2007	45,553	26,500	57,931	1	131.02	76.22	166.62	16

Metropolitan Statistical Area	PWID Population	Year	Number	Min	Max	Rank	Rate	Min	Max	Rank
Boston, MA--NH	Non-Hispanic Black	1992	4,376	4,114	4,742	24	255.83	240.50	277.26	59
		1993	6,369	4,259	11,635	15	365.35	244.29	667.43	33
		1994	4,836	4,417	5,227	23	271.12	247.61	293.04	48
		1995	6,717	4,578	11,686	13	365.91	249.36	636.59	21
		1996	6,822	4,731	11,671	13	362.08	251.10	619.47	19
		1997	6,874	4,889	11,637	13	351.31	249.85	594.69	16
		1998	6,897	5,049	11,610	14	342.27	250.56	576.20	14
		1999	5,353	5,218	5,506	17	256.88	250.40	264.22	24
		2000	6,994	5,039	11,683	12	327.07	235.65	546.36	11
		2001	5,570	4,861	5,956	16	253.44	221.19	271.00	21
		2002	7,088	4,896	11,003	12	314.81	217.45	488.66	12
		2003	7,197	4,986	10,709	12	313.26	217.05	466.14	12
		2004	7,303	4,974	10,432	12	312.86	213.09	446.92	13
		2005	7,422	4,824	10,205	12	312.43	203.09	429.59	13
		2006	7,627	4,715	10,089	13	314.50	194.42	416.01	14
		2007	7,911	4,602	10,060	13	320.29	186.33	407.32	14
	Hispanic	1992	3,606	3,390	3,908	22	211.47	198.79	229.18	29
		1993	5,455	3,648	9,966	13	307.18	205.40	561.16	14
		1994	4,372	3,993	4,725	16	236.60	216.09	255.73	22
		1995	6,485	4,419	11,282	11	334.66	228.07	582.22	12
		1996	7,087	4,915	12,125	9	349.42	242.32	597.81	11
		1997	7,716	5,487	13,061	7	363.62	258.61	615.53	8
		1998	8,362	6,121	14,077	5	375.66	275.00	632.41	8
		1999	6,984	6,808	7,183	8	299.52	291.96	308.07	12
		2000	9,746	7,022	16,281	4	400.45	288.53	668.94	9
		2001	8,201	7,157	8,769	4	320.28	279.52	342.46	11
		2002	10,872	7,509	16,875	4	408.03	281.84	633.36	8
		2003	11,298	7,828	16,812	4	409.63	283.82	609.55	7
		2004	11,491	7,826	16,414	4	404.14	275.26	577.31	8
		2005	11,414	7,420	15,695	4	389.16	252.96	535.09	8
		2006	11,132	6,882	14,725	4	366.85	226.78	485.26	8
		2007	10,584	6,157	13,460	4	336.67	195.85	428.15	9

Metropolitan Statistical Area	PWID Population	Year	Number	Min	Max	Rank	Rate	Min	Max	Rank
Boston, MA--NH	Male	1992	16,844	15,834	18,255	11	89.47	84.11	96.96	85
		1993	25,989	17,378	47,478	5	137.93	92.23	251.98	48
		1994	20,940	19,125	22,633	7	110.68	101.09	119.63	66
		1995	30,868	21,037	53,703	4	161.72	110.21	281.35	38
		1996	33,243	23,053	56,874	4	172.80	119.83	295.63	34
		1997	35,461	25,220	60,027	3	182.37	129.70	308.72	31
		1998	37,548	27,487	63,211	3	191.28	140.03	322.01	26
		1999	30,636	29,863	31,510	4	154.31	150.42	158.72	37
		2000	41,870	30,167	69,942	3	208.67	150.34	348.57	15
		2001	34,674	30,261	37,076	4	170.45	148.76	182.25	32
		2002	45,569	31,476	70,734	3	221.86	153.25	344.38	11
		2003	47,404	32,845	70,538	3	229.40	158.94	341.35	11
		2004	48,836	33,262	69,761	3	235.27	160.24	336.08	9
		2005	49,841	32,398	68,531	3	238.68	155.15	328.18	9
		2006	50,766	31,382	67,151	3	241.27	149.15	319.14	9
		2007	51,352	29,873	65,305	2	242.16	140.87	307.96	9
	Female	1992	8,898	8,365	9,644	15	45.97	43.21	49.82	81
		1993	13,800	9,228	25,211	4	71.24	47.63	130.14	46
		1994	11,092	10,130	11,989	7	56.98	52.04	61.59	66
		1995	16,209	11,046	28,200	3	82.49	56.21	143.51	38
		1996	17,220	11,942	29,461	3	86.88	60.25	148.64	36
		1997	18,057	12,842	30,566	3	90.04	64.04	152.42	31
		1998	18,752	13,727	31,568	3	92.55	67.75	155.80	29
		1999	14,991	14,613	15,419	5	73.14	71.30	75.23	49
		2000	20,080	14,468	33,543	3	96.91	69.82	161.88	21
		2001	16,323	14,246	17,453	5	77.83	67.93	83.22	45
		2002	21,115	14,585	32,776	4	100.00	69.07	155.22	21
		2003	21,709	15,041	32,303	4	102.37	70.93	152.33	20
		2004	22,223	15,136	31,745	4	104.38	71.09	149.11	20
		2005	22,689	14,749	31,198	3	106.12	68.98	145.92	19
		2006	23,312	14,411	30,836	2	108.44	67.04	143.45	18
		2007	24,023	13,975	30,550	2	111.06	64.61	141.24	16

Metropolitan Statistical Area	PWID Population	Year	Number	Min	Max	Rank	Rate	Min	Max	Rank
Boston, MA--NH	Young (15-29)	1992	8,096	7,611	8,774	9	61.70	58.00	66.87	72
		1993	12,900	8,626	23,566	3	100.91	67.47	184.34	45
		1994	10,658	9,734	11,520	5	85.26	77.87	92.16	51
		1995	16,021	10,919	27,874	3	130.02	88.61	226.21	25
		1996	17,525	12,153	29,982	2	143.88	99.78	246.16	15
		1997	18,939	13,469	32,059	1	156.32	111.17	264.61	12
		1998	20,293	14,855	34,162	1	168.27	123.18	283.28	9
		1999	16,756	16,334	17,235	3	139.76	136.23	143.75	20
		2000	23,210	16,723	38,771	1	194.20	139.92	324.40	6
		2001	19,530	17,045	20,883	3	162.83	142.11	174.11	16
		2002	26,177	18,081	40,633	1	217.65	150.34	337.84	6
		2003	27,904	19,334	41,521	1	230.68	159.83	343.25	6
		2004	29,627	20,179	42,321	1	243.44	165.80	347.74	5
		2005	31,369	20,391	43,132	1	255.46	166.06	351.26	5
		2006	33,395	20,644	44,174	1	268.76	166.15	355.51	4
		2007	35,597	20,708	45,270	1	283.70	165.04	360.79	3
	Old (30-64)	1992	18,382	17,280	19,922	14	73.35	68.95	79.49	88
		1993	27,542	18,416	50,315	5	108.30	72.42	197.86	51
		1994	21,649	19,772	23,400	8	83.63	76.38	90.40	78
		1995	31,271	21,311	54,404	4	118.38	80.68	205.95	43
		1996	33,121	22,969	56,665	4	123.23	85.45	210.82	41
		1997	34,835	24,775	58,969	4	127.22	90.48	215.36	39
		1998	36,415	26,657	61,302	3	130.84	95.78	220.26	36
		1999	29,327	28,587	30,165	6	103.41	100.80	106.37	53
		2000	39,502	28,461	65,986	3	136.99	98.70	228.84	28
		2001	32,140	28,050	34,366	5	109.62	95.67	117.21	43
		2002	41,300	28,528	64,108	3	139.40	96.29	216.38	24
		2003	41,718	28,905	62,077	4	140.11	97.08	208.49	20
		2004	41,327	28,148	59,035	4	138.33	94.21	197.60	19
		2005	40,006	26,005	55,008	4	133.43	86.73	183.46	18
		2006	37,923	23,443	50,163	4	125.94	77.85	166.59	19
		2007	34,799	20,244	44,255	4	114.89	66.84	146.11	22

Metropolitan Statistical Area	PWID Population	Year	Number	Min	Max	Rank	Rate	Min	Max	Rank
Buffalo--Niagara Falls, NY	Total	1992	4,989	3,746	6,046	77	64.96	48.78	78.73	86
		1993	5,974	4,073	8,084	67	77.96	53.16	105.51	69
		1994	5,530	4,369	6,208	74	72.46	57.24	81.33	79
		1995	6,385	4,620	8,226	66	84.05	60.82	108.28	63
		1996	6,584	4,852	8,318	64	86.94	64.07	109.84	58
		1997	6,696	4,775	8,391	64	88.82	63.34	111.30	56
		1998	6,732	4,382	8,482	64	89.50	58.26	112.77	56
		1999	6,091	3,773	7,890	68	81.21	50.31	105.20	61
		2000	6,656	3,268	8,667	61	88.90	43.65	115.77	55
		2001	5,972	2,999	8,096	70	79.68	40.01	108.02	63
		2002	6,608	3,126	8,231	61	87.97	41.62	109.59	55
		2003	6,730	3,646	8,166	58	89.31	48.38	108.35	53
		2004	6,802	4,023	8,166	55	90.05	53.26	108.12	54
		2005	6,827	4,319	8,149	55	90.39	57.18	107.88	53
		2006	6,844	4,626	8,089	59	90.64	61.27	107.13	52
		2007	6,841	4,896	8,036	60	90.55	64.81	106.38	52
	Non-Hispanic White	1992	1,524	1,144	1,847	94	23.19	17.41	28.10	99
		1993	1,911	1,303	2,586	82	29.25	19.94	39.59	95
		1994	1,841	1,454	2,066	85	28.40	22.44	31.88	98
		1995	2,200	1,592	2,835	76	34.22	24.76	44.09	86
		1996	2,340	1,724	2,956	76	36.65	27.01	46.30	84
		1997	2,448	1,746	3,068	75	38.63	27.55	48.41	81
		1998	2,527	1,645	3,184	76	40.10	26.10	50.53	81
		1999	2,347	1,454	3,040	79	37.46	23.20	48.52	88
		2000	2,633	1,293	3,429	77	42.22	20.73	54.98	77
		2001	2,428	1,219	3,292	82	39.00	19.58	52.87	87
		2002	2,766	1,309	3,445	74	44.42	21.02	55.34	78
		2003	2,907	1,575	3,527	73	46.64	25.27	56.59	77
		2004	3,039	1,798	3,649	73	48.74	28.83	58.52	75
		2005	3,165	2,003	3,778	73	50.84	32.16	60.67	74
		2006	3,302	2,232	3,902	73	53.16	35.93	62.82	75
		2007	3,443	2,464	4,045	71	55.53	39.74	65.23	73

Metropolitan Statistical Area	PWID Population	Year	Number	Min	Max	Rank	Rate	Min	Max	Rank
Buffalo--Niagara Falls, NY	Non-Hispanic Black	1992	2,570	1,930	3,115	45	320.68	240.77	388.64	46
		1993	2,976	2,029	4,027	33	366.77	250.07	496.34	32
		1994	2,635	2,082	2,957	37	320.18	252.95	359.38	39
		1995	2,879	2,083	3,709	31	347.11	251.18	447.20	25
		1996	2,783	2,051	3,516	31	331.82	244.54	419.22	22
		1997	2,631	1,876	3,297	32	311.84	222.39	390.78	21
		1998	2,441	1,589	3,076	33	287.12	186.90	361.78	22
		1999	2,029	1,257	2,628	41	237.25	146.97	307.33	34
		2000	2,032	998	2,647	38	236.07	115.91	307.43	31
		2001	1,674	841	2,270	51	192.97	96.90	261.61	43
		2002	1,709	809	2,129	45	195.76	92.62	243.87	36
		2003	1,622	878	1,967	47	184.11	99.74	223.38	37
		2004	1,547	915	1,858	51	174.92	103.45	210.01	40
		2005	1,493	945	1,782	49	168.07	106.32	200.59	41
		2006	1,472	995	1,740	54	164.97	111.51	194.98	46
		2007	1,487	1,064	1,747	55	165.70	118.59	194.65	46
	Hispanic	1992	692	519	838	47	423.49	317.96	513.24	8
		1993	865	590	1,171	41	509.20	347.18	689.10	5
		1994	838	662	941	43	473.56	374.13	531.54	5
		1995	1,012	733	1,304	40	554.30	401.10	714.13	5
		1996	1,089	803	1,376	40	573.62	422.73	724.70	5
		1997	1,152	822	1,444	40	591.57	421.87	741.33	5
		1998	1,198	780	1,510	40	598.66	389.69	754.32	4
		1999	1,115	691	1,444	43	542.77	336.23	703.07	5
		2000	1,243	610	1,619	42	589.97	289.67	768.31	4
		2001	1,128	567	1,530	43	521.82	262.02	707.41	5
		2002	1,251	592	1,558	43	564.44	267.06	703.15	4
		2003	1,262	684	1,531	43	555.19	300.77	673.61	4
		2004	1,249	739	1,499	44	538.63	318.57	646.69	5
		2005	1,211	766	1,445	45	513.70	324.98	613.11	4
		2006	1,156	782	1,367	47	480.46	324.76	567.85	5
		2007	1,084	776	1,274	47	438.04	313.50	514.57	4

Metropolitan Statistical Area	PWID Population	Year	Number	Min	Max	Rank	Rate	Min	Max	Rank
Buffalo--Niagara Falls, NY	Male	1992	3,482	2,614	4,219	77	93.26	70.02	113.02	82
		1993	4,148	2,828	5,614	64	111.26	75.86	150.57	63
		1994	3,827	3,024	4,296	72	102.90	81.29	115.49	74
		1995	4,410	3,191	5,681	60	119.03	86.13	153.35	55
		1996	4,543	3,348	5,740	57	122.84	90.52	155.19	48
		1997	4,622	3,296	5,792	57	125.45	89.47	157.21	48
		1998	4,651	3,027	5,860	55	126.45	82.31	159.33	48
		1999	4,215	2,611	5,460	63	114.88	71.17	148.82	59
		2000	4,615	2,266	6,010	54	125.98	61.86	164.06	46
		2001	4,151	2,084	5,627	65	113.05	56.77	153.26	57
		2002	4,604	2,178	5,735	52	124.97	59.13	155.69	43
		2003	4,700	2,546	5,703	51	126.95	68.77	154.02	42
		2004	4,760	2,815	5,715	51	128.11	75.77	153.81	41
		2005	4,786	3,028	5,712	51	128.60	81.35	153.48	41
		2006	4,802	3,246	5,676	53	129.01	87.20	152.47	39
		2007	4,801	3,436	5,640	53	128.77	92.16	151.27	38
	Female	1992	1,468	1,102	1,779	79	37.19	27.93	45.08	88
		1993	1,805	1,231	2,443	72	45.89	31.29	62.10	76
		1994	1,706	1,348	1,915	77	43.60	34.45	48.94	84
		1995	2,001	1,448	2,577	69	51.40	37.19	66.22	69
		1996	2,085	1,537	2,634	68	53.82	39.66	67.99	65
		1997	2,135	1,522	2,675	66	55.37	39.49	69.39	65
		1998	2,152	1,401	2,712	67	55.99	36.45	70.55	65
		1999	1,946	1,206	2,521	75	50.81	31.47	65.81	72
		2000	2,120	1,041	2,761	71	55.45	27.22	72.21	65
		2001	1,892	950	2,564	77	49.47	24.84	67.07	78
		2002	2,077	983	2,587	73	54.27	25.68	67.61	66
		2003	2,096	1,136	2,543	72	54.68	29.62	66.34	64
		2004	2,097	1,240	2,518	71	54.64	32.32	65.61	65
		2005	2,082	1,317	2,485	69	54.34	34.38	64.86	65
		2006	2,065	1,396	2,440	75	53.94	36.46	63.74	66
		2007	2,042	1,462	2,399	77	53.38	38.21	62.71	65

Metropolitan Statistical Area	PWID Population	Year	Number	Min	Max	Rank	Rate	Min	Max	Rank
Buffalo--Niagara Falls, NY	Young (15-29)	1992	765	575	928	89	30.67	23.02	37.16	98
		1993	891	608	1,206	81	36.54	24.91	49.45	87
		1994	824	651	925	86	34.64	27.36	38.88	95
		1995	973	704	1,254	81	41.86	30.29	53.93	83
		1996	1,049	773	1,326	81	45.99	33.89	58.11	81
		1997	1,136	810	1,424	79	50.72	36.17	63.56	72
		1998	1,235	804	1,556	77	55.79	36.32	70.30	69
		1999	1,222	757	1,583	84	55.90	34.63	72.41	79
		2000	1,473	723	1,918	78	68.04	33.41	88.61	67
		2001	1,466	736	1,987	81	67.41	33.85	91.38	71
		2002	1,802	853	2,245	71	81.92	38.76	102.06	63
		2003	2,038	1,104	2,473	66	91.01	49.30	110.42	56
		2004	2,278	1,347	2,735	63	100.01	59.15	120.08	52
		2005	2,512	1,589	2,999	58	108.56	68.68	129.57	43
		2006	2,745	1,855	3,244	53	116.67	78.86	137.89	35
		2007	2,960	2,118	3,477	52	123.81	88.61	145.44	35
	Old (30-64)	1992	4,254	3,194	5,155	69	82.06	61.61	99.45	80
		1993	5,109	3,483	6,914	61	97.80	66.68	132.35	60
		1994	4,730	3,737	5,309	66	90.02	71.12	101.04	72
		1995	5,444	3,940	7,014	55	103.28	74.74	133.06	52
		1996	5,581	4,113	7,051	53	105.46	77.72	133.24	51
		1997	5,623	4,010	7,047	52	106.12	75.68	132.99	49
		1998	5,579	3,632	7,030	51	105.10	68.41	132.43	47
		1999	4,959	3,072	6,424	61	93.33	57.82	120.89	59
		2000	5,295	2,600	6,896	55	99.50	48.85	129.58	49
		2001	4,614	2,317	6,255	64	86.72	43.54	117.56	58
		2002	4,920	2,328	6,129	56	92.64	43.83	115.40	51
		2003	4,787	2,593	5,808	57	90.38	48.96	109.66	49
		2004	4,574	2,705	5,492	57	86.70	51.28	104.09	51
		2005	4,287	2,712	5,117	61	81.82	51.76	97.66	53
		2006	3,954	2,673	4,673	64	76.07	51.42	89.90	54
		2007	3,576	2,559	4,201	72	69.26	49.57	81.36	58

Metropolitan Statistical Area	PWID Population	Year	Number	Min	Max	Rank	Rate	Min	Max	Rank
Charleston--North Charleston, SC	Total	1992	2,513	1,914	3,008	95	69.94	53.28	83.72	81
		1993	2,028	964	2,852	96	56.46	26.85	79.41	85
		1994	2,246	1,633	2,696	96	62.99	45.80	75.59	84
		1995	1,837	1,037	2,542	98	52.26	29.49	72.29	89
		1996	1,758	1,065	2,419	98	50.55	30.61	69.54	92
		1997	1,763	1,117	2,490	98	49.63	31.45	70.10	92
		1998	1,794	1,169	2,518	99	49.54	32.30	69.53	91
		1999	2,022	1,489	2,503	97	54.39	40.04	67.33	90
		2000	1,840	1,281	2,416	99	48.95	34.07	64.26	93
		2001	2,036	1,810	2,330	98	53.44	47.51	61.14	92
		2002	1,889	1,385	2,350	99	48.69	35.70	60.56	94
		2003	1,976	1,444	2,591	99	49.99	36.52	65.53	94
		2004	2,117	1,511	3,042	99	52.18	37.23	74.97	93
		2005	2,249	1,568	3,494	97	54.40	37.92	84.50	89
		2006	2,408	1,642	4,007	96	56.63	38.61	94.24	83
		2007	2,558	1,711	4,521	96	58.94	39.43	104.17	79
	Non-Hispanic White	1992	1,240	944	1,484	97	50.21	38.24	60.10	74
		1993	978	465	1,375	98	39.85	18.95	56.05	83
		1994	1,073	780	1,288	98	44.24	32.17	53.09	82
		1995	881	497	1,219	98	37.10	20.93	51.32	84
		1996	855	518	1,176	98	36.57	22.14	50.30	85
		1997	876	555	1,237	99	36.88	23.37	52.09	82
		1998	916	597	1,285	99	37.95	24.74	53.26	82
		1999	1,065	784	1,318	99	43.11	31.74	53.37	79
		2000	1,002	697	1,315	98	40.27	28.03	52.87	80
		2001	1,145	1,018	1,311	98	45.49	40.45	52.05	79
		2002	1,097	804	1,364	98	42.75	31.35	53.17	79
		2003	1,180	862	1,547	97	45.13	32.97	59.17	80
		2004	1,296	925	1,862	97	48.25	34.43	69.33	77
		2005	1,404	979	2,181	97	51.38	35.82	79.81	73
		2006	1,525	1,040	2,537	97	54.21	36.96	90.21	72
		2007	1,632	1,092	2,885	94	56.85	38.02	100.46	69

Metropolitan Statistical Area	PWID Population	Year	Number	Min	Max	Rank	Rate	Min	Max	Rank
Charleston--North Charleston, SC	Non-Hispanic Black	1992	959	730	1,148	81	95.13	72.47	113.88	97
		1993	850	404	1,196	83	83.40	39.66	117.31	98
		1994	995	724	1,194	75	97.58	70.95	117.10	96
		1995	834	470	1,153	77	82.15	46.35	113.64	95
		1996	797	483	1,096	76	78.92	47.79	108.56	95
		1997	781	495	1,103	75	75.54	47.87	106.69	95
		1998	762	497	1,070	76	72.36	47.17	101.55	94
		1999	810	596	1,003	70	75.07	55.28	92.94	93
		2000	686	477	900	75	62.54	43.53	82.11	94
		2001	698	620	798	73	62.83	55.86	71.88	93
		2002	592	434	736	77	52.47	38.48	65.26	94
		2003	565	413	740	78	49.31	36.02	64.64	95
		2004	555	396	797	79	47.51	33.90	68.26	97
		2005	547	381	849	79	45.90	31.99	71.29	97
		2006	554	378	921	79	45.53	31.05	75.77	96
		2007	572	382	1,010	78	46.30	30.97	81.82	96
	Hispanic	1992	24	18	29	93	41.67	31.74	49.88	89
		1993	20	9	28	93	33.88	16.11	47.66	91
		1994	23	16	27	95	38.32	27.86	45.98	91
		1995	19	11	27	96	30.66	17.30	42.41	92
		1996	19	12	27	96	29.53	17.88	40.62	91
		1997	20	13	29	96	27.78	17.60	39.23	89
		1998	22	14	31	95	27.20	17.73	38.17	89
		1999	26	19	33	94	29.30	21.58	36.28	88
		2000	26	18	34	94	26.89	18.72	35.30	86
		2001	30	27	35	94	30.55	27.17	34.96	84
		2002	30	22	37	94	29.18	21.40	36.30	83
		2003	33	24	44	94	31.29	22.86	41.02	79
		2004	38	27	54	94	33.13	23.64	47.61	77
		2005	42	29	66	94	35.72	24.90	55.48	72
		2006	48	32	79	93	37.30	25.43	62.06	73
		2007	53	35	94	91	39.33	26.31	69.51	71

Metropolitan Statistical Area	PWID Population	Year	Number	Min	Max	Rank	Rate	Min	Max	Rank
Charleston--North Charleston, SC	Male	1992	1,773	1,351	2,123	93	98.13	74.75	117.46	81
		1993	1,454	692	2,046	95	81.62	38.81	114.80	85
		1994	1,629	1,184	1,955	95	92.35	67.15	110.83	83
		1995	1,340	756	1,854	95	77.25	43.59	106.86	85
		1996	1,285	778	1,768	96	74.93	45.37	103.07	84
		1997	1,286	815	1,817	96	73.52	46.59	103.84	85
		1998	1,302	849	1,828	96	72.76	47.43	102.11	83
		1999	1,456	1,072	1,803	94	78.94	58.12	97.73	81
		2000	1,312	913	1,722	95	70.31	48.94	92.30	81
		2001	1,433	1,274	1,639	95	75.91	67.49	86.85	82
		2002	1,310	960	1,629	97	68.22	50.03	84.85	83
		2003	1,348	984	1,767	98	69.17	50.53	90.68	83
		2004	1,418	1,012	2,037	98	70.82	50.53	101.76	82
		2005	1,479	1,031	2,297	97	72.80	50.74	113.07	79
		2006	1,553	1,059	2,585	95	74.06	50.50	123.23	78
		2007	1,620	1,083	2,863	96	75.74	50.66	133.85	75
	Female	1992	720	548	862	95	40.31	30.70	48.25	85
		1993	567	269	797	97	31.32	14.89	44.06	91
		1994	621	452	746	96	34.47	25.06	41.36	92
		1995	509	287	704	100	28.59	16.13	39.55	94
		1996	493	299	679	100	27.97	16.94	38.48	95
		1997	505	320	713	100	28.00	17.75	39.55	95
		1998	528	344	740	99	28.81	18.78	40.43	95
		1999	613	451	759	99	32.74	24.10	40.53	92
		2000	577	401	757	99	30.44	21.18	39.95	95
		2001	659	586	754	99	34.27	30.47	39.21	93
		2002	631	462	784	99	32.16	23.58	39.99	95
		2003	678	495	888	98	33.79	24.68	44.29	95
		2004	742	529	1,066	98	36.10	25.75	51.87	94
		2005	800	558	1,243	97	38.04	26.52	59.09	91
		2006	863	588	1,435	97	40.03	27.29	66.60	87
		2007	913	611	1,614	96	41.48	27.75	73.31	84

Metropolitan Statistical Area	PWID Population	Year	Number	Min	Max	Rank	Rate	Min	Max	Rank
Charleston--North Charleston, SC	Young (15-29)	1992	678	516	811	93	49.29	37.55	59.00	80
		1993	475	226	668	97	35.80	17.02	50.36	88
		1994	481	350	577	97	37.36	27.17	44.84	91
		1995	378	213	523	97	30.29	17.09	41.90	93
		1996	363	220	499	99	29.69	17.98	40.85	93
		1997	378	240	534	99	30.68	19.45	43.34	92
		1998	411	268	576	97	32.93	21.46	46.21	93
		1999	502	370	622	97	39.66	29.20	49.09	93
		2000	501	348	657	99	39.70	27.63	52.12	89
		2001	607	539	694	98	48.26	42.91	55.21	86
		2002	612	449	761	99	48.30	35.42	60.07	87
		2003	687	502	901	99	53.73	39.25	70.44	85
		2004	775	553	1,114	98	58.85	41.99	84.56	85
		2005	847	590	1,316	98	63.63	44.35	98.82	81
		2006	906	618	1,508	98	65.59	44.72	109.13	84
		2007	929	621	1,641	97	66.34	44.38	117.24	81
	Old (30-64)	1992	1,854	1,412	2,219	95	83.57	63.66	100.03	77
		1993	1,571	747	2,209	96	69.33	32.97	97.51	85
		1994	1,790	1,302	2,148	95	78.56	57.13	94.28	83
		1995	1,486	838	2,055	96	65.51	36.96	90.62	84
		1996	1,427	864	1,963	96	63.28	38.31	87.04	85
		1997	1,424	903	2,012	96	61.41	38.92	86.74	85
		1998	1,432	933	2,009	97	60.30	39.31	84.63	83
		1999	1,582	1,165	1,958	97	64.54	47.52	79.90	83
		2000	1,402	976	1,840	98	56.09	39.04	73.63	85
		2001	1,500	1,333	1,716	97	58.73	52.22	67.20	82
		2002	1,338	981	1,664	98	51.21	37.55	63.69	88
		2003	1,341	980	1,758	98	50.13	36.63	65.72	88
		2004	1,375	981	1,975	98	50.16	35.79	72.07	85
		2005	1,401	976	2,175	95	49.96	34.82	77.59	84
		2006	1,447	987	2,408	95	50.41	34.37	83.88	81
		2007	1,500	1,003	2,650	95	51.00	34.11	90.13	76

Metropolitan Statistical Area	PWID Population	Year	Number	Min	Max	Rank	Rate	Min	Max	Rank
Charlotte--Gastonia--Rock Hill, NC--SC	Total	1992	7,224	6,344	8,230	59	87.10	76.48	99.22	65
		1993	6,154	3,388	8,171	64	72.64	39.99	96.46	73
		1994	6,954	6,195	8,155	61	80.11	71.36	93.95	73
		1995	5,967	3,311	8,167	69	66.81	37.07	91.44	77
		1996	5,888	3,266	8,210	69	64.02	35.51	89.27	78
		1997	5,806	3,220	8,253	68	61.27	33.98	87.09	80
		1998	5,774	3,166	8,456	68	59.17	32.44	86.66	81
		1999	6,614	5,170	8,664	65	65.82	51.45	86.22	77
		2000	5,780	2,908	9,222	67	56.00	28.18	89.35	82
		2001	6,845	4,869	9,777	64	64.75	46.06	92.49	75
		2002	6,014	2,652	10,698	66	55.69	24.56	99.05	83
		2003	6,193	2,505	11,630	65	56.24	22.75	105.62	82
		2004	6,408	2,377	12,655	64	56.92	21.12	112.41	81
		2005	6,695	2,279	13,841	59	57.66	19.62	119.20	80
		2006	7,069	2,189	15,259	55	58.56	18.13	126.42	79
		2007	7,458	2,107	16,747	54	59.51	16.81	133.64	78
	Non-Hispanic White	1992	3,675	3,227	4,186	62	57.10	50.14	65.05	68
		1993	3,130	1,723	4,156	66	47.94	26.39	63.65	72
		1994	3,545	3,158	4,157	63	53.40	47.57	62.62	69
		1995	3,055	1,695	4,181	66	45.10	25.03	61.73	75
		1996	3,032	1,681	4,227	66	43.89	24.34	61.20	76
		1997	3,010	1,669	4,279	69	42.76	23.71	60.78	79
		1998	3,016	1,654	4,417	69	42.08	23.07	61.63	77
		1999	3,483	2,722	4,562	66	47.77	37.34	62.58	73
		2000	3,068	1,544	4,895	69	41.48	20.87	66.19	78
		2001	3,661	2,604	5,230	62	48.78	34.70	69.67	74
		2002	3,240	1,429	5,762	67	42.61	18.79	75.80	82
		2003	3,356	1,358	6,303	68	43.64	17.65	81.96	82
		2004	3,490	1,295	6,892	65	44.76	16.60	88.38	83
		2005	3,658	1,245	7,562	65	45.86	15.61	94.82	82
		2006	3,868	1,198	8,349	60	47.08	14.58	101.63	82
		2007	4,077	1,152	9,157	60	48.23	13.63	108.30	82

Metropolitan Statistical Area	PWID Population	Year	Number	Min	Max	Rank	Rate	Min	Max	Rank
Charlotte--Gastonia--Rock Hill, NC--SC	Non-Hispanic Black	1992	3,078	2,703	3,506	36	190.00	166.85	216.46	80
		1993	2,712	1,493	3,601	37	162.69	89.57	216.02	83
		1994	3,101	2,762	3,636	31	180.21	160.53	211.33	75
		1995	2,645	1,468	3,620	35	148.41	82.35	203.12	79
		1996	2,559	1,419	3,567	34	138.81	76.99	193.54	79
		1997	2,448	1,357	3,479	34	128.33	71.16	182.41	80
		1998	2,346	1,286	3,435	34	119.27	65.40	174.67	79
		1999	2,582	2,018	3,382	33	127.53	99.69	167.06	79
		2000	2,171	1,092	3,464	34	104.41	52.54	166.60	80
		2001	2,491	1,772	3,558	30	115.66	82.28	165.22	78
		2002	2,145	946	3,815	33	96.35	42.49	171.39	80
		2003	2,201	890	4,134	32	95.93	38.80	180.16	79
		2004	2,320	861	4,582	29	97.73	36.25	193.00	74
		2005	2,532	862	5,234	27	102.26	34.80	211.40	72
		2006	2,867	888	6,188	25	110.18	34.12	237.85	67
		2007	3,324	939	7,466	23	122.14	34.51	274.28	63
	Hispanic	1992	21	18	24	94	18.73	16.44	21.33	99
		1993	19	10	25	94	13.97	7.69	18.55	100
		1994	23	21	27	95	13.94	12.42	16.35	100
		1995	23	13	31	93	11.04	6.13	15.11	100
		1996	26	15	37	93	10.21	5.66	14.24	100
		1997	31	17	44	92	9.62	5.33	13.67	100
		1998	37	20	54	91	9.31	5.11	13.64	100
		1999	51	40	67	91	10.51	8.21	13.76	100
		2000	53	27	85	88	9.28	4.67	14.81	100
		2001	74	53	106	86	11.84	8.42	16.91	100
		2002	75	33	133	86	11.10	4.90	19.75	100
		2003	86	35	161	83	11.97	4.84	22.48	100
		2004	94	35	186	84	12.42	4.61	24.54	99
		2005	100	34	207	83	12.29	4.18	25.40	99
		2006	102	31	219	84	11.48	3.55	24.77	100
		2007	97	27	218	85	10.01	2.83	22.48	100

Metropolitan Statistical Area	PWID Population	Year	Number	Min	Max	Rank	Rate	Min	Max	Rank
Charlotte--Gastonia--Rock Hill, NC--SC	Male	1992	4,523	3,972	5,153	61	110.77	97.27	126.19	66
		1993	3,848	2,118	5,109	69	92.30	50.81	122.55	77
		1994	4,345	3,870	5,095	64	101.55	90.46	119.09	77
		1995	3,727	2,068	5,101	69	84.56	46.92	115.73	79
		1996	3,677	2,039	5,127	69	80.93	44.89	112.84	81
		1997	3,624	2,010	5,151	68	77.26	42.85	109.83	80
		1998	3,600	1,974	5,272	69	74.44	40.82	109.02	82
		1999	4,116	3,217	5,392	65	82.42	64.42	107.97	78
		2000	3,585	1,804	5,721	71	69.64	35.04	111.12	83
		2001	4,227	3,007	6,037	64	80.20	57.05	114.56	77
		2002	3,689	1,627	6,563	68	68.57	30.24	121.97	82
		2003	3,766	1,523	7,072	67	68.86	27.85	129.33	84
		2004	3,852	1,429	7,607	65	68.90	25.56	136.06	86
		2005	3,966	1,350	8,198	64	68.86	23.44	142.36	86
		2006	4,110	1,273	8,872	60	68.64	21.25	148.18	84
		2007	4,238	1,197	9,517	60	68.40	19.33	153.60	85
	Female	1992	2,694	2,365	3,069	58	63.97	56.17	72.87	60
		1993	2,350	1,294	3,120	62	54.62	30.07	72.53	66
		1994	2,700	2,405	3,166	59	61.32	54.62	71.91	63
		1995	2,338	1,297	3,200	60	51.68	28.68	70.73	68
		1996	2,313	1,283	3,225	62	49.71	27.57	69.31	73
		1997	2,273	1,261	3,231	62	47.49	26.34	67.51	77
		1998	2,239	1,228	3,279	64	45.49	24.94	66.63	77
		1999	2,526	1,975	3,310	59	49.98	39.06	65.47	76
		2000	2,162	1,088	3,450	67	41.81	21.04	66.71	84
		2001	2,495	1,775	3,564	62	47.06	33.48	67.22	84
		2002	2,124	937	3,778	68	39.19	17.28	69.71	89
		2003	2,108	853	3,960	71	38.03	15.38	71.43	89
		2004	2,092	776	4,132	72	36.92	13.69	72.90	93
		2005	2,086	710	4,312	68	35.64	12.13	73.67	94
		2006	2,091	647	4,514	74	34.37	10.64	74.20	94
		2007	2,085	589	4,682	75	32.91	9.30	73.90	95

Metropolitan Statistical Area	PWID Population	Year	Number	Min	Max	Rank	Rate	Min	Max	Rank
Charlotte--Gastonia--Rock Hill, NC--SC	Young (15-29)	1992	1,868	1,640	2,128	59	66.15	58.09	75.36	65
		1993	1,540	848	2,044	66	54.50	30.01	72.37	71
		1994	1,686	1,501	1,977	66	59.16	52.70	69.38	69
		1995	1,404	779	1,922	71	48.55	26.94	66.45	74
		1996	1,350	749	1,883	71	45.79	25.40	63.84	82
		1997	1,303	723	1,852	73	43.17	23.94	61.37	84
		1998	1,275	699	1,868	76	41.37	22.68	60.58	85
		1999	1,447	1,131	1,896	77	46.18	36.09	60.49	86
		2000	1,263	636	2,015	82	39.58	19.91	63.15	91
		2001	1,507	1,072	2,153	80	47.17	33.55	67.38	87
		2002	1,348	594	2,397	84	42.09	18.56	74.87	97
		2003	1,428	578	2,682	82	44.51	18.01	83.60	96
		2004	1,539	571	3,038	80	47.22	17.52	93.25	97
		2005	1,694	577	3,503	77	50.40	17.15	104.20	96
		2006	1,909	591	4,121	76	54.36	16.83	117.34	94
		2007	2,176	615	4,886	67	59.93	16.93	134.57	88
	Old (30-64)	1992	5,437	4,775	6,194	58	99.39	87.28	113.23	65
		1993	4,663	2,567	6,191	64	82.57	45.46	109.64	74
		1994	5,315	4,734	6,232	61	91.13	81.18	106.87	71
		1995	4,605	2,555	6,302	66	76.25	42.31	104.36	76
		1996	4,589	2,545	6,398	65	73.44	40.73	102.39	75
		1997	4,565	2,531	6,489	65	70.68	39.20	100.47	76
		1998	4,572	2,507	6,696	63	68.49	37.55	100.31	76
		1999	5,262	4,113	6,893	56	76.10	59.48	99.69	73
		2000	4,606	2,317	7,349	61	64.59	32.50	103.07	73
		2001	5,443	3,872	7,775	55	73.79	52.49	105.40	69
		2002	4,750	2,095	8,450	58	62.52	27.57	111.21	74
		2003	4,832	1,955	9,075	56	61.92	25.05	116.30	74
		2004	4,905	1,820	9,687	53	61.32	22.75	121.09	74
		2005	4,981	1,695	10,296	52	60.37	20.55	124.81	72
		2006	5,044	1,562	10,889	51	58.94	18.25	127.24	71
		2007	5,013	1,417	11,258	48	56.32	15.91	126.48	69

Metropolitan Statistical Area	PWID Population	Year	Number	Min	Max	Rank	Rate	Min	Max	Rank
Chicago, IL	Total	1992	36,552	15,837	58,582	5	72.10	31.24	115.56	79
		1993	31,875	17,413	55,069	9	62.16	33.96	107.39	82
		1994	36,775	20,352	51,425	5	71.00	39.29	99.28	81
		1995	31,812	16,484	47,943	7	60.84	31.52	91.69	81
		1996	31,918	16,067	44,743	7	60.40	30.40	84.67	82
		1997	32,084	15,650	43,438	7	60.11	29.32	81.38	81
		1998	32,425	15,263	45,256	7	59.98	28.23	83.71	79
		1999	38,744	32,779	47,020	7	70.91	59.99	86.05	74
		2000	32,964	14,185	48,978	7	59.64	25.67	88.62	76
		2001	39,767	31,779	50,956	7	71.16	56.86	91.18	70
		2002	33,238	14,619	52,760	7	58.98	25.94	93.62	77
		2003	33,027	14,793	54,490	7	58.22	26.08	96.06	79
		2004	32,711	15,085	56,317	8	57.19	26.37	98.46	80
		2005	32,404	15,399	58,112	8	56.25	26.73	100.87	83
		2006	32,112	15,733	60,083	8	55.28	27.08	103.43	86
		2007	31,814	16,126	62,046	8	54.35	27.55	106.00	89
	Non-Hispanic White	1992	12,355	5,353	19,802	15	37.39	16.20	59.93	89
		1993	10,222	5,584	17,660	20	30.93	16.90	53.43	91
		1994	11,424	6,322	15,975	14	34.60	19.15	48.39	91
		1995	9,749	5,052	14,693	21	29.60	15.34	44.62	93
		1996	9,801	4,934	13,740	18	29.82	15.01	41.80	93
		1997	9,994	4,875	13,531	17	30.47	14.86	41.25	91
		1998	10,340	4,867	14,432	16	31.52	14.84	43.99	91
		1999	12,723	10,764	15,440	12	38.88	32.89	47.19	84
		2000	11,179	4,810	16,609	15	34.18	14.71	50.79	87
		2001	13,926	11,128	17,844	9	42.39	33.88	54.32	82
		2002	11,987	5,272	19,027	14	36.45	16.03	57.86	87
		2003	12,203	5,466	20,134	14	37.13	16.63	61.26	91
		2004	12,292	5,668	21,163	14	37.34	17.22	64.28	93
		2005	12,267	5,829	22,000	15	37.25	17.70	66.81	94
		2006	12,107	5,932	22,653	15	36.73	17.99	68.72	93
		2007	11,785	5,974	22,984	16	35.72	18.11	69.66	92

Metropolitan Statistical Area	PWID Population	Year	Number	Min	Max	Rank	Rate	Min	Max	Rank
Chicago, IL	Non-Hispanic Black	1992	19,525	8,460	31,292	5	207.92	90.09	333.24	73
		1993	17,227	9,411	29,763	6	181.52	99.16	313.60	74
		1994	19,818	10,968	27,713	5	207.03	114.57	289.50	68
		1995	16,891	8,753	25,457	5	174.85	90.60	263.51	71
		1996	16,538	8,325	23,184	5	169.81	85.48	238.04	68
		1997	16,101	7,854	21,799	5	163.93	79.96	221.94	66
		1998	15,680	7,381	21,885	6	158.38	74.55	221.05	68
		1999	18,012	15,239	21,859	3	180.57	152.77	219.14	59
		2000	14,748	6,346	21,913	6	147.00	63.26	218.41	66
		2001	17,205	13,749	22,045	4	170.91	136.58	219.00	56
		2002	14,030	6,171	22,270	5	138.94	61.11	220.55	61
		2003	13,781	6,173	22,737	5	136.09	60.95	224.52	59
		2004	13,723	6,328	23,627	5	134.65	62.09	231.83	58
		2005	13,944	6,626	25,006	3	136.11	64.68	244.10	59
		2006	14,480	7,094	27,092	3	140.57	68.87	263.01	55
		2007	15,344	7,778	29,925	3	148.47	75.26	289.56	51
	Hispanic	1992	3,408	1,477	5,462	24	55.11	23.88	88.32	83
		1993	3,123	1,706	5,396	26	47.80	26.11	82.57	82
		1994	3,773	2,088	5,276	21	54.75	30.30	76.56	78
		1995	3,402	1,763	5,127	21	46.86	24.28	70.62	79
		1996	3,541	1,782	4,963	20	46.13	23.22	64.67	79
		1997	3,673	1,791	4,972	18	45.45	22.17	61.53	76
		1998	3,809	1,793	5,317	16	44.55	20.97	62.18	74
		1999	4,645	3,929	5,637	16	51.62	43.68	62.65	71
		2000	4,009	1,725	5,956	17	42.45	18.27	63.07	74
		2001	4,876	3,897	6,248	16	49.91	39.88	63.95	69
		2002	4,085	1,797	6,484	17	40.54	17.83	64.35	72
		2003	4,044	1,811	6,672	17	39.08	17.50	64.48	71
		2004	3,967	1,830	6,831	17	37.40	17.25	64.39	72
		2005	3,872	1,840	6,944	18	35.56	16.90	63.78	73
		2006	3,762	1,843	7,038	19	33.62	16.47	62.90	75
		2007	3,638	1,844	7,096	20	31.66	16.05	61.74	77

Metropolitan Statistical Area	PWID Population	Year	Number	Min	Max	Rank	Rate	Min	Max	Rank
Chicago, IL	Male	1992	25,367	10,991	40,656	5	101.14	43.82	162.10	78
		1993	21,964	11,999	37,946	7	86.57	47.29	149.56	81
		1994	25,143	13,915	35,159	5	98.07	54.27	137.14	79
		1995	21,568	11,176	32,505	7	83.27	43.15	125.50	81
		1996	21,449	10,797	30,068	7	81.89	41.22	114.80	79
		1997	21,362	10,420	28,922	7	80.74	39.38	109.31	79
		1998	21,384	10,066	29,846	7	79.78	37.55	111.35	78
		1999	25,304	21,408	30,709	7	93.40	79.02	113.35	69
		2000	21,319	9,174	31,676	7	77.68	33.43	115.42	76
		2001	25,470	20,353	32,636	7	91.69	73.27	117.49	66
		2002	21,086	9,274	33,469	8	75.22	33.08	119.40	77
		2003	20,759	9,298	34,249	9	73.55	32.94	121.34	78
		2004	20,381	9,398	35,088	9	71.48	32.96	123.06	81
		2005	20,025	9,516	35,913	9	69.67	33.11	124.93	83
		2006	19,699	9,651	36,857	11	67.91	33.27	127.05	86
		2007	19,390	9,828	37,815	11	66.28	33.60	129.27	89
	Female	1992	11,209	4,856	17,964	7	43.76	18.96	70.14	84
		1993	10,009	5,468	17,291	12	38.63	21.10	66.74	85
		1994	11,824	6,544	16,534	6	45.20	25.02	63.21	83
		1995	10,469	5,425	15,777	8	39.67	20.56	59.78	85
		1996	10,741	5,407	15,057	8	40.30	20.29	56.49	85
		1997	11,027	5,379	14,929	7	40.96	19.98	55.45	86
		1998	11,363	5,348	15,859	7	41.69	19.62	58.18	83
		1999	13,814	11,687	16,765	7	50.15	42.43	60.86	75
		2000	11,930	5,134	17,726	6	42.88	18.45	63.71	81
		2001	14,569	11,642	18,667	6	51.83	41.42	66.42	71
		2002	12,287	5,404	19,504	6	43.38	19.08	68.86	82
		2003	12,278	5,499	20,256	7	43.08	19.29	71.07	83
		2004	12,182	5,618	20,973	8	42.47	19.59	73.12	83
		2005	12,038	5,721	21,589	8	41.71	19.82	74.79	84
		2006	11,846	5,804	22,165	8	40.74	19.96	76.22	86
		2007	11,597	5,878	22,617	8	39.60	20.07	77.24	86

Metropolitan Statistical Area	PWID Population	Year	Number	Min	Max	Rank	Rate	Min	Max	Rank
Chicago, IL	Young (15-29)	1992	7,414	3,212	11,882	13	42.87	18.58	68.71	85
		1993	5,959	3,255	10,295	18	34.70	18.96	59.95	89
		1994	6,701	3,708	9,370	11	39.21	21.70	54.84	88
		1995	5,925	3,070	8,930	16	34.64	17.95	52.20	88
		1996	6,312	3,177	8,849	11	36.70	18.48	51.45	89
		1997	6,925	3,378	9,376	11	40.00	19.51	54.16	86
		1998	7,768	3,657	10,842	7	44.37	20.89	61.93	81
		1999	10,371	8,774	12,587	7	58.81	49.76	71.37	72
		2000	9,833	4,231	14,610	6	55.36	23.82	82.25	76
		2001	13,075	10,449	16,754	6	73.71	58.90	94.45	67
		2002	11,836	5,206	18,788	6	66.96	29.45	106.29	72
		2003	12,445	5,574	20,532	6	70.49	31.57	116.30	71
		2004	12,678	5,846	21,826	6	71.29	32.87	122.73	76
		2005	12,486	5,933	22,392	6	69.92	33.23	125.39	77
		2006	11,804	5,783	22,086	7	65.62	32.15	122.78	83
		2007	10,593	5,370	20,660	9	58.53	29.67	114.16	89
	Old (30-64)	1992	29,737	12,884	47,659	5	89.03	38.57	142.69	72
		1993	26,095	14,255	45,082	7	76.51	41.80	132.18	79
		1994	30,053	16,632	42,025	5	86.58	47.92	121.08	76
		1995	25,775	13,356	38,845	7	73.26	37.96	110.41	79
		1996	25,482	12,827	35,721	7	71.49	35.98	100.21	78
		1997	25,091	12,239	33,970	7	69.57	33.93	94.19	77
		1998	24,701	11,627	34,476	7	67.58	31.81	94.32	77
		1999	28,611	24,206	34,723	7	77.32	65.41	93.83	72
		2000	23,514	10,119	34,937	8	62.69	26.98	93.15	77
		2001	27,360	21,864	35,057	7	71.72	57.32	91.90	73
		2002	22,088	9,715	35,061	11	57.11	25.12	90.64	81
		2003	21,308	9,544	35,154	11	54.53	24.42	89.97	79
		2004	20,673	9,533	35,592	11	52.45	24.19	90.31	81
		2005	20,321	9,657	36,443	9	51.12	24.29	91.68	81
		2006	20,303	9,947	37,988	9	50.63	24.81	94.74	79
		2007	20,635	10,460	40,244	9	51.03	25.87	99.52	75

Metropolitan Statistical Area	PWID Population	Year	Number	Min	Max	Rank	Rate	Min	Max	Rank
Cincinnati, OH--KY--IN	Total	1992	8,149	1,895	15,519	55	80.31	18.67	152.94	70
		1993	6,621	2,147	14,684	61	64.56	20.93	143.18	81
		1994	7,535	2,415	13,835	58	73.01	23.40	134.05	78
		1995	6,146	2,692	13,002	67	59.05	25.86	124.92	82
		1996	5,951	2,830	12,265	68	56.72	26.98	116.90	84
		1997	5,765	2,804	11,525	69	54.46	26.49	108.88	84
		1998	5,606	2,781	10,828	69	52.42	26.01	101.26	88
		1999	6,334	4,023	10,098	66	58.73	37.30	93.63	82
		2000	5,503	2,823	9,955	71	50.51	25.91	91.37	91
		2001	6,487	4,418	9,840	66	58.76	40.01	89.12	82
		2002	5,737	3,096	9,810	72	51.42	27.75	87.92	90
		2003	5,872	3,230	9,774	71	52.11	28.66	86.73	89
		2004	6,129	3,699	9,831	69	53.79	32.47	86.29	89
		2005	6,418	3,607	9,901	67	55.65	31.28	85.85	84
		2006	6,721	3,425	10,004	61	57.68	29.40	85.85	80
		2007	7,056	3,246	10,126	59	59.83	27.52	85.85	77
	Non-Hispanic White	1992	5,043	1,172	9,602	48	57.71	13.42	109.89	67
		1993	4,065	1,318	9,014	54	46.14	14.96	102.32	74
		1994	4,565	1,463	8,382	49	51.59	16.53	94.73	74
		1995	3,663	1,604	7,748	60	41.14	18.02	87.03	79
		1996	3,485	1,657	7,182	62	38.93	18.51	80.22	81
		1997	3,321	1,616	6,640	64	36.86	17.93	73.68	83
		1998	3,189	1,582	6,160	66	35.11	17.42	67.82	85
		1999	3,582	2,275	5,710	63	39.18	24.89	62.47	83
		2000	3,120	1,600	5,644	67	33.88	17.38	61.28	88
		2001	3,729	2,540	5,656	61	40.07	27.29	60.77	85
		2002	3,386	1,827	5,789	64	36.09	19.48	61.72	89
		2003	3,604	1,983	5,999	60	38.12	20.97	63.45	88
		2004	3,960	2,390	6,352	57	41.54	25.08	66.64	86
		2005	4,410	2,479	6,803	49	45.82	25.75	70.68	83
		2006	4,942	2,519	7,356	43	50.96	25.97	75.86	78
		2007	5,563	2,559	7,982	39	56.82	26.14	81.53	71

Metropolitan Statistical Area	PWID Population	Year	Number	Min	Max	Rank	Rate	Min	Max	Rank
Cincinnati, OH--KY--IN	Non-Hispanic Black	1992	2,770	644	5,275	41	224.76	52.26	428.02	69
		1993	2,241	727	4,970	43	178.38	57.83	395.58	76
		1994	2,565	822	4,710	39	201.48	64.57	369.94	71
		1995	2,117	927	4,478	43	163.67	71.67	346.21	75
		1996	2,078	988	4,283	43	158.49	75.38	326.64	74
		1997	2,038	991	4,074	43	153.14	74.49	306.15	75
		1998	1,995	990	3,854	41	147.92	73.39	285.72	71
		1999	2,249	1,429	3,586	38	164.98	104.78	263.03	67
		2000	1,925	987	3,483	41	139.63	71.62	252.58	68
		2001	2,198	1,497	3,334	39	156.05	106.28	236.69	63
		2002	1,843	994	3,151	43	128.55	69.36	219.81	67
		2003	1,740	957	2,897	43	119.47	65.72	198.85	66
		2004	1,622	979	2,601	46	109.16	65.89	175.10	71
		2005	1,457	819	2,247	54	96.16	54.05	148.33	73
		2006	1,249	637	1,859	60	80.85	41.20	120.34	79
		2007	1,019	469	1,462	65	64.55	29.69	92.63	88
	Hispanic	1992	44	10	83	87	70.60	16.42	134.45	71
		1993	43	14	95	87	63.77	20.68	141.43	71
		1994	56	18	103	85	77.82	24.94	142.89	66
		1995	51	22	107	86	65.41	28.64	138.36	65
		1996	52	25	108	86	62.22	29.59	128.22	67
		1997	53	26	105	86	57.80	28.12	115.55	68
		1998	51	26	99	88	51.65	25.63	99.77	72
		1999	57	36	91	88	52.85	33.57	84.26	69
		2000	48	25	87	91	39.68	20.35	71.79	75
		2001	54	37	82	91	41.47	28.24	62.90	74
		2002	46	25	78	91	32.83	17.71	56.13	79
		2003	44	24	74	91	30.29	16.66	50.41	81
		2004	44	27	71	92	28.24	17.05	45.31	82
		2005	44	25	69	92	26.89	15.12	41.48	84
		2006	46	23	68	94	26.09	13.30	38.84	83
		2007	48	22	69	94	25.91	11.92	37.18	85

Metropolitan Statistical Area	PWID Population	Year	Number	Min	Max	Rank	Rate	Min	Max	Rank
Cincinnati, OH--KY--IN	Male	1992	5,422	1,261	10,324	54	109.28	25.41	208.11	68
		1993	4,382	1,421	9,719	62	87.31	28.31	193.62	80
		1994	4,944	1,584	9,077	59	97.78	31.33	179.53	80
		1995	3,985	1,745	8,429	67	78.06	34.18	165.12	84
		1996	3,803	1,809	7,837	67	73.84	35.12	152.18	86
		1997	3,623	1,763	7,244	69	69.71	33.91	139.36	86
		1998	3,461	1,717	6,685	73	65.85	32.67	127.20	88
		1999	3,839	2,438	6,120	67	72.34	45.94	115.32	85
		2000	3,275	1,680	5,924	75	61.03	31.30	110.39	92
		2001	3,794	2,584	5,755	71	69.71	47.48	105.74	86
		2002	3,305	1,783	5,651	75	60.07	32.41	102.71	94
		2003	3,342	1,839	5,563	74	60.08	33.05	100.00	94
		2004	3,460	2,089	5,551	72	61.47	37.11	98.61	94
		2005	3,614	2,031	5,574	69	63.35	35.61	97.72	94
		2006	3,797	1,935	5,651	67	65.85	33.56	98.02	91
		2007	4,027	1,852	5,778	65	68.94	31.71	98.93	84
	Female	1992	2,881	670	5,486	52	55.55	12.92	105.79	68
		1993	2,376	770	5,269	61	45.38	14.71	100.64	80
		1994	2,755	883	5,058	57	52.32	16.77	96.07	75
		1995	2,295	1,005	4,856	61	43.28	18.95	91.56	82
		1996	2,274	1,082	4,687	63	42.57	20.25	87.74	84
		1997	2,256	1,097	4,510	64	41.88	20.37	83.73	84
		1998	2,246	1,114	4,339	63	41.31	20.50	79.79	84
		1999	2,597	1,649	4,140	58	47.40	30.11	75.57	82
		2000	2,304	1,182	4,168	64	41.68	21.38	75.40	85
		2001	2,768	1,885	4,198	58	49.44	33.67	74.99	79
		2002	2,487	1,342	4,252	63	43.97	23.73	75.18	80
		2003	2,576	1,417	4,288	60	45.14	24.83	75.13	80
		2004	2,710	1,635	4,346	57	47.01	28.38	75.41	78
		2005	2,845	1,599	4,389	52	48.82	27.44	75.31	75
		2006	2,971	1,514	4,422	52	50.46	25.72	75.11	69
		2007	3,090	1,421	4,434	49	51.91	23.88	74.49	68

Metropolitan Statistical Area	PWID Population	Year	Number	Min	Max	Rank	Rate	Min	Max	Rank
Cincinnati, OH--KY--IN	Young (15-29)	1992	2,117	492	4,031	52	62.17	14.45	118.39	71
		1993	1,523	494	3,377	67	44.99	14.59	99.77	78
		1994	1,606	515	2,950	67	47.88	15.34	87.91	81
		1995	1,269	556	2,684	73	37.94	16.61	80.26	86
		1996	1,238	589	2,551	74	37.01	17.60	76.28	87
		1997	1,249	607	2,496	75	37.24	18.11	74.44	89
		1998	1,299	644	2,509	74	38.67	19.19	74.69	89
		1999	1,602	1,017	2,553	74	47.89	30.42	76.35	84
		2000	1,538	789	2,782	75	46.10	23.65	83.39	84
		2001	2,016	1,373	3,058	64	60.35	41.10	91.54	77
		2002	1,981	1,069	3,388	66	59.19	31.93	101.20	79
		2003	2,240	1,232	3,729	63	66.35	36.50	110.43	77
		2004	2,556	1,543	4,100	54	74.91	45.22	120.16	69
		2005	2,885	1,622	4,451	47	83.59	46.98	128.94	65
		2006	3,204	1,633	4,769	43	91.85	46.81	136.71	55
		2007	3,502	1,611	5,025	39	99.28	45.67	142.46	45
	Old (30-64)	1992	6,196	1,441	11,799	55	91.91	21.37	175.02	69
		1993	5,248	1,701	11,638	58	76.38	24.76	169.39	80
		1994	6,104	1,956	11,207	53	87.62	28.08	160.89	75
		1995	5,019	2,198	10,617	60	71.06	31.12	150.31	80
		1996	4,847	2,305	9,989	62	67.81	32.25	139.75	80
		1997	4,639	2,257	9,274	63	64.14	31.20	128.24	83
		1998	4,416	2,191	8,530	65	60.21	29.87	116.30	84
		1999	4,841	3,074	7,717	66	65.05	41.32	103.71	81
		2000	4,042	2,073	7,312	67	53.48	27.43	96.74	89
		2001	4,540	3,092	6,887	66	58.96	40.15	89.43	81
		2002	3,799	2,050	6,496	71	48.65	26.25	83.18	92
		2003	3,664	2,016	6,099	74	46.42	25.54	77.27	93
		2004	3,604	2,176	5,782	75	45.16	27.26	72.44	92
		2005	3,579	2,011	5,520	74	44.29	24.89	68.32	91
		2006	3,597	1,833	5,354	71	44.06	22.46	65.58	89
		2007	3,696	1,700	5,303	67	44.70	20.56	64.15	87

Metropolitan Statistical Area	PWID Population	Year	Number	Min	Max	Rank	Rate	Min	Max	Rank
Cleveland--Lorain--Elyria, OH	Total	1992	12,187	6,699	17,766	36	84.82	46.63	123.65	68
		1993	10,322	5,632	17,017	42	71.67	39.10	118.15	74
		1994	11,588	6,971	16,284	41	80.31	48.32	112.86	72
		1995	9,988	6,041	15,567	43	69.02	41.74	107.58	74
		1996	9,848	6,258	14,921	42	67.89	43.14	102.87	75
		1997	9,682	6,460	14,231	42	66.78	44.56	98.16	75
		1998	9,581	6,678	13,754	40	66.10	46.07	94.89	72
		1999	10,336	7,675	13,267	40	71.37	53.00	91.62	72
		2000	9,479	6,868	13,319	41	65.46	47.43	91.98	71
		2001	10,379	8,209	13,389	42	71.58	56.62	92.34	69
		2002	9,507	6,557	13,970	42	65.43	45.13	96.15	73
		2003	9,453	6,024	14,544	44	64.97	41.40	99.95	74
		2004	9,384	5,505	15,149	44	64.42	37.79	103.99	75
		2005	9,310	4,975	15,747	44	63.87	34.13	108.03	73
		2006	9,211	4,445	16,341	46	63.28	30.53	112.26	74
		2007	9,120	3,935	16,939	47	62.72	27.06	116.49	73
	Non-Hispanic White	1992	5,033	2,767	7,338	49	44.37	24.39	64.69	82
		1993	4,051	2,210	6,678	55	35.77	19.52	58.97	86
		1994	4,359	2,623	6,126	52	38.56	23.20	54.19	86
		1995	3,636	2,199	5,667	62	32.18	19.46	50.16	89
		1996	3,504	2,226	5,308	61	31.04	19.73	47.04	89
		1997	3,401	2,269	4,999	63	30.25	20.18	44.46	92
		1998	3,359	2,341	4,821	63	29.97	20.89	43.02	92
		1999	3,655	2,714	4,691	62	32.76	24.33	42.05	93
		2000	3,416	2,475	4,800	62	30.75	22.28	43.20	94
		2001	3,852	3,047	4,969	60	34.70	27.45	44.76	93
		2002	3,669	2,530	5,391	59	33.07	22.81	48.60	95
		2003	3,826	2,438	5,887	57	34.54	22.01	53.14	94
		2004	4,014	2,354	6,480	54	36.30	21.29	58.60	94
		2005	4,233	2,262	7,160	54	38.38	20.51	64.91	91
		2006	4,471	2,157	7,932	54	40.71	19.65	72.23	86
		2007	4,737	2,044	8,799	49	43.32	18.69	80.47	86

Metropolitan Statistical Area	PWID Population	Year	Number	Min	Max	Rank	Rate	Min	Max	Rank
Cleveland--Lorain--Elyria, OH	Non-Hispanic Black	1992	6,026	3,312	8,784	18	243.75	133.99	355.34	62
		1993	5,126	2,797	8,450	19	205.01	111.86	337.97	62
		1994	5,735	3,451	8,060	18	227.30	136.75	319.43	62
		1995	4,894	2,960	7,627	18	192.11	116.18	299.42	65
		1996	4,745	3,015	7,189	19	184.83	117.45	280.04	64
		1997	4,559	3,042	6,701	18	176.71	117.90	259.73	63
		1998	4,384	3,055	6,293	18	169.13	117.88	242.80	61
		1999	4,569	3,393	5,865	20	175.62	130.41	225.42	63
		2000	4,027	2,918	5,659	19	154.07	111.63	216.49	62
		2001	4,218	3,337	5,442	19	160.95	127.30	207.63	62
		2002	3,682	2,540	5,411	20	139.61	96.30	205.16	59
		2003	3,478	2,216	5,351	21	131.16	83.58	201.79	63
		2004	3,274	1,920	5,285	22	122.72	71.98	198.10	63
		2005	3,079	1,645	5,207	24	114.64	61.26	193.90	65
		2006	2,892	1,395	5,130	24	107.12	51.69	190.04	68
		2007	2,729	1,178	5,069	28	100.69	43.44	187.02	73
	Hispanic	1992	865	475	1,260	41	249.73	137.28	364.05	24
		1993	851	464	1,403	42	234.59	127.99	386.73	26
		1994	1,089	655	1,530	39	289.55	174.20	406.91	14
		1995	1,049	634	1,635	39	267.25	161.63	416.54	15
		1996	1,135	721	1,720	39	277.90	176.59	421.05	14
		1997	1,203	803	1,769	39	285.22	190.30	419.22	13
		1998	1,262	880	1,812	39	288.76	201.26	414.54	13
		1999	1,420	1,054	1,822	39	314.15	233.28	403.24	11
		2000	1,336	968	1,878	39	283.16	205.15	397.88	12
		2001	1,479	1,170	1,909	39	303.23	239.84	391.17	12
		2002	1,351	932	1,985	41	268.87	185.45	395.10	12
		2003	1,321	842	2,032	42	255.67	162.92	393.34	13
		2004	1,272	746	2,054	42	240.43	141.03	388.13	14
		2005	1,209	646	2,045	46	222.86	119.09	376.95	16
		2006	1,133	547	2,011	48	204.61	98.74	363.00	18
		2007	1,052	454	1,954	48	183.95	79.36	341.67	18

Metropolitan Statistical Area	PWID Population	Year	Number	Min	Max	Rank	Rate	Min	Max	Rank
Cleveland--Lorain--Elyria, OH	Male	1992	8,208	4,512	11,965	37	118.05	64.89	172.09	62
		1993	7,007	3,823	11,552	40	100.43	54.79	165.55	71
		1994	7,902	4,754	11,104	38	112.97	67.96	158.76	63
		1995	6,821	4,125	10,631	41	97.15	58.76	151.42	68
		1996	6,717	4,268	10,178	40	95.40	60.62	144.55	71
		1997	6,581	4,391	9,673	40	93.48	62.37	137.40	68
		1998	6,478	4,515	9,299	38	91.98	64.11	132.05	68
		1999	6,940	5,153	8,908	37	98.58	73.20	126.54	65
		2000	6,312	4,573	8,869	40	89.61	64.93	125.92	68
		2001	6,848	5,416	8,834	38	97.03	76.75	125.17	65
		2002	6,211	4,284	9,127	41	87.73	60.51	128.92	68
		2003	6,115	3,897	9,408	41	86.11	54.87	132.47	67
		2004	6,011	3,526	9,704	41	84.53	49.58	136.45	68
		2005	5,909	3,158	9,995	42	82.96	44.33	140.32	69
		2006	5,800	2,799	10,289	46	81.49	39.32	144.56	72
		2007	5,706	2,462	10,598	46	80.21	34.60	148.98	72
	Female	1992	4,097	2,252	5,972	39	55.25	30.37	80.54	69
		1993	3,406	1,858	5,615	39	45.87	25.03	75.62	77
		1994	3,783	2,276	5,316	40	50.88	30.61	71.51	79
		1995	3,248	1,964	5,062	41	43.60	26.37	67.95	81
		1996	3,210	2,040	4,863	43	43.00	27.33	65.15	82
		1997	3,179	2,121	4,672	44	42.62	28.44	62.65	81
		1998	3,182	2,217	4,567	44	42.69	29.75	61.28	81
		1999	3,482	2,586	4,469	41	46.79	34.74	60.06	83
		2000	3,246	2,352	4,561	43	43.65	31.62	61.33	79
		2001	3,617	2,861	4,666	43	48.60	38.44	62.69	80
		2002	3,371	2,325	4,954	43	45.25	31.21	66.50	78
		2003	3,409	2,172	5,245	44	45.76	29.16	70.40	77
		2004	3,436	2,015	5,546	44	46.07	27.02	74.38	79
		2005	3,451	1,844	5,838	44	46.31	24.75	78.32	79
		2006	3,447	1,663	6,114	42	46.33	22.36	82.20	78
		2007	3,430	1,480	6,372	44	46.18	19.93	85.78	78

Metropolitan Statistical Area	PWID Population	Year	Number	Min	Max	Rank	Rate	Min	Max	Rank
Cleveland--Lorain--Elyria, OH	Young (15-29)	1992	1,935	1,063	2,820	57	43.06	23.67	62.77	84
		1993	1,631	890	2,689	60	36.85	20.11	60.75	86
		1994	1,842	1,108	2,588	58	42.23	25.41	59.34	84
		1995	1,613	975	2,513	64	37.30	22.56	58.14	87
		1996	1,630	1,036	2,469	63	37.96	24.12	57.51	86
		1997	1,656	1,105	2,434	65	38.92	25.97	57.20	87
		1998	1,707	1,190	2,451	63	40.49	28.22	58.13	87
		1999	1,931	1,434	2,479	62	46.50	34.53	59.68	85
		2000	1,869	1,354	2,626	62	45.58	33.02	64.04	85
		2001	2,170	1,717	2,800	60	53.68	42.46	69.25	83
		2002	2,118	1,461	3,112	61	52.60	36.28	77.30	82
		2003	2,251	1,435	3,464	62	55.82	35.57	85.87	83
		2004	2,396	1,405	3,867	59	59.09	34.66	95.39	84
		2005	2,552	1,364	4,316	57	62.68	33.49	106.01	82
		2006	2,712	1,309	4,812	54	66.45	32.06	117.88	82
		2007	2,885	1,245	5,358	53	70.41	30.38	130.77	76
	Old (30-64)	1992	10,432	5,734	15,207	34	105.64	58.07	154.00	58
		1993	8,805	4,804	14,515	37	88.26	48.15	145.49	67
		1994	9,847	5,924	13,839	37	97.82	58.85	137.47	62
		1995	8,453	5,112	13,174	38	83.30	50.38	129.83	71
		1996	8,293	5,270	12,565	37	81.21	51.61	123.05	71
		1997	8,105	5,407	11,912	37	79.13	52.79	116.30	69
		1998	7,960	5,548	11,427	37	77.44	53.97	111.16	68
		1999	8,507	6,317	10,919	37	82.37	61.16	105.73	65
		2000	7,710	5,586	10,833	37	74.27	53.81	104.36	64
		2001	8,319	6,580	10,731	38	79.56	62.93	102.64	63
		2002	7,484	5,162	10,998	38	71.25	49.14	104.70	63
		2003	7,280	4,639	11,200	38	69.22	44.11	106.49	64
		2004	7,034	4,126	11,356	38	66.91	39.24	108.01	64
		2005	6,751	3,607	11,418	37	64.26	34.34	108.69	66
		2006	6,409	3,093	11,371	38	61.19	29.53	108.56	69
		2007	6,027	2,600	11,194	43	57.70	24.90	107.18	68

Metropolitan Statistical Area	PWID Population	Year	Number	Min	Max	Rank	Rate	Min	Max	Rank
Columbus, OH	Total	1992	9,090	4,622	15,191	50	94.87	48.24	158.54	58
		1993	7,871	4,458	14,733	50	81.05	45.90	151.71	65
		1994	8,905	4,793	14,251	50	90.69	48.82	145.15	61
		1995	7,726	4,562	13,777	50	77.72	45.90	138.59	68
		1996	7,585	4,578	13,299	50	75.65	45.66	132.65	67
		1997	7,390	4,099	12,853	54	72.86	40.42	126.71	68
		1998	7,196	3,556	12,432	56	69.89	34.54	120.75	68
		1999	7,761	3,078	11,979	53	74.37	29.50	114.79	66
		2000	6,979	2,832	11,907	57	65.85	26.72	112.35	70
		2001	7,733	2,901	11,802	53	72.01	27.02	109.90	68
		2002	7,125	3,208	11,925	52	65.67	29.57	109.91	72
		2003	7,295	3,648	12,068	53	66.45	33.23	109.92	72
		2004	7,465	4,061	12,259	52	67.34	36.63	110.57	70
		2005	7,575	4,223	12,468	52	67.57	37.68	111.23	71
		2006	7,606	4,001	12,745	52	67.08	35.29	112.41	71
		2007	7,616	3,732	13,013	51	66.48	32.58	113.60	71
	Non-Hispanic White	1992	4,495	2,285	7,511	53	55.31	28.12	92.43	71
		1993	3,830	2,169	7,169	56	46.71	26.46	87.44	73
		1994	4,279	2,303	6,848	53	51.84	27.90	82.96	73
		1995	3,682	2,174	6,565	59	44.25	26.13	78.91	77
		1996	3,601	2,173	6,314	60	43.14	26.04	75.64	77
		1997	3,513	1,949	6,111	60	41.79	23.19	72.69	80
		1998	3,446	1,703	5,953	62	40.55	20.04	70.06	80
		1999	3,766	1,494	5,813	59	43.95	17.43	67.84	78
		2000	3,453	1,401	5,892	59	39.91	16.19	68.09	81
		2001	3,926	1,473	5,992	58	45.04	16.90	68.75	81
		2002	3,735	1,682	6,252	58	42.64	19.20	71.36	81
		2003	3,972	1,986	6,570	54	45.05	22.53	74.52	81
		2004	4,242	2,307	6,966	48	47.86	26.04	78.60	78
		2005	4,511	2,515	7,425	47	50.56	28.19	83.22	76
		2006	4,761	2,505	7,978	45	53.00	27.88	88.81	76
		2007	5,019	2,460	8,577	46	55.51	27.21	94.86	74

Metropolitan Statistical Area	PWID Population	Year	Number	Min	Max	Rank	Rate	Min	Max	Rank
Columbus, OH	Non-Hispanic Black	1992	4,153	2,111	6,940	26	359.59	182.84	600.92	36
		1993	3,617	2,049	6,771	27	304.30	172.35	569.59	43
		1994	4,096	2,205	6,556	26	336.65	181.21	538.79	36
		1995	3,539	2,090	6,310	27	283.19	167.23	504.97	42
		1996	3,440	2,077	6,033	26	269.33	162.56	472.26	41
		1997	3,301	1,831	5,741	25	252.73	140.21	439.57	41
		1998	3,146	1,555	5,435	25	235.34	116.31	406.59	39
		1999	3,299	1,308	5,092	24	241.50	95.78	372.76	32
		2000	2,864	1,162	4,887	25	204.25	82.87	348.49	40
		2001	3,041	1,141	4,642	24	211.11	79.21	322.21	33
		2002	2,664	1,200	4,459	25	180.67	81.36	302.38	41
		2003	2,571	1,286	4,254	28	169.95	84.99	281.14	42
		2004	2,459	1,338	4,039	28	159.00	86.49	261.09	48
		2005	2,313	1,289	3,807	32	146.25	81.54	240.73	53
		2006	2,135	1,123	3,577	32	132.01	69.44	221.21	58
		2007	1,953	957	3,336	36	118.37	58.01	202.26	64
	Hispanic	1992	131	67	220	72	137.91	70.12	230.46	49
		1993	113	64	211	74	107.30	60.77	200.84	53
		1994	127	68	203	75	111.14	59.83	177.88	52
		1995	110	65	196	76	87.50	51.67	156.03	55
		1996	107	65	188	78	78.12	47.15	136.98	58
		1997	104	58	181	78	69.22	38.40	120.38	61
		1998	101	50	174	77	61.14	30.22	105.64	63
		1999	108	43	167	77	59.46	23.58	91.78	66
		2000	96	39	164	80	47.95	19.46	81.81	68
		2001	105	39	160	79	47.72	17.90	72.83	71
		2002	95	43	158	81	40.39	18.19	67.59	73
		2003	95	47	156	82	37.74	18.87	62.43	73
		2004	94	51	154	84	35.40	19.26	58.13	74
		2005	92	51	152	85	33.02	18.41	54.36	76
		2006	89	47	150	87	30.25	15.91	50.69	79
		2007	86	42	146	87	27.68	13.56	47.29	83

Metropolitan Statistical Area	PWID Population	Year	Number	Min	Max	Rank	Rate	Min	Max	Rank
Columbus, OH	Male	1992	5,673	2,884	9,480	52	119.02	60.52	198.91	61
		1993	4,878	2,763	9,130	51	100.94	57.17	188.94	68
		1994	5,490	2,955	8,787	51	112.42	60.51	179.92	64
		1995	4,748	2,804	8,466	52	95.96	56.67	171.11	72
		1996	4,651	2,807	8,156	56	93.21	56.26	163.43	73
		1997	4,527	2,511	7,874	58	89.73	49.78	156.07	73
		1998	4,407	2,178	7,614	57	86.05	42.53	148.67	74
		1999	4,753	1,885	7,337	56	91.57	36.32	141.34	72
		2000	4,275	1,734	7,293	59	81.04	32.88	138.27	72
		2001	4,735	1,777	7,227	53	88.56	33.23	135.17	72
		2002	4,360	1,963	7,297	57	80.70	36.34	135.06	71
		2003	4,455	2,228	7,371	55	81.39	40.70	134.64	72
		2004	4,545	2,472	7,464	55	82.18	44.70	134.95	71
		2005	4,590	2,559	7,555	56	81.99	45.72	134.97	72
		2006	4,577	2,408	7,670	56	80.73	42.47	135.28	73
		2007	4,540	2,225	7,757	56	79.16	38.80	135.26	73
	Female	1992	3,535	1,797	5,908	41	73.41	37.33	122.67	47
		1993	3,092	1,751	5,788	44	63.38	35.89	118.63	55
		1994	3,524	1,897	5,641	43	71.43	38.45	114.32	52
		1995	3,074	1,815	5,482	46	61.57	36.36	109.79	57
		1996	3,028	1,828	5,310	48	60.14	36.30	105.46	59
		1997	2,956	1,640	5,142	48	57.99	32.17	100.85	60
		1998	2,881	1,424	4,977	49	55.67	27.52	96.19	66
		1999	3,107	1,232	4,795	51	59.24	23.50	91.44	62
		2000	2,792	1,133	4,764	52	52.45	21.28	89.48	67
		2001	3,091	1,160	4,718	51	57.34	21.51	87.52	65
		2002	2,847	1,282	4,765	51	52.26	23.53	87.47	68
		2003	2,914	1,457	4,821	49	52.95	26.48	87.59	67
		2004	2,985	1,624	4,901	51	53.72	29.22	88.22	66
		2005	3,034	1,692	4,995	48	54.07	30.14	88.99	66
		2006	3,058	1,608	5,124	48	53.94	28.37	90.38	65
		2007	3,078	1,509	5,260	51	53.81	26.37	91.95	64

Metropolitan Statistical Area	PWID Population	Year	Number	Min	Max	Rank	Rate	Min	Max	Rank
Columbus, OH	Young (15-29)	1992	1,837	934	3,070	60	53.03	26.96	88.62	76
		1993	1,601	907	2,997	62	46.45	26.31	86.94	77
		1994	1,845	993	2,952	57	53.76	28.94	86.04	72
		1995	1,648	973	2,938	62	47.96	28.32	85.52	77
		1996	1,681	1,015	2,947	62	48.91	29.52	85.76	72
		1997	1,717	952	2,986	62	49.68	27.56	86.40	73
		1998	1,765	872	3,050	62	50.73	25.07	87.64	76
		1999	2,023	802	3,122	61	57.94	22.98	89.43	76
		2000	1,942	788	3,314	60	55.39	22.47	94.50	75
		2001	2,307	866	3,521	57	65.81	24.69	100.45	74
		2002	2,284	1,029	3,823	55	65.10	29.31	108.95	73
		2003	2,517	1,259	4,163	51	71.12	35.57	117.65	69
		2004	2,772	1,508	4,551	48	77.89	42.37	127.90	65
		2005	3,023	1,685	4,975	45	84.60	47.17	139.26	61
		2006	3,255	1,712	5,454	42	90.72	47.72	152.02	56
		2007	3,483	1,707	5,952	40	96.73	47.41	165.28	48
	Old (30-64)	1992	7,422	3,774	12,403	44	121.31	61.68	202.72	48
		1993	6,370	3,608	11,924	44	101.69	57.60	190.35	54
		1994	7,143	3,845	11,433	45	111.85	60.21	179.00	51
		1995	6,141	3,626	10,950	47	94.39	55.74	168.32	61
		1996	5,967	3,602	10,463	48	90.56	54.66	158.79	60
		1997	5,745	3,187	9,992	49	85.90	47.66	149.41	62
		1998	5,516	2,726	9,530	52	80.93	39.99	139.81	63
		1999	5,847	2,319	9,025	52	84.20	33.40	129.97	63
		2000	5,148	2,089	8,783	57	72.59	29.45	123.86	66
		2001	5,557	2,085	8,481	54	76.83	28.83	117.26	66
		2002	4,958	2,233	8,298	55	67.54	30.42	113.05	67
		2003	4,878	2,439	8,069	54	65.57	32.79	108.46	66
		2004	4,751	2,584	7,802	54	63.11	34.33	103.64	71
		2005	4,533	2,527	7,461	57	59.35	33.09	97.70	74
		2006	4,214	2,217	7,062	59	54.37	28.60	91.11	75
		2007	3,833	1,878	6,549	63	48.80	23.91	83.38	79

Metropolitan Statistical Area	PWID Population	Year	Number	Min	Max	Rank	Rate	Min	Max	Rank
Dallas, TX	Total	1992	25,683	10,798	49,824	11	133.37	56.07	258.73	42
		1993	21,586	8,575	49,320	18	109.56	43.53	250.34	47
		1994	25,988	11,292	48,159	10	128.69	55.92	238.47	40
		1995	22,383	11,455	46,740	16	107.76	55.15	225.03	46
		1996	22,142	11,949	42,850	16	103.54	55.88	200.38	48
		1997	21,702	12,361	37,943	16	98.03	55.84	171.39	49
		1998	21,348	12,865	33,819	18	93.25	56.20	147.72	51
		1999	24,165	13,358	33,316	11	102.31	56.55	141.05	49
		2000	22,700	12,810	36,715	16	93.30	52.65	150.90	53
		2001	28,578	14,151	42,085	10	113.63	56.26	167.33	44
		2002	25,599	9,507	47,895	13	99.55	36.97	186.25	44
		2003	26,977	8,509	53,179	11	103.04	32.50	203.11	42
		2004	28,026	8,020	56,538	10	105.06	30.07	211.95	40
		2005	28,707	7,560	58,146	10	105.26	27.72	213.20	40
		2006	29,550	7,145	60,087	9	105.38	25.48	214.27	38
		2007	30,216	6,718	61,499	9	105.27	23.40	214.26	36
	Non-Hispanic White	1992	16,030	6,740	31,098	7	125.53	52.78	243.53	25
		1993	12,998	5,164	29,697	12	101.17	40.19	231.15	35
		1994	15,265	6,633	28,288	7	117.86	51.21	218.42	25
		1995	12,953	6,629	27,048	11	98.99	50.66	206.70	34
		1996	12,726	6,867	24,627	11	96.25	51.94	186.28	36
		1997	12,461	7,098	21,786	12	92.73	52.82	162.12	39
		1998	12,291	7,407	19,471	12	90.02	54.25	142.61	40
		1999	13,970	7,723	19,260	8	100.99	55.83	139.24	31
		2000	13,168	7,431	21,297	9	93.97	53.03	151.99	35
		2001	16,586	8,212	24,425	6	116.45	57.66	171.49	22
		2002	14,791	5,493	27,674	8	103.13	38.30	192.96	29
		2003	15,409	4,860	30,375	8	107.07	33.77	211.06	28
		2004	15,676	4,486	31,623	8	108.36	31.01	218.59	27
		2005	15,533	4,091	31,462	9	106.64	28.08	215.99	28
		2006	15,231	3,683	30,971	11	103.56	25.04	210.58	28
		2007	14,551	3,235	29,615	12	98.08	21.81	199.63	37

Metropolitan Statistical Area	PWID Population	Year	Number	Min	Max	Rank	Rate	Min	Max	Rank
Dallas, TX	Non-Hispanic Black	1992	6,736	2,832	13,068	15	232.38	97.70	450.82	66
		1993	6,032	2,396	13,782	17	203.14	80.70	464.15	63
		1994	7,492	3,255	13,884	14	245.50	106.67	454.95	58
		1995	6,491	3,322	13,555	14	206.17	105.51	430.52	56
		1996	6,335	3,419	12,260	15	195.88	105.70	379.07	56
		1997	6,041	3,441	10,562	15	180.90	103.04	316.27	60
		1998	5,732	3,454	9,080	16	166.75	100.49	264.16	63
		1999	6,239	3,449	8,602	15	177.04	97.87	244.08	60
		2000	5,652	3,190	9,142	16	157.12	88.67	254.13	60
		2001	6,927	3,430	10,201	12	186.26	92.23	274.30	48
		2002	6,135	2,279	11,479	13	161.44	59.96	302.04	52
		2003	6,535	2,061	12,883	13	168.78	53.24	332.72	43
		2004	7,056	2,019	14,234	13	178.39	51.05	359.87	38
		2005	7,756	2,043	15,710	11	191.10	50.33	387.08	36
		2006	8,868	2,144	18,032	9	208.46	50.40	423.88	29
		2007	10,404	2,313	21,175	9	238.81	53.09	486.06	21
	Hispanic	1992	1,631	686	3,165	36	55.66	23.40	107.99	82
		1993	1,423	565	3,251	37	44.91	17.84	102.62	86
		1994	1,774	771	3,287	36	51.93	22.56	96.23	81
		1995	1,580	808	3,299	36	42.73	21.87	89.23	84
		1996	1,613	870	3,122	36	40.18	21.68	77.76	83
		1997	1,630	928	2,849	36	37.30	21.25	65.21	83
		1998	1,650	995	2,614	36	34.85	21.00	55.21	82
		1999	1,922	1,062	2,649	36	37.62	20.80	51.87	81
		2000	1,856	1,048	3,002	35	33.86	19.11	54.76	80
		2001	2,403	1,190	3,539	31	41.03	20.32	60.42	76
		2002	2,216	823	4,146	32	35.91	13.34	67.19	75
		2003	2,407	759	4,745	27	37.25	11.75	73.44	74
		2004	2,584	739	5,212	27	38.30	10.96	77.26	71
		2005	2,744	723	5,558	27	38.80	10.22	78.59	71
		2006	2,943	712	5,984	26	39.60	9.57	80.52	71
		2007	3,155	701	6,420	25	40.51	9.01	82.44	68

Metropolitan Statistical Area	PWID Population	Year	Number	Min	Max	Rank	Rate	Min	Max	Rank
Dallas, TX	Male	1992	16,103	6,770	31,239	13	167.19	70.29	324.34	41
		1993	13,093	5,201	29,915	22	132.76	52.74	303.34	51
		1994	15,353	6,671	28,451	12	151.57	65.86	280.88	43
		1995	12,965	6,635	27,074	18	124.46	63.69	259.89	51
		1996	12,652	6,827	24,484	18	117.82	63.58	228.00	54
		1997	12,297	7,004	21,499	19	110.43	62.90	193.08	57
		1998	12,047	7,260	19,084	19	104.47	62.96	165.49	59
		1999	13,623	7,531	18,782	14	114.33	63.20	157.63	60
		2000	12,810	7,229	20,719	19	104.21	58.81	168.55	59
		2001	16,156	8,000	23,792	12	126.86	62.81	186.82	49
		2002	14,491	5,382	27,113	14	111.10	41.26	207.87	57
		2003	15,267	4,816	30,096	14	114.87	36.23	226.44	51
		2004	15,813	4,525	31,899	14	116.64	33.38	235.30	51
		2005	16,082	4,235	32,574	15	115.81	30.50	234.58	51
		2006	16,349	3,953	33,244	15	114.49	27.68	232.80	51
		2007	16,397	3,645	33,374	15	112.03	24.91	228.01	51
	Female	1992	9,877	4,153	19,161	11	102.61	43.14	199.06	34
		1993	8,722	3,465	19,930	17	88.65	35.22	202.55	33
		1994	10,918	4,744	20,233	8	108.48	47.13	201.02	24
		1995	9,691	4,959	20,236	12	93.60	47.90	195.45	32
		1996	9,805	5,291	18,975	9	92.10	49.70	178.24	31
		1997	9,767	5,563	17,076	9	88.76	50.56	155.19	32
		1998	9,714	5,854	15,389	12	85.49	51.52	135.43	36
		1999	11,071	6,120	15,263	8	94.58	52.29	130.40	28
		2000	10,436	5,889	16,879	11	86.69	48.92	140.20	34
		2001	13,152	6,512	19,368	7	105.93	52.45	156.00	21
		2002	11,775	4,373	22,030	7	92.92	34.51	173.85	24
		2003	12,393	3,909	24,430	6	96.14	30.32	189.51	22
		2004	12,860	3,680	25,942	6	98.02	28.05	197.75	22
		2005	13,170	3,468	26,675	7	98.38	25.91	199.26	22
		2006	13,578	3,283	27,610	7	98.66	23.86	200.62	20
		2007	13,944	3,100	28,381	7	99.13	22.04	201.77	20

Metropolitan Statistical Area	PWID Population	Year	Number	Min	Max	Rank	Rate	Min	Max	Rank
Dallas, TX	Young (15-29)	1992	9,210	3,872	17,867	5	135.25	56.86	262.38	24
		1993	7,520	2,987	17,182	12	110.22	43.79	251.84	29
		1994	8,842	3,842	16,386	6	128.45	55.81	238.03	23
		1995	7,477	3,826	15,614	8	106.66	54.58	222.72	36
		1996	7,299	3,939	14,124	8	101.37	54.70	196.17	40
		1997	7,093	4,040	12,401	9	95.17	54.21	166.40	43
		1998	6,949	4,188	11,009	11	89.97	54.22	142.54	46
		1999	7,869	4,350	10,849	8	98.71	54.57	136.09	41
		2000	7,424	4,190	12,007	9	90.68	51.18	146.67	49
		2001	9,421	4,665	13,874	8	112.72	55.81	166.00	38
		2002	8,535	3,170	15,968	9	101.17	37.57	189.28	47
		2003	9,123	2,878	17,984	9	107.73	33.98	212.35	46
		2004	9,639	2,758	19,445	9	113.04	32.35	228.03	40
		2005	10,063	2,650	20,382	9	116.43	30.66	235.84	35
		2006	10,576	2,557	21,505	9	120.31	29.09	244.64	32
		2007	11,057	2,458	22,505	8	123.99	27.57	252.37	34
	Old (30-64)	1992	16,686	7,015	32,371	16	134.05	56.36	260.06	43
		1993	14,221	5,650	32,494	22	110.42	43.87	252.30	47
		1994	17,328	7,529	32,111	15	130.18	56.57	241.24	42
		1995	15,078	7,716	31,486	19	109.57	56.07	228.81	48
		1996	15,043	8,118	29,111	18	106.06	57.23	205.24	49
		1997	14,844	8,455	25,952	18	101.07	57.57	176.71	52
		1998	14,674	8,843	23,245	18	96.73	58.29	153.23	56
		1999	16,661	9,210	22,971	15	106.47	58.86	146.79	51
		2000	15,671	8,844	25,346	17	97.07	54.78	156.99	54
		2001	19,719	9,764	29,038	14	117.42	58.14	172.92	39
		2002	17,625	6,546	32,976	15	102.00	37.88	190.84	44
		2003	18,505	5,837	36,478	14	104.47	32.95	205.94	39
		2004	19,124	5,473	38,580	12	105.38	30.16	212.58	36
		2005	19,456	5,124	39,407	12	104.43	27.50	211.52	34
		2006	19,854	4,800	40,371	11	103.13	24.94	209.70	33
		2007	20,076	4,463	40,861	11	101.47	22.56	206.52	31

Metropolitan Statistical Area	PWID Population	Year	Number	Min	Max	Rank	Rate	Min	Max	Rank
Dayton--Springfield, OH	Total	1992	4,078	2,455	5,759	83	64.11	38.60	90.53	88
		1993	3,448	2,034	5,342	83	54.17	31.96	83.93	90
		1994	3,756	2,707	4,940	85	59.23	42.68	77.89	88
		1995	3,284	2,290	4,563	85	51.70	36.06	71.85	91
		1996	3,238	2,425	4,243	86	51.05	38.23	66.89	90
		1997	3,178	2,557	3,908	86	50.37	40.53	61.93	90
		1998	3,203	2,701	3,916	86	50.78	42.83	62.09	89
		1999	3,361	2,766	3,912	88	53.48	44.01	62.24	92
		2000	3,328	2,620	4,180	88	53.06	41.78	66.64	85
		2001	3,481	2,482	4,446	88	55.61	39.65	71.03	89
		2002	3,606	2,357	4,962	88	57.58	37.64	79.24	78
		2003	3,826	2,236	5,473	88	61.13	35.73	87.44	76
		2004	4,105	2,126	6,000	88	65.47	33.91	95.72	72
		2005	4,468	2,018	6,519	86	71.27	32.19	103.99	68
		2006	4,831	1,911	7,022	80	77.21	30.54	112.23	63
		2007	5,197	1,801	7,506	78	83.40	28.90	120.46	58
	Non-Hispanic White	1992	2,079	1,252	2,936	81	38.57	23.22	54.47	88
		1993	1,647	972	2,552	88	30.63	18.07	47.46	92
		1994	1,712	1,233	2,251	88	32.02	23.07	42.11	96
		1995	1,453	1,014	2,020	92	27.23	18.99	37.84	97
		1996	1,417	1,061	1,857	93	26.67	19.97	34.94	97
		1997	1,399	1,126	1,720	93	26.53	21.35	32.62	96
		1998	1,440	1,214	1,760	93	27.38	23.09	33.47	95
		1999	1,562	1,286	1,818	89	29.90	24.61	34.80	94
		2000	1,616	1,272	2,030	91	31.10	24.48	39.05	93
		2001	1,779	1,269	2,273	89	34.37	24.50	43.89	94
		2002	1,950	1,275	2,683	86	37.72	24.66	51.91	86
		2003	2,194	1,282	3,138	82	42.55	24.87	60.86	83
		2004	2,495	1,292	3,647	80	48.39	25.07	70.75	76
		2005	2,873	1,298	4,192	77	55.80	25.21	81.43	69
		2006	3,275	1,296	4,761	74	63.84	25.25	92.80	63
		2007	3,698	1,281	5,341	65	72.49	25.12	104.70	56

Metropolitan Statistical Area	PWID Population	Year	Number	Min	Max	Rank	Rate	Min	Max	Rank
Dayton--Springfield, OH	Non-Hispanic Black	1992	1,804	1,086	2,548	59	217.49	130.95	307.15	71
		1993	1,642	969	2,544	57	195.53	115.36	302.91	66
		1994	1,874	1,350	2,464	56	221.88	159.88	291.79	65
		1995	1,678	1,170	2,332	56	196.13	136.81	272.58	64
		1996	1,663	1,245	2,179	53	192.85	144.42	252.69	58
		1997	1,613	1,298	1,983	55	186.47	150.05	229.29	56
		1998	1,581	1,334	1,933	56	181.46	153.03	221.84	56
		1999	1,590	1,308	1,850	55	181.70	149.52	211.46	58
		2000	1,485	1,169	1,865	56	168.29	132.51	211.36	54
		2001	1,445	1,030	1,845	58	163.54	116.61	208.88	61
		2002	1,373	897	1,889	57	154.42	100.94	212.48	55
		2003	1,319	771	1,887	58	147.91	86.45	211.57	56
		2004	1,268	657	1,854	59	141.69	73.39	207.13	57
		2005	1,228	555	1,792	62	136.72	61.76	199.50	57
		2006	1,178	466	1,712	63	130.73	51.72	190.03	59
		2007	1,127	391	1,628	61	125.06	43.34	180.63	61
	Hispanic	1992	12	7	17	100	22.34	13.45	31.56	98
		1993	10	6	16	100	18.11	10.68	28.06	98
		1994	11	8	15	100	19.14	13.79	25.17	97
		1995	10	7	13	100	15.96	11.13	22.17	98
		1996	10	7	12	100	15.09	11.30	19.77	98
		1997	9	8	11	100	14.31	11.52	17.60	98
		1998	9	8	12	100	13.85	11.68	16.93	98
		1999	10	8	12	100	13.98	11.50	16.27	97
		2000	10	8	13	100	13.29	10.46	16.69	97
		2001	11	8	14	100	13.62	9.71	17.39	97
		2002	11	7	16	100	13.83	9.04	19.04	95
		2003	13	7	18	99	14.71	8.60	21.04	95
		2004	15	8	21	99	15.98	8.28	23.35	95
		2005	17	8	25	99	18.04	8.15	26.33	94
		2006	20	8	30	98	20.90	8.27	30.37	92
		2007	25	9	36	97	24.89	8.63	35.96	88

Metropolitan Statistical Area	PWID Population	Year	Number	Min	Max	Rank	Rate	Min	Max	Rank
Dayton--Springfield, OH	Male	1992	2,557	1,540	3,612	84	82.39	49.61	116.36	89
		1993	2,116	1,248	3,278	86	68.09	40.18	105.49	93
		1994	2,264	1,631	2,977	86	73.13	52.69	96.17	93
		1995	1,950	1,360	2,711	86	62.88	43.86	87.38	94
		1996	1,902	1,424	2,492	87	61.39	45.97	80.44	94
		1997	1,851	1,490	2,276	87	60.06	48.33	73.85	94
		1998	1,855	1,564	2,268	87	60.22	50.79	73.62	93
		1999	1,938	1,595	2,256	87	63.12	51.95	73.46	94
		2000	1,914	1,507	2,404	87	62.44	49.16	78.41	91
		2001	1,997	1,424	2,551	87	65.27	46.54	83.36	94
		2002	2,065	1,350	2,842	87	67.42	44.07	92.78	85
		2003	2,185	1,277	3,126	87	71.32	41.68	102.01	81
		2004	2,336	1,210	3,415	87	76.03	39.38	111.15	76
		2005	2,529	1,142	3,690	87	82.22	37.14	119.98	71
		2006	2,713	1,073	3,943	86	88.34	34.95	128.41	65
		2007	2,886	1,000	4,169	83	94.37	32.70	136.30	63
	Female	1992	1,584	954	2,236	76	48.61	29.27	68.65	76
		1993	1,385	817	2,146	80	42.52	25.09	65.87	83
		1994	1,550	1,117	2,038	82	47.74	34.40	62.78	81
		1995	1,383	965	1,922	83	42.57	29.69	59.16	84
		1996	1,385	1,038	1,815	83	42.70	31.98	55.95	83
		1997	1,375	1,106	1,691	84	42.60	34.28	52.38	82
		1998	1,396	1,177	1,707	85	43.26	36.48	52.89	80
		1999	1,472	1,211	1,713	86	45.79	37.68	53.29	85
		2000	1,460	1,150	1,834	85	45.53	35.85	57.19	75
		2001	1,528	1,090	1,952	86	47.78	34.07	61.02	82
		2002	1,582	1,034	2,177	83	49.45	32.32	68.05	72
		2003	1,677	980	2,399	82	52.50	30.69	75.10	68
		2004	1,798	931	2,629	81	56.24	29.13	82.22	63
		2005	1,957	884	2,856	80	61.30	27.69	89.44	57
		2006	2,120	839	3,082	72	66.55	26.33	96.73	46
		2007	2,290	793	3,307	67	72.18	25.01	104.25	43

Metropolitan Statistical Area	PWID Population	Year	Number	Min	Max	Rank	Rate	Min	Max	Rank
Dayton--Springfield, OH	Young (15-29)	1992	802	483	1,133	88	37.85	22.79	53.46	92
		1993	535	316	829	95	25.61	15.11	39.67	99
		1994	499	359	656	96	24.26	17.48	31.91	99
		1995	403	281	560	96	19.75	13.78	27.45	100
		1996	394	295	516	96	19.46	14.58	25.50	100
		1997	407	327	500	96	20.31	16.34	24.97	100
		1998	452	381	553	95	22.69	19.13	27.73	100
		1999	543	447	632	95	27.56	22.68	32.07	100
		2000	629	495	790	95	32.25	25.39	40.51	99
		2001	777	554	993	95	40.40	28.81	51.60	96
		2002	950	621	1,308	93	49.43	32.31	68.02	85
		2003	1,175	687	1,681	89	61.11	35.72	87.40	80
		2004	1,439	745	2,104	85	74.43	38.55	108.81	71
		2005	1,741	787	2,541	76	89.55	40.45	130.67	55
		2006	2,030	803	2,950	73	104.18	41.21	151.43	43
		2007	2,275	789	3,286	62	117.15	40.60	169.21	36
	Old (30-64)	1992	3,367	2,027	4,754	79	79.35	47.78	112.07	85
		1993	2,970	1,752	4,601	82	69.49	41.00	107.66	84
		1994	3,310	2,385	4,352	80	77.21	55.64	101.54	84
		1995	2,922	2,038	4,061	82	67.78	47.28	94.19	83
		1996	2,885	2,161	3,781	84	66.80	50.03	87.53	83
		1997	2,814	2,265	3,461	85	65.33	52.57	80.33	79
		1998	2,798	2,360	3,421	85	64.88	54.71	79.32	78
		1999	2,873	2,364	3,344	85	66.59	54.80	77.50	80
		2000	2,757	2,171	3,463	85	63.79	50.23	80.12	76
		2001	2,765	1,971	3,531	85	63.78	45.48	81.47	79
		2002	2,714	1,774	3,734	84	62.53	40.87	86.04	73
		2003	2,696	1,576	3,857	84	62.18	36.34	88.94	73
		2004	2,682	1,389	3,921	83	61.87	32.05	90.44	72
		2005	2,692	1,216	3,929	83	62.26	28.12	90.85	69
		2006	2,687	1,063	3,906	83	62.36	24.67	90.65	66
		2007	2,694	934	3,891	82	62.81	21.77	90.72	65

Metropolitan Statistical Area	PWID Population	Year	Number	Min	Max	Rank	Rate	Min	Max	Rank
Denver, CO	Total	1992	21,546	15,905	26,329	21	182.60	134.80	223.14	20
		1993	20,467	16,063	24,871	19	167.31	131.30	203.31	23
		1994	20,006	16,053	23,142	22	159.29	127.82	184.26	24
		1995	19,432	16,075	21,345	20	150.40	124.41	165.20	28
		1996	18,862	16,066	20,925	20	142.17	121.10	157.72	31
		1997	18,214	16,060	21,304	20	133.68	117.87	156.36	32
		1998	17,775	15,986	21,598	21	127.56	114.73	155.00	33
		1999	15,878	15,004	16,619	27	110.74	104.65	115.91	43
		2000	17,267	14,308	21,891	21	118.37	98.08	150.07	39
		2001	15,665	13,667	17,527	26	104.89	91.51	117.36	47
		2002	16,650	13,081	19,074	21	110.01	86.43	126.02	39
		2003	16,289	12,400	20,100	21	106.86	81.35	131.87	40
		2004	15,976	11,840	21,440	23	103.70	76.86	139.17	41
		2005	15,638	11,291	22,855	24	100.23	72.37	146.48	42
		2006	15,402	10,803	24,554	23	97.04	68.06	154.69	45
		2007	15,141	9,356	26,353	24	93.59	57.84	162.91	48
	Non-Hispanic White	1992	10,419	7,692	12,733	21	113.19	83.56	138.33	31
		1993	9,827	7,712	11,942	21	103.84	81.49	126.19	34
		1994	9,621	7,720	11,129	21	99.73	80.03	115.36	37
		1995	9,438	7,807	10,367	22	95.91	79.34	105.35	36
		1996	9,323	7,941	10,343	23	93.16	79.35	103.34	40
		1997	9,226	8,135	10,792	22	90.64	79.91	106.01	41
		1998	9,284	8,350	11,281	23	90.12	81.05	109.51	39
		1999	8,596	8,123	8,997	22	81.99	77.48	85.81	47
		2000	9,729	8,062	12,334	19	92.33	76.51	117.06	40
		2001	9,215	8,040	10,310	22	86.17	75.18	96.41	40
		2002	10,246	8,049	11,737	19	95.39	74.95	109.28	35
		2003	10,494	7,989	12,949	19	97.78	74.43	120.66	33
		2004	10,773	7,984	14,458	18	100.07	74.16	134.29	33
		2005	11,026	7,961	16,114	18	101.76	73.47	148.72	33
		2006	11,331	7,947	18,064	17	103.48	72.58	164.97	29
		2007	11,591	7,163	20,175	17	104.45	64.54	181.79	28

Metropolitan Statistical Area	PWID Population	Year	Number	Min	Max	Rank	Rate	Min	Max	Rank
Denver, CO	Non-Hispanic Black	1992	3,137	2,316	3,834	34	473.29	349.39	578.37	18
		1993	2,758	2,165	3,352	35	402.66	316.01	489.31	24
		1994	2,534	2,033	2,931	41	362.26	290.68	419.03	26
		1995	2,344	1,939	2,575	38	329.16	272.29	361.56	28
		1996	2,195	1,869	2,435	40	301.03	256.41	333.95	29
		1997	2,067	1,822	2,418	42	276.44	243.74	323.33	31
		1998	1,987	1,787	2,414	42	259.02	232.96	314.73	28
		1999	1,762	1,665	1,845	48	223.75	211.44	234.19	41
		2000	1,917	1,588	2,430	42	238.30	197.46	302.11	29
		2001	1,748	1,525	1,955	48	211.81	184.80	236.99	31
		2002	1,874	1,472	2,146	41	222.83	175.07	255.27	26
		2003	1,853	1,410	2,286	40	219.53	167.12	270.90	26
		2004	1,838	1,362	2,467	37	212.00	157.12	284.51	27
		2005	1,820	1,314	2,660	36	208.46	150.51	304.66	28
		2006	1,812	1,271	2,889	38	202.33	141.90	322.54	33
		2007	1,801	1,113	3,135	43	197.17	121.85	343.19	37
	Hispanic	1992	5,737	4,235	7,011	12	363.95	268.67	444.76	12
		1993	5,213	4,091	6,335	14	306.74	240.73	372.75	15
		1994	4,885	3,920	5,651	14	270.98	217.44	313.45	18
		1995	4,559	3,771	5,007	16	236.62	195.74	259.91	20
		1996	4,261	3,629	4,726	17	206.62	175.99	229.21	22
		1997	3,969	3,500	4,643	17	180.44	159.10	211.05	26
		1998	3,745	3,368	4,551	17	160.41	144.26	194.91	27
		1999	3,241	3,063	3,392	21	129.78	122.64	135.84	34
		2000	3,421	2,835	4,337	20	129.28	107.12	163.89	33
		2001	3,019	2,634	3,378	25	108.24	94.43	121.11	40
		2002	3,128	2,457	3,583	24	107.65	84.57	123.32	38
		2003	2,990	2,276	3,690	25	99.54	75.77	122.83	42
		2004	2,873	2,129	3,856	26	92.88	68.84	124.65	43
		2005	2,765	1,996	4,041	26	86.38	62.37	126.24	43
		2006	2,687	1,885	4,284	27	81.06	56.85	129.23	47
		2007	2,619	1,619	4,559	27	76.26	47.12	132.73	49

Metropolitan Statistical Area	PWID Population	Year	Number	Min	Max	Rank	Rate	Min	Max	Rank
Denver, CO	Male	1992	14,459	10,674	17,670	20	245.17	180.99	299.61	19
		1993	13,356	10,482	16,231	20	218.05	171.13	264.97	25
		1994	12,788	10,261	14,792	21	203.20	163.05	235.04	29
		1995	12,253	10,136	13,459	22	188.81	156.19	207.39	33
		1996	11,810	10,059	13,101	23	176.98	150.74	196.33	33
		1997	11,389	10,042	13,321	23	165.86	146.24	194.00	35
		1998	11,153	10,030	13,551	22	158.45	142.50	192.53	36
		1999	10,032	9,480	10,500	27	138.18	130.57	144.63	42
		2000	11,011	9,124	13,960	22	148.69	123.20	188.50	40
		2001	10,094	8,807	11,294	26	132.92	115.97	148.72	47
		2002	10,843	8,519	12,421	21	140.79	110.61	161.28	39
		2003	10,711	8,153	13,217	20	138.29	105.27	170.64	40
		2004	10,586	7,846	14,207	20	134.91	99.99	181.06	40
		2005	10,415	7,520	15,221	20	131.24	94.76	191.80	40
		2006	10,271	7,204	16,374	22	127.12	89.16	202.65	42
		2007	10,064	6,219	17,516	23	122.16	75.49	212.63	43
	Female	1992	6,490	4,791	7,931	24	109.96	81.17	134.38	27
		1993	6,555	5,145	7,966	21	107.33	84.23	130.43	24
		1994	6,717	5,390	7,770	20	107.20	86.02	123.99	26
		1995	6,755	5,588	7,420	21	105.04	86.89	115.38	23
		1996	6,717	5,722	7,452	21	101.87	86.77	113.01	23
		1997	6,584	5,805	7,701	21	97.43	85.90	113.95	25
		1998	6,471	5,820	7,863	21	93.84	84.40	114.03	26
		1999	5,782	5,464	6,052	24	81.70	77.20	85.51	45
		2000	6,254	5,182	7,929	21	87.08	72.16	110.40	33
		2001	5,617	4,901	6,285	25	76.52	66.76	85.62	48
		2002	5,889	4,627	6,747	22	79.23	62.25	90.76	38
		2003	5,668	4,315	6,994	24	75.60	57.55	93.28	40
		2004	5,460	4,047	7,327	25	72.24	53.54	96.95	43
		2005	5,247	3,788	7,668	26	68.43	49.41	100.01	47
		2006	5,076	3,560	8,092	27	65.14	45.69	103.84	51
		2007	4,911	3,035	8,548	29	61.86	38.23	107.66	51

Metropolitan Statistical Area	PWID Population	Year	Number	Min	Max	Rank	Rate	Min	Max	Rank
Denver, CO	Young (15-29)	1992	5,301	3,913	6,478	21	146.06	107.83	178.50	18
		1993	5,149	4,041	6,257	19	139.29	109.32	169.27	21
		1994	5,177	4,154	5,989	21	138.50	111.14	160.21	18
		1995	5,192	4,295	5,703	19	135.24	111.87	148.55	19
		1996	5,212	4,439	5,782	18	131.37	111.89	145.73	22
		1997	5,202	4,587	6,084	18	126.34	111.40	147.77	26
		1998	5,235	4,708	6,361	17	122.86	110.49	149.28	29
		1999	4,801	4,537	5,025	20	108.79	102.80	113.87	36
		2000	5,329	4,416	6,756	19	118.19	97.93	149.84	33
		2001	4,896	4,271	5,478	23	107.64	93.91	120.44	39
		2002	5,219	4,100	5,978	23	114.79	90.18	131.50	39
		2003	5,062	3,853	6,246	23	112.06	85.30	138.27	43
		2004	4,856	3,599	6,517	24	107.04	79.33	143.65	46
		2005	4,577	3,305	6,690	28	100.62	72.65	147.06	52
		2006	4,262	2,989	6,795	31	92.60	64.95	147.62	54
		2007	3,878	2,397	6,751	36	82.93	51.25	144.34	57
	Old (30-64)	1992	15,893	11,733	19,422	19	194.53	143.60	237.72	23
		1993	15,025	11,792	18,258	20	176.01	138.13	213.89	26
		1994	14,599	11,715	16,887	20	165.50	132.80	191.43	32
		1995	14,084	11,650	15,470	20	155.08	128.29	170.35	29
		1996	13,569	11,558	15,053	22	145.90	124.27	161.86	32
		1997	12,999	11,462	15,204	23	136.72	120.55	159.92	35
		1998	12,582	11,316	15,288	24	130.07	116.98	158.05	37
		1999	11,148	10,535	11,668	28	112.34	106.15	117.58	46
		2000	12,030	9,968	15,252	23	119.36	98.90	151.32	39
		2001	10,841	9,458	12,129	27	104.37	91.06	116.77	49
		2002	11,462	9,005	13,130	23	108.25	85.04	124.00	39
		2003	11,178	8,509	13,793	23	104.22	79.34	128.60	40
		2004	10,957	8,121	14,705	22	100.82	74.72	135.30	41
		2005	10,753	7,764	15,715	23	97.28	70.24	142.17	42
		2006	10,651	7,470	16,979	24	94.51	66.28	150.66	40
		2007	10,559	6,525	18,379	23	91.82	56.74	159.82	37

Metropolitan Statistical Area	PWID Population	Year	Number	Min	Max	Rank	Rate	Min	Max	Rank
Detroit, MI	Total	1992	23,345	10,293	35,424	17	81.86	36.09	124.21	69
		1993	24,029	11,831	34,676	16	84.11	41.41	121.37	61
		1994	23,119	13,367	33,985	15	80.64	46.62	118.54	71
		1995	25,225	14,965	33,418	14	87.38	51.84	115.75	57
		1996	25,917	16,459	34,009	13	89.08	56.57	116.89	55
		1997	26,407	17,851	36,169	11	90.68	61.30	124.21	54
		1998	27,020	18,223	38,124	10	92.85	62.62	131.02	52
		1999	23,518	18,617	30,647	13	80.78	63.95	105.27	63
		2000	27,608	17,469	39,390	10	94.58	59.85	134.94	51
		2001	23,936	16,374	29,488	13	81.49	55.74	100.39	60
		2002	27,901	14,916	39,329	10	94.60	50.58	133.35	50
		2003	27,868	13,456	38,663	10	94.04	45.41	130.47	49
		2004	27,568	11,923	37,464	11	92.60	40.05	125.83	50
		2005	27,248	10,375	36,160	11	91.09	34.69	120.89	51
		2006	26,860	8,850	37,157	14	89.58	29.52	123.92	54
		2007	26,289	7,273	38,942	14	88.00	24.35	130.35	54
	Non-Hispanic White	1992	9,951	4,388	15,100	22	46.91	20.69	71.19	79
		1993	10,309	5,076	14,876	19	48.72	23.99	70.31	69
		1994	9,904	5,727	14,559	20	46.86	27.09	68.88	78
		1995	10,726	6,364	14,210	16	50.66	30.06	67.11	67
		1996	10,891	6,917	14,292	16	51.27	32.56	67.28	67
		1997	10,938	7,394	14,981	15	51.67	34.93	70.78	67
		1998	11,021	7,433	15,550	15	52.35	35.31	73.87	66
		1999	9,453	7,483	12,319	19	45.15	35.74	58.84	76
		2000	10,966	6,939	15,646	16	52.50	33.22	74.90	69
		2001	9,438	6,456	11,627	19	45.09	30.85	55.55	80
		2002	10,990	5,876	15,492	18	52.54	28.09	74.06	71
		2003	11,056	5,339	15,340	18	52.86	25.52	73.33	69
		2004	11,123	4,810	15,116	17	53.17	23.00	72.26	69
		2005	11,300	4,303	14,996	16	54.03	20.57	71.70	71
		2006	11,579	3,815	16,017	16	55.49	18.28	76.76	69
		2007	11,910	3,295	17,642	15	57.51	15.91	85.19	68

Metropolitan Statistical Area	PWID Population	Year	Number	Min	Max	Rank	Rate	Min	Max	Rank
Detroit, MI	Non-Hispanic Black	1992	12,458	5,493	18,904	7	202.10	89.11	306.67	75
		1993	12,519	6,164	18,067	7	201.37	99.14	290.59	64
		1994	11,848	6,850	17,416	8	188.64	109.07	277.31	73
		1995	12,796	7,592	16,952	8	201.06	119.28	266.36	60
		1996	13,081	8,307	17,166	8	203.55	129.27	267.11	53
		1997	13,314	9,000	18,235	8	206.29	139.45	282.55	52
		1998	13,642	9,200	19,248	8	210.56	142.01	297.09	46
		1999	11,904	9,424	15,513	8	182.67	144.61	238.05	56
		2000	14,008	8,864	19,986	7	214.06	135.45	305.41	37
		2001	12,155	8,315	14,975	8	184.12	125.95	226.83	51
		2002	14,140	7,560	19,932	4	212.44	113.58	299.46	31
		2003	14,036	6,777	19,474	4	208.95	100.89	289.89	29
		2004	13,724	5,935	18,650	4	202.12	87.41	274.66	29
		2005	13,315	5,070	17,671	6	193.71	73.76	257.07	33
		2006	12,779	4,210	17,678	6	183.99	60.62	254.52	38
		2007	12,063	3,337	17,868	7	173.05	47.88	256.34	45
	Hispanic	1992	447	197	678	56	76.12	33.56	115.51	66
		1993	464	229	670	52	76.35	37.59	110.18	64
		1994	445	257	654	55	70.30	40.65	103.35	72
		1995	478	283	633	53	71.95	42.69	95.32	61
		1996	479	304	629	53	68.07	43.23	89.33	62
		1997	475	321	650	55	64.72	43.75	88.65	64
		1998	471	318	664	56	61.64	41.57	86.97	61
		1999	398	315	518	58	50.07	39.63	65.24	73
		2000	455	288	649	57	54.81	34.68	78.20	64
		2001	387	265	477	60	44.59	30.50	54.93	72
		2002	448	240	632	59	49.55	26.49	69.85	64
		2003	451	218	625	59	48.06	23.21	66.68	65
		2004	458	198	622	59	47.20	20.41	64.14	65
		2005	475	181	630	59	47.33	18.02	62.82	66
		2006	504	166	697	60	48.94	16.12	67.70	65
		2007	549	152	813	60	52.37	14.49	77.58	62

Metropolitan Statistical Area	PWID Population	Year	Number	Min	Max	Rank	Rate	Min	Max	Rank
Detroit, MI	Male	1992	15,185	6,696	23,042	16	109.22	48.16	165.73	69
		1993	15,751	7,755	22,731	13	112.88	55.58	162.89	60
		1994	15,199	8,788	22,343	14	108.38	62.67	159.32	71
		1995	16,566	9,828	21,947	13	117.11	69.48	155.14	57
		1996	16,948	10,763	22,241	12	118.72	75.39	155.79	51
		1997	17,154	11,596	23,495	12	119.91	81.06	164.24	49
		1998	17,404	11,738	24,557	12	121.63	82.03	171.62	49
		1999	15,006	11,879	19,556	12	104.68	82.87	136.41	62
		2000	17,448	11,041	24,895	12	121.32	76.77	173.10	51
		2001	14,994	10,257	18,472	14	103.50	70.80	127.51	63
		2002	17,350	9,276	24,457	12	119.20	63.73	168.03	48
		2003	17,248	8,328	23,929	13	117.79	56.88	163.42	48
		2004	17,041	7,370	23,157	13	115.79	50.08	157.35	53
		2005	16,894	6,433	22,420	13	114.18	43.48	151.53	53
		2006	16,792	5,533	23,229	14	113.16	37.28	156.54	52
		2007	16,667	4,611	24,689	14	112.72	31.19	166.97	49
	Female	1992	8,139	3,589	12,350	17	55.69	24.55	84.50	67
		1993	8,410	4,141	12,136	18	57.54	28.33	83.03	62
		1994	8,156	4,716	11,989	16	55.69	32.20	81.86	71
		1995	8,998	5,338	11,920	15	61.12	36.26	80.97	58
		1996	9,367	5,948	12,291	14	63.21	40.14	82.95	56
		1997	9,679	6,543	13,257	11	65.33	44.17	89.49	56
		1998	10,044	6,774	14,172	8	67.92	45.80	95.83	53
		1999	8,858	7,012	11,544	12	59.95	47.45	78.12	61
		2000	10,517	6,655	15,005	9	71.02	44.94	101.33	49
		2001	9,196	6,291	11,329	11	61.77	42.26	76.10	59
		2002	10,771	5,758	15,182	9	72.10	38.55	101.64	46
		2003	10,760	5,196	14,929	9	71.77	34.66	99.57	44
		2004	10,588	4,579	14,389	9	70.33	30.42	95.57	48
		2005	10,342	3,938	13,725	11	68.42	26.05	90.80	48
		2006	9,999	3,294	13,832	11	66.03	21.75	91.34	48
		2007	9,514	2,632	14,093	12	63.06	17.45	93.40	49

Metropolitan Statistical Area	PWID Population	Year	Number	Min	Max	Rank	Rate	Min	Max	Rank
Detroit, MI	Young (15-29)	1992	3,508	1,547	5,323	36	37.70	16.62	57.20	93
		1993	3,521	1,733	5,080	29	38.47	18.94	55.51	85
		1994	3,331	1,926	4,897	34	36.91	21.34	54.25	92
		1995	3,608	2,141	4,780	28	40.18	23.84	53.23	84
		1996	3,718	2,361	4,880	26	41.55	26.39	54.53	84
		1997	3,842	2,597	5,263	25	43.34	29.30	59.36	83
		1998	4,034	2,721	5,692	25	46.01	31.03	64.92	80
		1999	3,647	2,887	4,753	29	42.14	33.36	54.92	89
		2000	4,506	2,851	6,429	23	52.64	33.31	75.10	78
		2001	4,167	2,850	5,133	28	49.39	33.79	60.85	85
		2002	5,251	2,807	7,402	21	62.97	33.66	88.76	77
		2003	5,748	2,775	7,974	19	69.03	33.33	95.76	73
		2004	6,313	2,730	8,579	17	75.86	32.81	103.08	67
		2005	7,011	2,670	9,305	16	83.76	31.90	111.16	63
		2006	7,847	2,585	10,855	13	93.28	30.73	129.04	53
		2007	8,789	2,432	13,019	12	104.79	28.99	155.22	41
	Old (30-64)	1992	20,079	8,853	30,469	11	104.51	46.08	158.58	60
		1993	20,764	10,223	29,965	13	106.93	52.65	154.31	53
		1994	20,045	11,590	29,466	11	102.04	59.00	150.00	59
		1995	21,914	13,001	29,031	9	110.19	65.37	145.97	47
		1996	22,521	14,302	29,553	8	111.79	70.99	146.70	46
		1997	22,907	15,485	31,375	8	113.09	76.45	154.90	44
		1998	23,341	15,742	32,933	8	114.80	77.43	161.98	42
		1999	20,172	15,968	26,287	12	98.60	78.05	128.49	55
		2000	23,427	14,824	33,426	11	113.56	71.86	162.03	40
		2001	20,006	13,685	24,645	12	95.55	65.36	117.71	52
		2002	22,838	12,210	32,193	9	107.96	57.72	152.18	40
		2003	22,179	10,709	30,770	9	104.09	50.26	144.41	41
		2004	21,128	9,137	28,711	9	98.50	42.60	133.85	44
		2005	19,847	7,557	26,339	11	92.13	35.08	122.27	43
		2006	18,265	6,018	25,266	12	84.67	27.90	117.13	48
		2007	16,284	4,505	24,122	14	75.78	20.97	112.26	53

Metropolitan Statistical Area	PWID Population	Year	Number	Min	Max	Rank	Rate	Min	Max	Rank
El Paso, TX	Total	1992	13,765	11,925	17,071	35	345.18	299.02	428.07	1
		1993	11,099	4,805	16,633	38	272.08	117.78	407.75	4
		1994	12,526	10,434	16,080	36	302.24	251.76	388.00	2
		1995	10,050	4,753	15,443	42	239.65	113.33	368.24	5
		1996	9,434	4,666	14,685	44	224.77	111.17	349.86	6
		1997	8,937	4,620	14,078	45	210.43	108.79	331.48	7
		1998	8,436	4,555	13,484	46	197.05	106.40	314.95	8
		1999	9,064	6,298	12,828	46	210.86	146.50	298.42	6
		2000	7,490	4,389	12,409	51	172.92	101.33	286.50	9
		2001	7,986	5,142	11,967	49	183.22	117.97	274.57	10
		2002	6,539	3,790	11,514	63	149.10	86.43	262.54	18
		2003	6,080	3,489	11,067	67	137.63	78.98	250.51	22
		2004	5,651	3,198	10,694	73	126.53	71.61	239.46	27
		2005	5,204	2,854	10,294	76	115.46	63.32	228.41	31
		2006	4,796	2,526	9,987	81	104.51	55.04	217.63	40
		2007	4,325	2,126	9,583	84	93.36	45.90	206.86	49
	Non-Hispanic White	1992	2,129	1,844	2,640	79	219.19	189.87	271.82	5
		1993	1,752	759	2,626	83	182.45	78.98	273.43	7
		1994	1,979	1,648	2,540	83	212.11	176.68	272.29	5
		1995	1,561	738	2,399	88	171.82	81.25	264.02	6
		1996	1,419	702	2,208	92	162.70	80.47	253.25	6
		1997	1,284	664	2,023	94	151.45	78.29	238.57	11
		1998	1,145	618	1,830	96	139.23	75.18	222.54	13
		1999	1,152	801	1,631	98	145.21	100.88	205.51	9
		2000	886	519	1,468	100	115.32	67.58	191.07	21
		2001	876	564	1,312	100	116.37	74.93	174.39	23
		2002	664	385	1,169	100	90.52	52.47	159.39	38
		2003	572	328	1,042	100	80.96	46.46	147.37	43
		2004	496	281	938	100	70.98	40.17	134.33	54
		2005	429	235	848	100	62.92	34.51	124.48	65
		2006	375	198	782	100	54.80	28.86	114.12	71
		2007	326	160	723	100	48.93	24.06	108.41	79

Metropolitan Statistical Area	PWID Population	Year	Number	Min	Max	Rank	Rate	Min	Max	Rank
El Paso, TX	Non-Hispanic Black	1992	772	669	957	85	549.62	476.12	681.59	13
		1993	549	237	822	91	387.58	167.78	580.86	29
		1994	569	474	730	88	415.28	345.92	533.11	20
		1995	434	205	667	92	318.02	150.39	488.65	32
		1996	400	198	623	93	297.69	147.24	463.36	32
		1997	382	198	602	92	284.99	147.33	448.94	27
		1998	371	200	593	92	278.21	150.22	444.68	25
		1999	417	289	590	89	314.46	218.48	445.05	16
		2000	363	213	602	92	276.76	162.18	458.54	20
		2001	411	265	616	90	318.95	205.36	477.97	12
		2002	357	207	628	92	277.16	160.66	488.02	13
		2003	350	201	636	92	281.36	161.47	512.13	14
		2004	339	192	641	92	268.08	151.71	507.33	16
		2005	321	176	634	92	253.76	139.17	502.00	17
		2006	298	157	620	94	222.38	117.11	463.11	23
		2007	265	130	588	97	200.27	98.46	443.73	35
	Hispanic	1992	10,516	9,110	13,041	6	373.52	323.57	463.21	11
		1993	8,379	3,627	12,558	7	287.55	124.48	430.93	16
		1994	9,378	7,812	12,039	5	311.55	259.51	399.94	13
		1995	7,488	3,541	11,506	8	242.89	114.86	373.21	19
		1996	7,019	3,472	10,925	11	224.63	111.10	349.64	18
		1997	6,660	3,443	10,491	11	208.24	107.65	328.03	19
		1998	6,315	3,410	10,093	11	193.82	104.66	309.80	20
		1999	6,832	4,747	9,669	9	206.73	143.62	292.57	18
		2000	5,696	3,338	9,437	11	169.30	99.21	280.50	20
		2001	6,136	3,951	9,196	13	180.05	115.93	269.82	19
		2002	5,082	2,946	8,949	15	147.22	85.34	259.23	22
		2003	4,783	2,745	8,706	16	136.12	78.12	247.77	25
		2004	4,501	2,547	8,517	16	126.20	71.42	238.83	27
		2005	4,195	2,301	8,299	16	115.82	63.52	229.12	32
		2006	3,911	2,060	8,145	17	105.98	55.81	220.70	34
		2007	3,565	1,753	7,899	22	95.00	46.71	210.49	39

Metropolitan Statistical Area	PWID Population	Year	Number	Min	Max	Rank	Rate	Min	Max	Rank
El Paso, TX	Male	1992	10,831	9,382	13,431	31	561.22	486.16	695.98	1
		1993	8,728	3,778	13,080	33	441.62	191.17	661.83	1
		1994	9,826	8,185	12,615	31	491.26	409.21	630.65	1
		1995	7,850	3,712	12,062	35	388.32	183.63	596.68	2
		1996	7,322	3,621	11,396	36	362.12	179.11	563.65	2
		1997	6,876	3,555	10,832	37	336.26	173.84	529.70	2
		1998	6,420	3,467	10,262	39	311.58	168.24	498.02	4
		1999	6,806	4,729	9,632	38	328.78	228.42	465.31	3
		2000	5,533	3,242	9,167	45	265.26	155.44	439.49	7
		2001	5,786	3,725	8,670	47	275.83	177.60	413.36	7
		2002	4,630	2,684	8,152	51	219.41	127.19	386.34	12
		2003	4,190	2,405	7,627	59	197.60	113.40	359.67	17
		2004	3,773	2,135	7,141	66	175.90	99.54	332.88	21
		2005	3,350	1,837	6,626	75	154.77	84.88	306.18	29
		2006	2,958	1,558	6,161	85	134.25	70.70	279.57	37
		2007	2,540	1,249	5,628	86	114.18	56.14	252.98	48
	Female	1992	3,112	2,695	3,859	47	151.19	130.97	187.49	9
		1993	2,471	1,070	3,703	58	117.50	50.86	176.09	22
		1994	2,793	2,327	3,586	56	130.27	108.51	167.23	17
		1995	2,279	1,078	3,501	62	104.90	49.60	161.18	24
		1996	2,203	1,090	3,429	64	101.26	50.08	157.61	24
		1997	2,172	1,123	3,422	65	98.64	50.99	155.38	24
		1998	2,152	1,162	3,440	67	96.91	52.33	154.90	22
		1999	2,442	1,697	3,456	63	109.58	76.13	155.08	18
		2000	2,139	1,254	3,545	69	95.28	55.83	157.85	24
		2001	2,423	1,560	3,631	64	107.16	69.00	160.59	20
		2002	2,107	1,221	3,710	71	92.61	53.68	163.06	25
		2003	2,077	1,192	3,781	73	90.43	51.89	164.59	26
		2004	2,040	1,154	3,860	75	87.90	49.74	166.35	28
		2005	1,975	1,083	3,908	78	84.33	46.25	166.82	27
		2006	1,903	1,002	3,964	79	79.80	42.02	166.17	34
		2007	1,782	876	3,949	80	74.01	36.39	163.98	37

Metropolitan Statistical Area	PWID Population	Year	Number	Min	Max	Rank	Rate	Min	Max	Rank
El Paso, TX	Young (15-29)	1992	5,094	4,412	6,317	22	323.75	280.45	401.49	1
		1993	3,823	1,655	5,729	27	241.08	104.36	361.30	2
		1994	4,060	3,382	5,212	25	257.70	214.66	330.81	1
		1995	3,102	1,467	4,767	32	196.72	93.03	302.28	5
		1996	2,807	1,388	4,369	36	178.83	88.45	278.36	6
		1997	2,594	1,341	4,086	39	164.07	84.82	258.46	8
		1998	2,417	1,305	3,864	44	152.29	82.23	243.41	14
		1999	2,594	1,802	3,672	47	163.75	113.77	231.75	15
		2000	2,165	1,269	3,587	55	136.79	80.15	226.63	24
		2001	2,356	1,517	3,530	55	149.09	95.99	223.42	22
		2002	1,987	1,152	3,499	65	126.41	73.28	222.58	29
		2003	1,920	1,102	3,495	70	122.49	70.30	222.96	31
		2004	1,869	1,058	3,537	75	118.36	66.98	223.99	35
		2005	1,814	995	3,588	75	114.32	62.69	226.14	37
		2006	1,770	932	3,687	77	109.37	57.60	227.76	40
		2007	1,697	835	3,761	81	103.88	51.08	230.18	42
	Old (30-64)	1992	8,760	7,588	10,863	38	362.78	314.27	449.89	1
		1993	7,279	3,151	10,909	42	291.89	126.36	437.44	5
		1994	8,447	7,037	10,844	41	328.84	273.92	422.14	2
		1995	6,950	3,287	10,679	42	265.58	125.59	408.09	6
		1996	6,667	3,298	10,377	43	253.70	125.48	394.88	6
		1997	6,428	3,323	10,126	46	241.10	124.64	379.80	7
		1998	6,149	3,320	9,828	46	228.24	123.24	364.81	7
		1999	6,661	4,628	9,427	45	245.40	170.49	347.30	5
		2000	5,520	3,234	9,145	53	200.81	117.67	332.71	8
		2001	5,866	3,777	8,791	51	211.13	135.94	316.39	7
		2002	4,755	2,757	8,373	57	169.03	97.98	297.62	11
		2003	4,342	2,492	7,903	64	152.35	87.43	277.31	15
		2004	3,923	2,220	7,425	65	135.91	76.91	257.20	20
		2005	3,467	1,901	6,859	76	118.73	65.11	234.88	27
		2006	3,014	1,587	6,277	78	101.48	53.45	211.34	34
		2007	2,506	1,232	5,552	85	83.56	41.08	185.14	45

Metropolitan Statistical Area	PWID Population	Year	Number	Min	Max	Rank	Rate	Min	Max	Rank
Fort Lauderdale, FL	Total	1992	9,119	6,482	12,521	49	111.64	79.36	153.28	48
		1993	7,422	2,884	11,768	55	87.86	34.15	139.32	57
		1994	8,759	6,352	10,940	52	100.47	72.86	125.49	50
		1995	7,226	3,059	10,127	56	80.49	34.07	112.80	66
		1996	7,321	3,149	9,693	54	79.29	34.11	104.98	65
		1997	7,592	3,271	10,105	50	79.37	34.20	105.65	65
		1998	7,872	3,388	10,228	49	79.70	34.30	103.54	64
		1999	9,504	7,479	11,226	44	93.59	73.65	110.55	55
		2000	7,807	3,507	11,830	48	74.53	33.48	112.94	66
		2001	8,655	6,411	11,482	48	80.34	59.51	106.57	61
		2002	6,970	3,765	10,383	55	63.16	34.12	94.09	76
		2003	6,609	3,867	8,812	60	58.86	34.44	78.48	78
		2004	6,472	3,946	9,672	60	56.61	34.51	84.60	82
		2005	6,581	4,035	11,137	63	56.48	34.63	95.57	82
		2006	6,638	4,062	12,508	64	56.77	34.74	106.98	82
		2007	6,664	3,588	13,753	63	57.42	30.92	118.52	83
	Non-Hispanic White	1992	6,265	4,454	8,602	36	110.24	78.37	151.36	35
		1993	5,139	1,997	8,148	41	89.58	34.81	142.04	38
		1994	6,030	4,373	7,531	37	104.29	75.63	130.26	33
		1995	4,894	2,072	6,858	43	84.38	35.72	118.25	46
		1996	4,837	2,080	6,404	43	83.31	35.83	110.30	48
		1997	4,862	2,095	6,472	40	83.05	35.79	110.55	48
		1998	4,869	2,095	6,326	37	82.71	35.59	107.45	48
		1999	5,673	4,464	6,701	36	96.54	75.97	114.03	33
		2000	4,513	2,027	6,838	42	76.47	34.36	115.87	51
		2001	4,881	3,616	6,475	43	82.77	61.31	109.81	45
		2002	3,883	2,098	5,785	54	66.13	35.72	98.52	59
		2003	3,698	2,163	4,930	58	63.60	37.21	84.79	63
		2004	3,709	2,261	5,543	62	64.42	39.27	96.27	63
		2005	3,944	2,418	6,674	58	68.99	42.30	116.75	55
		2006	4,241	2,595	7,991	57	75.75	46.35	142.74	52
		2007	4,606	2,480	9,506	55	84.79	45.65	175.00	44

Metropolitan Statistical Area	PWID Population	Year	Number	Min	Max	Rank	Rate	Min	Max	Rank
Fort Lauderdale, FL	Non-Hispanic Black	1992	2,252	1,601	3,092	49	162.01	115.16	222.43	85
		1993	1,647	640	2,612	56	110.20	42.83	174.74	93
		1994	1,846	1,339	2,306	58	115.23	83.57	143.92	94
		1995	1,515	641	2,124	58	88.63	37.52	124.20	94
		1996	1,583	681	2,096	56	87.17	37.49	115.41	94
		1997	1,733	747	2,307	52	89.04	38.37	118.52	93
		1998	1,920	826	2,495	45	92.61	39.85	120.31	91
		1999	2,479	1,951	2,929	36	112.94	88.87	133.40	84
		2000	2,157	969	3,268	35	93.59	42.04	141.81	87
		2001	2,483	1,840	3,294	32	102.94	76.25	136.56	83
		2002	2,018	1,090	3,006	37	80.12	43.28	119.36	89
		2003	1,858	1,087	2,477	39	71.18	41.64	94.91	91
		2004	1,680	1,025	2,511	43	61.93	37.76	92.56	91
		2005	1,481	908	2,507	52	52.66	32.28	89.10	94
		2006	1,195	731	2,253	62	41.61	25.46	78.41	99
		2007	870	468	1,795	72	30.10	16.20	62.11	99
	Hispanic	1992	550	391	755	51	59.39	42.21	81.54	78
		1993	464	180	736	52	45.20	17.56	71.66	85
		1994	573	415	716	51	50.83	36.86	63.49	82
		1995	497	210	696	52	40.07	16.96	56.16	85
		1996	531	228	703	52	38.96	16.76	51.57	84
		1997	582	251	775	52	38.98	16.80	51.88	81
		1998	638	275	829	52	39.28	16.90	51.03	79
		1999	813	640	961	45	45.98	36.18	54.31	76
		2000	704	316	1,066	51	36.43	16.37	55.21	77
		2001	818	606	1,086	48	38.86	28.79	51.56	78
		2002	688	371	1,024	54	30.34	16.39	45.21	82
		2003	676	396	902	54	28.08	16.43	37.44	83
		2004	682	416	1,019	54	26.77	16.32	40.01	84
		2005	709	435	1,200	54	26.34	16.15	44.57	85
		2006	725	444	1,366	55	26.05	15.94	49.10	84
		2007	731	394	1,510	55	25.71	13.84	53.06	86

Metropolitan Statistical Area	PWID Population	Year	Number	Min	Max	Rank	Rate	Min	Max	Rank
Fort Lauderdale, FL	Male	1992	5,957	4,235	8,179	48	147.46	104.82	202.46	47
		1993	4,857	1,888	7,702	52	116.28	45.19	184.38	57
		1994	5,725	4,152	7,150	48	132.89	96.37	165.97	51
		1995	4,704	1,991	6,592	53	106.10	44.91	148.69	64
		1996	4,736	2,037	6,270	52	103.91	44.69	137.57	64
		1997	4,872	2,100	6,486	51	103.20	44.47	137.37	64
		1998	5,007	2,155	6,505	49	102.78	44.23	133.52	62
		1999	5,986	4,711	7,071	44	119.61	94.12	141.28	54
		2000	4,870	2,188	7,379	49	94.39	42.40	143.02	66
		2001	5,350	3,963	7,097	49	100.76	74.64	133.66	64
		2002	4,274	2,309	6,367	59	78.59	42.46	117.09	74
		2003	4,029	2,357	5,371	63	72.76	42.56	97.01	79
		2004	3,931	2,397	5,876	64	69.71	42.50	104.19	84
		2005	3,997	2,451	6,764	62	69.54	42.63	117.66	84
		2006	4,047	2,476	7,625	62	70.16	42.93	132.21	83
		2007	4,096	2,205	8,453	63	71.51	38.50	147.58	80
	Female	1992	3,139	2,232	4,310	46	76.04	54.05	104.40	45
		1993	2,553	992	4,048	56	59.78	23.23	94.80	58
		1994	3,023	2,192	3,776	48	68.55	49.71	85.62	56
		1995	2,511	1,063	3,520	56	55.27	23.39	77.45	64
		1996	2,570	1,105	3,402	54	54.97	23.64	72.77	63
		1997	2,697	1,162	3,590	53	55.67	23.99	74.11	63
		1998	2,834	1,220	3,682	51	56.61	24.36	73.55	63
		1999	3,469	2,730	4,098	42	67.37	53.01	79.57	56
		2000	2,890	1,298	4,380	49	54.38	24.43	82.39	66
		2001	3,247	2,405	4,307	46	59.42	44.02	78.83	63
		2002	2,646	1,429	3,942	56	47.27	25.54	70.42	76
		2003	2,534	1,483	3,379	62	44.53	26.05	59.37	82
		2004	2,500	1,524	3,737	63	43.15	26.31	64.49	82
		2005	2,553	1,565	4,321	62	43.25	26.51	73.18	83
		2006	2,577	1,577	4,855	60	43.50	26.61	81.96	81
		2007	2,577	1,387	5,318	60	43.85	23.61	90.51	81

Metropolitan Statistical Area	PWID Population	Year	Number	Min	Max	Rank	Rate	Min	Max	Rank
Fort Lauderdale, FL	Young (15-29)	1992	1,810	1,287	2,485	61	73.39	52.17	100.77	60
		1993	1,436	558	2,277	71	57.69	22.42	91.47	64
		1994	1,701	1,234	2,125	65	67.84	49.20	84.73	62
		1995	1,444	611	2,023	69	56.98	24.12	79.85	65
		1996	1,534	660	2,031	67	59.65	25.66	78.97	64
		1997	1,693	729	2,253	64	64.00	27.58	85.19	60
		1998	1,885	811	2,449	60	69.30	29.82	90.04	59
		1999	2,452	1,930	2,897	49	88.07	69.31	104.03	52
		2000	2,170	975	3,288	53	76.07	34.17	115.26	58
		2001	2,578	1,909	3,419	48	88.79	65.77	117.79	57
		2002	2,204	1,191	3,284	58	74.64	40.32	111.20	66
		2003	2,192	1,283	2,923	64	73.18	42.81	97.57	68
		2004	2,217	1,351	3,313	65	72.62	44.27	108.52	75
		2005	2,284	1,400	3,865	62	73.27	44.92	123.99	74
		2006	2,283	1,397	4,301	65	73.15	44.76	137.85	71
		2007	2,212	1,191	4,565	64	71.78	38.65	148.15	72
	Old (30-64)	1992	7,377	5,244	10,129	45	129.38	91.97	177.64	46
		1993	6,026	2,342	9,555	49	101.14	39.31	160.36	56
		1994	7,093	5,143	8,858	46	114.20	82.81	142.62	47
		1995	5,804	2,457	8,134	52	90.07	38.13	126.22	66
		1996	5,804	2,497	7,684	51	87.14	37.48	115.36	65
		1997	5,913	2,548	7,871	47	85.45	36.82	113.74	63
		1998	5,998	2,581	7,792	47	83.79	36.06	108.85	61
		1999	7,055	5,552	8,334	42	95.73	75.33	113.07	58
		2000	5,632	2,530	8,533	49	73.88	33.19	111.95	65
		2001	6,058	4,487	8,036	47	76.96	57.01	102.10	65
		2002	4,736	2,559	7,056	59	58.60	31.66	87.30	79
		2003	4,374	2,559	5,831	63	53.13	31.08	70.83	82
		2004	4,195	2,558	6,270	63	50.06	30.52	74.81	86
		2005	4,215	2,584	7,132	62	49.38	30.27	83.56	85
		2006	4,246	2,598	8,001	58	49.54	30.31	93.35	83
		2007	4,311	2,321	8,898	56	50.58	27.23	104.40	77

Metropolitan Statistical Area	PWID Population	Year	Number	Min	Max	Rank	Rate	Min	Max	Rank
Fort Worth--Arlington, TX	Total	1992	23,902	22,297	26,911	14	249.44	232.69	280.85	5
		1993	19,746	8,579	25,987	22	203.48	88.40	267.80	14
		1994	23,252	21,780	25,280	14	234.29	219.47	254.74	6
		1995	19,477	8,807	24,544	19	191.78	86.72	241.68	11
		1996	19,316	8,909	23,763	19	186.03	85.80	228.87	10
		1997	19,243	9,049	23,444	19	180.31	84.79	219.68	10
		1998	19,105	9,193	23,423	19	174.11	83.78	213.46	10
		1999	22,168	21,348	23,398	14	196.36	189.10	207.25	8
		2000	18,853	9,162	22,831	20	162.49	78.97	196.78	11
		2001	21,922	21,184	22,429	16	184.37	178.16	188.63	9
		2002	18,324	8,244	23,726	19	150.35	67.65	194.68	17
		2003	17,921	7,706	24,958	19	144.14	61.98	200.73	17
		2004	17,366	7,134	26,177	19	137.05	56.30	206.58	21
		2005	16,810	6,505	27,488	20	129.91	50.27	212.42	24
		2006	16,359	5,884	29,274	21	122.96	44.23	220.04	27
		2007	15,840	5,161	31,112	23	115.91	37.77	227.65	28
	Non-Hispanic White	1992	14,576	13,597	16,411	9	203.56	189.89	229.19	9
		1993	11,848	5,147	15,593	15	165.46	71.88	217.76	12
		1994	13,835	12,960	15,043	9	191.37	179.26	208.07	11
		1995	11,567	5,231	14,577	14	158.50	71.67	199.73	11
		1996	11,507	5,307	14,157	14	156.45	72.15	192.47	11
		1997	11,538	5,426	14,057	14	154.81	72.80	188.60	8
		1998	11,552	5,559	14,163	14	152.80	73.53	187.33	6
		1999	13,524	13,024	14,275	9	176.42	169.90	186.21	6
		2000	11,595	5,635	14,041	14	149.41	72.61	180.94	8
		2001	13,562	13,106	13,876	11	173.16	167.33	177.16	6
		2002	11,365	5,113	14,716	17	143.56	64.59	185.88	12
		2003	11,094	4,770	15,449	17	139.07	59.80	193.67	13
		2004	10,666	4,382	16,078	19	132.66	54.50	199.96	13
		2005	10,170	3,935	16,631	20	125.28	48.48	204.86	16
		2006	9,663	3,476	17,293	21	117.61	42.31	210.47	21
		2007	9,039	2,945	17,753	22	108.63	35.39	213.35	26

Metropolitan Statistical Area	PWID Population	Year	Number	Min	Max	Rank	Rate	Min	Max	Rank
Fort Worth--Arlington, TX	Non-Hispanic Black	1992	6,046	5,640	6,807	17	620.17	578.52	698.25	8
		1993	5,025	2,183	6,614	20	503.55	218.77	662.72	12
		1994	5,858	5,488	6,370	17	565.96	530.14	615.34	7
		1995	4,794	2,168	6,042	19	446.47	201.88	562.63	13
		1996	4,597	2,120	5,655	20	414.68	191.25	510.17	13
		1997	4,393	2,066	5,352	21	381.95	179.62	465.34	13
		1998	4,165	2,004	5,106	21	349.79	168.32	428.84	13
		1999	4,608	4,438	4,864	19	373.06	359.27	393.76	9
		2000	3,744	1,820	4,534	21	293.06	142.43	354.89	14
		2001	4,182	4,041	4,279	21	314.14	303.58	321.41	14
		2002	3,389	1,525	4,388	22	245.27	110.35	317.58	22
		2003	3,256	1,400	4,534	22	228.48	98.25	318.19	22
		2004	3,152	1,295	4,750	24	214.90	88.29	323.93	25
		2005	3,111	1,204	5,087	23	205.76	79.62	336.46	29
		2006	3,163	1,138	5,661	23	197.74	71.13	353.86	34
		2007	3,289	1,072	6,460	24	198.23	64.59	389.33	36
	Hispanic	1992	1,936	1,806	2,179	35	168.40	157.09	189.60	38
		1993	1,621	704	2,134	35	132.16	57.42	173.94	41
		1994	1,932	1,810	2,101	34	146.59	137.31	159.38	38
		1995	1,636	740	2,062	35	115.36	52.16	145.37	45
		1996	1,639	756	2,016	35	107.27	49.48	131.98	46
		1997	1,649	775	2,009	35	99.92	46.99	121.74	46
		1998	1,655	796	2,029	35	93.03	44.76	114.05	45
		1999	1,943	1,872	2,051	35	101.40	97.66	107.03	41
		2000	1,677	815	2,031	37	81.25	39.49	98.39	49
		2001	1,985	1,918	2,030	37	89.98	86.95	92.06	46
		2002	1,696	763	2,196	37	72.38	32.57	93.72	54
		2003	1,705	733	2,374	37	69.20	29.76	96.37	57
		2004	1,710	703	2,578	36	66.30	27.24	99.93	58
		2005	1,728	668	2,825	36	63.84	24.70	104.39	58
		2006	1,772	637	3,171	36	61.94	22.28	110.84	59
		2007	1,830	596	3,594	35	60.28	19.64	118.40	59

Metropolitan Statistical Area	PWID Population	Year	Number	Min	Max	Rank	Rate	Min	Max	Rank
Fort Worth--Arlington, TX	Male	1992	15,027	14,018	16,919	17	312.41	291.43	351.74	9
		1993	12,345	5,363	16,247	23	253.65	110.20	333.83	13
		1994	14,489	13,572	15,753	15	290.92	272.51	316.30	8
		1995	12,117	5,479	15,269	23	238.02	107.63	299.94	14
		1996	12,009	5,538	14,774	21	230.71	106.41	283.84	14
		1997	11,959	5,624	14,570	20	223.46	105.08	272.25	13
		1998	11,866	5,710	14,547	20	215.57	103.73	264.30	13
		1999	13,744	13,236	14,506	13	242.62	233.65	256.08	12
		2000	11,648	5,661	14,105	20	199.93	97.17	242.12	18
		2001	13,463	13,010	13,775	16	225.17	217.59	230.38	14
		2002	11,151	5,017	14,438	20	181.77	81.78	235.36	23
		2003	10,761	4,628	14,987	19	172.03	73.98	239.58	23
		2004	10,238	4,206	15,432	22	160.46	65.92	241.86	26
		2005	9,669	3,741	15,810	25	148.25	57.37	242.42	34
		2006	9,110	3,277	16,302	26	135.86	48.87	243.12	36
		2007	8,461	2,757	16,618	32	122.74	39.99	241.06	41
	Female	1992	9,032	8,425	10,169	14	189.26	176.55	213.09	2
		1993	7,566	3,287	9,957	19	156.41	67.95	205.85	9
		1994	8,983	8,414	9,767	14	181.70	170.20	197.56	4
		1995	7,555	3,416	9,520	19	149.16	67.45	187.97	9
		1996	7,501	3,460	9,229	18	144.88	66.82	178.23	11
		1997	7,471	3,513	9,102	18	140.43	66.04	171.09	12
		1998	7,415	3,568	9,091	18	135.59	65.24	166.23	11
		1999	8,611	8,293	9,089	13	153.09	147.43	161.58	6
		2000	7,348	3,571	8,899	19	127.21	61.82	154.05	12
		2001	8,607	8,318	8,806	16	145.61	140.71	148.97	7
		2002	7,285	3,277	9,432	18	120.35	54.15	155.84	12
		2003	7,260	3,122	10,111	18	117.52	50.54	163.66	13
		2004	7,223	2,967	10,887	19	114.80	47.16	173.05	16
		2005	7,237	2,800	11,834	18	112.75	43.63	184.37	16
		2006	7,356	2,646	13,163	19	111.47	40.10	199.48	16
		2007	7,507	2,446	14,744	18	110.85	36.12	217.71	17

Metropolitan Statistical Area	PWID Population	Year	Number	Min	Max	Rank	Rate	Min	Max	Rank
Fort Worth--Arlington, TX	Young (15-29)	1992	8,319	7,760	9,366	7	249.24	232.49	280.61	4
		1993	6,245	2,713	8,219	16	189.46	82.31	249.35	7
		1994	6,908	6,470	7,510	9	208.89	195.67	227.12	6
		1995	5,605	2,535	7,063	17	167.78	75.87	211.43	8
		1996	5,531	2,551	6,805	17	162.83	75.10	200.32	9
		1997	5,603	2,635	6,826	16	160.82	75.63	195.93	11
		1998	5,747	2,765	7,046	14	160.64	77.30	196.94	12
		1999	6,960	6,703	7,346	9	189.64	182.63	200.16	8
		2000	6,206	3,016	7,515	14	165.28	80.33	200.16	15
		2001	7,559	7,305	7,734	11	197.52	190.88	202.09	11
		2002	6,580	2,961	8,520	13	168.17	75.66	217.76	17
		2003	6,634	2,853	9,238	15	166.61	71.64	232.02	17
		2004	6,529	2,682	9,842	16	161.23	66.24	243.03	18
		2005	6,299	2,437	10,300	17	152.58	59.04	249.49	23
		2006	5,966	2,146	10,676	18	140.61	50.58	251.62	27
		2007	5,458	1,778	10,720	21	125.25	40.81	246.01	31
	Old (30-64)	1992	15,510	14,468	17,462	21	248.37	231.69	279.64	13
		1993	13,505	5,867	17,774	23	210.76	91.57	277.39	18
		1994	16,399	15,361	17,830	16	247.82	232.13	269.44	8
		1995	13,945	6,305	17,572	21	204.62	92.52	257.85	13
		1996	13,874	6,399	17,068	21	198.59	91.59	244.32	12
		1997	13,736	6,459	16,735	21	191.09	89.86	232.81	14
		1998	13,452	6,473	16,492	21	181.90	87.53	223.01	14
		1999	15,305	14,739	16,154	16	200.87	193.44	212.01	8
		2000	12,713	6,179	15,396	22	162.01	78.73	196.19	16
		2001	14,417	13,932	14,751	19	178.80	172.78	182.93	9
		2002	11,771	5,296	15,241	22	142.25	64.00	184.20	20
		2003	11,301	4,860	15,738	22	133.71	57.50	186.21	25
		2004	10,844	4,455	16,346	23	125.78	51.67	189.59	25
		2005	10,523	4,072	17,207	25	119.42	46.21	195.27	26
		2006	10,419	3,748	18,645	25	114.99	41.36	205.77	26
		2007	10,430	3,398	20,485	24	112.05	36.51	220.07	25

Metropolitan Statistical Area	PWID Population	Year	Number	Min	Max	Rank	Rate	Min	Max	Rank
Fresno, CA	Total	1992	11,992	6,542	17,155	38	239.68	130.74	342.85	8
		1993	16,640	6,878	29,058	26	325.24	134.44	567.96	1
		1994	12,968	7,263	17,674	35	249.26	139.59	339.70	3
		1995	16,354	7,700	25,226	26	310.38	146.15	478.77	1
		1996	16,301	8,356	23,461	26	302.93	155.28	435.99	1
		1997	16,370	9,665	21,739	25	297.03	175.38	394.45	1
		1998	16,208	10,447	19,845	25	288.23	185.78	352.91	1
		1999	15,123	10,424	19,215	30	262.85	181.17	333.97	4
		2000	15,153	9,551	20,505	26	257.82	162.51	348.87	4
		2001	14,610	7,939	21,834	30	243.85	132.51	364.44	4
		2002	14,071	6,222	23,392	28	229.00	101.26	380.70	5
		2003	13,991	5,021	25,095	29	221.72	79.57	397.69	6
		2004	14,404	4,452	26,841	28	222.94	68.91	415.44	6
		2005	14,835	3,847	28,584	26	225.22	58.40	433.97	6
		2006	15,338	3,220	30,411	24	228.57	47.99	453.19	6
		2007	15,893	2,556	32,250	22	232.84	37.45	472.49	5
	Non-Hispanic White	1992	5,768	3,147	8,251	42	225.41	122.96	322.44	4
		1993	7,132	2,948	12,454	27	279.84	115.68	488.69	2
		1994	5,129	2,873	6,991	44	202.99	113.68	276.64	6
		1995	6,181	2,911	9,535	33	247.31	116.45	381.47	2
		1996	6,072	3,112	8,738	32	244.11	125.13	351.33	2
		1997	6,156	3,635	8,175	32	246.44	145.51	327.27	1
		1998	6,258	4,034	7,663	32	250.02	161.15	306.12	2
		1999	6,051	4,171	7,689	35	240.51	165.77	305.58	3
		2000	6,297	3,969	8,520	31	249.76	157.43	337.96	3
		2001	6,277	3,411	9,381	34	247.05	134.24	369.21	3
		2002	6,189	2,737	10,289	33	241.09	106.60	400.80	3
		2003	6,205	2,227	11,129	34	238.76	85.69	428.25	3
		2004	6,311	1,951	11,760	32	240.97	74.48	449.04	3
		2005	6,253	1,621	12,048	33	238.39	61.82	459.35	3
		2006	6,010	1,262	11,915	36	229.12	48.10	454.28	4
		2007	5,535	890	11,232	40	211.25	33.98	428.67	6

Metropolitan Statistical Area	PWID Population	Year	Number	Min	Max	Rank	Rate	Min	Max	Rank
Fresno, CA	Non-Hispanic Black	1992	997	544	1,426	80	450.11	245.53	643.88	24
		1993	1,444	597	2,522	62	627.39	259.34	1095.62	8
		1994	1,145	641	1,561	68	481.83	269.84	656.67	14
		1995	1,440	678	2,221	61	583.31	274.66	899.76	7
		1996	1,409	722	2,028	60	538.46	276.01	774.96	8
		1997	1,375	812	1,826	61	503.46	297.26	668.57	7
		1998	1,317	849	1,613	62	466.23	300.51	570.85	8
		1999	1,190	820	1,512	63	405.83	279.71	515.62	8
		2000	1,162	732	1,572	62	382.02	240.79	516.94	9
		2001	1,103	600	1,649	63	354.05	192.39	529.12	9
		2002	1,065	471	1,770	62	333.60	147.51	554.61	11
		2003	1,085	389	1,945	62	329.53	118.26	591.06	11
		2004	1,176	363	2,191	61	343.27	106.10	639.67	11
		2005	1,318	342	2,540	58	373.70	96.91	720.09	9
		2006	1,541	323	3,055	49	429.50	90.17	851.58	8
		2007	1,882	303	3,819	38	516.08	83.01	1047.24	6
	Hispanic	1992	5,036	2,747	7,204	15	272.67	148.74	390.05	21
		1993	7,041	2,910	12,296	9	362.31	149.76	632.70	9
		1994	5,540	3,103	7,551	12	273.02	152.90	372.09	17
		1995	7,062	3,325	10,894	9	336.09	158.25	518.43	11
		1996	7,119	3,649	10,245	8	323.65	165.90	465.81	12
		1997	7,228	4,268	9,598	9	315.11	186.06	418.46	12
		1998	7,229	4,659	8,851	9	303.74	195.77	371.89	12
		1999	6,804	4,689	8,644	11	275.20	189.68	349.65	13
		2000	6,862	4,325	9,286	9	267.44	168.57	361.88	13
		2001	6,643	3,610	9,928	9	251.38	136.59	375.68	14
		2002	6,403	2,831	10,645	8	233.64	103.31	388.42	15
		2003	6,349	2,278	11,387	9	223.52	80.22	400.91	16
		2004	6,489	2,006	12,092	9	220.96	68.29	411.74	16
		2005	6,602	1,712	12,721	9	217.70	56.46	419.49	17
		2006	6,704	1,407	13,292	8	214.06	44.94	424.43	16
		2007	6,778	1,090	13,754	8	209.93	33.77	425.99	17

Metropolitan Statistical Area	PWID Population	Year	Number	Min	Max	Rank	Rate	Min	Max	Rank
Fresno, CA	Male	1992	7,351	4,010	10,515	40	291.66	159.10	417.22	15
		1993	10,405	4,301	18,170	27	403.40	166.75	704.46	2
		1994	8,236	4,613	11,225	37	314.38	176.06	428.45	5
		1995	10,511	4,949	16,213	26	395.84	186.39	610.59	1
		1996	10,570	5,418	15,213	26	388.15	198.96	558.63	1
		1997	10,683	6,307	14,186	25	382.98	226.13	508.59	1
		1998	10,624	6,848	13,008	25	373.76	240.90	457.62	1
		1999	9,942	6,852	12,632	28	342.35	235.96	434.97	2
		2000	9,980	6,291	13,505	24	336.76	212.27	455.69	3
		2001	9,634	5,235	14,399	28	318.58	173.11	476.12	5
		2002	9,289	4,107	15,443	25	298.81	132.13	496.76	5
		2003	9,248	3,319	16,588	25	289.86	104.03	519.91	6
		2004	9,540	2,949	17,777	25	292.13	90.29	544.36	6
		2005	9,855	2,555	18,989	23	295.69	76.68	569.77	6
		2006	10,234	2,149	20,292	23	301.50	63.30	597.78	5
		2007	10,671	1,716	21,654	21	308.70	49.65	626.42	5
	Female	1992	4,595	2,507	6,573	35	185.03	100.93	264.68	3
		1993	6,217	2,570	10,857	23	245.07	101.30	427.96	2
		1994	4,750	2,660	6,474	36	183.91	103.00	250.65	3
		1995	5,901	2,779	9,102	24	225.78	106.31	348.27	2
		1996	5,818	2,982	8,373	24	218.90	112.21	315.05	2
		1997	5,798	3,424	7,700	24	213.04	125.78	282.90	3
		1998	5,712	3,682	6,994	25	205.42	132.40	251.51	3
		1999	5,312	3,661	6,749	28	186.43	128.50	236.87	4
		2000	5,310	3,347	7,186	26	182.24	114.87	246.60	4
		2001	5,108	2,776	7,634	29	172.16	93.55	257.30	4
		2002	4,905	2,169	8,154	29	161.56	71.44	268.60	6
		2003	4,855	1,742	8,707	31	155.62	55.85	279.13	6
		2004	4,963	1,534	9,249	28	155.33	48.01	289.45	6
		2005	5,060	1,312	9,749	27	155.49	40.32	299.61	6
		2006	5,157	1,083	10,225	26	155.53	32.65	308.37	5
		2007	5,242	843	10,637	25	155.60	25.03	315.75	5

Metropolitan Statistical Area	PWID Population	Year	Number	Min	Max	Rank	Rate	Min	Max	Rank
Fresno, CA	Young (15-29)	1992	2,605	1,421	3,726	44	140.43	76.60	200.88	21
		1993	3,258	1,347	5,689	33	173.83	71.85	303.55	11
		1994	2,306	1,291	3,142	44	122.45	68.57	166.88	26
		1995	2,662	1,253	4,106	39	140.23	66.03	216.31	16
		1996	2,450	1,256	3,526	43	126.28	64.73	181.74	23
		1997	2,292	1,353	3,044	46	115.16	67.99	152.93	35
		1998	2,133	1,375	2,612	55	104.61	67.43	128.08	37
		1999	1,888	1,301	2,399	64	90.25	62.20	114.66	48
		2000	1,811	1,142	2,451	64	84.45	53.23	114.27	54
		2001	1,687	917	2,522	74	77.06	41.88	115.17	65
		2002	1,585	701	2,635	76	70.11	31.00	116.56	68
		2003	1,551	557	2,782	78	66.45	23.85	119.19	76
		2004	1,586	490	2,956	78	65.80	20.34	122.62	77
		2005	1,638	425	3,156	78	66.08	17.14	127.34	78
		2006	1,713	360	3,396	79	67.39	14.15	133.62	80
		2007	1,812	291	3,676	77	69.83	11.23	141.69	77
	Old (30-64)	1992	9,526	5,196	13,627	36	302.55	165.04	432.80	4
		1993	13,500	5,580	23,575	24	416.40	172.12	727.17	1
		1994	10,729	6,009	14,623	33	323.20	181.00	440.48	3
		1995	13,773	6,485	21,246	23	408.64	192.41	630.33	1
		1996	13,947	7,149	20,073	20	405.36	207.78	583.40	1
		1997	14,196	8,382	18,852	19	403.23	238.08	535.47	1
		1998	14,214	9,162	17,404	19	396.60	255.62	485.59	1
		1999	13,381	9,222	17,001	21	365.46	251.89	464.33	1
		2000	13,496	8,506	18,262	21	361.55	227.89	489.23	1
		2001	13,068	7,101	19,531	21	343.75	186.79	513.73	3
		2002	12,614	5,578	20,970	20	324.75	143.60	539.88	3
		2003	12,541	4,501	22,495	20	315.41	113.20	565.74	3
		2004	12,879	3,981	24,000	20	317.98	98.28	592.54	3
		2005	13,193	3,421	25,421	19	321.11	83.27	618.74	3
		2006	13,516	2,837	26,798	20	324.23	68.07	642.85	2
		2007	13,803	2,220	28,010	19	326.24	52.48	662.01	2

Metropolitan Statistical Area	PWID Population	Year	Number	Min	Max	Rank	Rate	Min	Max	Rank
Gary, IN	Total	1992	4,688	2,568	6,400	79	116.48	63.82	159.04	47
		1993	4,166	2,363	6,486	82	102.83	58.32	160.09	50
		1994	4,835	2,163	6,556	79	118.83	53.16	161.15	44
		1995	4,634	1,965	6,640	81	113.21	47.99	162.21	43
		1996	4,851	1,784	6,718	80	117.91	43.36	163.28	40
		1997	5,035	1,597	6,840	77	122.08	38.74	165.85	38
		1998	5,232	1,493	7,160	73	126.64	36.13	173.31	36
		1999	5,249	1,386	7,474	79	126.94	33.52	180.76	35
		2000	5,532	1,498	7,784	70	133.47	36.14	187.79	28
		2001	5,575	1,611	8,099	76	134.10	38.75	194.82	29
		2002	5,681	1,887	8,486	74	136.14	45.22	203.36	24
		2003	5,790	2,167	8,882	72	138.14	51.70	211.90	21
		2004	5,914	2,475	9,315	71	140.08	58.61	220.62	20
		2005	6,060	2,793	9,774	70	142.20	65.53	229.35	19
		2006	6,206	3,131	10,230	70	144.41	72.86	238.03	18
		2007	6,362	3,474	10,689	69	146.85	80.18	246.71	18
	Non-Hispanic White	1992	2,454	1,345	3,351	76	83.99	46.02	114.68	45
		1993	2,064	1,171	3,213	79	70.45	39.96	109.68	55
		1994	2,309	1,033	3,131	79	78.82	35.26	106.89	48
		1995	2,172	921	3,112	79	74.08	31.41	106.15	53
		1996	2,267	834	3,139	77	77.31	28.43	107.06	52
		1997	2,378	755	3,231	77	81.41	25.83	110.60	51
		1998	2,524	720	3,454	77	86.82	24.77	118.81	42
		1999	2,607	688	3,712	77	90.05	23.78	128.23	38
		2000	2,841	769	3,998	74	98.49	26.66	138.57	31
		2001	2,967	857	4,311	76	102.86	29.72	149.43	33
		2002	3,132	1,040	4,678	69	108.57	36.07	162.17	27
		2003	3,298	1,234	5,059	69	114.36	42.80	175.41	22
		2004	3,467	1,451	5,460	66	120.04	50.23	189.07	17
		2005	3,634	1,675	5,862	66	125.44	57.81	202.32	15
		2006	3,783	1,909	6,236	62	130.31	65.74	214.78	13
		2007	3,910	2,135	6,570	61	134.47	73.42	225.91	13

Metropolitan Statistical Area	PWID Population	Year	Number	Min	Max	Rank	Rate	Min	Max	Rank
Gary, IN	Non-Hispanic Black	1992	1,770	970	2,416	62	237.30	130.00	323.98	65
		1993	1,668	946	2,598	54	221.22	125.47	344.41	58
		1994	2,004	896	2,717	53	263.23	117.75	356.97	52
		1995	1,947	826	2,790	49	253.54	107.49	363.30	48
		1996	2,032	747	2,814	45	263.59	96.94	365.02	43
		1997	2,073	658	2,816	41	267.10	84.75	362.87	34
		1998	2,091	597	2,862	39	268.52	76.60	367.45	27
		1999	2,017	532	2,872	42	258.53	68.27	368.15	23
		2000	2,028	549	2,853	39	258.67	70.03	363.93	22
		2001	1,939	560	2,818	42	246.88	71.34	358.65	22
		2002	1,872	622	2,797	42	236.80	78.67	353.73	23
		2003	1,811	678	2,777	41	227.30	85.06	348.65	23
		2004	1,765	738	2,780	40	218.71	91.51	344.46	24
		2005	1,743	803	2,811	38	213.22	98.26	343.89	27
		2006	1,745	881	2,877	41	210.99	106.45	347.77	27
		2007	1,783	974	2,995	45	212.94	116.27	357.76	31
	Hispanic	1992	300	164	409	61	93.53	51.24	127.70	63
		1993	267	152	416	60	81.00	45.94	126.11	62
		1994	313	140	424	59	92.57	41.41	125.53	58
		1995	304	129	436	59	86.46	36.65	123.89	56
		1996	324	119	449	59	88.53	32.56	122.60	51
		1997	344	109	467	59	90.39	28.68	122.80	49
		1998	366	104	500	58	92.32	26.33	126.33	48
		1999	376	99	535	59	92.15	24.33	131.22	45
		2000	407	110	572	59	96.57	26.15	135.87	44
		2001	421	122	611	59	97.86	28.28	142.16	41
		2002	440	146	657	60	100.21	33.29	149.69	43
		2003	460	172	705	58	102.04	38.19	156.52	40
		2004	481	201	758	58	103.54	43.33	163.08	38
		2005	505	233	815	58	104.73	48.26	168.92	37
		2006	530	268	874	59	105.94	53.45	174.62	35
		2007	557	304	936	59	107.46	58.67	180.53	33

Metropolitan Statistical Area	PWID Population	Year	Number	Min	Max	Rank	Rate	Min	Max	Rank
Gary, IN	Male	1992	3,504	1,920	4,784	76	179.12	98.13	244.56	37
		1993	3,052	1,731	4,752	80	154.82	87.81	241.04	40
		1994	3,489	1,561	4,732	76	176.10	78.78	238.81	35
		1995	3,310	1,403	4,742	76	165.78	70.28	237.54	36
		1996	3,443	1,266	4,769	74	171.51	63.08	237.51	36
		1997	3,564	1,131	4,842	72	176.93	56.14	240.36	34
		1998	3,705	1,057	5,070	67	183.49	52.34	251.09	28
		1999	3,724	983	5,304	72	184.16	48.63	262.24	25
		2000	3,938	1,066	5,540	64	194.18	52.57	273.19	20
		2001	3,981	1,150	5,783	68	195.80	56.58	284.45	19
		2002	4,066	1,351	6,074	63	199.25	66.19	297.63	17
		2003	4,148	1,552	6,363	61	202.28	75.70	310.27	15
		2004	4,230	1,770	6,662	59	204.89	85.74	322.71	15
		2005	4,312	1,987	6,955	59	206.98	95.38	333.83	13
		2006	4,376	2,208	7,212	57	208.38	105.13	343.47	12
		2007	4,419	2,413	7,424	57	208.85	114.04	350.88	12
	Female	1992	1,346	738	1,838	82	65.10	35.66	88.88	58
		1993	1,237	702	1,926	83	59.47	33.73	92.59	60
		1994	1,483	664	2,011	83	71.07	31.79	96.37	53
		1995	1,467	622	2,102	82	69.97	29.66	100.27	51
		1996	1,583	582	2,192	80	75.13	27.63	104.04	47
		1997	1,689	536	2,294	80	80.06	25.40	108.76	44
		1998	1,799	513	2,462	77	85.19	24.30	116.58	37
		1999	1,845	487	2,627	79	87.34	23.06	124.37	33
		2000	1,980	536	2,786	73	93.55	25.33	131.62	26
		2001	2,024	585	2,941	74	95.30	27.54	138.44	28
		2002	2,083	692	3,111	72	97.69	32.45	145.93	22
		2003	2,133	798	3,272	67	99.65	37.29	152.85	21
		2004	2,178	911	3,430	67	100.93	42.23	158.96	21
		2005	2,217	1,022	3,576	67	101.78	46.91	164.17	21
		2006	2,241	1,131	3,694	69	101.97	51.45	168.08	19
		2007	2,252	1,230	3,784	69	101.59	55.47	170.68	19

Metropolitan Statistical Area	PWID Population	Year	Number	Min	Max	Rank	Rate	Min	Max	Rank
Gary, IN	Young (15-29)	1992	1,204	659	1,643	79	92.09	50.45	125.73	48
		1993	868	492	1,351	82	66.76	37.86	103.93	60
		1994	889	398	1,205	83	68.79	30.77	93.29	60
		1995	814	345	1,167	85	62.91	26.67	90.14	57
		1996	874	321	1,210	84	67.26	24.74	93.14	58
		1997	982	312	1,334	84	75.45	23.94	102.50	57
		1998	1,150	328	1,573	83	88.39	25.21	120.96	48
		1999	1,329	351	1,893	82	103.02	27.20	146.70	38
		2000	1,627	441	2,290	73	127.22	34.44	178.98	27
		2001	1,892	547	2,749	69	149.66	43.25	217.42	20
		2002	2,185	726	3,264	59	173.64	57.68	259.37	15
		2003	2,458	920	3,771	52	194.81	72.90	298.82	11
		2004	2,685	1,123	4,228	52	210.56	88.11	331.64	9
		2005	2,835	1,306	4,572	48	219.75	101.27	354.42	11
		2006	2,867	1,446	4,725	50	220.03	111.01	362.67	8
		2007	2,751	1,502	4,622	55	209.90	114.61	352.64	11
	Old (30-64)	1992	3,657	2,004	4,993	76	134.61	73.74	183.78	42
		1993	3,406	1,932	5,303	79	123.80	70.22	192.74	44
		1994	4,057	1,815	5,501	76	146.09	65.35	198.12	38
		1995	3,936	1,668	5,639	73	140.61	59.61	201.48	35
		1996	4,129	1,518	5,717	71	146.64	53.93	203.07	31
		1997	4,258	1,351	5,785	69	150.86	47.87	204.95	27
		1998	4,363	1,245	5,971	66	154.14	43.97	210.94	25
		1999	4,282	1,131	6,098	72	150.56	39.76	214.39	27
		2000	4,380	1,186	6,162	64	152.85	41.38	215.05	19
		2001	4,248	1,228	6,172	72	146.84	42.43	213.32	25
		2002	4,135	1,374	6,177	65	141.88	47.13	211.94	22
		2003	4,003	1,498	6,141	66	136.65	51.14	209.61	23
		2004	3,874	1,621	6,102	69	131.44	55.00	207.02	24
		2005	3,767	1,736	6,076	68	126.77	58.42	204.47	23
		2006	3,689	1,861	6,081	69	123.18	62.15	203.04	20
		2007	3,666	2,002	6,159	68	121.31	66.24	203.81	19

Metropolitan Statistical Area	PWID Population	Year	Number	Min	Max	Rank	Rate	Min	Max	Rank
Grand Rapids--Muskegon--Holland, MI	Total	1992	3,165	2,579	3,601	89	50.55	41.18	57.50	96
		1993	2,666	1,412	3,472	89	42.02	22.25	54.71	97
		1994	3,016	2,425	3,360	90	46.73	37.56	52.05	96
		1995	2,659	1,790	3,248	90	40.45	27.23	49.41	97
		1996	2,662	1,994	3,237	90	39.70	29.74	48.27	97
		1997	2,660	2,194	3,219	90	38.97	32.15	47.15	97
		1998	2,686	2,201	3,193	90	38.76	31.76	46.06	97
		1999	2,728	2,213	3,175	90	38.66	31.36	45.00	98
		2000	2,758	2,353	3,154	90	38.43	32.80	43.96	97
		2001	2,756	2,494	3,129	91	37.83	34.23	42.94	98
		2002	2,944	2,590	3,113	90	39.93	35.13	42.22	97
		2003	3,059	2,529	3,741	90	41.05	33.94	50.21	97
		2004	3,184	2,475	4,521	90	42.33	32.90	60.09	97
		2005	3,312	2,423	5,321	90	43.54	31.86	69.97	97
		2006	3,449	2,302	6,201	90	44.96	30.01	80.83	97
		2007	3,580	2,057	7,084	90	46.34	26.63	91.70	97
	Non-Hispanic White	1992	1,680	1,368	1,911	88	30.35	24.73	34.53	97
		1993	1,442	764	1,877	93	25.83	13.68	33.63	99
		1994	1,648	1,325	1,836	91	29.16	23.44	32.48	97
		1995	1,458	982	1,781	91	25.47	17.15	31.11	98
		1996	1,459	1,093	1,774	91	25.12	18.82	30.55	98
		1997	1,452	1,198	1,757	91	24.70	20.38	29.89	99
		1998	1,458	1,194	1,733	92	24.58	20.14	29.22	99
		1999	1,472	1,194	1,713	91	24.55	19.92	28.58	99
		2000	1,482	1,264	1,695	93	24.47	20.88	27.99	99
		2001	1,479	1,338	1,679	94	24.14	21.84	27.41	100
		2002	1,585	1,394	1,676	94	25.63	22.55	27.10	98
		2003	1,662	1,374	2,033	94	26.67	22.05	32.62	97
		2004	1,758	1,367	2,496	94	28.02	21.77	39.77	98
		2005	1,873	1,370	3,009	92	29.58	21.64	47.54	98
		2006	2,014	1,344	3,620	86	31.62	21.10	56.84	98
		2007	2,174	1,250	4,303	85	33.98	19.53	67.25	96

Metropolitan Statistical Area	PWID Population	Year	Number	Min	Max	Rank	Rate	Min	Max	Rank
Grand Rapids--Muskegon--Holland, MI	Non-Hispanic Black	1992	1,048	854	1,193	77	251.38	204.80	285.97	60
		1993	910	482	1,185	80	212.74	112.66	277.01	60
		1994	1,045	840	1,164	71	237.64	191.03	264.68	59
		1995	923	621	1,127	72	204.13	137.44	249.33	57
		1996	915	685	1,112	69	197.58	148.01	240.22	54
		1997	896	739	1,084	68	188.81	155.77	228.47	55
		1998	878	719	1,044	68	180.70	148.06	214.78	57
		1999	859	697	1,000	67	172.05	139.58	200.28	64
		2000	831	709	950	68	162.20	138.43	185.55	56
		2001	791	715	897	71	152.25	137.76	172.83	64
		2002	801	705	847	69	152.79	134.43	161.57	56
		2003	789	652	965	69	148.71	122.96	181.90	55
		2004	779	605	1,106	71	144.90	112.63	205.72	55
		2005	771	564	1,239	71	141.75	103.70	227.76	54
		2006	770	514	1,384	74	139.50	93.12	250.80	56
		2007	774	445	1,531	76	138.64	79.67	274.36	55
	Hispanic	1992	303	247	345	59	141.52	115.30	160.99	48
		1993	221	117	288	63	95.31	50.48	124.10	55
		1994	222	179	248	66	87.89	70.65	97.89	61
		1995	179	120	218	68	64.28	43.28	78.52	68
		1996	166	125	202	69	53.98	40.44	65.62	73
		1997	158	130	191	69	46.74	38.57	56.56	74
		1998	154	126	183	72	41.55	34.05	49.39	76
		1999	153	124	178	73	37.70	30.59	43.88	80
		2000	153	131	175	73	34.76	29.66	39.76	78
		2001	153	138	173	74	32.61	29.50	37.01	82
		2002	164	144	173	73	33.54	29.51	35.46	78
		2003	172	142	210	73	33.88	28.01	41.44	77
		2004	181	141	257	73	34.48	26.80	48.95	75
		2005	190	139	305	73	34.92	25.55	56.12	74
		2006	200	133	359	73	35.54	23.72	63.90	74
		2007	208	120	412	73	36.03	20.70	71.30	74

Metropolitan Statistical Area	PWID Population	Year	Number	Min	Max	Rank	Rate	Min	Max	Rank
Grand Rapids--Muskegon--Holland, MI	Male	1992	2,279	1,857	2,593	88	73.44	59.83	83.54	94
		1993	1,880	995	2,448	88	59.68	31.61	77.71	97
		1994	2,087	1,677	2,324	89	65.03	52.28	72.43	97
		1995	1,809	1,218	2,209	88	55.28	37.22	67.52	97
		1996	1,785	1,337	2,170	88	53.41	40.01	64.93	97
		1997	1,762	1,454	2,132	88	51.74	42.68	62.60	98
		1998	1,762	1,444	2,094	88	50.88	41.69	60.47	98
		1999	1,775	1,440	2,066	89	50.28	40.79	58.53	98
		2000	1,783	1,522	2,040	88	49.65	42.37	56.80	97
		2001	1,774	1,605	2,014	89	48.61	43.98	55.18	98
		2002	1,888	1,661	1,997	88	51.10	44.97	54.04	97
		2003	1,957	1,618	2,394	88	52.37	43.30	64.06	97
		2004	2,034	1,581	2,888	88	53.89	41.89	76.51	97
		2005	2,113	1,546	3,394	88	55.34	40.49	88.93	97
		2006	2,198	1,467	3,951	88	57.04	38.08	102.56	97
		2007	2,277	1,309	4,507	87	58.71	33.74	116.19	97
	Female	1992	941	767	1,071	92	29.79	24.27	33.89	97
		1993	832	440	1,083	92	26.02	13.78	33.88	98
		1994	981	789	1,093	92	30.21	24.29	33.65	96
		1995	896	603	1,095	91	27.14	18.27	33.15	99
		1996	925	693	1,124	91	27.50	20.60	33.43	98
		1997	948	782	1,147	89	27.70	22.85	33.52	96
		1998	976	800	1,161	89	28.15	23.06	33.46	96
		1999	1,007	817	1,172	91	28.56	23.17	33.24	97
		2000	1,029	879	1,178	91	28.72	24.51	32.85	97
		2001	1,036	937	1,176	93	28.49	25.78	32.34	98
		2002	1,110	976	1,173	92	30.17	26.54	31.90	97
		2003	1,151	952	1,408	91	31.01	25.64	37.93	97
		2004	1,193	927	1,693	91	31.82	24.73	45.17	97
		2005	1,229	899	1,975	91	32.45	23.74	52.15	97
		2006	1,264	844	2,273	89	33.11	22.10	59.52	95
		2007	1,291	742	2,555	89	33.57	19.29	66.43	94

Metropolitan Statistical Area	PWID Population	Year	Number	Min	Max	Rank	Rate	Min	Max	Rank
Grand Rapids--Muskegon--Holland, MI	Young (15-29)	1992	819	667	932	86	37.37	30.45	42.51	95
		1993	672	356	875	89	30.71	16.26	39.99	95
		1994	747	601	833	89	34.00	27.33	37.86	96
		1995	654	441	799	92	29.43	19.82	35.95	95
		1996	658	493	800	92	29.13	21.82	35.42	95
		1997	668	551	808	92	29.10	24.01	35.21	97
		1998	693	568	824	92	29.79	24.41	35.41	97
		1999	732	594	852	92	31.08	25.22	36.18	99
		2000	779	665	891	92	32.73	27.93	37.44	98
		2001	830	751	942	94	34.53	31.24	39.20	100
		2002	955	840	1,010	91	39.41	34.68	41.67	99
		2003	1,080	893	1,322	91	44.14	36.50	53.99	97
		2004	1,236	960	1,754	89	50.09	38.93	71.11	94
		2005	1,421	1,039	2,283	87	57.02	41.72	91.62	88
		2006	1,643	1,097	2,954	80	65.46	43.70	117.69	85
		2007	1,895	1,089	3,749	76	75.30	43.27	149.02	68
	Old (30-64)	1992	2,416	1,969	2,749	88	59.37	48.37	67.54	93
		1993	2,041	1,081	2,657	89	49.09	26.00	63.91	95
		1994	2,314	1,860	2,577	89	54.36	43.70	60.55	94
		1995	2,043	1,376	2,496	88	46.97	31.62	57.37	95
		1996	2,045	1,532	2,487	88	45.99	34.46	55.92	96
		1997	2,038	1,682	2,467	88	44.98	37.11	54.43	96
		1998	2,046	1,676	2,432	88	44.42	36.40	52.80	95
		1999	2,055	1,667	2,392	89	43.71	35.46	50.88	97
		2000	2,042	1,743	2,336	88	42.59	36.35	48.72	95
		2001	1,991	1,802	2,260	88	40.79	36.90	46.30	97
		2002	2,054	1,807	2,172	88	41.50	36.52	43.89	95
		2003	2,034	1,682	2,488	88	40.65	33.61	49.73	95
		2004	1,983	1,541	2,815	88	39.21	30.48	55.66	95
		2005	1,885	1,379	3,028	88	36.86	26.96	59.22	96
		2006	1,737	1,160	3,124	93	33.66	22.47	60.51	96
		2007	1,531	880	3,029	94	29.38	16.88	58.14	99

Metropolitan Statistical Area	PWID Population	Year	Number	Min	Max	Rank	Rate	Min	Max	Rank
Greensboro--Winston-Salem--High Point, NC	Total	1992	6,553	4,659	8,067	65	88.40	62.86	108.83	63
		1993	5,275	1,684	7,673	75	70.14	22.39	102.02	77
		1994	6,401	4,779	7,285	66	83.70	62.49	95.25	66
		1995	5,184	1,690	7,269	74	66.52	21.68	93.28	78
		1996	5,149	1,711	7,381	75	64.85	21.55	92.97	77
		1997	5,112	1,740	7,481	75	63.32	21.56	92.66	77
		1998	5,080	1,772	7,575	77	61.83	21.56	92.19	77
		1999	6,115	5,094	7,644	67	73.38	61.13	91.72	68
		2000	4,997	1,882	7,777	79	59.14	22.27	92.04	78
		2001	5,947	4,941	7,894	71	69.57	57.81	92.35	72
		2002	4,893	2,018	8,014	80	56.72	23.39	92.92	81
		2003	4,829	2,080	8,116	81	55.62	23.95	93.48	83
		2004	4,779	2,154	8,223	81	54.54	24.58	93.84	86
		2005	4,757	2,236	8,377	81	53.49	25.14	94.19	93
		2006	4,750	2,327	8,560	82	52.44	25.69	94.51	93
		2007	4,725	2,400	8,724	81	51.36	26.09	94.83	92
	Non-Hispanic White	1992	3,495	2,485	4,303	64	60.32	42.90	74.26	61
		1993	2,845	909	4,139	69	48.70	15.55	70.84	71
		1994	3,489	2,605	3,970	66	59.15	44.16	67.31	62
		1995	2,853	930	4,000	68	47.85	15.60	67.09	71
		1996	2,861	951	4,101	72	47.48	15.78	68.07	71
		1997	2,867	976	4,196	72	47.25	16.09	69.14	72
		1998	2,877	1,003	4,289	73	47.09	16.42	70.21	73
		1999	3,497	2,913	4,371	65	57.12	47.58	71.40	66
		2000	2,887	1,087	4,492	72	47.10	17.74	73.31	74
		2001	3,473	2,885	4,610	65	56.47	46.92	74.96	69
		2002	2,890	1,192	4,734	73	46.90	19.34	76.83	76
		2003	2,887	1,243	4,852	74	46.87	20.18	78.76	76
		2004	2,895	1,305	4,980	76	46.89	21.13	80.67	80
		2005	2,921	1,373	5,144	76	46.98	22.08	82.73	80
		2006	2,958	1,449	5,332	77	47.10	23.07	84.88	81
		2007	2,987	1,518	5,516	78	47.23	24.00	87.20	84

Metropolitan Statistical Area	PWID Population	Year	Number	Min	Max	Rank	Rate	Min	Max	Rank
Greensboro--Winston-Salem--High Point, NC	Non-Hispanic Black	1992	2,871	2,041	3,534	39	197.88	140.71	243.60	78
		1993	2,233	713	3,249	45	150.29	47.99	218.60	84
		1994	2,620	1,956	2,982	38	172.02	128.43	195.76	78
		1995	2,052	669	2,878	45	131.38	42.82	184.23	85
		1996	1,972	655	2,827	47	123.53	41.04	177.08	85
		1997	1,895	645	2,773	47	116.38	39.62	170.30	84
		1998	1,823	636	2,718	48	109.82	38.30	163.74	85
		1999	2,125	1,770	2,656	39	126.17	105.10	157.71	81
		2000	1,683	634	2,619	49	98.32	37.02	153.02	86
		2001	1,942	1,614	2,578	41	111.40	92.56	147.88	80
		2002	1,551	640	2,541	53	87.44	36.06	143.24	86
		2003	1,488	641	2,501	54	82.72	35.62	139.02	87
		2004	1,434	646	2,468	55	78.16	35.23	134.49	87
		2005	1,393	655	2,453	55	74.09	34.82	130.47	86
		2006	1,362	667	2,454	56	70.33	34.45	126.74	87
		2007	1,332	677	2,459	57	67.07	34.08	123.84	87
	Hispanic	1992	26	18	31	92	31.91	22.69	39.28	94
		1993	22	7	32	92	22.73	7.26	33.06	95
		1994	30	22	34	92	24.10	18.00	27.43	95
		1995	27	9	38	92	17.52	5.71	24.57	97
		1996	31	10	44	91	15.74	5.23	22.56	97
		1997	35	12	51	91	14.28	4.86	20.89	99
		1998	40	14	60	89	13.14	4.58	19.59	99
		1999	55	46	69	89	14.94	12.44	18.67	96
		2000	52	20	81	89	11.80	4.44	18.37	98
		2001	71	59	94	87	14.77	12.27	19.61	95
		2002	66	27	108	88	12.95	5.34	21.21	97
		2003	73	31	122	88	13.59	5.85	22.83	97
		2004	79	36	136	88	14.03	6.32	24.14	97
		2005	84	40	149	87	14.17	6.66	24.96	97
		2006	89	43	160	87	14.02	6.87	25.28	98
		2007	90	46	167	86	13.45	6.83	24.83	97

Metropolitan Statistical Area	PWID Population	Year	Number	Min	Max	Rank	Rate	Min	Max	Rank
Greensboro--Winston-Salem--High Point, NC	Male	1992	4,217	2,999	5,192	68	116.79	83.04	143.77	63
		1993	3,369	1,076	4,900	75	92.03	29.38	133.85	78
		1994	4,062	3,033	4,623	68	109.01	81.39	124.06	69
		1995	3,273	1,067	4,590	77	86.15	28.08	120.80	78
		1996	3,239	1,076	4,643	77	83.63	27.79	119.89	78
		1997	3,207	1,092	4,694	78	81.35	27.69	119.04	78
		1998	3,182	1,110	4,744	78	79.25	27.64	118.16	79
		1999	3,827	3,188	4,784	69	93.80	78.14	117.25	68
		2000	3,127	1,178	4,867	80	75.36	28.38	117.29	78
		2001	3,723	3,094	4,943	73	88.73	73.72	117.78	71
		2002	3,066	1,265	5,023	80	72.46	29.88	118.69	80
		2003	3,031	1,305	5,094	82	71.34	30.72	119.90	80
		2004	3,004	1,354	5,169	83	69.99	31.55	120.43	83
		2005	2,995	1,407	5,274	84	68.80	32.33	121.15	87
		2006	2,995	1,467	5,397	83	67.44	33.04	121.55	88
		2007	2,983	1,516	5,508	80	66.29	33.68	122.41	88
	Female	1992	2,407	1,711	2,963	63	63.31	45.02	77.94	62
		1993	1,961	626	2,853	68	50.81	16.22	73.91	71
		1994	2,406	1,796	2,738	63	61.35	45.80	69.81	62
		1995	1,966	641	2,756	71	49.23	16.05	69.03	76
		1996	1,966	653	2,819	71	48.36	16.07	69.33	77
		1997	1,963	668	2,873	73	47.52	16.18	69.54	76
		1998	1,958	683	2,919	74	46.60	16.25	69.49	76
		1999	2,361	1,967	2,951	64	55.50	46.23	69.38	67
		2000	1,929	726	3,002	76	44.85	16.89	69.81	77
		2001	2,291	1,903	3,041	68	52.64	43.74	69.88	69
		2002	1,877	774	3,074	77	42.71	17.61	69.97	84
		2003	1,841	793	3,093	77	41.51	17.88	69.76	84
		2004	1,806	814	3,108	80	40.39	18.20	69.49	86
		2005	1,778	836	3,132	81	39.17	18.41	68.97	88
		2006	1,752	858	3,158	82	37.95	18.59	68.40	92
		2007	1,716	872	3,169	81	36.51	18.55	67.42	91

Metropolitan Statistical Area	PWID Population	Year	Number	Min	Max	Rank	Rate	Min	Max	Rank
Greensboro--Winston-Salem--High Point, NC	Young (15-29)	1992	1,808	1,286	2,226	63	73.13	52.00	90.03	61
		1993	1,341	428	1,950	74	54.51	17.40	79.29	69
		1994	1,541	1,151	1,754	71	62.59	46.73	71.22	66
		1995	1,212	395	1,700	75	48.94	15.95	68.63	73
		1996	1,196	398	1,715	78	47.95	15.93	68.74	74
		1997	1,203	410	1,760	77	47.75	16.26	69.88	77
		1998	1,228	428	1,832	78	48.32	16.85	72.05	78
		1999	1,536	1,280	1,920	76	60.16	50.12	75.20	71
		2000	1,313	494	2,043	80	51.02	19.21	79.41	80
		2001	1,638	1,361	2,175	77	64.06	53.23	85.04	76
		2002	1,412	582	2,313	82	55.48	22.88	90.87	80
		2003	1,453	626	2,443	81	57.54	24.78	96.69	81
		2004	1,488	671	2,561	81	58.66	26.44	100.93	86
		2005	1,516	712	2,669	81	58.80	27.63	103.54	86
		2006	1,527	748	2,751	82	57.85	28.34	104.25	91
		2007	1,506	765	2,781	83	56.48	28.69	104.28	91
	Old (30-64)	1992	4,857	3,453	5,979	62	98.32	69.92	121.04	66
		1993	3,985	1,272	5,796	72	78.73	25.14	114.51	77
		1994	4,898	3,657	5,574	63	94.45	70.52	107.49	64
		1995	3,998	1,303	5,607	72	75.22	24.52	105.48	77
		1996	3,986	1,324	5,714	73	73.22	24.33	104.96	76
		1997	3,956	1,347	5,790	72	71.22	24.25	104.22	75
		1998	3,915	1,365	5,837	73	69.00	24.06	102.88	75
		1999	4,676	3,895	5,844	67	80.89	67.38	101.11	68
		2000	3,777	1,422	5,878	74	64.26	24.20	100.01	75
		2001	4,427	3,678	5,876	68	73.90	61.41	98.10	68
		2002	3,575	1,474	5,856	76	58.79	24.24	96.31	78
		2003	3,451	1,486	5,800	77	56.06	24.14	94.21	77
		2004	3,330	1,501	5,729	77	53.47	24.10	92.01	78
		2005	3,220	1,513	5,671	77	50.99	23.96	89.78	82
		2006	3,113	1,525	5,610	76	48.50	23.76	87.42	85
		2007	2,987	1,518	5,516	77	45.73	23.23	84.43	85

Metropolitan Statistical Area	PWID Population	Year	Number	Min	Max	Rank	Rate	Min	Max	Rank
Greenville--Spartanburg--Anderson, SC	Total	1992	2,831	1,855	3,329	91	49.28	32.29	57.95	97
		1993	2,274	749	3,386	94	39.11	12.88	58.23	98
		1994	2,742	1,841	3,455	92	46.55	31.25	58.67	97
		1995	2,318	1,132	3,539	93	38.72	18.91	59.10	98
		1996	2,352	1,327	3,632	92	38.65	21.80	59.67	99
		1997	2,385	1,515	3,721	92	38.60	24.53	60.23	98
		1998	2,426	1,717	3,818	92	38.60	27.32	60.74	98
		1999	2,655	1,881	3,918	91	41.50	29.40	61.24	97
		2000	2,472	1,822	4,079	92	38.08	28.07	62.83	98
		2001	2,717	1,906	4,229	92	41.39	29.03	64.41	97
		2002	2,511	1,745	4,385	92	37.90	26.33	66.18	98
		2003	2,534	1,663	4,540	93	37.93	24.89	67.95	98
		2004	2,575	1,556	4,708	93	38.14	23.05	69.72	98
		2005	2,624	1,458	4,886	94	38.39	21.34	71.49	99
		2006	2,689	1,373	5,094	95	38.67	19.74	73.26	99
		2007	2,762	1,287	5,319	95	38.96	18.16	75.02	99
	Non-Hispanic White	1992	1,587	1,040	1,866	92	33.99	22.28	39.97	95
		1993	1,326	436	1,974	95	28.17	9.27	41.94	96
		1994	1,651	1,108	2,081	89	34.77	23.35	43.83	89
		1995	1,435	701	2,190	93	29.89	14.59	45.62	92
		1996	1,490	841	2,301	89	30.66	17.30	47.34	91
		1997	1,541	979	2,405	88	31.40	19.96	49.00	89
		1998	1,595	1,129	2,510	88	32.07	22.70	50.47	89
		1999	1,771	1,255	2,614	86	35.19	24.93	51.94	92
		2000	1,670	1,231	2,756	88	32.90	24.26	54.29	91
		2001	1,857	1,303	2,890	86	36.33	25.48	56.54	92
		2002	1,733	1,204	3,027	92	33.77	23.46	58.97	94
		2003	1,766	1,159	3,163	93	34.26	22.48	61.38	95
		2004	1,809	1,093	3,307	93	34.90	21.09	63.79	95
		2005	1,858	1,033	3,459	93	35.58	19.78	66.25	95
		2006	1,918	979	3,633	88	36.27	18.52	68.71	95
		2007	1,984	924	3,820	87	36.99	17.23	71.21	91

Metropolitan Statistical Area	PWID Population	Year	Number	Min	Max	Rank	Rate	Min	Max	Rank
Greenville--Spartanburg--Anderson, SC	Non-Hispanic Black	1992	1,246	817	1,465	68	128.72	84.35	151.35	92
		1993	913	300	1,359	79	92.34	30.40	137.48	96
		1994	1,007	676	1,269	73	99.80	67.00	125.77	95
		1995	783	382	1,195	81	75.80	37.01	115.71	97
		1996	735	414	1,134	81	69.77	39.35	107.72	97
		1997	693	440	1,081	80	64.85	41.21	101.20	98
		1998	660	467	1,038	79	60.90	43.10	95.84	97
		1999	680	482	1,004	80	61.90	43.86	91.35	97
		2000	601	443	992	80	53.75	39.62	88.68	96
		2001	631	443	983	80	55.57	38.98	86.49	96
		2002	561	390	981	81	48.70	33.84	85.04	97
		2003	549	360	984	80	46.93	30.80	84.07	98
		2004	545	329	996	80	45.79	27.67	83.70	98
		2005	546	303	1,016	80	45.02	25.02	83.83	98
		2006	554	283	1,050	79	44.75	22.85	84.78	97
		2007	569	265	1,095	79	45.07	21.00	86.77	97
	Hispanic	1992	21	14	24	94	37.42	24.52	44.00	92
		1993	18	6	27	95	28.13	9.26	41.88	94
		1994	23	15	29	95	32.03	21.50	40.36	93
		1995	20	10	31	95	24.06	11.75	36.73	93
		1996	22	12	34	95	22.07	12.45	34.07	93
		1997	23	15	36	94	19.21	12.21	29.98	93
		1998	24	17	38	94	17.16	12.14	27.00	93
		1999	28	19	41	93	16.33	11.57	24.10	95
		2000	27	20	44	93	13.78	10.16	22.74	96
		2001	30	21	47	94	14.43	10.12	22.46	96
		2002	29	20	51	95	12.93	8.99	22.58	98
		2003	31	21	56	95	12.97	8.51	23.23	98
		2004	34	20	62	95	13.26	8.02	24.25	98
		2005	37	21	69	95	13.65	7.59	25.42	98
		2006	42	21	79	95	14.17	7.23	26.83	97
		2007	48	22	92	94	14.90	6.94	28.69	96

Metropolitan Statistical Area	PWID Population	Year	Number	Min	Max	Rank	Rate	Min	Max	Rank
Greenville--Spartanburg--Anderson, SC	Male	1992	2,036	1,334	2,393	91	71.85	47.08	84.48	96
		1993	1,620	533	2,412	93	56.52	18.60	84.14	98
		1994	1,930	1,295	2,432	92	66.51	44.65	83.82	96
		1995	1,609	786	2,456	93	54.57	26.65	83.30	98
		1996	1,607	907	2,481	91	53.59	30.23	82.74	96
		1997	1,601	1,018	2,499	91	52.58	33.41	82.05	97
		1998	1,599	1,132	2,517	91	51.56	36.49	81.13	97
		1999	1,716	1,216	2,533	91	54.19	38.39	79.96	97
		2000	1,567	1,155	2,585	91	48.65	35.86	80.27	98
		2001	1,688	1,184	2,628	91	51.86	36.38	80.71	97
		2002	1,531	1,064	2,673	92	46.57	32.36	81.31	98
		2003	1,518	996	2,719	93	45.77	30.04	82.00	99
		2004	1,518	917	2,775	94	45.27	27.36	82.76	99
		2005	1,527	849	2,843	95	44.95	24.99	83.71	99
		2006	1,550	791	2,936	97	44.87	22.91	85.00	100
		2007	1,584	738	3,049	97	44.95	20.95	86.55	100
	Female	1992	779	511	916	93	26.76	17.53	31.46	98
		1993	656	216	977	95	22.25	7.32	33.12	100
		1994	830	557	1,046	93	27.77	18.65	35.00	99
		1995	736	360	1,124	94	24.24	11.84	37.00	100
		1996	784	442	1,210	94	25.38	14.31	39.18	100
		1997	831	528	1,297	94	26.54	16.86	41.41	99
		1998	883	625	1,389	95	27.72	19.62	43.63	97
		1999	1,005	712	1,483	93	31.10	22.04	45.90	95
		2000	969	714	1,598	93	29.60	21.82	48.85	96
		2001	1,097	770	1,708	91	33.14	23.25	51.58	95
		2002	1,039	722	1,815	94	31.12	21.62	54.34	96
		2003	1,068	701	1,914	94	31.73	20.83	56.85	96
		2004	1,099	664	2,008	93	32.33	19.53	59.09	96
		2005	1,125	625	2,094	93	32.71	18.18	60.92	96
		2006	1,149	586	2,176	92	32.82	16.76	62.18	96
		2007	1,166	543	2,244	92	32.69	15.23	62.93	96

Metropolitan Statistical Area	PWID Population	Year	Number	Min	Max	Rank	Rate	Min	Max	Rank
Greenville--Spartanburg--Anderson, SC	Young (15-29)	1992	884	579	1,039	85	44.73	29.32	52.60	83
		1993	675	222	1,005	88	34.37	11.31	51.17	91
		1994	792	531	998	87	40.53	27.21	51.08	86
		1995	664	324	1,014	91	33.91	16.56	51.77	91
		1996	681	384	1,051	91	34.41	19.41	53.13	91
		1997	706	449	1,102	91	35.44	22.52	55.30	91
		1998	743	526	1,170	91	36.92	26.13	58.10	91
		1999	847	600	1,250	91	41.69	29.54	61.52	91
		2000	823	607	1,359	91	40.40	29.79	66.67	88
		2001	945	663	1,471	91	46.51	32.62	72.39	89
		2002	909	632	1,588	94	44.92	31.21	78.45	92
		2003	950	623	1,702	94	46.79	30.71	83.83	94
		2004	991	599	1,811	95	48.41	29.25	88.49	95
		2005	1,024	569	1,908	93	49.43	27.47	92.04	97
		2006	1,051	537	1,992	93	49.85	25.45	94.44	97
		2007	1,065	496	2,050	94	49.69	23.16	95.67	95
	Old (30-64)	1992	1,982	1,299	2,331	94	52.59	34.46	61.83	96
		1993	1,633	538	2,431	95	42.41	13.96	63.14	99
		1994	2,001	1,343	2,521	94	50.83	34.12	64.06	96
		1995	1,707	833	2,605	95	42.37	20.69	64.67	98
		1996	1,736	979	2,680	94	42.26	23.83	65.24	97
		1997	1,755	1,115	2,738	92	41.93	26.65	65.43	97
		1998	1,771	1,253	2,787	92	41.45	29.33	65.22	97
		1999	1,913	1,355	2,822	92	43.80	31.03	64.64	96
		2000	1,749	1,290	2,887	89	39.27	28.95	64.80	97
		2001	1,881	1,319	2,927	91	41.49	29.10	64.56	96
		2002	1,693	1,177	2,957	91	36.80	25.57	64.25	97
		2003	1,659	1,089	2,972	93	35.67	23.41	63.89	97
		2004	1,632	986	2,984	93	34.69	20.96	63.41	97
		2005	1,607	893	2,992	94	33.75	18.76	62.84	97
		2006	1,589	811	3,010	94	32.80	16.74	62.13	97
		2007	1,574	734	3,031	93	31.82	14.83	61.27	96

Metropolitan Statistical Area	PWID Population	Year	Number	Min	Max	Rank	Rate	Min	Max	Rank
Harrisburg--Lebanon--Carlisle, PA	Total	1992	3,480	1,948	4,341	86	87.58	49.02	109.24	64
		1993	3,241	1,773	4,627	86	81.07	44.36	115.75	63
		1994	3,990	1,997	5,126	84	99.24	49.65	127.48	51
		1995	3,688	1,974	5,649	84	91.33	48.88	139.91	53
		1996	3,949	2,056	6,271	84	97.13	50.57	154.24	49
		1997	4,253	2,085	7,091	83	104.30	51.12	173.89	46
		1998	4,486	2,144	7,549	83	109.26	52.21	183.85	44
		1999	5,337	2,200	7,486	77	129.28	53.30	181.33	32
		2000	4,610	2,331	6,973	82	111.16	56.21	168.14	41
		2001	5,134	2,464	7,027	80	123.17	59.13	168.61	33
		2002	4,371	2,647	7,438	83	103.96	62.96	176.89	42
		2003	4,446	2,769	7,875	84	104.76	65.24	185.57	41
		2004	4,778	2,852	8,317	82	111.85	66.76	194.67	35
		2005	5,098	2,943	8,807	79	118.21	68.25	204.23	29
		2006	5,463	3,043	9,346	76	125.18	69.73	214.16	24
		2007	5,848	3,139	9,872	73	132.78	71.27	224.14	22
	Non-Hispanic White	1992	1,677	939	2,092	89	46.89	26.24	58.48	80
		1993	1,561	854	2,229	89	43.54	23.82	62.16	78
		1994	1,963	982	2,521	84	54.60	27.32	70.14	66
		1995	1,882	1,007	2,883	82	52.31	28.00	80.14	66
		1996	2,111	1,099	3,352	80	58.56	30.49	92.99	60
		1997	2,393	1,173	3,989	76	66.40	32.55	110.71	56
		1998	2,655	1,269	4,468	74	73.54	35.14	123.74	55
		1999	3,312	1,365	4,646	71	91.58	37.75	128.45	35
		2000	2,981	1,507	4,508	71	82.36	41.65	124.58	44
		2001	3,431	1,647	4,696	66	94.58	45.40	129.47	36
		2002	2,991	1,811	5,089	71	82.01	49.67	139.54	42
		2003	3,081	1,919	5,457	72	83.99	52.31	148.78	40
		2004	3,313	1,977	5,767	69	90.00	53.72	156.65	39
		2005	3,486	2,012	6,022	68	94.05	54.30	162.49	36
		2006	3,618	2,015	6,189	66	96.87	53.96	165.72	40
		2007	3,664	1,966	6,185	66	97.49	52.33	164.57	40

Metropolitan Statistical Area	PWID Population	Year	Number	Min	Max	Rank	Rate	Min	Max	Rank
Harrisburg--Lebanon--Carlisle, PA	Non-Hispanic Black	1992	1,270	711	1,584	66	471.52	263.89	588.10	19
		1993	1,178	645	1,682	67	425.63	232.89	607.71	17
		1994	1,403	702	1,802	64	496.46	248.39	637.76	12
		1995	1,222	654	1,872	64	423.37	226.60	648.58	14
		1996	1,207	629	1,917	64	405.90	211.35	644.56	14
		1997	1,178	577	1,964	64	390.34	191.33	650.79	12
		1998	1,111	531	1,870	64	356.74	170.47	600.30	12
		1999	1,174	484	1,647	64	369.88	152.49	518.81	11
		2000	901	456	1,363	66	278.67	140.92	421.53	19
		2001	899	432	1,231	65	274.48	131.75	375.73	20
		2002	698	423	1,188	72	209.44	126.84	356.37	34
		2003	665	414	1,178	74	195.52	121.77	346.35	34
		2004	694	414	1,207	73	200.89	119.90	349.66	32
		2005	751	434	1,297	72	213.88	123.48	369.52	25
		2006	860	479	1,471	72	239.27	133.29	409.34	17
		2007	1,041	559	1,758	64	285.03	152.99	481.15	15
	Hispanic	1992	515	288	643	54	703.46	393.70	877.39	3
		1993	487	267	695	51	623.71	341.26	890.52	4
		1994	604	302	776	48	727.86	364.17	935.02	3
		1995	558	298	854	51	632.11	338.33	968.36	4
		1996	593	309	941	51	619.57	322.61	983.85	4
		1997	631	309	1,052	51	617.78	302.81	1029.98	4
		1998	654	313	1,101	51	598.55	286.02	1007.18	5
		1999	763	315	1,070	47	656.83	270.79	921.29	4
		2000	645	326	976	54	522.91	264.43	790.98	5
		2001	702	337	961	52	545.64	261.92	746.93	4
		2002	585	354	995	55	431.47	261.31	734.17	7
		2003	583	363	1,033	56	409.35	254.95	725.13	8
		2004	617	368	1,074	56	411.09	245.36	715.52	7
		2005	651	376	1,125	56	413.45	238.70	714.32	7
		2006	696	388	1,191	56	415.11	231.25	710.18	7
		2007	750	403	1,267	54	427.23	229.31	721.20	5

Metropolitan Statistical Area	PWID Population	Year	Number	Min	Max	Rank	Rate	Min	Max	Rank
Harrisburg--Lebanon--Carlisle, PA	Male	1992	2,396	1,341	2,988	87	121.98	68.27	152.14	60
		1993	2,232	1,221	3,187	84	112.87	61.76	161.15	61
		1994	2,755	1,378	3,539	82	138.49	69.29	177.90	47
		1995	2,554	1,367	3,913	83	127.89	68.45	195.92	48
		1996	2,746	1,430	4,360	83	136.45	71.05	216.68	45
		1997	2,968	1,455	4,949	82	147.16	72.13	245.34	42
		1998	3,141	1,501	5,285	81	154.32	73.74	259.68	39
		1999	3,745	1,544	5,252	71	182.97	75.43	256.64	27
		2000	3,236	1,636	4,895	76	157.36	79.58	238.04	36
		2001	3,598	1,727	4,926	75	174.04	83.54	238.24	28
		2002	3,051	1,848	5,192	81	146.10	88.48	248.59	37
		2003	3,080	1,918	5,456	80	146.03	90.95	258.67	37
		2004	3,273	1,954	5,698	76	153.99	91.91	268.02	31
		2005	3,436	1,984	5,937	72	160.03	92.39	276.49	26
		2006	3,602	2,007	6,163	71	165.60	92.25	283.30	24
		2007	3,745	2,010	6,321	71	170.59	91.57	287.98	24
	Female	1992	1,210	677	1,509	84	60.19	33.69	75.08	63
		1993	1,114	610	1,591	84	55.17	30.19	78.77	65
		1994	1,355	678	1,741	84	66.70	33.37	85.69	58
		1995	1,236	662	1,894	85	60.59	32.43	92.82	60
		1996	1,307	681	2,076	85	63.65	33.14	101.07	55
		1997	1,391	682	2,319	83	67.49	33.08	112.52	53
		1998	1,452	694	2,443	84	70.12	33.50	117.98	52
		1999	1,714	706	2,404	82	82.31	33.93	115.45	44
		2000	1,473	745	2,228	84	70.47	35.63	106.59	51
		2001	1,640	787	2,244	83	78.07	37.47	106.87	44
		2002	1,402	849	2,386	86	66.24	40.12	112.72	56
		2003	1,440	897	2,551	85	67.47	42.02	119.51	49
		2004	1,573	939	2,738	85	73.28	43.74	127.55	42
		2005	1,717	992	2,967	82	79.32	45.79	137.04	34
		2006	1,898	1,057	3,247	80	86.72	48.31	148.36	26
		2007	2,112	1,134	3,566	74	95.62	51.32	161.41	21

Metropolitan Statistical Area	PWID Population	Year	Number	Min	Max	Rank	Rate	Min	Max	Rank
Harrisburg--Lebanon--Carlisle, PA	Young (15-29)	1992	924	517	1,152	84	72.27	40.45	90.14	63
		1993	849	465	1,213	83	67.26	36.80	96.03	59
		1994	1,072	536	1,377	81	85.96	43.01	110.42	49
		1995	1,047	560	1,604	79	84.77	45.37	129.87	52
		1996	1,211	631	1,924	77	98.56	51.32	156.50	42
		1997	1,429	701	2,383	71	117.30	57.50	195.57	33
		1998	1,662	794	2,796	66	136.85	65.40	230.28	19
		1999	2,177	897	3,053	56	181.41	74.79	254.45	9
		2000	2,056	1,040	3,110	58	173.25	87.61	262.06	13
		2001	2,475	1,188	3,388	53	209.95	100.78	287.40	7
		2002	2,244	1,359	3,819	56	189.13	114.54	321.81	11
		2003	2,389	1,488	4,232	55	198.87	123.86	352.27	9
		2004	2,637	1,574	4,590	53	218.00	130.12	379.44	8
		2005	2,827	1,632	4,884	49	230.21	132.91	397.75	6
		2006	2,968	1,653	5,078	46	237.05	132.05	405.54	7
		2007	3,017	1,619	5,093	46	237.83	127.66	401.49	6
	Old (30-64)	1992	2,716	1,520	3,387	84	100.74	56.38	125.65	64
		1993	2,506	1,371	3,578	85	91.63	50.14	130.83	64
		1994	3,034	1,518	3,897	83	109.37	54.72	140.50	54
		1995	2,735	1,464	4,190	85	97.59	52.23	149.50	58
		1996	2,835	1,476	4,502	85	99.93	52.04	158.69	55
		1997	2,931	1,437	4,887	84	102.51	50.25	170.91	51
		1998	2,945	1,407	4,955	84	101.83	48.66	171.36	52
		1999	3,313	1,366	4,646	80	113.12	46.64	158.67	44
		2000	2,690	1,360	4,069	86	90.88	45.96	137.47	57
		2001	2,809	1,348	3,845	84	93.97	45.11	128.64	54
		2002	2,243	1,359	3,817	87	74.33	45.01	126.47	60
		2003	2,150	1,339	3,809	87	70.68	44.02	125.21	63
		2004	2,199	1,312	3,828	87	71.80	42.85	124.97	61
		2005	2,264	1,307	3,912	86	73.40	42.38	126.81	58
		2006	2,387	1,330	4,084	86	76.70	42.73	131.22	52
		2007	2,573	1,381	4,344	84	82.07	44.05	138.54	47

Metropolitan Statistical Area	PWID Population	Year	Number	Min	Max	Rank	Rate	Min	Max	Rank
Hartford, CT	Total	1992	11,278	10,339	12,839	41	150.42	137.90	171.24	33
		1993	11,370	10,426	13,001	36	152.74	140.05	174.65	29
		1994	11,603	10,546	13,137	39	156.47	142.21	177.15	27
		1995	11,469	10,647	13,196	35	155.40	144.25	178.79	24
		1996	11,433	10,425	12,726	33	154.65	141.02	172.14	21
		1997	11,323	10,198	12,132	34	152.63	137.46	163.53	22
		1998	11,150	9,970	12,547	36	149.75	133.90	168.51	22
		1999	11,412	9,737	13,000	35	152.31	129.95	173.50	21
		2000	10,957	8,863	13,544	38	144.78	117.11	178.96	20
		2001	11,469	8,229	14,089	38	150.11	107.70	184.42	19
		2002	10,997	7,799	14,726	38	142.19	100.85	190.42	19
		2003	11,096	7,535	15,359	39	141.89	96.36	196.42	19
		2004	11,134	7,180	15,963	39	141.59	91.31	203.01	19
		2005	11,175	6,729	16,627	39	140.87	84.82	209.59	20
		2006	11,216	6,279	17,294	38	140.17	78.47	216.13	20
		2007	11,209	5,777	17,907	39	139.37	71.83	222.66	20
	Non-Hispanic White	1992	3,706	3,397	4,218	61	60.03	55.04	68.34	64
		1993	3,722	3,413	4,256	59	61.21	56.13	69.99	58
		1994	3,805	3,458	4,307	60	63.22	57.46	71.58	57
		1995	3,784	3,512	4,353	55	63.52	58.97	73.08	57
		1996	3,810	3,474	4,240	55	64.30	58.63	71.57	56
		1997	3,824	3,444	4,097	55	64.77	58.33	69.39	57
		1998	3,827	3,423	4,307	52	65.03	58.15	73.18	58
		1999	3,992	3,406	4,547	52	67.86	57.90	77.31	59
		2000	3,912	3,164	4,836	52	66.29	53.63	81.95	58
		2001	4,185	3,003	5,142	55	70.62	50.67	86.76	57
		2002	4,106	2,912	5,498	48	68.81	48.81	92.15	58
		2003	4,240	2,879	5,869	46	70.69	48.01	97.86	56
		2004	4,354	2,808	6,243	46	72.53	46.77	103.98	53
		2005	4,471	2,692	6,652	48	74.18	44.66	110.36	52
		2006	4,585	2,567	7,070	52	75.91	42.49	117.04	51
		2007	4,676	2,410	7,471	52	77.46	39.92	123.75	49

Metropolitan Statistical Area	PWID Population	Year	Number	Min	Max	Rank	Rate	Min	Max	Rank
Hartford, CT	Non-Hispanic Black	1992	2,145	1,966	2,442	53	332.09	304.45	378.04	43
		1993	2,136	1,959	2,443	47	325.33	298.31	371.98	39
		1994	2,118	1,925	2,398	48	317.89	288.92	359.92	40
		1995	2,006	1,862	2,307	46	298.71	277.28	343.68	37
		1996	1,896	1,728	2,110	48	277.77	253.29	309.18	38
		1997	1,767	1,592	1,894	49	253.88	228.65	272.01	39
		1998	1,633	1,460	1,837	54	228.77	204.56	257.44	43
		1999	1,569	1,338	1,787	57	215.08	183.50	245.00	45
		2000	1,420	1,149	1,755	57	190.79	154.33	235.84	44
		2001	1,413	1,014	1,736	60	186.97	134.15	229.70	46
		2002	1,305	926	1,748	60	169.43	120.17	226.89	46
		2003	1,290	876	1,786	59	164.49	111.70	227.70	48
		2004	1,297	836	1,859	58	162.84	105.01	233.46	44
		2005	1,338	805	1,990	57	164.76	99.21	245.15	44
		2006	1,422	796	2,193	55	171.98	96.27	265.17	44
		2007	1,559	803	2,491	53	186.04	95.88	297.22	41
	Hispanic	1992	4,958	4,545	5,644	16	946.46	867.68	1077.43	2
		1993	4,826	4,425	5,518	16	890.50	816.54	1018.22	2
		1994	4,856	4,413	5,498	15	865.50	786.63	979.91	2
		1995	4,814	4,468	5,538	14	839.25	779.05	965.59	2
		1996	4,872	4,443	5,423	14	814.35	742.56	906.43	2
		1997	4,938	4,447	5,291	14	793.57	714.69	850.23	2
		1998	4,991	4,463	5,616	14	777.54	695.28	874.99	2
		1999	5,236	4,468	5,965	14	786.25	670.84	895.65	2
		2000	5,127	4,147	6,337	14	737.24	596.36	911.30	2
		2001	5,424	3,891	6,663	15	753.38	540.54	925.54	2
		2002	5,191	3,682	6,951	14	696.83	494.22	933.15	2
		2003	5,141	3,491	7,117	14	665.77	452.11	921.60	2
		2004	4,955	3,195	7,104	15	627.74	404.81	900.01	2
		2005	4,642	2,795	6,906	15	571.88	344.34	850.88	2
		2006	4,188	2,345	6,458	16	497.91	278.73	767.70	3
		2007	3,582	1,846	5,723	21	413.67	213.20	660.89	6

Metropolitan Statistical Area	PWID Population	Year	Number	Min	Max	Rank	Rate	Min	Max	Rank
Hartford, CT	Male	1992	8,762	8,033	9,974	36	236.51	216.82	269.23	22
		1993	8,667	7,947	9,910	34	235.73	216.15	269.54	20
		1994	8,701	7,908	9,851	36	237.48	215.84	268.87	20
		1995	8,486	7,878	9,764	32	233.05	216.33	268.13	16
		1996	8,371	7,633	9,318	32	229.55	209.31	255.50	15
		1997	8,227	7,409	8,815	32	224.82	202.47	240.87	12
		1998	8,060	7,207	9,070	34	219.51	196.29	247.02	11
		1999	8,225	7,018	9,370	34	222.67	189.98	253.65	15
		2000	7,888	6,381	9,751	34	211.38	170.98	261.28	14
		2001	8,259	5,926	10,146	34	219.12	157.21	269.19	15
		2002	7,929	5,623	10,618	34	207.54	147.19	277.92	15
		2003	8,014	5,442	11,094	34	207.28	140.76	286.93	14
		2004	8,056	5,195	11,551	34	207.08	133.54	296.90	14
		2005	8,098	4,876	12,049	33	205.92	123.99	306.39	14
		2006	8,133	4,553	12,540	34	205.22	114.88	316.43	13
		2007	8,123	4,186	12,977	35	203.72	105.00	325.48	13
	Female	1992	2,634	2,414	2,998	59	69.44	63.66	79.04	53
		1993	2,787	2,556	3,187	49	73.98	67.84	84.59	44
		1994	2,962	2,692	3,354	51	78.96	71.76	89.39	45
		1995	3,027	2,810	3,483	48	80.95	75.14	93.13	41
		1996	3,098	2,825	3,449	47	82.71	75.42	92.06	40
		1997	3,131	2,820	3,355	46	83.31	75.03	89.26	40
		1998	3,129	2,798	3,521	45	82.91	74.14	93.30	41
		1999	3,234	2,759	3,684	48	85.12	72.62	96.96	39
		2000	3,120	2,524	3,856	44	81.32	65.78	100.52	39
		2001	3,267	2,344	4,014	45	84.41	60.56	103.70	37
		2002	3,122	2,215	4,181	46	79.80	56.59	106.86	36
		2003	3,129	2,125	4,331	46	79.15	53.75	109.57	36
		2004	3,109	2,005	4,457	46	78.25	50.46	112.18	37
		2005	3,081	1,855	4,584	47	77.01	46.37	114.58	38
		2006	3,046	1,705	4,697	49	75.42	42.22	116.29	39
		2007	2,994	1,543	4,783	52	73.82	38.05	117.94	38

Metropolitan Statistical Area	PWID Population	Year	Number	Min	Max	Rank	Rate	Min	Max	Rank
Hartford, CT	Young (15-29)	1992	3,173	2,909	3,613	38	131.15	120.23	149.30	25
		1993	3,042	2,790	3,479	36	130.52	119.68	149.24	24
		1994	2,993	2,720	3,388	38	132.64	120.55	150.18	21
		1995	2,889	2,682	3,324	36	131.84	122.38	151.69	22
		1996	2,848	2,597	3,170	35	132.57	120.89	147.56	20
		1997	2,822	2,541	3,023	35	133.03	119.80	142.52	19
		1998	2,809	2,512	3,161	37	133.50	119.38	150.23	23
		1999	2,933	2,503	3,341	38	140.55	119.92	160.10	19
		2000	2,897	2,344	3,581	39	138.86	112.33	171.64	20
		2001	3,141	2,254	3,859	39	149.54	107.29	183.71	21
		2002	3,137	2,225	4,201	39	146.48	103.89	196.16	20
		2003	3,312	2,249	4,584	38	151.29	102.74	209.42	20
		2004	3,487	2,249	4,999	37	157.04	101.27	225.16	20
		2005	3,679	2,215	5,474	36	162.20	97.66	241.33	17
		2006	3,884	2,174	5,988	36	167.81	93.94	258.73	18
		2007	4,079	2,102	6,517	35	173.97	89.67	277.95	17
	Old (30-64)	1992	8,179	7,499	9,311	39	161.08	147.67	183.37	36
		1993	8,310	7,620	9,502	39	162.52	149.02	185.83	31
		1994	8,545	7,766	9,675	39	165.62	150.53	187.52	31
		1995	8,505	7,895	9,786	36	163.90	152.15	188.58	26
		1996	8,526	7,774	9,490	35	162.58	148.25	180.96	24
		1997	8,475	7,633	9,080	35	159.99	144.08	171.41	24
		1998	8,354	7,470	9,401	36	156.38	139.84	175.98	23
		1999	8,531	7,279	9,718	36	157.81	134.65	179.77	23
		2000	8,141	6,585	10,062	36	148.49	120.12	183.55	22
		2001	8,428	6,047	10,354	37	152.14	109.16	186.90	24
		2002	7,948	5,637	10,643	35	142.13	100.81	190.34	21
		2003	7,833	5,319	10,843	36	139.12	94.47	192.57	21
		2004	7,611	4,908	10,912	35	134.88	86.98	193.38	21
		2005	7,315	4,404	10,883	35	129.13	77.75	192.13	21
		2006	6,927	3,878	10,680	34	121.79	68.18	187.79	22
		2007	6,406	3,302	10,234	36	112.42	57.94	179.61	24

Metropolitan Statistical Area	PWID Population	Year	Number	Min	Max	Rank	Rate	Min	Max	Rank
Honolulu,HI	Total	1992	5,074	3,839	6,339	76	86.76	65.64	108.41	66
		1993	4,648	3,112	6,479	79	79.56	53.28	110.91	67
		1994	5,268	3,967	6,651	77	89.90	67.69	113.50	64
		1995	4,670	2,615	6,796	80	79.78	44.66	116.09	67
		1996	4,683	2,381	6,959	81	79.98	40.66	118.85	64
		1997	4,725	2,169	7,162	82	80.23	36.83	121.61	64
		1998	4,756	1,950	7,353	81	80.47	33.00	124.40	63
		1999	5,727	4,216	7,441	70	97.89	72.07	127.20	53
		2000	4,738	1,465	7,742	81	81.26	25.13	132.79	61
		2001	5,976	4,153	8,103	69	102.06	70.94	138.38	48
		2002	4,948	1,332	8,627	79	83.97	22.61	146.40	58
		2003	5,056	1,263	9,139	77	85.43	21.35	154.42	56
		2004	5,188	1,201	9,711	76	86.96	20.14	162.77	55
		2005	5,328	1,148	10,300	75	88.53	19.07	171.12	55
		2006	5,455	1,090	10,884	77	90.15	18.01	179.89	53
		2007	5,500	1,022	11,302	76	91.81	17.06	188.66	50
	Non-Hispanic White	1992	2,722	2,059	3,401	72	154.57	116.95	193.13	18
		1993	2,493	1,669	3,475	72	145.44	97.39	202.75	18
		1994	2,830	2,131	3,574	73	168.07	126.55	212.20	13
		1995	2,515	1,408	3,661	74	153.46	85.92	223.32	12
		1996	2,529	1,286	3,758	73	158.29	80.47	235.23	8
		1997	2,556	1,173	3,874	74	163.38	75.00	247.65	6
		1998	2,573	1,055	3,978	75	167.64	68.74	259.18	5
		1999	3,091	2,276	4,017	73	208.18	153.26	270.51	4
		2000	2,544	787	4,158	78	175.95	54.42	287.51	5
		2001	3,180	2,210	4,311	68	217.61	151.24	295.04	4
		2002	2,597	699	4,527	77	176.03	47.39	306.91	6
		2003	2,602	650	4,703	77	177.32	44.31	320.53	7
		2004	2,600	602	4,868	77	174.58	40.43	326.80	7
		2005	2,581	556	4,989	80	170.48	36.73	329.52	8
		2006	2,531	506	5,050	81	165.32	33.03	329.87	8
		2007	2,419	450	4,972	81	162.63	30.23	334.21	8

Metropolitan Statistical Area	PWID Population	Year	Number	Min	Max	Rank	Rate	Min	Max	Rank
Honolulu,HI	Non-Hispanic Black	1992	320	242	400	98	159.39	120.60	199.16	86
		1993	258	173	360	98	131.54	88.08	183.38	89
		1994	261	197	330	98	132.21	99.55	166.93	91
		1995	209	117	305	98	107.31	60.08	156.16	92
		1996	193	98	287	98	101.15	51.42	150.31	92
		1997	182	83	275	98	96.22	44.17	145.85	92
		1998	173	71	268	97	92.95	38.12	143.71	89
		1999	201	148	261	96	115.33	84.91	149.86	83
		2000	163	50	266	97	92.38	28.57	150.96	88
		2001	205	142	278	96	103.70	72.07	140.60	82
		2002	172	46	300	96	80.64	21.71	140.59	88
		2003	182	45	329	97	84.88	21.21	153.44	85
		2004	196	46	368	97	90.99	21.07	170.33	79
		2005	217	47	419	97	92.10	19.85	178.03	76
		2006	243	49	485	97	98.37	19.65	196.29	73
		2007	275	51	565	96	114.08	21.20	234.43	66
	Hispanic	1992	200	151	250	68	53.55	40.52	66.91	84
		1993	203	136	283	67	54.37	36.40	75.79	78
		1994	250	188	315	63	67.03	50.47	84.63	74
		1995	236	132	344	62	64.07	35.87	93.23	69
		1996	249	127	371	62	67.03	34.08	99.61	63
		1997	261	120	396	62	69.05	31.70	104.66	62
		1998	270	111	417	62	71.43	29.29	110.43	58
		1999	331	243	430	60	90.11	66.34	117.09	46
		2000	276	85	451	63	75.49	23.35	123.35	53
		2001	349	243	474	62	91.63	63.68	124.23	44
		2002	289	78	504	63	73.03	19.66	127.32	53
		2003	295	74	534	64	73.06	18.26	132.07	52
		2004	303	70	567	64	73.20	16.95	137.03	53
		2005	312	67	604	64	72.76	15.68	140.64	52
		2006	323	65	644	65	73.34	14.65	146.33	52
		2007	332	62	683	66	76.16	14.15	156.50	51

Metropolitan Statistical Area	PWID Population	Year	Number	Min	Max	Rank	Rate	Min	Max	Rank
Honolulu,HI	Male	1992	3,725	2,819	4,655	73	123.41	93.37	154.20	59
		1993	3,307	2,215	4,611	76	109.87	73.57	153.17	64
		1994	3,656	2,753	4,616	74	120.97	91.09	152.73	57
		1995	3,181	1,781	4,629	80	105.45	59.04	153.46	66
		1996	3,151	1,602	4,683	80	104.62	53.19	155.47	63
		1997	3,160	1,450	4,790	79	104.60	48.01	158.54	60
		1998	3,176	1,302	4,911	80	104.66	42.92	161.81	58
		1999	3,835	2,823	4,983	68	127.96	94.21	166.28	47
		2000	3,190	987	5,213	77	106.66	32.99	174.28	57
		2001	4,052	2,816	5,494	67	134.40	93.41	182.23	44
		2002	3,380	910	5,894	74	111.43	30.00	194.29	55
		2003	3,478	869	6,287	72	114.80	28.69	207.52	52
		2004	3,588	831	6,716	69	117.46	27.20	219.87	49
		2005	3,695	796	7,142	67	119.09	25.66	230.19	46
		2006	3,779	755	7,541	69	120.85	24.14	241.14	46
		2007	3,790	704	7,788	69	123.02	22.86	252.79	40
	Female	1992	1,325	1,003	1,656	83	46.85	35.45	58.54	79
		1993	1,323	886	1,845	81	46.73	31.29	65.15	75
		1994	1,601	1,206	2,022	80	56.42	42.48	71.23	67
		1995	1,489	834	2,167	81	52.48	29.38	76.37	67
		1996	1,543	785	2,294	82	54.28	27.59	80.66	64
		1997	1,589	729	2,409	81	55.40	25.43	83.97	64
		1998	1,615	662	2,497	80	56.16	23.03	86.82	64
		1999	1,946	1,432	2,528	75	68.19	50.20	88.60	54
		2000	1,599	494	2,612	82	56.30	17.41	92.00	64
		2001	1,991	1,384	2,699	76	70.08	48.71	95.02	53
		2002	1,620	436	2,824	82	56.65	15.25	98.77	63
		2003	1,622	405	2,932	84	56.14	14.03	101.48	63
		2004	1,628	377	3,048	84	55.92	12.95	104.68	64
		2005	1,637	353	3,164	84	56.12	12.09	108.48	63
		2006	1,643	328	3,278	84	56.20	11.23	112.15	62
		2007	1,631	303	3,351	82	56.03	10.41	115.15	62

Metropolitan Statistical Area	PWID Population	Year	Number	Min	Max	Rank	Rate	Min	Max	Rank
Honolulu,HI	Young (15-29)	1992	973	736	1,215	82	47.27	35.77	59.07	81
		1993	828	554	1,154	84	41.15	27.56	57.37	82
		1994	887	668	1,119	84	44.77	33.71	56.53	83
		1995	756	423	1,100	87	38.82	21.73	56.49	85
		1996	740	376	1,099	89	38.54	19.59	57.27	85
		1997	740	340	1,121	89	38.76	17.79	58.75	88
		1998	747	306	1,156	89	39.18	16.07	60.57	88
		1999	914	673	1,188	88	48.72	35.87	63.31	83
		2000	776	240	1,269	93	41.48	12.83	67.77	86
		2001	1,014	705	1,374	88	54.24	37.70	73.54	82
		2002	875	236	1,526	95	46.78	12.60	81.56	89
		2003	938	234	1,695	95	50.42	12.60	91.14	88
		2004	1,013	235	1,897	93	54.08	12.52	101.23	88
		2005	1,098	237	2,123	91	57.53	12.40	111.21	87
		2006	1,188	237	2,371	91	61.47	12.28	122.66	87
		2007	1,265	235	2,600	86	67.28	12.50	138.26	79
	Old (30-64)	1992	4,143	3,134	5,176	71	109.29	82.69	136.56	54
		1993	3,838	2,570	5,351	76	100.19	67.09	139.67	58
		1994	4,393	3,308	5,547	68	113.24	85.26	142.97	48
		1995	3,926	2,198	5,714	74	100.47	56.25	146.21	56
		1996	3,962	2,014	5,888	74	100.67	51.18	149.59	53
		1997	4,015	1,843	6,085	71	100.84	46.29	152.85	53
		1998	4,049	1,660	6,259	71	101.14	41.47	156.37	53
		1999	4,872	3,586	6,330	64	122.60	90.26	159.30	37
		2000	4,016	1,242	6,563	68	101.46	31.38	165.78	48
		2001	5,031	3,497	6,821	59	126.21	87.72	171.12	37
		2002	4,122	1,110	7,186	66	102.47	27.59	178.66	43
		2003	4,147	1,036	7,496	65	102.18	25.53	184.71	42
		2004	4,166	965	7,798	64	101.78	23.57	190.52	40
		2005	4,156	895	8,033	63	101.12	21.79	195.46	39
		2006	4,089	817	8,159	62	99.30	19.84	198.15	36
		2007	3,905	726	8,025	62	95.01	17.66	195.23	35

Metropolitan Statistical Area	PWID Population	Year	Number	Min	Max	Rank	Rate	Min	Max	Rank
Houston, TX	Total	1992	57,770	50,145	66,832	3	240.75	208.98	278.52	7
		1993	43,653	7,109	63,659	4	178.38	29.05	260.13	20
		1994	53,637	47,037	60,321	3	215.07	188.60	241.87	11
		1995	40,717	8,520	57,131	5	160.13	33.51	224.67	22
		1996	39,285	9,330	54,112	6	151.51	35.98	208.70	24
		1997	38,015	10,278	51,445	6	143.25	38.73	193.86	27
		1998	36,626	11,292	49,026	6	134.53	41.48	180.08	29
		1999	42,836	37,980	46,732	6	153.34	135.95	167.28	19
		2000	34,406	13,615	44,421	6	120.36	47.63	155.39	38
		2001	39,911	33,538	43,881	6	136.14	114.40	149.69	27
		2002	34,195	16,884	48,637	6	113.46	56.02	161.38	38
		2003	34,492	18,539	53,283	6	112.04	60.22	173.08	38
		2004	34,942	20,198	58,670	6	111.15	64.25	186.64	38
		2005	35,491	21,124	64,320	6	110.46	65.75	200.19	34
		2006	36,571	17,781	71,190	6	109.99	53.48	214.10	32
		2007	37,131	13,971	77,305	6	109.52	41.21	228.01	33
	Non-Hispanic White	1992	25,378	22,028	29,359	3	189.45	164.45	219.17	12
		1993	18,762	3,056	27,361	6	140.00	22.80	204.15	20
		1994	22,499	19,731	25,303	4	167.93	147.27	188.86	14
		1995	16,642	3,482	23,351	7	124.32	26.01	174.43	22
		1996	15,635	3,713	21,537	7	117.05	27.80	161.22	23
		1997	14,739	3,985	19,946	7	110.07	29.76	148.95	27
		1998	13,856	4,272	18,547	9	102.90	31.73	137.74	32
		1999	15,857	14,060	17,299	6	117.20	103.91	127.86	23
		2000	12,515	4,953	16,158	11	92.35	36.55	119.24	39
		2001	14,346	12,055	15,773	8	104.78	88.05	115.20	31
		2002	12,229	6,039	17,395	12	88.16	43.53	125.40	40
		2003	12,374	6,651	19,116	13	88.65	47.65	136.94	38
		2004	12,691	7,336	21,309	13	90.31	52.21	151.64	38
		2005	13,179	7,844	23,885	12	93.15	55.44	168.82	38
		2006	14,029	6,821	27,308	12	97.98	47.64	190.72	36
		2007	14,863	5,592	30,943	11	103.30	38.87	215.06	33

Metropolitan Statistical Area	PWID Population	Year	Number	Min	Max	Rank	Rate	Min	Max	Rank
Houston, TX	Non-Hispanic Black	1992	22,764	19,759	26,334	2	545.11	473.16	630.62	14
		1993	17,631	2,871	25,711	5	414.62	67.52	604.63	19
		1994	22,019	19,309	24,762	3	507.42	444.98	570.66	9
		1995	16,868	3,530	23,667	6	381.11	79.75	534.74	17
		1996	16,322	3,876	22,483	6	362.53	86.10	499.36	17
		1997	15,759	4,261	21,327	7	343.01	92.74	464.20	18
		1998	15,084	4,651	20,191	7	321.06	98.99	429.76	17
		1999	17,465	15,485	19,053	6	363.46	322.26	396.51	12
		2000	13,851	5,481	17,882	8	283.07	112.02	365.46	17
		2001	15,837	13,308	17,412	6	316.49	265.95	347.97	13
		2002	13,364	6,599	19,009	8	260.71	128.73	370.82	16
		2003	13,282	7,139	20,518	7	254.11	136.58	392.55	17
		2004	13,276	7,674	22,292	6	248.72	143.77	417.62	17
		2005	13,342	7,941	24,179	5	244.21	145.35	442.58	18
		2006	13,657	6,640	26,585	4	232.33	112.96	452.26	19
		2007	13,848	5,210	28,832	5	233.45	87.84	486.04	23
	Hispanic	1992	8,537	7,410	9,876	7	160.21	139.06	185.34	40
		1993	6,023	981	8,783	12	106.81	17.39	155.76	54
		1994	7,154	6,274	8,046	8	120.27	105.47	135.26	46
		1995	5,408	1,132	7,588	13	86.13	18.02	120.84	57
		1996	5,320	1,264	7,328	13	80.09	19.02	110.32	55
		1997	5,342	1,444	7,230	13	75.87	20.51	102.68	54
		1998	5,403	1,666	7,233	12	72.36	22.31	96.86	57
		1999	6,672	5,915	7,278	12	84.35	74.78	92.02	55
		2000	5,658	2,239	7,304	13	67.68	26.78	87.38	58
		2001	6,893	5,792	7,579	8	78.57	66.02	86.39	57
		2002	6,140	3,032	8,734	11	66.66	32.91	94.81	58
		2003	6,344	3,410	9,800	11	66.06	35.51	102.05	58
		2004	6,454	3,731	10,836	11	64.62	37.36	108.51	59
		2005	6,423	3,823	11,641	11	61.77	36.76	111.94	60
		2006	6,295	3,061	12,255	11	58.00	28.20	112.91	61
		2007	5,868	2,208	12,217	12	51.89	19.53	108.04	63

Metropolitan Statistical Area	PWID Population	Year	Number	Min	Max	Rank	Rate	Min	Max	Rank
Houston, TX	Male	1992	37,638	32,670	43,542	4	312.32	271.09	361.31	11
		1993	28,214	4,595	41,144	4	229.64	37.40	334.89	22
		1994	34,558	30,305	38,864	4	275.74	241.81	310.10	13
		1995	26,268	5,497	36,857	5	205.80	43.06	288.76	27
		1996	25,478	6,051	35,094	5	195.75	46.49	269.63	28
		1997	24,867	6,723	33,653	6	186.64	50.46	252.58	28
		1998	24,231	7,471	32,434	6	177.31	54.67	237.34	31
		1999	28,720	25,465	31,332	6	204.81	181.59	223.43	19
		2000	23,412	9,265	30,226	6	163.10	64.54	210.58	33
		2001	27,584	23,180	30,328	6	187.17	157.28	205.79	23
		2002	24,012	11,856	34,154	6	158.21	78.12	225.03	33
		2003	24,603	13,224	38,007	6	158.65	85.27	245.08	28
		2004	25,302	14,626	42,484	6	159.70	92.31	268.15	28
		2005	26,064	15,513	47,236	6	160.77	95.69	291.37	24
		2006	27,203	13,227	52,954	6	162.30	78.91	315.94	26
		2007	27,932	10,510	58,154	6	163.28	61.43	339.94	26
	Female	1992	20,649	17,923	23,888	3	172.88	150.06	200.00	6
		1993	15,747	2,564	22,964	3	129.22	21.04	188.45	15
		1994	19,381	16,996	21,797	3	156.22	136.99	175.68	8
		1995	14,640	3,063	20,541	4	115.60	24.19	162.20	17
		1996	13,971	3,318	19,244	6	108.19	25.70	149.03	20
		1997	13,301	3,596	18,000	6	100.66	27.22	136.23	22
		1998	12,550	3,869	16,800	6	92.57	28.54	123.91	28
		1999	14,318	12,695	15,621	6	102.91	91.25	112.27	22
		2000	11,181	4,425	14,436	7	78.56	31.09	101.42	44
		2001	12,577	10,569	13,828	8	86.28	72.50	94.86	36
		2002	10,429	5,150	14,834	11	69.71	34.42	99.16	51
		2003	10,171	5,467	15,712	12	66.57	35.78	102.84	52
		2004	9,958	5,757	16,721	12	63.87	36.92	107.24	54
		2005	9,783	5,823	17,730	12	61.46	36.58	111.39	56
		2006	9,767	4,749	19,012	12	59.23	28.80	115.30	58
		2007	9,634	3,625	20,059	11	57.36	21.58	119.42	57

Metropolitan Statistical Area	PWID Population	Year	Number	Min	Max	Rank	Rate	Min	Max	Rank
Houston, TX	Young (15-29)	1992	16,802	14,584	19,438	3	202.02	175.35	233.71	8
		1993	11,971	1,950	17,458	4	143.59	23.38	209.39	18
		1994	14,105	12,369	15,862	3	168.27	147.57	189.24	13
		1995	10,435	2,184	14,641	5	122.96	25.73	172.52	27
		1996	9,962	2,366	13,723	5	115.17	27.35	158.64	33
		1997	9,674	2,615	13,092	6	109.07	29.49	147.61	38
		1998	9,470	2,920	12,677	6	103.76	31.99	138.89	38
		1999	11,379	10,089	12,413	6	121.37	107.62	132.41	31
		2000	9,476	3,750	12,234	7	99.01	39.18	127.83	47
		2001	11,483	9,649	12,625	7	118.61	99.67	130.41	35
		2002	10,336	5,104	14,702	7	104.74	51.72	148.98	44
		2003	10,997	5,911	16,989	7	110.17	59.22	170.20	44
		2004	11,777	6,808	19,775	7	116.51	67.35	195.64	37
		2005	12,652	7,530	22,929	5	123.31	73.40	223.48	29
		2006	13,775	6,697	26,814	5	129.71	63.07	252.50	28
		2007	14,740	5,546	30,688	5	136.86	51.49	284.93	26
	Old (30-64)	1992	40,815	35,428	47,218	3	260.33	225.97	301.17	11
		1993	31,590	5,145	46,067	4	195.79	31.89	285.51	23
		1994	39,438	34,586	44,353	4	238.19	208.88	267.87	9
		1995	30,226	6,325	42,411	5	178.41	37.33	250.33	24
		1996	29,284	6,955	40,337	6	169.49	40.25	233.46	23
		1997	28,320	7,657	38,326	6	160.29	43.34	216.93	23
		1998	27,151	8,371	36,343	6	150.03	46.26	200.82	26
		1999	31,471	27,904	34,333	5	169.55	150.33	184.97	17
		2000	24,960	9,878	32,226	6	131.26	51.94	169.46	34
		2001	28,497	23,947	31,332	6	145.14	121.96	159.58	26
		2002	23,963	11,832	34,084	7	118.22	58.38	168.16	35
		2003	23,671	12,723	36,567	7	113.79	61.16	175.78	33
		2004	23,446	13,553	39,368	7	109.93	63.55	184.59	29
		2005	23,262	13,846	42,158	7	106.37	63.31	192.78	29
		2006	23,407	11,381	45,565	6	103.43	50.29	201.34	32
		2007	23,213	8,734	48,329	6	100.34	37.75	208.91	32

Metropolitan Statistical Area	PWID Population	Year	Number	Min	Max	Rank	Rate	Min	Max	Rank
Indianapolis, IN	Total	1992	9,805	5,982	11,734	46	102.16	62.32	122.26	52
		1993	8,698	5,686	12,026	45	89.10	58.25	123.20	56
		1994	9,307	5,377	12,247	47	93.91	54.25	123.56	55
		1995	8,115	5,055	12,342	47	80.67	50.25	122.70	65
		1996	7,709	4,747	11,952	48	75.47	46.47	117.01	68
		1997	7,246	4,411	11,358	56	70.12	42.69	109.92	70
		1998	6,759	4,074	10,636	61	64.66	38.98	101.76	74
		1999	7,148	3,737	10,470	61	67.45	35.26	98.80	76
		2000	6,484	3,719	11,270	64	60.23	34.55	104.69	75
		2001	7,532	3,697	12,591	56	68.93	33.83	115.22	73
		2002	7,016	4,040	14,096	54	63.34	36.47	127.26	75
		2003	7,322	3,964	15,462	52	65.28	35.35	137.86	73
		2004	7,380	3,880	15,791	53	64.92	34.13	138.91	73
		2005	7,036	3,789	14,486	54	61.07	32.89	125.74	77
		2006	6,703	3,702	13,117	63	57.29	31.64	112.10	81
		2007	6,298	3,595	11,488	71	53.09	30.30	96.83	91
	Non-Hispanic White	1992	4,649	2,836	5,564	51	57.07	34.81	68.29	69
		1993	4,169	2,725	5,764	53	50.49	33.00	69.81	66
		1994	4,537	2,621	5,970	51	54.34	31.39	71.50	67
		1995	4,041	2,517	6,146	52	47.91	29.84	72.86	69
		1996	3,931	2,420	6,094	53	46.10	28.39	71.48	73
		1997	3,786	2,305	5,935	57	44.13	26.86	69.17	75
		1998	3,616	2,180	5,691	56	41.89	25.25	65.92	78
		1999	3,908	2,043	5,724	56	44.88	23.46	65.74	77
		2000	3,610	2,070	6,274	57	41.02	23.53	71.30	79
		2001	4,252	2,087	7,107	54	47.79	23.45	79.88	75
		2002	3,993	2,300	8,023	52	44.45	25.60	89.32	77
		2003	4,175	2,261	8,817	47	46.10	24.96	97.35	78
		2004	4,184	2,200	8,952	51	45.79	24.07	97.97	81
		2005	3,930	2,116	8,092	59	42.63	22.95	87.77	86
		2006	3,651	2,016	7,143	65	39.19	21.64	76.68	88
		2007	3,303	1,885	6,025	73	35.13	20.05	64.08	94

Metropolitan Statistical Area	PWID Population	Year	Number	Min	Max	Rank	Rate	Min	Max	Rank
Indianapolis, IN	Non-Hispanic Black	1992	4,728	2,885	5,659	21	381.92	232.99	457.06	34
		1993	4,142	2,707	5,726	25	325.81	212.98	450.49	38
		1994	4,337	2,505	5,707	24	332.62	192.15	437.65	37
		1995	3,669	2,285	5,580	26	274.38	170.91	417.30	43
		1996	3,355	2,066	5,202	27	245.50	151.16	380.61	46
		1997	3,012	1,834	4,722	29	215.74	131.34	338.17	48
		1998	2,665	1,607	4,194	31	187.45	112.99	294.99	53
		1999	2,657	1,389	3,892	29	184.21	96.30	269.84	54
		2000	2,259	1,296	3,927	33	153.99	88.32	267.66	63
		2001	2,451	1,203	4,097	33	163.58	80.29	273.45	60
		2002	2,126	1,225	4,272	34	139.41	80.28	280.11	60
		2003	2,066	1,119	4,363	33	132.81	71.91	280.46	62
		2004	1,942	1,021	4,156	34	122.03	64.16	261.11	64
		2005	1,735	934	3,572	40	106.85	57.54	220.00	71
		2006	1,561	862	3,054	48	93.76	51.78	183.46	75
		2007	1,401	800	2,555	56	82.33	46.99	150.17	80
	Hispanic	1992	117	72	140	75	115.35	70.37	138.05	56
		1993	106	70	147	76	92.20	60.27	127.49	58
		1994	118	68	156	76	90.22	52.12	118.71	60
		1995	109	68	165	78	71.88	44.77	109.32	62
		1996	110	68	171	77	63.09	38.85	97.81	65
		1997	111	68	175	76	55.32	33.68	86.72	72
		1998	113	68	178	75	48.34	29.14	76.07	73
		1999	130	68	191	75	48.60	25.41	71.19	74
		2000	130	74	225	74	42.61	24.44	74.07	73
		2001	165	81	276	73	49.92	24.50	83.44	68
		2002	169	98	340	72	47.42	27.31	95.28	66
		2003	194	105	410	71	50.77	27.49	107.20	64
		2004	215	113	459	71	52.57	27.64	112.48	64
		2005	224	121	461	71	51.27	27.61	105.56	64
		2006	232	128	455	69	49.87	27.54	97.58	64
		2007	237	135	433	71	47.89	27.33	87.35	65

Metropolitan Statistical Area	PWID Population	Year	Number	Min	Max	Rank	Rate	Min	Max	Rank
Indianapolis, IN	Male	1992	7,086	4,323	8,481	41	150.90	92.06	180.59	46
		1993	6,116	3,998	8,457	43	127.86	83.58	176.79	52
		1994	6,380	3,686	8,395	42	131.25	75.82	172.70	52
		1995	5,435	3,386	8,267	45	110.02	68.53	167.32	60
		1996	5,059	3,115	7,844	48	100.73	62.02	156.17	66
		1997	4,673	2,845	7,325	55	91.87	55.93	144.01	71
		1998	4,299	2,591	6,765	60	83.42	50.28	131.28	75
		1999	4,499	2,352	6,590	61	85.95	44.93	125.90	77
		2000	4,053	2,324	7,044	62	76.13	43.66	132.32	77
		2001	4,692	2,303	7,844	56	86.78	42.59	145.06	74
		2002	4,371	2,517	8,783	56	79.69	45.89	160.11	73
		2003	4,579	2,479	9,669	54	82.35	44.59	173.89	71
		2004	4,647	2,443	9,943	54	82.37	43.31	176.25	69
		2005	4,474	2,409	9,211	57	78.22	42.12	161.05	75
		2006	4,317	2,384	8,446	59	74.30	41.03	145.38	77
		2007	4,117	2,350	7,509	62	69.83	39.86	127.37	82
	Female	1992	2,831	1,727	3,388	54	57.76	35.24	69.13	64
		1993	2,660	1,739	3,678	53	53.44	34.93	73.89	67
		1994	3,012	1,740	3,963	49	59.64	34.45	78.47	64
		1995	2,772	1,726	4,215	51	54.15	33.73	82.36	65
		1996	2,768	1,704	4,291	51	53.32	32.83	82.66	66
		1997	2,721	1,657	4,266	52	51.87	31.58	81.31	68
		1998	2,639	1,591	4,153	55	49.80	30.02	78.37	72
		1999	2,881	1,506	4,221	56	53.73	28.09	78.70	69
		2000	2,677	1,535	4,653	56	49.20	28.22	85.51	72
		2001	3,157	1,550	5,278	47	57.19	28.07	95.60	66
		2002	2,956	1,703	5,940	49	52.88	30.45	106.24	67
		2003	3,068	1,661	6,480	48	54.26	29.38	114.58	65
		2004	3,038	1,597	6,501	47	53.05	27.89	113.51	68
		2005	2,805	1,511	5,776	53	48.36	26.04	99.56	76
		2006	2,547	1,407	4,984	61	43.24	23.88	84.60	84
		2007	2,237	1,277	4,081	71	37.49	21.40	68.37	89

Metropolitan Statistical Area	PWID Population	Year	Number	Min	Max	Rank	Rate	Min	Max	Rank
Indianapolis, IN	Young (15-29)	1992	2,848	1,738	3,409	40	88.53	54.01	105.95	53
		1993	2,165	1,415	2,994	47	67.32	44.00	93.08	58
		1994	2,074	1,198	2,730	49	64.48	37.25	84.84	63
		1995	1,691	1,053	2,571	58	52.32	32.59	79.57	68
		1996	1,563	962	2,423	66	47.99	29.55	74.40	73
		1997	1,480	901	2,320	69	45.19	27.51	70.83	79
		1998	1,431	863	2,253	72	43.55	26.25	68.53	82
		1999	1,605	839	2,351	73	48.80	25.51	71.48	82
		2000	1,568	899	2,725	74	47.64	27.32	82.80	82
		2001	1,978	971	3,306	66	60.11	29.50	100.47	79
		2002	2,005	1,155	4,029	64	60.70	34.96	121.96	78
		2003	2,269	1,229	4,792	60	68.14	36.89	143.90	74
		2004	2,458	1,293	5,260	57	73.04	38.40	156.29	74
		2005	2,486	1,338	5,118	59	73.25	39.44	150.81	75
		2006	2,469	1,363	4,830	62	71.73	39.61	140.35	75
		2007	2,368	1,351	4,318	59	68.16	38.90	124.33	78
	Old (30-64)	1992	7,165	4,371	8,575	48	112.30	68.51	134.39	52
		1993	6,628	4,332	9,164	43	101.27	66.20	140.02	55
		1994	7,300	4,217	9,605	44	109.05	63.00	143.49	55
		1995	6,488	4,041	9,867	45	95.03	59.20	144.53	60
		1996	6,234	3,839	9,666	47	89.61	55.17	138.92	61
		1997	5,889	3,585	9,230	48	83.43	50.79	130.78	66
		1998	5,487	3,307	8,635	53	76.58	46.16	120.52	69
		1999	5,764	3,013	8,443	53	78.87	41.23	115.53	69
		2000	5,162	2,961	8,973	56	69.07	39.62	120.06	71
		2001	5,884	2,888	9,835	49	77.04	37.81	128.78	64
		2002	5,339	3,075	10,728	52	68.68	39.55	138.00	65
		2003	5,386	2,916	11,373	48	68.30	36.98	144.24	65
		2004	5,199	2,734	11,125	51	64.97	34.16	139.02	66
		2005	4,697	2,529	9,670	54	57.79	31.12	118.99	75
		2006	4,187	2,312	8,193	60	50.70	28.00	99.20	78
		2007	3,628	2,071	6,617	71	43.23	24.68	78.86	89

Metropolitan Statistical Area	PWID Population	Year	Number	Min	Max	Rank	Rate	Min	Max	Rank
Jacksonville, FL	Total	1992	10,368	7,581	14,532	43	163.83	119.79	229.63	25
		1993	7,883	1,894	13,218	49	123.37	29.65	206.87	39
		1994	9,408	7,909	11,923	45	145.46	122.28	184.35	32
		1995	7,312	2,244	10,785	54	111.10	34.10	163.88	44
		1996	7,189	2,433	9,929	57	105.48	35.70	145.68	47
		1997	7,004	2,584	9,656	59	99.91	36.86	137.74	48
		1998	7,036	2,720	10,448	57	98.35	38.02	146.04	48
		1999	8,563	6,757	11,487	48	117.71	92.89	157.91	39
		2000	7,365	3,063	12,562	52	99.39	41.33	169.53	50
		2001	8,956	6,133	13,259	46	118.08	80.86	174.80	38
		2002	7,340	3,067	12,622	51	94.38	39.44	162.29	51
		2003	6,882	3,055	10,735	55	86.72	38.49	135.26	55
		2004	6,447	3,008	9,350	63	79.06	36.89	114.66	61
		2005	5,979	2,956	10,025	72	71.64	35.42	120.10	67
		2006	5,507	2,901	10,727	74	64.42	33.94	125.49	73
		2007	5,003	2,413	11,362	80	57.63	27.79	130.87	82
	Non-Hispanic White	1992	5,867	4,290	8,223	41	122.01	89.21	171.02	27
		1993	4,478	1,076	7,508	51	92.83	22.31	155.66	37
		1994	5,338	4,488	6,766	43	110.01	92.48	139.42	29
		1995	4,132	1,268	6,095	51	84.26	25.86	124.29	47
		1996	4,041	1,368	5,581	49	80.17	27.14	110.73	51
		1997	3,916	1,445	5,399	53	76.09	28.07	104.90	52
		1998	3,922	1,516	5,824	51	75.09	29.03	111.50	54
		1999	4,776	3,769	6,407	44	90.50	71.41	121.40	37
		2000	4,133	1,719	7,049	49	77.37	32.17	131.96	49
		2001	5,091	3,486	7,536	40	93.61	64.10	138.57	38
		2002	4,260	1,780	7,325	46	76.87	32.12	132.19	51
		2003	4,113	1,826	6,415	49	73.16	32.47	114.11	53
		2004	4,002	1,868	5,805	55	69.77	32.56	101.19	56
		2005	3,887	1,921	6,516	61	66.62	32.94	111.69	61
		2006	3,772	1,988	7,348	63	63.64	33.53	123.96	64
		2007	3,628	1,750	8,239	68	60.70	29.27	137.85	64

Metropolitan Statistical Area	PWID Population	Year	Number	Min	Max	Rank	Rate	Min	Max	Rank
Jacksonville, FL	Non-Hispanic Black	1992	3,247	2,374	4,551	32	269.55	197.09	377.81	55
		1993	2,642	635	4,430	39	214.55	51.56	359.76	59
		1994	3,301	2,776	4,184	29	261.70	220.01	331.68	53
		1995	2,637	810	3,890	36	203.03	62.32	299.49	58
		1996	2,623	888	3,623	33	192.64	65.20	266.05	59
		1997	2,548	940	3,512	33	179.78	66.33	247.86	62
		1998	2,519	974	3,741	32	172.41	66.66	256.02	60
		1999	2,983	2,354	4,002	26	198.53	156.67	266.33	48
		2000	2,471	1,027	4,214	29	160.26	66.64	273.36	57
		2001	2,866	1,963	4,243	26	180.70	123.75	267.51	52
		2002	2,223	929	3,822	32	135.78	56.74	233.50	65
		2003	1,959	870	3,056	35	116.39	51.66	181.53	68
		2004	1,718	802	2,492	42	98.67	46.04	143.10	73
		2005	1,488	736	2,495	51	83.01	41.04	139.16	80
		2006	1,282	675	2,497	59	69.29	36.50	134.97	89
		2007	1,095	528	2,487	63	57.88	27.92	131.45	91
	Hispanic	1992	213	156	299	67	123.87	90.57	173.63	53
		1993	150	36	252	71	83.22	20.00	139.55	60
		1994	172	144	217	71	90.34	75.95	114.50	59
		1995	132	40	194	73	64.95	19.93	95.80	67
		1996	132	45	182	73	58.93	19.94	81.38	69
		1997	134	49	185	73	55.72	20.56	76.82	71
		1998	144	56	214	73	56.80	21.96	84.34	68
		1999	191	151	257	71	71.58	56.48	96.02	61
		2000	183	76	313	67	63.72	26.50	108.68	60
		2001	252	173	373	66	80.66	55.24	119.41	55
		2002	237	99	408	66	69.80	29.17	120.03	56
		2003	259	115	404	66	71.43	31.70	111.42	55
		2004	285	133	414	65	71.90	33.55	104.28	54
		2005	314	155	526	63	73.38	36.28	123.02	51
		2006	345	182	672	64	74.25	39.12	144.63	51
		2007	376	181	855	63	75.86	36.59	172.29	53

Metropolitan Statistical Area	PWID Population	Year	Number	Min	Max	Rank	Rate	Min	Max	Rank
Jacksonville, FL	Male	1992	5,959	4,357	8,352	47	189.81	138.78	266.04	36
		1993	4,474	1,075	7,502	59	142.19	34.17	238.43	45
		1994	5,302	4,457	6,719	53	166.85	140.27	211.47	39
		1995	4,110	1,262	6,063	65	127.19	39.04	187.62	49
		1996	4,046	1,369	5,588	65	120.44	40.76	166.34	49
		1997	3,956	1,460	5,454	64	114.36	42.19	157.66	53
		1998	3,995	1,545	5,933	64	113.41	43.85	168.41	55
		1999	4,892	3,860	6,562	54	137.02	108.12	183.81	43
		2000	4,231	1,760	7,217	60	116.29	48.36	198.36	55
		2001	5,168	3,539	7,650	51	138.76	95.02	205.42	40
		2002	4,244	1,773	7,298	60	111.16	46.45	191.16	56
		2003	3,975	1,764	6,199	64	102.39	45.44	159.71	60
		2004	3,703	1,728	5,371	67	92.71	43.26	134.46	65
		2005	3,399	1,680	5,698	73	83.08	41.07	139.28	68
		2006	3,077	1,621	5,994	81	73.49	38.72	143.15	80
		2007	2,727	1,315	6,193	85	64.19	30.96	145.78	93
	Female	1992	4,468	3,267	6,263	37	140.10	102.44	196.37	17
		1993	3,434	825	5,758	38	105.90	25.45	177.57	25
		1994	4,116	3,460	5,216	38	125.09	105.17	158.54	18
		1995	3,195	981	4,713	42	95.39	29.28	140.70	28
		1996	3,126	1,058	4,317	46	90.43	30.61	124.89	32
		1997	3,021	1,115	4,165	47	85.09	31.40	117.32	39
		1998	3,007	1,162	4,465	46	82.81	32.02	122.97	42
		1999	3,624	2,860	4,862	40	97.84	77.21	131.25	26
		2000	3,091	1,285	5,272	46	81.95	34.08	139.77	38
		2001	3,736	2,558	5,530	42	96.76	66.26	143.23	27
		2002	3,054	1,276	5,252	48	77.13	32.23	132.63	40
		2003	2,872	1,274	4,479	52	70.82	31.43	110.46	46
		2004	2,715	1,267	3,937	56	65.25	30.45	94.64	53
		2005	2,560	1,266	4,292	61	60.16	29.74	100.86	59
		2006	2,418	1,274	4,711	66	55.46	29.22	108.04	64
		2007	2,275	1,097	5,167	68	51.32	24.75	116.54	69

Metropolitan Statistical Area	PWID Population	Year	Number	Min	Max	Rank	Rate	Min	Max	Rank
Jacksonville, FL	Young (15-29)	1992	2,402	1,756	3,366	47	111.78	81.73	156.68	34
		1993	1,634	393	2,740	59	77.99	18.74	130.78	51
		1994	1,808	1,520	2,291	62	87.44	73.51	110.83	46
		1995	1,347	414	1,987	72	65.25	20.03	96.25	56
		1996	1,311	444	1,810	72	61.66	20.87	85.16	60
		1997	1,299	479	1,790	74	59.80	22.06	82.45	65
		1998	1,359	525	2,018	73	61.82	23.90	91.80	65
		1999	1,757	1,386	2,357	66	79.51	62.74	106.66	57
		2000	1,630	678	2,780	72	72.87	30.30	124.30	62
		2001	2,161	1,480	3,199	61	95.61	65.47	141.54	48
		2002	1,943	812	3,341	67	84.49	35.31	145.30	59
		2003	2,002	889	3,123	67	86.16	38.24	134.39	61
		2004	2,057	960	2,984	68	85.89	40.08	124.57	61
		2005	2,082	1,029	3,490	69	85.07	42.05	142.61	60
		2006	2,074	1,093	4,040	71	82.40	43.41	160.51	64
		2007	2,016	972	4,579	69	78.86	38.03	179.09	64
	Old (30-64)	1992	8,020	5,864	11,241	41	191.86	140.28	268.92	25
		1993	6,274	1,508	10,520	45	146.10	35.11	244.98	35
		1994	7,617	6,404	9,654	43	173.11	145.53	219.40	25
		1995	5,972	1,833	8,809	49	132.21	40.58	195.03	38
		1996	5,882	1,991	8,123	49	125.40	42.44	173.18	40
		1997	5,704	2,105	7,864	51	117.90	43.50	162.54	42
		1998	5,672	2,193	8,423	49	114.46	44.25	169.97	43
		1999	6,794	5,361	9,114	44	134.15	105.86	179.96	33
		2000	5,718	2,378	9,753	47	110.52	45.96	188.52	42
		2001	6,764	4,632	10,013	43	127.02	86.99	188.04	35
		2002	5,361	2,240	9,220	49	97.87	40.89	168.30	48
		2003	4,836	2,147	7,544	55	86.16	38.24	134.39	54
		2004	4,338	2,024	6,292	62	75.33	35.15	109.25	59
		2005	3,840	1,898	6,437	66	65.08	32.18	109.11	64
		2006	3,367	1,774	6,559	75	55.83	29.42	108.76	73
		2007	2,913	1,405	6,616	79	47.56	22.94	108.02	82

Metropolitan Statistical Area	PWID Population	Year	Number	Min	Max	Rank	Rate	Min	Max	Rank
Jersey City, NJ	Total	1992	8,986	5,939	13,109	51	231.62	153.10	337.90	13
		1993	7,251	3,182	11,652	58	185.64	81.47	298.31	17
		1994	8,192	6,987	10,201	54	208.19	177.57	259.24	14
		1995	6,588	3,301	8,944	63	165.32	82.85	224.44	19
		1996	6,070	3,402	7,858	67	150.09	84.11	194.31	25
		1997	5,596	3,521	6,925	70	135.94	85.53	168.22	29
		1998	5,245	3,623	6,070	72	125.84	86.94	145.64	38
		1999	5,608	5,307	5,939	72	133.24	126.08	141.09	30
		2000	5,299	3,864	7,203	74	124.70	90.93	169.52	33
		2001	5,929	4,051	8,315	72	138.31	94.50	193.98	24
		2002	5,336	3,487	8,496	75	125.20	81.81	199.32	31
		2003	4,967	3,002	7,471	79	117.20	70.83	176.29	31
		2004	4,559	2,589	5,872	84	107.99	61.32	139.09	39
		2005	4,498	2,237	6,830	85	106.72	53.09	162.06	38
		2006	4,547	1,927	8,188	84	108.13	45.82	194.69	34
		2007	4,632	1,620	9,644	82	110.52	38.65	230.09	32
	Non-Hispanic White	1992	4,210	2,782	6,141	54	248.49	164.25	362.51	3
		1993	3,226	1,416	5,184	64	194.18	85.22	312.03	6
		1994	3,508	2,992	4,368	65	214.49	182.94	267.09	3
		1995	2,749	1,377	3,731	71	169.89	85.14	230.64	7
		1996	2,494	1,398	3,229	75	155.70	87.26	201.56	12
		1997	2,285	1,438	2,828	79	143.44	90.25	177.50	12
		1998	2,143	1,480	2,480	80	135.74	93.78	157.10	14
		1999	2,304	2,181	2,440	81	147.44	139.52	156.13	8
		2000	2,196	1,601	2,985	81	142.20	103.69	193.31	11
		2001	2,481	1,695	3,479	80	159.23	108.80	223.33	8
		2002	2,252	1,472	3,585	82	147.00	96.06	234.04	8
		2003	2,108	1,274	3,171	85	139.40	84.24	209.67	12
		2004	1,938	1,100	2,496	88	129.14	73.33	166.33	14
		2005	1,902	946	2,889	89	127.65	63.50	193.84	13
		2006	1,899	805	3,419	89	128.32	54.37	231.04	15
		2007	1,892	662	3,939	91	128.43	44.92	267.37	17

Metropolitan Statistical Area	PWID Population	Year	Number	Min	Max	Rank	Rate	Min	Max	Rank
Jersey City, NJ	Non-Hispanic Black	1992	2,648	1,750	3,863	43	542.48	358.57	791.39	15
		1993	2,089	917	3,357	51	425.64	186.79	683.98	16
		1994	2,304	1,965	2,869	45	464.93	396.55	578.94	15
		1995	1,808	906	2,455	52	360.69	180.75	489.66	22
		1996	1,627	912	2,106	55	319.89	179.27	414.11	25
		1997	1,466	923	1,814	57	283.84	178.58	351.25	28
		1998	1,347	930	1,559	58	257.93	178.20	298.50	29
		1999	1,416	1,340	1,500	59	269.49	255.01	285.35	21
		2000	1,322	964	1,797	59	250.29	182.52	340.25	24
		2001	1,470	1,004	2,062	57	278.51	190.29	390.61	18
		2002	1,323	865	2,107	59	252.21	164.81	401.53	20
		2003	1,242	750	1,867	61	238.49	144.13	358.72	20
		2004	1,159	658	1,493	62	222.50	126.34	286.57	22
		2005	1,174	584	1,782	63	224.83	111.84	341.41	22
		2006	1,231	522	2,217	61	236.19	100.08	425.28	18
		2007	1,316	460	2,740	58	253.73	88.74	528.23	18
	Hispanic	1992	2,268	1,499	3,309	29	163.85	108.30	239.03	39
		1993	1,874	822	3,011	33	131.84	57.86	211.86	42
		1994	2,181	1,860	2,716	33	149.92	127.87	186.68	36
		1995	1,814	909	2,462	34	121.12	60.70	164.43	42
		1996	1,733	971	2,244	34	112.22	62.89	145.27	43
		1997	1,660	1,045	2,054	34	104.05	65.46	128.76	43
		1998	1,617	1,117	1,871	37	98.78	68.24	114.32	43
		1999	1,794	1,698	1,900	37	107.63	101.85	113.97	39
		2000	1,757	1,281	2,388	36	103.21	75.26	140.30	40
		2001	2,031	1,388	2,849	35	118.58	81.02	166.31	35
		2002	1,883	1,230	2,998	35	109.93	71.84	175.02	36
		2003	1,797	1,086	2,704	36	105.13	63.53	158.13	38
		2004	1,684	956	2,169	37	98.96	56.19	127.46	40
		2005	1,687	839	2,562	37	99.51	49.50	151.11	39
		2006	1,722	729	3,100	37	102.02	43.23	183.70	38
		2007	1,759	615	3,661	38	104.84	36.67	218.26	34

Metropolitan Statistical Area	PWID Population	Year	Number	Min	Max	Rank	Rate	Min	Max	Rank
Jersey City, NJ	Male	1992	6,127	4,050	8,938	45	316.50	209.20	461.72	8
		1993	4,887	2,145	7,853	49	250.55	109.95	402.62	17
		1994	5,512	4,701	6,864	49	280.16	238.95	348.86	11
		1995	4,461	2,236	6,056	59	223.56	112.03	303.50	18
		1996	4,162	2,332	5,388	64	205.45	115.13	265.96	24
		1997	3,900	2,454	4,827	66	189.01	118.92	233.89	27
		1998	3,721	2,571	4,307	66	178.03	123.00	206.04	29
		1999	4,051	3,833	4,289	66	191.53	181.24	202.81	23
		2000	3,888	2,835	5,286	65	181.99	132.71	247.40	25
		2001	4,406	3,011	6,180	62	204.21	139.53	286.40	17
		2002	3,999	2,613	6,367	64	186.14	121.64	296.35	20
		2003	3,733	2,256	5,615	68	174.53	105.48	262.52	22
		2004	3,413	1,938	4,396	74	160.03	90.87	206.11	27
		2005	3,325	1,654	5,050	77	155.95	77.58	236.81	28
		2006	3,284	1,391	5,912	75	154.08	65.29	277.43	28
		2007	3,218	1,125	6,700	76	151.34	52.93	315.06	29
	Female	1992	2,875	1,900	4,194	53	147.93	97.78	215.80	12
		1993	2,392	1,050	3,843	60	122.31	53.68	196.55	20
		1994	2,727	2,326	3,395	58	138.57	118.19	172.55	13
		1995	2,174	1,090	2,952	66	109.30	54.78	148.39	21
		1996	1,960	1,098	2,538	72	97.13	54.43	125.74	27
		1997	1,751	1,102	2,167	77	85.28	53.65	105.53	37
		1998	1,580	1,092	1,828	82	76.05	52.54	88.02	47
		1999	1,623	1,536	1,719	83	77.50	73.34	82.07	47
		2000	1,476	1,076	2,006	83	69.86	50.94	94.97	52
		2001	1,599	1,093	2,243	84	75.13	51.33	105.36	49
		2002	1,409	921	2,244	85	66.67	43.57	106.14	54
		2003	1,304	788	1,962	87	62.13	37.55	93.45	60
		2004	1,214	689	1,564	89	58.12	33.00	74.85	60
		2005	1,244	619	1,889	89	59.74	29.72	90.72	60
		2006	1,342	569	2,417	88	64.70	27.42	116.50	53
		2007	1,502	525	3,127	86	72.75	25.44	151.45	40

Metropolitan Statistical Area	PWID Population	Year	Number	Min	Max	Rank	Rate	Min	Max	Rank
Jersey City, NJ	Young (15-29)	1992	2,427	1,604	3,541	46	172.41	113.96	251.51	12
		1993	2,081	913	3,343	48	148.87	65.33	239.22	15
		1994	2,446	2,086	3,045	42	176.06	150.17	219.24	11
		1995	2,010	1,007	2,729	49	144.48	72.40	196.14	15
		1996	1,864	1,045	2,414	56	133.06	74.57	172.25	19
		1997	1,709	1,075	2,115	63	120.22	75.64	148.77	28
		1998	1,575	1,088	1,823	68	109.40	75.58	126.61	36
		1999	1,642	1,554	1,738	71	112.92	106.85	119.56	35
		2000	1,502	1,095	2,042	77	102.63	74.84	139.52	44
		2001	1,620	1,107	2,273	79	113.05	77.24	158.56	37
		2002	1,403	917	2,233	83	101.55	66.36	161.68	46
		2003	1,256	759	1,889	87	94.09	56.86	141.52	52
		2004	1,112	632	1,432	91	86.05	48.86	110.84	60
		2005	1,064	529	1,616	92	84.48	42.03	128.29	62
		2006	1,052	446	1,895	92	85.62	36.28	154.16	59
		2007	1,060	371	2,207	95	88.53	30.96	184.29	53
	Old (30-64)	1992	6,640	4,389	9,687	49	268.65	177.57	391.91	7
		1993	5,230	2,295	8,405	60	208.52	91.51	335.08	19
		1994	5,813	4,958	7,239	57	228.32	194.74	284.31	13
		1995	4,634	2,322	6,291	64	178.66	89.53	242.55	23
		1996	4,262	2,388	5,517	68	161.27	90.38	208.77	25
		1997	3,946	2,483	4,883	73	146.38	92.10	181.15	31
		1998	3,730	2,577	4,317	75	136.73	94.47	158.24	33
		1999	4,037	3,820	4,275	73	146.53	138.66	155.16	28
		2000	3,868	2,820	5,258	72	138.85	101.25	188.75	25
		2001	4,392	3,001	6,159	71	153.92	105.17	215.87	22
		2002	4,011	2,621	6,386	67	139.22	90.98	221.65	25
		2003	3,784	2,287	5,692	73	130.37	78.79	196.09	26
		2004	3,516	1,997	4,529	76	120.03	68.15	154.59	28
		2005	3,503	1,742	5,319	75	118.54	58.97	180.01	28
		2006	3,565	1,511	6,419	72	119.77	50.75	215.66	24
		2007	3,641	1,273	7,579	69	121.60	42.53	253.16	18

Metropolitan Statistical Area	PWID Population	Year	Number	Min	Max	Rank	Rate	Min	Max	Rank
Kansas City, MO--KS	Total	1992	8,461	4,627	11,516	54	79.50	43.48	108.22	71
		1993	7,503	4,884	11,282	54	69.64	45.33	104.72	78
		1994	8,262	5,543	11,056	53	75.66	50.76	101.24	77
		1995	7,276	4,701	10,816	55	65.83	42.53	97.86	79
		1996	7,169	4,614	10,611	58	63.90	41.13	94.59	79
		1997	7,058	4,517	10,410	58	61.99	39.68	91.43	79
		1998	6,749	4,406	10,187	63	58.55	38.22	88.37	82
		1999	7,153	5,516	9,976	59	61.25	47.23	85.42	79
		2000	6,268	4,178	9,783	66	52.89	35.26	82.56	86
		2001	6,769	4,278	9,590	65	56.33	35.60	79.80	88
		2002	6,219	3,891	9,406	65	51.00	31.91	77.14	91
		2003	6,328	3,723	9,183	64	51.38	30.23	74.56	91
		2004	6,527	3,551	8,975	59	52.41	28.52	72.07	92
		2005	6,734	3,370	9,553	57	53.45	26.74	75.82	94
		2006	6,967	3,184	10,473	57	54.64	24.97	82.13	90
		2007	7,203	2,988	11,413	57	55.82	23.16	88.44	86
	Non-Hispanic White	1992	6,028	3,297	8,205	38	68.31	37.36	92.98	55
		1993	5,387	3,506	8,100	40	60.55	39.41	91.05	59
		1994	5,956	3,996	7,971	38	66.35	44.52	88.79	56
		1995	5,248	3,391	7,802	38	58.04	37.49	86.28	60
		1996	5,155	3,318	7,630	37	56.46	36.34	83.58	61
		1997	5,041	3,227	7,435	37	54.70	35.01	80.67	64
		1998	4,768	3,113	7,198	40	51.38	33.54	77.55	68
		1999	4,978	3,839	6,943	41	53.22	41.04	74.23	71
		2000	4,274	2,850	6,672	47	45.31	30.21	70.73	76
		2001	4,498	2,843	6,373	47	47.18	29.82	66.85	76
		2002	4,001	2,503	6,052	51	41.48	25.95	62.73	83
		2003	3,912	2,302	5,676	55	40.28	23.70	58.46	86
		2004	3,843	2,091	5,284	59	39.26	21.36	53.98	88
		2005	3,739	1,871	5,304	62	37.86	18.95	53.72	93
		2006	3,608	1,649	5,424	67	36.23	16.56	54.47	96
		2007	3,437	1,426	5,445	72	34.19	14.18	54.17	95

Metropolitan Statistical Area	PWID Population	Year	Number	Min	Max	Rank	Rate	Min	Max	Rank
Kansas City, MO--KS	Non-Hispanic Black	1992	1,882	1,029	2,561	58	145.65	79.65	198.25	89
		1993	1,597	1,039	2,401	59	121.47	79.07	182.65	92
		1994	1,694	1,137	2,268	59	126.34	84.76	169.07	92
		1995	1,450	937	2,155	59	106.15	68.58	157.81	93
		1996	1,402	902	2,075	61	100.56	64.72	148.85	93
		1997	1,370	877	2,021	62	96.39	61.69	142.16	91
		1998	1,317	860	1,989	62	91.32	59.61	137.84	93
		1999	1,425	1,099	1,988	58	97.43	75.13	135.88	87
		2000	1,295	863	2,021	60	87.08	58.06	135.93	89
		2001	1,474	931	2,088	56	97.95	61.91	138.77	85
		2002	1,452	909	2,196	55	95.61	59.82	144.60	82
		2003	1,612	948	2,338	48	105.17	61.88	152.60	72
		2004	1,842	1,002	2,533	36	118.84	64.66	163.41	66
		2005	2,137	1,069	3,031	34	136.57	68.34	193.75	58
		2006	2,512	1,148	3,776	28	158.26	72.33	237.91	47
		2007	2,969	1,232	4,704	26	185.36	76.90	293.70	42
	Hispanic	1992	237	129	322	64	71.05	38.85	96.70	69
		1993	207	135	311	66	57.89	37.68	87.05	76
		1994	223	150	299	65	58.22	39.06	77.91	77
		1995	193	125	286	66	46.71	30.18	69.44	80
		1996	186	120	275	67	41.48	26.70	61.40	81
		1997	179	115	265	67	36.83	23.57	54.31	84
		1998	169	110	254	68	31.96	20.87	48.25	84
		1999	176	136	246	72	30.95	23.86	43.16	85
		2000	154	102	240	72	24.94	16.62	38.93	89
		2001	166	105	235	72	25.50	16.12	36.13	89
		2002	155	97	234	74	22.50	14.08	34.03	88
		2003	161	95	234	74	22.38	13.17	32.47	89
		2004	173	94	238	74	23.02	12.53	31.66	89
		2005	190	95	269	73	24.00	12.01	34.05	86
		2006	212	97	319	72	25.64	11.72	38.54	85
		2007	244	101	386	69	28.16	11.68	44.62	82

Metropolitan Statistical Area	PWID Population	Year	Number	Min	Max	Rank	Rate	Min	Max	Rank
Kansas City, MO--KS	Male	1992	5,750	3,144	7,826	51	109.94	60.12	149.64	67
		1993	4,913	3,198	7,387	48	92.73	60.36	139.44	75
		1994	5,256	3,526	7,034	55	97.76	65.59	130.82	81
		1995	4,534	2,929	6,740	56	83.28	53.80	123.80	80
		1996	4,409	2,838	6,526	60	79.77	51.34	118.08	82
		1997	4,311	2,759	6,358	62	76.79	49.15	113.26	81
		1998	4,113	2,685	6,208	63	72.31	47.21	109.15	84
		1999	4,364	3,365	6,086	62	75.61	58.30	105.45	83
		2000	3,832	2,555	5,981	67	65.38	43.59	102.05	87
		2001	4,146	2,620	5,874	66	69.71	44.06	98.77	85
		2002	3,808	2,383	5,760	67	63.09	39.47	95.42	89
		2003	3,859	2,271	5,599	66	63.29	37.24	91.84	91
		2004	3,941	2,144	5,419	62	63.89	34.76	87.84	93
		2005	3,994	1,999	5,667	63	63.99	32.02	90.78	93
		2006	4,019	1,837	6,041	64	63.49	29.02	95.44	94
		2007	3,986	1,654	6,316	67	62.23	25.81	98.59	94
	Female	1992	2,801	1,532	3,812	55	51.75	28.30	70.44	72
		1993	2,673	1,740	4,019	52	48.82	31.78	73.42	73
		1994	3,103	2,082	4,152	46	55.97	37.55	74.90	69
		1995	2,833	1,830	4,211	49	50.51	32.63	75.09	74
		1996	2,855	1,838	4,227	49	50.17	32.29	74.27	72
		1997	2,847	1,822	4,199	49	49.33	31.57	72.75	73
		1998	2,736	1,786	4,130	52	46.85	30.59	70.72	75
		1999	2,900	2,236	4,044	55	49.08	37.85	68.45	79
		2000	2,534	1,689	3,955	58	42.31	28.21	66.04	83
		2001	2,728	1,724	3,864	59	44.94	28.40	63.67	86
		2002	2,503	1,566	3,786	61	40.65	25.44	61.49	86
		2003	2,556	1,504	3,709	61	41.10	24.18	59.64	86
		2004	2,664	1,449	3,663	59	42.38	23.06	58.28	84
		2005	2,802	1,402	3,975	54	44.08	22.06	62.53	82
		2006	2,989	1,366	4,493	51	46.54	21.27	69.96	77
		2007	3,226	1,338	5,112	47	49.64	20.59	78.65	72

Metropolitan Statistical Area	PWID Population	Year	Number	Min	Max	Rank	Rate	Min	Max	Rank
Kansas City, MO--KS	Young (15-29)	1992	2,212	1,210	3,011	51	65.00	35.55	88.47	68
		1993	2,045	1,331	3,075	49	60.45	39.35	90.90	63
		1994	2,316	1,554	3,099	43	68.49	45.95	91.66	61
		1995	2,074	1,340	3,084	48	61.10	39.48	90.84	59
		1996	2,062	1,327	3,052	49	60.02	38.63	88.85	62
		1997	2,037	1,304	3,004	54	58.52	37.46	86.32	66
		1998	1,949	1,272	2,942	57	55.30	36.10	83.47	71
		1999	2,067	1,594	2,883	58	58.09	44.80	81.02	75
		2000	1,817	1,211	2,836	63	50.73	33.82	79.18	81
		2001	1,978	1,250	2,802	66	55.26	34.92	78.29	81
		2002	1,846	1,155	2,793	69	51.18	32.02	77.41	84
		2003	1,928	1,135	2,798	68	53.24	31.33	77.26	87
		2004	2,067	1,124	2,842	67	56.53	30.76	77.73	87
		2005	2,247	1,124	3,187	66	60.93	30.49	86.43	83
		2006	2,487	1,137	3,739	60	66.50	30.40	99.97	81
		2007	2,794	1,159	4,426	54	74.05	30.72	117.32	69
	Old (30-64)	1992	6,345	3,470	8,636	54	87.65	47.93	119.31	73
		1993	5,541	3,607	8,332	55	74.98	48.81	112.75	81
		1994	6,043	4,054	8,086	54	80.15	53.77	107.25	82
		1995	5,295	3,421	7,872	57	69.14	44.67	102.79	81
		1996	5,210	3,353	7,712	58	66.94	43.09	99.09	82
		1997	5,135	3,286	7,573	59	64.95	41.57	95.80	80
		1998	4,918	3,211	7,423	60	61.46	40.12	92.77	82
		1999	5,219	4,025	7,279	57	64.26	49.56	89.63	84
		2000	4,570	3,046	7,133	62	55.27	36.85	86.27	87
		2001	4,917	3,107	6,966	60	58.27	36.83	82.56	83
		2002	4,481	2,804	6,777	62	52.19	32.65	78.93	87
		2003	4,497	2,646	6,525	60	51.72	30.43	75.05	85
		2004	4,538	2,469	6,240	58	51.58	28.07	70.93	83
		2005	4,533	2,268	6,431	57	50.87	25.46	72.17	83
		2006	4,476	2,046	6,729	56	49.67	22.70	74.67	82
		2007	4,329	1,796	6,859	55	47.41	19.67	75.11	83

Metropolitan Statistical Area	PWID Population	Year	Number	Min	Max	Rank	Rate	Min	Max	Rank
Knoxville, TN	Total	1992	3,825	2,462	5,485	85	92.38	59.45	132.47	60
		1993	3,417	1,657	5,257	84	81.06	39.31	124.69	64
		1994	4,203	3,709	5,020	83	97.60	86.12	116.58	52
		1995	3,751	1,782	4,771	83	85.29	40.53	108.47	59
		1996	3,950	1,869	5,168	83	88.16	41.71	115.35	57
		1997	4,103	1,963	5,828	84	90.51	43.30	128.56	55
		1998	4,145	2,054	6,052	84	90.57	44.88	132.23	55
		1999	4,695	3,552	5,760	84	101.63	76.90	124.70	51
		2000	3,934	2,257	5,195	85	84.21	48.31	111.21	59
		2001	4,296	3,096	5,166	84	90.74	65.40	109.12	57
		2002	3,938	2,903	5,378	86	82.10	60.53	112.14	61
		2003	4,127	3,137	5,599	86	84.96	64.59	115.26	57
		2004	4,491	3,219	5,829	85	91.26	65.42	118.46	52
		2005	4,901	3,313	6,197	80	98.02	66.26	123.93	45
		2006	5,347	3,426	7,163	78	105.04	67.30	140.71	39
		2007	5,801	3,533	8,166	74	112.21	68.34	157.96	30
	Non-Hispanic White	1992	2,650	1,706	3,800	73	69.17	44.51	99.18	54
		1993	2,387	1,157	3,672	73	61.27	29.71	94.25	57
		1994	2,977	2,627	3,557	71	74.91	66.10	89.48	51
		1995	2,707	1,286	3,443	73	66.76	31.72	84.91	54
		1996	2,912	1,378	3,810	71	70.60	33.40	92.37	54
		1997	3,094	1,480	4,395	67	74.22	35.50	105.43	54
		1998	3,199	1,585	4,671	65	76.11	37.72	111.12	52
		1999	3,705	2,804	4,547	60	87.46	66.17	107.31	41
		2000	3,170	1,819	4,187	65	74.21	42.57	98.00	54
		2001	3,528	2,543	4,242	63	81.61	58.81	98.13	49
		2002	3,287	2,424	4,490	66	75.20	55.44	102.71	52
		2003	3,493	2,655	4,738	64	79.04	60.09	107.23	46
		2004	3,842	2,754	4,987	60	86.03	61.67	111.67	42
		2005	4,227	2,857	5,344	55	93.32	63.08	117.99	37
		2006	4,634	2,969	6,207	48	100.75	64.56	134.97	35
		2007	5,033	3,065	7,086	45	108.08	65.82	152.14	27

Metropolitan Statistical Area	PWID Population	Year	Number	Min	Max	Rank	Rate	Min	Max	Rank
Knoxville, TN	Non-Hispanic Black	1992	1,031	663	1,478	78	435.93	280.56	625.14	26
		1993	874	424	1,344	81	361.68	175.38	556.37	34
		1994	1,002	884	1,197	74	407.61	359.67	486.90	21
		1995	822	391	1,046	79	327.79	155.76	416.92	29
		1996	785	372	1,027	77	307.21	145.34	401.95	27
		1997	732	350	1,039	78	283.24	135.49	402.33	29
		1998	657	326	960	80	252.07	124.92	368.03	34
		1999	658	498	808	81	250.54	189.56	307.41	27
		2000	487	279	643	87	181.24	103.97	239.36	49
		2001	470	339	565	87	171.94	123.92	206.75	55
		2002	383	282	523	89	137.81	101.61	188.24	63
		2003	360	274	489	89	126.83	96.42	172.06	65
		2004	357	256	464	89	122.86	88.07	159.47	62
		2005	362	245	458	89	121.26	81.97	153.32	63
		2006	376	241	503	91	121.29	77.72	162.48	63
		2007	400	243	563	89	125.84	76.64	177.16	60
	Hispanic	1992	17	11	24	97	67.91	43.71	97.39	72
		1993	16	8	25	98	59.29	28.75	91.20	73
		1994	20	18	24	97	67.28	59.37	80.37	73
		1995	18	9	23	97	54.23	25.77	68.97	74
		1996	19	9	25	96	49.94	23.63	65.34	75
		1997	19	9	27	97	45.57	21.80	64.73	75
		1998	18	9	27	98	39.29	19.47	57.37	78
		1999	20	15	24	98	38.11	28.83	46.75	79
		2000	16	9	21	99	26.31	15.09	34.75	87
		2001	16	12	19	98	25.52	18.39	30.69	88
		2002	14	10	19	98	20.69	15.25	28.26	91
		2003	15	11	20	98	20.04	15.23	27.18	91
		2004	16	11	21	98	20.31	14.56	26.37	92
		2005	18	12	23	97	21.27	14.38	26.90	92
		2006	21	13	28	97	22.90	14.67	30.68	88
		2007	25	15	35	97	25.26	15.38	35.56	87

Metropolitan Statistical Area	PWID Population	Year	Number	Min	Max	Rank	Rate	Min	Max	Rank
Knoxville, TN	Male	1992	2,509	1,615	3,597	85	123.85	79.71	177.60	58
		1993	2,177	1,055	3,348	85	105.45	51.13	162.21	66
		1994	2,614	2,307	3,123	83	123.91	109.33	148.01	54
		1995	2,291	1,089	2,914	84	106.22	50.47	135.10	63
		1996	2,382	1,127	3,116	84	108.33	51.25	141.74	61
		1997	2,452	1,173	3,483	84	110.31	52.77	156.69	58
		1998	2,465	1,222	3,599	84	109.74	54.39	160.23	57
		1999	2,786	2,108	3,418	84	122.73	92.85	150.58	52
		2000	2,333	1,339	3,082	86	101.57	58.26	134.14	62
		2001	2,549	1,837	3,066	85	109.50	78.92	131.67	59
		2002	2,337	1,723	3,193	86	99.19	73.13	135.49	62
		2003	2,448	1,861	3,321	86	102.68	78.06	139.30	59
		2004	2,656	1,904	3,448	86	109.77	78.69	142.48	56
		2005	2,882	1,948	3,644	85	117.07	79.13	148.01	48
		2006	3,112	1,994	4,169	79	124.23	79.60	166.42	44
		2007	3,324	2,024	4,680	75	130.42	79.43	183.60	36
	Female	1992	1,383	890	1,983	81	65.37	42.07	93.75	57
		1993	1,290	625	1,984	82	59.94	29.06	92.20	57
		1994	1,643	1,450	1,963	79	74.81	66.02	89.37	47
		1995	1,509	717	1,919	80	67.33	31.99	85.64	54
		1996	1,625	769	2,126	79	71.22	33.69	93.19	51
		1997	1,717	822	2,440	79	74.35	35.57	105.61	48
		1998	1,757	871	2,565	79	75.40	37.37	110.09	48
		1999	2,007	1,519	2,463	73	85.44	64.65	104.84	37
		2000	1,690	970	2,232	80	71.20	40.84	94.03	48
		2001	1,849	1,333	2,224	80	76.86	55.39	92.42	47
		2002	1,693	1,249	2,313	81	69.42	51.18	94.82	52
		2003	1,770	1,345	2,401	80	71.54	54.38	97.05	45
		2004	1,916	1,374	2,487	77	76.63	54.93	99.46	39
		2005	2,079	1,405	2,628	71	81.89	55.36	103.54	31
		2006	2,252	1,443	3,017	68	87.09	55.80	116.67	24
		2007	2,425	1,477	3,413	63	92.52	56.35	130.24	22

Metropolitan Statistical Area	PWID Population	Year	Number	Min	Max	Rank	Rate	Min	Max	Rank
Knoxville, TN	Young (15-29)	1992	1,302	838	1,867	77	94.67	60.93	135.76	45
		1993	1,048	508	1,611	78	76.08	36.89	117.03	52
		1994	1,196	1,056	1,429	78	86.45	76.28	103.27	48
		1995	1,022	486	1,300	80	73.13	34.75	93.01	55
		1996	1,060	502	1,387	80	75.06	35.51	98.22	55
		1997	1,113	532	1,581	81	79.02	37.80	112.25	53
		1998	1,162	576	1,697	81	82.24	40.75	120.07	55
		1999	1,385	1,048	1,699	79	98.09	74.21	120.36	42
		2000	1,238	710	1,635	83	87.68	50.29	115.79	53
		2001	1,456	1,049	1,750	82	102.46	73.85	123.21	44
		2002	1,443	1,064	1,971	81	100.85	74.35	137.75	48
		2003	1,638	1,245	2,222	77	113.04	85.93	153.36	41
		2004	1,924	1,379	2,497	73	130.35	93.45	169.19	27
		2005	2,254	1,524	2,850	65	149.86	101.30	189.47	24
		2006	2,618	1,677	3,507	57	170.52	109.26	228.42	17
		2007	2,993	1,823	4,213	49	191.02	116.33	268.90	14
	Old (30-64)	1992	2,561	1,648	3,672	86	92.59	59.59	132.78	68
		1993	2,385	1,157	3,669	86	84.02	40.74	129.25	69
		1994	3,020	2,665	3,608	84	103.34	91.19	123.44	58
		1995	2,747	1,305	3,494	84	91.54	43.50	116.43	63
		1996	2,923	1,383	3,824	82	95.25	45.06	124.63	59
		1997	3,043	1,455	4,322	82	97.38	46.58	138.32	56
		1998	3,057	1,515	4,464	81	96.64	47.90	141.11	57
		1999	3,417	2,585	4,193	79	106.55	80.61	130.73	49
		2000	2,801	1,607	3,700	83	85.95	49.30	113.51	58
		2001	2,965	2,137	3,565	81	89.47	64.48	107.59	55
		2002	2,605	1,920	3,558	86	77.41	57.07	105.73	58
		2003	2,583	1,964	3,504	86	75.78	57.61	102.81	58
		2004	2,619	1,878	3,400	85	76.04	54.51	98.70	56
		2005	2,616	1,768	3,307	84	74.83	50.58	94.60	56
		2006	2,556	1,638	3,424	85	71.88	46.06	96.30	56
		2007	2,425	1,477	3,413	86	67.30	40.99	94.74	59

Metropolitan Statistical Area	PWID Population	Year	Number	Min	Max	Rank	Rate	Min	Max	Rank
Las Vegas, NV--AZ	Total	1992	13,979	9,732	17,927	34	212.09	147.65	271.98	14
		1993	11,643	4,195	17,512	35	168.91	60.86	254.04	22
		1994	14,690	12,296	17,547	32	197.78	165.55	236.24	15
		1995	12,837	5,991	17,374	32	162.16	75.68	219.47	21
		1996	13,339	6,915	17,090	30	159.15	82.50	203.89	19
		1997	14,024	7,944	17,053	29	156.20	88.48	189.93	18
		1998	14,377	8,904	17,715	29	151.44	93.79	186.59	20
		1999	16,294	14,190	18,336	25	162.85	141.82	183.26	16
		2000	14,502	9,076	17,884	29	137.48	86.05	169.56	24
		2001	16,104	15,491	17,112	24	146.68	141.10	155.86	21
		2002	13,949	7,746	17,022	29	122.49	68.02	149.47	32
		2003	13,659	6,944	18,373	30	115.87	58.91	155.86	33
		2004	13,717	6,746	19,984	30	111.20	54.69	162.00	36
		2005	13,709	6,579	21,564	29	106.89	51.30	168.13	36
		2006	13,759	6,458	23,339	30	103.06	48.38	174.82	41
		2007	13,667	6,320	24,963	31	99.38	45.96	181.51	40
	Non-Hispanic White	1992	10,887	7,579	13,961	19	218.39	152.04	280.06	6
		1993	8,800	3,171	13,235	23	170.95	61.60	257.12	11
		1994	10,802	9,041	12,902	17	197.97	165.71	236.47	8
		1995	9,211	4,299	12,467	23	161.41	75.33	218.45	9
		1996	9,371	4,858	12,006	22	157.90	81.86	202.29	9
		1997	9,677	5,481	11,767	19	155.78	88.24	189.42	7
		1998	9,775	6,054	12,045	19	152.34	94.35	187.71	7
		1999	10,947	9,533	12,318	17	165.72	144.32	186.48	7
		2000	9,650	6,040	11,901	21	141.41	88.50	174.40	12
		2001	10,636	10,231	11,302	18	152.14	146.35	161.66	11
		2002	9,158	5,085	11,175	21	128.18	71.17	156.41	15
		2003	8,923	4,536	12,003	22	122.12	62.08	164.27	16
		2004	8,920	4,387	12,995	21	118.30	58.18	172.34	19
		2005	8,874	4,259	13,958	21	115.42	55.39	181.56	23
		2006	8,858	4,158	15,026	23	113.07	53.07	191.80	23
		2007	8,741	4,042	15,966	23	110.59	51.14	201.99	25

Metropolitan Statistical Area	PWID Population	Year	Number	Min	Max	Rank	Rate	Min	Max	Rank
Las Vegas, NV--AZ	Non-Hispanic Black	1992	1,890	1,316	2,424	56	358.84	249.81	460.17	37
		1993	1,547	557	2,326	60	283.28	102.07	426.07	49
		1994	1,912	1,600	2,284	55	321.62	269.21	384.15	38
		1995	1,634	763	2,212	57	257.83	120.33	348.96	47
		1996	1,660	860	2,126	54	247.43	128.28	317.00	45
		1997	1,706	966	2,075	53	235.54	133.42	286.42	45
		1998	1,713	1,061	2,111	51	222.05	137.52	273.60	44
		1999	1,908	1,661	2,147	46	233.90	203.70	263.21	36
		2000	1,675	1,048	2,066	51	195.46	122.33	241.05	42
		2001	1,845	1,775	1,960	45	205.14	197.33	217.98	39
		2002	1,595	886	1,946	49	169.30	94.00	206.59	47
		2003	1,571	799	2,113	51	159.92	81.30	215.12	51
		2004	1,601	787	2,333	47	153.45	75.47	223.55	51
		2005	1,642	788	2,582	45	148.27	71.16	233.23	52
		2006	1,712	804	2,904	44	145.60	68.34	246.98	54
		2007	1,794	829	3,276	44	145.66	67.36	266.04	53
	Hispanic	1992	873	608	1,119	40	113.26	78.85	145.24	58
		1993	698	251	1,049	48	81.37	29.32	122.38	61
		1994	867	726	1,035	41	87.56	73.29	104.58	62
		1995	763	356	1,032	47	66.80	31.17	90.40	64
		1996	813	421	1,041	45	62.79	32.55	80.44	66
		1997	889	504	1,081	44	59.18	33.52	71.96	66
		1998	958	593	1,181	43	56.15	34.77	69.18	69
		1999	1,150	1,001	1,294	42	60.07	52.31	67.60	65
		2000	1,087	680	1,341	44	51.23	32.07	63.19	66
		2001	1,282	1,233	1,362	42	55.99	53.86	59.50	64
		2002	1,176	653	1,435	44	48.10	26.71	58.70	65
		2003	1,212	616	1,630	44	46.75	23.77	62.89	66
		2004	1,270	624	1,849	43	45.68	22.47	66.55	66
		2005	1,308	628	2,058	43	43.77	21.00	68.84	68
		2006	1,335	626	2,264	43	41.43	19.45	70.27	67
		2007	1,326	613	2,422	43	38.62	17.86	70.53	73

Metropolitan Statistical Area	PWID Population	Year	Number	Min	Max	Rank	Rate	Min	Max	Rank
Las Vegas, NV--AZ	Male	1992	9,044	6,296	11,598	35	268.09	186.64	343.79	16
		1993	7,631	2,750	11,478	38	216.64	78.06	325.83	26
		1994	9,722	8,137	11,612	33	255.91	214.21	305.67	16
		1995	8,552	3,991	11,575	31	211.27	98.60	285.94	25
		1996	8,923	4,626	11,432	31	208.33	108.00	266.90	22
		1997	9,398	5,323	11,427	28	204.59	115.89	248.78	22
		1998	9,632	5,965	11,868	28	198.25	122.78	244.28	22
		1999	10,893	9,486	12,257	22	212.61	185.15	239.24	17
		2000	9,658	6,045	11,911	27	178.74	111.87	220.44	27
		2001	10,670	10,264	11,337	23	189.98	182.75	201.87	22
		2002	9,184	5,099	11,207	26	157.95	87.70	192.74	34
		2003	8,927	4,538	12,007	28	148.43	75.46	199.66	36
		2004	8,892	4,373	12,954	29	141.24	69.46	205.76	38
		2005	8,809	4,228	13,856	29	134.37	64.49	211.36	39
		2006	8,761	4,112	14,861	29	128.28	60.21	217.60	41
		2007	8,622	3,987	15,749	29	122.50	56.65	223.74	42
	Female	1992	5,042	3,510	6,466	31	156.69	109.08	200.93	8
		1993	4,098	1,476	6,163	35	121.58	43.81	182.86	21
		1994	5,072	4,246	6,059	31	139.79	117.01	166.97	12
		1995	4,371	2,040	5,916	32	112.99	52.73	152.93	20
		1996	4,501	2,333	5,766	32	109.81	56.93	140.68	19
		1997	4,709	2,668	5,726	31	107.39	60.83	130.59	19
		1998	4,824	2,988	5,945	29	104.07	64.45	128.23	18
		1999	5,483	4,775	6,170	26	112.31	97.81	126.38	17
		2000	4,909	3,072	6,054	29	95.42	59.72	117.68	23
		2001	5,498	5,289	5,842	28	102.53	98.63	108.95	24
		2002	4,814	2,673	5,875	32	86.38	47.96	105.40	33
		2003	4,773	2,427	6,420	32	82.66	42.03	111.19	33
		2004	4,860	2,390	7,080	31	80.45	39.57	117.21	34
		2005	4,928	2,365	7,752	31	78.60	37.72	123.63	36
		2006	5,022	2,357	8,518	28	77.01	36.15	130.62	37
		2007	5,064	2,342	9,250	27	75.43	34.88	137.77	36

Metropolitan Statistical Area	PWID Population	Year	Number	Min	Max	Rank	Rate	Min	Max	Rank
Las Vegas, NV--AZ	Young (15-29)	1992	3,003	2,091	3,851	39	143.86	100.15	184.48	19
		1993	2,271	818	3,416	45	106.51	38.38	160.20	35
		1994	2,758	2,308	3,294	39	122.39	102.45	146.19	27
		1995	2,435	1,136	3,295	43	102.93	48.04	139.31	40
		1996	2,653	1,375	3,399	38	106.77	55.35	136.79	37
		1997	2,998	1,698	3,646	33	112.82	63.90	137.18	36
		1998	3,347	2,073	4,124	33	118.42	73.34	145.92	32
		1999	4,135	3,601	4,653	25	138.01	120.19	155.30	21
		2000	3,974	2,487	4,901	27	125.18	78.35	154.38	29
		2001	4,677	4,499	4,969	24	143.43	137.97	152.40	25
		2002	4,176	2,319	5,096	28	124.78	69.29	152.27	31
		2003	4,064	2,066	5,467	31	117.49	59.73	158.04	39
		2004	3,871	1,904	5,640	35	106.78	52.51	155.56	47
		2005	3,456	1,659	5,436	38	91.17	43.75	143.41	54
		2006	2,867	1,346	4,864	50	72.17	33.88	122.42	73
		2007	2,133	986	3,896	68	52.01	24.05	94.99	93
	Old (30-64)	1992	11,546	8,038	14,807	33	256.36	178.47	328.76	12
		1993	9,371	3,377	14,095	34	196.84	70.92	296.06	21
		1994	11,619	9,725	13,878	31	224.55	187.95	268.21	14
		1995	10,053	4,692	13,607	28	181.11	84.52	245.12	21
		1996	10,403	5,393	13,328	28	176.41	91.46	226.01	21
		1997	10,924	6,188	13,284	28	172.83	97.90	210.16	20
		1998	11,188	6,929	13,785	27	167.79	103.91	206.74	19
		1999	12,630	10,999	14,212	24	180.18	156.92	202.76	13
		2000	11,130	6,966	13,727	26	150.96	94.48	186.18	21
		2001	12,123	11,662	12,882	23	157.07	151.09	166.90	19
		2002	10,150	5,636	12,386	26	126.23	70.09	154.03	28
		2003	9,394	4,776	12,636	29	112.78	57.34	151.71	35
		2004	8,607	4,233	12,539	32	98.82	48.60	143.96	42
		2005	7,420	3,561	11,672	34	82.12	39.41	129.17	52
		2006	5,883	2,762	9,980	43	62.74	29.45	106.42	65
		2007	4,065	1,880	7,425	60	42.12	19.48	76.93	91

Metropolitan Statistical Area	PWID Population	Year	Number	Min	Max	Rank	Rate	Min	Max	Rank
Little Rock--North Little Rock, AR	Total	1992	6,244	5,029	7,601	67	177.54	143.01	216.13	21
		1993	5,499	3,824	7,148	72	153.14	106.49	199.03	28
		1994	5,815	4,833	6,636	72	159.99	132.96	182.58	22
		1995	5,133	3,840	6,107	76	139.62	104.45	166.12	33
		1996	4,954	3,813	5,811	77	132.76	102.17	155.72	34
		1997	4,758	3,740	5,738	81	125.65	98.76	151.53	36
		1998	4,588	3,651	5,647	82	119.82	95.35	147.46	41
		1999	4,727	4,163	5,588	83	121.38	106.91	143.49	36
		2000	4,399	3,531	5,520	83	111.27	89.31	139.63	40
		2001	4,620	3,852	5,419	83	115.84	96.57	135.88	41
		2002	4,218	2,807	5,322	84	104.78	69.75	132.22	41
		2003	4,181	2,438	5,603	85	102.82	59.96	137.79	43
		2004	4,145	2,036	6,149	86	100.73	49.47	149.45	43
		2005	4,112	1,620	6,717	88	98.63	38.86	161.11	43
		2006	4,116	1,199	7,402	88	96.99	28.26	174.41	46
		2007	4,093	765	8,054	86	95.39	17.84	187.71	45
	Non-Hispanic White	1992	4,131	3,327	5,028	55	149.46	120.38	181.94	20
		1993	3,829	2,663	4,976	57	136.69	95.06	177.66	23
		1994	4,230	3,515	4,827	56	150.01	124.67	171.19	17
		1995	3,874	2,899	4,610	53	136.84	102.37	162.81	19
		1996	3,857	2,968	4,524	54	135.42	104.21	158.83	18
		1997	3,799	2,986	4,581	56	132.23	103.93	159.46	17
		1998	3,738	2,974	4,600	54	129.43	103.00	159.28	16
		1999	3,910	3,444	4,623	55	133.96	117.99	158.37	18
		2000	3,679	2,953	4,617	56	124.85	100.21	156.67	17
		2001	3,891	3,244	4,564	59	131.60	109.71	154.36	16
		2002	3,560	2,370	4,493	60	120.09	79.94	151.55	17
		2003	3,522	2,054	4,720	62	118.13	68.89	158.31	18
		2004	3,466	1,702	5,143	67	115.58	56.76	171.48	23
		2005	3,393	1,337	5,542	69	112.35	44.27	183.51	25
		2006	3,325	969	5,980	72	109.00	31.76	196.02	26
		2007	3,205	599	6,307	77	104.44	19.53	205.52	29

Metropolitan Statistical Area	PWID Population	Year	Number	Min	Max	Rank	Rate	Min	Max	Rank
Little Rock--North Little Rock, AR	Non-Hispanic Black	1992	1,793	1,444	2,182	60	264.16	212.77	321.57	56
		1993	1,383	962	1,798	63	195.37	135.86	253.93	67
		1994	1,272	1,057	1,451	65	175.34	145.72	200.09	77
		1995	972	727	1,156	68	130.37	97.53	155.11	87
		1996	811	624	951	75	105.25	81.00	123.46	91
		1997	676	532	816	83	85.52	67.22	103.14	94
		1998	571	454	703	86	70.52	56.12	86.79	95
		1999	522	460	617	86	62.74	55.26	74.17	96
		2000	440	353	552	88	51.65	41.46	64.82	97
		2001	428	356	502	88	49.26	41.06	57.78	98
		2002	372	248	469	91	41.91	27.90	52.89	100
		2003	363	212	486	88	40.16	23.42	53.82	100
		2004	368	181	546	88	39.76	19.53	58.99	100
		2005	389	153	635	88	41.08	16.19	67.10	100
		2006	433	126	779	87	44.26	12.90	79.59	98
		2007	504	94	991	85	50.54	9.45	99.46	94
	Hispanic	1992	19	15	23	96	58.55	47.16	71.28	79
		1993	16	11	21	98	44.15	30.70	57.38	87
		1994	17	14	20	99	41.64	34.61	47.52	89
		1995	16	12	19	99	33.16	24.81	39.45	91
		1996	17	13	19	99	28.64	22.04	33.59	92
		1997	18	14	21	98	27.68	21.76	33.39	91
		1998	19	15	24	97	27.42	21.82	33.74	88
		1999	23	20	27	95	29.48	25.97	34.85	87
		2000	25	20	31	95	29.32	23.53	36.79	82
		2001	31	26	37	93	34.24	28.55	40.17	81
		2002	34	23	43	93	34.93	23.25	44.08	76
		2003	40	24	54	92	39.26	22.90	52.62	69
		2004	48	24	71	91	44.02	21.62	65.31	67
		2005	57	23	94	89	48.44	19.09	79.12	65
		2006	68	20	123	89	54.20	15.79	97.46	62
		2007	81	15	159	88	60.01	11.22	118.10	60

Metropolitan Statistical Area	PWID Population	Year	Number	Min	Max	Rank	Rate	Min	Max	Rank
Little Rock--North Little Rock, AR	Male	1992	5,224	4,208	6,360	56	305.68	246.21	372.11	12
		1993	4,403	3,062	5,722	61	252.19	175.38	327.78	16
		1994	4,476	3,720	5,108	61	253.03	210.28	288.74	17
		1995	3,828	2,864	4,555	68	213.64	159.84	254.20	23
		1996	3,613	2,781	4,238	71	198.37	152.66	232.67	27
		1997	3,425	2,692	4,131	75	185.13	145.51	223.26	29
		1998	3,287	2,616	4,045	76	175.58	139.72	216.07	33
		1999	3,392	2,987	4,010	77	177.94	156.73	210.36	32
		2000	3,175	2,548	3,984	78	163.97	131.60	205.75	32
		2001	3,361	2,802	3,942	78	172.21	143.56	201.99	31
		2002	3,092	2,058	3,902	79	157.03	104.52	198.16	35
		2003	3,082	1,797	4,130	78	155.08	90.45	207.83	32
		2004	3,060	1,503	4,540	82	152.07	74.69	225.63	32
		2005	3,023	1,191	4,938	82	148.28	58.43	242.20	33
		2006	2,988	871	5,373	84	143.86	41.92	258.70	32
		2007	2,901	542	5,708	82	138.15	25.83	271.85	35
	Female	1992	1,025	826	1,248	88	56.72	45.69	69.05	65
		1993	1,099	764	1,429	85	59.57	41.42	77.42	59
		1994	1,343	1,116	1,533	85	71.99	59.83	82.15	49
		1995	1,313	982	1,562	84	69.65	52.11	82.87	52
		1996	1,355	1,043	1,590	84	70.94	54.59	83.21	52
		1997	1,354	1,064	1,633	86	69.91	54.95	84.32	52
		1998	1,329	1,057	1,635	86	67.89	54.02	83.55	54
		1999	1,369	1,206	1,619	87	68.87	60.66	81.42	53
		2000	1,259	1,010	1,580	87	62.41	50.09	78.32	58
		2001	1,295	1,080	1,519	87	63.61	53.03	74.61	57
		2002	1,154	768	1,457	89	56.14	37.37	70.84	65
		2003	1,118	652	1,498	93	53.77	31.36	72.06	66
		2004	1,089	535	1,615	94	51.79	25.43	76.84	69
		2005	1,072	423	1,752	94	50.34	19.83	82.22	69
		2006	1,082	315	1,946	95	49.93	14.55	89.79	71
		2007	1,107	207	2,178	95	50.51	9.44	99.39	71

Metropolitan Statistical Area	PWID Population	Year	Number	Min	Max	Rank	Rate	Min	Max	Rank
Little Rock--North Little Rock, AR	Young (15-29)	1992	2,731	2,200	3,325	41	222.70	179.38	271.10	6
		1993	2,265	1,575	2,944	46	183.77	127.80	238.86	8
		1994	2,291	1,904	2,614	45	186.28	154.81	212.57	9
		1995	1,962	1,468	2,334	52	158.92	118.90	189.09	12
		1996	1,859	1,431	2,181	57	148.60	114.36	174.30	13
		1997	1,770	1,391	2,135	61	140.07	110.09	168.92	16
		1998	1,703	1,356	2,096	64	134.11	106.72	165.05	22
		1999	1,758	1,549	2,078	65	137.43	121.04	162.46	22
		2000	1,640	1,317	2,058	70	128.04	102.76	160.67	26
		2001	1,724	1,437	2,022	73	135.74	113.16	159.22	29
		2002	1,566	1,042	1,976	77	123.63	82.29	156.01	32
		2003	1,532	894	2,053	80	120.41	70.22	161.37	34
		2004	1,482	728	2,199	82	115.70	56.83	171.67	38
		2005	1,413	557	2,309	88	109.23	43.04	178.41	42
		2006	1,334	389	2,399	88	101.20	29.49	181.98	47
		2007	1,222	229	2,405	87	92.26	17.25	181.56	52
	Old (30-64)	1992	3,627	2,921	4,415	77	158.35	127.55	192.77	38
		1993	3,299	2,294	4,287	80	139.86	97.26	181.78	39
		1994	3,575	2,971	4,080	78	148.67	123.55	169.65	37
		1995	3,215	2,405	3,825	81	131.65	98.50	156.64	39
		1996	3,146	2,421	3,690	81	126.81	97.59	148.74	39
		1997	3,050	2,397	3,678	81	120.87	95.00	145.77	40
		1998	2,958	2,354	3,641	83	115.59	91.98	142.24	41
		1999	3,056	2,691	3,612	82	116.85	102.92	138.13	39
		2000	2,844	2,283	3,569	82	106.43	85.42	133.56	44
		2001	2,981	2,485	3,497	80	109.67	91.43	128.64	42
		2002	2,713	1,806	3,423	85	98.34	65.46	124.10	47
		2003	2,679	1,562	3,590	85	95.87	55.91	128.48	47
		2004	2,644	1,299	3,923	84	93.32	45.83	138.45	46
		2005	2,614	1,030	4,269	85	90.91	35.82	148.49	44
		2006	2,608	760	4,690	84	89.16	25.98	160.33	44
		2007	2,587	484	5,092	83	87.24	16.31	171.68	42

Metropolitan Statistical Area	PWID Population	Year	Number	Min	Max	Rank	Rate	Min	Max	Rank
Los Angeles--Long Beach, CA	Total	1992	97,375	60,902	141,958	2	160.87	100.62	234.53	26
		1993	106,117	60,681	141,173	2	175.67	100.45	233.70	21
		1994	91,225	60,320	129,242	2	151.88	100.43	215.18	29
		1995	97,883	59,971	127,287	2	163.88	100.40	213.11	20
		1996	94,501	59,884	120,957	2	158.01	100.13	202.25	20
		1997	92,145	60,493	115,788	2	152.10	99.85	191.13	23
		1998	90,679	61,103	112,996	2	147.15	99.16	183.37	24
		1999	83,516	61,670	111,956	2	133.33	98.46	178.74	29
		2000	86,946	61,154	111,728	2	136.93	96.31	175.97	25
		2001	82,286	60,558	111,372	2	127.96	94.17	173.19	30
		2002	82,228	59,025	114,998	2	126.37	90.71	176.73	30
		2003	79,951	57,300	118,389	2	121.73	87.25	180.26	30
		2004	77,715	54,988	122,804	2	117.46	83.11	185.61	29
		2005	75,228	51,781	126,920	2	113.19	77.91	190.96	32
		2006	72,479	41,544	130,944	3	108.83	62.38	196.62	33
		2007	69,664	31,253	134,870	3	104.48	46.87	202.27	38
	Non-Hispanic White	1992	34,194	21,387	49,850	1	143.06	89.48	208.56	22
		1993	35,777	20,459	47,596	1	154.38	88.28	205.37	15
		1994	29,635	19,596	41,985	1	132.18	87.40	187.27	23
		1995	30,760	18,846	40,000	1	141.47	86.67	183.97	16
		1996	28,853	18,284	36,931	2	135.58	85.92	173.54	17
		1997	27,468	18,032	34,515	2	129.97	85.32	163.31	18
		1998	26,532	17,878	33,062	2	125.75	84.74	156.70	18
		1999	24,128	17,817	32,345	3	114.62	84.64	153.65	25
		2000	24,962	17,557	32,077	3	119.32	83.92	153.33	19
		2001	23,638	17,396	31,994	3	113.06	83.20	153.02	25
		2002	23,808	17,090	33,297	4	114.11	81.91	159.58	22
		2003	23,510	16,849	34,813	4	113.26	81.17	167.71	23
		2004	23,387	16,548	36,956	4	113.21	80.10	178.89	24
		2005	23,347	16,070	39,390	3	113.89	78.39	192.15	24
		2006	23,369	13,395	42,219	3	114.95	65.89	207.67	22
		2007	23,495	10,540	45,487	3	116.72	52.36	225.97	23

Metropolitan Statistical Area	PWID Population	Year	Number	Min	Max	Rank	Rate	Min	Max	Rank
Los Angeles--Long Beach, CA	Non-Hispanic Black	1992	16,512	10,328	24,073	6	262.11	163.94	382.12	57
		1993	19,586	11,200	26,056	4	314.36	179.76	418.21	41
		1994	17,709	11,709	25,089	6	287.69	190.23	407.58	46
		1995	19,437	11,909	25,276	3	319.60	195.81	415.60	31
		1996	18,777	11,899	24,034	3	311.05	197.11	398.13	26
		1997	18,015	11,827	22,637	3	297.11	195.05	373.34	24
		1998	17,238	11,616	21,481	3	282.56	190.40	352.10	24
		1999	15,334	11,323	20,556	7	249.86	184.50	334.95	28
		2000	15,395	10,828	19,783	4	251.18	176.67	322.78	23
		2001	14,104	10,380	19,090	7	229.49	168.89	310.61	26
		2002	13,772	9,886	19,261	7	223.43	160.39	312.48	25
		2003	13,284	9,521	19,671	6	215.33	154.32	318.85	27
		2004	13,078	9,254	20,666	7	211.67	149.77	334.48	28
		2005	13,163	9,060	22,208	7	213.54	146.99	360.28	26
		2006	13,602	7,797	24,575	5	221.92	127.20	400.93	24
		2007	14,514	6,512	28,100	4	238.31	106.91	461.36	22
	Hispanic	1992	35,218	22,027	51,342	2	152.88	95.62	222.87	45
		1993	38,358	21,934	51,029	2	163.34	93.40	217.30	39
		1994	32,938	21,779	46,665	2	138.28	91.43	195.90	40
		1995	35,307	21,632	45,913	2	146.55	89.79	190.57	37
		1996	34,082	21,597	43,623	2	138.95	88.05	177.84	37
		1997	33,277	21,846	41,815	2	132.04	86.68	165.92	37
		1998	32,864	22,145	40,953	2	126.34	85.13	157.43	37
		1999	30,465	22,496	40,839	2	113.73	83.98	152.46	37
		2000	32,038	22,534	41,170	2	116.32	81.82	149.48	37
		2001	30,763	22,640	41,637	2	109.10	80.29	147.67	39
		2002	31,347	22,501	43,839	2	108.75	78.06	152.09	37
		2003	31,258	22,402	46,286	2	106.34	76.21	157.46	37
		2004	31,359	22,188	49,553	2	105.01	74.30	165.94	36
		2005	31,545	21,713	53,220	2	104.28	71.78	175.94	38
		2006	31,808	18,232	57,466	2	104.20	59.73	188.26	36
		2007	32,224	14,456	62,385	2	104.64	46.94	202.58	35

Metropolitan Statistical Area	PWID Population	Year	Number	Min	Max	Rank	Rate	Min	Max	Rank
Los Angeles--Long Beach, CA	Male	1992	64,612	40,411	94,195	2	210.48	131.64	306.85	29
		1993	70,990	40,595	94,442	2	232.21	132.78	308.92	21
		1994	61,391	40,593	86,974	2	202.33	133.78	286.64	31
		1995	66,145	40,526	86,015	2	219.64	134.57	285.61	19
		1996	64,038	40,580	81,966	2	212.84	134.87	272.42	20
		1997	62,558	41,069	78,608	2	205.71	135.05	258.49	21
		1998	61,642	41,537	76,813	2	199.62	134.51	248.75	21
		1999	56,836	41,969	76,191	2	181.36	133.92	243.12	29
		2000	59,249	41,673	76,137	2	186.59	131.24	239.77	22
		2001	56,180	41,345	76,038	2	174.58	128.48	236.28	27
		2002	56,300	40,413	78,737	2	172.83	124.06	241.71	24
		2003	54,967	39,394	81,394	2	167.20	119.83	247.58	26
		2004	53,738	38,023	84,916	2	162.16	114.74	256.24	25
		2005	52,418	36,081	88,437	2	157.30	108.28	265.40	27
		2006	51,001	29,234	92,142	2	152.74	87.55	275.95	31
		2007	49,621	22,261	96,066	3	148.32	66.54	287.16	32
	Female	1992	32,288	20,194	47,071	1	108.24	67.70	157.79	28
		1993	34,996	20,012	46,557	1	117.30	67.07	156.05	23
		1994	29,961	19,811	42,447	2	100.81	66.66	142.82	31
		1995	32,043	19,632	41,669	1	108.20	66.29	140.71	22
		1996	30,846	19,547	39,482	2	103.79	65.77	132.85	22
		1997	29,987	19,686	37,680	2	99.39	65.25	124.89	23
		1998	29,403	19,812	36,639	2	95.64	64.44	119.18	23
		1999	26,952	19,902	36,130	2	86.11	63.59	115.44	35
		2000	27,881	19,610	35,828	2	87.84	61.78	112.88	32
		2001	26,164	19,255	35,412	1	81.44	59.94	110.23	39
		2002	25,855	18,559	36,159	2	79.56	57.11	111.27	37
		2003	24,781	17,760	36,695	2	75.55	54.15	111.87	41
		2004	23,656	16,738	37,381	3	71.64	50.69	113.20	44
		2005	22,392	15,413	37,779	4	67.57	46.51	114.00	49
		2006	20,994	12,034	37,929	4	63.22	36.24	114.22	55
		2007	19,530	8,762	37,811	4	58.78	26.37	113.81	54

Metropolitan Statistical Area	PWID Population	Year	Number	Min	Max	Rank	Rate	Min	Max	Rank
Los Angeles--Long Beach, CA	Young (15-29)	1992	23,030	14,404	33,574	1	101.88	63.72	148.52	40
		1993	22,526	12,881	29,968	1	101.97	58.31	135.66	42
		1994	17,830	11,790	25,261	2	82.71	54.69	117.18	54
		1995	18,029	11,046	23,444	2	85.39	52.31	111.04	51
		1996	16,734	10,604	21,419	3	80.09	50.75	102.51	54
		1997	15,946	10,469	20,037	3	75.97	49.87	95.46	56
		1998	15,526	10,462	19,347	3	73.16	49.29	91.16	58
		1999	14,264	10,533	19,121	4	66.51	49.11	89.16	65
		2000	14,871	10,460	19,110	4	68.61	48.26	88.17	65
		2001	14,090	10,369	19,070	5	65.25	48.02	88.31	75
		2002	14,032	10,072	19,624	5	65.09	46.72	91.03	74
		2003	13,477	9,659	19,957	5	62.61	44.87	92.70	79
		2004	12,773	9,038	20,184	5	59.32	41.97	93.73	83
		2005	11,846	8,154	19,986	8	54.99	37.85	92.78	94
		2006	10,696	6,131	19,324	8	49.65	28.46	89.71	98
		2007	9,379	4,208	18,157	11	43.55	19.54	84.32	100
	Old (30-64)	1992	76,678	47,958	111,786	2	202.19	126.46	294.77	19
		1993	83,748	47,890	111,415	2	218.57	124.98	290.77	14
		1994	72,596	48,002	102,849	2	188.54	124.66	267.10	19
		1995	78,856	48,314	102,544	2	204.21	125.11	265.55	14
		1996	77,210	48,927	98,825	2	198.42	125.74	253.97	13
		1997	76,343	50,119	95,931	2	192.83	126.59	242.30	13
		1998	76,057	51,250	94,776	2	188.26	126.86	234.59	12
		1999	70,716	52,218	94,798	2	171.67	126.77	230.14	15
		2000	74,045	52,080	95,150	2	177.06	124.54	227.53	12
		2001	70,160	51,634	94,960	1	164.27	120.89	222.33	15
		2002	69,800	50,104	97,618	2	160.41	115.15	224.34	15
		2003	67,079	48,075	99,328	2	151.94	108.89	224.98	16
		2004	63,800	45,142	100,815	2	142.96	101.15	225.90	15
		2005	59,532	40,977	100,439	2	132.53	91.22	223.59	19
		2006	54,000	30,953	97,560	2	119.85	68.70	216.52	23
		2007	47,026	21,097	91,043	2	104.17	46.73	201.68	28

Metropolitan Statistical Area	PWID Population	Year	Number	Min	Max	Rank	Rate	Min	Max	Rank
Louisville, KY--IN	Total	1992	8,846	3,599	14,634	52	137.54	55.95	227.53	41
		1993	7,371	2,569	14,458	56	113.53	39.58	222.70	42
		1994	9,051	4,358	14,202	49	138.35	66.61	217.08	38
		1995	7,666	3,452	13,957	52	116.14	52.31	211.46	42
		1996	7,640	3,816	13,687	49	114.91	57.40	205.84	43
		1997	7,474	3,440	13,371	51	111.92	51.51	200.23	44
		1998	7,345	2,721	13,135	52	109.08	40.40	195.06	45
		1999	8,156	2,238	12,909	52	119.98	32.92	189.89	38
		2000	7,237	2,084	12,424	54	105.44	30.37	181.01	43
		2001	7,859	2,056	11,889	51	113.79	29.76	172.12	43
		2002	6,912	1,984	11,488	56	99.44	28.54	165.27	45
		2003	6,741	1,825	11,089	57	96.31	26.07	158.42	46
		2004	6,559	1,577	10,776	58	92.87	22.33	152.58	49
		2005	6,448	1,688	10,445	65	90.59	23.71	146.75	52
		2006	6,515	2,452	10,449	66	90.76	34.16	145.57	51
		2007	6,615	2,582	10,655	64	91.13	35.58	146.80	51
	Non-Hispanic White	1992	5,676	2,309	9,390	43	102.76	41.80	169.99	37
		1993	4,706	1,641	9,232	47	84.61	29.49	165.96	40
		1994	5,739	2,763	9,004	40	102.75	49.47	161.21	35
		1995	4,819	2,170	8,774	44	85.83	38.66	156.27	45
		1996	4,757	2,376	8,522	45	84.44	42.18	151.27	46
		1997	4,608	2,121	8,244	45	81.78	37.63	146.31	49
		1998	4,486	1,661	8,021	43	79.36	29.39	141.91	49
		1999	4,937	1,355	7,815	42	86.98	23.87	137.68	43
		2000	4,350	1,253	7,468	46	76.30	21.97	130.98	52
		2001	4,702	1,230	7,112	45	82.21	21.50	124.36	47
		2002	4,128	1,185	6,860	47	71.93	20.64	119.55	54
		2003	4,033	1,092	6,634	52	69.96	18.94	115.08	57
		2004	3,947	949	6,485	58	68.18	16.40	112.02	57
		2005	3,921	1,026	6,352	60	67.44	17.65	109.25	59
		2006	4,022	1,514	6,452	59	68.74	25.87	110.26	59
		2007	4,167	1,627	6,712	57	70.76	27.63	113.99	58

Metropolitan Statistical Area	PWID Population	Year	Number	Min	Max	Rank	Rate	Min	Max	Rank
Louisville, KY--IN	Non-Hispanic Black	1992	2,625	1,068	4,342	44	326.76	132.93	540.56	45
		1993	2,366	825	4,640	42	290.64	101.31	570.10	47
		1994	2,997	1,443	4,703	34	360.64	173.63	565.85	27
		1995	2,525	1,137	4,598	37	297.36	133.93	541.43	38
		1996	2,433	1,215	4,358	37	281.74	140.73	504.70	35
		1997	2,251	1,036	4,028	36	256.44	118.02	458.79	36
		1998	2,063	764	3,690	40	229.83	85.13	410.99	42
		1999	2,123	583	3,360	40	231.42	63.51	366.28	39
		2000	1,750	504	3,005	45	187.18	53.91	321.32	46
		2001	1,789	468	2,707	47	188.71	49.36	285.46	45
		2002	1,517	435	2,521	54	157.85	45.30	262.34	53
		2003	1,475	399	2,427	55	151.88	41.12	249.82	53
		2004	1,496	360	2,457	53	150.13	36.10	246.64	53
		2005	1,612	422	2,612	46	158.59	41.51	256.91	46
		2006	1,884	709	3,022	35	182.70	68.77	293.04	40
		2007	2,322	906	3,740	31	219.11	85.54	352.95	28
	Hispanic	1992	53	22	88	85	117.39	47.76	194.21	55
		1993	47	17	93	86	92.72	32.32	181.87	57
		1994	62	30	97	84	110.32	53.11	173.10	54
		1995	55	25	100	85	88.74	39.97	161.57	54
		1996	57	29	102	85	81.74	40.83	146.43	54
		1997	58	27	103	85	73.17	33.67	130.91	57
		1998	58	22	104	86	64.57	23.92	115.46	60
		1999	66	18	104	86	63.57	17.45	100.62	63
		2000	59	17	101	87	50.50	14.54	86.70	67
		2001	64	17	97	89	51.01	13.34	77.16	67
		2002	57	16	94	89	42.24	12.12	70.20	69
		2003	55	15	90	89	38.85	10.52	63.90	72
		2004	53	13	88	89	35.71	8.59	58.67	73
		2005	52	14	84	91	33.29	8.71	53.93	75
		2006	53	20	84	91	32.44	12.21	52.04	77
		2007	53	21	86	91	30.88	12.06	49.75	79

Metropolitan Statistical Area	PWID Population	Year	Number	Min	Max	Rank	Rate	Min	Max	Rank
Louisville, KY--IN	Male	1992	5,396	2,195	8,926	55	172.45	70.15	285.29	38
		1993	4,726	1,647	9,271	54	149.65	52.16	293.54	43
		1994	5,987	2,883	9,394	46	187.78	90.40	294.62	33
		1995	5,158	2,323	9,392	47	160.09	72.10	291.49	39
		1996	5,174	2,584	9,269	47	159.25	79.54	285.28	38
		1997	5,051	2,324	9,036	46	154.59	71.14	276.57	41
		1998	4,922	1,823	8,802	51	149.17	55.25	266.75	43
		1999	5,393	1,480	8,536	49	161.54	44.33	255.68	35
		2000	4,709	1,356	8,083	51	139.52	40.18	239.50	42
		2001	5,027	1,315	7,604	52	147.82	38.66	223.61	37
		2002	4,351	1,249	7,231	58	127.07	36.47	211.19	42
		2003	4,192	1,135	6,895	58	121.68	32.94	200.16	46
		2004	4,055	975	6,661	60	116.31	27.97	191.08	52
		2005	3,998	1,046	6,476	61	113.71	29.76	184.20	54
		2006	4,095	1,541	6,569	61	115.67	43.54	185.53	49
		2007	4,269	1,667	6,877	59	118.97	46.45	191.65	46
	Female	1992	3,636	1,479	6,015	40	110.08	44.78	182.11	26
		1993	2,818	982	5,527	48	84.51	29.46	165.77	38
		1994	3,292	1,585	5,166	44	98.18	47.27	154.04	33
		1995	2,711	1,221	4,935	52	80.24	36.14	146.09	42
		1996	2,676	1,337	4,794	52	78.70	39.31	140.99	43
		1997	2,632	1,211	4,709	54	77.18	35.52	138.08	47
		1998	2,631	974	4,704	57	76.59	28.37	136.97	46
		1999	2,991	821	4,734	53	86.46	23.73	136.85	34
		2000	2,726	785	4,680	54	78.16	22.51	134.17	45
		2001	3,040	795	4,598	53	86.68	22.67	131.12	35
		2002	2,733	784	4,542	53	77.48	22.24	128.77	39
		2003	2,704	732	4,447	55	76.06	20.59	125.11	38
		2004	2,640	635	4,337	60	73.81	17.75	121.27	41
		2005	2,567	672	4,158	60	71.25	18.65	115.42	44
		2006	2,518	948	4,039	63	69.23	26.06	111.04	43
		2007	2,427	947	3,909	62	66.12	25.81	106.51	47

Metropolitan Statistical Area	PWID Population	Year	Number	Min	Max	Rank	Rate	Min	Max	Rank
Louisville, KY--IN	Young (15-29)	1992	1,678	683	2,776	67	80.84	32.89	133.73	55
		1993	1,264	441	2,480	75	61.50	21.44	120.64	62
		1994	1,462	704	2,295	74	71.52	34.43	112.22	58
		1995	1,211	545	2,205	76	59.28	26.70	107.94	63
		1996	1,219	609	2,184	75	59.68	29.81	106.91	63
		1997	1,239	570	2,217	76	60.62	27.90	108.45	63
		1998	1,294	479	2,313	75	63.22	23.42	113.05	62
		1999	1,552	426	2,456	75	75.96	20.84	120.23	61
		2000	1,505	433	2,583	76	74.09	21.34	127.19	60
		2001	1,796	470	2,716	72	89.49	23.40	135.36	54
		2002	1,737	498	2,886	73	86.94	24.95	144.49	54
		2003	1,855	502	3,051	72	92.88	25.14	152.78	55
		2004	1,960	471	3,220	69	97.20	23.37	159.68	54
		2005	2,068	541	3,349	72	101.94	26.68	165.14	51
		2006	2,208	831	3,541	67	108.24	40.74	173.61	41
		2007	2,327	908	3,748	60	112.08	43.75	180.54	38
	Old (30-64)	1992	7,289	2,965	12,058	47	167.32	68.07	276.80	33
		1993	6,226	2,170	12,212	46	140.33	48.92	275.27	38
		1994	7,757	3,735	12,171	42	172.48	83.04	270.62	27
		1995	6,616	2,980	12,047	44	145.17	65.38	264.31	34
		1996	6,602	3,298	11,828	44	143.34	71.60	256.78	34
		1997	6,432	2,960	11,508	45	138.81	63.88	248.34	34
		1998	6,264	2,320	11,201	45	133.62	49.49	238.94	35
		1999	6,855	1,881	10,849	43	144.16	39.56	228.16	29
		2000	5,961	1,717	10,233	45	123.35	35.52	211.74	37
		2001	6,306	1,649	9,539	46	128.68	33.66	194.66	33
		2002	5,368	1,541	8,921	48	108.37	31.10	180.11	38
		2003	5,034	1,363	8,281	53	100.63	27.24	165.53	44
		2004	4,680	1,125	7,689	56	92.75	22.30	152.38	47
		2005	4,371	1,144	7,080	59	85.87	22.48	139.10	49
		2006	4,175	1,572	6,697	61	81.26	30.58	130.33	49
		2007	3,998	1,561	6,440	61	77.14	30.12	124.26	51

Metropolitan Statistical Area	PWID Population	Year	Number	Min	Max	Rank	Rate	Min	Max	Rank
Memphis, TN--AR--MS	Total	1992	4,822	3,155	5,868	78	70.89	46.38	86.28	80
		1993	4,826	3,077	6,102	78	70.27	44.80	88.85	76
		1994	4,946	3,034	6,384	78	70.98	43.54	91.62	82
		1995	4,842	2,992	6,680	79	68.42	42.28	94.39	75
		1996	4,865	2,977	6,977	79	67.76	41.46	97.18	76
		1997	4,883	2,952	7,260	79	67.24	40.64	99.97	73
		1998	4,924	2,965	7,587	80	66.94	40.31	103.13	70
		1999	5,438	2,982	7,929	75	72.91	39.98	106.30	70
		2000	5,089	3,142	8,313	78	67.38	41.60	110.07	68
		2001	5,769	3,288	8,662	74	75.82	43.22	113.84	67
		2002	5,257	3,235	9,012	76	68.47	42.13	117.38	68
		2003	5,349	3,099	9,373	75	69.01	39.99	120.92	69
		2004	5,457	2,993	9,735	74	69.63	38.20	124.23	69
		2005	5,562	2,867	10,103	74	70.21	36.19	127.53	69
		2006	5,694	2,750	10,525	73	70.79	34.18	130.84	68
		2007	5,240	5,240	5,240	77	64.63	64.63	64.63	72
	Non-Hispanic White	1992	2,001	1,309	2,436	83	50.35	32.94	61.27	73
		1993	2,047	1,305	2,588	80	51.63	32.91	65.28	65
		1994	2,155	1,322	2,781	80	54.14	33.21	69.88	68
		1995	2,176	1,344	3,001	78	54.41	33.62	75.06	62
		1996	2,260	1,383	3,241	78	56.38	34.49	80.85	62
		1997	2,349	1,420	3,493	78	58.57	35.40	87.09	61
		1998	2,454	1,478	3,781	78	61.10	36.79	94.14	60
		1999	2,806	1,538	4,091	76	69.67	38.20	101.58	58
		2000	2,714	1,675	4,434	76	67.26	41.53	109.88	57
		2001	3,174	1,809	4,765	69	78.85	44.94	118.39	52
		2002	2,974	1,830	5,099	72	74.01	45.54	126.87	53
		2003	3,102	1,798	5,436	71	77.25	44.76	135.36	48
		2004	3,232	1,773	5,765	71	80.56	44.19	143.72	45
		2005	3,349	1,726	6,083	71	83.50	43.04	151.67	45
		2006	3,470	1,675	6,413	71	86.25	41.65	159.42	42
		2007	3,215	3,215	3,215	76	80.30	80.30	80.30	45

Metropolitan Statistical Area	PWID Population	Year	Number	Min	Max	Rank	Rate	Min	Max	Rank
Memphis, TN--AR--MS	Non-Hispanic Black	1992	2,258	1,477	2,748	48	84.37	55.20	102.68	99
		1993	2,233	1,424	2,824	45	81.58	52.01	103.15	99
		1994	2,237	1,372	2,888	46	79.80	48.95	103.00	98
		1995	2,120	1,310	2,925	42	73.83	45.62	101.85	98
		1996	2,046	1,252	2,935	44	69.61	42.59	99.84	98
		1997	1,961	1,185	2,915	46	65.40	39.53	97.24	96
		1998	1,879	1,132	2,895	47	61.45	37.00	94.68	96
		1999	1,968	1,079	2,870	44	63.08	34.59	91.97	95
		2000	1,749	1,080	2,857	46	55.13	34.03	90.06	95
		2001	1,889	1,077	2,837	43	58.63	33.42	88.03	94
		2002	1,653	1,017	2,833	48	50.39	31.00	86.38	95
		2003	1,632	946	2,859	46	48.82	28.29	85.54	96
		2004	1,639	899	2,924	45	48.02	26.34	85.67	96
		2005	1,674	863	3,040	43	48.11	24.80	87.39	96
		2006	1,753	846	3,240	40	49.16	23.74	90.86	94
		2007	1,687	1,687	1,687	50	46.47	46.47	46.47	95
	Hispanic	1992	34	22	42	89	49.54	32.41	60.30	87
		1993	35	23	45	88	45.68	29.12	57.76	84
		1994	37	23	48	91	41.80	25.64	53.95	88
		1995	36	22	50	89	35.65	22.03	49.18	88
		1996	36	22	52	89	30.78	18.83	44.14	89
		1997	36	22	53	89	26.84	16.22	39.90	92
		1998	36	21	55	92	22.98	13.84	35.41	92
		1999	39	21	57	92	22.03	12.08	32.12	92
		2000	36	22	59	92	17.95	11.08	29.32	93
		2001	40	23	61	92	19.29	10.99	28.96	92
		2002	37	23	63	92	16.78	10.33	28.77	94
		2003	38	22	67	93	16.77	9.72	29.39	94
		2004	41	22	73	93	16.89	9.26	30.13	94
		2005	44	23	80	92	17.35	8.95	31.52	95
		2006	49	24	91	92	18.24	8.81	33.71	95
		2007	51	51	51	93	18.02	18.02	18.02	94

Metropolitan Statistical Area	PWID Population	Year	Number	Min	Max	Rank	Rate	Min	Max	Rank
Memphis, TN--AR--MS	Male	1992	3,285	2,149	3,998	79	100.46	65.73	122.26	79
		1993	3,272	2,086	4,137	77	99.09	63.17	125.29	73
		1994	3,323	2,038	4,289	79	99.13	60.80	127.94	78
		1995	3,210	1,983	4,428	79	94.26	58.24	130.02	73
		1996	3,171	1,940	4,547	79	91.71	56.11	131.53	75
		1997	3,120	1,886	4,639	81	89.18	53.90	132.59	74
		1998	3,077	1,853	4,741	82	86.77	52.25	133.69	72
		1999	3,319	1,820	4,839	78	92.13	50.51	134.32	71
		2000	3,030	1,871	4,951	83	82.98	51.23	135.57	71
		2001	3,354	1,911	5,035	80	91.18	51.97	136.89	67
		2002	2,988	1,838	5,122	82	80.54	49.56	138.06	72
		2003	2,980	1,727	5,222	83	79.65	46.15	139.55	73
		2004	2,992	1,641	5,338	84	78.96	43.31	140.86	73
		2005	3,019	1,556	5,483	83	78.78	40.60	143.09	74
		2006	3,079	1,487	5,692	80	79.27	38.28	146.52	74
		2007	2,846	2,846	2,846	84	72.63	72.63	72.63	79
	Female	1992	1,627	1,065	1,980	74	46.07	30.14	56.07	80
		1993	1,634	1,042	2,066	76	45.83	29.22	57.95	78
		1994	1,708	1,047	2,204	76	47.22	28.96	60.95	82
		1995	1,726	1,067	2,381	77	47.01	29.05	64.85	78
		1996	1,807	1,105	2,591	77	48.53	29.70	69.61	76
		1997	1,899	1,148	2,824	76	50.46	30.50	75.02	72
		1998	2,009	1,210	3,096	72	52.74	31.76	81.26	68
		1999	2,326	1,275	3,391	66	60.30	33.06	87.92	60
		2000	2,272	1,402	3,711	65	58.24	35.95	95.14	61
		2001	2,672	1,523	4,012	60	67.98	38.75	102.07	54
		2002	2,505	1,542	4,295	60	63.14	38.85	108.24	58
		2003	2,597	1,505	4,550	58	64.76	37.53	113.48	57
		2004	2,666	1,462	4,756	58	65.89	36.14	117.54	52
		2005	2,696	1,390	4,897	58	65.93	33.98	119.75	52
		2006	2,692	1,300	4,976	57	64.73	31.26	119.65	52
		2007	2,366	2,366	2,366	65	56.49	56.49	56.49	61

Metropolitan Statistical Area	PWID Population	Year	Number	Min	Max	Rank	Rate	Min	Max	Rank
Memphis, TN--AR--MS	Young (15-29)	1992	1,585	1,037	1,928	71	65.87	43.09	80.16	66
		1993	1,350	861	1,707	73	56.53	36.04	71.47	67
		1994	1,233	756	1,591	76	51.65	31.68	66.66	76
		1995	1,125	695	1,552	78	47.01	29.05	64.85	78
		1996	1,100	673	1,578	79	45.68	27.95	65.52	83
		1997	1,114	673	1,656	80	46.13	27.88	68.59	78
		1998	1,167	703	1,798	80	47.98	28.89	73.93	79
		1999	1,367	750	1,993	80	55.99	30.70	81.63	78
		2000	1,376	849	2,247	79	56.27	34.74	91.93	74
		2001	1,686	961	2,532	75	69.66	39.70	104.59	69
		2002	1,659	1,021	2,843	75	68.82	42.35	117.98	71
		2003	1,807	1,047	3,167	73	74.79	43.34	131.05	67
		2004	1,948	1,069	3,475	72	79.77	43.75	142.31	63
		2005	2,060	1,062	3,741	73	83.64	43.11	151.92	64
		2006	2,137	1,032	3,950	68	85.68	41.37	158.36	58
		2007	1,938	1,938	1,938	73	77.00	77.00	77.00	66
	Old (30-64)	1992	3,305	2,162	4,022	81	75.19	49.19	91.50	87
		1993	3,519	2,244	4,450	78	78.56	50.08	99.33	78
		1994	3,758	2,305	4,851	77	82.03	50.31	105.88	79
		1995	3,778	2,334	5,212	76	80.67	49.85	111.28	72
		1996	3,855	2,359	5,529	75	80.79	49.43	115.86	72
		1997	3,896	2,355	5,792	74	80.36	48.57	119.48	68
		1998	3,926	2,364	6,049	72	79.72	48.01	122.83	67
		1999	4,303	2,359	6,274	71	85.77	47.03	125.06	61
		2000	3,970	2,451	6,485	71	77.71	47.98	126.95	61
		2001	4,407	2,512	6,617	69	84.94	48.41	127.53	60
		2002	3,905	2,403	6,694	69	74.13	45.61	127.08	61
		2003	3,836	2,223	6,721	71	71.90	41.66	125.98	62
		2004	3,749	2,056	6,689	72	69.51	38.12	124.00	62
		2005	3,633	1,873	6,599	73	66.55	34.30	120.89	63
		2006	3,509	1,694	6,486	74	63.23	30.53	116.87	64
		2007	3,024	3,024	3,024	75	54.09	54.09	54.09	71

Metropolitan Statistical Area	PWID Population	Year	Number	Min	Max	Rank	Rate	Min	Max	Rank
Miami, FL	Total	1992	20,559	6,141	31,324	23	156.15	46.64	237.92	29
		1993	14,635	3,415	26,974	29	111.62	26.05	205.74	44
		1994	16,576	5,551	23,202	27	124.34	41.64	174.04	42
		1995	12,084	3,663	19,951	33	88.68	26.88	146.42	55
		1996	10,905	3,813	17,484	36	78.17	27.33	125.33	66
		1997	9,898	3,928	15,746	40	69.88	27.73	111.17	71
		1998	9,185	4,034	13,982	43	64.06	28.13	97.52	76
		1999	10,405	7,726	12,754	39	71.09	52.78	87.13	73
		2000	8,536	4,436	11,606	47	57.14	29.69	77.69	80
		2001	9,437	7,858	10,878	45	62.36	51.92	71.87	78
		2002	8,221	5,094	11,351	48	53.68	33.26	74.11	84
		2003	8,352	5,407	13,084	48	54.13	35.04	84.79	85
		2004	8,647	4,814	15,126	48	55.53	30.92	97.14	84
		2005	8,662	4,086	15,925	48	55.14	26.01	101.38	86
		2006	8,194	3,449	14,650	50	51.76	21.78	92.54	94
		2007	7,607	2,795	12,942	52	48.00	17.64	81.67	96
	Non-Hispanic White	1992	7,428	2,219	11,318	31	213.70	63.83	325.60	7
		1993	5,469	1,276	10,080	39	164.61	38.41	303.41	13
		1994	6,247	2,092	8,744	35	191.75	64.22	268.39	9
		1995	4,506	1,366	7,440	49	140.99	42.74	232.79	18
		1996	3,968	1,387	6,363	52	126.56	44.25	202.92	19
		1997	3,485	1,383	5,544	62	113.47	45.03	180.51	22
		1998	3,117	1,369	4,746	68	103.72	45.55	157.91	31
		1999	3,412	2,533	4,182	67	115.65	85.87	141.75	24
		2000	2,726	1,417	3,707	75	94.11	48.90	127.95	34
		2001	2,978	2,480	3,433	75	103.11	85.85	118.85	32
		2002	2,616	1,621	3,613	76	91.00	56.38	125.64	37
		2003	2,750	1,780	4,307	76	96.37	62.39	150.96	35
		2004	3,031	1,688	5,302	74	106.34	59.20	186.03	29
		2005	3,331	1,571	6,123	72	117.05	55.21	215.20	22
		2006	3,550	1,494	6,348	68	125.05	52.63	223.58	19
		2007	3,789	1,392	6,447	63	135.33	49.72	230.25	12

Metropolitan Statistical Area	PWID Population	Year	Number	Min	Max	Rank	Rate	Min	Max	Rank
Miami, FL	Non-Hispanic Black	1992	9,731	2,906	14,827	11	387.58	115.77	590.55	32
		1993	6,358	1,483	11,718	16	253.51	59.15	467.26	52
		1994	6,722	2,251	9,409	15	261.61	87.61	366.17	54
		1995	4,649	1,409	7,676	21	176.49	53.50	291.41	68
		1996	4,037	1,411	6,473	23	149.76	52.36	240.12	78
		1997	3,566	1,415	5,673	24	129.42	51.36	205.88	79
		1998	3,246	1,426	4,942	24	115.62	50.77	176.01	82
		1999	3,623	2,690	4,441	23	126.14	93.66	154.61	82
		2000	2,930	1,523	3,984	24	100.79	52.37	137.04	84
		2001	3,182	2,649	3,668	23	109.26	90.97	125.94	81
		2002	2,702	1,674	3,731	24	92.42	57.27	127.61	84
		2003	2,645	1,712	4,143	26	90.46	58.56	141.70	84
		2004	2,596	1,445	4,541	27	88.40	49.22	154.65	81
		2005	2,414	1,139	4,438	29	81.89	38.62	150.55	82
		2006	2,068	870	3,697	34	70.00	29.46	125.14	88
		2007	1,687	620	2,870	50	57.70	21.20	98.17	92
	Hispanic	1992	4,302	1,285	6,555	18	61.81	18.46	94.18	77
		1993	3,247	758	5,984	23	46.02	10.74	84.83	83
		1994	3,903	1,307	5,463	19	53.71	17.99	75.17	80
		1995	3,015	914	4,977	27	39.94	12.11	65.94	86
		1996	2,870	1,004	4,602	27	36.51	12.76	58.54	86
		1997	2,731	1,084	4,345	26	33.83	13.43	53.82	85
		1998	2,634	1,157	4,010	27	31.91	14.01	48.58	85
		1999	3,070	2,279	3,762	26	35.96	26.70	44.07	82
		2000	2,559	1,330	3,479	27	28.91	15.03	39.31	83
		2001	2,835	2,360	3,268	27	31.34	26.09	36.12	83
		2002	2,435	1,509	3,362	27	26.39	16.35	36.43	85
		2003	2,394	1,550	3,751	28	25.58	16.56	40.07	87
		2004	2,348	1,307	4,107	29	24.74	13.77	43.27	88
		2005	2,173	1,025	3,994	34	22.59	10.65	41.53	89
		2006	1,844	776	3,297	35	18.93	7.97	33.85	93
		2007	1,486	546	2,528	42	15.12	5.56	25.73	95

Metropolitan Statistical Area	PWID Population	Year	Number	Min	Max	Rank	Rate	Min	Max	Rank
Miami, FL	Male	1992	13,178	3,936	20,079	22	205.08	61.26	312.48	31
		1993	9,638	2,249	17,765	31	150.72	35.17	277.80	42
		1994	11,113	3,722	15,555	27	170.80	57.20	239.07	36
		1995	8,188	2,482	13,519	33	123.08	37.31	203.21	52
		1996	7,427	2,597	11,908	34	108.94	38.09	174.67	60
		1997	6,747	2,677	10,732	38	97.48	38.68	155.06	65
		1998	6,246	2,743	9,508	40	89.12	39.14	135.68	71
		1999	7,046	5,231	8,636	35	98.40	73.06	120.61	66
		2000	5,750	2,988	7,818	42	78.61	40.85	106.87	75
		2001	6,327	5,268	7,292	41	85.32	71.04	98.34	75
		2002	5,493	3,404	7,585	44	73.16	45.33	101.02	78
		2003	5,578	3,611	8,737	44	73.68	47.69	115.41	77
		2004	5,794	3,226	10,136	44	75.78	42.19	132.56	77
		2005	5,853	2,761	10,761	44	75.82	35.76	139.40	78
		2006	5,617	2,364	10,043	47	72.19	30.38	129.06	81
		2007	5,324	1,956	9,058	48	68.27	25.08	116.16	86
	Female	1992	7,158	2,138	10,906	20	106.20	31.72	161.81	31
		1993	4,914	1,147	9,057	28	73.17	17.07	134.86	45
		1994	5,424	1,816	7,592	27	79.48	26.62	111.25	43
		1995	3,888	1,179	6,420	37	55.77	16.90	92.07	63
		1996	3,478	1,216	5,577	39	48.77	17.05	78.19	75
		1997	3,150	1,250	5,010	45	43.48	17.26	69.17	80
		1998	2,929	1,286	4,459	48	39.96	17.55	60.84	86
		1999	3,337	2,477	4,090	44	44.62	33.13	54.69	87
		2000	2,756	1,432	3,747	53	36.16	18.79	49.16	92
		2001	3,068	2,555	3,536	52	39.75	33.09	45.81	88
		2002	2,687	1,665	3,710	54	34.41	21.32	47.51	93
		2003	2,735	1,770	4,284	53	34.79	22.52	54.51	94
		2004	2,823	1,572	4,939	53	35.62	19.83	62.32	95
		2005	2,802	1,322	5,152	54	35.08	16.55	64.50	95
		2006	2,606	1,097	4,659	58	32.37	13.63	57.88	97
		2007	2,355	865	4,007	66	29.26	10.75	49.79	98

Metropolitan Statistical Area	PWID Population	Year	Number	Min	Max	Rank	Rate	Min	Max	Rank
Miami, FL	Young (15-29)	1992	4,197	1,253	6,394	29	95.33	28.47	145.25	44
		1993	3,001	700	5,532	38	69.80	16.29	128.66	57
		1994	3,475	1,164	4,864	32	80.85	27.08	113.17	55
		1995	2,626	796	4,335	40	60.59	18.37	100.05	61
		1996	2,481	867	3,978	41	56.56	19.78	90.69	66
		1997	2,374	942	3,776	45	53.53	21.24	85.15	69
		1998	2,328	1,023	3,545	47	51.97	22.82	79.12	75
		1999	2,788	2,070	3,418	42	61.05	45.32	74.82	68
		2000	2,410	1,252	3,277	48	51.98	27.01	70.67	79
		2001	2,790	2,323	3,216	47	60.25	50.16	69.44	78
		2002	2,522	1,563	3,483	49	54.67	33.88	75.49	81
		2003	2,630	1,702	4,120	48	57.22	37.04	89.63	82
		2004	2,755	1,534	4,820	49	59.92	33.36	104.82	81
		2005	2,748	1,296	5,052	52	59.39	28.01	109.19	85
		2006	2,539	1,068	4,539	58	54.45	22.92	97.35	93
		2007	2,250	827	3,828	63	48.19	17.70	81.99	99
	Old (30-64)	1992	16,542	4,941	25,205	17	188.76	56.38	287.61	26
		1993	11,714	2,733	21,591	28	132.95	31.02	245.04	41
		1994	13,158	4,407	18,418	23	145.68	48.79	203.90	39
		1995	9,487	2,876	15,664	33	102.10	30.95	168.57	53
		1996	8,447	2,953	13,543	36	88.32	30.88	141.61	63
		1997	7,547	2,995	12,005	38	77.56	30.78	123.38	72
		1998	6,878	3,021	10,471	43	69.78	30.64	106.23	74
		1999	7,642	5,674	9,367	40	75.89	56.35	93.02	74
		2000	6,142	3,192	8,351	43	59.62	30.98	81.07	81
		2001	6,653	5,539	7,668	44	63.34	52.73	73.00	80
		2002	5,684	3,522	7,849	47	53.11	32.91	73.33	85
		2003	5,678	3,676	8,895	47	52.41	33.93	82.10	83
		2004	5,802	3,230	10,149	46	52.87	29.44	92.49	79
		2005	5,765	2,719	10,599	46	52.03	24.54	95.65	79
		2006	5,443	2,291	9,731	47	48.73	20.51	87.13	84
		2007	5,076	1,865	8,636	47	45.41	16.68	77.26	86

Metropolitan Statistical Area	PWID Population	Year	Number	Min	Max	Rank	Rate	Min	Max	Rank
Middlesex--Somerset--Hunterdon, NJ	Total	1992	5,581	3,293	7,654	71	77.07	45.47	105.69	74
		1993	5,459	3,547	7,143	73	74.91	48.67	98.01	71
		1994	5,305	3,828	6,633	76	72.29	52.15	90.38	80
		1995	5,229	4,119	6,162	73	70.64	55.64	83.25	73
		1996	5,151	4,421	5,743	74	68.79	59.04	76.69	73
		1997	5,101	4,747	5,370	76	67.10	62.44	70.64	74
		1998	5,086	5,006	5,164	76	65.96	64.92	66.97	73
		1999	5,068	4,686	5,579	81	64.81	59.93	71.35	78
		2000	5,100	4,380	5,707	77	64.28	55.20	71.93	72
		2001	5,066	4,089	5,831	81	62.99	50.85	72.50	76
		2002	5,158	3,810	5,692	77	63.41	46.83	69.98	74
		2003	5,170	3,540	6,100	76	63.01	43.14	74.34	75
		2004	5,168	3,289	6,530	77	62.42	39.74	78.89	76
		2005	5,177	3,059	6,973	78	61.94	36.60	83.43	76
		2006	5,162	2,824	7,408	79	61.56	33.68	88.35	75
		2007	5,172	2,600	7,879	79	61.23	30.78	93.26	76
	Non-Hispanic White	1992	3,382	1,995	4,638	66	60.10	35.46	82.43	63
		1993	3,198	2,077	4,184	65	57.44	37.32	75.15	62
		1994	3,042	2,195	3,804	69	55.18	39.81	68.99	64
		1995	2,971	2,340	3,501	67	54.36	42.82	64.07	63
		1996	2,930	2,515	3,267	68	53.98	46.33	60.17	65
		1997	2,931	2,727	3,085	71	54.16	50.40	57.02	65
		1998	2,971	2,924	3,016	72	55.11	54.24	55.95	64
		1999	3,024	2,796	3,329	74	56.37	52.12	62.05	67
		2000	3,117	2,677	3,487	68	58.29	50.06	65.23	64
		2001	3,173	2,562	3,653	71	59.65	48.15	68.66	65
		2002	3,311	2,445	3,654	65	62.63	46.26	69.12	62
		2003	3,394	2,324	4,004	67	64.72	44.31	76.35	61
		2004	3,458	2,201	4,370	68	66.34	42.23	83.83	61
		2005	3,518	2,079	4,739	67	67.90	40.12	91.46	58
		2006	3,546	1,940	5,088	69	69.06	37.78	99.10	58
		2007	3,570	1,795	5,439	69	70.05	35.21	106.70	59

Metropolitan Statistical Area	PWID Population	Year	Number	Min	Max	Rank	Rate	Min	Max	Rank
Middlesex--Somerset--Hunterdon, NJ	Non-Hispanic Black	1992	1,233	727	1,691	69	237.58	140.17	325.84	64
		1993	1,232	800	1,612	66	230.84	149.97	302.02	54
		1994	1,186	855	1,482	67	215.53	155.50	269.48	66
		1995	1,127	888	1,329	66	199.00	156.76	234.54	62
		1996	1,050	901	1,170	67	179.65	154.19	200.27	67
		1997	967	900	1,018	66	160.30	149.18	168.76	71
		1998	887	873	901	66	142.77	140.51	144.95	74
		1999	810	749	892	70	127.26	117.67	140.10	80
		2000	748	643	837	72	114.91	98.68	128.58	77
		2001	688	555	792	74	102.72	82.92	118.24	84
		2002	659	487	727	74	96.13	71.00	106.09	81
		2003	636	435	750	75	90.68	62.08	106.98	83
		2004	630	401	796	76	87.74	55.85	110.88	82
		2005	650	384	875	76	88.82	52.48	119.64	79
		2006	697	381	1,000	76	93.36	51.07	133.97	76
		2007	789	397	1,202	75	103.57	52.07	157.77	72
	Hispanic	1992	837	494	1,148	42	143.48	84.65	196.78	46
		1993	807	524	1,056	44	129.96	84.43	170.04	43
		1994	779	562	974	44	118.27	85.33	147.87	48
		1995	767	605	904	46	109.66	86.38	129.24	47
		1996	759	651	846	46	101.90	87.46	113.60	47
		1997	756	704	796	47	95.40	88.78	100.43	48
		1998	759	747	771	48	90.48	89.05	91.86	51
		1999	762	705	839	48	86.30	79.80	95.00	52
		2000	772	663	863	48	83.10	71.37	92.99	48
		2001	769	620	885	51	78.60	63.45	90.47	56
		2002	782	578	863	51	76.31	56.37	84.22	51
		2003	779	533	919	52	72.78	49.83	85.87	53
		2004	770	490	973	52	69.31	44.12	87.58	55
		2005	757	447	1,019	53	65.48	38.69	88.20	57
		2006	735	402	1,055	54	61.64	33.72	88.46	60
		2007	712	358	1,084	57	57.86	29.09	88.13	61

Metropolitan Statistical Area	PWID Population	Year	Number	Min	Max	Rank	Rate	Min	Max	Rank
Middlesex--Somerset--Hunterdon, NJ	Male	1992	3,809	2,247	5,224	71	105.38	62.17	144.52	73
		1993	3,654	2,374	4,781	71	100.49	65.29	131.48	69
		1994	3,488	2,517	4,361	77	95.31	68.76	119.17	82
		1995	3,384	2,666	3,988	75	91.70	72.24	108.08	75
		1996	3,288	2,822	3,666	76	88.12	75.64	98.24	76
		1997	3,218	2,994	3,387	77	84.98	79.08	89.46	77
		1998	3,177	3,127	3,226	79	82.77	81.46	84.03	76
		1999	3,141	2,905	3,458	82	80.68	74.60	88.81	79
		2000	3,143	2,699	3,517	79	79.50	68.28	88.97	74
		2001	3,109	2,510	3,579	83	77.52	62.58	89.23	79
		2002	3,158	2,333	3,486	78	77.77	57.44	85.83	75
		2003	3,164	2,166	3,733	77	77.15	52.82	91.02	75
		2004	3,165	2,015	3,999	79	76.46	48.67	96.63	75
		2005	3,177	1,878	4,280	79	75.94	44.88	102.30	77
		2006	3,179	1,739	4,562	77	75.70	41.41	108.63	75
		2007	3,199	1,608	4,873	77	75.48	37.94	114.98	76
	Female	1992	1,827	1,078	2,506	69	50.36	29.71	69.07	73
		1993	1,852	1,203	2,423	71	50.71	32.94	66.35	72
		1994	1,862	1,343	2,328	71	50.60	36.51	63.27	80
		1995	1,894	1,492	2,232	73	51.02	40.19	60.14	72
		1996	1,921	1,649	2,141	74	51.12	43.88	56.99	69
		1997	1,952	1,817	2,055	74	51.15	47.60	53.85	69
		1998	1,991	1,959	2,021	73	51.40	50.59	52.19	71
		1999	2,020	1,868	2,224	72	51.46	47.59	56.65	71
		2000	2,063	1,771	2,308	72	51.80	44.48	57.96	69
		2001	2,068	1,670	2,381	72	51.30	41.41	59.05	73
		2002	2,116	1,563	2,335	69	51.94	38.36	57.32	69
		2003	2,119	1,451	2,500	69	51.62	35.34	60.90	71
		2004	2,103	1,338	2,657	69	50.80	32.34	64.20	72
		2005	2,078	1,228	2,799	72	49.78	29.42	67.06	71
		2006	2,029	1,110	2,912	77	48.48	26.52	69.57	74
		2007	1,975	993	3,008	78	46.91	23.58	71.46	76

Metropolitan Statistical Area	PWID Population	Year	Number	Min	Max	Rank	Rate	Min	Max	Rank
Middlesex--Somerset--Hunterdon, NJ	Young (15-29)	1992	1,771	1,045	2,429	64	76.98	45.42	105.58	58
		1993	1,850	1,202	2,420	54	82.30	53.46	107.67	49
		1994	1,906	1,375	2,383	54	86.51	62.42	108.17	47
		1995	1,979	1,559	2,332	51	91.08	71.75	107.35	48
		1996	2,041	1,752	2,275	52	94.71	81.29	105.58	44
		1997	2,102	1,957	2,213	53	97.68	90.90	102.83	42
		1998	2,168	2,134	2,201	52	100.36	98.78	101.89	40
		1999	2,221	2,054	2,445	53	102.42	94.70	112.75	39
		2000	2,286	1,963	2,558	51	104.70	89.92	117.16	41
		2001	2,308	1,863	2,656	56	105.31	85.01	121.21	42
		2002	2,375	1,754	2,621	51	108.09	79.84	119.29	43
		2003	2,393	1,638	2,824	54	108.33	74.17	127.81	45
		2004	2,390	1,521	3,020	60	107.45	68.40	135.78	45
		2005	2,378	1,405	3,203	61	105.48	62.33	142.08	46
		2006	2,341	1,280	3,359	63	103.33	56.52	148.28	44
		2007	2,300	1,156	3,503	61	100.41	50.47	152.94	43
	Old (30-64)	1992	3,956	2,334	5,426	75	80.06	47.23	109.81	83
		1993	3,705	2,407	4,848	77	73.51	47.76	96.18	82
		1994	3,464	2,499	4,330	79	67.44	48.65	84.32	88
		1995	3,301	2,600	3,891	80	63.13	49.73	74.40	87
		1996	3,163	2,715	3,526	80	59.30	50.90	66.11	87
		1997	3,062	2,850	3,224	80	56.18	52.29	59.15	89
		1998	2,999	2,952	3,045	82	54.03	53.18	54.86	92
		1999	2,947	2,725	3,245	83	52.16	48.23	57.42	91
		2000	2,935	2,520	3,284	81	51.02	43.81	57.09	93
		2001	2,890	2,333	3,326	82	49.39	39.87	56.85	91
		2002	2,920	2,157	3,223	80	49.18	36.33	54.28	91
		2003	2,905	1,989	3,427	80	48.45	33.17	57.16	89
		2004	2,878	1,832	3,637	80	47.54	30.26	60.08	89
		2005	2,851	1,685	3,840	82	46.71	27.60	62.92	88
		2006	2,800	1,532	4,018	82	45.75	25.03	65.65	88
		2007	2,748	1,381	4,185	81	44.62	22.43	67.97	88

Metropolitan Statistical Area	PWID Population	Year	Number	Min	Max	Rank	Rate	Min	Max	Rank
Milwaukee--Waukesha, WI	Total	1992	5,876	3,792	7,799	69	61.88	39.94	82.13	90
		1993	4,940	2,235	7,660	76	51.72	23.40	80.20	93
		1994	5,794	4,028	7,500	73	60.40	41.99	78.19	85
		1995	4,995	2,714	7,348	77	51.75	28.12	76.14	90
		1996	5,060	2,953	7,335	76	52.11	30.40	75.53	89
		1997	5,127	3,165	7,398	74	52.71	32.54	76.06	88
		1998	5,226	3,381	7,561	74	53.60	34.68	77.54	85
		1999	5,925	4,613	7,776	69	60.52	47.11	79.43	80
		2000	5,467	3,959	7,863	73	55.54	40.21	79.87	83
		2001	6,045	4,892	8,002	68	60.86	49.25	80.56	81
		2002	5,771	4,580	8,280	70	57.54	45.66	82.56	79
		2003	5,980	4,893	8,721	70	59.14	48.39	86.24	77
		2004	6,336	5,050	9,804	68	62.22	49.58	96.27	77
		2005	6,710	4,975	10,969	58	65.51	48.57	107.09	72
		2006	7,101	4,899	12,203	54	68.95	47.57	118.49	70
		2007	7,504	4,817	13,492	53	72.54	46.57	130.43	67
	Non-Hispanic White	1992	2,377	1,534	3,155	78	30.89	19.94	41.00	96
		1993	2,131	964	3,304	76	27.72	12.54	42.98	97
		1994	2,558	1,779	3,312	74	33.41	23.22	43.24	94
		1995	2,190	1,190	3,222	77	28.66	15.57	42.16	95
		1996	2,153	1,256	3,121	79	28.22	16.47	40.91	94
		1997	2,080	1,284	3,001	81	27.43	16.93	39.58	94
		1998	1,999	1,293	2,892	82	26.49	17.14	38.32	96
		1999	2,128	1,656	2,793	84	28.30	22.03	37.15	95
		2000	1,850	1,340	2,661	86	24.66	17.85	35.46	98
		2001	1,953	1,580	2,585	85	25.93	20.99	34.33	98
		2002	1,821	1,445	2,612	88	24.10	19.12	34.57	100
		2003	1,901	1,556	2,773	89	25.10	20.54	36.61	100
		2004	2,112	1,683	3,267	84	27.84	22.18	43.07	99
		2005	2,445	1,813	3,998	81	32.23	23.89	52.69	96
		2006	2,948	2,033	5,065	78	38.88	26.82	66.81	91
		2007	3,660	2,349	6,581	67	48.33	31.02	86.89	81

Metropolitan Statistical Area	PWID Population	Year	Number	Min	Max	Rank	Rate	Min	Max	Rank
Milwaukee--Waukesha, WI	Non-Hispanic Black	1992	2,683	1,731	3,560	42	212.89	137.40	282.55	72
		1993	2,145	971	3,326	46	166.54	75.36	258.23	81
		1994	2,448	1,702	3,168	43	185.60	129.03	240.26	74
		1995	2,092	1,137	3,078	44	154.87	84.15	227.83	77
		1996	2,132	1,244	3,091	41	154.03	89.88	223.28	76
		1997	2,195	1,355	3,167	40	156.64	96.69	226.02	72
		1998	2,283	1,477	3,303	35	161.25	104.32	233.28	66
		1999	2,641	2,056	3,467	32	183.74	143.04	241.16	55
		2000	2,475	1,792	3,560	28	170.10	123.17	244.64	53
		2001	2,754	2,228	3,645	27	186.37	150.81	246.70	47
		2002	2,610	2,071	3,745	26	173.30	137.51	248.63	44
		2003	2,636	2,157	3,844	27	172.07	140.79	250.94	41
		2004	2,659	2,119	4,114	26	170.95	136.24	264.50	42
		2005	2,600	1,928	4,250	26	164.78	122.17	269.38	43
		2006	2,445	1,687	4,202	29	152.58	105.25	262.20	49
		2007	2,190	1,406	3,937	32	135.08	86.71	242.87	56
	Hispanic	1992	563	363	747	49	158.89	102.54	210.88	42
		1993	438	198	679	54	115.85	52.42	179.63	49
		1994	499	347	645	52	122.38	85.08	158.42	45
		1995	434	236	638	55	98.70	53.63	145.20	51
		1996	458	267	664	55	97.40	56.83	141.18	48
		1997	496	306	715	53	98.87	61.03	142.66	47
		1998	549	355	795	53	102.47	66.29	148.24	41
		1999	684	533	898	51	120.27	93.63	157.85	35
		2000	695	504	1,000	52	115.03	83.29	165.44	38
		2001	845	684	1,118	47	133.07	107.68	176.15	29
		2002	878	697	1,259	49	131.97	104.72	189.34	27
		2003	976	798	1,423	49	140.86	115.26	205.43	23
		2004	1,087	866	1,682	48	151.31	120.58	234.10	23
		2005	1,180	875	1,929	47	158.48	117.50	259.08	20
		2006	1,243	857	2,136	44	160.89	110.98	276.47	21
		2007	1,261	810	2,267	45	157.45	101.07	283.09	22

Metropolitan Statistical Area	PWID Population	Year	Number	Min	Max	Rank	Rate	Min	Max	Rank
Milwaukee--Waukesha, WI	Male	1992	4,243	2,738	5,631	65	91.26	58.90	121.12	84
		1993	3,526	1,595	5,467	74	75.31	34.08	116.78	87
		1994	4,103	2,853	5,311	67	87.20	60.62	112.88	85
		1995	3,522	1,914	5,181	72	74.30	40.38	109.31	86
		1996	3,563	2,079	5,165	73	74.66	43.56	108.22	85
		1997	3,613	2,231	5,214	71	75.57	46.65	109.04	82
		1998	3,692	2,389	5,341	68	76.97	49.80	111.35	80
		1999	4,198	3,268	5,510	64	87.11	67.82	114.33	76
		2000	3,885	2,813	5,588	66	80.11	58.01	115.21	73
		2001	4,306	3,484	5,699	63	87.90	71.13	116.35	73
		2002	4,114	3,265	5,903	61	83.08	65.92	119.19	69
		2003	4,257	3,483	6,208	57	85.14	69.67	124.17	68
		2004	4,492	3,580	6,950	56	89.09	71.00	137.85	67
		2005	4,720	3,499	7,715	53	92.95	68.91	151.95	63
		2006	4,933	3,403	8,476	51	96.59	66.63	165.98	62
		2007	5,117	3,285	9,200	52	99.68	63.99	179.23	60
	Female	1992	1,766	1,140	2,344	72	36.45	23.52	48.37	89
		1993	1,516	686	2,350	79	31.12	14.08	48.26	92
		1994	1,798	1,250	2,327	73	36.78	25.57	47.62	88
		1995	1,555	845	2,288	79	31.67	17.21	46.59	92
		1996	1,572	917	2,279	81	31.84	18.58	46.15	92
		1997	1,583	977	2,284	82	32.01	19.76	46.19	93
		1998	1,599	1,035	2,313	81	32.27	20.88	46.69	94
		1999	1,795	1,397	2,356	81	36.11	28.11	47.39	91
		2000	1,641	1,188	2,361	81	32.86	23.80	47.27	94
		2001	1,803	1,459	2,387	81	35.81	28.98	47.40	91
		2002	1,717	1,363	2,464	80	33.82	26.84	48.53	94
		2003	1,786	1,461	2,604	79	34.93	28.58	50.94	93
		2004	1,913	1,525	2,960	78	37.21	29.66	57.58	91
		2005	2,068	1,533	3,381	73	40.04	29.68	65.45	86
		2006	2,258	1,558	3,880	67	43.49	30.00	74.74	82
		2007	2,492	1,599	4,480	61	47.82	30.69	85.97	74

Metropolitan Statistical Area	PWID Population	Year	Number	Min	Max	Rank	Rate	Min	Max	Rank
Milwaukee--Waukesha, WI	Young (15-29)	1992	1,425	920	1,892	74	45.25	29.20	60.05	82
		1993	1,046	473	1,622	79	33.61	15.21	52.12	93
		1994	1,112	773	1,440	80	36.05	25.07	46.67	93
		1995	902	490	1,327	82	29.35	15.95	43.18	96
		1996	891	520	1,292	83	29.01	16.93	42.05	96
		1997	909	561	1,311	85	29.71	18.34	42.87	95
		1998	958	620	1,386	86	31.40	20.31	45.42	94
		1999	1,150	895	1,510	85	37.83	29.45	49.66	94
		2000	1,145	829	1,647	85	37.79	27.36	54.34	95
		2001	1,384	1,120	1,832	84	46.01	37.23	60.90	91
		2002	1,457	1,156	2,090	79	48.34	38.36	69.36	86
		2003	1,671	1,367	2,437	76	55.17	45.15	80.47	84
		2004	1,958	1,561	3,030	71	64.52	51.42	99.82	79
		2005	2,283	1,692	3,732	63	75.42	55.91	123.29	73
		2006	2,638	1,819	4,532	56	87.12	60.10	149.71	57
		2007	3,009	1,932	5,411	47	99.38	63.80	178.69	44
	Old (30-64)	1992	4,530	2,924	6,013	67	71.40	46.08	94.76	89
		1993	3,964	1,794	6,146	74	61.55	27.85	95.43	91
		1994	4,761	3,310	6,164	65	73.16	50.86	94.70	86
		1995	4,159	2,260	6,118	71	63.23	34.36	93.02	86
		1996	4,234	2,470	6,137	69	63.76	37.21	92.43	84
		1997	4,282	2,643	6,179	68	64.21	39.64	92.65	82
		1998	4,331	2,802	6,265	67	64.64	41.82	93.52	79
		1999	4,844	3,771	6,358	65	71.76	55.87	94.18	77
		2000	4,386	3,176	6,308	63	64.37	46.61	92.58	74
		2001	4,736	3,832	6,268	63	68.38	55.34	90.52	76
		2002	4,394	3,487	6,305	64	62.64	49.71	89.87	72
		2003	4,410	3,609	6,432	62	62.27	50.95	90.81	72
		2004	4,518	3,601	6,990	59	63.20	50.37	97.79	69
		2005	4,626	3,430	7,562	55	64.10	47.53	104.80	67
		2006	4,746	3,274	8,155	54	65.27	45.02	112.16	62
		2007	4,887	3,137	8,787	52	66.80	42.88	120.11	61

Metropolitan Statistical Area	PWID Population	Year	Number	Min	Max	Rank	Rate	Min	Max	Rank
Minneapolis--St. Paul, MN--WI	Total	1992	10,023	5,804	17,642	45	56.79	32.89	99.95	92
		1993	8,173	3,644	16,616	48	45.49	20.28	92.48	95
		1994	9,302	5,280	15,461	48	50.90	28.89	84.59	95
		1995	7,695	4,020	14,283	51	41.33	21.59	76.71	96
		1996	7,464	4,221	13,127	52	39.37	22.26	69.24	98
		1997	7,208	4,427	11,897	57	37.42	22.98	61.76	99
		1998	6,886	4,192	10,364	59	35.22	21.44	53.01	99
		1999	7,148	3,937	8,811	61	35.90	19.77	44.25	99
		2000	6,683	3,932	9,058	60	32.92	19.37	44.63	100
		2001	7,203	3,917	9,415	60	34.89	18.97	45.60	99
		2002	7,120	3,882	9,740	53	34.06	18.57	46.59	100
		2003	7,505	3,837	10,636	51	35.54	18.17	50.36	100
		2004	8,068	3,785	12,496	50	37.79	17.73	58.53	99
		2005	8,641	3,731	14,395	49	40.03	17.28	66.69	98
		2006	9,266	3,681	16,471	45	42.42	16.85	75.41	98
		2007	9,892	3,627	18,582	43	44.78	16.42	84.12	98
	Non-Hispanic White	1992	5,884	3,407	10,357	40	36.45	21.11	64.16	91
		1993	4,925	2,196	10,011	45	30.14	13.44	61.28	94
		1994	5,706	3,239	9,484	41	34.54	19.61	57.41	92
		1995	4,775	2,495	8,863	45	28.56	14.92	53.02	96
		1996	4,664	2,638	8,202	46	27.57	15.59	48.50	96
		1997	4,519	2,775	7,459	46	26.50	16.28	43.75	97
		1998	4,321	2,631	6,504	46	25.18	15.33	37.89	98
		1999	4,484	2,470	5,527	45	25.87	14.25	31.89	98
		2000	4,189	2,465	5,678	48	23.88	14.05	32.37	100
		2001	4,514	2,454	5,899	46	25.43	13.83	33.24	99
		2002	4,467	2,435	6,111	43	24.99	13.62	34.18	99
		2003	4,725	2,416	6,697	43	26.30	13.45	37.27	98
		2004	5,115	2,399	7,922	41	28.28	13.27	43.80	97
		2005	5,537	2,390	9,224	40	30.42	13.13	50.68	97
		2006	6,027	2,394	10,714	35	32.88	13.06	58.46	97
		2007	6,562	2,406	12,327	34	35.56	13.04	66.80	93

Metropolitan Statistical Area	PWID Population	Year	Number	Min	Max	Rank	Rate	Min	Max	Rank
Minneapolis--St. Paul, MN--WI	Non-Hispanic Black	1992	2,866	1,660	5,044	40	458.85	265.72	807.63	22
		1993	2,118	944	4,305	49	314.32	140.15	638.99	42
		1994	2,218	1,259	3,686	47	304.61	172.91	506.29	41
		1995	1,713	895	3,180	54	219.02	114.42	406.54	52
		1996	1,572	889	2,765	57	188.33	106.52	331.24	62
		1997	1,453	893	2,399	58	163.22	100.24	269.41	67
		1998	1,342	817	2,020	59	140.94	85.80	212.12	75
		1999	1,358	748	1,673	60	132.55	73.00	163.39	76
		2000	1,244	732	1,686	61	114.57	67.40	155.28	78
		2001	1,318	717	1,723	61	115.74	62.93	151.27	77
		2002	1,283	699	1,755	61	108.48	59.15	148.41	74
		2003	1,330	680	1,885	57	108.18	55.31	153.31	71
		2004	1,402	658	2,172	57	110.02	51.61	170.41	68
		2005	1,466	633	2,442	53	111.11	47.97	185.11	67
		2006	1,525	606	2,711	53	111.20	44.18	197.68	66
		2007	1,570	576	2,949	52	110.58	40.55	207.74	68
	Hispanic	1992	303	176	534	59	106.74	61.82	187.88	60
		1993	242	108	492	62	76.84	34.26	156.22	63
		1994	270	153	448	62	78.34	44.47	130.21	64
		1995	219	114	406	63	57.21	29.89	106.18	73
		1996	209	118	367	65	48.69	27.54	85.63	76
		1997	198	122	328	66	41.08	25.23	67.80	79
		1998	187	114	282	66	34.54	21.02	51.98	83
		1999	192	106	237	69	31.95	17.59	39.38	84
		2000	179	105	242	68	27.04	15.91	36.64	85
		2001	192	104	251	71	27.25	14.81	35.61	86
		2002	190	103	260	68	25.43	13.86	34.78	86
		2003	201	103	285	68	25.69	13.13	36.41	86
		2004	219	103	339	68	26.77	12.56	41.46	85
		2005	239	103	399	69	27.94	12.06	46.55	81
		2006	264	105	470	68	29.50	11.72	52.44	81
		2007	293	108	551	68	31.50	11.55	59.18	78

Metropolitan Statistical Area	PWID Population	Year	Number	Min	Max	Rank	Rate	Min	Max	Rank
Minneapolis--St. Paul, MN--WI	Male	1992	6,682	3,869	11,761	43	75.99	44.00	133.74	93
		1993	5,492	2,449	11,165	46	61.29	27.33	124.60	96
		1994	6,275	3,562	10,430	44	68.81	39.06	114.37	94
		1995	5,195	2,714	9,642	46	55.87	29.19	103.70	96
		1996	5,029	2,844	8,845	49	53.09	30.02	93.37	98
		1997	4,837	2,971	7,984	54	50.25	30.86	82.93	99
		1998	4,596	2,798	6,917	56	47.03	28.63	70.79	99
		1999	4,741	2,611	5,844	57	47.60	26.22	58.67	99
		2000	4,403	2,591	5,968	57	43.34	25.50	58.74	99
		2001	4,716	2,564	6,163	55	45.59	24.79	59.58	99
		2002	4,636	2,528	6,342	49	44.22	24.11	60.50	99
		2003	4,869	2,489	6,900	49	45.90	23.47	65.05	98
		2004	5,228	2,452	8,097	47	48.72	22.85	75.46	98
		2005	5,609	2,422	9,344	47	51.66	22.30	86.06	98
		2006	6,047	2,402	10,750	43	55.02	21.86	97.80	98
		2007	6,518	2,390	12,244	40	58.61	21.49	110.10	98
	Female	1992	3,312	1,918	5,829	44	37.39	21.65	65.82	87
		1993	2,696	1,202	5,481	51	29.93	13.35	60.85	95
		1994	3,079	1,748	5,117	47	33.62	19.08	55.88	93
		1995	2,565	1,340	4,762	54	27.52	14.38	51.09	98
		1996	2,514	1,422	4,421	57	26.50	14.99	46.60	99
		1997	2,457	1,509	4,055	60	25.50	15.66	42.09	100
		1998	2,376	1,447	3,576	60	24.30	14.79	36.57	100
		1999	2,496	1,375	3,077	62	25.09	13.82	30.92	100
		2000	2,359	1,388	3,197	63	23.26	13.69	31.53	100
		2001	2,562	1,393	3,349	61	24.87	13.52	32.51	100
		2002	2,544	1,387	3,480	59	24.41	13.31	33.40	100
		2003	2,681	1,370	3,799	56	25.50	13.04	36.14	100
		2004	2,865	1,344	4,437	52	26.97	12.65	41.78	100
		2005	3,030	1,308	5,048	49	28.24	12.19	47.05	98
		2006	3,183	1,265	5,659	46	29.33	11.65	52.14	98
		2007	3,299	1,210	6,197	45	30.08	11.03	56.51	97

Metropolitan Statistical Area	PWID Population	Year	Number	Min	Max	Rank	Rate	Min	Max	Rank
Minneapolis--St. Paul, MN--WI	Young (15-29)	1992	3,824	2,214	6,731	34	65.30	37.82	114.94	67
		1993	3,047	1,358	6,194	35	52.44	23.38	106.62	72
		1994	3,417	1,940	5,679	33	59.08	33.53	98.19	71
		1995	2,807	1,466	5,210	37	48.23	25.19	89.52	76
		1996	2,721	1,539	4,786	37	46.24	26.15	81.33	79
		1997	2,642	1,622	4,360	37	44.31	27.21	73.13	80
		1998	2,549	1,551	3,836	39	42.12	25.64	63.40	83
		1999	2,681	1,477	3,305	44	43.65	24.04	53.81	88
		2000	2,546	1,498	3,451	46	40.90	24.07	55.44	87
		2001	2,792	1,518	3,649	46	44.84	24.38	58.60	93
		2002	2,807	1,531	3,841	45	44.92	24.49	61.45	93
		2003	3,009	1,538	4,264	44	47.82	24.45	67.78	93
		2004	3,283	1,540	5,084	39	51.81	24.30	80.24	91
		2005	3,559	1,536	5,929	37	55.81	24.10	92.98	93
		2006	3,849	1,529	6,842	37	59.84	23.77	106.37	88
		2007	4,126	1,513	7,750	33	63.71	23.36	119.68	84
	Old (30-64)	1992	6,431	3,724	11,319	52	54.53	31.58	95.97	95
		1993	5,233	2,333	10,638	59	43.04	19.19	87.50	98
		1994	5,956	3,381	9,900	55	47.68	27.06	79.24	97
		1995	4,935	2,578	9,160	62	38.55	20.14	71.56	100
		1996	4,796	2,712	8,435	63	36.68	20.74	64.51	100
		1997	4,638	2,848	7,655	64	34.87	21.42	57.56	100
		1998	4,430	2,697	6,667	64	32.82	19.98	49.39	100
		1999	4,586	2,526	5,653	68	33.31	18.34	41.06	98
		2000	4,261	2,507	5,776	65	30.28	17.82	41.04	100
		2001	4,544	2,471	5,939	65	31.51	17.13	41.19	98
		2002	4,419	2,409	6,045	63	30.16	16.44	41.25	99
		2003	4,551	2,327	6,450	58	30.70	15.69	43.50	98
		2004	4,740	2,224	7,342	55	31.57	14.81	48.90	98
		2005	4,865	2,101	8,106	53	31.99	13.81	53.29	98
		2006	4,932	1,959	8,768	52	32.00	12.71	56.89	98
		2007	4,893	1,794	9,192	51	31.34	11.49	58.88	97

Metropolitan Statistical Area	PWID Population	Year	Number	Min	Max	Rank	Rate	Min	Max	Rank
Monmouth--Ocean, NJ	Total	1992	6,684	5,565	8,478	63	106.59	88.75	135.20	49
		1993	5,683	3,522	7,875	69	89.76	55.62	124.37	55
		1994	6,132	5,378	7,267	68	95.89	84.11	113.64	53
		1995	5,554	4,522	6,730	70	85.57	69.66	103.68	58
		1996	5,502	4,702	6,227	72	83.58	71.42	94.59	61
		1997	5,489	4,377	6,170	71	81.96	65.35	92.12	63
		1998	5,524	4,114	6,355	70	81.15	60.45	93.36	61
		1999	5,124	3,858	6,544	80	74.07	55.77	94.60	67
		2000	5,638	3,631	7,594	68	80.12	51.60	107.91	63
		2001	4,913	3,553	6,899	82	68.52	49.56	96.22	74
		2002	5,708	3,752	8,139	73	77.94	51.23	111.12	65
		2003	5,709	3,704	7,894	73	76.71	49.77	106.07	64
		2004	5,806	3,423	7,549	72	77.01	45.41	100.14	63
		2005	6,048	3,157	7,145	71	79.38	41.43	93.78	59
		2006	6,282	2,897	8,756	69	81.85	37.75	114.09	59
		2007	6,528	2,633	10,595	65	84.56	34.10	137.24	56
	Non-Hispanic White	1992	3,918	3,262	4,970	59	71.81	59.79	91.09	52
		1993	3,374	2,091	4,675	62	61.49	38.10	85.20	56
		1994	3,696	3,242	4,381	61	66.96	58.74	79.36	55
		1995	3,407	2,773	4,128	63	61.03	49.68	73.94	58
		1996	3,438	2,938	3,891	63	60.98	52.12	69.02	59
		1997	3,498	2,789	3,931	61	61.22	48.81	68.80	59
		1998	3,590	2,674	4,130	58	62.05	46.21	71.38	59
		1999	3,396	2,557	4,337	68	57.95	43.63	74.02	65
		2000	3,808	2,453	5,130	54	64.13	41.30	86.38	60
		2001	3,380	2,444	4,746	67	56.12	40.59	78.81	71
		2002	3,993	2,625	5,694	52	65.21	42.86	92.97	60
		2003	4,055	2,631	5,607	51	65.43	42.45	90.48	58
		2004	4,180	2,465	5,435	52	66.78	39.38	86.83	58
		2005	4,404	2,299	5,203	51	69.83	36.45	82.50	54
		2006	4,617	2,129	6,435	49	72.89	33.62	101.60	54
		2007	4,830	1,948	7,839	48	76.11	30.69	123.53	52

Metropolitan Statistical Area	PWID Population	Year	Number	Min	Max	Rank	Rate	Min	Max	Rank
Monmouth--Ocean, NJ	Non-Hispanic Black	1992	1,976	1,646	2,507	55	515.19	428.97	653.49	16
		1993	1,626	1,007	2,253	58	421.93	261.45	584.66	18
		1994	1,679	1,472	1,989	60	431.37	378.36	511.20	17
		1995	1,442	1,173	1,747	60	365.99	297.94	443.44	20
		1996	1,343	1,148	1,520	63	336.94	287.95	381.36	21
		1997	1,253	999	1,409	63	310.17	247.31	348.61	22
		1998	1,175	875	1,352	63	287.01	213.78	330.20	23
		1999	1,014	763	1,295	66	245.23	184.65	313.21	31
		2000	1,039	669	1,400	65	248.48	160.03	334.70	25
		2001	847	613	1,189	67	198.40	143.48	278.58	41
		2002	927	609	1,321	66	213.98	140.64	305.10	29
		2003	882	572	1,219	66	201.53	130.76	278.67	32
		2004	865	510	1,124	68	195.50	115.27	254.20	33
		2005	884	461	1,044	68	198.44	103.58	234.45	31
		2006	919	424	1,281	67	205.86	94.94	286.94	31
		2007	981	396	1,592	67	218.54	88.13	354.70	29
	Hispanic	1992	526	438	668	53	192.07	159.93	243.63	34
		1993	371	230	513	58	126.97	78.68	175.94	44
		1994	350	307	415	58	113.49	99.55	134.50	49
		1995	292	238	354	60	89.06	72.50	107.91	53
		1996	279	238	315	60	79.70	68.11	90.21	57
		1997	277	221	312	61	74.59	59.48	83.84	55
		1998	286	213	329	61	72.68	54.14	83.62	56
		1999	278	209	355	63	67.01	50.46	85.59	62
		2000	324	208	436	62	74.03	47.68	99.72	55
		2001	300	217	421	63	64.60	46.72	90.71	61
		2002	370	243	528	61	75.03	49.31	106.97	52
		2003	389	252	538	61	74.72	48.48	103.32	51
		2004	409	241	532	60	75.40	44.46	98.05	51
		2005	430	225	508	61	76.40	39.88	90.27	49
		2006	438	202	611	61	75.14	34.66	104.74	49
		2007	431	174	699	61	71.18	28.70	115.52	55

Metropolitan Statistical Area	PWID Population	Year	Number	Min	Max	Rank	Rate	Min	Max	Rank
Monmouth--Ocean, NJ	Male	1992	4,218	3,512	5,351	67	136.92	114.01	173.68	54
		1993	3,548	2,199	4,917	72	114.08	70.69	158.07	59
		1994	3,808	3,340	4,512	73	121.20	106.31	143.63	56
		1995	3,445	2,805	4,174	74	107.99	87.91	130.85	62
		1996	3,421	2,924	3,872	75	105.74	90.37	119.68	62
		1997	3,430	2,735	3,855	74	104.17	83.06	117.08	61
		1998	3,473	2,587	3,995	72	103.73	77.26	119.34	60
		1999	3,243	2,442	4,142	81	95.35	71.79	121.78	67
		2000	3,589	2,311	4,834	69	103.75	66.81	139.74	60
		2001	3,141	2,272	4,411	82	89.01	64.38	124.99	69
		2002	3,656	2,403	5,213	69	101.30	66.58	144.43	60
		2003	3,650	2,368	5,047	69	99.39	64.49	137.44	61
		2004	3,690	2,175	4,797	68	99.02	58.39	128.75	60
		2005	3,798	1,982	4,487	66	100.69	52.56	118.96	60
		2006	3,872	1,786	5,397	66	101.82	46.96	141.93	57
		2007	3,914	1,579	6,353	68	102.18	41.21	165.84	57
	Female	1992	2,523	2,101	3,201	61	79.10	65.86	100.34	43
		1993	2,164	1,341	2,998	64	67.18	41.63	93.08	49
		1994	2,347	2,059	2,782	64	72.16	63.30	85.52	48
		1995	2,132	1,736	2,584	67	64.61	52.60	78.29	56
		1996	2,115	1,807	2,393	67	63.16	53.98	71.49	57
		1997	2,109	1,682	2,371	68	61.95	49.39	69.63	57
		1998	2,120	1,579	2,439	68	61.31	45.67	70.53	57
		1999	1,965	1,479	2,509	74	55.87	42.07	71.36	66
		2000	2,160	1,391	2,910	68	60.39	38.89	81.35	60
		2001	1,884	1,362	2,645	78	51.73	37.41	72.64	72
		2002	2,193	1,441	3,127	67	59.03	38.80	84.17	61
		2003	2,202	1,429	3,045	66	58.43	37.91	80.79	61
		2004	2,255	1,330	2,933	65	59.16	34.88	76.93	59
		2005	2,374	1,239	2,805	66	61.71	32.21	72.91	55
		2006	2,501	1,154	3,486	64	64.59	29.79	90.03	54
		2007	2,648	1,068	4,298	56	68.10	27.46	110.53	45

Metropolitan Statistical Area	PWID Population	Year	Number	Min	Max	Rank	Rate	Min	Max	Rank
Monmouth--Ocean, NJ	Young (15-29)	1992	1,682	1,400	2,133	66	91.69	76.35	116.31	49
		1993	1,592	987	2,206	63	88.54	54.86	122.68	47
		1994	1,904	1,670	2,256	55	107.71	94.48	127.64	33
		1995	1,899	1,546	2,301	53	108.27	88.13	131.18	34
		1996	2,056	1,757	2,327	51	118.03	100.87	133.59	31
		1997	2,223	1,772	2,498	48	127.61	101.75	143.42	25
		1998	2,402	1,789	2,764	45	137.11	102.13	157.74	18
		1999	2,370	1,785	3,027	51	134.42	101.21	171.68	26
		2000	2,747	1,769	3,700	43	154.29	99.36	207.82	17
		2001	2,496	1,805	3,505	51	136.92	99.02	192.25	27
		2002	2,994	1,968	4,269	43	158.82	104.39	226.44	18
		2003	3,060	1,985	4,231	41	157.31	102.07	217.53	19
		2004	3,146	1,855	4,091	41	157.14	92.66	204.33	19
		2005	3,277	1,710	3,871	40	159.32	83.15	188.22	18
		2006	3,361	1,550	4,685	39	160.20	73.89	223.31	20
		2007	3,403	1,373	5,524	41	159.43	64.29	258.75	20
	Old (30-64)	1992	5,125	4,267	6,501	61	115.51	96.18	146.52	51
		1993	4,151	2,572	5,752	68	91.57	56.74	126.89	65
		1994	4,264	3,740	5,054	71	92.17	80.84	109.23	69
		1995	3,680	2,996	4,459	78	77.69	63.25	94.13	74
		1996	3,478	2,973	3,937	79	71.84	61.40	81.32	77
		1997	3,318	2,646	3,730	79	66.97	53.40	75.27	78
		1998	3,202	2,385	3,683	79	63.34	47.18	72.87	80
		1999	2,857	2,151	3,648	86	55.42	41.73	70.78	87
		2000	3,033	1,953	4,086	80	57.70	37.16	77.72	84
		2001	2,560	1,851	3,595	86	47.88	34.62	67.22	93
		2002	2,890	1,899	4,120	82	53.13	34.92	75.75	84
		2003	2,816	1,827	3,894	82	51.24	33.24	70.85	86
		2004	2,799	1,650	3,639	81	50.55	29.81	65.73	84
		2005	2,854	1,490	3,372	81	51.31	26.78	60.62	80
		2006	2,906	1,340	4,051	81	52.11	24.04	72.64	77
		2007	2,964	1,195	4,811	78	53.07	21.40	86.13	73

Metropolitan Statistical Area	PWID Population	Year	Number	Min	Max	Rank	Rate	Min	Max	Rank
Nashville, TN	Total	1992	7,215	5,414	9,671	60	102.61	76.99	137.53	51
		1993	6,112	2,433	10,255	65	84.69	33.71	142.09	60
		1994	7,484	4,542	10,865	59	100.70	61.12	146.18	49
		1995	6,518	3,187	11,498	64	85.20	41.66	150.28	60
		1996	6,738	3,601	12,151	63	85.64	45.76	154.43	60
		1997	6,953	3,139	12,817	61	86.03	38.83	158.58	57
		1998	7,260	3,107	13,439	54	87.95	37.63	162.79	57
		1999	8,460	3,061	14,033	49	100.67	36.43	167.00	52
		2000	7,767	3,331	14,194	49	90.72	38.90	165.80	54
		2001	8,910	3,613	14,372	47	102.04	41.38	164.59	50
		2002	8,229	4,058	14,370	47	92.79	45.76	162.05	52
		2003	8,468	4,521	14,384	47	93.90	50.14	159.51	51
		2004	8,766	5,009	14,510	47	95.10	54.35	157.42	47
		2005	9,082	5,530	14,668	46	96.18	58.56	155.34	47
		2006	9,394	6,013	14,819	44	97.07	62.13	153.13	44
		2007	9,692	6,506	14,946	44	97.87	65.70	150.93	42
	Non-Hispanic White	1992	4,044	3,035	5,421	56	69.56	52.19	93.24	53
		1993	3,452	1,374	5,791	61	58.14	23.14	97.55	61
		1994	4,275	2,595	6,206	54	70.24	42.63	101.97	52
		1995	3,778	1,847	6,664	56	60.59	29.63	106.87	59
		1996	3,971	2,122	7,161	51	62.29	33.28	112.32	57
		1997	4,172	1,883	7,691	47	64.08	28.92	118.11	58
		1998	4,439	1,900	8,217	45	67.14	28.73	124.28	57
		1999	5,271	1,907	8,743	38	78.81	28.52	130.73	49
		2000	4,928	2,113	9,006	38	72.85	31.24	133.14	55
		2001	5,751	2,332	9,276	37	83.79	33.98	135.14	43
		2002	5,393	2,660	9,419	38	77.75	38.34	135.78	48
		2003	5,625	3,003	9,555	38	80.11	42.77	136.08	44
		2004	5,887	3,364	9,744	35	82.48	47.14	136.52	44
		2005	6,148	3,743	9,930	34	84.55	51.48	136.56	42
		2006	6,390	4,090	10,080	32	86.14	55.14	135.90	43
		2007	6,600	4,430	10,177	32	87.34	58.63	134.68	42

Metropolitan Statistical Area	PWID Population	Year	Number	Min	Max	Rank	Rate	Min	Max	Rank
Nashville, TN	Non-Hispanic Black	1992	2,984	2,239	3,999	38	285.41	214.16	382.56	53
		1993	2,416	962	4,054	41	222.70	88.64	373.63	56
		1994	2,819	1,711	4,093	36	251.44	152.61	365.02	57
		1995	2,333	1,141	4,115	39	201.57	98.56	355.55	59
		1996	2,285	1,221	4,120	38	191.10	102.12	344.59	61
		1997	2,228	1,006	4,108	37	180.58	81.51	332.87	61
		1998	2,195	939	4,063	37	174.09	74.49	322.25	58
		1999	2,409	872	3,997	37	187.81	67.96	311.55	49
		2000	2,083	893	3,806	37	159.01	68.19	290.60	58
		2001	2,250	912	3,629	38	166.96	67.70	269.31	58
		2002	1,958	966	3,420	38	142.05	70.05	248.07	58
		2003	1,904	1,017	3,234	36	134.89	72.02	229.14	61
		2004	1,869	1,068	3,093	35	128.33	73.34	212.43	61
		2005	1,845	1,123	2,980	35	122.51	74.59	197.86	62
		2006	1,832	1,172	2,890	37	117.93	75.49	186.04	64
		2007	1,832	1,230	2,825	41	114.94	77.16	177.25	65
	Hispanic	1992	68	51	91	82	95.73	71.83	128.31	62
		1993	58	23	97	83	69.75	27.76	117.02	66
		1994	73	44	105	82	72.42	43.95	105.13	71
		1995	65	32	115	83	53.74	26.28	94.79	75
		1996	71	38	127	82	47.76	25.52	86.12	77
		1997	77	35	141	81	43.28	19.53	79.77	78
		1998	84	36	156	81	39.33	16.83	72.81	77
		1999	104	37	172	78	41.00	14.84	68.02	77
		2000	100	43	183	78	33.87	14.52	61.90	79
		2001	121	49	195	77	37.47	15.19	60.44	79
		2002	116	57	203	77	33.86	16.70	59.13	77
		2003	124	66	211	78	33.92	18.11	57.62	76
		2004	132	76	219	78	33.50	19.14	55.45	76
		2005	139	85	225	77	32.87	20.01	53.09	78
		2006	145	93	229	77	31.97	20.46	50.43	78
		2007	149	100	229	79	30.58	20.53	47.17	80

Metropolitan Statistical Area	PWID Population	Year	Number	Min	Max	Rank	Rate	Min	Max	Rank
Nashville, TN	Male	1992	4,792	3,596	6,423	58	139.38	104.58	186.81	51
		1993	4,073	1,621	6,833	66	115.23	45.87	193.33	58
		1994	5,001	3,035	7,260	58	137.23	83.29	199.22	48
		1995	4,365	2,135	7,700	62	116.19	56.81	204.94	58
		1996	4,519	2,415	8,150	58	116.76	62.40	210.55	55
		1997	4,666	2,106	8,601	56	117.29	52.94	216.20	51
		1998	4,870	2,084	9,014	52	119.57	51.16	221.34	51
		1999	5,664	2,049	9,395	46	136.27	49.31	226.05	44
		2000	5,183	2,222	9,471	48	122.11	52.36	223.16	48
		2001	5,916	2,399	9,542	45	136.58	55.38	220.29	43
		2002	5,425	2,675	9,474	45	123.28	60.79	215.30	45
		2003	5,531	2,953	9,395	45	123.72	66.06	210.16	43
		2004	5,658	3,234	9,366	45	123.60	70.64	204.60	43
		2005	5,776	3,517	9,329	46	123.11	74.96	198.83	44
		2006	5,867	3,756	9,256	44	122.20	78.22	192.79	45
		2007	5,922	3,975	9,132	44	120.30	80.76	185.51	44
	Female	1992	2,525	1,895	3,384	60	70.27	52.73	94.19	49
		1993	2,095	834	3,514	65	56.88	22.64	95.43	63
		1994	2,546	1,545	3,696	61	67.21	40.79	97.57	57
		1995	2,227	1,089	3,928	64	57.19	27.97	100.88	61
		1996	2,334	1,247	4,208	61	58.38	31.20	105.27	61
		1997	2,458	1,110	4,531	59	59.90	27.04	110.42	58
		1998	2,632	1,126	4,872	56	62.93	26.93	116.48	56
		1999	3,152	1,141	5,229	49	74.23	26.86	123.14	48
		2000	2,975	1,276	5,437	47	68.92	29.55	125.95	54
		2001	3,502	1,420	5,649	44	79.58	32.27	128.35	41
		2002	3,305	1,630	5,772	44	73.98	36.48	129.19	43
		2003	3,456	1,846	5,871	43	76.01	40.58	129.12	39
		2004	3,609	2,063	5,974	42	77.79	44.46	128.77	38
		2005	3,737	2,275	6,035	41	78.67	47.90	127.05	35
		2006	3,820	2,445	6,026	40	78.33	50.14	123.58	35
		2007	3,842	2,579	5,925	38	77.15	51.79	118.97	35

Metropolitan Statistical Area	PWID Population	Year	Number	Min	Max	Rank	Rate	Min	Max	Rank
Nashville, TN	Young (15-29)	1992	2,399	1,800	3,215	48	99.51	74.67	133.38	41
		1993	1,773	706	2,975	55	72.71	28.94	121.99	56
		1994	1,955	1,186	2,838	53	79.00	47.95	114.68	56
		1995	1,582	774	2,791	65	62.76	30.69	110.70	58
		1996	1,568	838	2,828	65	60.87	32.53	109.76	61
		1997	1,596	720	2,941	66	60.56	27.34	111.63	64
		1998	1,684	721	3,117	65	62.82	26.88	116.29	63
		1999	2,024	732	3,358	60	74.74	27.05	123.99	62
		2000	1,948	835	3,560	59	71.24	30.55	130.20	64
		2001	2,370	961	3,823	54	86.38	35.03	139.32	60
		2002	2,338	1,153	4,082	52	85.54	42.18	149.39	57
		2003	2,576	1,375	4,375	49	93.72	50.04	159.20	53
		2004	2,851	1,629	4,719	45	101.79	58.17	168.50	51
		2005	3,143	1,914	5,076	41	110.15	67.07	177.90	41
		2006	3,430	2,195	5,411	38	118.03	75.55	186.20	33
		2007	3,693	2,479	5,695	37	124.33	83.47	191.73	32
	Old (30-64)	1992	4,853	3,641	6,505	63	105.02	78.80	140.76	59
		1993	4,359	1,735	7,314	66	91.23	36.32	153.07	66
		1994	5,562	3,376	8,075	60	112.18	68.09	162.86	49
		1995	4,984	2,437	8,791	61	97.16	47.51	171.37	59
		1996	5,247	2,804	9,462	56	99.15	52.99	178.80	57
		1997	5,470	2,469	10,082	55	100.41	45.32	185.09	54
		1998	5,729	2,451	10,604	48	102.75	43.97	190.20	51
		1999	6,651	2,407	11,033	46	116.79	42.26	193.73	40
		2000	6,045	2,592	11,047	44	103.74	44.49	189.59	45
		2001	6,819	2,765	10,999	42	113.88	46.18	183.68	40
		2002	6,148	3,032	10,736	42	100.20	49.41	174.99	45
		2003	6,127	3,271	10,408	42	97.73	52.18	166.01	46
		2004	6,087	3,479	10,076	44	94.87	54.22	157.04	45
		2005	5,988	3,646	9,672	45	90.88	55.34	146.78	45
		2006	5,809	3,718	9,164	45	85.79	54.91	135.34	46
		2007	5,541	3,720	8,545	46	79.93	53.66	123.26	48

Metropolitan Statistical Area	PWID Population	Year	Number	Min	Max	Rank	Rate	Min	Max	Rank
Nassau--Suffolk, NY	Total	1992	9,457	5,813	13,604	47	53.61	32.95	77.12	94
		1993	9,604	5,835	13,583	43	54.54	33.13	77.14	89
		1994	9,501	5,837	13,549	43	54.10	33.24	77.15	93
		1995	10,280	5,848	13,535	41	58.61	33.34	77.17	83
		1996	10,629	5,850	13,835	39	60.57	33.34	78.85	81
		1997	11,006	5,870	15,163	36	62.51	33.34	86.12	78
		1998	11,439	5,905	16,595	34	64.45	33.27	93.51	75
		1999	9,809	5,933	13,800	43	54.89	33.20	77.22	89
		2000	12,107	5,935	18,890	33	67.25	32.96	104.92	69
		2001	9,941	5,971	14,096	44	54.49	32.72	77.25	90
		2002	12,505	5,973	19,997	32	67.75	32.36	108.34	69
		2003	12,424	5,971	19,489	32	66.57	31.99	104.43	71
		2004	12,217	5,948	18,543	34	64.84	31.57	98.42	74
		2005	11,930	5,907	17,370	38	62.91	31.15	91.59	74
		2006	11,605	5,868	16,092	36	60.88	30.78	84.42	76
		2007	11,255	5,826	14,815	38	58.76	30.42	77.34	80
	Non-Hispanic White	1992	6,187	3,803	8,900	37	42.81	26.31	61.58	84
		1993	6,350	3,858	8,981	32	44.45	27.01	62.87	76
		1994	6,308	3,875	8,996	34	44.72	27.47	63.77	81
		1995	6,821	3,880	8,980	29	48.89	27.81	64.37	68
		1996	7,022	3,865	9,140	28	50.80	27.96	66.13	68
		1997	7,223	3,852	9,951	27	52.58	28.04	72.44	66
		1998	7,447	3,844	10,805	26	54.28	28.02	78.76	65
		1999	6,335	3,832	8,913	33	46.29	27.99	65.12	75
		2000	7,766	3,807	12,117	26	56.85	27.86	88.70	67
		2001	6,348	3,813	9,001	33	46.35	27.84	65.72	78
		2002	7,978	3,810	12,758	25	58.15	27.78	93.00	65
		2003	7,957	3,824	12,482	25	57.88	27.81	90.79	65
		2004	7,901	3,847	11,992	26	57.32	27.91	87.01	67
		2005	7,841	3,882	11,416	27	56.87	28.16	82.80	68
		2006	7,804	3,946	10,821	26	56.66	28.65	78.56	68
		2007	7,795	4,035	10,260	28	56.68	29.34	74.60	72

Metropolitan Statistical Area	PWID Population	Year	Number	Min	Max	Rank	Rate	Min	Max	Rank
Nassau--Suffolk, NY	Non-Hispanic Black	1992	1,883	1,158	2,709	57	141.97	87.26	204.22	91
		1993	1,916	1,164	2,710	52	141.45	85.94	200.07	87
		1994	1,873	1,151	2,672	57	135.56	83.28	193.33	89
		1995	1,979	1,126	2,606	47	140.33	79.82	184.76	82
		1996	1,978	1,089	2,574	46	137.70	75.79	179.25	80
		1997	1,962	1,046	2,703	45	133.62	71.26	184.09	78
		1998	1,939	1,001	2,814	43	128.90	66.54	187.02	77
		1999	1,574	952	2,214	56	102.38	61.92	144.03	85
		2000	1,832	898	2,858	44	117.01	57.36	182.57	76
		2001	1,417	851	2,009	59	87.55	52.58	124.13	88
		2002	1,680	802	2,686	46	101.09	48.28	161.67	78
		2003	1,578	758	2,475	49	93.05	44.72	145.97	81
		2004	1,475	718	2,239	54	85.57	41.66	129.88	84
		2005	1,382	684	2,013	56	79.44	39.33	115.66	84
		2006	1,306	660	1,811	58	74.21	37.52	102.91	84
		2007	1,251	648	1,647	60	70.29	36.39	92.52	86
	Hispanic	1992	696	428	1,002	46	52.51	32.27	75.53	86
		1993	715	435	1,012	47	51.01	30.99	72.14	81
		1994	728	447	1,039	46	49.36	30.33	70.40	83
		1995	821	467	1,081	44	52.99	30.14	69.77	76
		1996	893	491	1,162	42	54.85	30.19	71.40	72
		1997	976	521	1,345	42	57.11	30.46	78.68	69
		1998	1,072	553	1,555	42	59.65	30.79	86.54	67
		1999	968	586	1,362	44	51.41	31.09	72.32	72
		2000	1,250	613	1,950	41	63.21	30.98	98.63	61
		2001	1,062	638	1,506	44	51.17	30.73	72.55	66
		2002	1,364	652	2,181	40	62.89	30.04	100.56	60
		2003	1,361	654	2,134	41	60.19	28.92	94.41	60
		2004	1,317	641	1,998	41	56.51	27.51	85.77	62
		2005	1,236	612	1,800	44	51.78	25.63	75.38	63
		2006	1,126	569	1,561	49	46.06	23.29	63.87	66
		2007	992	514	1,306	51	39.60	20.50	52.13	69

Metropolitan Statistical Area	PWID Population	Year	Number	Min	Max	Rank	Rate	Min	Max	Rank
Nassau--Suffolk, NY	Male	1992	6,044	3,715	8,694	46	69.63	42.80	100.17	97
		1993	6,313	3,835	8,929	41	72.85	44.26	103.04	89
		1994	6,388	3,924	9,110	41	73.93	45.42	105.43	92
		1995	7,036	4,002	9,264	39	81.53	46.38	107.35	82
		1996	7,376	4,060	9,602	35	85.48	47.05	111.27	77
		1997	7,719	4,117	10,634	34	89.14	47.54	122.82	75
		1998	8,084	4,173	11,728	33	92.61	47.81	134.36	67
		1999	6,969	4,215	9,804	36	79.30	47.96	111.56	80
		2000	8,629	4,230	13,463	32	97.45	47.77	152.05	65
		2001	7,096	4,262	10,061	36	78.96	47.43	111.96	78
		2002	8,926	4,263	14,275	29	98.06	46.83	156.81	64
		2003	8,859	4,258	13,897	31	96.09	46.18	150.73	64
		2004	8,695	4,233	13,198	31	93.33	45.44	141.66	64
		2005	8,470	4,194	12,332	31	90.17	44.64	131.28	65
		2006	8,216	4,154	11,393	33	86.92	43.95	120.52	66
		2007	7,947	4,114	10,460	37	83.53	43.24	109.95	69
	Female	1992	2,797	1,719	4,023	56	31.21	19.18	44.90	94
		1993	2,862	1,739	4,048	46	32.00	19.44	45.26	89
		1994	2,848	1,749	4,061	54	31.92	19.61	45.52	95
		1995	3,093	1,759	4,072	45	34.71	19.74	45.70	88
		1996	3,205	1,764	4,172	44	35.94	19.78	46.78	88
		1997	3,320	1,771	4,575	41	37.11	19.79	51.13	89
		1998	3,448	1,780	5,003	40	38.23	19.74	55.47	89
		1999	2,951	1,785	4,152	54	32.50	19.65	45.72	93
		2000	3,632	1,780	5,667	40	39.69	19.46	61.93	88
		2001	2,971	1,784	4,212	54	32.08	19.27	45.49	96
		2002	3,719	1,776	5,948	42	39.76	18.99	63.58	88
		2003	3,676	1,767	5,766	42	38.93	18.71	61.07	88
		2004	3,594	1,750	5,456	43	37.74	18.37	57.28	89
		2005	3,490	1,728	5,081	43	36.47	18.05	53.09	93
		2006	3,375	1,707	4,680	44	35.12	17.76	48.71	93
		2007	3,256	1,685	4,286	46	33.77	17.48	44.45	93

Metropolitan Statistical Area	PWID Population	Year	Number	Min	Max	Rank	Rate	Min	Max	Rank
Nassau--Suffolk, NY	Young (15-29)	1992	2,017	1,239	2,901	55	36.98	22.73	53.20	96
		1993	2,286	1,389	3,234	44	43.22	26.26	61.12	79
		1994	2,506	1,539	3,574	40	48.82	29.99	69.62	80
		1995	2,979	1,695	3,923	35	59.54	33.87	78.39	62
		1996	3,356	1,847	4,369	29	68.56	37.73	89.24	56
		1997	3,754	2,002	5,172	26	77.88	41.54	107.30	54
		1998	4,178	2,156	6,061	24	87.42	45.13	126.83	49
		1999	3,802	2,300	5,349	28	80.48	48.68	113.23	56
		2000	4,937	2,420	7,703	22	105.12	51.53	164.01	40
		2001	4,228	2,539	5,995	27	88.86	53.37	125.99	56
		2002	5,498	2,626	8,792	20	113.86	54.38	182.08	40
		2003	5,597	2,690	8,780	21	113.47	54.53	178.00	40
		2004	5,590	2,722	8,485	20	110.95	54.02	168.40	41
		2005	5,494	2,720	7,999	21	106.71	52.83	155.37	44
		2006	5,327	2,694	7,387	22	101.59	51.37	140.88	46
		2007	5,099	2,639	6,712	24	95.46	49.41	125.65	49
	Old (30-64)	1992	7,558	4,645	10,872	43	62.01	38.12	89.21	92
		1993	7,395	4,493	10,459	41	60.03	36.47	84.90	92
		1994	7,051	4,332	10,055	47	56.73	34.85	80.90	93
		1995	7,361	4,187	9,692	41	58.72	33.40	77.31	91
		1996	7,354	4,048	9,573	41	58.13	31.99	75.66	91
		1997	7,370	3,931	10,154	40	57.64	30.74	79.41	88
		1998	7,425	3,833	10,773	38	57.25	29.55	83.06	88
		1999	6,181	3,739	8,696	49	47.02	28.44	66.15	95
		2000	7,415	3,634	11,569	39	55.72	27.31	86.94	86
		2001	5,922	3,557	8,396	48	43.90	26.37	62.25	95
		2002	7,247	3,461	11,589	39	53.17	25.40	85.03	83
		2003	7,002	3,365	10,983	39	51.00	24.51	80.00	87
		2004	6,687	3,256	10,150	39	48.45	23.59	73.54	87
		2005	6,329	3,133	9,214	41	45.81	22.68	66.69	89
		2006	5,945	3,006	8,244	42	43.02	21.75	59.66	92
		2007	5,542	2,869	7,295	45	40.13	20.77	52.82	92

Metropolitan Statistical Area	PWID Population	Year	Number	Min	Max	Rank	Rate	Min	Max	Rank
New Haven--Meriden, CT	Total	1992	16,264	15,333	17,270	30	150.34	141.74	159.64	34
		1993	15,695	14,243	16,971	27	145.68	132.20	157.52	31
		1994	16,106	14,836	16,773	30	149.79	137.98	156.00	31
		1995	15,617	14,353	16,953	28	145.14	133.40	157.56	30
		1996	15,647	14,422	17,201	28	144.67	133.35	159.05	30
		1997	15,498	14,443	16,719	27	142.27	132.59	153.49	28
		1998	15,363	14,442	16,121	27	140.24	131.84	147.17	27
		1999	15,396	14,572	15,995	29	139.74	132.27	145.18	26
		2000	14,855	13,499	16,352	28	133.82	121.60	147.31	27
		2001	15,119	12,467	17,091	29	135.19	111.47	152.82	28
		2002	14,302	11,194	18,080	27	126.85	99.28	160.36	29
		2003	14,039	10,018	19,059	28	123.66	88.24	167.89	29
		2004	13,838	9,289	19,948	29	121.30	81.42	174.85	28
		2005	13,651	8,551	20,860	30	118.98	74.53	181.81	28
		2006	13,447	7,848	21,713	32	116.70	68.11	188.43	30
		2007	13,269	7,203	22,570	32	114.66	62.25	195.05	29
	Non-Hispanic White	1992	7,188	6,777	7,633	32	83.50	78.73	88.67	46
		1993	6,973	6,327	7,539	28	82.11	74.51	88.78	45
		1994	7,184	6,617	7,481	29	85.42	78.69	88.96	44
		1995	6,986	6,420	7,583	27	83.72	76.95	90.89	49
		1996	7,012	6,463	7,708	29	84.34	77.74	92.73	47
		1997	6,951	6,478	7,499	28	83.77	78.07	90.37	46
		1998	6,890	6,477	7,230	28	83.27	78.28	87.38	47
		1999	6,898	6,529	7,166	29	83.66	79.19	86.92	45
		2000	6,644	6,037	7,313	28	80.68	73.31	88.81	46
		2001	6,745	5,561	7,624	32	81.95	67.57	92.64	48
		2002	6,360	4,977	8,039	32	77.30	60.50	97.72	49
		2003	6,218	4,437	8,442	33	75.67	53.99	102.73	52
		2004	6,103	4,097	8,797	34	74.40	49.94	107.25	49
		2005	5,991	3,753	9,155	35	73.22	45.86	111.88	53
		2006	5,871	3,427	9,480	37	72.06	42.06	116.36	55
		2007	5,762	3,128	9,802	38	71.02	38.56	120.81	57

Metropolitan Statistical Area	PWID Population	Year	Number	Min	Max	Rank	Rate	Min	Max	Rank
New Haven--Meriden, CT	Non-Hispanic Black	1992	4,701	4,432	4,992	22	439.44	414.31	466.63	25
		1993	4,353	3,950	4,707	24	401.56	364.40	434.19	25
		1994	4,234	3,901	4,410	25	387.66	357.10	403.72	24
		1995	3,851	3,540	4,181	25	348.21	320.03	378.00	24
		1996	3,589	3,308	3,945	25	320.02	294.97	351.82	24
		1997	3,284	3,061	3,543	26	287.66	268.09	310.33	26
		1998	2,996	2,817	3,144	27	257.24	241.82	269.94	31
		1999	2,760	2,613	2,868	28	233.41	220.92	242.48	37
		2000	2,453	2,229	2,701	31	204.65	185.96	225.28	39
		2001	2,312	1,907	2,614	37	190.43	157.02	215.27	44
		2002	2,044	1,599	2,583	35	165.89	129.83	209.70	51
		2003	1,898	1,354	2,576	37	151.81	108.32	206.10	54
		2004	1,799	1,207	2,593	38	142.44	95.62	205.33	56
		2005	1,741	1,091	2,661	39	135.95	85.16	207.74	60
		2006	1,725	1,007	2,786	43	133.16	77.71	215.00	57
		2007	1,763	957	2,999	46	134.39	72.96	228.59	57
	Hispanic	1992	4,658	4,391	4,946	17	525.50	495.44	558.01	7
		1993	4,274	3,879	4,622	17	461.21	418.53	498.68	7
		1994	4,301	3,962	4,479	17	445.61	410.47	464.07	7
		1995	4,199	3,859	4,558	17	416.91	383.18	452.58	6
		1996	4,326	3,988	4,756	16	407.62	375.71	448.12	6
		1997	4,475	4,171	4,828	15	401.49	374.17	433.14	7
		1998	4,679	4,399	4,910	15	403.53	379.35	423.45	7
		1999	4,968	4,703	5,162	15	410.69	388.72	426.66	7
		2000	5,078	4,615	5,590	15	401.53	364.87	442.01	8
		2001	5,451	4,494	6,161	14	413.85	341.24	467.83	7
		2002	5,392	4,220	6,817	13	392.57	307.24	496.24	9
		2003	5,472	3,905	7,429	13	384.04	274.03	521.38	9
		2004	5,494	3,688	7,919	12	373.63	250.81	538.60	9
		2005	5,420	3,395	8,283	13	356.35	223.22	544.53	9
		2006	5,223	3,048	8,433	13	331.08	193.22	534.58	11
		2007	4,904	2,663	8,343	15	300.27	163.01	510.76	11

Metropolitan Statistical Area	PWID Population	Year	Number	Min	Max	Rank	Rate	Min	Max	Rank
New Haven--Meriden, CT	Male	1992	11,754	11,081	12,481	26	221.72	209.04	235.44	26
		1993	11,152	10,120	12,059	26	211.37	191.81	228.54	29
		1994	11,307	10,416	11,776	26	214.84	197.90	223.74	25
		1995	10,883	10,003	11,814	25	206.66	189.94	224.34	26
		1996	10,868	10,017	11,947	25	205.35	189.28	225.75	25
		1997	10,765	10,032	11,613	24	201.99	188.25	217.91	24
		1998	10,700	10,059	11,228	24	199.76	187.79	209.63	20
		1999	10,771	10,195	11,190	23	200.01	189.31	207.79	20
		2000	10,449	9,495	11,502	23	192.52	174.94	211.92	21
		2001	10,695	8,818	12,089	22	195.36	161.09	220.84	20
		2002	10,167	7,957	12,852	23	183.95	143.97	232.53	22
		2003	10,016	7,147	13,598	24	179.80	128.30	244.10	21
		2004	9,889	6,638	14,255	24	176.44	118.44	254.35	20
		2005	9,744	6,104	14,890	24	172.45	108.03	263.52	22
		2006	9,554	5,576	15,426	24	168.34	98.24	271.81	23
		2007	9,341	5,071	15,890	25	163.59	88.81	278.26	25
	Female	1992	4,719	4,449	5,011	33	85.54	80.65	90.83	40
		1993	4,686	4,253	5,067	31	85.25	77.36	92.18	36
		1994	4,902	4,516	5,105	34	89.31	82.27	93.01	35
		1995	4,806	4,417	5,218	28	87.49	80.41	94.97	35
		1996	4,836	4,458	5,317	29	87.56	80.71	96.26	34
		1997	4,784	4,458	5,161	29	85.98	80.13	92.76	36
		1998	4,715	4,432	4,948	31	84.22	79.18	88.38	39
		1999	4,682	4,432	4,864	36	83.14	78.69	86.37	43
		2000	4,467	4,059	4,918	33	78.74	71.55	86.68	43
		2001	4,491	3,703	5,077	38	78.67	64.86	88.92	43
		2002	4,197	3,285	5,306	36	73.03	57.16	92.31	45
		2003	4,075	2,908	5,533	39	70.49	50.30	95.70	47
		2004	3,984	2,674	5,743	40	68.64	46.08	98.95	49
		2005	3,913	2,451	5,979	40	67.19	42.09	102.68	51
		2006	3,857	2,251	6,228	39	65.96	38.49	106.50	49
		2007	3,834	2,081	6,521	39	65.40	35.51	111.25	48

Metropolitan Statistical Area	PWID Population	Year	Number	Min	Max	Rank	Rate	Min	Max	Rank
New Haven--Meriden, CT	Young (15-29)	1992	4,164	3,925	4,421	31	122.79	115.77	130.39	28
		1993	3,815	3,462	4,125	28	116.17	105.42	125.61	28
		1994	3,802	3,502	3,960	28	119.14	109.75	124.08	28
		1995	3,655	3,359	3,968	27	117.15	107.67	127.18	28
		1996	3,697	3,407	4,064	27	120.32	110.90	132.27	27
		1997	3,753	3,498	4,049	27	123.08	114.71	132.79	27
		1998	3,862	3,630	4,052	27	127.24	119.61	133.52	28
		1999	4,055	3,838	4,213	26	134.49	127.29	139.72	25
		2000	4,126	3,749	4,541	26	137.00	124.49	150.81	23
		2001	4,443	3,664	5,023	26	147.47	121.60	166.71	23
		2002	4,451	3,484	5,627	27	146.20	114.42	184.81	21
		2003	4,619	3,296	6,270	26	150.14	107.13	203.83	21
		2004	4,792	3,217	6,908	25	153.55	103.08	221.35	21
		2005	4,945	3,097	7,556	24	155.30	97.28	237.31	20
		2006	5,053	2,949	8,159	25	156.99	91.62	253.49	21
		2007	5,121	2,780	8,711	23	157.03	85.25	267.11	21
	Old (30-64)	1992	12,263	11,561	13,021	29	165.12	155.67	175.33	35
		1993	11,911	10,808	12,878	27	159.03	144.31	171.95	32
		1994	12,262	11,296	12,770	26	162.18	149.39	168.90	35
		1995	11,894	10,932	12,912	27	155.68	143.09	169.00	28
		1996	11,886	10,956	13,067	26	153.51	141.49	168.76	28
		1997	11,705	10,909	12,628	26	149.24	139.08	161.00	29
		1998	11,497	10,808	12,065	26	145.18	136.48	152.35	28
		1999	11,374	10,766	11,817	27	142.14	134.54	147.67	32
		2000	10,790	9,805	11,878	27	133.38	121.20	146.83	32
		2001	10,749	8,864	12,151	28	131.56	108.48	148.72	31
		2002	9,905	7,752	12,521	28	120.36	94.20	152.14	33
		2003	9,420	6,722	12,789	28	113.83	81.22	154.53	32
		2004	8,943	6,003	12,892	28	107.91	72.44	155.55	34
		2005	8,439	5,286	12,896	31	101.80	63.77	155.56	37
		2006	7,888	4,604	12,737	32	94.99	55.44	153.37	39
		2007	7,319	3,973	12,449	32	88.07	47.81	149.80	41

Metropolitan Statistical Area	PWID Population	Year	Number	Min	Max	Rank	Rate	Min	Max	Rank
New Orleans, LA	Total	1992	16,835	12,753	19,701	29	196.51	148.86	229.97	17
		1993	13,427	3,754	20,288	32	155.78	43.55	235.38	27
		1994	16,481	11,913	20,890	28	189.93	137.30	240.75	16
		1995	13,244	3,962	21,524	30	151.43	45.31	246.11	27
		1996	13,125	4,050	22,076	31	149.51	46.14	251.48	26
		1997	13,028	4,137	22,657	32	147.69	46.90	256.85	26
		1998	12,934	4,222	23,193	32	145.97	47.65	261.75	25
		1999	15,692	9,959	23,730	28	176.33	111.91	266.65	11
		2000	12,730	4,497	23,954	31	142.92	50.49	268.93	21
		2001	15,249	9,440	24,150	28	171.25	106.02	271.21	14
		2002	12,395	4,471	24,417	33	138.82	50.07	273.45	22
		2003	12,231	4,461	24,666	34	136.72	49.87	275.70	24
		2004	12,105	4,450	24,956	36	134.71	49.53	277.73	23
		2005	11,943	4,413	25,169	36	132.75	49.05	279.76	22
		2006	9,096	3,378	19,573	47	130.79	48.56	281.42	22
		2007	9,970	3,708	21,915	42	128.79	47.89	283.09	23
	Non-Hispanic White	1992	7,724	5,851	9,039	28	150.91	114.32	176.61	19
		1993	6,281	1,756	9,490	34	123.09	34.42	186.00	27
		1994	7,784	5,627	9,867	27	152.83	110.48	193.72	16
		1995	6,270	1,876	10,190	31	123.23	36.87	200.28	24
		1996	6,194	1,911	10,418	31	122.34	37.75	205.77	21
		1997	6,108	1,940	10,623	33	120.92	38.40	210.29	20
		1998	6,015	1,964	10,786	34	119.35	38.96	214.01	21
		1999	7,240	4,595	10,949	27	144.20	91.52	218.06	11
		2000	5,840	2,063	10,989	35	117.16	41.38	220.45	20
		2001	6,983	4,323	11,060	29	140.96	87.27	223.24	14
		2002	5,700	2,056	11,229	37	115.40	41.63	227.32	19
		2003	5,692	2,076	11,478	37	115.61	42.17	233.13	21
		2004	5,751	2,114	11,856	37	116.94	42.99	241.08	21
		2005	5,849	2,161	12,327	37	119.53	44.16	251.91	21
		2006	4,639	1,723	9,983	47	111.05	41.23	238.94	25
		2007	5,347	1,988	11,752	42	119.54	44.45	262.75	21

Metropolitan Statistical Area	PWID Population	Year	Number	Min	Max	Rank	Rate	Min	Max	Rank
New Orleans, LA	Non-Hispanic Black	1992	8,578	6,498	10,039	13	297.59	225.43	348.26	49
		1993	6,537	1,828	9,878	13	222.35	62.17	335.97	57
		1994	7,752	5,604	9,826	13	259.02	187.24	328.32	55
		1995	6,080	1,819	9,882	16	199.42	59.67	324.11	61
		1996	5,934	1,831	9,981	16	191.76	59.18	322.53	60
		1997	5,842	1,855	10,160	16	186.15	59.11	323.74	57
		1998	5,784	1,888	10,372	15	181.83	59.36	326.04	55
		1999	7,022	4,457	10,619	13	217.63	138.12	329.09	43
		2000	5,709	2,017	10,743	15	175.23	61.90	329.72	51
		2001	6,850	4,241	10,849	13	209.33	129.59	331.52	36
		2002	5,564	2,007	10,960	16	168.84	60.90	332.59	48
		2003	5,463	1,992	11,016	16	164.86	60.13	332.45	47
		2004	5,345	1,965	11,020	16	159.92	58.80	329.70	45
		2005	5,172	1,911	10,900	16	153.80	56.82	324.12	48
		2006	3,825	1,420	8,231	19	178.25	66.19	383.54	43
		2007	4,024	1,497	8,846	19	157.05	58.40	345.20	49
	Hispanic	1992	287	218	336	63	75.69	57.33	88.57	67
		1993	212	59	321	64	56.10	15.69	84.77	77
		1994	246	178	312	64	64.23	46.43	81.41	75
		1995	189	57	307	67	48.41	14.49	78.68	77
		1996	182	56	306	68	46.41	14.32	78.06	78
		1997	177	56	309	68	44.87	14.25	78.03	77
		1998	175	57	314	67	43.92	14.34	78.75	75
		1999	214	136	323	66	53.30	33.83	80.60	68
		2000	176	62	332	69	43.47	15.36	81.80	72
		2001	216	134	343	68	51.88	32.12	82.16	65
		2002	182	66	358	71	42.39	15.29	83.50	68
		2003	187	68	377	72	42.52	15.51	85.74	67
		2004	195	72	401	72	43.15	15.86	88.96	68
		2005	203	75	429	72	43.98	16.25	92.68	67
		2006	165	61	356	75	41.23	15.31	88.71	69
		2007	195	73	430	74	43.27	16.09	95.12	67

Metropolitan Statistical Area	PWID Population	Year	Number	Min	Max	Rank	Rate	Min	Max	Rank
New Orleans, LA	Male	1992	12,462	9,440	14,584	24	302.37	229.06	353.86	13
		1993	9,852	2,755	14,887	28	238.04	66.55	359.69	19
		1994	11,988	8,666	15,195	23	287.39	207.75	364.27	9
		1995	9,550	2,857	15,520	29	227.13	67.96	369.14	17
		1996	9,383	2,896	15,782	29	222.41	68.63	374.08	17
		1997	9,236	2,933	16,063	29	217.82	69.17	378.82	15
		1998	9,097	2,970	16,311	29	213.47	69.69	382.78	15
		1999	10,953	6,952	16,564	21	255.82	162.36	386.85	9
		2000	8,824	3,117	16,604	31	205.75	72.68	387.15	17
		2001	10,503	6,502	16,633	24	244.80	151.55	387.70	9
		2002	8,490	3,062	16,724	33	197.25	71.15	388.55	19
		2003	8,339	3,042	16,816	33	193.54	70.60	390.30	19
		2004	8,222	3,023	16,951	33	189.71	69.75	391.11	18
		2005	8,091	2,989	17,051	34	186.46	68.89	392.96	18
		2006	6,154	2,285	13,241	40	183.01	67.96	393.80	18
		2007	6,744	2,508	14,824	38	180.45	67.10	396.64	19
	Female	1992	4,597	3,482	5,379	34	103.40	78.33	121.01	33
		1993	3,783	1,058	5,716	37	84.44	23.61	127.59	39
		1994	4,776	3,453	6,054	35	106.00	76.63	134.37	27
		1995	3,937	1,178	6,398	36	86.68	25.93	140.87	36
		1996	3,990	1,231	6,711	34	87.51	27.01	147.19	35
		1997	4,041	1,283	7,028	36	88.21	28.01	153.42	33
		1998	4,084	1,333	7,323	36	88.80	28.99	159.22	33
		1999	5,033	3,195	7,611	32	109.01	69.18	164.84	19
		2000	4,140	1,462	7,790	38	89.64	31.66	168.67	29
		2001	5,019	3,107	7,949	32	108.78	67.34	172.28	18
		2002	4,123	1,487	8,122	37	89.16	32.16	175.62	29
		2003	4,106	1,498	8,281	38	88.53	32.29	178.53	27
		2004	4,096	1,506	8,445	37	88.06	32.38	181.55	27
		2005	4,070	1,504	8,577	37	87.38	32.29	184.15	25
		2006	3,119	1,158	6,712	47	86.83	32.24	186.83	25
		2007	3,438	1,279	7,558	43	85.87	31.93	188.76	27

Metropolitan Statistical Area	PWID Population	Year	Number	Min	Max	Rank	Rate	Min	Max	Rank
New Orleans, LA	Young (15-29)	1992	4,332	3,281	5,069	25	147.95	112.08	173.14	17
		1993	3,473	971	5,247	31	119.79	33.49	181.00	27
		1994	4,413	3,190	5,594	24	152.95	110.57	193.87	15
		1995	3,757	1,124	6,106	26	130.15	38.94	211.52	24
		1996	4,007	1,236	6,739	24	139.16	42.94	234.06	17
		1997	4,319	1,371	7,511	23	150.11	47.67	261.07	14
		1998	4,667	1,524	8,369	21	162.41	53.02	291.23	11
		1999	6,144	3,899	9,291	15	214.73	136.28	324.72	5
		2000	5,365	1,895	10,095	18	189.31	66.87	356.22	7
		2001	6,834	4,231	10,824	14	243.37	150.67	385.43	5
		2002	5,820	2,099	11,464	18	207.74	74.93	409.20	7
		2003	5,910	2,155	11,917	17	211.23	77.05	425.96	7
		2004	5,895	2,167	12,153	18	209.11	76.88	431.11	11
		2005	5,720	2,113	12,055	18	202.59	74.85	426.94	12
		2006	4,159	1,544	8,950	32	200.51	74.45	431.44	13
		2007	4,193	1,559	9,215	31	177.52	66.02	390.20	16
	Old (30-64)	1992	12,661	9,591	14,816	28	224.52	170.08	262.75	17
		1993	10,104	2,825	15,267	31	176.64	49.39	266.90	25
		1994	12,276	8,874	15,561	25	211.96	153.22	268.67	17
		1995	9,673	2,894	15,721	32	165.09	49.39	268.31	25
		1996	9,318	2,876	15,673	33	157.96	48.75	265.69	27
		1997	8,920	2,832	15,513	33	150.06	47.65	260.97	28
		1998	8,483	2,769	15,211	34	141.69	46.26	254.08	29
		1999	9,813	6,228	14,840	32	162.53	103.15	245.78	21
		2000	7,580	2,678	14,264	38	124.81	44.09	234.85	36
		2001	8,672	5,368	13,733	35	142.24	88.06	225.27	27
		2002	6,788	2,448	13,370	40	110.77	39.96	218.20	37
		2003	6,539	2,385	13,187	40	106.35	38.79	214.47	38
		2004	6,441	2,368	13,279	40	104.45	38.40	215.33	38
		2005	6,475	2,392	13,646	39	104.89	38.75	221.04	32
		2006	5,155	1,914	11,093	49	105.63	39.22	227.29	29
		2007	6,052	2,251	13,302	41	112.49	41.83	247.26	23

Metropolitan Statistical Area	PWID Population	Year	Number	Min	Max	Rank	Rate	Min	Max	Rank
New York, NY	Total	1992	109,820	36,685	172,972	1	188.53	62.98	296.94	19
		1993	110,884	38,021	164,818	1	188.75	64.72	280.56	15
		1994	103,815	39,346	156,287	1	175.54	66.53	264.27	18
		1995	105,295	40,697	148,140	1	176.82	68.34	248.76	16
		1996	103,014	42,301	140,829	1	171.29	70.34	234.17	15
		1997	101,005	44,031	134,176	1	165.94	72.34	220.43	15
		1998	99,315	45,590	128,174	1	160.78	73.80	207.50	14
		1999	92,143	47,002	121,967	1	147.57	75.27	195.33	23
		2000	97,808	47,522	120,992	1	155.32	75.46	192.13	15
		2001	88,076	48,026	109,871	1	138.75	75.66	173.08	23
		2002	102,284	48,789	149,110	1	160.38	76.50	233.80	10
		2003	105,580	49,575	166,077	1	164.72	77.34	259.11	10
		2004	107,678	50,444	177,590	1	166.88	78.18	275.23	10
		2005	106,849	51,363	176,902	1	164.36	79.01	272.11	10
		2006	105,490	52,095	174,080	1	161.30	79.66	266.19	10
		2007	103,877	52,917	169,859	1	157.64	80.31	257.78	13
	Non-Hispanic White	1992	29,738	9,934	46,838	2	112.39	37.54	177.02	32
		1993	30,158	10,341	44,827	2	115.14	39.48	171.14	31
		1994	28,263	10,712	42,548	2	109.06	41.33	164.18	31
		1995	28,589	11,050	40,221	3	111.38	43.05	156.71	29
		1996	27,788	11,411	37,989	3	108.89	44.71	148.86	27
		1997	26,963	11,754	35,818	3	106.03	46.22	140.85	29
		1998	26,129	11,994	33,722	3	102.69	47.14	132.53	33
		1999	23,793	12,137	31,494	4	93.74	47.82	124.08	34
		2000	24,681	11,992	30,532	4	97.89	47.56	121.09	33
		2001	21,626	11,792	26,977	4	85.30	46.51	106.41	41
		2002	24,330	11,605	35,468	3	96.11	45.85	140.12	34
		2003	24,222	11,373	38,102	3	95.66	44.92	150.48	37
		2004	23,723	11,113	39,126	3	93.34	43.73	153.95	36
		2005	22,511	10,821	37,269	4	88.14	42.37	145.92	41
		2006	21,167	10,453	34,929	5	82.63	40.81	136.36	45
		2007	19,777	10,075	32,339	5	76.82	39.13	125.61	51

Metropolitan Statistical Area	PWID Population	Year	Number	Min	Max	Rank	Rate	Min	Max	Rank
New York, NY	Non-Hispanic Black	1992	41,908	13,999	66,007	1	307.80	102.82	484.80	47
		1993	41,263	14,149	61,333	1	300.13	102.91	446.11	44
		1994	37,445	14,192	56,371	1	269.90	102.29	406.33	49
		1995	36,631	14,158	51,536	1	261.91	101.23	368.48	45
		1996	34,441	14,143	47,084	1	244.14	100.25	333.77	47
		1997	32,384	14,117	43,019	1	226.59	98.78	301.00	47
		1998	30,521	14,011	39,390	1	210.26	96.52	271.36	47
		1999	27,180	13,864	35,978	2	185.32	94.53	245.30	53
		2000	27,787	13,501	34,374	2	188.17	91.43	232.77	45
		2001	24,234	13,214	30,231	2	164.17	89.52	204.79	59
		2002	27,469	13,103	40,045	2	186.04	88.74	271.22	40
		2003	27,954	13,126	43,972	2	189.08	88.78	297.42	36
		2004	28,454	13,330	46,928	2	191.82	89.86	316.37	35
		2005	28,588	13,742	47,331	2	192.22	92.40	318.24	35
		2006	29,046	14,344	47,931	2	195.09	96.34	321.94	35
		2007	29,954	15,259	48,981	2	201.35	102.57	329.25	34
	Hispanic	1992	39,432	13,172	62,107	1	294.31	98.31	463.56	17
		1993	39,010	13,376	57,985	1	283.72	97.28	421.72	18
		1994	36,110	13,685	54,361	1	256.95	97.38	386.83	19
		1995	36,517	14,114	51,376	1	254.47	98.36	358.02	16
		1996	35,901	14,742	49,080	1	244.80	100.52	334.66	17
		1997	35,623	15,529	47,322	1	237.27	103.44	315.20	16
		1998	35,669	16,373	46,034	1	232.06	106.52	299.49	15
		1999	33,884	17,284	44,851	1	216.35	110.36	286.38	17
		2000	36,999	17,977	45,769	1	231.33	112.40	286.16	15
		2001	34,408	18,762	42,923	1	212.42	115.83	264.99	16
		2002	41,401	19,748	60,356	1	252.66	120.52	368.33	13
		2003	44,394	20,845	69,832	1	268.23	125.95	421.93	12
		2004	47,128	22,078	77,726	1	282.03	132.12	465.14	12
		2005	48,745	23,432	80,704	1	288.76	138.81	478.07	12
		2006	50,203	24,792	82,845	1	294.75	145.56	486.40	12
		2007	51,578	26,275	84,339	1	299.10	152.37	489.08	12

Metropolitan Statistical Area	PWID Population	Year	Number	Min	Max	Rank	Rate	Min	Max	Rank
New York, NY	Male	1992	72,420	24,192	114,064	1	259.92	86.83	409.39	17
		1993	74,194	25,440	110,283	1	263.85	90.47	392.18	12
		1994	70,313	26,649	105,852	1	248.35	94.12	373.87	18
		1995	72,055	27,849	101,374	1	252.72	97.68	355.55	12
		1996	71,135	29,211	97,248	1	247.08	101.46	337.78	11
		1997	70,329	30,659	93,427	1	241.28	105.18	320.51	9
		1998	69,710	32,000	89,966	1	235.63	108.17	304.10	9
		1999	65,206	33,261	86,311	1	217.89	111.14	288.41	16
		2000	69,822	33,925	86,372	1	231.18	112.33	285.98	9
		2001	63,483	34,616	79,193	1	208.25	113.56	259.79	16
		2002	74,530	35,551	108,651	1	243.13	115.97	354.44	7
		2003	77,890	36,573	122,521	1	252.47	118.55	397.13	7
		2004	80,563	37,741	132,870	1	259.13	121.39	427.38	8
		2005	81,220	39,043	134,470	1	258.85	124.43	428.56	8
		2006	81,616	40,305	134,683	1	258.26	127.54	426.18	8
		2007	81,938	41,741	133,985	1	256.89	130.87	420.07	8
	Female	1992	31,517	10,528	49,641	2	103.71	34.64	163.35	32
		1993	32,417	11,115	48,185	2	105.85	36.29	157.33	26
		1994	30,828	11,684	46,409	1	100.00	37.90	150.54	32
		1995	31,646	12,231	44,523	2	101.96	39.41	143.44	25
		1996	31,206	12,814	42,661	1	99.54	40.88	136.08	25
		1997	30,691	13,379	40,771	1	96.75	42.18	128.53	26
		1998	30,104	13,819	38,852	1	93.53	42.93	120.71	27
		1999	27,690	14,125	36,653	1	85.16	43.44	112.72	38
		2000	28,937	14,060	35,796	1	88.30	42.90	109.23	31
		2001	25,454	13,879	31,752	2	77.14	42.06	96.23	46
		2002	28,622	13,653	41,725	1	86.41	41.22	125.98	32
		2003	28,327	13,301	44,558	1	85.21	40.01	134.03	32
		2004	27,396	12,834	45,183	1	81.94	38.38	135.13	31
		2005	25,465	12,241	42,160	1	75.71	36.40	125.35	40
		2006	23,232	11,473	38,337	3	68.74	33.95	113.44	44
		2007	20,826	10,609	34,054	3	61.26	31.21	100.17	52

Metropolitan Statistical Area	PWID Population	Year	Number	Min	Max	Rank	Rate	Min	Max	Rank
New York, NY	Young (15-29)	1992	21,150	7,065	33,312	2	106.64	35.62	167.97	36
		1993	19,966	6,846	29,678	2	101.08	34.66	150.24	44
		1994	18,117	6,866	27,274	1	92.25	34.96	138.88	42
		1995	18,351	7,093	25,818	1	93.81	36.26	131.98	46
		1996	18,354	7,537	25,092	1	93.62	38.44	127.98	46
		1997	18,704	8,154	24,847	2	94.63	41.25	125.70	44
		1998	19,298	8,858	24,905	2	96.57	44.33	124.63	45
		1999	18,839	9,610	24,936	1	93.80	47.85	124.16	44
		2000	20,963	10,185	25,931	2	104.21	50.63	128.91	42
		2001	19,592	10,683	24,441	2	98.15	53.52	122.44	47
		2002	23,242	11,086	33,882	2	117.50	56.05	171.30	36
		2003	23,974	11,257	37,712	2	121.86	57.22	191.69	32
		2004	23,754	11,128	39,176	2	121.04	56.70	199.62	32
		2005	22,106	10,627	36,600	3	112.49	54.08	186.25	39
		2006	19,602	9,680	32,348	3	99.69	49.23	164.52	48
		2007	16,448	8,379	26,896	4	83.34	42.46	136.28	55
	Old (30-64)	1992	89,749	29,980	141,359	1	233.61	78.04	367.95	15
		1993	91,602	31,409	136,156	1	234.92	80.55	349.18	8
		1994	86,182	32,663	129,742	1	218.17	82.69	328.45	15
		1995	87,442	33,797	123,022	1	218.67	84.52	307.65	8
		1996	85,268	35,014	116,569	1	210.36	86.38	287.58	8
		1997	83,083	36,219	110,369	1	202.13	88.12	268.51	8
		1998	80,992	37,178	104,527	1	193.82	88.97	250.14	11
		1999	74,375	37,938	98,447	1	175.58	89.56	232.41	14
		2000	78,082	37,938	96,590	1	182.19	88.52	225.38	11
		2001	69,575	37,938	86,792	2	159.88	87.18	199.44	16
		2002	80,101	38,208	116,773	1	182.06	86.84	265.41	8
		2003	82,253	38,621	129,384	1	185.16	86.94	291.26	8
		2004	83,866	39,289	138,318	1	186.79	87.50	308.06	8
		2005	83,713	40,242	138,597	1	184.56	88.72	305.55	8
		2006	83,694	41,331	138,113	1	183.00	90.37	301.98	8
		2007	83,979	42,781	137,322	1	181.94	92.68	297.50	8

Metropolitan Statistical Area	PWID Population	Year	Number	Min	Max	Rank	Rate	Min	Max	Rank
Newark, NJ	Total	1992	22,952	17,418	30,577	19	177.50	134.71	236.47	22
		1993	19,583	12,414	28,154	23	151.06	95.76	217.18	30
		1994	20,991	17,551	25,718	18	161.64	135.15	198.04	21
		1995	18,369	13,192	23,489	22	141.02	101.27	180.32	32
		1996	17,779	13,682	21,500	23	135.76	104.48	164.18	33
		1997	17,325	14,255	19,726	23	131.29	108.02	149.48	34
		1998	17,015	14,992	18,116	23	127.91	112.70	136.19	32
		1999	17,034	16,245	18,266	22	127.22	121.33	136.42	34
		2000	16,522	15,194	18,475	23	122.69	112.83	137.19	36
		2001	15,791	13,923	18,697	25	116.51	102.73	137.95	40
		2002	15,985	12,752	18,898	24	117.25	93.53	138.62	35
		2003	15,679	11,649	19,051	24	114.63	85.16	139.28	35
		2004	15,545	10,625	19,184	24	113.44	77.54	140.00	32
		2005	15,872	9,687	19,308	22	115.68	70.60	140.72	30
		2006	16,267	8,824	21,455	22	118.33	64.19	156.07	28
		2007	16,690	7,958	24,831	21	121.32	57.84	180.49	27
	Non-Hispanic White	1992	6,922	5,253	9,221	33	84.43	64.07	112.48	44
		1993	6,039	3,828	8,682	35	74.34	47.13	106.88	53
		1994	6,632	5,545	8,125	32	82.33	68.84	100.87	47
		1995	5,956	4,278	7,616	34	74.45	53.47	95.20	52
		1996	5,926	4,560	7,166	33	74.49	57.32	90.08	53
		1997	5,944	4,891	6,768	34	74.91	61.63	85.29	53
		1998	6,016	5,301	6,406	33	75.98	66.95	80.90	53
		1999	6,215	5,927	6,664	34	78.69	75.04	84.38	52
		2000	6,225	5,725	6,961	33	79.08	72.72	88.42	48
		2001	6,149	5,422	7,281	35	78.36	69.09	92.78	54
		2002	6,437	5,135	7,610	31	82.32	65.67	97.32	41
		2003	6,531	4,852	7,935	31	83.94	62.36	101.99	41
		2004	6,698	4,578	8,266	29	86.56	59.16	106.82	40
		2005	7,073	4,317	8,604	28	91.89	56.08	111.78	40
		2006	7,492	4,064	9,882	28	97.78	53.04	128.97	37
		2007	7,939	3,785	11,811	26	104.32	49.74	155.20	31

Metropolitan Statistical Area	PWID Population	Year	Number	Min	Max	Rank	Rate	Min	Max	Rank
Newark, NJ	Non-Hispanic Black	1992	12,077	9,165	16,089	8	423.28	321.23	563.91	27
		1993	9,911	6,283	14,249	9	344.83	218.60	495.75	35
		1994	10,211	8,538	12,511	9	353.30	295.40	432.85	29
		1995	8,585	6,166	10,978	12	295.55	212.26	377.92	40
		1996	7,983	6,143	9,654	12	272.68	209.84	329.75	39
		1997	7,476	6,151	8,512	12	253.04	208.19	288.10	40
		1998	7,061	6,221	7,518	13	236.53	208.41	251.84	38
		1999	6,808	6,493	7,300	14	226.37	215.88	242.74	40
		2000	6,373	5,861	7,126	13	211.04	194.07	235.97	38
		2001	5,895	5,198	6,980	15	194.83	171.78	230.68	42
		2002	5,796	4,624	6,852	15	190.86	152.26	225.64	37
		2003	5,546	4,120	6,739	15	182.44	135.55	221.68	39
		2004	5,393	3,686	6,655	15	177.39	121.25	218.91	39
		2005	5,434	3,316	6,610	15	179.29	109.42	218.10	40
		2006	5,535	3,003	7,300	15	182.94	99.24	241.29	39
		2007	5,691	2,713	8,466	15	188.26	89.76	280.08	39
	Hispanic	1992	3,936	2,987	5,244	19	274.32	208.18	365.46	20
		1993	3,334	2,114	4,793	22	223.03	141.38	320.64	28
		1994	3,574	2,988	4,378	22	231.06	193.19	283.09	23
		1995	3,147	2,260	4,024	24	196.23	140.93	250.93	28
		1996	3,082	2,372	3,727	24	184.67	142.11	223.32	29
		1997	3,053	2,512	3,476	25	175.83	144.67	200.20	27
		1998	3,060	2,696	3,258	25	169.67	149.51	180.66	24
		1999	3,136	2,991	3,363	24	168.64	160.82	180.83	21
		2000	3,123	2,872	3,492	24	162.60	149.53	181.81	22
		2001	3,071	2,707	3,636	24	153.68	135.50	181.96	23
		2002	3,203	2,555	3,787	22	154.50	123.25	182.66	21
		2003	3,242	2,408	3,939	21	151.68	112.69	184.30	21
		2004	3,320	2,269	4,097	21	151.70	103.69	187.21	22
		2005	3,506	2,139	4,264	20	156.22	95.34	190.04	21
		2006	3,719	2,017	4,905	20	162.20	87.99	213.93	20
		2007	3,954	1,885	5,883	18	168.67	80.42	250.94	21

Metropolitan Statistical Area	PWID Population	Year	Number	Min	Max	Rank	Rate	Min	Max	Rank
Newark, NJ	Male	1992	15,335	11,638	20,430	14	243.04	184.44	323.78	20
		1993	13,125	8,320	18,870	21	207.48	131.53	298.29	31
		1994	14,097	11,787	17,272	17	222.41	185.96	272.49	23
		1995	12,349	8,868	15,790	21	194.14	139.43	248.25	31
		1996	11,952	9,198	14,454	22	186.93	143.85	226.05	32
		1997	11,638	9,575	13,250	21	180.66	148.64	205.69	32
		1998	11,411	10,054	12,149	21	175.74	154.85	187.12	32
		1999	11,398	10,870	12,222	20	174.42	166.34	187.03	33
		2000	11,023	10,137	12,326	21	167.70	154.22	187.52	31
		2001	10,499	9,257	12,431	25	158.53	139.78	187.70	35
		2002	10,586	8,445	12,515	22	158.69	126.59	187.61	32
		2003	10,338	7,681	12,562	22	154.28	114.63	187.47	34
		2004	10,202	6,973	12,590	23	151.86	103.79	187.40	33
		2005	10,365	6,326	12,609	21	153.87	93.91	187.18	31
		2006	10,569	5,733	13,939	20	156.40	84.84	206.29	27
		2007	10,787	5,143	16,049	20	159.28	75.94	236.97	27
	Female	1992	7,604	5,770	10,130	19	114.85	87.16	153.00	23
		1993	6,457	4,093	9,283	22	97.28	61.67	139.85	29
		1994	6,922	5,787	8,480	19	104.12	87.06	127.57	29
		1995	6,082	4,368	7,777	23	91.24	65.53	116.67	33
		1996	5,929	4,562	7,170	23	88.47	68.08	106.98	33
		1997	5,832	4,798	6,640	23	86.34	71.04	98.31	34
		1998	5,789	5,101	6,164	23	85.02	74.92	90.53	38
		1999	5,862	5,590	6,286	23	85.52	81.55	91.70	36
		2000	5,748	5,286	6,427	24	83.38	76.68	93.24	37
		2001	5,548	4,892	6,569	27	80.05	70.58	94.78	40
		2002	5,660	4,515	6,692	24	81.30	64.85	96.11	35
		2003	5,580	4,146	6,781	25	79.98	59.42	97.18	35
		2004	5,542	3,788	6,839	24	79.35	54.23	97.92	36
		2005	5,644	3,444	6,865	24	80.80	49.31	98.29	32
		2006	5,740	3,114	7,571	24	82.12	44.55	108.31	29
		2007	5,810	2,770	8,644	23	83.19	39.66	123.76	28

Metropolitan Statistical Area	PWID Population	Year	Number	Min	Max	Rank	Rate	Min	Max	Rank
Newark, NJ	Young (15-29)	1992	5,707	4,331	7,603	20	140.88	106.92	187.69	20
		1993	4,871	3,088	7,003	21	122.95	77.94	176.76	26
		1994	5,289	4,423	6,481	19	136.57	114.19	167.33	20
		1995	4,737	3,402	6,057	21	124.52	89.43	159.22	26
		1996	4,727	3,638	5,716	21	125.82	96.82	152.15	25
		1997	4,774	3,928	5,435	20	127.94	105.26	145.67	24
		1998	4,871	4,292	5,186	20	130.83	115.28	139.30	24
		1999	5,069	4,834	5,435	19	136.45	130.12	146.31	24
		2000	5,100	4,690	5,703	20	137.86	126.78	154.15	21
		2001	5,037	4,441	5,963	21	136.83	120.64	162.01	28
		2002	5,236	4,177	6,191	22	142.34	113.55	168.28	23
		2003	5,234	3,888	6,359	22	141.74	105.31	172.23	25
		2004	5,236	3,579	6,462	22	141.12	96.46	174.15	24
		2005	5,332	3,254	6,487	23	142.56	87.01	173.42	26
		2006	5,376	2,916	7,091	20	142.33	77.21	187.72	25
		2007	5,340	2,546	7,945	22	140.39	66.94	208.87	25
	Old (30-64)	1992	17,510	13,288	23,327	15	197.20	149.65	262.71	20
		1993	14,832	9,402	21,324	21	164.77	104.45	236.88	29
		1994	15,777	13,191	19,330	18	173.13	144.75	212.11	24
		1995	13,696	9,836	17,513	24	148.51	106.66	189.90	32
		1996	13,145	10,115	15,896	24	140.75	108.32	170.21	36
		1997	12,694	10,444	14,453	24	134.11	110.34	152.70	36
		1998	12,346	10,879	13,145	25	128.89	113.57	137.23	38
		1999	12,233	11,666	13,117	25	126.44	120.58	135.58	36
		2000	11,734	10,791	13,120	24	120.14	110.48	134.33	38
		2001	11,085	9,773	13,125	26	112.28	99.00	132.94	41
		2002	11,086	8,844	13,106	25	111.37	88.84	131.66	36
		2003	10,741	7,980	13,052	25	107.56	79.91	130.70	36
		2004	10,520	7,190	12,982	24	105.28	71.96	129.92	37
		2005	10,610	6,475	12,907	24	106.30	64.88	129.32	31
		2006	10,740	5,826	14,166	23	107.73	58.44	142.09	28
		2007	10,880	5,187	16,187	22	109.31	52.12	162.62	26

Metropolitan Statistical Area	PWID Population	Year	Number	Min	Max	Rank	Rate	Min	Max	Rank
Norfolk--Virginia Beach--Newport News, VA--NC	Total	1992	7,990	5,919	9,794	57	78.94	58.48	96.77	72
		1993	7,844	5,185	11,035	51	76.53	50.59	107.66	70
		1994	9,385	6,822	12,116	46	91.13	66.25	117.66	60
		1995	8,788	5,029	13,182	45	84.95	48.61	127.42	61
		1996	9,267	4,962	14,205	45	89.09	47.71	136.56	54
		1997	9,503	4,874	14,282	44	91.02	46.69	136.80	53
		1998	9,482	4,759	13,755	41	91.00	45.67	132.01	54
		1999	11,142	8,430	13,211	38	105.97	80.18	125.65	46
		2000	9,439	4,713	12,482	42	88.67	44.28	117.25	56
		2001	10,744	7,667	13,125	40	100.38	71.64	122.63	51
		2002	9,107	4,468	13,913	45	83.96	41.19	128.27	59
		2003	9,104	4,352	14,728	45	82.94	39.64	134.17	58
		2004	9,201	4,234	15,625	45	82.64	38.03	140.35	58
		2005	9,264	4,042	16,416	45	82.84	36.15	146.81	57
		2006	9,418	3,874	17,350	43	83.33	34.28	153.51	57
		2007	9,451	3,625	18,070	45	83.81	32.15	160.25	57
	Non-Hispanic White	1992	3,488	2,584	4,276	65	51.34	38.03	62.93	72
		1993	3,365	2,224	4,734	63	49.27	32.57	69.31	67
		1994	3,958	2,877	5,110	58	58.10	42.24	75.01	63
		1995	3,649	2,088	5,473	61	53.81	30.79	80.72	64
		1996	3,796	2,033	5,819	57	56.21	30.10	86.16	63
		1997	3,851	1,975	5,788	54	57.36	29.42	86.21	62
		1998	3,817	1,915	5,537	53	57.47	28.84	83.37	63
		1999	4,475	3,386	5,306	46	67.38	50.99	79.90	60
		2000	3,803	1,899	5,029	55	57.01	28.47	75.40	66
		2001	4,370	3,118	5,338	49	65.47	46.72	79.98	59
		2002	3,764	1,847	5,751	57	55.94	27.45	85.47	66
		2003	3,853	1,842	6,233	56	56.80	27.15	91.90	66
		2004	4,016	1,848	6,820	53	58.64	26.98	99.59	66
		2005	4,200	1,833	7,443	56	61.47	26.83	108.94	66
		2006	4,465	1,837	8,226	55	65.09	26.77	119.91	62
		2007	4,713	1,808	9,012	51	69.23	26.56	132.37	61

Metropolitan Statistical Area	PWID Population	Year	Number	Min	Max	Rank	Rate	Min	Max	Rank
Norfolk--Virginia Beach--Newport News, VA--NC	Non-Hispanic Black	1992	4,181	3,097	5,125	25	150.06	111.17	183.95	88
		1993	4,121	2,724	5,798	26	144.35	95.42	203.07	86
		1994	4,955	3,602	6,397	22	170.55	123.98	220.19	80
		1995	4,662	2,668	6,993	20	157.49	90.12	236.23	76
		1996	4,933	2,642	7,562	17	163.54	87.57	250.68	72
		1997	5,065	2,598	7,612	17	165.02	84.64	248.02	65
		1998	5,042	2,530	7,315	17	162.54	81.57	235.79	65
		1999	5,884	4,453	6,978	16	185.94	140.70	220.49	52
		2000	4,923	2,458	6,510	17	152.51	76.16	201.68	64
		2001	5,494	3,921	6,712	17	168.39	120.17	205.70	57
		2002	4,527	2,221	6,917	17	136.25	66.84	208.16	64
		2003	4,355	2,082	7,046	17	128.88	61.60	208.50	64
		2004	4,184	1,925	7,106	17	121.82	56.06	206.88	65
		2005	3,949	1,723	6,998	19	113.48	49.52	201.09	66
		2006	3,704	1,523	6,823	20	104.61	43.03	192.71	69
		2007	3,369	1,292	6,441	21	94.78	36.36	181.21	77
	Hispanic	1992	68	51	84	82	28.85	21.37	35.36	95
		1993	81	53	114	77	32.98	21.80	46.40	92
		1994	113	82	145	77	45.05	32.75	58.16	86
		1995	119	68	179	74	45.62	26.11	68.44	81
		1996	138	74	211	72	50.10	26.83	76.79	74
		1997	152	78	229	71	52.94	27.15	79.56	73
		1998	161	81	233	70	53.97	27.08	78.29	71
		1999	198	150	235	68	63.29	47.89	75.05	64
		2000	175	87	231	71	52.70	26.32	69.69	65
		2001	208	148	254	69	60.23	42.98	73.58	63
		2002	186	91	283	69	50.82	24.93	77.64	63
		2003	198	94	320	69	52.53	25.11	84.97	63
		2004	217	100	368	69	53.81	24.76	91.39	63
		2005	243	106	431	68	57.85	25.24	102.52	61
		2006	284	117	524	67	63.79	26.24	117.52	58
		2007	340	131	651	65	74.77	28.68	142.96	54

Metropolitan Statistical Area	PWID Population	Year	Number	Min	Max	Rank	Rate	Min	Max	Rank
Norfolk--Virginia Beach--Newport News, VA--NC	Male	1992	4,490	3,326	5,504	62	86.74	64.26	106.33	87
		1993	4,461	2,949	6,276	60	85.46	56.49	120.23	82
		1994	5,371	3,904	6,934	52	102.89	74.79	132.84	75
		1995	5,038	2,883	7,556	49	96.39	55.16	144.58	71
		1996	5,302	2,839	8,127	44	101.21	54.20	155.14	65
		1997	5,413	2,776	8,135	45	103.21	52.94	155.11	63
		1998	5,368	2,694	7,788	45	102.85	51.61	149.20	61
		1999	6,267	4,742	7,431	43	119.05	90.08	141.17	56
		2000	5,277	2,635	6,978	47	99.01	49.45	130.93	63
		2001	5,980	4,267	7,305	44	111.67	79.69	136.41	58
		2002	5,059	2,482	7,730	47	93.45	45.85	142.78	66
		2003	5,067	2,422	8,198	47	93.23	44.56	150.82	65
		2004	5,156	2,372	8,756	48	93.35	42.95	158.53	63
		2005	5,256	2,294	9,314	48	95.06	41.48	168.46	62
		2006	5,445	2,240	10,031	48	96.96	39.88	178.62	61
		2007	5,607	2,151	10,720	47	100.26	38.46	191.70	59
	Female	1992	3,150	2,333	3,861	45	63.69	47.18	78.07	61
		1993	3,140	2,076	4,417	42	62.42	41.26	87.81	56
		1994	3,818	2,775	4,929	39	75.19	54.66	97.07	46
		1995	3,635	2,080	5,452	39	71.00	40.63	106.50	49
		1996	3,894	2,085	5,969	37	75.42	40.39	115.61	46
		1997	4,053	2,079	6,091	35	78.00	40.01	117.23	46
		1998	4,096	2,056	5,942	35	78.78	39.54	114.28	45
		1999	4,864	3,680	5,767	34	92.64	70.10	109.85	31
		2000	4,151	2,073	5,489	37	78.09	39.00	103.27	46
		2001	4,742	3,384	5,793	35	88.67	63.28	108.32	33
		2002	4,017	1,971	6,137	39	73.93	36.27	112.95	44
		2003	3,992	1,908	6,459	40	72.05	34.44	116.56	43
		2004	3,989	1,835	6,774	39	71.10	32.72	120.75	46
		2005	3,944	1,721	6,988	39	69.76	30.44	123.62	46
		2006	3,907	1,607	7,198	38	68.71	28.26	126.59	45
		2007	3,788	1,453	7,242	40	66.63	25.56	127.40	46

Metropolitan Statistical Area	PWID Population	Year	Number	Min	Max	Rank	Rate	Min	Max	Rank
Norfolk--Virginia Beach--Newport News, VA--NC	Young (15-29)	1992	997	739	1,222	80	25.62	18.98	31.41	100
		1993	1,143	755	1,607	77	29.87	19.74	42.02	96
		1994	1,572	1,143	2,030	69	42.18	30.66	54.46	85
		1995	1,668	954	2,502	61	45.49	26.03	68.23	81
		1996	1,962	1,051	3,008	53	54.13	28.99	82.98	68
		1997	2,212	1,134	3,324	49	61.62	31.60	92.61	62
		1998	2,390	1,199	3,467	46	67.42	33.84	97.81	61
		1999	2,996	2,267	3,553	37	84.49	63.93	100.18	54
		2000	2,670	1,333	3,530	45	74.85	37.38	98.98	59
		2001	3,150	2,248	3,848	38	88.71	63.31	108.37	58
		2002	2,728	1,338	4,168	46	75.62	37.10	115.54	65
		2003	2,746	1,313	4,442	47	75.62	36.14	122.33	66
		2004	2,752	1,266	4,674	51	74.02	34.06	125.71	73
		2005	2,704	1,180	4,793	54	72.44	31.61	128.38	76
		2006	2,639	1,085	4,862	55	69.14	28.44	127.37	78
		2007	2,497	958	4,774	58	65.92	25.28	126.03	82
	Old (30-64)	1992	5,359	3,970	6,569	59	86.01	63.72	105.44	75
		1993	5,648	3,733	7,945	54	87.91	58.11	123.67	68
		1994	7,047	5,123	9,098	48	107.26	77.97	138.47	57
		1995	6,753	3,864	10,130	43	101.12	57.87	151.68	55
		1996	7,191	3,851	11,023	42	106.11	56.82	162.66	48
		1997	7,372	3,781	11,079	39	107.61	55.20	161.74	48
		1998	7,296	3,662	10,584	40	106.12	53.26	153.95	46
		1999	8,451	6,394	10,020	38	121.29	91.77	143.82	38
		2000	7,025	3,508	9,290	41	99.25	49.56	131.25	51
		2001	7,828	5,587	9,563	39	109.45	78.11	133.71	44
		2002	6,499	3,188	9,929	41	89.77	44.04	137.15	54
		2003	6,387	3,053	10,333	41	86.96	41.56	140.67	53
		2004	6,394	2,942	10,859	41	86.23	39.68	146.44	53
		2005	6,449	2,814	11,428	40	86.57	37.78	153.42	47
		2006	6,658	2,738	12,265	36	88.94	36.58	163.85	45
		2007	6,883	2,640	13,160	34	91.91	35.25	175.73	36

Metropolitan Statistical Area	PWID Population	Year	Number	Min	Max	Rank	Rate	Min	Max	Rank
Oakland, CA	Total	1992	23,412	21,305	27,398	16	159.98	145.58	187.21	27
		1993	27,443	21,040	40,852	11	186.91	143.31	278.25	16
		1994	22,531	20,609	25,377	16	153.25	140.17	172.61	28
		1995	25,835	20,327	36,420	13	175.02	137.70	246.72	17
		1996	25,038	20,313	34,340	14	168.01	136.30	230.43	18
		1997	24,558	21,113	32,674	14	160.88	138.32	214.05	17
		1998	24,036	18,592	30,987	14	153.33	118.60	197.67	19
		1999	21,638	17,252	24,099	15	135.12	107.73	150.49	28
		2000	23,225	18,694	26,289	13	141.99	114.29	160.73	22
		2001	23,006	21,837	25,332	14	137.97	130.96	151.92	26
		2002	22,385	19,457	25,502	17	133.98	116.46	152.64	26
		2003	21,120	17,237	25,630	17	126.37	103.14	153.36	28
		2004	19,625	14,232	25,761	18	117.39	85.13	154.09	30
		2005	18,655	11,216	25,970	18	111.21	66.86	154.82	33
		2006	18,714	8,192	27,559	19	111.02	48.60	163.50	31
		2007	19,026	5,233	32,754	19	111.83	30.76	192.51	31
	Non-Hispanic White	1992	9,852	8,965	11,529	23	117.30	106.75	137.27	28
		1993	11,404	8,744	16,977	18	138.18	105.94	205.70	21
		1994	9,149	8,369	10,305	24	112.94	103.30	127.20	27
		1995	10,168	8,000	14,334	19	127.42	100.25	179.62	21
		1996	9,497	7,705	13,025	21	120.01	97.36	164.60	22
		1997	8,950	7,695	11,908	24	112.22	96.48	149.31	24
		1998	8,416	6,510	10,850	24	104.30	80.68	134.46	27
		1999	7,302	5,822	8,133	26	89.98	71.74	100.21	40
		2000	7,605	6,121	8,608	27	93.17	74.99	105.46	37
		2001	7,385	7,010	8,131	25	90.32	85.73	99.44	39
		2002	7,143	6,209	8,138	26	88.66	77.06	101.00	39
		2003	6,815	5,562	8,271	28	85.76	69.99	104.07	39
		2004	6,532	4,737	8,574	31	83.36	60.45	109.42	43
		2005	6,541	3,933	9,107	31	84.50	50.80	117.63	43
		2006	7,062	3,091	10,399	29	92.15	40.34	135.71	41
		2007	7,876	2,166	13,559	27	103.31	28.41	177.84	32

Metropolitan Statistical Area	PWID Population	Year	Number	Min	Max	Rank	Rate	Min	Max	Rank
Oakland, CA	Non-Hispanic Black	1992	8,032	7,309	9,399	14	395.33	359.75	462.62	31
		1993	9,811	7,522	14,605	11	485.94	372.57	723.39	13
		1994	8,353	7,640	9,408	12	416.61	381.06	469.23	19
		1995	9,884	7,776	13,933	9	495.29	389.69	698.22	11
		1996	9,836	7,980	13,490	9	492.72	399.74	675.78	9
		1997	9,856	8,473	13,113	9	487.82	419.38	649.02	9
		1998	9,804	7,583	12,639	9	479.24	370.69	617.84	7
		1999	8,921	7,113	9,935	9	432.39	344.75	481.57	6
		2000	9,623	7,745	10,892	9	464.02	373.50	525.25	7
		2001	9,522	9,038	10,484	9	460.50	437.11	507.05	6
		2002	9,196	7,993	10,477	11	450.13	391.26	512.81	6
		2003	8,554	6,981	10,381	11	421.78	344.24	511.86	7
		2004	7,782	5,643	10,214	11	385.20	279.34	505.62	8
		2005	7,188	4,322	10,007	13	355.45	213.71	494.83	11
		2006	6,956	3,045	10,244	14	342.88	150.09	504.95	12
		2007	6,775	1,863	11,663	14	332.40	91.42	572.23	13
	Hispanic	1992	3,910	3,558	4,575	20	191.41	174.18	223.99	35
		1993	4,232	3,244	6,299	18	198.68	152.32	295.76	32
		1994	3,293	3,012	3,709	25	148.77	136.08	167.56	37
		1995	3,664	2,883	5,165	20	159.61	125.58	225.00	34
		1996	3,514	2,851	4,820	21	146.84	119.13	201.39	35
		1997	3,468	2,982	4,615	21	137.49	118.20	182.92	36
		1998	3,459	2,676	4,460	21	129.78	100.38	167.31	36
		1999	3,203	2,553	3,567	22	114.58	91.36	127.61	36
		2000	3,555	2,861	4,024	19	121.31	97.64	137.31	35
		2001	3,650	3,464	4,019	18	119.01	112.97	131.04	34
		2002	3,677	3,196	4,189	18	116.34	101.13	132.54	33
		2003	3,578	2,920	4,342	19	110.75	90.39	134.41	34
		2004	3,405	2,470	4,470	20	103.19	74.83	135.46	39
		2005	3,284	1,975	4,572	21	97.14	58.41	135.23	40
		2006	3,302	1,445	4,863	22	95.30	41.72	140.34	41
		2007	3,316	912	5,709	24	92.95	25.56	160.02	41

Metropolitan Statistical Area	PWID Population	Year	Number	Min	Max	Rank	Rate	Min	Max	Rank
Oakland, CA	Male	1992	13,973	12,716	16,352	21	190.85	173.67	223.33	35
		1993	16,660	12,773	24,800	12	227.15	174.16	338.14	23
		1994	13,842	12,661	15,591	19	188.64	172.55	212.47	32
		1995	15,996	12,586	22,550	14	217.40	171.04	306.47	21
		1996	15,572	12,634	21,358	14	209.85	170.25	287.82	21
		1997	15,303	13,156	20,360	14	202.01	173.67	268.77	23
		1998	14,978	11,585	19,310	14	192.76	149.10	248.51	25
		1999	13,468	10,738	15,000	15	169.68	135.29	188.99	34
		2000	14,430	11,615	16,334	14	177.83	143.14	201.29	28
		2001	14,272	13,547	15,714	15	172.35	163.59	189.77	29
		2002	13,875	12,061	15,807	16	167.14	145.28	190.42	26
		2003	13,100	10,692	15,898	18	157.76	128.76	191.45	31
		2004	12,208	8,853	16,025	18	146.82	106.47	192.73	37
		2005	11,670	7,016	16,246	19	139.70	83.99	194.48	37
		2006	11,813	5,171	17,396	19	140.65	61.57	207.14	34
		2007	12,166	3,346	20,943	19	143.32	39.42	246.72	33
	Female	1992	9,406	8,559	11,007	13	128.62	117.04	150.51	20
		1993	10,774	8,261	16,039	9	146.63	112.42	218.28	13
		1994	8,704	7,961	9,803	15	118.18	108.10	133.11	19
		1995	9,876	7,771	13,923	11	133.40	104.96	188.05	13
		1996	9,519	7,722	13,055	12	127.22	103.21	174.49	14
		1997	9,320	8,013	12,400	14	121.21	104.20	161.26	14
		1998	9,130	7,062	11,771	14	115.49	89.33	148.89	13
		1999	8,240	6,570	9,178	15	102.02	81.34	113.62	23
		2000	8,871	7,140	10,042	16	107.64	86.64	121.84	16
		2001	8,809	8,361	9,699	13	104.95	99.62	115.56	22
		2002	8,577	7,455	9,771	15	102.03	88.69	116.24	19
		2003	8,075	6,590	9,799	16	96.03	78.38	116.54	23
		2004	7,458	5,409	9,790	17	88.75	64.36	116.50	25
		2005	7,011	4,215	9,760	20	83.26	50.06	115.91	28
		2006	6,913	3,026	10,180	20	81.73	35.78	120.36	33
		2007	6,856	1,885	11,802	20	80.41	22.12	138.43	32

Metropolitan Statistical Area	PWID Population	Year	Number	Min	Max	Rank	Rate	Min	Max	Rank
Oakland, CA	Young (15-29)	1992	4,206	3,828	4,922	28	89.29	81.25	104.48	51
		1993	4,810	3,688	7,161	23	104.24	79.92	155.18	38
		1994	3,842	3,514	4,327	27	84.84	77.60	95.55	52
		1995	4,280	3,368	6,034	23	95.43	75.08	134.52	45
		1996	4,031	3,270	5,528	23	89.91	72.95	123.32	48
		1997	3,849	3,309	5,121	24	84.67	72.79	112.65	52
		1998	3,680	2,847	4,744	28	79.24	61.29	102.16	56
		1999	3,254	2,594	3,624	33	68.77	54.83	76.59	64
		2000	3,455	2,781	3,911	34	71.52	57.57	80.95	63
		2001	3,415	3,242	3,760	35	69.85	66.31	76.92	68
		2002	3,352	2,914	3,819	36	69.39	60.32	79.06	69
		2003	3,230	2,636	3,919	40	67.66	55.22	82.11	75
		2004	3,108	2,254	4,080	42	65.51	47.51	85.99	78
		2005	3,107	1,868	4,326	43	65.24	39.23	90.83	79
		2006	3,333	1,459	4,909	40	69.43	30.39	102.25	77
		2007	3,687	1,014	6,348	38	75.39	20.73	129.79	67
	Old (30-64)	1992	19,452	17,701	22,763	12	196.01	178.37	229.38	21
		1993	22,862	17,529	34,034	11	227.09	174.11	338.05	13
		1994	18,858	17,249	21,240	14	185.37	169.55	208.78	20
		1995	21,751	17,113	30,663	11	211.66	166.53	298.38	11
		1996	21,215	17,212	29,097	12	203.61	165.18	279.25	9
		1997	20,938	18,001	27,857	13	195.33	167.93	259.89	11
		1998	20,602	15,936	26,561	13	186.75	144.45	240.76	13
		1999	18,620	14,846	20,739	13	165.05	131.59	183.82	20
		2000	20,029	16,122	22,672	12	173.78	139.87	196.70	13
		2001	19,840	18,832	21,846	13	168.35	159.80	185.37	13
		2002	19,253	16,736	21,935	12	162.11	140.91	184.68	14
		2003	18,060	14,740	21,917	15	151.27	123.46	183.58	17
		2004	16,618	12,051	21,813	17	138.79	100.65	182.18	18
		2005	15,559	9,355	21,660	17	129.53	77.88	180.33	20
		2006	15,260	6,680	22,473	17	126.59	55.41	186.42	18
		2007	15,005	4,127	25,832	15	123.77	34.04	213.08	16

Metropolitan Statistical Area	PWID Population	Year	Number	Min	Max	Rank	Rate	Min	Max	Rank
Oklahoma City, OK	Total	1992	6,950	5,714	8,073	61	106.19	87.31	123.35	50
		1993	7,146	5,943	8,699	59	107.29	89.22	130.61	48
		1994	6,298	6,172	6,522	67	93.17	91.30	96.48	57
		1995	6,433	5,263	7,866	65	94.14	77.02	115.13	52
		1996	6,180	4,634	7,483	65	89.05	66.78	107.83	56
		1997	5,972	4,184	7,090	66	84.79	59.40	100.65	59
		1998	5,787	3,872	7,155	67	81.15	54.30	100.35	60
		1999	5,467	3,715	7,449	74	75.35	51.21	102.66	65
		2000	5,638	3,806	7,795	68	76.84	51.87	106.23	65
		2001	5,699	4,176	8,149	75	76.79	56.27	109.80	64
		2002	5,865	4,553	8,738	69	78.13	60.66	116.42	64
		2003	6,017	4,339	9,327	69	79.38	57.24	123.04	61
		2004	6,110	4,123	9,914	70	80.05	54.02	129.89	59
		2005	6,113	3,932	10,550	69	79.24	50.97	136.75	60
		2006	6,128	3,760	11,241	71	78.34	48.07	143.71	61
		2007	6,114	3,575	11,920	72	77.29	45.19	150.68	61
	Non-Hispanic White	1992	5,532	4,548	6,426	44	106.42	87.50	123.61	36
		1993	5,710	4,749	6,951	38	108.49	90.22	132.07	33
		1994	5,040	4,939	5,219	45	94.86	92.96	98.24	42
		1995	5,145	4,209	6,292	39	96.29	78.78	117.75	35
		1996	4,930	3,697	5,970	39	91.43	68.56	110.71	42
		1997	4,743	3,323	5,631	43	87.32	61.17	103.65	45
		1998	4,567	3,056	5,647	42	83.72	56.02	103.52	46
		1999	4,280	2,909	5,831	49	77.85	52.90	106.06	54
		2000	4,370	2,950	6,042	45	79.15	53.43	109.43	47
		2001	4,364	3,198	6,241	51	78.79	57.74	112.66	53
		2002	4,428	3,438	6,599	44	79.56	61.77	118.54	46
		2003	4,471	3,224	6,930	44	79.97	57.67	123.96	45
		2004	4,457	3,008	7,232	45	79.56	53.69	129.10	46
		2005	4,369	2,810	7,540	53	77.60	49.91	133.90	48
		2006	4,281	2,627	7,854	56	75.39	46.27	138.31	53
		2007	4,166	2,436	8,123	58	73.07	42.73	142.46	55

Metropolitan Statistical Area	PWID Population	Year	Number	Min	Max	Rank	Rate	Min	Max	Rank
Oklahoma City, OK	Non-Hispanic Black	1992	669	550	778	87	100.44	82.58	116.67	96
		1993	638	531	777	87	93.79	78.00	114.18	95
		1994	525	515	544	89	75.59	74.07	78.28	99
		1995	505	414	618	89	71.80	58.74	87.80	99
		1996	461	346	558	89	64.28	48.20	77.84	99
		1997	427	299	507	89	58.24	40.80	69.13	99
		1998	401	268	496	91	53.44	35.75	66.07	99
		1999	371	252	505	92	48.17	32.73	65.63	99
		2000	378	255	523	91	48.15	32.51	66.57	99
		2001	383	281	548	92	47.54	34.84	67.98	99
		2002	399	310	595	87	48.77	37.87	72.67	96
		2003	421	303	652	86	50.79	36.63	78.73	94
		2004	444	300	721	86	53.49	36.09	86.80	94
		2005	469	302	810	86	55.44	35.66	95.67	93
		2006	504	309	925	84	58.41	35.84	107.15	92
		2007	549	321	1,070	81	61.87	36.18	120.63	89
	Hispanic	1992	130	107	151	73	52.66	43.29	61.17	85
		1993	145	120	176	72	54.18	45.06	65.96	79
		1994	132	129	137	73	45.78	44.87	47.41	85
		1995	135	111	166	72	43.58	35.66	53.29	82
		1996	127	96	154	74	37.62	28.21	45.55	85
		1997	118	83	141	75	31.98	22.40	37.96	87
		1998	109	73	135	76	27.05	18.10	33.45	91
		1999	98	67	134	80	22.12	15.03	30.14	91
		2000	97	65	134	79	20.42	13.78	28.23	91
		2001	95	70	136	82	18.73	13.73	26.78	93
		2002	97	75	144	80	18.03	13.99	26.86	92
		2003	101	73	157	80	18.03	13.00	27.94	93
		2004	109	74	177	80	18.53	12.50	30.06	93
		2005	122	78	210	80	19.61	12.61	33.83	93
		2006	143	88	263	78	21.97	13.48	40.31	89
		2007	179	105	349	76	26.20	15.32	51.08	84

Metropolitan Statistical Area	PWID Population	Year	Number	Min	Max	Rank	Rate	Min	Max	Rank
Oklahoma City, OK	Male	1992	4,581	3,767	5,321	60	141.48	116.32	164.33	49
		1993	4,635	3,854	5,642	56	140.41	116.76	170.92	47
		1994	4,026	3,945	4,169	69	120.12	117.71	124.39	58
		1995	4,058	3,320	4,963	66	119.71	97.95	146.40	54
		1996	3,852	2,888	4,664	66	111.82	83.84	135.39	59
		1997	3,681	2,579	4,370	67	105.28	73.75	124.98	59
		1998	3,528	2,361	4,362	71	99.67	66.69	123.24	63
		1999	3,297	2,240	4,492	79	91.47	62.16	124.63	73
		2000	3,361	2,269	4,648	73	92.24	62.27	127.53	67
		2001	3,356	2,459	4,799	79	90.86	66.59	129.92	68
		2002	3,406	2,644	5,075	72	91.10	70.73	135.74	67
		2003	3,440	2,480	5,332	73	91.29	65.83	141.51	66
		2004	3,428	2,313	5,563	73	90.15	60.83	146.28	66
		2005	3,356	2,159	5,792	74	87.38	56.20	150.78	66
		2006	3,278	2,012	6,014	76	84.19	51.67	154.46	68
		2007	3,171	1,854	6,183	78	80.40	47.01	156.74	71
	Female	1992	2,442	2,008	2,837	62	73.85	60.72	85.78	46
		1993	2,585	2,150	3,147	55	76.95	63.99	93.68	41
		1994	2,340	2,293	2,423	65	68.65	67.28	71.10	55
		1995	2,449	2,004	2,995	57	71.14	58.20	87.00	48
		1996	2,407	1,805	2,914	60	68.88	51.64	83.40	54
		1997	2,375	1,664	2,819	61	66.95	46.90	79.48	54
		1998	2,347	1,570	2,901	62	65.35	43.73	80.80	55
		1999	2,258	1,534	3,076	67	61.85	42.03	84.26	59
		2000	2,370	1,600	3,276	62	64.16	43.31	88.70	56
		2001	2,435	1,785	3,483	63	65.33	47.88	93.42	56
		2002	2,547	1,978	3,796	58	67.62	52.50	100.76	53
		2003	2,656	1,915	4,116	57	69.65	50.23	107.96	48
		2004	2,740	1,849	4,446	55	71.55	48.28	116.10	45
		2005	2,786	1,792	4,807	56	71.93	46.26	124.12	43
		2006	2,839	1,742	5,207	53	72.26	44.34	132.56	41
		2007	2,881	1,684	5,616	53	72.63	42.47	141.60	42

Metropolitan Statistical Area	PWID Population	Year	Number	Min	Max	Rank	Rate	Min	Max	Rank
Oklahoma City, OK	Young (15-29)	1992	2,338	1,922	2,715	49	102.28	84.09	118.81	39
		1993	2,458	2,044	2,992	42	106.74	88.76	129.94	34
		1994	2,211	2,166	2,289	48	95.36	93.44	98.75	40
		1995	2,298	1,880	2,811	44	98.09	80.25	119.95	44
		1996	2,242	1,681	2,715	46	94.06	70.53	113.89	45
		1997	2,195	1,537	2,605	51	90.28	63.24	107.17	46
		1998	2,148	1,437	2,656	54	86.91	58.15	107.46	52
		1999	2,045	1,390	2,786	59	81.42	55.33	110.93	55
		2000	2,120	1,431	2,931	57	83.99	56.70	116.13	55
		2001	2,147	1,574	3,070	62	84.33	61.80	120.59	62
		2002	2,208	1,714	3,290	57	85.96	66.74	128.09	55
		2003	2,257	1,628	3,499	61	87.22	62.90	135.20	59
		2004	2,277	1,536	3,695	64	87.59	59.11	142.13	57
		2005	2,256	1,451	3,894	64	86.06	55.35	148.50	57
		2006	2,233	1,370	4,096	66	83.93	51.50	153.97	61
		2007	2,192	1,282	4,273	66	81.74	47.79	159.36	60
	Old (30-64)	1992	4,725	3,885	5,488	65	110.92	91.20	128.85	53
		1993	4,760	3,958	5,794	62	109.23	90.84	132.97	48
		1994	4,137	4,055	4,285	73	93.15	91.28	96.47	68
		1995	4,193	3,430	5,127	69	93.38	76.40	114.20	62
		1996	4,015	3,010	4,861	72	88.12	66.08	106.70	64
		1997	3,879	2,717	4,605	75	84.09	58.91	99.82	65
		1998	3,764	2,518	4,654	74	80.79	54.06	99.89	64
		1999	3,561	2,420	4,852	77	75.07	51.02	102.28	75
		2000	3,673	2,479	5,078	75	76.29	51.50	105.48	62
		2001	3,702	2,713	5,294	77	75.94	55.65	108.59	67
		2002	3,784	2,938	5,639	72	76.64	59.51	114.20	59
		2003	3,834	2,765	5,944	72	76.81	55.39	119.05	57
		2004	3,816	2,575	6,192	71	75.83	51.17	123.04	58
		2005	3,706	2,384	6,395	71	72.77	46.81	125.57	60
		2006	3,558	2,183	6,527	73	68.92	42.30	126.44	61
		2007	3,341	1,954	6,515	73	63.90	37.36	124.58	63

Metropolitan Statistical Area	PWID Population	Year	Number	Min	Max	Rank	Rate	Min	Max	Rank
Omaha, NE--IA	Total	1992	2,491	1,703	3,358	96	57.62	39.39	77.66	91
		1993	2,502	1,672	3,331	92	57.56	38.46	76.63	84
		1994	2,453	1,648	3,335	95	55.83	37.51	75.91	91
		1995	2,468	1,635	3,362	91	55.20	36.56	75.18	86
		1996	2,477	1,623	3,430	91	54.24	35.54	75.10	85
		1997	2,469	1,600	3,476	91	53.30	34.53	75.02	85
		1998	2,483	1,627	3,573	91	53.00	34.72	76.25	86
		1999	2,565	1,656	3,674	92	54.10	34.92	77.48	91
		2000	2,579	1,791	3,939	91	53.74	37.31	82.08	84
		2001	2,823	1,925	4,202	90	58.24	39.71	86.69	83
		2002	2,752	2,059	4,542	91	56.18	42.03	92.71	82
		2003	2,851	2,002	4,889	91	57.57	40.42	98.73	80
		2004	2,943	1,930	5,249	91	58.64	38.45	104.58	78
		2005	3,038	1,848	5,623	91	59.66	36.29	110.43	79
		2006	3,123	1,759	5,987	91	60.61	34.13	116.21	77
		2007	3,200	1,650	6,348	92	61.49	31.71	121.99	74
	Non-Hispanic White	1992	1,671	1,143	2,252	91	43.99	30.08	59.29	83
		1993	1,722	1,150	2,292	85	45.30	30.27	60.30	75
		1994	1,720	1,155	2,338	87	45.02	30.25	61.22	80
		1995	1,754	1,161	2,389	85	45.39	30.06	61.82	74
		1996	1,776	1,164	2,460	86	45.30	29.68	62.73	74
		1997	1,782	1,154	2,508	86	45.03	29.17	63.39	74
		1998	1,799	1,178	2,588	86	45.16	29.58	64.97	75
		1999	1,861	1,201	2,665	85	46.44	29.97	66.51	74
		2000	1,872	1,300	2,859	85	46.42	32.23	70.90	75
		2001	2,047	1,396	3,048	84	50.53	34.45	75.21	72
		2002	1,994	1,492	3,291	85	48.92	36.60	80.73	75
		2003	2,064	1,449	3,539	86	50.29	35.31	86.24	74
		2004	2,130	1,397	3,799	83	51.37	33.68	91.61	71
		2005	2,200	1,338	4,072	83	52.46	31.91	97.10	72
		2006	2,265	1,276	4,344	82	53.59	30.18	102.75	74
		2007	2,330	1,202	4,623	83	54.74	28.23	108.61	75

Metropolitan Statistical Area	PWID Population	Year	Number	Min	Max	Rank	Rate	Min	Max	Rank
Omaha, NE--IA	Non-Hispanic Black	1992	599	410	808	89	184.14	125.89	248.19	81
		1993	551	368	734	89	167.72	112.06	223.26	80
		1994	494	332	671	91	146.17	98.21	198.75	86
		1995	454	300	618	91	131.10	86.83	178.57	86
		1996	416	272	576	91	116.64	76.43	161.51	88
		1997	379	246	534	93	103.49	67.04	145.67	89
		1998	350	229	504	93	93.80	61.45	134.95	88
		1999	334	215	478	93	87.75	56.64	125.67	91
		2000	311	216	475	94	80.23	55.71	122.54	91
		2001	319	217	474	93	80.82	55.11	120.30	91
		2002	293	220	484	94	73.80	55.21	121.79	91
		2003	290	204	498	94	72.17	50.67	123.77	88
		2004	290	190	517	94	70.93	46.51	126.50	88
		2005	294	179	544	94	70.92	43.14	131.27	87
		2006	302	170	580	93	71.91	40.50	137.87	86
		2007	316	163	628	94	74.49	38.41	147.78	84
	Hispanic	1992	72	49	97	80	57.18	39.09	77.06	81
		1993	72	48	95	80	51.52	34.42	68.58	80
		1994	71	48	96	83	46.18	31.03	62.79	84
		1995	74	49	100	80	43.03	28.50	58.60	83
		1996	77	51	107	80	40.63	26.62	56.26	82
		1997	81	53	114	80	39.41	25.52	55.46	80
		1998	87	57	125	80	38.91	25.49	55.98	80
		1999	96	62	137	81	39.42	25.45	56.46	78
		2000	103	71	157	77	39.36	27.33	60.11	76
		2001	119	81	177	78	42.86	29.22	63.80	73
		2002	122	91	201	75	41.45	31.01	68.40	71
		2003	131	92	225	75	42.47	29.82	72.84	68
		2004	139	91	247	76	43.14	28.29	76.94	69
		2005	144	88	267	76	42.63	25.93	78.90	69
		2006	146	82	280	76	41.40	23.32	79.39	68
		2007	144	74	286	81	39.31	20.27	77.99	72

Metropolitan Statistical Area	PWID Population	Year	Number	Min	Max	Rank	Rate	Min	Max	Rank
Omaha, NE--IA	Male	1992	1,836	1,255	2,474	92	86.05	58.83	115.98	88
		1993	1,775	1,186	2,363	91	82.69	55.25	110.08	83
		1994	1,679	1,128	2,283	94	77.27	51.92	105.07	88
		1995	1,636	1,083	2,228	92	73.92	48.96	100.68	87
		1996	1,595	1,045	2,209	93	70.49	46.19	97.61	87
		1997	1,552	1,005	2,185	92	67.55	43.75	95.08	88
		1998	1,530	1,002	2,201	92	65.78	43.10	94.64	89
		1999	1,556	1,004	2,228	92	66.01	42.61	94.53	89
		2000	1,546	1,074	2,362	92	64.74	44.95	98.88	88
		2001	1,679	1,145	2,499	92	69.51	47.40	103.46	87
		2002	1,630	1,219	2,690	89	66.71	49.91	110.09	86
		2003	1,685	1,183	2,890	89	68.28	47.94	117.10	85
		2004	1,741	1,142	3,105	89	69.47	45.55	123.90	85
		2005	1,801	1,096	3,334	89	70.77	43.05	130.99	82
		2006	1,857	1,046	3,561	91	72.04	40.57	138.13	82
		2007	1,910	985	3,790	91	73.38	37.84	145.58	78
	Female	1992	697	477	940	96	31.83	21.76	42.90	93
		1993	767	512	1,021	93	34.85	23.29	46.39	88
		1994	812	546	1,105	94	36.58	24.58	49.74	89
		1995	873	578	1,189	93	38.65	25.60	52.64	86
		1996	925	606	1,281	91	40.15	26.31	55.60	86
		1997	964	624	1,357	88	41.28	26.74	58.10	85
		1998	1,004	658	1,444	88	42.54	27.87	61.20	82
		1999	1,064	687	1,524	88	44.63	28.81	63.92	86
		2000	1,090	757	1,665	88	45.20	31.39	69.04	76
		2001	1,205	822	1,794	88	49.57	33.80	73.79	77
		2002	1,179	882	1,945	88	47.99	35.90	79.19	74
		2003	1,216	854	2,085	89	48.95	34.37	83.94	74
		2004	1,241	814	2,213	88	49.38	32.38	88.06	73
		2005	1,257	765	2,327	88	49.37	30.03	91.37	74
		2006	1,259	709	2,415	91	48.93	27.55	93.81	73
		2007	1,248	644	2,476	91	48.00	24.75	95.22	73

Metropolitan Statistical Area	PWID Population	Year	Number	Min	Max	Rank	Rate	Min	Max	Rank
Omaha, NE--IA	Young (15-29)	1992	737	504	993	92	49.94	34.14	67.31	79
		1993	750	501	998	87	51.45	34.37	68.48	73
		1994	752	505	1,022	88	51.67	34.71	70.25	74
		1995	779	516	1,061	86	52.88	35.02	72.03	67
		1996	808	529	1,118	87	53.70	35.19	74.36	69
		1997	832	539	1,171	88	54.44	35.26	76.63	68
		1998	863	565	1,242	88	55.84	36.58	80.33	68
		1999	915	591	1,311	87	58.60	37.83	83.93	73
		2000	938	651	1,433	88	59.72	41.47	91.21	72
		2001	1,036	707	1,542	87	65.85	44.90	98.02	73
		2002	1,008	754	1,663	89	63.60	47.58	104.96	76
		2003	1,026	720	1,759	92	64.08	44.99	109.89	78
		2004	1,023	671	1,824	92	63.16	41.41	112.65	80
		2005	998	607	1,847	94	60.70	36.92	112.36	84
		2006	945	532	1,811	96	56.79	31.98	108.89	92
		2007	865	446	1,716	98	51.34	26.47	101.85	94
	Old (30-64)	1992	1,833	1,253	2,471	96	64.37	44.01	86.76	91
		1993	1,796	1,200	2,391	94	62.17	41.54	82.76	89
		1994	1,727	1,160	2,348	96	58.77	39.49	79.91	91
		1995	1,712	1,134	2,332	94	57.12	37.83	77.80	93
		1996	1,701	1,114	2,355	95	55.52	36.38	76.88	93
		1997	1,683	1,090	2,368	95	54.20	35.11	76.29	92
		1998	1,682	1,102	2,419	93	53.56	35.09	77.06	93
		1999	1,726	1,114	2,473	95	54.29	35.05	77.76	88
		2000	1,723	1,197	2,632	92	53.37	37.06	81.52	91
		2001	1,867	1,273	2,779	92	57.04	38.89	84.90	84
		2002	1,794	1,342	2,961	89	54.14	40.50	89.34	82
		2003	1,821	1,279	3,123	89	54.35	38.16	93.20	80
		2004	1,827	1,198	3,259	89	53.74	35.24	95.84	77
		2005	1,813	1,103	3,355	92	52.56	31.97	97.29	78
		2006	1,764	994	3,383	92	50.57	28.48	96.96	80
		2007	1,678	865	3,330	92	47.69	24.59	94.61	81

Metropolitan Statistical Area	PWID Population	Year	Number	Min	Max	Rank	Rate	Min	Max	Rank
Orange County, CA	Total	1992	23,564	18,291	28,829	15	137.54	106.76	168.27	40
		1993	24,492	18,095	31,169	14	142.21	105.06	180.97	34
		1994	21,031	17,909	22,665	17	121.39	103.37	130.82	43
		1995	22,082	17,765	28,717	18	126.41	101.70	164.39	36
		1996	21,510	17,654	27,572	18	121.91	100.06	156.26	38
		1997	21,596	17,843	26,797	17	119.14	98.44	147.84	41
		1998	21,568	18,029	25,953	16	115.85	96.85	139.41	42
		1999	19,778	18,031	23,087	17	104.51	95.28	122.00	47
		2000	20,157	16,569	23,726	19	104.92	86.25	123.50	44
		2001	17,593	14,506	20,330	21	90.43	74.56	104.50	58
		2002	16,942	11,987	20,342	20	86.23	61.01	103.54	56
		2003	15,598	9,689	18,544	25	78.70	48.88	93.56	63
		2004	14,989	8,223	17,565	25	75.08	41.19	87.98	65
		2005	14,949	7,302	20,696	25	74.67	36.47	103.38	65
		2006	14,960	6,439	24,003	25	74.57	32.10	119.65	65
		2007	15,101	5,728	27,769	25	75.03	28.46	137.96	64
	Non-Hispanic White	1992	11,871	9,214	14,523	16	111.61	86.63	136.55	33
		1993	12,032	8,890	15,312	14	115.28	85.17	146.70	29
		1994	10,071	8,576	10,854	19	98.22	83.64	105.85	40
		1995	10,309	8,294	13,406	17	101.92	82.00	132.54	31
		1996	9,795	8,039	12,556	19	97.90	80.35	125.49	34
		1997	9,604	7,935	11,917	20	95.22	78.68	118.16	37
		1998	9,384	7,845	11,292	22	92.27	77.13	111.03	37
		1999	8,441	7,696	9,854	23	83.20	75.85	97.12	46
		2000	8,467	6,960	9,966	23	83.79	68.88	98.63	43
		2001	7,303	6,022	8,439	26	72.43	59.72	83.70	56
		2002	6,984	4,942	8,386	28	69.53	49.19	83.48	57
		2003	6,422	3,989	7,634	32	64.18	39.87	76.31	62
		2004	6,201	3,402	7,267	33	62.30	34.18	73.01	64
		2005	6,256	3,056	8,661	32	63.46	30.99	87.85	63
		2006	6,376	2,744	10,230	33	65.26	28.09	104.70	61
		2007	6,600	2,504	12,137	32	68.18	25.86	125.38	62

Metropolitan Statistical Area	PWID Population	Year	Number	Min	Max	Rank	Rate	Min	Max	Rank
Orange County, CA	Non-Hispanic Black	1992	599	465	733	89	203.25	157.76	248.66	74
		1993	670	495	852	86	224.84	166.11	286.12	55
		1994	598	509	645	86	198.06	168.66	213.45	72
		1995	635	511	826	86	207.69	167.09	270.09	55
		1996	612	502	784	86	196.68	161.43	252.11	55
		1997	597	493	741	87	185.95	153.64	230.74	58
		1998	573	479	690	85	173.69	145.19	209.01	59
		1999	503	458	587	87	150.61	137.31	175.81	72
		2000	490	403	577	86	144.89	119.10	170.54	67
		2001	411	339	475	90	118.23	97.48	136.62	76
		2002	385	273	462	88	108.20	76.56	129.91	75
		2003	351	218	417	91	96.40	59.88	114.61	78
		2004	341	187	399	91	91.63	50.27	107.38	78
		2005	354	173	490	91	93.69	45.76	129.71	75
		2006	383	165	614	89	99.87	42.99	160.24	72
		2007	436	165	802	87	113.04	42.88	207.86	67
	Hispanic	1992	8,186	6,354	10,015	8	197.26	153.11	241.33	33
		1993	8,562	6,326	10,896	6	198.51	146.66	252.62	33
		1994	7,408	6,308	7,984	7	165.44	140.88	178.30	34
		1995	7,844	6,310	10,200	6	169.13	136.06	219.94	33
		1996	7,705	6,324	9,876	6	160.28	131.55	205.45	32
		1997	7,797	6,442	9,675	6	154.47	127.62	191.67	33
		1998	7,841	6,554	9,435	7	147.94	123.67	178.02	33
		1999	7,228	6,590	8,438	7	131.48	119.87	153.48	33
		2000	7,391	6,075	8,699	6	129.70	106.61	152.66	32
		2001	6,455	5,323	7,460	12	110.19	90.85	127.33	38
		2002	6,202	4,388	7,447	9	103.53	73.26	124.31	41
		2003	5,677	3,527	6,750	12	92.95	57.74	110.50	44
		2004	5,404	2,964	6,332	13	87.02	47.74	101.97	44
		2005	5,314	2,596	7,357	14	84.58	41.31	117.09	44
		2006	5,219	2,246	8,374	14	82.27	35.41	131.99	46
		2007	5,144	1,951	9,459	14	79.85	30.29	146.84	47

Metropolitan Statistical Area	PWID Population	Year	Number	Min	Max	Rank	Rate	Min	Max	Rank
Orange County, CA	Male	1992	14,664	11,382	17,940	19	166.66	129.36	203.89	42
		1993	15,576	11,508	19,822	14	176.35	130.29	224.42	35
		1994	13,612	11,591	14,670	20	153.63	130.82	165.56	42
		1995	14,496	11,662	18,851	15	162.59	130.80	211.44	37
		1996	14,279	11,720	18,304	15	158.89	130.41	203.67	39
		1997	14,462	11,949	17,945	15	157.02	129.73	194.83	39
		1998	14,540	12,155	17,497	15	154.07	128.80	185.40	40
		1999	13,400	12,217	15,642	16	140.06	127.69	163.50	41
		2000	13,707	11,267	16,133	16	141.22	116.08	166.22	41
		2001	11,994	9,890	13,860	20	121.92	100.53	140.89	54
		2002	11,572	8,188	13,894	19	116.40	82.36	139.76	53
		2003	10,668	6,627	12,683	21	106.36	66.07	126.45	57
		2004	10,265	5,631	12,029	21	101.51	55.69	118.96	59
		2005	10,251	5,007	14,191	22	100.99	49.33	139.82	59
		2006	10,276	4,423	16,488	21	100.97	43.46	162.00	59
		2007	10,398	3,944	19,120	22	101.70	38.58	187.01	58
	Female	1992	8,524	6,616	10,428	16	102.29	79.40	125.14	35
		1993	8,724	6,445	11,102	16	103.97	76.82	132.31	27
		1994	7,386	6,289	7,960	18	87.26	74.30	94.04	38
		1995	7,655	6,158	9,955	18	89.50	72.00	116.39	34
		1996	7,368	6,047	9,445	19	85.10	69.85	109.09	38
		1997	7,317	6,045	9,079	19	82.07	67.81	101.84	42
		1998	7,234	6,047	8,705	20	78.81	65.88	94.83	44
		1999	6,572	5,991	7,671	21	70.23	64.03	81.98	52
		2000	6,639	5,457	7,814	20	69.84	57.41	82.21	53
		2001	5,746	4,738	6,640	22	59.74	49.26	69.04	62
		2002	5,489	3,884	6,590	26	56.55	40.01	67.90	64
		2003	5,013	3,114	5,960	27	51.21	31.81	60.89	72
		2004	4,780	2,622	5,602	32	48.52	26.62	56.86	75
		2005	4,730	2,310	6,548	33	47.92	23.41	66.35	78
		2006	4,696	2,021	7,534	33	47.51	20.45	76.23	75
		2007	4,701	1,783	8,645	33	47.47	18.01	87.29	75

Metropolitan Statistical Area	PWID Population	Year	Number	Min	Max	Rank	Rate	Min	Max	Rank
Orange County, CA	Young (15-29)	1992	7,895	6,128	9,659	11	128.38	99.65	157.06	27
		1993	7,698	5,688	9,797	11	127.91	94.50	162.78	25
		1994	6,292	5,358	6,781	15	106.52	90.71	114.80	34
		1995	6,376	5,129	8,291	12	109.18	87.84	141.98	33
		1996	6,070	4,982	7,781	13	104.44	85.72	133.87	38
		1997	6,024	4,977	7,475	13	102.06	84.32	126.64	40
		1998	6,005	5,019	7,226	13	100.03	83.62	120.37	41
		1999	5,539	5,050	6,466	17	91.77	83.66	107.12	45
		2000	5,714	4,697	6,725	16	93.76	77.07	110.35	48
		2001	5,068	4,179	5,857	19	83.60	68.93	96.61	63
		2002	4,972	3,518	5,970	24	82.42	58.32	98.96	62
		2003	4,666	2,899	5,548	24	77.35	48.05	91.96	65
		2004	4,566	2,505	5,350	29	75.49	41.41	88.46	68
		2005	4,623	2,258	6,400	27	76.25	37.24	105.56	71
		2006	4,675	2,012	7,501	26	76.75	33.03	123.14	66
		2007	4,740	1,798	8,716	27	77.04	29.22	141.66	65
	Old (30-64)	1992	15,907	12,347	19,461	18	144.84	112.42	177.20	41
		1993	16,948	12,521	21,568	16	151.26	111.75	192.49	33
		1994	14,842	12,639	15,995	19	129.99	110.69	140.09	43
		1995	15,825	12,731	20,580	16	136.09	109.48	176.97	37
		1996	15,592	12,797	19,986	17	131.77	108.15	168.91	37
		1997	15,776	13,034	19,575	16	129.06	106.63	160.14	38
		1998	15,820	13,225	19,037	16	125.42	104.85	150.92	39
		1999	14,516	13,234	16,945	17	112.63	102.69	131.48	45
		2000	14,753	12,127	17,365	18	112.47	92.45	132.38	41
		2001	12,796	10,551	14,787	22	95.54	78.78	110.41	53
		2002	12,204	8,635	14,654	21	89.64	63.43	107.64	55
		2003	11,089	6,888	13,184	24	80.43	49.96	95.62	56
		2004	10,480	5,749	12,280	25	75.31	41.31	88.25	60
		2005	10,236	5,000	14,171	26	73.34	35.82	101.53	59
		2006	9,986	4,298	16,022	26	71.49	30.77	114.70	57
		2007	9,771	3,706	17,967	26	69.92	26.52	128.57	56

Metropolitan Statistical Area	PWID Population	Year	Number	Min	Max	Rank	Rate	Min	Max	Rank
Orlando, FL	Total	1992	6,549	3,406	8,174	66	75.00	39.00	93.60	76
		1993	6,092	3,128	8,770	66	68.12	34.98	98.06	80
		1994	7,611	5,483	9,418	57	82.96	59.76	102.65	69
		1995	6,937	3,284	10,087	59	73.75	34.92	107.24	71
		1996	7,278	3,387	10,843	56	75.15	34.97	111.96	69
		1997	7,651	3,514	11,729	49	76.11	34.96	116.68	66
		1998	8,134	3,636	12,667	48	78.18	34.95	121.75	65
		1999	10,296	7,816	13,579	41	96.18	73.01	126.83	54
		2000	9,232	3,832	14,689	43	83.31	34.58	132.55	60
		2001	11,692	7,816	15,841	35	102.06	68.23	138.27	49
		2002	9,959	3,674	16,944	41	84.40	31.14	143.61	57
		2003	9,977	3,567	18,061	41	82.27	29.41	148.95	60
		2004	10,026	3,476	19,378	41	79.84	27.68	154.31	60
		2005	10,138	3,346	20,843	42	77.66	25.63	159.67	63
		2006	10,140	3,170	22,162	41	75.46	23.59	164.93	64
		2007	9,996	2,894	23,214	41	73.28	21.21	170.19	66
	Non-Hispanic White	1992	4,022	2,091	5,019	57	60.43	31.42	75.42	60
		1993	3,547	1,822	5,106	60	52.93	27.18	76.20	63
		1994	4,237	3,052	5,243	55	62.61	45.10	77.47	59
		1995	3,726	1,764	5,418	58	54.66	25.88	79.48	61
		1996	3,808	1,772	5,673	56	55.27	25.72	82.34	64
		1997	3,936	1,808	6,034	52	56.07	25.76	85.96	63
		1998	4,153	1,856	6,467	49	58.22	26.03	90.67	62
		1999	5,262	3,994	6,939	39	73.15	55.53	96.46	55
		2000	4,760	1,976	7,573	39	65.18	27.06	103.71	59
		2001	6,123	4,093	8,295	36	82.32	55.03	111.53	46
		2002	5,328	1,965	9,064	39	70.59	26.04	120.11	56
		2003	5,477	1,958	9,915	39	71.75	25.65	129.90	55
		2004	5,666	1,964	10,951	38	72.97	25.30	141.04	52
		2005	5,911	1,951	12,152	36	74.79	24.69	153.76	51
		2006	6,104	1,908	13,341	34	76.68	23.97	167.59	49
		2007	6,212	1,798	14,427	36	78.14	22.62	181.48	47

Metropolitan Statistical Area	PWID Population	Year	Number	Min	Max	Rank	Rate	Min	Max	Rank
Orlando, FL	Non-Hispanic Black	1992	1,134	590	1,415	74	112.13	58.31	139.93	95
		1993	1,150	591	1,656	69	108.16	55.54	155.70	94
		1994	1,514	1,091	1,874	61	135.67	97.73	167.86	88
		1995	1,413	669	2,055	62	120.97	57.28	175.91	89
		1996	1,484	691	2,211	59	121.21	56.41	180.58	86
		1997	1,534	705	2,352	56	118.24	54.31	181.26	82
		1998	1,583	708	2,465	55	115.63	51.69	180.08	81
		1999	1,929	1,464	2,543	45	134.78	102.31	177.75	75
		2000	1,659	689	2,640	53	110.63	45.92	176.02	79
		2001	2,020	1,350	2,736	40	129.18	86.36	175.01	73
		2002	1,666	614	2,834	47	102.45	37.79	174.31	77
		2003	1,637	585	2,963	45	96.73	34.58	175.11	77
		2004	1,644	570	3,177	44	92.70	32.14	179.16	76
		2005	1,702	562	3,498	42	91.06	30.05	187.20	77
		2006	1,794	561	3,922	39	91.81	28.70	200.66	77
		2007	1,929	559	4,481	37	96.19	27.85	223.38	76
	Hispanic	1992	1,342	698	1,675	38	157.56	81.93	196.63	43
		1993	1,167	599	1,679	38	123.80	63.57	178.21	46
		1994	1,424	1,026	1,762	38	137.77	99.24	170.47	41
		1995	1,316	623	1,914	38	115.45	54.66	167.89	44
		1996	1,443	672	2,150	38	114.02	53.06	169.87	42
		1997	1,619	743	2,481	37	115.46	53.03	177.00	39
		1998	1,859	831	2,896	34	120.57	53.90	187.77	38
		1999	2,556	1,940	3,371	27	150.81	114.48	198.89	28
		2000	2,482	1,030	3,949	28	132.92	55.18	211.50	31
		2001	3,375	2,256	4,572	19	166.95	111.61	226.19	20
		2002	3,041	1,122	5,175	25	140.25	51.74	238.63	23
		2003	3,161	1,130	5,723	23	136.31	48.73	246.77	24
		2004	3,215	1,115	6,215	22	128.30	44.48	247.96	25
		2005	3,192	1,054	6,563	23	116.90	38.58	240.33	29
		2006	3,019	944	6,599	25	102.70	32.10	224.46	37
		2007	2,686	778	6,239	26	87.18	25.24	202.45	43

Metropolitan Statistical Area	PWID Population	Year	Number	Min	Max	Rank	Rate	Min	Max	Rank
Orlando, FL	Male	1992	4,457	2,318	5,562	63	102.02	53.05	127.31	76
		1993	4,090	2,100	5,888	65	91.40	46.94	131.58	79
		1994	5,039	3,630	6,235	57	109.89	79.16	135.97	67
		1995	4,527	2,143	6,583	57	96.40	45.64	140.19	69
		1996	4,681	2,179	6,974	54	96.91	45.10	144.39	68
		1997	4,852	2,229	7,438	53	96.78	44.45	148.36	66
		1998	5,088	2,274	7,923	48	98.18	43.89	152.91	65
		1999	6,359	4,827	8,386	41	119.35	90.60	157.39	55
		2000	5,637	2,340	8,970	44	102.26	42.45	162.71	61
		2001	7,071	4,727	9,580	37	124.03	82.92	168.04	53
		2002	5,978	2,205	10,171	42	101.82	37.56	173.25	59
		2003	5,959	2,130	10,788	43	98.63	35.26	178.56	62
		2004	5,976	2,072	11,550	42	95.40	33.07	184.37	62
		2005	6,050	1,997	12,438	41	92.87	30.65	190.93	64
		2006	6,079	1,900	13,287	41	90.59	28.32	197.99	64
		2007	6,044	1,750	14,036	43	88.59	25.65	205.74	65
	Female	1992	2,171	1,129	2,710	66	49.77	25.88	62.11	74
		1993	2,039	1,047	2,936	66	45.64	23.44	65.71	79
		1994	2,585	1,862	3,199	60	56.33	40.58	69.70	68
		1995	2,399	1,136	3,488	59	50.93	24.11	74.06	73
		1996	2,569	1,195	3,827	55	52.91	24.62	78.83	67
		1997	2,760	1,268	4,230	51	54.76	25.15	83.94	66
		1998	2,998	1,340	4,670	47	57.42	25.67	89.42	61
		1999	3,877	2,943	5,112	39	72.09	54.72	95.06	51
		2000	3,543	1,471	5,638	42	63.62	26.41	101.24	57
		2001	4,563	3,050	6,182	37	79.28	53.00	107.41	42
		2002	3,937	1,452	6,699	40	66.41	24.50	113.00	55
		2003	3,978	1,422	7,202	41	65.38	23.37	118.36	55
		2004	4,010	1,390	7,751	38	63.72	22.09	123.16	55
		2005	4,043	1,334	8,312	38	61.82	20.40	127.09	54
		2006	4,002	1,251	8,747	37	59.49	18.60	130.03	57
		2007	3,872	1,121	8,993	37	56.79	16.44	131.90	60

Metropolitan Statistical Area	PWID Population	Year	Number	Min	Max	Rank	Rate	Min	Max	Rank
Orlando, FL	Young (15-29)	1992	1,560	811	1,946	72	52.05	27.07	64.96	77
		1993	1,456	748	2,096	69	48.59	24.95	69.95	75
		1994	1,894	1,364	2,344	56	62.92	45.33	77.86	65
		1995	1,848	875	2,687	55	61.03	28.89	88.74	60
		1996	2,114	984	3,149	48	68.55	31.90	102.13	57
		1997	2,445	1,123	3,749	44	76.84	35.30	117.80	55
		1998	2,863	1,280	4,458	35	87.35	39.05	136.04	51
		1999	3,965	3,010	5,229	27	118.14	89.68	155.80	32
		2000	3,842	1,595	6,113	28	111.17	46.15	176.88	36
		2001	5,169	3,456	7,003	18	145.96	97.58	197.75	24
		2002	4,579	1,689	7,791	25	126.42	46.64	215.10	28
		2003	4,654	1,664	8,425	25	125.32	44.80	226.87	27
		2004	4,606	1,597	8,902	28	119.94	41.58	231.81	34
		2005	4,421	1,459	9,090	31	110.79	36.57	227.78	40
		2006	4,005	1,252	8,753	34	97.29	30.41	212.63	49
		2007	3,359	973	7,801	42	80.67	23.35	187.34	61
	Old (30-64)	1992	5,144	2,675	6,420	60	89.68	46.64	111.92	71
		1993	4,723	2,425	6,799	63	79.42	40.78	114.33	76
		1994	5,778	4,162	7,149	59	93.73	67.52	115.97	65
		1995	5,115	2,422	7,438	59	80.20	37.97	116.63	73
		1996	5,175	2,408	7,709	59	78.39	36.48	116.78	73
		1997	5,208	2,392	7,984	58	75.81	34.82	116.21	73
		1998	5,272	2,357	8,211	56	73.98	33.07	115.21	71
		1999	6,335	4,809	8,354	48	86.20	65.43	113.67	60
		2000	5,394	2,239	8,583	54	70.74	29.37	112.56	68
		2001	6,522	4,360	8,836	45	82.40	55.09	111.64	61
		2002	5,360	1,977	9,119	51	65.55	24.18	111.53	68
		2003	5,264	1,882	9,529	51	62.57	22.37	113.28	71
		2004	5,295	1,836	10,233	49	60.74	21.06	117.38	75
		2005	5,491	1,812	11,289	49	60.58	20.00	124.55	71
		2006	5,779	1,806	12,630	46	61.99	19.38	135.49	68
		2007	6,133	1,776	14,244	40	64.72	18.74	150.31	62

Metropolitan Statistical Area	PWID Population	Year	Number	Min	Max	Rank	Rate	Min	Max	Rank
Philadelphia, PA--NJ	Total	1992	49,848	45,554	52,220	4	153.86	140.61	161.19	31
		1993	46,814	35,025	53,969	3	144.39	108.03	166.46	32
		1994	51,633	44,556	56,195	4	158.84	137.06	172.87	25
		1995	48,034	34,582	58,422	3	147.33	106.07	179.19	29
		1996	48,426	34,381	60,804	4	148.12	105.16	185.98	27
		1997	48,845	34,144	63,689	4	149.00	104.16	194.29	25
		1998	49,334	33,984	65,451	4	149.75	103.16	198.67	21
		1999	55,414	40,471	65,915	3	167.28	122.17	198.98	14
		2000	51,069	34,120	65,712	4	153.22	102.37	197.15	17
		2001	57,670	46,979	63,468	3	172.07	140.17	189.37	11
		2002	52,335	34,914	64,062	5	155.00	103.40	189.73	14
		2003	52,820	35,335	65,569	5	155.35	103.92	192.84	14
		2004	54,067	35,740	67,167	5	157.78	104.30	196.01	14
		2005	54,373	36,087	68,726	5	157.62	104.61	199.22	14
		2006	54,669	36,424	73,633	5	157.48	104.92	212.10	13
		2007	54,962	32,043	79,293	5	157.53	91.84	227.26	14
	Non-Hispanic White	1992	21,630	19,767	22,660	4	89.72	82.00	93.99	42
		1993	20,381	15,249	23,496	5	85.05	63.63	98.05	39
		1994	22,698	19,587	24,704	3	95.05	82.02	103.45	41
		1995	21,432	15,430	26,067	4	90.02	64.81	109.49	42
		1996	22,017	15,632	27,645	4	92.80	65.89	116.52	41
		1997	22,695	15,864	29,591	4	95.98	67.09	125.14	35
		1998	23,466	16,164	31,131	4	99.31	68.41	131.76	35
		1999	27,004	19,722	32,122	1	114.32	83.49	135.99	26
		2000	25,495	17,034	32,805	2	107.84	72.05	138.77	26
		2001	29,466	24,004	32,429	1	124.49	101.41	137.01	20
		2002	27,322	18,227	33,444	2	115.16	76.83	140.97	21
		2003	28,108	18,803	34,892	2	118.30	79.14	146.85	17
		2004	29,238	19,327	36,322	2	122.80	81.18	152.56	16
		2005	29,773	19,760	37,632	2	124.91	82.90	157.88	17
		2006	30,185	20,111	40,656	2	126.55	84.32	170.45	17
		2007	30,458	17,757	43,941	2	127.70	74.45	184.23	18

Metropolitan Statistical Area	PWID Population	Year	Number	Min	Max	Rank	Rate	Min	Max	Rank
Philadelphia, PA--NJ	Non-Hispanic Black	1992	21,463	19,614	22,485	3	348.99	318.92	365.60	40
		1993	19,754	14,779	22,773	3	318.06	237.97	366.67	40
		1994	21,145	18,247	23,014	4	337.18	290.96	366.97	35
		1995	18,927	13,626	23,020	4	299.18	215.39	363.89	36
		1996	18,219	12,935	22,876	4	286.07	203.10	359.19	33
		1997	17,432	12,185	22,729	4	271.65	189.89	354.20	32
		1998	16,615	11,445	22,043	4	256.62	176.77	340.46	32
		1999	17,552	12,819	20,878	5	268.50	196.10	319.38	22
		2000	15,191	10,149	19,546	5	230.63	154.08	296.75	32
		2001	16,124	13,135	17,745	5	243.01	197.96	267.44	23
		2002	13,802	9,208	16,895	6	205.84	137.32	251.96	35
		2003	13,222	8,845	16,414	8	194.91	130.38	241.95	35
		2004	12,968	8,572	16,110	8	188.57	124.65	234.26	36
		2005	12,654	8,398	15,994	8	181.90	120.72	229.91	39
		2006	12,544	8,357	16,895	7	178.43	118.88	240.32	41
		2007	12,677	7,391	18,289	6	178.86	104.28	258.04	43
	Hispanic	1992	6,973	6,372	7,304	9	565.35	516.65	592.26	5
		1993	6,038	4,517	6,961	11	470.37	351.92	542.26	6
		1994	6,250	5,394	6,803	11	468.22	404.04	509.59	6
		1995	5,549	3,995	6,749	12	399.49	287.61	485.88	7
		1996	5,420	3,848	6,806	12	372.97	264.79	468.30	8
		1997	5,371	3,754	7,003	12	354.57	247.85	462.32	9
		1998	5,394	3,716	7,156	13	342.33	235.81	454.16	11
		1999	6,089	4,447	7,243	13	373.30	272.64	444.04	8
		2000	5,692	3,803	7,324	12	336.50	224.82	432.99	11
		2001	6,571	5,353	7,232	11	374.61	305.16	412.27	9
		2002	6,136	4,094	7,511	12	336.92	224.77	412.42	11
		2003	6,407	4,286	7,953	8	339.01	226.79	420.84	11
		2004	6,813	4,504	8,464	8	346.83	229.27	430.87	11
		2005	7,143	4,741	9,029	6	351.22	233.11	443.94	11
		2006	7,508	5,002	10,113	6	355.65	236.96	479.03	9
		2007	7,909	4,611	11,410	5	361.92	211.01	522.15	8

Metropolitan Statistical Area	PWID Population	Year	Number	Min	Max	Rank	Rate	Min	Max	Rank
Philadelphia, PA--NJ	Male	1992	38,043	34,765	39,853	3	241.26	220.47	252.74	21
		1993	34,719	25,976	40,026	3	219.98	164.59	253.60	24
		1994	37,412	32,284	40,718	3	236.43	204.03	257.32	21
		1995	34,187	24,613	41,581	3	215.34	155.03	261.91	22
		1996	34,021	24,154	42,717	3	213.77	151.77	268.41	19
		1997	34,008	23,772	44,343	4	213.09	148.95	277.84	19
		1998	34,136	23,515	45,288	4	212.89	146.65	282.43	16
		1999	38,163	27,872	45,395	3	236.74	172.90	281.60	13
		2000	35,006	23,388	45,043	4	215.85	144.21	277.74	12
		2001	39,289	32,005	43,239	3	240.65	196.04	264.84	11
		2002	35,328	23,569	43,245	4	214.57	143.15	262.66	14
		2003	35,161	23,522	43,648	5	211.75	141.65	262.86	13
		2004	35,250	23,302	43,791	5	210.40	139.09	261.38	13
		2005	34,398	22,830	43,478	5	203.78	135.25	257.58	15
		2006	33,138	22,079	44,633	5	194.92	129.87	262.53	15
		2007	31,385	18,298	45,280	5	183.51	106.99	264.74	18
	Female	1992	12,674	11,582	13,277	4	76.22	69.65	79.85	44
		1993	12,349	9,240	14,237	7	74.22	55.53	85.57	42
		1994	14,186	12,242	15,440	4	85.03	73.38	92.54	39
		1995	13,770	9,914	16,749	6	82.32	59.27	100.13	39
		1996	14,483	10,282	18,185	5	86.32	61.28	108.38	37
		1997	15,209	10,632	19,831	5	90.42	63.20	117.89	29
		1998	15,934	10,976	21,139	5	94.23	64.91	125.02	25
		1999	18,466	13,486	21,965	4	108.58	79.30	129.16	20
		2000	17,438	11,651	22,438	5	101.90	68.08	131.12	18
		2001	20,013	16,303	22,025	4	116.42	94.84	128.13	14
		2002	18,277	12,193	22,373	5	105.64	70.48	129.32	18
		2003	18,354	12,278	22,784	5	105.51	70.58	130.97	19
		2004	18,449	12,195	22,919	5	105.34	69.63	130.86	19
		2005	17,943	11,909	22,679	5	101.84	67.59	128.73	20
		2006	17,139	11,419	23,084	5	96.75	64.46	130.31	22
		2007	16,034	9,348	23,132	5	90.14	52.55	130.05	23

Metropolitan Statistical Area	PWID Population	Year	Number	Min	Max	Rank	Rate	Min	Max	Rank
Philadelphia, PA--NJ	Young (15-29)	1992	11,883	10,860	12,449	4	111.23	101.65	116.53	35
		1993	10,849	8,117	12,507	5	103.29	77.28	119.08	40
		1994	11,983	10,341	13,042	4	115.79	99.92	126.02	29
		1995	11,447	8,241	13,923	4	111.95	80.60	136.17	29
		1996	12,088	8,582	15,178	4	119.51	84.85	150.06	28
		1997	12,958	9,058	16,895	4	128.95	90.13	168.13	23
		1998	14,036	9,668	18,621	4	140.45	96.75	186.33	16
		1999	16,976	12,398	20,193	2	171.22	125.05	203.67	13
		2000	16,833	11,247	21,660	3	170.83	114.13	219.81	14
		2001	20,351	16,579	22,397	1	207.58	169.10	228.45	9
		2002	19,601	13,077	23,994	3	199.19	132.88	243.82	8
		2003	20,748	13,879	25,755	3	209.00	139.81	259.45	8
		2004	21,946	14,507	27,263	3	218.56	144.48	271.52	7
		2005	22,409	14,873	28,324	2	220.73	146.50	279.00	9
		2006	22,409	14,931	30,183	2	218.35	145.48	294.09	9
		2007	21,869	12,750	31,550	2	211.53	123.33	305.18	9
	Old (30-64)	1992	38,928	35,574	40,780	4	179.27	163.83	187.80	29
		1993	36,200	27,084	41,733	3	165.17	123.57	190.41	28
		1994	39,547	34,126	43,041	3	178.48	154.01	194.25	21
		1995	36,434	26,230	44,314	3	162.81	117.21	198.02	27
		1996	36,344	25,803	45,633	3	160.95	114.27	202.09	26
		1997	36,210	25,311	47,214	3	159.29	111.34	207.69	25
		1998	36,030	24,819	47,801	4	156.99	108.14	208.27	22
		1999	39,726	29,014	47,254	3	171.15	125.00	203.58	16
		2000	35,766	23,896	46,021	5	152.35	101.79	196.03	20
		2001	39,218	31,948	43,161	4	165.40	134.73	182.02	14
		2002	34,294	22,879	41,979	5	143.34	95.63	175.47	19
		2003	33,029	22,095	41,001	5	137.20	91.78	170.31	22
		2004	31,863	21,063	39,583	5	131.52	86.94	163.39	23
		2005	29,720	19,725	37,566	5	122.08	81.02	154.30	24
		2006	27,157	18,094	36,578	5	111.06	74.00	149.59	27
		2007	24,202	14,110	34,915	5	98.57	57.47	142.21	33

Metropolitan Statistical Area	PWID Population	Year	Number	Min	Max	Rank	Rate	Min	Max	Rank
Phoenix--Mesa, AZ	Total	1992	18,535	9,640	29,799	25	120.13	62.48	193.13	46
		1993	17,846	9,144	28,569	25	111.51	57.14	178.51	45
		1994	18,110	8,793	27,847	23	106.99	51.95	164.51	48
		1995	17,994	8,324	26,777	23	101.14	46.79	150.51	50
		1996	17,891	7,730	25,759	22	96.48	41.69	138.92	51
		1997	17,911	7,558	24,694	21	92.35	38.97	127.33	52
		1998	18,417	7,944	25,377	20	91.71	39.55	126.36	53
		1999	18,845	8,958	26,031	19	90.78	43.15	125.39	57
		2000	20,259	10,495	28,297	18	94.55	48.98	132.06	52
		2001	21,980	12,217	30,769	15	99.11	55.09	138.74	52
		2002	22,724	13,772	33,763	16	99.13	60.08	147.29	46
		2003	23,878	15,055	36,770	15	101.20	63.81	155.84	45
		2004	25,152	15,964	40,465	15	103.07	65.41	165.81	42
		2005	26,659	16,874	44,750	13	104.72	66.28	175.78	41
		2006	28,240	17,852	49,324	11	106.46	67.30	185.94	35
		2007	29,573	17,967	53,590	10	108.22	65.75	196.10	34
	Non-Hispanic White	1992	13,167	6,848	21,168	14	114.31	59.45	183.77	29
		1993	12,912	6,616	20,670	13	109.52	56.12	175.33	32
		1994	13,105	6,363	20,151	11	106.38	51.66	163.58	32
		1995	12,839	5,939	19,107	12	100.45	46.47	149.49	32
		1996	12,438	5,374	17,908	12	94.73	40.93	136.39	38
		1997	12,012	5,068	16,560	13	88.60	37.39	122.15	42
		1998	11,820	5,098	16,287	13	85.38	36.83	117.65	44
		1999	11,520	5,476	15,914	15	81.64	38.81	112.78	48
		2000	11,790	6,108	16,468	13	82.15	42.56	114.75	45
		2001	12,239	6,803	17,133	16	83.53	46.43	116.93	44
		2002	12,248	7,423	18,198	11	81.86	49.61	121.63	43
		2003	12,687	7,999	19,537	12	83.27	52.50	128.22	42
		2004	13,496	8,566	21,713	11	86.51	54.91	139.17	41
		2005	14,850	9,399	24,927	11	92.44	58.51	155.16	39
		2006	16,784	10,610	29,316	7	101.86	64.39	177.92	31
		2007	19,185	11,656	34,766	7	114.83	69.77	208.09	24

Metropolitan Statistical Area	PWID Population	Year	Number	Min	Max	Rank	Rate	Min	Max	Rank
Phoenix--Mesa, AZ	Non-Hispanic Black	1992	1,427	742	2,294	64	271.04	140.96	435.73	54
		1993	993	509	1,589	73	181.23	92.86	290.12	75
		1994	816	396	1,255	83	137.68	66.85	211.71	87
		1995	729	337	1,085	84	115.39	53.38	171.72	91
		1996	714	308	1,028	83	106.26	45.91	152.99	89
		1997	759	320	1,046	76	106.76	45.05	147.19	88
		1998	879	379	1,212	67	118.32	51.03	163.03	80
		1999	1,059	504	1,463	65	136.84	65.05	189.03	74
		2000	1,377	714	1,924	58	170.46	88.31	238.10	52
		2001	1,826	1,015	2,556	46	215.66	119.87	301.89	29
		2002	2,295	1,391	3,409	31	258.16	156.46	383.57	17
		2003	2,871	1,810	4,420	25	309.52	195.15	476.62	13
		2004	3,481	2,209	5,600	20	356.11	226.02	572.92	9
		2005	4,058	2,569	6,812	18	389.09	246.28	653.12	8
		2006	4,474	2,828	7,814	16	398.42	251.85	695.88	9
		2007	4,568	2,775	8,278	17	383.18	232.80	694.38	12
	Hispanic	1992	2,729	1,419	4,387	28	97.91	50.92	157.40	61
		1993	2,861	1,466	4,580	28	94.46	48.40	151.23	56
		1994	3,111	1,510	4,783	27	93.51	45.41	143.80	57
		1995	3,265	1,510	4,858	23	89.81	41.55	133.66	52
		1996	3,387	1,463	4,876	23	85.82	37.08	123.56	52
		1997	3,501	1,477	4,827	20	81.86	34.54	112.86	53
		1998	3,686	1,590	5,078	19	80.15	34.57	110.43	53
		1999	3,835	1,823	5,297	17	77.84	37.00	107.52	56
		2000	4,171	2,161	5,826	16	79.15	41.00	110.55	51
		2001	4,563	2,536	6,388	17	81.16	45.11	113.62	54
		2002	4,751	2,879	7,058	16	79.63	48.26	118.31	48
		2003	5,029	3,171	7,744	15	80.11	50.51	123.37	48
		2004	5,349	3,395	8,606	14	80.84	51.31	130.06	46
		2005	5,751	3,640	9,654	12	81.43	51.54	136.69	46
		2006	6,221	3,933	10,866	12	82.45	52.12	144.01	45
		2007	6,712	4,078	12,162	9	84.15	51.12	152.49	46

Metropolitan Statistical Area	PWID Population	Year	Number	Min	Max	Rank	Rate	Min	Max	Rank
Phoenix--Mesa, AZ	Male	1992	12,822	6,668	20,613	23	164.98	85.80	265.23	43
		1993	12,327	6,316	19,734	24	152.63	78.21	244.35	41
		1994	12,521	6,080	19,253	22	146.27	71.02	224.91	45
		1995	12,478	5,772	18,569	20	138.41	64.03	205.97	44
		1996	12,464	5,385	17,945	19	132.44	57.23	190.68	47
		1997	12,552	5,296	17,305	18	127.31	53.72	175.52	47
		1998	12,994	5,604	17,904	18	127.12	54.83	175.16	47
		1999	13,392	6,366	18,500	17	126.54	60.15	174.80	48
		2000	14,505	7,514	20,260	13	132.54	68.66	185.13	44
		2001	15,852	8,811	22,191	13	139.76	77.69	195.65	39
		2002	16,501	10,000	24,516	13	140.62	85.22	208.93	40
		2003	17,444	10,998	26,861	11	144.41	91.05	222.38	38
		2004	18,467	11,721	29,710	11	147.71	93.75	237.63	36
		2005	19,647	12,436	32,979	11	150.61	95.33	252.81	32
		2006	20,858	13,185	36,431	8	153.30	96.91	267.76	29
		2007	21,851	13,276	39,597	8	155.71	94.60	282.16	28
	Female	1992	6,117	3,181	9,834	26	79.88	41.54	128.42	42
		1993	5,883	3,014	9,418	26	74.20	38.02	118.79	43
		1994	5,940	2,884	9,133	25	70.99	34.47	109.15	54
		1995	5,850	2,706	8,706	26	66.66	30.84	99.20	55
		1996	5,748	2,484	8,276	25	62.95	27.20	90.63	58
		1997	5,671	2,393	7,818	25	59.47	25.10	82.00	59
		1998	5,732	2,472	7,898	24	58.13	25.07	80.09	59
		1999	5,754	2,735	7,948	25	56.54	26.88	78.10	65
		2000	6,058	3,138	8,462	23	57.79	29.94	80.72	62
		2001	6,430	3,574	9,000	21	59.34	32.98	83.06	64
		2002	6,497	3,938	9,653	21	58.07	35.19	86.28	62
		2003	6,670	4,206	10,272	21	57.93	36.52	89.20	62
		2004	6,866	4,358	11,046	21	57.69	36.61	92.81	61
		2005	7,116	4,504	11,945	19	57.33	36.29	96.23	62
		2006	7,380	4,665	12,890	18	57.12	36.11	99.76	60
		2007	7,581	4,606	13,738	17	57.03	34.65	103.34	58

Metropolitan Statistical Area	PWID Population	Year	Number	Min	Max	Rank	Rate	Min	Max	Rank
Phoenix--Mesa, AZ	Young (15-29)	1992	6,168	3,208	9,915	18	113.44	59.00	182.38	33
		1993	5,997	3,073	9,601	17	108.15	55.42	173.15	31
		1994	6,060	2,943	9,319	16	104.91	50.94	161.32	37
		1995	5,931	2,744	8,826	15	98.33	45.49	146.33	43
		1996	5,763	2,490	8,297	16	91.88	39.70	132.29	47
		1997	5,612	2,368	7,737	15	85.60	36.12	118.02	51
		1998	5,605	2,418	7,723	16	82.35	35.52	113.47	54
		1999	5,583	2,654	7,712	16	79.30	37.70	109.54	58
		2000	5,876	3,044	8,208	15	80.96	41.94	113.08	57
		2001	6,303	3,504	8,824	16	84.85	47.16	118.77	61
		2002	6,530	3,958	9,703	14	85.81	52.01	127.50	56
		2003	6,995	4,410	10,771	12	90.00	56.75	138.60	57
		2004	7,665	4,865	12,331	12	95.92	60.88	154.32	55
		2005	8,643	5,471	14,509	11	104.39	66.07	175.23	47
		2006	9,974	6,305	17,421	11	116.15	73.42	202.87	36
		2007	11,639	7,071	21,091	7	132.36	80.41	239.85	28
	Old (30-64)	1992	12,672	6,591	20,373	27	126.82	65.96	203.88	47
		1993	12,068	6,184	19,320	26	115.39	59.12	184.72	46
		1994	12,234	5,940	18,811	27	109.71	53.27	168.70	53
		1995	12,235	5,660	18,208	26	104.04	48.13	154.83	51
		1996	12,308	5,318	17,720	25	100.30	43.34	144.41	54
		1997	12,497	5,273	17,230	25	97.34	41.07	134.20	57
		1998	13,034	5,622	17,959	23	98.17	42.34	135.27	55
		1999	13,499	6,417	18,647	20	98.39	46.77	135.91	56
		2000	14,632	7,580	20,437	19	103.27	53.50	144.25	46
		2001	15,916	8,847	22,281	16	107.92	59.98	151.07	46
		2002	16,378	9,926	24,334	17	106.95	64.82	158.91	41
		2003	16,967	10,697	26,127	17	107.23	67.61	165.13	37
		2004	17,399	11,043	27,992	15	106.00	67.28	170.54	35
		2005	17,644	11,168	29,617	15	102.72	65.02	172.42	36
		2006	17,445	11,028	30,469	14	97.24	61.47	169.85	37
		2007	16,453	9,996	29,815	13	88.77	53.93	160.87	39

Metropolitan Statistical Area	PWID Population	Year	Number	Min	Max	Rank	Rate	Min	Max	Rank
Pittsburgh, PA	Total	1992	11,831	5,213	16,456	39	76.89	33.88	106.95	75
		1993	10,794	5,730	16,336	41	70.31	37.32	106.41	75
		1994	12,144	6,356	16,201	38	79.37	41.55	105.89	74
		1995	10,851	6,481	16,062	39	71.19	42.52	105.37	72
		1996	10,939	6,126	15,971	35	71.95	40.30	105.05	72
		1997	11,121	6,184	15,868	35	73.40	40.81	104.72	67
		1998	11,545	6,852	15,750	33	76.39	45.34	104.21	66
		1999	13,463	10,742	15,647	33	89.22	71.19	103.70	58
		2000	12,723	9,730	15,358	32	84.40	64.55	101.89	58
		2001	13,996	12,779	15,090	31	92.82	84.75	100.08	55
		2002	13,845	12,511	14,647	30	91.71	82.87	97.02	53
		2003	14,310	12,236	16,528	27	94.72	80.99	109.40	48
		2004	14,644	11,404	19,080	27	96.96	75.51	126.33	46
		2005	14,782	10,572	20,843	27	97.90	70.02	138.05	46
		2006	14,699	9,658	21,812	27	97.34	63.96	144.45	43
		2007	14,611	8,753	22,724	26	96.65	57.91	150.33	43
	Non-Hispanic White	1992	6,653	2,932	9,254	35	47.56	20.96	66.16	78
		1993	5,784	3,070	8,754	37	41.51	22.03	62.82	81
		1994	6,374	3,336	8,503	33	45.96	24.06	61.32	79
		1995	5,716	3,414	8,461	35	41.44	24.75	61.34	78
		1996	5,898	3,303	8,612	34	42.95	24.05	62.70	78
		1997	6,224	3,461	8,880	31	45.54	25.32	64.98	73
		1998	6,760	4,012	9,222	29	49.67	29.48	67.76	71
		1999	8,272	6,600	9,613	24	60.99	48.66	70.88	61
		2000	8,191	6,264	9,888	24	60.59	46.34	73.14	61
		2001	9,400	8,582	10,135	20	69.64	63.58	75.08	58
		2002	9,635	8,707	10,193	20	71.43	64.55	75.57	55
		2003	10,232	8,750	11,819	20	75.98	64.97	87.76	51
		2004	10,659	8,301	13,888	20	79.29	61.75	103.31	47
		2005	10,835	7,749	15,278	19	80.75	57.75	113.86	46
		2006	10,717	7,042	15,902	19	80.00	52.57	118.72	46
		2007	10,438	6,253	16,234	20	78.02	46.74	121.35	48

Metropolitan Statistical Area	PWID Population	Year	Number	Min	Max	Rank	Rate	Min	Max	Rank
Pittsburgh, PA	Non-Hispanic Black	1992	4,646	2,047	6,463	23	405.06	178.49	563.43	29
		1993	4,489	2,383	6,793	22	389.34	206.67	589.22	28
		1994	5,138	2,690	6,855	21	443.97	232.39	592.32	16
		1995	4,522	2,701	6,694	23	388.61	232.10	575.21	16
		1996	4,368	2,446	6,377	22	373.30	209.07	545.02	15
		1997	4,153	2,310	5,926	22	352.86	196.21	503.46	15
		1998	3,952	2,345	5,391	22	334.20	198.34	455.91	16
		1999	4,157	3,317	4,831	22	349.15	278.57	405.78	13
		2000	3,509	2,684	4,236	22	292.63	223.78	353.24	15
		2001	3,440	3,141	3,709	22	284.89	260.10	307.14	16
		2002	3,053	2,758	3,229	23	250.17	226.07	264.66	21
		2003	2,877	2,460	3,323	24	233.12	199.34	269.26	21
		2004	2,757	2,147	3,593	25	221.75	172.68	288.92	23
		2005	2,705	1,935	3,815	25	216.08	154.54	304.68	24
		2006	2,740	1,800	4,066	27	217.00	142.59	322.01	25
		2007	2,928	1,754	4,555	27	228.78	137.06	355.83	25
	Hispanic	1992	101	45	141	76	109.86	48.41	152.81	59
		1993	107	57	162	75	113.79	60.41	172.22	51
		1994	130	68	173	74	134.77	70.54	179.80	42
		1995	117	70	173	75	119.20	71.20	176.44	43
		1996	113	63	164	76	111.14	62.25	162.26	44
		1997	105	59	150	77	101.91	56.67	145.40	45
		1998	98	58	134	78	92.85	55.11	126.67	47
		1999	102	81	118	79	94.11	75.09	109.37	43
		2000	86	65	103	81	75.03	57.38	90.57	54
		2001	85	78	92	84	69.89	63.81	75.35	59
		2002	78	71	83	84	60.61	54.77	64.12	61
		2003	79	67	91	86	57.99	49.59	66.98	62
		2004	83	65	108	85	58.65	45.67	76.41	60
		2005	93	67	131	84	63.25	45.23	89.18	59
		2006	112	73	166	83	72.34	47.54	107.35	54
		2007	149	89	231	79	90.96	54.50	141.48	42

Metropolitan Statistical Area	PWID Population	Year	Number	Min	Max	Rank	Rate	Min	Max	Rank
Pittsburgh, PA	Male	1992	7,823	3,447	10,881	39	104.76	46.16	145.71	74
		1993	7,065	3,750	10,692	39	94.71	50.28	143.34	74
		1994	7,878	4,124	10,511	39	105.84	55.40	141.20	72
		1995	6,986	4,173	10,341	40	94.10	56.20	139.29	74
		1996	6,996	3,919	10,215	38	94.43	52.89	137.87	72
		1997	7,073	3,933	10,092	35	95.67	53.20	136.51	67
		1998	7,306	4,336	9,967	35	98.96	58.73	135.00	64
		1999	8,482	6,768	9,858	33	114.94	91.71	133.58	58
		2000	7,983	6,105	9,637	33	108.19	82.73	130.59	56
		2001	8,748	7,987	9,431	32	118.40	108.10	127.64	56
		2002	8,619	7,789	9,119	32	116.33	105.13	123.07	54
		2003	8,871	7,586	10,246	29	119.47	102.16	137.99	47
		2004	9,036	7,037	11,773	28	121.60	94.69	158.43	45
		2005	9,072	6,488	12,792	26	122.02	87.27	172.05	45
		2006	8,964	5,890	13,302	28	120.38	79.10	178.62	47
		2007	8,844	5,298	13,755	27	118.44	70.96	184.21	47
	Female	1992	4,193	1,848	5,833	38	52.95	23.33	73.66	71
		1993	3,810	2,022	5,765	36	48.27	25.62	73.05	74
		1994	4,303	2,253	5,741	37	54.78	28.67	73.08	73
		1995	3,888	2,322	5,755	37	49.73	29.70	73.60	75
		1996	3,984	2,231	5,817	35	51.12	28.63	74.63	71
		1997	4,134	2,299	5,899	34	53.28	29.63	76.01	67
		1998	4,391	2,606	5,990	34	56.79	33.71	77.48	62
		1999	5,244	4,184	6,094	29	68.02	54.27	79.05	55
		2000	5,074	3,880	6,124	27	65.94	50.42	79.59	55
		2001	5,705	5,209	6,151	23	74.19	67.74	79.99	51
		2002	5,753	5,199	6,086	23	74.83	67.62	79.16	42
		2003	6,037	5,162	6,973	22	78.59	67.20	90.77	37
		2004	6,243	4,861	8,133	22	81.36	63.36	106.01	32
		2005	6,328	4,525	8,922	22	82.57	59.05	116.42	29
		2006	6,273	4,122	9,308	22	81.96	53.86	121.63	32
		2007	6,164	3,693	9,586	22	80.57	48.27	125.32	31

Metropolitan Statistical Area	PWID Population	Year	Number	Min	Max	Rank	Rate	Min	Max	Rank
Pittsburgh, PA	Young (15-29)	1992	2,723	1,200	3,788	42	58.36	25.71	81.17	74
		1993	1,890	1,003	2,860	52	41.20	21.87	62.36	81
		1994	1,809	947	2,413	61	40.17	21.03	53.60	87
		1995	1,531	915	2,267	66	34.55	20.64	51.15	89
		1996	1,607	900	2,346	64	36.73	20.57	53.62	88
		1997	1,832	1,019	2,613	59	42.38	23.57	60.47	85
		1998	2,249	1,335	3,068	48	52.60	31.21	71.75	73
		1999	3,197	2,551	3,716	34	76.04	60.67	88.37	60
		2000	3,714	2,840	4,484	32	89.64	68.55	108.21	52
		2001	4,965	4,533	5,353	22	120.96	110.44	130.41	34
		2002	5,812	5,252	6,148	19	141.84	128.18	150.06	24
		2003	6,855	5,861	7,918	14	166.56	142.42	192.38	18
		2004	7,689	5,988	10,018	11	186.14	144.95	242.51	15
		2005	8,156	5,833	11,501	12	196.17	140.30	276.61	14
		2006	8,147	5,354	12,090	12	194.67	127.91	288.86	15
		2007	7,710	4,619	11,991	13	182.81	109.52	284.33	15
	Old (30-64)	1992	9,344	4,118	12,998	37	87.17	38.41	121.25	74
		1993	8,992	4,773	13,608	36	83.52	44.34	126.40	72
		1994	10,371	5,428	13,836	35	96.04	50.27	128.13	63
		1995	9,336	5,576	13,819	34	86.35	51.58	127.82	68
		1996	9,358	5,241	13,663	32	86.42	48.40	126.17	66
		1997	9,348	5,198	13,338	31	86.31	48.00	123.15	61
		1998	9,414	5,587	12,843	31	86.86	51.55	118.50	60
		1999	10,497	8,375	12,199	29	96.44	76.94	112.08	57
		2000	9,334	7,138	11,267	31	85.39	65.30	103.08	60
		2001	9,507	8,680	10,250	31	86.64	79.10	93.41	59
		2002	8,595	7,767	9,093	34	78.14	70.61	82.66	57
		2003	8,071	6,901	9,322	34	73.43	62.79	84.81	61
		2004	7,542	5,873	9,827	36	68.74	53.53	89.56	63
		2005	7,084	5,067	9,989	36	64.75	46.31	91.30	65
		2006	6,779	4,454	10,059	35	62.11	40.81	92.16	67
		2007	6,794	4,070	10,566	35	62.33	37.34	96.95	66

Metropolitan Statistical Area	PWID Population	Year	Number	Min	Max	Rank	Rate	Min	Max	Rank
Portland--Vancouver, OR--WA	Total	1992	21,661	17,170	25,773	20	202.30	160.36	240.71	15
		1993	24,273	17,910	33,996	15	219.37	161.87	307.24	7
		1994	20,264	18,558	22,091	21	178.39	163.37	194.47	17
		1995	23,114	19,065	33,889	15	197.79	163.14	289.98	9
		1996	22,831	18,198	33,807	15	189.04	150.67	279.91	9
		1997	22,641	17,155	33,363	15	182.41	138.21	268.80	9
		1998	22,699	16,554	32,694	15	178.91	130.48	257.68	9
		1999	19,699	15,874	22,131	18	152.31	122.74	171.12	20
		2000	23,005	16,599	30,468	14	174.66	126.03	231.33	8
		2001	21,401	17,444	23,513	17	158.66	129.32	174.31	16
		2002	23,344	19,341	26,276	15	169.49	140.42	190.77	8
		2003	23,296	21,155	24,791	16	166.86	151.53	177.57	9
		2004	23,158	21,295	25,299	16	164.06	150.86	179.22	11
		2005	23,021	18,818	26,004	16	160.14	130.90	180.89	13
		2006	22,923	16,279	26,851	16	156.23	110.94	182.99	14
		2007	22,797	13,643	28,939	16	152.44	91.23	193.51	17
	Non-Hispanic White	1992	15,441	12,240	18,373	8	162.64	128.92	193.52	16
		1993	17,424	12,856	24,403	7	178.65	131.82	250.21	8
		1994	14,613	13,383	15,930	8	146.92	134.56	160.17	19
		1995	16,710	13,783	24,499	6	164.62	135.78	241.36	8
		1996	16,517	13,166	24,458	5	158.59	126.41	234.83	7
		1997	16,365	12,400	24,115	5	154.08	116.75	227.05	9
		1998	16,368	11,937	23,575	5	152.00	110.85	218.93	8
		1999	14,151	11,403	15,898	7	130.07	104.82	146.13	20
		2000	16,441	11,863	21,774	6	149.63	107.96	198.17	7
		2001	15,197	12,387	16,697	7	135.80	110.69	149.20	15
		2002	16,452	13,631	18,518	6	144.66	119.86	162.83	9
		2003	16,275	14,779	17,320	6	141.78	128.74	150.88	11
		2004	16,021	14,732	17,502	6	138.58	127.43	151.39	11
		2005	15,756	12,879	17,798	8	134.49	109.94	151.92	12
		2006	15,507	11,012	18,164	9	130.38	92.59	152.72	12
		2007	15,232	9,115	19,335	9	126.34	75.60	160.37	19

Metropolitan Statistical Area	PWID Population	Year	Number	Min	Max	Rank	Rate	Min	Max	Rank
Portland--Vancouver, OR--WA	Non-Hispanic Black	1992	3,055	2,422	3,635	37	1104.49	875.51	1314.17	1
		1993	3,233	2,386	4,528	29	1124.72	829.89	1575.23	1
		1994	2,529	2,316	2,757	42	840.63	769.87	916.40	2
		1995	2,688	2,217	3,941	34	853.22	703.75	1250.94	2
		1996	2,466	1,966	3,652	36	748.22	596.38	1107.91	2
		1997	2,271	1,721	3,346	35	670.53	508.05	988.07	3
		1998	2,119	1,545	3,052	38	605.18	441.36	871.66	3
		1999	1,721	1,387	1,934	51	478.78	385.82	537.89	5
		2000	1,896	1,368	2,512	43	510.95	368.68	676.71	4
		2001	1,684	1,372	1,850	49	441.50	359.86	485.06	7
		2002	1,778	1,473	2,001	44	453.21	375.50	510.13	5
		2003	1,747	1,587	1,859	42	436.65	396.52	464.68	5
		2004	1,746	1,605	1,907	41	429.94	395.36	469.69	7
		2005	1,787	1,461	2,019	37	428.64	350.38	484.18	7
		2006	1,883	1,338	2,206	36	437.20	310.47	512.11	7
		2007	2,046	1,225	2,598	34	461.59	276.23	585.94	7
	Hispanic	1992	835	662	993	43	201.09	159.40	239.27	32
		1993	902	666	1,263	39	194.22	143.31	272.02	34
		1994	736	674	803	45	141.96	130.01	154.76	39
		1995	831	686	1,219	43	142.64	117.65	209.13	38
		1996	822	656	1,218	44	127.07	101.28	188.15	39
		1997	825	625	1,216	45	114.19	86.52	168.26	40
		1998	844	616	1,216	46	106.12	77.40	152.85	40
		1999	753	607	846	49	86.12	69.40	96.76	53
		2000	911	657	1,206	46	96.05	69.31	127.22	45
		2001	882	719	969	46	86.77	70.73	95.33	47
		2002	1,007	834	1,134	46	93.38	77.37	105.11	44
		2003	1,056	959	1,124	47	93.72	85.11	99.74	43
		2004	1,108	1,019	1,210	47	94.25	86.67	102.97	42
		2005	1,166	953	1,318	48	93.99	76.83	106.17	42
		2006	1,235	877	1,446	45	94.19	66.89	110.33	42
		2007	1,310	784	1,663	44	94.49	56.55	119.95	40

Metropolitan Statistical Area	PWID Population	Year	Number	Min	Max	Rank	Rate	Min	Max	Rank
Portland--Vancouver, OR--WA	Male	1992	12,287	9,739	14,619	25	229.41	181.85	272.96	24
		1993	14,035	10,356	19,657	17	253.18	186.81	354.59	14
		1994	11,881	10,881	12,952	24	208.53	190.98	227.33	27
		1995	13,682	11,285	20,060	16	233.17	192.32	341.86	15
		1996	13,595	10,836	20,130	16	223.94	178.50	331.60	16
		1997	13,521	10,245	19,924	16	216.53	164.06	319.07	16
		1998	13,562	9,891	19,534	16	212.29	154.82	305.77	17
		1999	11,755	9,472	13,206	19	180.27	145.27	202.53	31
		2000	13,691	9,879	18,133	17	205.97	148.62	272.78	16
		2001	12,694	10,347	13,946	18	186.52	152.03	204.92	24
		2002	13,797	11,431	15,530	17	198.52	164.48	223.46	18
		2003	13,725	12,464	14,606	16	194.85	176.95	207.36	18
		2004	13,613	12,518	14,872	16	191.21	175.83	208.88	17
		2005	13,524	11,055	15,277	16	186.47	152.43	210.63	17
		2006	13,486	9,577	15,797	16	182.20	129.39	213.42	19
		2007	13,467	8,059	17,095	17	178.54	106.85	226.64	20
	Female	1992	7,693	6,098	9,153	18	143.75	113.95	171.04	14
		1993	8,835	6,519	12,375	15	160.02	118.07	224.12	5
		1994	7,546	6,911	8,226	17	133.27	122.06	145.29	16
		1995	8,787	7,247	12,883	17	151.02	124.56	221.41	8
		1996	8,840	7,046	13,090	16	147.17	117.31	217.92	9
		1997	8,909	6,750	13,127	16	144.44	109.44	212.85	8
		1998	9,053	6,602	13,039	16	143.72	104.81	207.00	8
		1999	7,942	6,400	8,923	17	123.84	99.80	139.13	13
		2000	9,349	6,746	12,382	12	143.31	103.41	189.80	8
		2001	8,741	7,125	9,603	14	130.79	106.61	143.70	9
		2002	9,552	7,914	10,752	13	139.98	115.98	157.56	8
		2003	9,517	8,643	10,128	13	137.59	124.94	146.42	8
		2004	9,412	8,655	10,283	13	134.54	123.72	146.98	8
		2005	9,274	7,580	10,475	13	130.19	106.42	147.06	8
		2006	9,115	6,473	10,676	13	125.35	89.02	146.83	12
		2007	8,908	5,331	11,308	13	120.19	71.93	152.57	14

Metropolitan Statistical Area	PWID Population	Year	Number	Min	Max	Rank	Rate	Min	Max	Rank
Portland--Vancouver, OR--WA	Young (15-29)	1992	7,380	5,850	8,781	14	222.14	176.08	264.31	7
		1993	8,033	5,927	11,250	9	236.69	174.65	331.50	4
		1994	6,572	6,019	7,164	12	189.79	173.82	206.90	8
		1995	7,404	6,107	10,856	9	207.82	171.41	304.68	3
		1996	7,272	5,796	10,768	9	197.00	157.02	291.70	4
		1997	7,209	5,462	10,623	8	189.07	143.25	278.60	5
		1998	7,255	5,291	10,449	8	185.20	135.06	266.74	6
		1999	6,336	5,106	7,119	12	158.38	127.63	177.93	16
		2000	7,457	5,381	9,876	8	183.51	132.41	243.04	11
		2001	6,991	5,699	7,681	12	170.55	139.01	187.37	15
		2002	7,676	6,359	8,639	11	185.38	153.60	208.67	12
		2003	7,689	6,982	8,183	11	185.22	168.19	197.10	14
		2004	7,643	7,028	8,350	13	184.40	169.57	201.45	16
		2005	7,559	6,179	8,538	13	180.67	147.68	204.08	16
		2006	7,439	5,283	8,714	14	175.95	124.95	206.10	16
		2007	7,256	4,342	9,210	14	169.75	101.59	215.49	18
	Old (30-64)	1992	14,373	11,393	17,102	24	194.63	154.28	231.58	22
		1993	16,282	12,014	22,804	17	212.25	156.61	297.27	16
		1994	13,713	12,559	14,949	22	173.66	159.04	189.31	23
		1995	15,752	12,992	23,094	17	193.91	159.94	284.29	17
		1996	15,641	12,467	23,160	16	186.51	148.66	276.17	17
		1997	15,564	11,792	22,934	17	181.00	137.14	266.71	18
		1998	15,628	11,398	22,510	17	178.19	129.96	256.66	15
		1999	13,558	10,926	15,232	19	151.78	122.31	170.52	25
		2000	15,798	11,399	20,923	16	173.47	125.17	229.74	14
		2001	14,637	11,930	16,081	18	155.88	127.06	171.26	20
		2002	15,872	13,151	17,866	18	164.77	136.51	185.46	13
		2003	15,721	14,276	16,730	18	160.26	145.53	170.55	12
		2004	15,486	14,240	16,918	18	155.31	142.82	169.67	13
		2005	15,229	12,449	17,202	18	149.42	122.14	168.78	13
		2006	14,971	10,632	17,536	18	143.33	101.79	167.89	12
		2007	14,662	8,774	18,612	17	137.28	82.15	174.26	12

Metropolitan Statistical Area	PWID Population	Year	Number	Min	Max	Rank	Rate	Min	Max	Rank
Providence--Fall River--Warwick, RI--MA	Total	1992	4,493	3,301	5,346	81	74.50	54.74	88.64	77
		1993	5,956	3,148	10,151	68	98.91	52.28	168.58	51
		1994	4,654	3,039	5,539	80	77.16	50.40	91.85	75
		1995	6,693	2,931	12,519	61	110.79	48.52	207.24	45
		1996	7,080	2,878	13,661	59	116.50	47.35	224.78	42
		1997	7,448	2,827	14,700	52	121.67	46.18	240.14	39
		1998	7,863	2,815	15,824	50	126.95	45.46	255.49	34
		1999	5,376	2,795	7,067	76	86.05	44.74	113.12	59
		2000	8,578	2,942	17,398	46	135.35	46.42	274.51	26
		2001	5,902	3,090	7,945	73	91.89	48.11	123.69	56
		2002	8,894	3,186	17,108	46	136.54	48.92	262.64	23
		2003	9,024	3,275	16,906	46	137.03	49.73	256.70	23
		2004	8,923	3,381	15,830	46	134.66	51.03	238.91	24
		2005	8,771	3,473	14,633	47	132.15	52.33	220.46	23
		2006	8,592	3,546	13,399	49	129.54	53.46	202.01	23
		2007	8,398	3,620	12,090	49	126.62	54.59	182.29	25
	Non-Hispanic White	1992	3,184	2,339	3,788	68	59.78	43.92	71.13	65
		1993	4,216	2,228	7,186	52	79.86	42.21	136.11	46
		1994	3,287	2,147	3,912	68	62.63	40.91	74.55	58
		1995	4,710	2,063	8,811	47	90.32	39.55	168.94	40
		1996	4,960	2,016	9,570	38	95.28	38.73	183.83	37
		1997	5,187	1,969	10,238	36	99.77	37.87	196.92	33
		1998	5,437	1,947	10,942	36	104.27	37.34	209.84	29
		1999	3,687	1,917	4,847	61	70.66	36.74	92.89	57
		2000	5,827	1,998	11,817	36	110.98	38.06	225.09	24
		2001	3,966	2,076	5,338	57	75.04	39.29	101.01	55
		2002	5,903	2,115	11,355	36	110.91	39.73	213.34	23
		2003	5,908	2,144	11,069	35	110.50	40.10	207.01	27
		2004	5,755	2,181	10,209	36	107.56	40.76	190.83	28
		2005	5,564	2,203	9,283	38	104.47	41.37	174.29	31
		2006	5,354	2,209	8,349	40	101.13	41.73	157.71	33
		2007	5,133	2,213	7,390	43	97.50	42.04	140.37	39

Metropolitan Statistical Area	PWID Population	Year	Number	Min	Max	Rank	Rate	Min	Max	Rank
Providence--Fall River--Warwick, RI--MA	Non-Hispanic Black	1992	736	541	876	86	328.41	241.28	390.73	44
		1993	947	501	1,614	77	411.93	217.71	702.03	20
		1994	702	459	836	85	293.23	191.52	349.02	44
		1995	941	412	1,760	69	379.15	166.05	709.22	19
		1996	915	372	1,765	69	352.00	143.07	679.18	20
		1997	876	333	1,730	69	322.85	122.55	637.22	20
		1998	839	301	1,689	69	294.31	105.39	592.30	20
		1999	522	271	686	86	175.84	91.42	231.16	62
		2000	762	261	1,546	71	246.83	84.66	500.62	26
		2001	487	255	655	85	152.14	79.65	204.78	65
		2002	694	249	1,335	73	209.46	75.04	402.91	33
		2003	683	248	1,280	73	200.31	72.70	375.27	33
		2004	677	257	1,201	74	193.01	73.14	342.43	34
		2005	692	274	1,155	74	193.70	76.70	323.15	34
		2006	738	304	1,150	75	203.84	84.12	317.88	32
		2007	824	355	1,186	73	223.22	96.24	321.36	27
	Hispanic	1992	222	163	264	66	66.48	48.85	79.10	74
		1993	389	206	663	56	108.74	57.47	185.31	52
		1994	370	241	440	57	96.94	63.32	115.39	55
		1995	602	264	1,126	49	147.83	64.74	276.52	36
		1996	681	277	1,315	49	156.86	63.76	302.66	34
		1997	732	278	1,444	48	157.17	59.66	310.20	32
		1998	761	273	1,532	47	152.86	54.74	307.63	32
		1999	500	260	658	56	94.07	48.91	123.67	44
		2000	756	259	1,534	49	133.30	45.72	270.35	29
		2001	491	257	661	56	81.62	42.73	109.86	52
		2002	703	252	1,351	53	110.50	39.59	212.55	35
		2003	690	250	1,292	53	103.24	37.47	193.40	39
		2004	679	257	1,205	55	98.15	37.19	174.13	41
		2005	693	275	1,157	55	96.68	38.28	161.29	41
		2006	743	307	1,159	53	100.40	41.43	156.56	39
		2007	847	365	1,219	53	111.33	48.00	160.27	32

Metropolitan Statistical Area	PWID Population	Year	Number	Min	Max	Rank	Rate	Min	Max	Rank
Providence--Fall River--Warwick, RI--MA	Male	1992	2,950	2,167	3,509	80	99.69	73.24	118.61	80
		1993	3,940	2,083	6,716	67	133.39	70.50	227.33	49
		1994	3,094	2,021	3,683	80	104.56	68.29	124.46	73
		1995	4,463	1,954	8,348	58	150.70	66.00	281.89	42
		1996	4,727	1,921	9,120	53	158.76	64.53	306.32	40
		1997	4,971	1,887	9,812	48	165.79	62.93	327.22	36
		1998	5,244	1,878	10,553	46	172.93	61.92	348.02	34
		1999	3,581	1,862	4,707	73	117.08	60.87	153.92	57
		2000	5,707	1,957	11,575	43	184.05	63.13	373.29	23
		2001	3,925	2,055	5,283	69	124.73	65.30	167.88	52
		2002	5,917	2,120	11,382	43	185.18	66.34	356.20	21
		2003	6,016	2,183	11,271	42	186.10	67.54	348.63	20
		2004	5,972	2,263	10,595	43	183.19	69.42	325.00	19
		2005	5,908	2,339	9,856	43	180.76	71.57	301.56	19
		2006	5,839	2,410	9,106	45	178.74	73.76	278.73	21
		2007	5,776	2,490	8,315	45	176.58	76.13	254.22	21
	Female	1992	1,512	1,111	1,799	78	49.20	36.15	58.54	75
		1993	2,006	1,060	3,418	67	65.38	34.55	111.43	52
		1994	1,571	1,026	1,870	81	51.16	33.41	60.89	77
		1995	2,269	994	4,245	63	73.69	32.27	137.85	46
		1996	2,413	981	4,655	59	77.82	31.63	150.15	44
		1997	2,551	968	5,034	57	81.67	31.00	161.20	43
		1998	2,704	968	5,443	53	85.55	30.63	172.16	35
		1999	1,855	964	2,438	78	58.16	30.24	76.45	64
		2000	2,963	1,016	6,010	48	91.55	31.40	185.67	27
		2001	2,036	1,066	2,741	73	62.15	32.54	83.65	58
		2002	3,054	1,094	5,875	48	92.05	32.98	177.06	26
		2003	3,073	1,115	5,758	47	91.66	33.26	171.71	25
		2004	3,000	1,137	5,322	49	89.11	33.77	158.10	24
		2005	2,895	1,146	4,830	51	85.93	34.03	143.36	26
		2006	2,768	1,142	4,316	54	82.24	33.94	128.25	28
		2007	2,622	1,131	3,775	59	78.01	33.63	112.31	33

Metropolitan Statistical Area	PWID Population	Year	Number	Min	Max	Rank	Rate	Min	Max	Rank
Providence--Fall River--Warwick, RI--MA	Young (15-29)	1992	1,239	910	1,474	78	58.58	43.04	69.69	73
		1993	1,735	917	2,957	57	83.68	44.23	142.61	48
		1994	1,427	932	1,699	75	69.92	45.67	83.23	59
		1995	2,149	941	4,019	47	106.72	46.74	199.63	35
		1996	2,365	961	4,563	45	118.46	48.15	228.57	29
		1997	2,569	975	5,071	40	129.22	49.05	255.04	22
		1998	2,777	995	5,589	38	139.84	50.07	281.42	17
		1999	1,926	1,001	2,532	63	97.63	50.76	128.35	43
		2000	3,085	1,058	6,257	37	156.39	53.64	317.19	16
		2001	2,105	1,102	2,834	63	105.77	55.37	142.36	41
		2002	3,104	1,112	5,971	40	154.06	55.19	296.33	19
		2003	3,036	1,102	5,687	42	148.62	53.94	278.43	23
		2004	2,843	1,077	5,044	46	137.77	52.21	244.42	25
		2005	2,595	1,028	4,329	56	124.71	49.38	208.05	28
		2006	2,307	952	3,598	64	110.43	45.57	172.21	39
		2007	1,994	860	2,871	71	94.96	40.94	136.70	51
	Old (30-64)	1992	3,331	2,448	3,964	80	85.06	62.49	101.20	76
		1993	4,292	2,269	7,315	67	108.73	57.46	185.30	49
		1994	3,271	2,136	3,894	81	81.98	53.55	97.58	80
		1995	4,607	2,018	8,618	65	114.40	50.10	213.98	46
		1996	4,794	1,949	9,251	64	117.47	47.75	226.66	44
		1997	4,983	1,891	9,835	61	120.55	45.76	237.93	41
		1998	5,220	1,869	10,505	58	124.07	44.43	249.69	40
		1999	3,556	1,849	4,675	78	83.20	43.26	109.38	64
		2000	5,678	1,947	11,515	48	130.06	44.61	263.79	35
		2001	3,923	2,054	5,280	75	88.50	46.33	119.11	56
		2002	5,956	2,134	11,456	44	132.38	47.43	254.64	26
		2003	6,107	2,216	11,441	43	134.42	48.78	251.82	24
		2004	6,118	2,318	10,854	42	134.10	50.81	237.91	22
		2005	6,106	2,418	10,187	43	134.01	53.07	223.58	17
		2006	6,082	2,510	9,484	41	133.86	55.24	208.74	14
		2007	6,045	2,606	8,702	42	133.38	57.50	192.01	14

Metropolitan Statistical Area	PWID Population	Year	Number	Min	Max	Rank	Rate	Min	Max	Rank
Raleigh--Durham--Chapel Hill, NC	Total	1992	6,654	6,101	7,052	64	101.99	93.52	108.09	53
		1993	5,363	1,706	6,766	74	79.56	25.30	100.37	68
		1994	6,503	6,374	6,689	64	93.28	91.44	95.95	56
		1995	5,468	2,585	6,586	71	75.67	35.77	91.15	70
		1996	5,505	2,991	6,742	71	73.64	40.00	90.19	70
		1997	5,419	3,360	6,939	72	70.13	43.49	89.81	69
		1998	5,296	3,587	7,157	71	66.33	44.92	89.63	71
		1999	5,607	4,419	7,355	73	68.18	53.74	89.44	75
		2000	4,855	3,248	7,658	80	57.45	38.43	90.61	79
		2001	5,315	3,390	8,011	79	60.90	38.84	91.78	80
		2002	4,708	2,879	8,400	82	52.55	32.14	93.77	85
		2003	4,751	2,955	8,766	82	51.90	32.28	95.76	90
		2004	4,948	3,161	9,201	78	52.79	33.72	98.17	90
		2005	5,187	3,380	9,723	77	53.65	34.96	100.57	92
		2006	5,482	3,463	10,326	75	54.63	34.52	102.92	91
		2007	5,798	3,285	10,960	75	55.68	31.55	105.26	88
	Non-Hispanic White	1992	2,857	2,620	3,028	71	60.16	55.17	63.76	62
		1993	2,099	668	2,648	78	43.01	13.68	54.26	80
		1994	2,389	2,342	2,457	77	47.66	46.72	49.02	76
		1995	1,942	918	2,339	81	37.63	17.79	45.32	83
		1996	1,940	1,054	2,377	82	36.68	19.92	44.92	83
		1997	1,940	1,203	2,484	82	35.82	22.21	45.87	84
		1998	1,962	1,329	2,651	84	35.40	23.97	47.83	84
		1999	2,178	1,716	2,857	83	38.59	30.41	50.62	85
		2000	1,994	1,334	3,146	84	34.83	23.30	54.93	86
		2001	2,319	1,479	3,495	83	39.44	25.16	59.45	86
		2002	2,181	1,334	3,891	83	36.38	22.25	64.92	88
		2003	2,329	1,448	4,296	80	38.25	23.79	70.57	87
		2004	2,550	1,629	4,742	79	41.18	26.31	76.58	87
		2005	2,787	1,816	5,225	78	43.91	28.62	82.31	85
		2006	3,042	1,922	5,730	76	46.42	29.33	87.46	84
		2007	3,285	1,862	6,210	75	48.56	27.52	91.80	80

Metropolitan Statistical Area	PWID Population	Year	Number	Min	Max	Rank	Rate	Min	Max	Rank
Raleigh--Durham--Chapel Hill, NC	Non-Hispanic Black	1992	3,427	3,143	3,632	29	227.35	208.47	240.94	68
		1993	2,992	951	3,774	32	192.49	61.21	242.85	71
		1994	3,797	3,722	3,906	27	237.14	232.45	243.91	60
		1995	3,258	1,540	3,924	29	197.29	93.26	237.65	63
		1996	3,278	1,781	4,015	28	192.98	104.83	236.35	57
		1997	3,168	1,965	4,058	27	181.57	112.60	232.54	59
		1998	2,992	2,027	4,043	28	167.55	113.47	226.39	62
		1999	3,015	2,376	3,956	25	165.45	130.40	217.05	66
		2000	2,452	1,640	3,867	32	131.51	87.98	207.44	73
		2001	2,491	1,588	3,754	30	129.86	82.82	195.71	72
		2002	2,029	1,241	3,620	36	102.99	62.98	183.76	76
		2003	1,874	1,165	3,457	38	92.92	57.80	171.44	82
		2004	1,787	1,142	3,323	39	86.22	55.07	160.32	83
		2005	1,729	1,126	3,240	41	80.72	52.60	151.30	83
		2006	1,711	1,081	3,223	45	77.02	48.67	145.10	81
		2007	1,733	982	3,276	47	75.32	42.68	142.39	83
	Hispanic	1992	31	29	33	91	27.07	24.82	28.69	96
		1993	28	9	35	91	20.08	6.38	25.33	96
		1994	38	38	39	89	22.27	21.83	22.91	96
		1995	37	18	45	88	17.74	8.39	21.37	96
		1996	44	24	54	88	17.00	9.24	20.82	96
		1997	52	32	67	87	16.22	10.06	20.78	96
		1998	61	41	83	85	15.63	10.58	21.12	94
		1999	78	61	102	83	16.60	13.08	21.77	94
		2000	81	54	128	83	14.74	9.86	23.25	94
		2001	105	67	159	79	17.72	11.30	26.70	94
		2002	109	67	195	79	17.17	10.50	30.63	93
		2003	127	79	235	76	18.92	11.77	34.91	92
		2004	149	95	278	75	21.05	13.45	39.14	91
		2005	172	112	323	75	22.80	14.86	42.73	87
		2006	194	123	366	74	23.94	15.12	45.09	87
		2007	212	120	401	72	24.38	13.82	46.09	89

Metropolitan Statistical Area	PWID Population	Year	Number	Min	Max	Rank	Rate	Min	Max	Rank
Raleigh--Durham--Chapel Hill, NC	Male	1992	4,415	4,048	4,678	64	137.56	126.13	145.78	52
		1993	3,533	1,123	4,457	73	106.65	33.92	134.56	65
		1994	4,256	4,172	4,377	65	124.07	121.62	127.62	53
		1995	3,558	1,682	4,286	71	99.97	47.26	120.42	67
		1996	3,564	1,936	4,364	72	96.71	52.54	118.44	69
		1997	3,491	2,165	4,471	73	91.49	56.74	117.17	72
		1998	3,397	2,300	4,590	74	86.06	58.29	116.29	73
		1999	3,580	2,822	4,697	74	87.83	69.23	115.22	74
		2000	3,087	2,065	4,869	82	73.32	49.05	115.65	79
		2001	3,364	2,146	5,070	77	77.40	49.36	116.65	80
		2002	2,965	1,814	5,291	83	66.49	40.66	118.64	87
		2003	2,977	1,851	5,492	84	65.48	40.73	120.81	88
		2004	3,081	1,968	5,729	81	66.13	42.24	122.96	89
		2005	3,207	2,090	6,012	78	66.76	43.51	125.14	88
		2006	3,362	2,125	6,334	74	67.33	42.54	126.84	89
		2007	3,523	1,997	6,661	74	68.18	38.64	128.90	87
	Female	1992	2,329	2,135	2,468	65	70.26	64.42	74.46	51
		1993	1,892	602	2,387	69	55.17	17.55	69.61	64
		1994	2,314	2,268	2,380	66	65.34	64.05	67.20	59
		1995	1,963	928	2,365	72	53.53	25.31	64.48	66
		1996	1,995	1,084	2,443	69	52.62	28.59	64.45	68
		1997	1,982	1,229	2,538	71	50.67	31.42	64.89	71
		1998	1,955	1,324	2,641	75	48.41	32.79	65.41	74
		1999	2,087	1,645	2,738	71	50.33	39.67	66.03	73
		2000	1,822	1,219	2,874	77	42.95	28.73	67.75	80
		2001	2,009	1,281	3,028	75	45.84	29.24	69.09	85
		2002	1,790	1,095	3,194	79	39.79	24.33	70.99	87
		2003	1,815	1,129	3,349	78	39.38	24.49	72.66	87
		2004	1,897	1,212	3,527	79	40.24	25.70	74.82	87
		2005	1,991	1,298	3,733	77	40.95	26.68	76.75	85
		2006	2,104	1,329	3,964	73	41.75	26.38	78.65	85
		2007	2,221	1,258	4,198	72	42.34	23.99	80.03	82

Metropolitan Statistical Area	PWID Population	Year	Number	Min	Max	Rank	Rate	Min	Max	Rank
Raleigh--Durham--Chapel Hill, NC	Young (15-29)	1992	1,937	1,776	2,053	56	79.37	72.78	84.12	56
		1993	1,405	447	1,773	72	56.73	18.04	71.57	66
		1994	1,572	1,541	1,617	69	62.35	61.12	64.14	67
		1995	1,249	591	1,505	74	48.39	22.88	58.29	75
		1996	1,216	661	1,489	76	46.04	25.01	56.38	80
		1997	1,181	732	1,513	78	43.66	27.07	55.91	81
		1998	1,159	785	1,567	82	41.95	28.41	56.68	84
		1999	1,251	986	1,641	83	44.46	35.05	58.33	87
		2000	1,117	747	1,761	86	39.21	26.23	61.85	92
		2001	1,271	811	1,915	85	44.15	28.16	66.54	94
		2002	1,176	719	2,099	87	40.54	24.80	72.34	98
		2003	1,243	773	2,293	88	42.82	26.63	79.01	98
		2004	1,354	865	2,518	88	46.16	29.49	85.83	99
		2005	1,479	964	2,773	83	49.33	32.14	92.46	98
		2006	1,619	1,023	3,050	81	52.14	32.94	98.22	95
		2007	1,758	996	3,324	80	55.08	31.21	104.13	92
	Old (30-64)	1992	4,820	4,420	5,108	64	118.05	108.24	125.10	49
		1993	4,004	1,273	5,051	71	93.89	29.86	118.45	63
		1994	4,966	4,868	5,108	62	111.61	109.40	114.79	52
		1995	4,247	2,008	5,116	68	91.44	43.23	110.15	65
		1996	4,326	2,350	5,299	67	89.50	48.62	109.61	62
		1997	4,289	2,660	5,492	67	85.40	52.96	109.38	64
		1998	4,203	2,847	5,679	69	80.50	54.52	108.78	65
		1999	4,442	3,501	5,828	69	82.10	64.71	107.71	66
		2000	3,824	2,558	6,031	73	68.23	45.64	107.63	72
		2001	4,143	2,642	6,243	73	70.82	45.16	106.73	75
		2002	3,613	2,210	6,447	75	59.65	36.48	106.43	77
		2003	3,572	2,222	6,590	75	57.13	35.53	105.40	76
		2004	3,622	2,313	6,734	74	56.24	35.92	104.57	76
		2005	3,669	2,391	6,877	72	55.02	35.86	103.14	76
		2006	3,714	2,347	6,997	68	53.61	33.87	100.99	76
		2007	3,722	2,109	7,035	66	51.54	29.21	97.44	74

Metropolitan Statistical Area	PWID Population	Year	Number	Min	Max	Rank	Rate	Min	Max	Rank
Richmond--Petersburg, VA	Total	1992	8,085	6,312	9,634	56	132.85	103.71	158.30	43
		1993	6,803	3,085	9,620	60	110.19	49.96	155.83	46
		1994	7,988	6,232	9,588	56	127.61	99.56	153.17	41
		1995	6,801	3,438	9,541	60	107.29	54.23	150.52	48
		1996	6,793	3,607	9,489	61	105.76	56.16	147.74	46
		1997	6,784	3,759	9,441	63	104.16	57.71	144.96	47
		1998	6,761	3,906	9,370	60	102.57	59.26	142.14	47
		1999	7,640	5,927	9,304	54	114.40	88.76	139.32	41
		2000	6,777	4,364	9,350	58	99.99	64.39	137.94	49
		2001	7,507	5,616	9,377	57	109.32	81.78	136.57	46
		2002	6,768	4,592	9,607	58	96.98	65.80	137.65	48
		2003	6,781	4,712	9,829	56	95.71	66.50	138.73	47
		2004	6,790	4,817	10,062	56	94.38	66.96	139.87	48
		2005	6,810	4,720	10,323	56	93.02	64.47	141.00	50
		2006	6,840	4,468	10,633	60	91.73	59.92	142.60	50
		2007	6,836	4,186	10,903	61	90.42	55.36	144.20	53
	Non-Hispanic White	1992	2,011	1,570	2,397	82	48.55	37.90	57.85	77
		1993	1,688	765	2,387	86	40.37	18.31	57.10	82
		1994	2,005	1,564	2,407	82	47.55	37.10	57.07	77
		1995	1,748	884	2,452	86	41.08	20.76	57.63	80
		1996	1,805	958	2,521	85	42.10	22.36	58.81	79
		1997	1,876	1,040	2,612	85	43.53	24.12	60.59	76
		1998	1,957	1,131	2,713	85	45.27	26.16	62.74	74
		1999	2,322	1,801	2,828	80	53.44	41.46	65.08	69
		2000	2,165	1,394	2,986	82	49.32	31.76	68.04	72
		2001	2,518	1,883	3,145	78	56.93	42.59	71.12	68
		2002	2,378	1,613	3,375	79	53.24	36.12	75.57	68
		2003	2,486	1,727	3,603	79	55.13	38.31	79.92	67
		2004	2,584	1,833	3,829	78	56.79	40.29	84.16	68
		2005	2,673	1,853	4,052	79	58.04	40.23	87.98	67
		2006	2,749	1,796	4,273	80	58.93	38.49	91.60	67
		2007	2,790	1,708	4,450	79	59.31	36.31	94.59	65

Metropolitan Statistical Area	PWID Population	Year	Number	Min	Max	Rank	Rate	Min	Max	Rank
Richmond--Petersburg, VA	Non-Hispanic Black	1992	5,153	4,023	6,140	20	294.64	230.02	351.10	51
		1993	4,489	2,035	6,347	22	251.66	114.11	355.88	53
		1994	5,365	4,186	6,440	20	295.18	230.30	354.31	43
		1995	4,589	2,319	6,437	22	249.14	125.93	349.51	51
		1996	4,553	2,418	6,360	21	242.94	129.01	339.36	48
		1997	4,475	2,479	6,227	20	233.25	129.24	324.62	46
		1998	4,352	2,514	6,031	19	221.91	128.21	307.53	45
		1999	4,764	3,696	5,802	18	238.15	184.77	290.02	33
		2000	4,070	2,621	5,616	18	200.77	129.29	276.99	41
		2001	4,325	3,235	5,402	18	210.23	157.27	262.62	34
		2002	3,734	2,534	5,300	18	177.75	120.60	252.29	43
		2003	3,588	2,493	5,201	20	167.98	116.72	243.48	44
		2004	3,463	2,457	5,132	21	159.02	112.82	235.64	47
		2005	3,378	2,342	5,121	22	152.20	105.49	230.72	49
		2006	3,343	2,184	5,197	22	147.84	96.56	229.82	52
		2007	3,344	2,048	5,334	22	145.52	89.10	232.08	54
	Hispanic	1992	85	67	102	77	113.29	88.44	135.00	57
		1993	72	33	102	80	87.41	39.64	123.61	59
		1994	87	67	104	79	95.69	74.65	114.85	56
		1995	77	39	107	79	77.27	39.06	108.40	58
		1996	80	43	112	79	73.36	38.96	102.48	59
		1997	85	47	119	79	70.19	38.89	97.68	60
		1998	91	53	126	79	67.28	38.87	93.24	59
		1999	110	86	134	76	73.67	57.16	89.72	59
		2000	105	68	145	76	62.63	40.33	86.41	62
		2001	125	93	156	76	67.70	50.64	84.57	60
		2002	120	81	170	76	59.75	40.54	84.80	62
		2003	127	88	184	76	58.18	40.42	84.33	61
		2004	133	94	197	77	56.92	40.39	84.35	61
		2005	138	95	209	78	54.29	37.63	82.30	62
		2006	141	92	219	79	51.01	33.32	79.29	63
		2007	141	87	226	82	47.90	29.33	76.39	64

Metropolitan Statistical Area	PWID Population	Year	Number	Min	Max	Rank	Rate	Min	Max	Rank
Richmond--Petersburg, VA	Male	1992	4,924	3,844	5,868	57	167.24	130.56	199.28	40
		1993	4,268	1,935	6,036	63	142.67	64.69	201.75	44
		1994	5,097	3,976	6,117	56	168.08	131.13	201.75	37
		1995	4,368	2,208	6,128	61	142.30	71.93	199.63	43
		1996	4,357	2,314	6,086	61	140.19	74.44	195.82	44
		1997	4,317	2,392	6,008	60	136.90	75.85	190.53	45
		1998	4,248	2,454	5,887	61	132.89	76.77	184.16	45
		1999	4,724	3,665	5,753	58	145.73	113.06	177.47	39
		2000	4,117	2,651	5,680	61	124.91	80.44	172.33	47
		2001	4,481	3,352	5,597	60	134.09	100.31	167.51	45
		2002	3,979	2,699	5,647	65	117.03	79.40	166.10	51
		2003	3,943	2,740	5,715	65	113.99	79.21	165.23	55
		2004	3,931	2,789	5,826	64	111.94	79.41	165.87	54
		2005	3,961	2,745	6,004	65	110.80	76.80	167.96	55
		2006	4,039	2,638	6,279	63	110.66	72.28	172.02	55
		2007	4,147	2,539	6,614	61	111.91	68.52	178.48	53
	Female	1992	3,002	2,343	3,577	48	95.55	74.60	113.86	38
		1993	2,467	1,118	3,488	59	77.52	35.15	109.63	40
		1994	2,860	2,231	3,432	53	88.61	69.13	106.36	36
		1995	2,428	1,227	3,406	58	74.25	37.53	104.17	45
		1996	2,437	1,294	3,404	58	73.50	39.03	102.67	49
		1997	2,461	1,363	3,425	58	73.24	40.58	101.94	49
		1998	2,491	1,439	3,452	59	73.38	42.40	101.69	51
		1999	2,867	2,225	3,492	57	83.44	64.74	101.62	42
		2000	2,594	1,670	3,578	57	74.48	47.96	102.75	47
		2001	2,927	2,190	3,657	55	83.04	62.12	103.74	38
		2002	2,683	1,821	3,809	55	74.97	50.87	106.41	41
		2003	2,724	1,892	3,948	54	75.11	52.19	108.87	42
		2004	2,749	1,950	4,073	54	74.65	52.96	110.62	40
		2005	2,760	1,913	4,184	57	73.67	51.06	111.67	42
		2006	2,753	1,798	4,280	55	72.33	47.24	112.44	40
		2007	2,706	1,657	4,316	55	70.21	42.99	111.96	44

Metropolitan Statistical Area	PWID Population	Year	Number	Min	Max	Rank	Rate	Min	Max	Rank
Richmond--Petersburg, VA	Young (15-29)	1992	1,712	1,336	2,040	65	86.37	67.43	102.92	54
		1993	1,478	670	2,090	68	75.19	34.09	106.33	54
		1994	1,809	1,411	2,171	61	92.83	72.42	111.42	41
		1995	1,624	821	2,278	63	83.74	42.33	117.47	53
		1996	1,722	914	2,405	61	88.84	47.18	124.10	49
		1997	1,831	1,014	2,548	60	93.60	51.86	130.26	45
		1998	1,941	1,122	2,690	59	98.46	56.88	136.44	43
		1999	2,323	1,803	2,829	52	117.55	91.21	143.16	34
		2000	2,166	1,395	2,988	54	108.60	69.94	149.83	38
		2001	2,495	1,866	3,116	52	124.28	92.97	155.25	33
		2002	2,308	1,566	3,275	54	113.23	76.82	160.71	41
		2003	2,333	1,621	3,382	57	112.22	77.97	162.66	42
		2004	2,313	1,641	3,427	62	108.89	77.26	161.37	44
		2005	2,244	1,555	3,402	67	102.31	70.91	155.09	49
		2006	2,122	1,386	3,298	69	93.80	61.27	145.82	52
		2007	1,932	1,183	3,081	74	83.56	51.16	133.26	54
	Old (30-64)	1992	6,360	4,965	7,579	53	154.97	120.98	184.66	39
		1993	5,300	2,403	7,495	57	125.95	57.11	178.11	43
		1994	6,144	4,794	7,375	52	142.53	111.20	171.07	40
		1995	5,152	2,604	7,228	58	117.09	59.18	164.26	44
		1996	5,057	2,685	7,063	60	112.75	59.87	157.50	45
		1997	4,954	2,745	6,895	62	108.72	60.24	151.30	47
		1998	4,839	2,796	6,706	62	104.74	60.51	145.14	48
		1999	5,358	4,157	6,525	55	113.95	88.41	138.78	43
		2000	4,662	3,002	6,432	60	97.46	62.76	134.46	53
		2001	5,079	3,799	6,344	57	104.52	78.19	130.56	48
		2002	4,522	3,068	6,418	61	91.52	62.09	129.90	53
		2003	4,500	3,127	6,523	59	89.90	62.46	130.30	51
		2004	4,508	3,198	6,680	60	88.91	63.08	131.76	48
		2005	4,561	3,161	6,914	56	88.95	61.65	134.83	46
		2006	4,663	3,045	7,248	55	89.75	58.62	139.52	43
		2007	4,782	2,928	7,626	53	91.10	55.78	145.29	38

Metropolitan Statistical Area	PWID Population	Year	Number	Min	Max	Rank	Rate	Min	Max	Rank
Riverside--San Bernardino, CA	Total	1992	29,526	16,834	46,386	8	167.55	95.53	263.23	24
		1993	32,322	14,901	46,844	8	181.84	83.83	263.54	19
		1994	25,513	13,124	38,406	11	142.26	73.18	214.15	35
		1995	28,157	11,447	41,748	9	155.04	63.03	229.88	25
		1996	26,589	11,830	39,057	11	144.71	64.38	212.56	29
		1997	25,497	13,315	36,641	13	135.51	70.76	194.74	30
		1998	24,818	14,414	34,277	13	128.10	74.40	176.92	31
		1999	21,238	14,539	25,795	16	106.09	72.63	128.85	45
		2000	22,848	13,730	27,939	15	110.46	66.38	135.08	42
		2001	20,840	11,946	26,847	18	96.91	55.55	124.84	53
		2002	21,221	9,718	27,479	18	94.90	43.46	122.89	49
		2003	20,829	8,173	28,893	18	89.10	34.96	123.60	54
		2004	20,865	7,649	31,905	17	85.22	31.24	130.32	57
		2005	20,834	7,264	34,902	17	81.81	28.52	137.04	58
		2006	20,703	6,801	38,036	17	78.50	25.79	144.23	60
		2007	20,380	3,679	40,922	18	75.41	13.61	151.41	63
	Non-Hispanic White	1992	13,551	7,726	21,289	13	129.53	73.85	203.50	24
		1993	14,609	6,735	21,173	11	142.33	65.62	206.28	19
		1994	11,345	5,836	17,078	16	112.57	57.91	169.45	28
		1995	12,311	5,005	18,254	13	123.88	50.37	183.68	23
		1996	11,431	5,086	16,792	15	116.61	51.88	171.29	24
		1997	10,784	5,632	15,498	16	109.90	57.39	157.94	28
		1998	10,339	6,005	14,280	17	104.65	60.78	144.53	26
		1999	8,730	5,977	10,604	21	87.39	59.83	106.14	42
		2000	9,291	5,584	11,362	22	91.93	55.25	112.42	41
		2001	8,411	4,822	10,836	23	81.50	46.72	104.99	51
		2002	8,534	3,908	11,051	23	81.16	37.17	105.09	45
		2003	8,385	3,290	11,632	24	77.96	30.59	108.14	47
		2004	8,452	3,098	12,924	24	76.87	28.18	117.55	48
		2005	8,540	2,978	14,306	23	76.88	26.81	128.79	49
		2006	8,638	2,837	15,869	24	77.58	25.48	142.53	47
		2007	8,706	1,572	17,482	24	78.48	14.17	157.58	46

Metropolitan Statistical Area	PWID Population	Year	Number	Min	Max	Rank	Rate	Min	Max	Rank
Riverside--San Bernardino, CA	Non-Hispanic Black	1992	2,464	1,405	3,872	47	198.11	112.95	311.24	77
		1993	2,693	1,241	3,902	38	210.33	96.97	304.83	61
		1994	2,108	1,084	3,173	49	160.22	82.42	241.18	82
		1995	2,295	933	3,403	41	168.10	68.34	249.24	73
		1996	2,130	948	3,129	42	151.61	67.46	222.71	77
		1997	2,004	1,047	2,881	44	137.47	71.79	197.55	77
		1998	1,914	1,111	2,643	46	126.04	73.20	174.08	78
		1999	1,608	1,101	1,954	54	101.61	69.56	123.41	86
		2000	1,704	1,024	2,084	48	103.87	62.42	127.01	81
		2001	1,538	881	1,981	54	89.97	51.57	115.90	87
		2002	1,558	713	2,017	52	87.80	40.21	113.69	85
		2003	1,533	601	2,126	53	82.97	32.56	115.09	86
		2004	1,554	570	2,376	49	80.40	29.47	122.95	86
		2005	1,588	554	2,660	47	78.88	27.50	132.14	85
		2006	1,639	538	3,011	46	78.53	25.80	144.28	80
		2007	1,705	308	3,423	48	79.69	14.38	160.01	82
	Hispanic	1992	10,905	6,218	17,133	5	217.66	124.10	341.96	27
		1993	11,855	5,466	17,182	4	224.53	103.52	325.42	27
		1994	9,297	4,782	13,995	6	167.50	86.16	252.14	33
		1995	10,198	4,146	15,121	4	175.01	71.15	259.49	32
		1996	9,579	4,262	14,071	4	157.06	69.88	230.70	33
		1997	9,145	4,776	13,143	4	142.17	74.24	204.31	35
		1998	8,872	5,153	12,254	4	130.22	75.63	179.85	35
		1999	7,577	5,187	9,203	5	104.88	71.80	127.39	40
		2000	8,147	4,896	9,963	5	106.45	63.97	130.17	39
		2001	7,441	4,265	9,586	6	91.62	52.52	118.02	45
		2002	7,603	3,482	9,845	5	87.99	40.30	113.94	47
		2003	7,506	2,945	10,412	5	81.22	31.87	112.67	47
		2004	7,583	2,780	11,596	5	76.60	28.08	117.13	49
		2005	7,662	2,672	12,835	5	72.49	25.28	121.44	53
		2006	7,732	2,540	14,205	5	68.72	22.57	126.26	56
		2007	7,761	1,401	15,583	7	65.69	11.86	131.90	57

Metropolitan Statistical Area	PWID Population	Year	Number	Min	Max	Rank	Rate	Min	Max	Rank
Riverside--San Bernardino, CA	Male	1992	17,719	10,103	27,838	8	198.03	112.91	311.11	34
		1993	19,282	8,889	27,945	9	213.71	98.52	309.72	28
		1994	15,204	7,821	22,887	13	167.31	86.06	251.85	38
		1995	16,832	6,843	24,956	12	182.88	74.35	271.16	34
		1996	15,997	7,117	23,498	13	171.96	76.51	252.59	35
		1997	15,474	8,081	22,238	13	162.67	84.95	233.77	37
		1998	15,216	8,837	21,015	13	155.50	90.31	214.77	38
		1999	13,161	9,010	15,986	18	130.35	89.23	158.32	45
		2000	14,308	8,598	17,496	15	137.17	82.43	167.73	43
		2001	13,174	7,552	16,972	17	121.21	69.48	156.14	55
		2002	13,518	6,191	17,505	18	119.77	54.85	155.09	47
		2003	13,339	5,234	18,503	17	113.11	44.38	156.90	56
		2004	13,390	4,909	20,475	17	108.22	39.67	165.48	57
		2005	13,347	4,654	22,360	17	103.51	36.09	173.41	58
		2006	13,179	4,329	24,213	17	98.60	32.39	181.15	60
		2007	12,819	2,314	25,740	18	93.50	16.88	187.74	64
	Female	1992	11,749	6,699	18,459	6	135.45	77.23	212.80	18
		1993	13,065	6,023	18,934	6	149.27	68.82	216.34	12
		1994	10,389	5,344	15,639	9	117.44	60.41	176.78	20
		1995	11,472	4,664	17,009	7	128.07	52.07	189.89	15
		1996	10,778	4,795	15,832	7	118.80	52.86	174.51	15
		1997	10,236	5,345	14,711	8	110.03	57.46	158.13	16
		1998	9,836	5,713	13,585	9	102.57	59.57	141.67	20
		1999	8,291	5,676	10,070	14	83.56	57.20	101.49	41
		2000	8,777	5,274	10,732	17	85.60	51.44	104.68	36
		2001	7,880	4,517	10,151	19	74.09	42.47	95.45	52
		2002	7,909	3,622	10,242	17	71.42	32.71	92.49	48
		2003	7,675	3,011	10,646	17	66.25	26.00	91.90	53
		2004	7,632	2,798	11,671	16	63.03	23.10	96.38	58
		2005	7,608	2,653	12,745	17	60.51	21.10	101.36	58
		2006	7,600	2,497	13,963	16	58.43	19.19	107.35	59
		2007	7,583	1,369	15,227	16	56.95	10.28	114.35	59

Metropolitan Statistical Area	PWID Population	Year	Number	Min	Max	Rank	Rate	Min	Max	Rank
Riverside--San Bernardino, CA	Young (15-29)	1992	8,677	4,947	13,632	6	138.25	78.83	217.21	22
		1993	8,872	4,090	12,859	6	142.88	65.87	207.07	19
		1994	6,548	3,368	9,856	13	106.33	54.70	160.06	35
		1995	6,772	2,753	10,041	11	109.75	44.62	162.72	32
		1996	6,017	2,677	8,839	14	96.92	43.12	142.37	43
		1997	5,460	2,851	7,846	17	86.17	45.00	123.84	49
		1998	5,065	2,942	6,996	19	77.65	45.10	107.24	57
		1999	4,169	2,854	5,064	24	61.88	42.36	75.16	67
		2000	4,361	2,621	5,332	24	62.59	37.61	76.53	69
		2001	3,916	2,244	5,044	31	53.14	30.46	68.46	84
		2002	3,981	1,823	5,154	31	51.22	23.46	66.33	83
		2003	3,962	1,555	5,496	32	47.89	18.79	66.43	92
		2004	4,093	1,500	6,258	31	46.30	16.97	70.80	98
		2005	4,290	1,496	7,187	32	45.85	15.99	76.81	99
		2006	4,559	1,498	8,375	27	46.40	15.24	85.24	99
		2007	4,888	882	9,815	26	48.30	8.72	96.98	98
	Old (30-64)	1992	21,238	12,109	33,366	9	187.18	106.72	294.08	27
		1993	23,749	10,949	34,419	9	205.35	94.67	297.61	20
		1994	19,161	9,857	28,844	13	162.71	83.70	244.94	33
		1995	21,606	8,784	32,035	13	180.19	73.26	267.17	22
		1996	20,815	9,261	30,575	14	171.08	76.12	251.31	22
		1997	20,316	10,609	29,195	14	162.79	85.01	233.94	22
		1998	20,068	11,655	27,716	14	156.16	90.70	215.68	24
		1999	17,368	11,890	21,095	14	130.77	89.52	158.83	35
		2000	18,827	11,314	23,022	14	137.26	82.48	167.84	27
		2001	17,234	9,879	22,201	15	121.91	69.88	157.05	38
		2002	17,533	8,029	22,703	16	120.17	55.03	155.61	34
		2003	17,107	6,712	23,730	16	113.26	44.44	157.11	34
		2004	16,929	6,206	25,887	16	108.23	39.67	165.49	33
		2005	16,566	5,776	27,752	16	102.82	35.85	172.25	35
		2006	15,953	5,241	29,310	16	96.41	31.67	177.13	38
		2007	14,976	2,703	30,070	16	88.58	15.99	177.87	40

Metropolitan Statistical Area	PWID Population	Year	Number	Min	Max	Rank	Rate	Min	Max	Rank
Rochester, NY	Total	1992	5,536	3,147	8,426	72	77.79	44.21	118.39	73
		1993	4,872	3,030	8,290	77	68.20	42.42	116.05	79
		1994	5,417	3,129	8,132	75	75.90	43.85	113.95	76
		1995	4,870	3,113	7,969	78	68.36	43.69	111.85	76
		1996	4,903	3,113	7,879	78	68.68	43.61	110.38	74
		1997	4,945	3,116	7,798	78	69.06	43.52	108.91	72
		1998	5,014	3,145	7,795	79	69.77	43.76	108.46	69
		1999	5,277	3,168	7,777	78	73.29	44.00	108.02	69
		2000	5,251	3,294	8,068	75	72.39	45.40	111.22	67
		2001	5,548	3,412	8,343	77	76.09	46.80	114.43	65
		2002	5,749	3,549	9,115	71	78.43	48.42	124.36	63
		2003	6,057	3,682	9,881	68	82.32	50.04	134.29	59
		2004	6,375	3,822	10,700	66	86.27	51.71	144.78	56
		2005	6,684	3,955	11,504	60	90.22	53.39	155.28	54
		2006	7,015	4,104	12,366	56	94.33	55.18	166.29	49
		2007	7,343	4,251	13,228	56	98.42	56.98	177.30	41
	Non-Hispanic White	1992	2,448	1,391	3,725	77	39.98	22.73	60.86	86
		1993	2,106	1,310	3,583	77	34.42	21.41	58.57	88
		1994	2,310	1,334	3,467	78	37.96	21.93	56.98	87
		1995	2,064	1,319	3,377	80	34.12	21.80	55.82	87
		1996	2,079	1,320	3,341	81	34.44	21.87	55.34	87
		1997	2,108	1,329	3,325	80	34.96	22.03	55.13	86
		1998	2,158	1,354	3,355	79	35.77	22.44	55.62	83
		1999	2,297	1,379	3,385	82	38.14	22.90	56.21	87
		2000	2,315	1,452	3,556	79	38.31	24.03	58.86	83
		2001	2,475	1,522	3,722	81	40.84	25.12	61.41	84
		2002	2,593	1,601	4,111	78	42.66	26.33	67.64	80
		2003	2,755	1,674	4,494	75	45.27	27.51	73.84	79
		2004	2,913	1,746	4,889	75	47.76	28.63	80.16	79
		2005	3,054	1,807	5,256	74	50.03	29.61	86.11	77
		2006	3,188	1,865	5,619	75	52.16	30.51	91.95	77
		2007	3,297	1,909	5,939	74	53.90	31.20	97.10	76

Metropolitan Statistical Area	PWID Population	Year	Number	Min	Max	Rank	Rate	Min	Max	Rank
Rochester, NY	Non-Hispanic Black	1992	2,195	1,248	3,341	52	345.07	196.14	525.20	42
		1993	1,880	1,169	3,198	53	289.32	179.95	492.30	48
		1994	1,995	1,153	2,996	54	301.88	174.40	453.19	42
		1995	1,684	1,076	2,755	55	251.42	160.69	411.35	49
		1996	1,568	996	2,520	58	230.34	146.24	370.17	49
		1997	1,445	911	2,280	60	208.77	131.58	329.25	51
		1998	1,328	833	2,064	60	188.54	118.26	293.10	52
		1999	1,260	757	1,857	62	176.87	106.19	260.67	61
		2000	1,131	709	1,738	63	155.74	97.68	239.28	61
		2001	1,084	667	1,630	64	147.83	90.92	222.30	67
		2002	1,031	636	1,635	63	138.77	85.67	220.04	62
		2003	1,015	617	1,656	63	135.02	82.07	220.25	60
		2004	1,023	613	1,717	64	134.48	80.62	225.70	59
		2005	1,059	627	1,823	65	138.00	81.67	237.52	56
		2006	1,139	666	2,007	64	146.42	85.66	258.12	53
		2007	1,273	737	2,293	59	161.68	93.60	291.27	47
	Hispanic	1992	801	455	1,219	44	360.09	204.68	548.07	13
		1993	798	496	1,357	45	342.63	213.10	583.00	11
		1994	982	567	1,475	40	405.44	234.22	608.65	9
		1995	959	613	1,570	41	382.56	244.50	625.91	8
		1996	1,031	655	1,657	41	395.85	251.33	636.16	7
		1997	1,094	689	1,725	41	404.89	255.18	638.54	6
		1998	1,152	723	1,791	41	412.12	258.51	640.68	6
		1999	1,245	747	1,834	40	432.96	259.94	638.09	6
		2000	1,260	790	1,935	40	418.92	262.74	643.64	7
		2001	1,343	826	2,020	41	438.37	269.62	659.22	6
		2002	1,396	862	2,214	39	444.77	274.58	705.24	6
		2003	1,469	893	2,396	39	455.52	276.88	743.10	6
		2004	1,540	923	2,584	39	467.85	280.46	785.21	6
		2005	1,606	951	2,765	39	480.32	284.25	826.71	6
		2006	1,679	982	2,960	39	490.54	286.97	864.75	4
		2007	1,756	1,017	3,164	39	500.05	289.48	900.83	2

Metropolitan Statistical Area	PWID Population	Year	Number	Min	Max	Rank	Rate	Min	Max	Rank
Rochester, NY	Male	1992	3,746	2,129	5,702	72	106.89	60.76	162.69	72
		1993	3,257	2,026	5,542	79	92.52	57.54	157.43	76
		1994	3,593	2,075	5,393	75	102.09	58.98	153.26	76
		1995	3,217	2,056	5,263	78	91.53	58.50	149.75	76
		1996	3,236	2,054	5,200	78	91.88	58.33	147.66	74
		1997	3,270	2,061	5,157	76	92.54	58.32	145.94	69
		1998	3,331	2,089	5,179	75	93.90	58.90	145.97	66
		1999	3,527	2,118	5,198	75	99.27	59.60	146.30	64
		2000	3,535	2,217	5,432	72	98.73	61.92	151.69	64
		2001	3,764	2,315	5,660	72	104.56	64.31	157.24	60
		2002	3,930	2,426	6,232	66	108.62	67.06	172.24	58
		2003	4,171	2,535	6,804	60	114.74	69.74	187.17	53
		2004	4,417	2,648	7,413	57	120.89	72.47	202.90	46
		2005	4,653	2,754	8,008	55	126.90	75.10	218.41	42
		2006	4,897	2,865	8,633	52	133.02	77.82	234.50	38
		2007	5,129	2,969	9,239	51	138.68	80.28	249.82	34
	Female	1992	1,737	987	2,643	73	48.08	27.33	73.18	77
		1993	1,606	999	2,733	77	44.34	27.58	75.44	81
		1994	1,849	1,068	2,776	72	51.12	29.53	76.75	78
		1995	1,700	1,086	2,781	78	47.09	30.09	77.04	77
		1996	1,731	1,099	2,781	78	47.85	30.38	76.90	78
		1997	1,749	1,102	2,758	78	48.23	30.40	76.06	75
		1998	1,764	1,106	2,742	78	48.46	30.39	75.33	73
		1999	1,834	1,101	2,703	80	50.29	30.19	74.12	74
		2000	1,795	1,126	2,758	78	48.86	30.64	75.07	73
		2001	1,858	1,143	2,794	79	50.34	30.96	75.70	76
		2002	1,883	1,162	2,985	76	50.73	31.32	80.43	71
		2003	1,939	1,178	3,163	76	52.07	31.65	84.94	69
		2004	1,996	1,196	3,350	76	53.42	32.02	89.65	67
		2005	2,052	1,214	3,532	75	54.84	32.45	94.39	64
		2006	2,121	1,241	3,739	71	56.49	33.04	99.58	61
		2007	2,200	1,274	3,963	73	58.47	33.85	105.33	56

Metropolitan Statistical Area	PWID Population	Year	Number	Min	Max	Rank	Rate	Min	Max	Rank
Rochester, NY	Young (15-29)	1992	981	558	1,494	81	41.05	23.33	62.48	89
		1993	965	600	1,643	80	41.14	25.59	70.00	83
		1994	1,186	685	1,781	79	51.66	29.85	77.56	75
		1995	1,166	745	1,908	77	51.86	33.14	84.84	69
		1996	1,272	808	2,045	73	57.43	36.46	92.29	65
		1997	1,379	869	2,175	72	62.93	39.66	99.24	61
		1998	1,492	936	2,320	71	68.67	43.07	106.75	60
		1999	1,665	999	2,453	69	77.38	46.46	114.04	59
		2000	1,746	1,095	2,683	65	81.29	50.99	124.90	56
		2001	1,935	1,190	2,910	67	89.49	55.04	134.58	53
		2002	2,094	1,293	3,320	62	95.74	59.10	151.80	52
		2003	2,296	1,396	3,746	58	103.49	62.90	168.82	49
		2004	2,507	1,503	4,207	55	110.92	66.49	186.15	42
		2005	2,720	1,609	4,681	53	118.14	69.91	203.34	33
		2006	2,947	1,724	5,195	47	125.76	73.57	221.70	29
		2007	3,180	1,841	5,728	43	133.44	77.25	240.40	27
	Old (30-64)	1992	4,629	2,631	7,046	66	97.96	55.68	149.10	67
		1993	3,965	2,466	6,747	73	82.66	51.41	140.65	73
		1994	4,295	2,481	6,447	69	88.72	51.25	133.18	74
		1995	3,766	2,407	6,161	77	77.25	49.37	126.38	75
		1996	3,702	2,350	5,949	77	75.20	47.75	120.86	74
		1997	3,650	2,300	5,756	78	73.45	46.30	115.84	74
		1998	3,620	2,271	5,627	78	72.19	45.28	112.23	72
		1999	3,725	2,237	5,490	75	73.79	44.30	108.75	76
		2000	3,622	2,272	5,565	77	70.93	44.49	108.99	67
		2001	3,732	2,296	5,613	76	72.78	44.76	109.44	71
		2002	3,762	2,323	5,966	73	73.16	45.17	116.01	62
		2003	3,842	2,335	6,267	68	74.74	45.43	121.93	59
		2004	3,897	2,336	6,540	67	75.96	45.54	127.49	57
		2005	3,908	2,313	6,727	65	76.54	45.30	131.74	54
		2006	3,885	2,273	6,849	66	76.29	44.63	134.49	53
		2007	3,803	2,202	6,852	65	74.90	43.36	134.93	54

Metropolitan Statistical Area	PWID Population	Year	Number	Min	Max	Rank	Rate	Min	Max	Rank
Sacramento, CA	Total	1992	18,817	14,515	25,762	24	198.97	153.48	272.41	16
		1993	19,558	14,919	25,414	24	206.07	157.19	267.77	13
		1994	16,364	15,117	18,671	29	172.07	158.96	196.34	19
		1995	17,515	14,668	23,196	25	182.25	152.64	241.38	14
		1996	16,947	14,210	22,178	24	173.91	145.82	227.60	14
		1997	16,845	13,840	21,270	24	169.19	139.01	213.63	14
		1998	17,127	13,466	20,379	22	167.81	131.94	199.67	11
		1999	16,607	13,085	18,948	24	158.49	124.88	180.83	17
		2000	17,154	12,385	19,934	22	159.29	115.01	185.10	13
		2001	16,467	11,755	21,182	22	147.30	105.15	189.47	20
		2002	16,269	10,989	22,477	22	140.38	94.82	193.95	20
		2003	16,163	10,079	23,681	22	135.50	84.49	198.52	25
		2004	16,127	9,049	24,830	22	131.98	74.06	203.21	25
		2005	15,754	7,911	25,862	23	126.71	63.63	208.01	25
		2006	14,771	6,727	26,820	26	117.25	53.40	212.90	29
		2007	13,688	5,508	27,786	30	107.29	43.17	217.80	35
	Non-Hispanic White	1992	11,406	8,798	15,615	18	167.45	129.17	229.26	15
		1993	11,557	8,816	15,017	16	171.10	130.52	222.34	9
		1994	9,491	8,768	10,830	23	141.76	130.96	161.75	20
		1995	10,032	8,401	13,286	20	149.72	125.39	198.29	14
		1996	9,631	8,076	12,604	20	143.12	120.00	187.30	14
		1997	9,534	7,833	12,038	21	139.73	114.81	176.44	14
		1998	9,675	7,607	11,512	20	139.31	109.53	165.76	12
		1999	9,373	7,385	10,695	20	132.38	104.31	151.05	19
		2000	9,671	6,983	11,239	20	133.93	96.70	155.63	14
		2001	9,260	6,610	11,912	21	124.79	89.08	160.52	19
		2002	9,101	6,147	12,573	22	119.77	80.90	165.47	18
		2003	8,957	5,586	13,124	21	115.76	72.18	169.60	20
		2004	8,807	4,942	13,560	22	112.54	63.15	173.27	25
		2005	8,422	4,229	13,825	26	107.05	53.76	175.73	27
		2006	7,666	3,491	13,919	27	97.28	44.31	176.64	39
		2007	6,828	2,747	13,860	31	86.46	34.79	175.52	43

Metropolitan Statistical Area	PWID Population	Year	Number	Min	Max	Rank	Rate	Min	Max	Rank
Sacramento, CA	Non-Hispanic Black	1992	2,536	1,957	3,473	46	358.39	276.45	490.66	38
		1993	2,875	2,193	3,735	34	400.09	305.20	519.89	26
		1994	2,542	2,348	2,900	40	350.63	323.91	400.08	32
		1995	2,802	2,346	3,710	32	379.49	317.81	502.59	18
		1996	2,735	2,293	3,579	32	362.38	303.85	474.24	18
		1997	2,699	2,217	3,408	31	348.33	286.19	439.83	17
		1998	2,694	2,118	3,205	29	338.41	266.07	402.66	15
		1999	2,546	2,006	2,905	35	311.11	245.13	354.97	17
		2000	2,558	1,847	2,973	27	304.30	219.71	353.62	12
		2001	2,394	1,709	3,080	35	274.56	195.99	353.18	19
		2002	2,323	1,569	3,209	29	256.81	173.46	354.80	18
		2003	2,293	1,430	3,360	31	245.51	153.09	359.71	19
		2004	2,312	1,297	3,560	31	239.50	134.39	368.74	19
		2005	2,333	1,172	3,830	31	234.87	117.94	385.56	19
		2006	2,321	1,057	4,214	31	227.78	103.74	413.59	22
		2007	2,353	947	4,777	29	225.62	90.78	457.99	26
	Hispanic	1992	3,169	2,444	4,338	26	293.80	226.63	402.24	18
		1993	3,151	2,404	4,095	25	281.44	214.69	365.71	19
		1994	2,533	2,340	2,890	28	218.86	202.18	249.72	25
		1995	2,617	2,192	3,466	31	218.28	182.81	289.09	23
		1996	2,456	2,059	3,214	31	196.89	165.09	257.66	25
		1997	2,382	1,957	3,007	31	183.04	150.38	231.12	24
		1998	2,377	1,869	2,828	29	174.59	137.27	207.74	23
		1999	2,277	1,794	2,598	32	160.02	126.08	182.58	22
		2000	2,341	1,691	2,721	29	156.76	113.19	182.17	24
		2001	2,256	1,610	2,902	32	141.05	100.69	181.44	25
		2002	2,256	1,524	3,117	31	131.89	89.08	182.22	28
		2003	2,292	1,429	3,358	31	126.90	79.13	185.93	29
		2004	2,363	1,326	3,639	28	124.49	69.86	191.68	29
		2005	2,414	1,212	3,963	28	121.90	61.21	200.11	27
		2006	2,398	1,092	4,354	29	116.55	53.08	211.62	31
		2007	2,386	960	4,844	29	111.89	45.02	227.13	31

Metropolitan Statistical Area	PWID Population	Year	Number	Min	Max	Rank	Rate	Min	Max	Rank
Sacramento, CA	Male	1992	11,571	8,925	15,841	27	246.67	190.27	337.71	18
		1993	11,921	9,094	15,491	25	253.13	193.10	328.93	15
		1994	9,872	9,120	11,264	29	209.45	193.49	238.99	26
		1995	10,447	8,749	13,836	27	219.46	183.80	290.65	20
		1996	9,989	8,376	13,073	27	207.03	173.59	270.93	23
		1997	9,813	8,062	12,390	27	199.11	163.59	251.41	25
		1998	9,866	7,757	11,739	26	195.34	153.58	232.42	24
		1999	9,472	7,463	10,808	31	182.74	143.99	208.51	28
		2000	9,706	7,008	11,279	26	182.10	131.48	211.61	24
		2001	9,268	6,616	11,921	29	167.32	119.44	215.23	34
		2002	9,137	6,172	12,623	27	158.96	107.37	219.61	31
		2003	9,093	5,671	13,323	26	153.72	95.86	225.23	35
		2004	9,130	5,123	14,057	26	150.54	84.48	231.79	34
		2005	9,019	4,529	14,805	28	145.90	73.27	239.52	35
		2006	8,596	3,915	15,609	31	137.23	62.50	249.17	35
		2007	8,143	3,276	16,529	34	128.19	51.58	260.21	39
	Female	1992	7,124	5,495	9,754	21	149.47	115.29	204.63	11
		1993	7,560	5,767	9,824	20	158.11	120.61	205.45	8
		1994	6,467	5,974	7,379	24	134.83	124.55	153.84	15
		1995	7,080	5,930	9,377	20	145.99	122.27	193.35	12
		1996	7,004	5,873	9,166	20	142.37	119.38	186.32	12
		1997	7,108	5,840	8,975	20	141.36	116.15	178.50	9
		1998	7,363	5,789	8,761	19	142.82	112.29	169.93	9
		1999	7,252	5,714	8,275	18	136.96	107.91	156.27	9
		2000	7,582	5,474	8,810	18	139.40	100.65	161.99	9
		2001	7,334	5,235	9,434	20	130.03	92.82	167.26	11
		2002	7,264	4,907	10,036	19	124.37	84.00	171.82	11
		2003	7,193	4,485	10,538	19	119.61	74.59	175.25	11
		2004	7,104	3,986	10,937	20	115.43	64.77	177.72	15
		2005	6,815	3,422	11,188	21	109.02	54.75	178.96	18
		2006	6,220	2,833	11,294	23	98.21	44.73	178.32	21
		2007	5,552	2,234	11,270	24	86.68	34.88	175.96	26

Metropolitan Statistical Area	PWID Population	Year	Number	Min	Max	Rank	Rate	Min	Max	Rank
Sacramento, CA	Young (15-29)	1992	4,234	3,266	5,797	27	137.86	106.34	188.74	23
		1993	4,434	3,383	5,762	25	147.42	112.45	191.56	16
		1994	3,693	3,411	4,213	29	125.26	115.71	142.92	25
		1995	3,895	3,262	5,158	25	132.37	110.86	175.31	21
		1996	3,686	3,091	4,824	28	124.50	104.39	162.93	26
		1997	3,565	2,929	4,502	29	118.24	97.14	149.29	32
		1998	3,518	2,766	4,186	32	113.88	89.54	135.51	34
		1999	3,310	2,608	3,777	32	104.41	82.27	119.14	37
		2000	3,327	2,402	3,866	36	102.01	73.65	118.54	45
		2001	3,124	2,230	4,018	40	91.11	65.04	117.20	51
		2002	3,044	2,056	4,206	41	84.29	56.93	116.45	60
		2003	3,017	1,881	4,420	43	80.13	49.97	117.41	63
		2004	3,044	1,708	4,687	43	77.86	43.69	119.88	66
		2005	3,058	1,535	5,020	44	76.02	38.17	124.80	72
		2006	3,004	1,368	5,455	45	73.08	33.28	132.70	72
		2007	2,978	1,198	6,045	51	71.17	28.64	144.47	75
	Old (30-64)	1992	14,858	11,461	20,342	23	232.67	179.48	318.55	16
		1993	15,350	11,709	19,946	18	236.77	180.62	307.66	7
		1994	12,840	11,861	14,651	24	195.68	180.76	223.27	18
		1995	13,804	11,561	18,282	22	207.03	173.39	274.19	12
		1996	13,460	11,286	17,615	23	198.41	166.36	259.65	14
		1997	13,506	11,096	17,054	22	194.58	159.86	245.69	12
		1998	13,867	10,903	16,500	20	194.83	153.18	231.82	9
		1999	13,566	10,689	15,479	18	185.62	146.26	211.80	11
		2000	14,113	10,190	16,400	20	187.98	135.73	218.44	9
		2001	13,611	9,716	17,508	20	175.60	125.35	225.88	11
		2002	13,465	9,095	18,602	19	168.78	114.00	233.18	12
		2003	13,340	8,319	19,545	19	163.39	101.89	239.40	11
		2004	13,205	7,410	20,332	19	158.93	89.18	244.69	11
		2005	12,711	6,383	20,866	20	151.12	75.89	248.09	12
		2006	11,631	5,297	21,119	21	137.05	62.42	248.84	13
		2007	10,372	4,174	21,055	25	120.98	48.68	245.58	20

Metropolitan Statistical Area	PWID Population	Year	Number	Min	Max	Rank	Rate	Min	Max	Rank
St. Louis, MO--IL	Total	1992	15,653	6,526	22,263	32	95.37	39.77	135.64	57
		1993	12,304	5,008	20,778	34	74.56	30.35	125.91	72
		1994	13,823	5,733	19,276	34	83.32	34.56	116.18	68
		1995	10,961	5,078	17,765	38	65.68	30.43	106.45	80
		1996	10,317	5,006	16,321	41	61.50	29.84	97.29	80
		1997	9,684	4,661	14,882	41	57.35	27.61	88.14	83
		1998	9,276	4,515	14,167	42	54.74	26.65	83.60	83
		1999	10,205	4,381	13,486	42	59.83	25.69	79.07	81
		2000	8,860	4,702	13,578	45	51.53	27.34	78.97	89
		2001	10,120	5,043	13,714	43	58.20	29.00	78.87	84
		2002	9,159	4,861	15,236	44	52.14	27.68	86.74	88
		2003	9,486	4,767	16,753	43	53.58	26.93	94.62	86
		2004	9,918	4,648	18,676	43	55.45	25.98	104.40	85
		2005	10,363	4,523	20,632	41	57.36	25.03	114.19	81
		2006	10,845	4,398	22,682	39	59.40	24.09	124.23	78
		2007	11,307	4,269	24,703	36	61.46	23.21	134.27	75
	Non-Hispanic White	1992	8,844	3,687	12,578	26	66.65	27.79	94.79	58
		1993	6,492	2,643	10,963	31	48.84	19.88	82.47	68
		1994	6,934	2,876	9,668	31	52.07	21.60	72.61	71
		1995	5,320	2,465	8,622	37	39.84	18.46	64.57	82
		1996	4,925	2,390	7,791	41	36.82	17.86	58.24	82
		1997	4,610	2,219	7,086	44	34.36	16.54	52.81	87
		1998	4,453	2,168	6,801	44	33.17	16.15	50.66	87
		1999	4,978	2,137	6,578	41	36.97	15.87	48.86	89
		2000	4,412	2,341	6,761	44	32.62	17.31	49.98	92
		2001	5,152	2,567	6,981	39	37.76	18.81	51.16	89
		2002	4,761	2,527	7,920	42	34.63	18.38	57.61	92
		2003	5,017	2,521	8,860	40	36.30	18.24	64.11	93
		2004	5,305	2,486	9,990	40	38.12	17.86	71.78	91
		2005	5,561	2,427	11,071	39	39.69	17.32	79.02	88
		2006	5,779	2,343	12,086	38	40.95	16.60	85.64	85
		2007	5,908	2,231	12,908	37	41.67	15.74	91.05	87

Metropolitan Statistical Area	PWID Population	Year	Number	Min	Max	Rank	Rate	Min	Max	Rank
St. Louis, MO--IL	Non-Hispanic Black	1992	6,260	2,610	8,903	16	229.81	95.82	326.86	67
		1993	5,303	2,159	8,955	18	191.68	78.02	323.70	72
		1994	6,246	2,591	8,709	16	222.15	92.14	309.77	64
		1995	5,080	2,354	8,234	17	178.23	82.58	288.88	67
		1996	4,821	2,339	7,627	18	167.00	81.04	264.19	69
		1997	4,499	2,166	6,914	19	153.38	73.83	235.72	74
		1998	4,237	2,062	6,471	20	143.04	69.62	218.45	73
		1999	4,543	1,950	6,003	21	151.27	64.94	199.90	71
		2000	3,821	2,028	5,856	20	125.56	66.63	192.42	74
		2001	4,217	2,101	5,714	20	136.65	68.09	185.17	68
		2002	3,689	1,958	6,136	19	117.88	62.57	196.09	68
		2003	3,708	1,863	6,548	18	116.95	58.78	206.54	67
		2004	3,793	1,777	7,142	19	117.63	55.12	221.49	67
		2005	3,925	1,713	7,814	20	119.65	52.22	238.20	64
		2006	4,132	1,676	8,643	18	123.77	50.19	258.88	60
		2007	4,416	1,668	9,649	18	130.41	49.24	284.93	58
	Hispanic	1992	80	34	114	78	44.06	18.37	62.67	88
		1993	70	28	118	82	36.51	14.86	61.66	89
		1994	84	35	117	80	42.31	17.55	58.99	87
		1995	70	33	114	82	34.05	15.77	55.18	89
		1996	69	34	109	83	31.85	15.45	50.39	88
		1997	67	32	103	83	29.29	14.10	45.02	88
		1998	66	32	100	84	27.52	13.39	42.02	87
		1999	74	32	98	84	29.54	12.68	39.04	86
		2000	66	35	101	86	25.04	13.29	38.38	88
		2001	78	39	106	85	27.92	13.91	37.83	85
		2002	75	40	125	86	25.13	13.34	41.80	87
		2003	83	42	147	85	26.77	13.45	47.27	85
		2004	96	45	181	82	29.17	13.67	54.92	80
		2005	114	50	227	82	32.98	14.40	65.67	77
		2006	141	57	294	79	38.65	15.68	80.85	72
		2007	180	68	394	75	47.50	17.94	103.77	66

Metropolitan Statistical Area	PWID Population	Year	Number	Min	Max	Rank	Rate	Min	Max	Rank
St. Louis, MO--IL	Male	1992	10,012	4,175	14,240	34	125.36	52.27	178.30	57
		1993	7,965	3,242	13,451	36	99.15	40.36	167.44	72
		1994	8,992	3,730	12,539	35	111.32	46.17	155.22	65
		1995	7,122	3,300	11,543	38	87.63	40.60	142.02	77
		1996	6,662	3,233	10,539	41	81.50	39.54	128.92	80
		1997	6,188	2,979	9,510	41	75.23	36.21	115.61	84
		1998	5,845	2,845	8,928	43	70.84	34.48	108.19	85
		1999	6,325	2,715	8,358	42	76.13	32.68	100.61	82
		2000	5,391	2,861	8,262	46	64.34	34.15	98.61	89
		2001	6,041	3,010	8,186	43	71.26	35.51	96.57	84
		2002	5,365	2,848	8,925	46	62.62	33.24	104.18	92
		2003	5,462	2,745	9,645	46	63.21	31.77	111.63	93
		2004	5,629	2,638	10,599	46	64.38	30.17	121.23	92
		2005	5,821	2,541	11,590	45	65.90	28.76	131.20	91
		2006	6,064	2,459	12,684	42	67.91	27.54	142.04	85
		2007	6,338	2,393	13,848	41	70.41	26.59	153.83	81
	Female	1992	5,793	2,415	8,239	27	68.75	28.67	97.78	54
		1993	4,478	1,823	7,562	33	52.88	21.52	89.30	69
		1994	5,007	2,077	6,981	33	58.81	24.39	82.01	65
		1995	3,991	1,849	6,468	35	46.62	21.60	75.55	79
		1996	3,808	1,848	6,024	38	44.27	21.48	70.04	81
		1997	3,645	1,755	5,602	37	42.10	20.26	64.70	83
		1998	3,576	1,741	5,462	39	41.14	20.03	62.83	85
		1999	4,039	1,734	5,337	37	46.17	19.82	61.01	84
		2000	3,601	1,911	5,518	41	40.85	21.68	62.60	86
		2001	4,218	2,102	5,716	39	47.34	23.59	64.14	83
		2002	3,903	2,072	6,493	41	43.38	23.03	72.17	81
		2003	4,115	2,068	7,268	37	45.40	22.82	80.18	79
		2004	4,354	2,040	8,199	35	47.61	22.31	89.65	76
		2005	4,570	1,995	9,098	35	49.49	21.60	98.52	72
		2006	4,761	1,931	9,958	32	51.04	20.70	106.75	68
		2007	4,890	1,846	10,684	31	52.04	19.65	113.71	67

Metropolitan Statistical Area	PWID Population	Year	Number	Min	Max	Rank	Rate	Min	Max	Rank
St. Louis, MO--IL	Young (15-29)	1992	4,074	1,699	5,795	32	76.86	32.05	109.32	59
		1993	3,002	1,222	5,069	37	57.41	23.37	96.96	65
		1994	3,276	1,359	4,568	35	63.35	26.28	88.34	64
		1995	2,604	1,206	4,220	41	50.54	23.42	81.92	72
		1996	2,523	1,224	3,991	40	48.95	23.75	77.44	71
		1997	2,487	1,197	3,823	42	48.16	23.18	74.02	76
		1998	2,539	1,236	3,878	40	49.13	23.92	75.04	77
		1999	3,003	1,289	3,969	36	58.09	24.94	76.77	74
		2000	2,811	1,492	4,308	40	54.38	28.86	83.34	77
		2001	3,454	1,721	4,681	34	66.60	33.19	90.25	72
		2002	3,340	1,773	5,557	37	63.98	33.96	106.44	75
		2003	3,657	1,838	6,459	34	69.44	34.90	122.63	72
		2004	3,985	1,868	7,504	33	74.33	34.83	139.96	72
		2005	4,265	1,862	8,492	33	78.47	34.25	156.22	68
		2006	4,479	1,816	9,368	29	81.20	32.93	169.84	65
		2007	4,574	1,727	9,993	28	82.12	31.01	179.43	59
	Old (30-64)	1992	11,786	4,914	16,763	32	106.06	44.22	150.85	57
		1993	9,434	3,840	15,933	33	83.69	34.06	141.33	71
		1994	10,689	4,433	14,904	34	93.59	38.82	130.51	66
		1995	8,479	3,928	13,742	37	73.49	34.05	119.11	78
		1996	7,929	3,848	12,544	39	68.23	33.11	107.93	79
		1997	7,348	3,537	11,293	41	62.69	30.18	96.35	84
		1998	6,907	3,362	10,549	41	58.65	28.55	89.58	87
		1999	7,413	3,183	9,797	41	62.37	26.78	82.42	85
		2000	6,245	3,314	9,571	42	51.94	27.56	79.60	92
		2001	6,890	3,434	9,337	41	56.47	28.14	76.52	86
		2002	6,002	3,186	9,985	43	48.62	25.81	80.89	93
		2003	5,973	3,002	10,548	45	48.02	24.13	84.80	91
		2004	6,002	2,813	11,302	45	47.92	22.45	90.22	88
		2005	6,044	2,638	12,033	44	47.85	20.88	95.26	87
		2006	6,130	2,486	12,822	40	48.11	19.51	100.63	87
		2007	6,248	2,359	13,650	38	48.70	18.39	106.41	80

Metropolitan Statistical Area	PWID Population	Year	Number	Min	Max	Rank	Rate	Min	Max	Rank
Salt Lake City--Ogden, UT	Total	1992	5,234	2,478	7,911	75	73.27	34.69	110.73	78
		1993	8,371	3,030	16,051	46	112.58	40.75	215.89	43
		1994	6,403	3,587	8,744	65	82.91	46.45	113.22	70
		1995	9,337	4,135	16,357	44	117.11	51.86	205.16	40
		1996	9,708	4,408	16,296	43	118.31	53.72	198.60	39
		1997	10,067	4,632	16,179	39	119.63	55.04	192.26	40
		1998	10,362	4,847	15,905	39	121.13	56.66	185.93	40
		1999	9,039	5,121	11,832	47	104.21	59.04	136.41	48
		2000	10,924	5,664	14,795	39	124.02	64.30	167.95	35
		2001	10,382	6,486	14,501	41	115.83	72.36	161.78	42
		2002	11,834	7,359	16,049	34	129.91	80.78	176.18	28
		2003	12,290	8,025	17,764	33	132.74	86.68	191.86	26
		2004	12,695	8,338	19,668	32	134.86	88.58	208.94	22
		2005	13,044	8,141	21,760	32	136.40	85.13	227.53	21
		2006	13,520	7,937	24,252	31	137.96	80.99	247.47	21
		2007	13,949	7,573	26,717	28	139.69	75.84	267.56	19
	Non-Hispanic White	1992	3,992	1,890	6,033	58	62.67	29.67	94.71	59
		1993	6,304	2,282	12,089	33	95.58	34.60	183.29	36
		1994	4,779	2,677	6,526	48	70.23	39.35	95.91	53
		1995	6,925	3,067	12,132	28	99.20	43.93	173.78	33
		1996	7,169	3,255	12,034	26	100.61	45.68	168.88	33
		1997	7,409	3,409	11,907	25	102.37	47.10	164.53	32
		1998	7,600	3,555	11,665	25	104.27	48.77	160.05	28
		1999	6,600	3,740	8,640	31	90.04	51.01	117.86	39
		2000	7,926	4,109	10,734	25	107.22	55.59	145.20	27
		2001	7,460	4,661	10,419	24	99.76	62.33	139.34	34
		2002	8,385	5,214	11,371	24	110.87	68.94	150.35	24
		2003	8,537	5,574	12,339	23	111.52	72.82	161.19	26
		2004	8,580	5,635	13,292	23	110.72	72.72	171.53	26
		2005	8,494	5,301	14,169	25	108.43	67.68	180.88	26
		2006	8,377	4,918	15,027	25	104.96	61.62	188.28	27
		2007	8,096	4,396	15,507	25	100.28	54.45	192.07	35

Metropolitan Statistical Area	PWID Population	Year	Number	Min	Max	Rank	Rate	Min	Max	Rank
Salt Lake City--Ogden, UT	Non-Hispanic Black	1992	427	202	645	93	596.07	282.22	900.85	9
		1993	594	215	1,139	88	795.46	287.94	1525.35	4
		1994	401	225	548	94	504.67	282.73	689.15	11
		1995	525	232	920	88	630.52	279.21	1104.57	5
		1996	500	227	839	88	579.87	263.28	973.36	5
		1997	486	224	781	88	536.92	247.05	862.91	6
		1998	481	225	738	88	513.86	240.34	788.74	6
		1999	415	235	543	91	424.44	240.49	555.62	7
		2000	512	265	693	85	493.93	256.08	668.92	5
		2001	514	321	717	84	476.41	297.63	665.39	5
		2002	640	398	868	75	576.68	358.60	782.07	4
		2003	755	493	1,091	72	661.57	431.99	956.21	3
		2004	920	604	1,426	66	780.79	512.81	1209.61	2
		2005	1,161	724	1,936	64	948.65	592.08	1582.50	2
		2006	1,535	901	2,753	51	1190.96	699.17	2136.28	2
		2007	2,090	1,135	4,003	33	1551.63	842.44	2971.97	1
	Hispanic	1992	550	260	831	51	120.53	57.07	182.17	54
		1993	837	303	1,606	43	166.51	60.27	319.30	38
		1994	615	345	840	47	110.75	62.04	151.23	53
		1995	868	385	1,521	42	142.58	63.14	249.78	39
		1996	881	400	1,479	43	129.85	58.96	217.96	38
		1997	898	413	1,443	43	118.68	54.60	190.73	38
		1998	915	428	1,404	44	110.69	51.77	169.91	39
		1999	796	451	1,042	46	89.80	50.88	117.55	47
		2000	967	501	1,309	45	103.15	53.48	139.69	41
		2001	930	581	1,299	45	93.85	58.63	131.07	42
		2002	1,082	673	1,467	45	104.24	64.82	141.37	40
		2003	1,155	754	1,670	45	106.78	69.72	154.33	36
		2004	1,238	813	1,917	45	109.77	72.10	170.06	35
		2005	1,331	831	2,220	42	112.96	70.50	188.44	34
		2006	1,458	856	2,615	42	117.06	68.72	209.98	29
		2007	1,607	872	3,078	40	121.73	66.09	233.16	26

Metropolitan Statistical Area	PWID Population	Year	Number	Min	Max	Rank	Rate	Min	Max	Rank
Salt Lake City--Ogden, UT	Male	1992	3,682	1,743	5,565	74	102.73	48.64	155.26	75
		1993	5,844	2,115	11,206	45	156.39	56.61	299.89	39
		1994	4,419	2,475	6,034	63	113.63	63.66	155.17	62
		1995	6,348	2,811	11,121	42	157.82	69.89	276.47	41
		1996	6,486	2,945	10,888	42	156.43	71.02	262.58	41
		1997	6,597	3,035	10,603	39	154.84	71.24	248.85	40
		1998	6,655	3,113	10,215	37	153.39	71.74	235.44	42
		1999	5,689	3,224	7,448	45	129.10	73.15	169.00	46
		2000	6,748	3,499	9,139	36	150.54	78.05	203.88	39
		2001	6,311	3,942	8,814	42	138.23	86.36	193.06	42
		2002	7,107	4,419	9,637	35	152.99	95.14	207.48	36
		2003	7,331	4,787	10,596	35	155.06	101.25	224.12	33
		2004	7,574	4,974	11,734	36	157.40	103.38	243.85	29
		2005	7,845	4,896	13,087	35	160.29	100.04	267.39	25
		2006	8,269	4,855	14,833	32	164.60	96.63	295.24	25
		2007	8,757	4,754	16,772	28	170.80	92.74	327.16	23
	Female	1992	1,564	740	2,364	77	43.94	20.80	66.40	83
		1993	2,550	923	4,890	57	68.95	24.96	132.22	48
		1994	2,007	1,124	2,741	69	52.35	29.33	71.48	74
		1995	3,031	1,342	5,310	47	76.73	33.98	134.43	43
		1996	3,277	1,488	5,500	42	80.73	36.65	135.51	41
		1997	3,538	1,628	5,686	39	85.17	39.19	136.88	38
		1998	3,789	1,772	5,816	38	89.88	42.04	137.96	32
		1999	3,429	1,943	4,489	43	80.36	45.53	105.20	46
		2000	4,279	2,219	5,795	35	98.91	51.28	133.96	20
		2001	4,172	2,606	5,827	40	94.86	59.26	132.48	31
		2002	4,838	3,009	6,561	31	108.37	67.39	146.97	16
		2003	5,063	3,306	7,317	26	111.74	72.96	161.51	16
		2004	5,208	3,421	8,069	26	113.19	74.34	175.35	17
		2005	5,257	3,281	8,769	25	112.59	70.27	187.82	17
		2006	5,267	3,092	9,447	25	110.27	64.74	197.80	17
		2007	5,151	2,797	9,866	26	106.02	57.56	203.06	18

Metropolitan Statistical Area	PWID Population	Year	Number	Min	Max	Rank	Rate	Min	Max	Rank
Salt Lake City--Ogden, UT	Young (15-29)	1992	1,876	888	2,835	58	67.84	32.12	102.53	64
		1993	2,963	1,073	5,683	39	102.75	37.19	197.03	41
		1994	2,242	1,256	3,062	47	74.46	41.71	101.67	57
		1995	3,238	1,434	5,673	31	103.43	45.80	181.19	39
		1996	3,342	1,517	5,610	31	103.00	46.76	172.89	39
		1997	3,447	1,586	5,540	32	102.98	47.38	165.50	39
		1998	3,540	1,656	5,434	29	103.58	48.45	158.99	39
		1999	3,091	1,751	4,046	35	88.93	50.39	116.41	51
		2000	3,752	1,945	5,082	31	106.39	55.16	144.08	39
		2001	3,597	2,247	5,024	33	101.73	63.55	142.08	45
		2002	4,153	2,583	5,632	29	116.82	72.64	158.43	37
		2003	4,390	2,867	6,345	29	122.61	80.06	177.21	29
		2004	4,639	3,047	7,186	26	128.76	84.57	199.48	28
		2005	4,900	3,058	8,175	25	135.47	84.55	225.99	27
		2006	5,249	3,082	9,416	23	143.21	84.08	256.89	24
		2007	5,625	3,054	10,775	20	152.79	82.96	292.65	22
	Old (30-64)	1992	3,482	1,649	5,262	78	79.51	37.65	120.17	84
		1993	5,510	1,995	10,566	56	121.08	43.83	232.18	45
		1994	4,195	2,350	5,729	72	89.04	49.88	121.58	73
		1995	6,117	2,709	10,715	48	126.34	55.95	221.32	41
		1996	6,380	2,897	10,709	46	128.60	58.39	215.87	38
		1997	6,648	3,059	10,684	44	131.20	60.37	210.85	37
		1998	6,878	3,217	10,558	43	133.91	62.63	205.54	34
		1999	6,024	3,413	7,886	51	115.89	65.66	151.71	41
		2000	7,293	3,781	9,877	40	138.08	71.59	187.00	26
		2001	6,918	4,322	9,662	40	127.45	79.62	178.01	34
		2002	7,832	4,870	10,621	36	141.00	87.68	191.22	23
		2003	8,027	5,242	11,603	35	141.38	92.31	204.34	19
		2004	8,118	5,332	12,577	34	139.71	91.76	216.44	16
		2005	8,081	5,043	13,480	32	135.90	84.82	226.71	15
		2006	8,002	4,698	14,354	31	130.44	76.58	233.98	17
		2007	7,744	4,205	14,834	31	122.86	66.70	235.32	17

Metropolitan Statistical Area	PWID Population	Year	Number	Min	Max	Rank	Rate	Min	Max	Rank
San Antonio, TX	Total	1992	23,204	20,878	25,229	18	259.02	233.06	281.64	4
		1993	19,796	13,813	24,368	21	216.55	151.09	266.55	9
		1994	20,428	17,077	23,585	20	218.10	182.32	251.80	9
		1995	17,664	13,459	22,745	24	184.09	140.27	237.05	13
		1996	16,526	13,125	21,745	25	169.36	134.50	222.83	16
		1997	15,446	11,769	20,733	28	155.41	118.41	208.61	19
		1998	14,558	10,799	19,762	28	143.75	106.63	195.14	26
		1999	14,388	10,132	18,739	31	139.49	98.23	181.67	27
		2000	13,361	9,837	18,616	30	127.08	93.57	177.07	32
		2001	13,664	10,280	18,417	32	127.95	96.27	172.46	31
		2002	13,036	10,707	18,833	31	119.58	98.22	172.75	33
		2003	12,927	10,162	19,191	31	116.55	91.62	173.04	32
		2004	12,722	9,527	19,598	31	112.29	84.09	172.99	33
		2005	12,302	8,801	19,978	34	106.49	76.18	172.93	39
		2006	11,989	8,116	20,670	35	100.87	68.28	173.89	42
		2007	11,590	7,361	21,357	35	94.89	60.27	174.85	47
	Non-Hispanic White	1992	5,459	4,912	5,935	45	136.24	122.59	148.14	23
		1993	5,042	3,518	6,206	44	124.92	87.16	153.77	26
		1994	5,436	4,544	6,276	42	132.77	110.99	153.28	22
		1995	4,768	3,633	6,140	46	115.12	87.71	148.23	27
		1996	4,414	3,505	5,807	47	106.32	84.44	139.89	28
		1997	3,996	3,045	5,364	48	95.68	72.90	128.43	36
		1998	3,583	2,658	4,864	59	85.11	63.14	115.54	45
		1999	3,320	2,338	4,325	69	78.34	55.17	102.03	53
		2000	2,859	2,105	3,983	73	67.28	49.54	93.74	56
		2001	2,690	2,024	3,626	77	63.06	47.44	84.99	60
		2002	2,354	1,933	3,401	80	54.69	44.92	79.01	67
		2003	2,144	1,686	3,183	83	49.58	38.98	73.61	75
		2004	1,951	1,461	3,006	87	44.48	33.31	68.53	84
		2005	1,766	1,263	2,868	94	39.84	28.50	64.69	87
		2006	1,639	1,109	2,826	95	36.28	24.56	62.55	94
		2007	1,543	980	2,844	95	33.63	21.36	61.97	97

Metropolitan Statistical Area	PWID Population	Year	Number	Min	Max	Rank	Rate	Min	Max	Rank
San Antonio, TX	Non-Hispanic Black	1992	4,137	3,722	4,498	27	711.02	639.75	773.09	5
		1993	2,758	1,924	3,395	35	467.17	325.95	575.03	15
		1994	2,403	2,009	2,774	44	394.99	330.19	456.02	23
		1995	1,889	1,440	2,433	51	301.38	229.63	388.07	35
		1996	1,719	1,365	2,262	52	269.57	214.09	354.68	40
		1997	1,655	1,261	2,221	54	254.51	193.92	341.63	38
		1998	1,684	1,249	2,285	52	253.50	188.04	344.12	33
		1999	1,861	1,311	2,424	47	274.16	193.07	357.08	20
		2000	1,979	1,457	2,758	40	286.84	211.19	399.65	16
		2001	2,345	1,764	3,161	36	335.69	252.57	452.47	11
		2002	2,593	2,130	3,747	27	363.54	298.59	525.19	9
		2003	2,951	2,320	4,382	23	411.93	323.82	611.57	8
		2004	3,271	2,450	5,039	23	443.05	331.77	682.51	5
		2005	3,472	2,484	5,638	21	459.85	328.99	746.80	5
		2006	3,598	2,436	6,203	21	450.03	304.63	775.85	6
		2007	3,568	2,266	6,574	20	432.91	274.95	797.73	9
	Hispanic	1992	13,014	11,709	14,150	4	308.57	277.64	335.51	15
		1993	11,135	7,769	13,706	5	255.84	178.51	314.92	23
		1994	11,492	9,607	13,268	4	255.97	213.98	295.52	20
		1995	9,917	7,556	12,769	5	213.71	162.83	275.18	24
		1996	9,244	7,342	12,163	5	193.65	153.80	254.79	27
		1997	8,598	6,551	11,541	5	175.24	133.52	235.22	29
		1998	8,060	5,979	10,942	6	160.00	118.68	217.20	28
		1999	7,923	5,580	10,320	4	153.23	107.90	199.57	27
		2000	7,322	5,391	10,202	7	137.19	101.01	191.15	27
		2001	7,460	5,613	10,055	5	136.43	102.64	183.88	28
		2002	7,102	5,833	10,259	6	126.21	103.66	182.33	32
		2003	7,042	5,536	10,455	6	121.81	95.75	180.84	32
		2004	6,949	5,204	10,705	6	117.34	87.87	180.75	32
		2005	6,760	4,837	10,979	8	111.36	79.67	180.85	35
		2006	6,653	4,504	11,470	9	106.34	71.98	183.33	33
		2007	6,521	4,142	12,016	11	100.78	64.00	185.70	37

Metropolitan Statistical Area	PWID Population	Year	Number	Min	Max	Rank	Rate	Min	Max	Rank
San Antonio, TX	Male	1992	16,885	15,193	18,360	9	383.80	345.33	417.30	2
		1993	14,031	9,790	17,271	18	313.12	218.47	385.42	6
		1994	14,195	11,866	16,388	16	308.66	258.02	356.35	7
		1995	12,108	9,226	15,591	24	257.26	196.02	331.26	11
		1996	11,235	8,923	14,783	24	234.80	186.48	308.94	12
		1997	10,458	7,968	14,037	26	214.59	163.51	288.05	18
		1998	9,844	7,302	13,363	27	198.22	147.04	269.08	23
		1999	9,729	6,851	12,671	29	192.20	135.35	250.33	22
		2000	9,033	6,651	12,586	28	175.04	128.88	243.88	29
		2001	9,221	6,937	12,428	31	175.78	132.25	236.93	26
		2002	8,752	7,189	12,644	31	163.41	134.22	236.07	29
		2003	8,593	6,755	12,757	32	158.05	124.24	234.64	29
		2004	8,317	6,228	12,812	32	149.47	111.93	230.25	35
		2005	7,837	5,607	12,727	36	138.01	98.73	224.12	38
		2006	7,354	4,978	12,677	38	125.76	85.13	216.81	43
		2007	6,734	4,277	12,409	39	112.01	71.14	206.40	52
	Female	1992	6,558	5,900	7,130	23	143.86	129.44	156.41	13
		1993	6,012	4,195	7,400	25	128.99	90.00	158.78	16
		1994	6,534	5,462	7,543	23	137.05	114.56	158.22	14
		1995	5,854	4,460	7,538	25	119.75	91.24	154.20	16
		1996	5,603	4,450	7,373	26	112.67	89.48	148.24	16
		1997	5,306	4,043	7,123	26	104.76	79.82	140.62	20
		1998	5,033	3,733	6,832	27	97.52	72.34	132.38	21
		1999	4,985	3,511	6,493	33	94.90	66.83	123.61	27
		2000	4,633	3,411	6,455	32	86.55	63.73	120.59	35
		2001	4,747	3,572	6,398	34	87.37	65.73	117.76	34
		2002	4,555	3,741	6,580	33	82.14	67.46	118.66	34
		2003	4,572	3,594	6,787	34	80.86	63.57	120.05	34
		2004	4,595	3,441	7,078	34	79.70	59.68	122.78	35
		2005	4,588	3,283	7,451	34	78.11	55.88	126.85	37
		2006	4,678	3,167	8,065	34	77.46	52.44	133.55	36
		2007	4,800	3,048	8,845	32	77.39	49.15	142.61	34

Metropolitan Statistical Area	PWID Population	Year	Number	Min	Max	Rank	Rate	Min	Max	Rank
San Antonio, TX	Young (15-29)	1992	8,191	7,370	8,906	8	249.05	224.08	270.79	5
		1993	6,533	4,558	8,042	14	199.00	138.85	244.95	6
		1994	6,427	5,373	7,420	14	193.93	162.12	223.89	7
		1995	5,397	4,112	6,949	18	160.72	122.45	206.94	11
		1996	4,985	3,959	6,560	19	147.28	116.97	193.78	14
		1997	4,667	3,556	6,264	21	135.92	103.57	182.45	18
		1998	4,457	3,306	6,050	22	127.60	94.65	173.21	27
		1999	4,503	3,171	5,865	22	127.14	89.53	165.59	29
		2000	4,302	3,167	5,994	25	120.04	88.39	167.26	32
		2001	4,541	3,416	6,120	25	125.68	94.56	169.40	31
		2002	4,474	3,675	6,464	26	121.71	99.97	175.83	34
		2003	4,574	3,596	6,791	27	123.00	96.69	182.61	28
		2004	4,621	3,461	7,119	27	121.35	90.87	186.93	31
		2005	4,558	3,261	7,402	29	117.33	83.94	190.54	34
		2006	4,492	3,041	7,744	28	112.21	75.95	193.44	38
		2007	4,344	2,759	8,004	29	105.25	66.84	193.94	40
	Old (30-64)	1992	15,092	13,580	16,410	22	266.22	239.53	289.46	8
		1993	13,381	9,337	16,471	25	228.40	159.36	281.14	11
		1994	14,185	11,858	16,377	21	234.39	195.93	270.60	11
		1995	12,487	9,514	16,079	25	200.21	152.55	257.81	15
		1996	11,806	9,377	15,534	27	185.25	147.13	243.74	18
		1997	11,080	8,443	14,873	27	170.33	129.78	228.63	21
		1998	10,428	7,736	14,157	28	157.19	116.60	213.39	21
		1999	10,240	7,211	13,337	31	151.19	106.47	196.92	26
		2000	9,404	6,924	13,103	29	135.70	99.91	189.07	29
		2001	9,471	7,126	12,766	32	134.04	100.85	180.67	28
		2002	8,867	7,283	12,810	31	122.72	100.80	177.29	32
		2003	8,601	6,761	12,770	32	116.67	91.72	173.22	31
		2004	8,260	6,185	12,724	33	109.83	82.24	169.19	31
		2005	7,779	5,565	12,633	33	101.44	72.58	164.75	38
		2006	7,374	4,991	12,712	33	93.54	63.32	161.25	41
		2007	6,929	4,400	12,767	33	85.67	54.41	157.87	43

Metropolitan Statistical Area	PWID Population	Year	Number	Min	Max	Rank	Rate	Min	Max	Rank
San Diego, CA	Total	1992	24,835	19,297	34,330	13	142.76	110.93	197.34	35
		1993	28,016	19,909	37,635	10	161.90	115.05	217.49	24
		1994	24,920	20,108	34,010	13	143.89	116.10	196.38	33
		1995	27,130	19,714	33,939	10	156.71	113.87	196.04	23
		1996	26,940	19,504	34,271	9	154.31	111.72	196.30	22
		1997	27,039	19,497	34,981	10	151.94	109.56	196.57	24
		1998	26,805	17,800	35,848	11	147.20	97.75	196.86	23
		1999	26,469	16,050	36,823	10	141.72	85.94	197.16	25
		2000	26,246	13,637	38,073	11	138.28	71.85	200.60	23
		2001	26,698	11,169	39,454	11	138.07	57.76	204.04	25
		2002	25,714	9,325	41,335	11	130.91	47.47	210.44	27
		2003	25,360	7,359	42,913	13	128.14	37.18	216.83	27
		2004	25,170	6,326	44,411	14	126.87	31.89	223.85	26
		2005	24,996	5,291	45,940	15	125.61	26.59	230.86	26
		2006	24,946	4,517	47,620	15	124.83	22.60	238.29	25
		2007	24,991	3,751	49,503	15	124.05	18.62	245.72	26
	Non-Hispanic White	1992	13,840	10,754	19,131	12	124.61	96.82	172.25	26
		1993	14,925	10,606	20,050	9	137.44	97.67	184.63	22
		1994	12,785	10,316	17,449	12	119.56	96.47	163.18	24
		1995	13,499	9,810	16,887	9	128.23	93.18	160.42	20
		1996	13,085	9,473	16,646	9	125.03	90.52	159.06	20
		1997	12,892	9,296	16,679	11	122.33	88.21	158.26	19
		1998	12,605	8,371	16,858	11	118.22	78.51	158.11	22
		1999	12,323	7,472	17,144	13	113.94	69.09	158.50	27
		2000	12,129	6,302	17,595	12	111.75	58.06	162.12	22
		2001	12,267	5,132	18,128	15	111.81	46.77	165.23	26
		2002	11,753	4,262	18,893	15	106.39	38.58	171.01	28
		2003	11,523	3,344	19,499	16	104.44	30.31	176.73	29
		2004	11,351	2,853	20,028	16	103.65	26.05	182.89	31
		2005	11,158	2,362	20,508	17	102.67	21.73	188.70	32
		2006	10,982	1,989	20,963	18	101.43	18.37	193.63	32
		2007	10,798	1,621	21,390	19	99.94	15.00	197.96	36

Metropolitan Statistical Area	PWID Population	Year	Number	Min	Max	Rank	Rate	Min	Max	Rank
San Diego, CA	Non-Hispanic Black	1992	3,083	2,395	4,262	35	289.08	224.62	399.61	52
		1993	3,473	2,468	4,665	28	331.13	235.31	444.83	37
		1994	3,063	2,472	4,181	33	292.10	235.69	398.65	45
		1995	3,286	2,388	4,111	28	313.49	227.80	392.16	33
		1996	3,196	2,314	4,066	29	302.54	219.03	384.87	28
		1997	3,127	2,255	4,046	28	290.76	209.67	376.17	25
		1998	3,009	1,998	4,024	26	274.80	182.48	367.51	26
		1999	2,873	1,742	3,997	27	256.21	155.36	356.44	25
		2000	2,748	1,428	3,986	26	242.54	126.01	351.83	27
		2001	2,690	1,125	3,975	28	236.45	98.92	349.42	24
		2002	2,490	903	4,002	28	218.61	79.27	351.41	28
		2003	2,358	684	3,991	29	210.19	60.99	355.68	28
		2004	2,250	565	3,970	32	202.00	50.77	356.41	31
		2005	2,153	456	3,957	33	196.52	41.60	361.19	32
		2006	2,080	377	3,970	33	190.60	34.51	363.82	37
		2007	2,032	305	4,025	35	187.92	28.21	372.24	40
	Hispanic	1992	6,545	5,086	9,048	11	179.61	139.56	248.28	36
		1993	7,617	5,413	10,232	8	202.89	144.18	272.55	31
		1994	6,903	5,570	9,421	9	178.19	143.78	243.19	29
		1995	7,581	5,509	9,484	7	190.60	138.50	238.43	29
		1996	7,535	5,455	9,586	7	183.08	132.54	232.90	31
		1997	7,526	5,427	9,737	8	175.58	126.61	227.15	28
		1998	7,399	4,913	9,895	8	165.14	109.66	220.86	26
		1999	7,234	4,386	10,064	6	154.59	93.74	215.06	26
		2000	7,107	3,692	10,309	8	146.31	76.02	212.25	25
		2001	7,183	3,005	10,615	7	142.96	59.81	211.26	24
		2002	6,910	2,506	11,108	7	133.12	48.27	213.99	25
		2003	6,859	1,990	11,607	7	128.91	37.41	218.13	28
		2004	6,920	1,739	12,211	7	127.43	32.03	224.85	26
		2005	7,074	1,497	13,001	7	127.22	26.93	233.81	24
		2006	7,373	1,335	14,074	7	130.11	23.56	248.35	26
		2007	7,841	1,177	15,531	6	134.76	20.23	266.94	25

Metropolitan Statistical Area	PWID Population	Year	Number	Min	Max	Rank	Rate	Min	Max	Rank
San Diego, CA	Male	1992	15,258	11,856	21,091	15	167.94	130.49	232.15	39
		1993	17,580	12,493	23,617	11	195.53	138.95	262.66	33
		1994	15,900	12,829	21,700	11	177.20	142.98	241.84	34
		1995	17,535	12,742	21,936	9	195.95	142.39	245.13	29
		1996	17,588	12,733	22,374	11	195.22	141.34	248.35	29
		1997	17,791	12,829	23,017	11	194.03	139.91	251.03	26
		1998	17,748	11,785	23,736	11	189.62	125.92	253.60	27
		1999	17,618	10,683	24,510	11	183.60	111.33	255.41	26
		2000	17,555	9,121	25,466	11	180.30	93.68	261.55	26
		2001	17,946	7,508	26,521	11	180.79	75.63	267.17	25
		2002	17,382	6,303	27,942	11	172.21	62.45	276.83	25
		2003	17,259	5,008	29,204	12	170.50	49.48	288.51	24
		2004	17,271	4,341	30,475	12	170.53	42.86	300.90	23
		2005	17,329	3,668	31,850	12	170.24	36.04	312.89	23
		2006	17,514	3,171	33,432	12	170.94	30.95	326.31	22
		2007	17,813	2,674	35,284	12	172.63	25.91	341.95	22
	Female	1992	9,497	7,379	13,128	12	114.26	88.78	157.95	24
		1993	10,385	7,380	13,951	11	124.92	88.77	167.81	19
		1994	9,014	7,273	12,302	13	108.00	87.14	147.39	25
		1995	9,631	6,998	12,048	13	115.14	83.67	144.04	19
		1996	9,429	6,826	11,995	13	111.60	80.79	141.96	18
		1997	9,364	6,752	12,114	13	108.54	78.27	140.43	17
		1998	9,206	6,113	12,312	13	104.02	69.07	139.12	19
		1999	9,025	5,473	12,556	11	99.39	60.27	138.27	24
		2000	8,883	4,615	12,886	14	96.11	49.93	139.41	22
		2001	8,956	3,747	13,235	12	95.18	39.82	140.66	29
		2002	8,526	3,092	13,706	16	89.29	32.38	143.53	28
		2003	8,277	2,402	14,007	15	85.61	24.84	144.87	31
		2004	8,042	2,021	14,190	15	82.81	20.81	146.12	29
		2005	7,765	1,644	14,271	15	79.88	16.91	146.82	33
		2006	7,470	1,353	14,259	17	76.71	13.89	146.43	38
		2007	7,141	1,072	14,145	19	72.66	10.91	143.94	41

Metropolitan Statistical Area	PWID Population	Year	Number	Min	Max	Rank	Rate	Min	Max	Rank
San Diego, CA	Young (15-29)	1992	6,342	4,928	8,766	17	95.72	74.38	132.32	43
		1993	6,722	4,777	9,030	13	105.00	74.61	141.05	36
		1994	5,750	4,639	7,847	17	91.76	74.04	125.24	44
		1995	6,144	4,465	7,686	13	99.71	72.46	124.74	42
		1996	6,095	4,413	7,754	12	99.16	71.79	126.14	41
		1997	6,199	4,470	8,020	12	99.72	71.91	129.02	41
		1998	6,293	4,179	8,417	12	99.62	66.15	133.23	42
		1999	6,410	3,887	8,918	11	99.15	60.12	137.93	40
		2000	6,580	3,419	9,545	12	100.54	52.24	145.85	46
		2001	6,931	2,899	10,242	13	104.48	43.71	154.41	43
		2002	6,891	2,499	11,078	12	102.43	37.14	164.65	45
		2003	6,973	2,023	11,799	13	103.81	30.12	175.65	48
		2004	7,035	1,768	12,413	14	105.02	26.40	185.30	49
		2005	7,017	1,485	12,896	15	104.03	22.02	191.19	48
		2006	6,927	1,254	13,222	16	101.75	18.43	194.23	45
		2007	6,738	1,011	13,347	17	98.19	14.74	194.50	47
	Old (30-64)	1992	18,774	14,588	25,951	13	174.29	135.43	240.93	31
		1993	21,455	15,246	28,821	12	196.78	139.84	264.35	22
		1994	19,248	15,531	26,269	12	174.14	140.51	237.66	22
		1995	21,058	15,302	26,343	14	188.85	137.23	236.25	19
		1996	20,947	15,165	26,648	13	185.19	134.07	235.58	19
		1997	21,001	15,143	27,169	12	181.35	130.77	234.62	17
		1998	20,739	13,772	27,736	12	174.40	115.81	233.24	17
		1999	20,351	12,340	28,311	11	166.65	101.05	231.84	19
		2000	20,006	10,395	29,022	13	160.89	83.59	233.39	17
		2001	20,137	8,424	29,759	11	158.52	66.32	234.27	18
		2002	19,161	6,949	30,802	13	148.37	53.80	238.50	18
		2003	18,648	5,411	31,556	13	142.64	41.39	241.37	18
		2004	18,252	4,587	32,204	14	138.90	34.91	245.08	17
		2005	17,870	3,783	32,843	14	135.85	28.76	249.68	16
		2006	17,582	3,184	33,562	13	133.44	24.16	254.71	15
		2007	17,365	2,607	34,397	12	130.72	19.62	258.94	15

Metropolitan Statistical Area	PWID Population	Year	Number	Min	Max	Rank	Rate	Min	Max	Rank
San Francisco, CA	Total	1992	30,862	23,461	38,352	6	269.30	204.72	334.66	3
		1993	33,161	24,229	44,380	7	287.80	210.28	385.16	3
		1994	27,925	24,934	32,683	9	241.74	215.85	282.94	4
		1995	30,401	23,979	42,019	8	262.04	206.69	362.19	3
		1996	29,484	22,797	40,838	8	251.82	194.70	348.79	3
		1997	29,032	22,684	39,867	8	243.01	189.87	333.69	5
		1998	29,221	23,385	38,666	8	240.77	192.68	318.59	5
		1999	26,898	24,949	30,097	9	219.71	203.80	245.85	5
		2000	29,835	26,698	34,136	9	241.93	216.50	276.81	5
		2001	29,844	28,421	32,059	9	241.08	229.59	258.98	5
		2002	30,217	27,625	32,585	9	246.50	225.35	265.81	4
		2003	30,342	24,264	33,163	9	249.62	199.61	272.82	4
		2004	30,014	21,430	33,862	9	248.20	177.21	280.01	4
		2005	29,355	18,901	34,817	9	242.31	156.01	287.40	5
		2006	28,737	16,429	35,942	10	235.82	134.82	294.94	5
		2007	28,088	14,111	37,203	13	228.39	114.74	302.50	6
	Non-Hispanic White	1992	19,427	14,769	24,143	5	297.69	226.31	369.94	2
		1993	20,397	14,903	27,298	4	314.69	229.93	421.15	1
		1994	16,676	14,890	19,518	6	259.52	231.72	303.74	2
		1995	17,530	13,827	24,230	5	274.42	216.45	379.30	1
		1996	16,346	12,638	22,640	6	255.79	197.77	354.28	1
		1997	15,429	12,055	21,186	6	238.01	185.96	326.82	3
		1998	14,867	11,898	19,672	6	226.77	181.47	300.06	4
		1999	13,112	12,163	14,672	11	199.24	184.81	222.94	5
		2000	13,980	12,511	15,996	8	212.62	190.27	243.27	4
		2001	13,523	12,878	14,527	12	205.58	195.78	220.84	5
		2002	13,357	12,211	14,404	9	206.63	188.90	222.82	4
		2003	13,237	10,586	14,468	9	207.86	166.22	227.18	4
		2004	13,107	9,358	14,787	12	207.81	148.37	234.45	4
		2005	13,038	8,394	15,464	13	206.97	133.26	245.48	5
		2006	13,201	7,547	16,511	13	208.78	119.36	261.12	6
		2007	13,567	6,816	17,970	13	213.48	107.25	282.76	5

Metropolitan Statistical Area	PWID Population	Year	Number	Min	Max	Rank	Rate	Min	Max	Rank
San Francisco, CA	Non-Hispanic Black	1992	6,011	4,569	7,470	19	789.10	599.88	980.61	3
		1993	6,470	4,727	8,659	14	869.51	635.31	1163.67	2
		1994	5,575	4,978	6,525	19	770.32	687.81	901.59	3
		1995	6,315	4,981	8,728	15	890.08	702.05	1230.24	1
		1996	6,451	4,988	8,935	14	926.19	716.10	1282.83	1
		1997	6,744	5,270	9,261	14	975.70	762.35	1339.80	1
		1998	7,234	5,789	9,571	12	1059.12	847.57	1401.42	1
		1999	7,092	6,578	7,936	11	1058.89	982.19	1184.84	1
		2000	8,343	7,466	9,546	11	1275.34	1141.28	1459.21	1
		2001	8,781	8,362	9,433	11	1364.66	1299.60	1465.97	1
		2002	9,250	8,457	9,975	9	1463.58	1338.00	1578.26	1
		2003	9,527	7,618	10,412	9	1536.45	1228.67	1679.26	1
		2004	9,501	6,784	10,719	9	1555.32	1110.48	1754.71	1
		2005	9,183	5,913	10,892	9	1519.48	978.34	1802.21	1
		2006	8,684	4,965	10,861	11	1451.70	829.95	1815.64	1
		2007	7,991	4,015	10,585	12	1349.87	678.15	1787.92	2
	Hispanic	1992	3,739	2,843	4,647	21	221.38	168.30	275.11	26
		1993	4,145	3,029	5,547	19	239.57	175.04	320.62	24
		1994	3,539	3,160	4,142	23	200.17	178.73	234.28	26
		1995	3,851	3,037	5,323	18	213.57	168.45	295.19	25
		1996	3,690	2,853	5,110	18	199.52	154.26	276.34	23
		1997	3,557	2,779	4,885	19	186.02	145.35	255.44	23
		1998	3,485	2,789	4,611	20	176.98	141.63	234.18	22
		1999	3,112	2,887	3,482	25	154.92	143.70	173.35	25
		2000	3,348	2,996	3,831	21	162.88	145.76	186.36	21
		2001	3,258	3,103	3,500	21	157.95	150.42	169.68	22
		2002	3,229	2,952	3,482	21	157.14	143.65	169.45	20
		2003	3,204	2,562	3,502	22	156.10	124.83	170.61	20
		2004	3,174	2,266	3,580	23	154.19	110.09	173.95	21
		2005	3,161	2,035	3,749	24	152.06	97.91	180.36	22
		2006	3,217	1,839	4,024	23	153.15	87.56	191.55	22
		2007	3,350	1,683	4,437	23	156.60	78.67	207.42	23

Metropolitan Statistical Area	PWID Population	Year	Number	Min	Max	Rank	Rate	Min	Max	Rank
San Francisco, CA	Male	1992	20,476	15,566	25,446	6	349.21	265.47	433.96	5
		1993	22,070	16,126	29,537	6	374.07	273.31	500.62	3
		1994	18,685	16,683	21,869	8	316.20	282.33	370.08	4
		1995	20,485	16,157	28,313	8	344.88	272.02	476.68	3
		1996	20,029	15,486	27,741	8	334.11	258.33	462.76	3
		1997	19,894	15,544	27,318	8	325.18	254.08	446.53	3
		1998	20,200	16,165	26,729	8	324.76	259.89	429.72	3
		1999	18,750	17,392	20,980	9	298.44	276.83	333.94	6
		2000	20,954	18,752	23,975	8	330.67	295.91	378.35	4
		2001	21,093	20,088	22,659	9	331.36	315.56	355.96	2
		2002	21,458	19,616	23,139	7	340.26	311.06	366.92	3
		2003	21,604	17,276	23,612	7	345.43	276.23	377.54	3
		2004	21,376	15,262	24,116	7	343.30	245.12	387.32	3
		2005	20,853	13,426	24,733	8	333.70	214.86	395.79	4
		2006	20,293	11,602	25,381	9	322.77	184.53	403.69	4
		2007	19,640	9,867	26,014	9	309.30	155.39	409.67	4
	Female	1992	10,133	7,703	12,592	9	181.07	137.65	225.01	4
		1993	10,977	8,020	14,690	8	195.24	142.65	261.29	3
		1994	9,239	8,249	10,813	12	163.74	146.20	191.64	6
		1995	9,980	7,872	13,795	9	176.27	139.03	243.64	4
		1996	9,549	7,383	13,226	11	167.13	129.22	231.48	5
		1997	9,235	7,216	12,681	15	158.42	123.78	217.54	6
		1998	9,102	7,284	12,043	15	153.83	123.11	203.55	6
		1999	8,190	7,597	9,164	16	137.42	127.47	153.77	8
		2000	8,879	7,946	10,159	15	148.11	132.54	169.46	7
		2001	8,693	8,278	9,338	15	144.56	137.67	155.30	8
		2002	8,640	7,899	9,317	14	145.15	132.70	156.52	7
		2003	8,555	6,841	9,350	14	144.97	115.93	158.45	7
		2004	8,397	5,996	9,474	14	143.14	102.20	161.49	7
		2005	8,214	5,289	9,743	14	140.04	90.17	166.10	7
		2006	8,120	4,642	10,156	14	137.66	78.70	172.17	7
		2007	8,104	4,072	10,734	14	136.25	68.45	180.46	7

Metropolitan Statistical Area	PWID Population	Year	Number	Min	Max	Rank	Rate	Min	Max	Rank
San Francisco, CA	Young (15-29)	1992	6,957	5,289	8,646	15	197.95	150.49	246.00	11
		1993	8,210	5,999	10,988	8	237.02	173.18	317.20	3
		1994	7,287	6,506	8,529	8	214.17	191.23	250.66	4
		1995	8,086	6,378	11,176	7	239.26	188.72	330.70	1
		1996	7,781	6,016	10,777	7	230.34	178.10	319.04	1
		1997	7,441	5,814	10,218	7	217.20	169.71	298.25	1
		1998	7,158	5,728	9,471	9	206.57	165.31	273.34	3
		1999	6,230	5,779	6,971	13	178.80	165.85	200.07	12
		2000	6,502	5,818	7,439	13	185.58	166.08	212.34	9
		2001	6,128	5,835	6,583	17	181.52	172.87	195.00	13
		2002	5,893	5,387	6,355	16	184.80	168.94	199.28	13
		2003	5,706	4,563	6,236	20	187.73	150.12	205.17	13
		2004	5,564	3,973	6,277	21	189.77	135.49	214.10	14
		2005	5,523	3,556	6,550	20	192.29	123.81	228.07	15
		2006	5,683	3,249	7,107	19	199.22	113.90	249.16	14
		2007	6,076	3,052	8,048	19	211.67	106.34	280.36	8
	Old (30-64)	1992	24,170	18,374	30,036	6	304.20	231.25	378.03	3
		1993	25,177	18,396	33,695	8	312.44	228.28	418.14	3
		1994	20,797	18,569	24,340	9	255.20	227.87	298.69	7
		1995	22,473	17,726	31,061	8	273.33	215.59	377.79	4
		1996	21,858	16,900	30,274	11	262.39	202.87	363.42	5
		1997	21,752	16,996	29,870	11	255.27	199.45	350.53	6
		1998	22,236	17,795	29,423	11	256.42	205.21	339.30	5
		1999	20,831	19,322	23,309	9	237.85	220.62	266.14	7
		2000	23,510	21,039	26,900	9	266.30	238.31	304.69	5
		2001	23,881	22,742	25,653	9	265.25	252.60	284.94	5
		2002	24,470	22,370	26,387	6	269.80	246.65	290.94	5
		2003	24,755	19,796	27,056	6	271.56	217.16	296.80	5
		2004	24,534	17,517	27,680	6	267.81	191.22	302.15	5
		2005	23,871	15,370	28,313	6	258.27	166.29	306.33	5
		2006	23,027	13,164	28,799	7	246.71	141.04	308.56	5
		2007	21,872	10,988	28,970	7	231.99	116.55	307.28	5

Metropolitan Statistical Area	PWID Population	Year	Number	Min	Max	Rank	Rate	Min	Max	Rank
San Jose, CA	Total	1992	16,949	11,477	22,181	28	158.83	107.55	207.86	28
		1993	14,335	10,724	19,568	30	133.71	100.02	182.52	35
		1994	14,062	10,894	16,961	33	130.84	101.36	157.82	39
		1995	11,628	8,520	14,510	34	107.29	78.62	133.88	47
		1996	10,631	7,555	12,938	38	96.55	68.62	117.51	50
		1997	9,615	6,673	12,098	43	85.36	59.24	107.40	58
		1998	8,620	5,910	11,596	45	75.10	51.49	101.04	67
		1999	8,398	4,661	11,161	50	72.38	40.17	96.19	71
		2000	7,169	3,513	11,014	55	61.10	29.94	93.86	74
		2001	7,359	3,685	10,612	59	62.51	31.31	90.15	77
		2002	6,657	4,211	9,873	59	57.24	36.21	84.90	80
		2003	6,551	4,755	9,557	61	56.66	41.13	82.67	81
		2004	6,465	4,983	9,482	61	56.00	43.16	82.12	83
		2005	6,420	5,004	9,617	66	55.16	42.99	82.63	85
		2006	6,399	4,702	9,843	67	54.39	39.96	83.66	92
		2007	6,381	4,258	10,146	68	53.53	35.72	85.12	90
	Non-Hispanic White	1992	9,334	6,320	12,215	24	156.27	105.82	204.52	17
		1993	7,914	5,921	10,803	26	135.82	101.60	185.40	24
		1994	7,701	5,966	9,289	28	135.64	105.08	163.61	21
		1995	6,260	4,587	7,812	32	112.32	82.30	140.16	28
		1996	5,582	3,967	6,793	35	101.10	71.85	123.05	32
		1997	4,889	3,393	6,151	38	88.58	61.47	111.44	43
		1998	4,217	2,892	5,674	47	76.66	52.56	103.13	51
		1999	3,935	2,184	5,229	54	72.39	40.18	96.21	56
		2000	3,205	1,570	4,923	64	59.64	29.23	91.63	63
		2001	3,132	1,569	4,517	72	59.55	29.82	85.87	66
		2002	2,698	1,706	4,001	75	52.96	33.50	78.55	69
		2003	2,533	1,839	3,696	78	51.06	37.06	74.49	72
		2004	2,398	1,848	3,517	81	49.34	38.03	72.36	73
		2005	2,301	1,793	3,446	82	47.89	37.33	71.74	79
		2006	2,239	1,645	3,443	84	47.03	34.55	72.34	83
		2007	2,207	1,473	3,509	84	46.62	31.11	74.13	85

Metropolitan Statistical Area	PWID Population	Year	Number	Min	Max	Rank	Rate	Min	Max	Rank
San Jose, CA	Non-Hispanic Black	1992	1,768	1,197	2,314	63	471.33	319.17	616.84	20
		1993	1,508	1,128	2,059	61	406.87	304.37	555.40	21
		1994	1,476	1,144	1,781	62	404.88	313.66	488.37	22
		1995	1,207	884	1,506	65	333.48	244.36	416.14	26
		1996	1,083	769	1,318	65	300.09	213.27	365.23	31
		1997	955	663	1,202	67	263.76	183.04	331.85	35
		1998	832	571	1,120	71	230.30	157.91	309.84	41
		1999	786	436	1,045	72	219.68	121.94	291.98	42
		2000	651	319	1,000	78	184.34	90.33	283.20	48
		2001	649	325	935	79	185.80	93.06	267.94	49
		2002	572	362	848	78	166.93	105.59	247.57	49
		2003	551	400	804	79	162.72	118.10	237.39	49
		2004	537	414	787	81	159.49	122.93	233.89	46
		2005	531	414	796	81	156.81	122.23	234.92	47
		2006	535	393	823	81	156.50	114.98	240.70	48
		2007	549	366	873	81	158.26	105.60	251.61	48
	Hispanic	1992	5,097	3,452	6,671	14	231.51	156.77	302.98	25
		1993	4,088	3,058	5,581	20	180.97	135.38	247.03	35
		1994	3,871	2,999	4,669	20	167.57	129.82	202.13	32
		1995	3,140	2,301	3,919	25	132.91	97.39	165.85	41
		1996	2,857	2,031	3,478	28	117.34	83.39	142.81	40
		1997	2,604	1,807	3,276	29	103.42	71.77	130.11	44
		1998	2,376	1,629	3,196	31	91.59	62.80	123.22	49
		1999	2,374	1,318	3,155	31	89.79	49.84	119.34	48
		2000	2,090	1,024	3,212	33	77.30	37.88	118.76	52
		2001	2,221	1,112	3,203	33	82.08	41.11	118.36	51
		2002	2,083	1,317	3,089	34	77.60	49.08	115.08	49
		2003	2,124	1,542	3,099	34	78.24	56.79	114.15	49
		2004	2,169	1,672	3,180	33	78.68	60.64	115.39	47
		2005	2,220	1,730	3,326	33	78.72	61.36	117.93	47
		2006	2,271	1,668	3,493	31	78.52	57.69	120.78	48
		2007	2,310	1,541	3,672	31	77.95	52.01	123.93	48

Metropolitan Statistical Area	PWID Population	Year	Number	Min	Max	Rank	Rate	Min	Max	Rank
San Jose, CA	Male	1992	11,313	7,661	14,805	28	204.49	138.47	267.62	32
		1993	9,432	7,056	12,875	32	169.71	126.96	231.66	37
		1994	9,132	7,074	11,015	34	164.13	127.15	197.97	40
		1995	7,463	5,469	9,313	36	133.05	97.49	166.03	47
		1996	6,753	4,800	8,219	39	118.63	84.31	144.38	52
		1997	6,055	4,202	7,618	42	104.08	72.23	130.95	62
		1998	5,389	3,695	7,250	44	90.97	62.37	122.38	69
		1999	5,221	2,898	6,940	51	87.15	48.37	115.83	75
		2000	4,440	2,176	6,822	56	73.20	35.87	112.45	80
		2001	4,548	2,278	6,558	59	74.60	37.36	107.58	83
		2002	4,112	2,601	6,099	62	68.15	43.11	101.07	84
		2003	4,052	2,941	5,911	62	67.46	48.96	98.41	86
		2004	4,010	3,091	5,881	61	66.73	51.43	97.86	88
		2005	4,000	3,118	5,992	60	65.87	51.34	98.68	92
		2006	4,011	2,947	6,170	65	65.26	47.94	100.37	92
		2007	4,030	2,689	6,408	64	64.62	43.12	102.74	92
	Female	1992	5,739	3,886	7,511	28	111.68	75.62	146.15	25
		1993	4,982	3,727	6,801	27	96.49	72.18	131.71	31
		1994	5,008	3,879	6,040	32	96.61	74.84	116.53	34
		1995	4,235	3,103	5,284	33	81.00	59.35	101.07	40
		1996	3,951	2,808	4,809	36	74.30	52.80	90.43	48
		1997	3,638	2,525	4,577	38	66.79	46.35	84.04	55
		1998	3,311	2,270	4,455	42	59.63	40.88	80.22	58
		1999	3,265	1,812	4,340	47	58.19	32.30	77.34	63
		2000	2,813	1,378	4,321	51	49.63	24.32	76.25	71
		2001	2,903	1,454	4,186	57	51.15	25.62	73.77	74
		2002	2,631	1,664	3,902	57	47.02	29.74	69.73	77
		2003	2,583	1,875	3,768	59	46.49	33.74	67.83	76
		2004	2,532	1,951	3,713	62	45.73	35.25	67.06	80
		2005	2,485	1,937	3,723	64	44.64	34.79	66.87	80
		2006	2,436	1,790	3,747	65	43.35	31.85	66.68	83
		2007	2,375	1,585	3,777	64	41.80	27.89	66.45	83

Metropolitan Statistical Area	PWID Population	Year	Number	Min	Max	Rank	Rate	Min	Max	Rank
San Jose, CA	Young (15-29)	1992	4,273	2,894	5,592	26	116.22	78.70	152.09	31
		1993	3,856	2,884	5,263	26	106.81	79.91	145.81	33
		1994	3,912	3,031	4,719	26	110.60	85.68	133.41	32
		1995	3,264	2,392	4,073	29	92.88	68.06	115.90	47
		1996	2,951	2,097	3,592	34	83.68	59.47	101.85	52
		1997	2,598	1,803	3,269	38	72.68	50.44	91.44	59
		1998	2,242	1,537	3,016	49	62.00	42.51	83.41	64
		1999	2,087	1,158	2,774	57	57.47	31.90	76.38	77
		2000	1,699	833	2,610	67	46.43	22.75	71.34	83
		2001	1,667	835	2,404	76	46.82	23.45	67.51	88
		2002	1,454	920	2,156	80	42.95	27.17	63.70	95
		2003	1,398	1,015	2,039	85	42.76	31.04	62.39	99
		2004	1,374	1,059	2,015	87	42.98	33.13	63.04	100
		2005	1,393	1,086	2,087	89	43.71	34.07	65.49	100
		2006	1,459	1,072	2,244	83	45.35	33.32	69.75	100
		2007	1,579	1,054	2,511	82	48.37	32.27	76.90	97
	Old (30-64)	1992	12,841	8,695	16,805	26	183.59	124.32	240.26	28
		1993	10,580	7,914	14,442	29	148.76	111.28	203.06	34
		1994	10,237	7,931	12,348	36	141.99	110.00	171.26	41
		1995	8,448	6,190	10,542	39	115.36	84.53	143.96	45
		1996	7,781	5,530	9,470	40	103.97	73.89	126.54	52
		1997	7,136	4,952	8,978	42	92.81	64.41	116.77	59
		1998	6,508	4,462	8,756	44	82.79	56.76	111.38	62
		1999	6,455	3,583	8,580	47	80.98	44.95	107.63	67
		2000	5,602	2,745	8,606	51	69.37	33.99	106.57	69
		2001	5,826	2,918	8,401	52	70.96	35.54	102.32	74
		2002	5,316	3,362	7,884	53	64.47	40.78	95.62	69
		2003	5,245	3,807	7,653	52	63.26	45.91	92.29	69
		2004	5,153	3,972	7,558	52	61.72	47.58	90.52	73
		2005	5,045	3,932	7,557	51	59.68	46.52	89.41	73
		2006	4,890	3,593	7,521	53	57.21	42.03	87.99	72
		2007	4,646	3,100	7,387	54	53.69	35.82	85.35	72

Metropolitan Statistical Area	PWID Population	Year	Number	Min	Max	Rank	Rate	Min	Max	Rank
San Juan-Caguas-Arecibo, PR	Total	1992	17,064	13,926	22,393	27	142.49	116.29	186.99	36
		1993	15,625	10,783	21,760	28	129.30	89.23	180.06	38
		1994	17,398	15,171	21,115	24	142.68	124.41	173.16	34
		1995	16,028	11,876	20,482	27	130.27	96.52	166.47	35
		1996	16,038	12,321	19,867	27	129.20	99.26	160.05	35
		1997	16,016	12,674	19,269	26	127.90	101.21	153.87	35
		1998	16,010	13,031	18,687	26	126.74	103.16	147.93	35
		1999	16,851	13,783	18,648	23	132.25	108.17	146.36	31
		2000	15,957	13,084	19,303	24	124.17	101.81	150.21	34
		2001	16,414	12,237	19,966	23	126.64	94.42	154.05	32
		2002	15,346	11,487	20,581	25	117.42	87.89	157.47	34
		2003	15,127	11,044	21,203	26	114.78	83.80	160.88	34
		2004	14,909	10,694	21,805	26	112.19	80.47	164.08	34
		2005	14,630	10,149	22,414	28	109.19	75.75	167.29	35
		2006	14,284	9,352	23,011	28	105.74	69.23	170.35	36
		2007	13,910	8,493	23,614	29	102.15	62.37	173.41	39
	Hispanic	1992	17,064	13,926	22,393	3	159.90	130.50	209.84	41
		1993	15,625	10,783	21,760	3	145.74	100.57	202.96	40
		1994	17,398	15,171	21,115	3	161.89	141.16	196.47	35
		1995	16,028	11,876	20,482	3	147.89	109.58	189.00	35
		1996	16,038	12,321	19,867	3	145.66	111.90	180.43	36
		1997	16,016	12,674	19,269	3	142.19	112.51	171.06	34
		1998	16,010	13,031	18,687	3	139.49	113.54	162.82	34
		1999	16,851	13,783	18,648	3	145.23	118.79	160.72	29
		2000	15,957	13,084	19,303	3	135.90	111.43	164.40	28
		2001	16,414	12,237	19,966	3	139.85	104.27	170.12	26
		2002	15,346	11,487	20,581	3	132.37	99.08	177.52	26
		2003	15,127	11,044	21,203	3	131.11	95.72	183.77	27
		2004	14,909	10,694	21,805	3	129.21	92.68	188.97	24
		2005	14,630	10,149	22,414	3	122.64	85.08	187.90	26
		2006	14,284	9,352	23,011	3	118.76	77.75	191.31	27
		2007	13,910	8,493	23,614	3	114.70	70.04	194.72	29

Metropolitan Statistical Area	PWID Population	Year	Number	Min	Max	Rank	Rate	Min	Max	Rank
Sarasota--Bradenton, FL	Total	1992	2,614	2,262	2,962	94	93.71	81.07	106.17	59
		1993	2,554	1,618	3,089	91	90.46	57.32	109.43	54
		1994	3,145	3,021	3,246	89	109.31	104.99	112.83	47
		1995	3,013	1,703	3,663	88	102.45	57.90	124.56	49
		1996	3,253	1,738	4,132	85	108.70	58.07	138.10	45
		1997	3,530	1,781	4,654	85	115.03	58.04	151.63	42
		1998	3,832	1,831	5,185	85	121.38	58.00	164.24	39
		1999	4,877	4,435	5,737	82	150.35	136.72	176.85	22
		2000	4,382	1,957	6,127	84	131.07	58.54	183.26	30
		2001	5,487	4,678	6,519	78	159.65	136.12	189.67	15
		2002	4,811	1,951	6,886	81	135.94	55.12	194.56	25
		2003	5,024	1,940	7,243	78	138.34	53.41	199.45	20
		2004	5,340	1,938	7,611	75	142.26	51.63	202.76	18
		2005	5,768	1,933	8,026	73	148.10	49.64	206.07	18
		2006	6,103	1,891	8,252	72	153.82	47.66	207.96	16
		2007	6,385	1,817	8,425	67	159.88	45.50	210.98	11
	Non-Hispanic White	1992	2,125	1,838	2,407	80	86.22	74.59	97.69	43
		1993	2,039	1,292	2,466	81	82.23	52.11	99.48	43
		1994	2,477	2,379	2,557	76	98.53	94.63	101.70	39
		1995	2,352	1,329	2,860	75	92.08	52.04	111.96	39
		1996	2,527	1,350	3,211	74	97.85	52.27	124.30	35
		1997	2,739	1,382	3,611	73	104.02	52.48	137.12	31
		1998	2,978	1,423	4,030	71	110.56	52.83	149.59	23
		1999	3,805	3,459	4,475	58	138.33	125.78	162.70	14
		2000	3,436	1,535	4,803	60	122.02	54.50	170.61	18
		2001	4,326	3,688	5,139	52	150.51	128.32	178.81	12
		2002	3,813	1,546	5,458	55	129.67	52.58	185.59	14
		2003	4,000	1,545	5,767	53	133.42	51.52	192.36	14
		2004	4,265	1,548	6,079	47	138.41	50.24	197.28	12
		2005	4,612	1,546	6,417	46	145.17	48.66	201.99	11
		2006	4,873	1,510	6,588	44	151.78	47.03	205.20	11
		2007	5,072	1,444	6,693	44	158.34	45.06	208.95	9

Metropolitan Statistical Area	PWID Population	Year	Number	Min	Max	Rank	Rate	Min	Max	Rank
Sarasota--Bradenton, FL	Non-Hispanic Black	1992	345	299	391	96	195.55	169.16	221.55	79
		1993	350	222	424	95	194.17	123.04	234.90	68
		1994	432	415	446	92	233.53	224.30	241.06	61
		1995	403	228	490	93	211.48	119.52	257.13	54
		1996	413	221	525	92	212.36	113.44	269.79	52
		1997	420	212	553	91	208.95	105.43	275.45	49
		1998	422	201	570	89	203.59	97.29	275.48	49
		1999	495	450	582	88	232.13	211.08	273.03	38
		2000	411	184	575	89	186.55	83.33	260.83	47
		2001	482	411	572	86	211.22	180.08	250.94	32
		2002	402	163	575	86	169.90	68.90	243.17	45
		2003	410	158	591	87	167.21	64.56	241.08	45
		2004	439	160	626	87	172.68	62.67	246.13	41
		2005	499	167	694	84	188.72	63.26	262.58	37
		2006	581	180	786	77	211.07	65.40	285.38	26
		2007	706	201	932	77	249.36	70.97	329.05	19
	Hispanic	1992	68	59	77	82	57.19	49.48	64.80	80
		1993	77	49	93	78	58.43	37.02	70.68	74
		1994	106	102	109	78	73.59	70.68	75.96	69
		1995	110	62	134	76	69.13	39.07	84.06	63
		1996	127	68	161	74	72.02	38.47	91.49	60
		1997	144	72	189	72	74.37	37.52	98.03	56
		1998	161	77	217	70	75.89	36.27	102.69	55
		1999	209	190	246	67	89.58	81.45	105.36	49
		2000	191	85	267	66	73.98	33.04	103.44	56
		2001	243	207	288	67	86.02	73.34	102.20	48
		2002	217	88	311	67	71.40	28.96	102.20	55
		2003	234	90	337	67	71.65	27.67	103.30	54
		2004	260	94	371	67	74.03	26.87	105.52	52
		2005	300	100	417	65	78.66	26.37	109.45	48
		2006	347	107	469	63	85.62	26.53	115.76	43
		2007	409	116	540	62	95.69	27.23	126.27	38

Metropolitan Statistical Area	PWID Population	Year	Number	Min	Max	Rank	Rate	Min	Max	Rank
Sarasota--Bradenton, FL	Male	1992	1,690	1,462	1,915	96	125.72	108.76	142.44	56
		1993	1,641	1,040	1,985	92	120.37	76.27	145.62	56
		1994	2,006	1,926	2,070	91	144.27	138.57	148.92	46
		1995	1,905	1,077	2,317	87	133.92	75.69	162.83	46
		1996	2,039	1,089	2,590	86	140.56	75.08	178.56	43
		1997	2,191	1,106	2,889	85	147.04	74.19	193.83	43
		1998	2,355	1,125	3,187	85	153.43	73.32	207.61	41
		1999	2,968	2,698	3,490	83	187.98	170.93	221.10	24
		2000	2,640	1,179	3,691	84	162.13	72.42	226.68	34
		2001	3,275	2,792	3,891	81	195.25	166.47	231.97	21
		2002	2,847	1,154	4,075	85	164.54	66.72	235.49	27
		2003	2,950	1,139	4,253	85	165.66	63.96	238.84	27
		2004	3,116	1,131	4,441	80	168.84	61.28	240.65	24
		2005	3,350	1,123	4,662	75	174.70	58.56	243.07	21
		2006	3,536	1,095	4,780	73	180.56	55.94	244.12	20
		2007	3,697	1,052	4,878	72	187.11	53.25	246.91	17
	Female	1992	971	840	1,100	91	67.19	58.12	76.12	55
		1993	951	602	1,150	87	65.13	41.27	78.79	53
		1994	1,177	1,131	1,215	86	79.18	76.05	81.73	44
		1995	1,136	642	1,382	86	74.86	42.31	91.02	44
		1996	1,239	662	1,574	86	80.34	42.92	102.07	42
		1997	1,359	686	1,792	85	86.10	43.44	113.50	35
		1998	1,493	713	2,020	83	92.03	43.98	124.53	31
		1999	1,923	1,749	2,262	77	115.48	105.01	135.83	14
		2000	1,748	781	2,444	79	101.95	45.54	142.54	17
		2001	2,213	1,887	2,630	69	125.79	107.25	149.44	13
		2002	1,960	795	2,805	75	108.35	43.94	155.07	17
		2003	2,064	797	2,975	74	111.51	43.05	160.76	17
		2004	2,207	801	3,146	66	115.66	41.98	164.86	14
		2005	2,393	802	3,329	65	121.03	40.57	168.41	12
		2006	2,534	785	3,425	62	126.05	39.06	170.43	9
		2007	2,643	752	3,488	58	130.99	37.28	172.85	8

Metropolitan Statistical Area	PWID Population	Year	Number	Min	Max	Rank	Rate	Min	Max	Rank
Sarasota--Bradenton, FL	Young (15-29)	1992	537	465	609	98	72.60	62.80	82.25	62
		1993	545	345	660	94	74.62	47.29	90.28	55
		1994	711	683	734	91	98.08	94.20	101.24	39
		1995	732	414	890	88	100.91	57.03	122.69	41
		1996	859	459	1,091	85	117.85	62.95	149.71	32
		1997	1,018	514	1,342	82	137.60	69.42	181.38	17
		1998	1,209	578	1,637	79	160.11	76.51	216.65	13
		1999	1,681	1,528	1,977	68	218.58	198.75	257.09	4
		2000	1,640	733	2,293	70	208.63	93.19	291.70	4
		2001	2,212	1,886	2,628	58	269.74	229.98	320.46	3
		2002	2,067	838	2,958	63	240.09	97.36	343.63	4
		2003	2,272	877	3,275	59	250.97	96.90	361.83	4
		2004	2,507	910	3,573	55	261.55	94.93	372.80	4
		2005	2,769	928	3,853	51	272.05	91.19	378.52	3
		2006	2,946	913	3,984	48	279.14	86.49	377.40	2
		2007	3,041	865	4,013	45	282.60	80.43	372.92	4
	Old (30-64)	1992	2,122	1,836	2,404	93	103.54	89.57	117.31	62
		1993	2,048	1,298	2,478	88	97.88	62.02	118.41	59
		1994	2,479	2,381	2,559	87	115.21	110.65	118.92	45
		1995	2,323	1,313	2,824	87	104.86	59.26	127.50	49
		1996	2,439	1,303	3,099	86	107.76	57.56	136.90	47
		1997	2,561	1,292	3,376	86	109.94	55.47	144.93	46
		1998	2,673	1,277	3,617	86	111.30	53.19	150.60	44
		1999	3,254	2,959	3,827	81	131.49	119.56	154.65	34
		2000	2,783	1,243	3,892	84	108.84	48.62	152.18	43
		2001	3,307	2,820	3,929	79	126.37	107.74	150.13	36
		2002	2,748	1,114	3,933	83	102.60	41.60	146.84	42
		2003	2,722	1,051	3,925	83	99.86	38.55	143.96	45
		2004	2,756	1,000	3,928	82	98.60	35.78	140.53	43
		2005	2,855	957	3,972	80	99.24	33.27	138.09	41
		2006	2,927	907	3,957	80	100.48	31.13	135.86	35
		2007	3,006	855	3,966	76	103.03	29.32	135.96	29

Metropolitan Statistical Area	PWID Population	Year	Number	Min	Max	Rank	Rate	Min	Max	Rank
Scranton--Wilkes-Barre--Hazleton, PA	Total	1992	1,671	1,155	1,947	98	41.51	28.71	48.39	98
		1993	1,806	1,173	2,153	98	44.92	29.17	53.54	96
		1994	1,711	1,188	1,999	98	42.64	29.60	49.81	98
		1995	1,874	1,205	2,295	97	46.73	30.03	57.22	95
		1996	1,904	1,219	2,341	97	47.53	30.44	58.44	93
		1997	1,922	1,231	2,354	97	48.18	30.85	59.03	93
		1998	1,946	1,248	2,373	97	48.90	31.36	59.61	92
		1999	1,833	1,267	2,194	99	46.10	31.86	55.17	95
		2000	2,030	1,314	2,421	96	51.17	33.12	61.01	90
		2001	1,970	1,364	2,467	99	49.67	34.39	62.22	95
		2002	2,080	1,433	2,690	98	52.38	36.09	67.76	86
		2003	2,123	1,505	2,919	98	53.30	37.79	73.29	88
		2004	2,170	1,584	3,163	98	54.23	39.58	79.06	88
		2005	2,212	1,576	3,409	99	55.05	39.23	84.82	88
		2006	2,257	1,385	3,661	99	55.97	34.36	90.79	84
		2007	2,303	1,186	3,920	98	56.84	29.29	96.76	84
	Non-Hispanic White	1992	1,357	939	1,582	95	34.61	23.93	40.34	93
		1993	1,466	952	1,747	91	37.45	24.32	44.64	84
		1994	1,400	972	1,636	95	35.88	24.91	41.92	88
		1995	1,556	1,000	1,906	89	39.96	25.68	48.93	81
		1996	1,610	1,031	1,979	87	41.46	26.55	50.98	80
		1997	1,655	1,060	2,027	87	42.87	27.45	52.52	78
		1998	1,705	1,093	2,078	87	44.32	28.42	54.03	76
		1999	1,629	1,126	1,949	88	42.45	29.34	50.81	80
		2000	1,823	1,180	2,174	87	47.72	30.89	56.90	73
		2001	1,780	1,232	2,230	88	46.74	32.36	58.55	77
		2002	1,883	1,297	2,436	87	49.51	34.11	64.05	73
		2003	1,916	1,358	2,634	87	50.39	35.72	69.28	73
		2004	1,936	1,413	2,823	89	50.89	37.15	74.19	72
		2005	1,932	1,376	2,976	88	50.74	36.16	78.18	75
		2006	1,894	1,163	3,073	91	49.85	30.60	80.86	80
		2007	1,804	929	3,070	92	47.53	24.49	80.91	83

Metropolitan Statistical Area	PWID Population	Year	Number	Min	Max	Rank	Rate	Min	Max	Rank
Scranton--Wilkes-Barre--Hazleton, PA	Non-Hispanic Black	1992	192	133	224	99	405.14	280.20	472.29	28
		1993	199	129	237	99	404.75	262.85	482.42	23
		1994	175	122	205	99	348.46	241.94	407.09	33
		1995	174	112	213	99	332.06	213.43	406.64	27
		1996	157	101	193	99	281.49	180.29	346.12	36
		1997	139	89	170	99	239.57	153.41	293.52	44
		1998	122	78	149	99	203.80	130.69	248.44	48
		1999	100	69	120	99	161.04	111.31	192.74	68
		2000	97	63	115	100	149.98	97.10	178.84	65
		2001	83	57	104	100	122.84	85.05	153.89	75
		2002	79	55	102	100	111.40	76.75	144.10	71
		2003	75	53	103	100	99.53	70.56	136.86	74
		2004	74	54	108	100	92.63	67.62	135.04	77
		2005	76	54	117	100	90.38	64.40	139.26	78
		2006	82	50	133	100	91.08	55.91	147.75	78
		2007	94	48	160	100	97.27	50.12	165.58	75
	Hispanic	1992	58	40	68	84	211.44	146.23	246.48	31
		1993	50	32	60	85	169.75	110.24	202.33	37
		1994	39	27	45	88	124.72	86.60	145.71	44
		1995	35	23	43	91	106.08	68.18	129.91	48
		1996	31	20	38	91	85.40	54.70	105.01	53
		1997	27	18	34	93	70.76	45.31	86.69	59
		1998	25	16	31	93	60.20	38.60	73.38	65
		1999	22	15	26	96	48.44	33.48	57.97	75
		2000	23	15	27	96	45.89	29.71	54.72	69
		2001	21	15	26	96	38.88	26.92	48.71	77
		2002	22	15	28	96	36.62	25.23	47.37	74
		2003	22	16	30	96	32.48	23.03	44.67	78
		2004	23	17	33	96	29.47	21.51	42.97	79
		2005	24	17	37	96	27.15	19.34	41.83	83
		2006	26	16	42	96	25.63	15.73	41.58	86
		2007	28	15	48	96	24.19	12.46	41.17	91

Metropolitan Statistical Area	PWID Population	Year	Number	Min	Max	Rank	Rate	Min	Max	Rank
Scranton--Wilkes-Barre--Hazleton, PA	Male	1992	1,233	853	1,438	97	62.12	42.96	72.42	98
		1993	1,314	853	1,566	97	66.12	42.94	78.81	94
		1994	1,228	853	1,434	97	61.81	42.91	72.21	98
		1995	1,328	853	1,626	96	66.79	42.93	81.79	91
		1996	1,332	853	1,638	95	67.02	42.93	82.41	91
		1997	1,330	852	1,629	95	67.09	42.96	82.20	89
		1998	1,333	855	1,625	95	67.32	43.17	82.07	87
		1999	1,245	860	1,490	98	62.79	43.40	75.15	95
		2000	1,367	885	1,631	94	69.04	44.70	82.33	84
		2001	1,318	912	1,650	98	66.58	46.10	83.41	91
		2002	1,383	953	1,789	95	69.75	48.05	90.22	81
		2003	1,406	997	1,933	95	70.58	50.03	97.04	82
		2004	1,432	1,045	2,088	96	71.51	52.20	104.26	80
		2005	1,457	1,038	2,245	98	72.47	51.64	111.66	80
		2006	1,485	912	2,409	98	73.54	45.14	119.29	79
		2007	1,515	781	2,579	98	74.66	38.47	127.09	77
	Female	1992	479	331	558	100	23.48	16.24	27.37	100
		1993	532	345	634	99	26.16	16.99	31.18	97
		1994	518	359	605	100	25.55	17.74	29.85	100
		1995	582	374	713	97	28.79	18.51	35.26	93
		1996	607	389	747	97	30.09	19.27	37.00	94
		1997	628	402	769	98	31.28	20.03	38.32	94
		1998	650	417	792	97	32.49	20.84	39.61	93
		1999	624	431	747	98	31.28	21.62	37.43	94
		2000	702	455	837	97	35.33	22.87	42.12	93
		2001	690	477	864	98	34.72	24.03	43.49	92
		2002	734	506	950	97	36.95	25.46	47.80	91
		2003	752	533	1,034	97	37.78	26.78	51.95	91
		2004	767	560	1,119	97	38.40	28.03	55.98	88
		2005	777	553	1,197	98	38.68	27.56	59.60	89
		2006	782	480	1,268	98	38.83	23.84	62.99	88
		2007	781	402	1,330	98	38.65	19.91	65.79	87

Metropolitan Statistical Area	PWID Population	Year	Number	Min	Max	Rank	Rate	Min	Max	Rank
Scranton--Wilkes-Barre--Hazleton, PA	Young (15-29)	1992	542	375	632	97	41.89	28.97	48.84	87
		1993	607	394	723	91	47.69	30.97	56.84	76
		1994	611	424	714	92	48.94	33.98	57.17	79
		1995	725	466	888	89	58.80	37.79	72.01	64
		1996	806	516	992	88	66.12	42.35	81.30	59
		1997	893	572	1,094	86	74.23	47.54	90.95	58
		1998	989	634	1,205	85	83.10	53.29	101.30	53
		1999	1,009	697	1,208	86	86.14	59.54	103.10	53
		2000	1,196	774	1,426	84	103.59	67.07	123.53	43
		2001	1,224	847	1,533	86	106.44	73.69	133.34	40
		2002	1,342	925	1,736	85	116.44	80.22	150.62	38
		2003	1,400	993	1,926	84	120.37	85.34	165.52	35
		2004	1,437	1,049	2,095	86	121.83	88.93	177.61	29
		2005	1,441	1,027	2,220	86	120.55	85.90	185.74	31
		2006	1,409	865	2,285	85	116.70	71.63	189.30	34
		2007	1,329	685	2,262	85	108.90	56.11	185.38	39
	Old (30-64)	1992	1,199	829	1,398	97	43.91	30.37	51.18	99
		1993	1,253	814	1,494	97	45.60	29.61	54.35	96
		1994	1,138	790	1,329	99	41.16	28.57	48.08	99
		1995	1,183	760	1,449	99	42.60	27.38	52.17	97
		1996	1,131	725	1,391	99	40.60	26.01	49.92	98
		1997	1,066	683	1,306	99	38.26	24.50	46.87	98
		1998	1,001	642	1,220	99	35.87	23.00	43.72	98
		1999	871	602	1,042	99	31.03	21.45	37.14	99
		2000	890	576	1,062	99	31.64	20.48	37.73	99
		2001	801	554	1,003	100	28.43	19.68	35.62	100
		2002	791	545	1,023	100	28.06	19.33	36.29	100
		2003	765	542	1,052	100	27.13	19.24	37.31	100
		2004	755	551	1,101	100	26.76	19.53	39.01	100
		2005	761	542	1,172	100	26.94	19.19	41.51	100
		2006	788	484	1,278	99	27.88	17.11	45.22	99
		2007	841	433	1,432	99	29.72	15.31	50.59	98

Metropolitan Statistical Area	PWID Population	Year	Number	Min	Max	Rank	Rate	Min	Max	Rank
Seattle--Bellevue--Everett, WA	Total	1992	17,628	2,555	33,130	26	120.48	17.46	226.43	45
		1993	23,643	2,852	42,677	17	158.21	19.08	285.58	26
		1994	16,856	3,167	29,574	25	111.24	20.90	195.18	46
		1995	22,325	3,501	39,948	17	144.85	22.72	259.19	31
		1996	21,814	3,908	38,646	17	139.04	24.91	246.33	32
		1997	21,484	4,371	37,640	18	133.21	27.10	233.38	33
		1998	21,567	4,908	36,362	17	130.75	29.75	220.44	30
		1999	17,037	5,419	25,974	21	101.87	32.40	155.31	50
		2000	22,222	6,811	33,276	17	131.36	40.26	196.71	29
		2001	20,181	8,280	31,957	19	117.28	48.12	185.72	39
		2002	24,137	9,792	34,972	14	139.00	56.39	201.39	21
		2003	24,943	11,284	37,883	14	142.93	64.66	217.07	18
		2004	25,689	12,920	40,226	13	145.83	73.35	228.35	17
		2005	26,550	14,646	42,784	14	148.71	82.03	239.64	17
		2006	27,528	16,628	45,370	13	151.46	91.49	249.62	17
		2007	28,402	18,589	47,810	11	154.22	100.94	259.61	16
	Non-Hispanic White	1992	11,814	1,712	22,202	17	95.86	13.89	180.15	39
		1993	15,938	1,922	28,770	8	127.68	15.40	230.47	25
		1994	11,396	2,141	19,994	15	90.85	17.07	159.40	43
		1995	15,096	2,367	27,013	8	119.44	18.73	213.72	25
		1996	14,718	2,637	26,074	8	115.52	20.69	204.66	26
		1997	14,431	2,936	25,283	8	111.26	22.63	194.92	26
		1998	14,393	3,275	24,268	8	109.55	24.93	184.71	24
		1999	11,275	3,586	17,190	16	85.51	27.20	130.37	44
		2000	14,558	4,462	21,799	7	110.22	33.78	165.04	25
		2001	13,065	5,360	20,688	14	97.96	40.19	155.12	35
		2002	15,417	6,254	22,338	7	115.36	46.80	167.14	20
		2003	15,696	7,101	23,839	7	117.63	53.21	178.66	19
		2004	15,907	8,000	24,909	7	118.88	59.79	186.16	18
		2005	16,160	8,914	26,041	6	119.97	66.18	193.33	20
		2006	16,456	9,940	27,122	8	120.89	73.02	199.25	20
		2007	16,666	10,908	28,055	8	121.69	79.65	204.85	20

Metropolitan Statistical Area	PWID Population	Year	Number	Min	Max	Rank	Rate	Min	Max	Rank
Seattle--Bellevue--Everett, WA	Non-Hispanic Black	1992	3,344	485	6,284	31	572.66	83.00	1076.23	12
		1993	4,430	534	7,997	23	729.17	87.95	1316.21	5
		1994	3,094	581	5,428	32	489.06	91.88	858.06	13
		1995	3,986	625	7,132	24	605.39	94.94	1083.28	6
		1996	3,770	675	6,680	24	546.69	97.93	968.53	7
		1997	3,586	730	6,283	23	492.70	100.23	863.19	8
		1998	3,475	791	5,860	23	463.79	105.53	781.96	9
		1999	2,656	845	4,049	31	344.44	109.55	525.13	14
		2000	3,367	1,032	5,042	23	424.40	130.07	635.53	8
		2001	2,992	1,228	4,738	25	365.47	149.94	578.72	8
		2002	3,535	1,434	5,121	21	422.67	171.47	612.41	7
		2003	3,651	1,652	5,545	19	429.58	194.34	652.44	6
		2004	3,813	1,918	5,970	18	439.26	220.92	687.83	6
		2005	4,064	2,242	6,549	17	454.87	250.92	733.00	6
		2006	4,433	2,677	7,306	17	481.14	290.63	792.99	5
		2007	4,918	3,219	8,279	16	521.87	341.56	878.48	5
	Hispanic	1992	706	102	1,327	45	155.04	22.47	291.38	44
		1993	885	107	1,597	40	178.56	21.54	322.32	36
		1994	601	113	1,054	49	112.54	21.14	197.46	51
		1995	771	121	1,380	45	133.01	20.86	238.00	40
		1996	742	133	1,315	48	117.31	21.01	207.83	41
		1997	731	149	1,281	49	104.56	21.27	183.18	42
		1998	744	169	1,255	49	97.87	22.27	165.01	44
		1999	604	192	920	53	73.76	23.46	112.46	58
		2000	818	251	1,225	47	93.65	28.70	140.23	47
		2001	780	320	1,235	49	83.76	34.36	132.64	49
		2002	989	401	1,432	47	101.59	41.21	147.20	42
		2003	1,093	494	1,660	46	108.31	49.00	164.51	35
		2004	1,214	611	1,901	46	115.33	58.01	180.60	33
		2005	1,365	753	2,199	41	123.71	68.24	199.35	25
		2006	1,551	937	2,557	41	133.50	80.64	220.03	24
		2007	1,769	1,158	2,978	37	145.89	95.48	245.58	24

Metropolitan Statistical Area	PWID Population	Year	Number	Min	Max	Rank	Rate	Min	Max	Rank
Seattle--Bellevue--Everett, WA	Male	1992	10,512	1,524	19,757	32	142.54	20.66	267.88	48
		1993	14,229	1,716	25,684	16	188.72	22.76	340.65	34
		1994	10,198	1,916	17,892	28	133.34	25.05	233.94	49
		1995	13,535	2,123	24,219	17	173.89	27.27	311.15	35
		1996	13,221	2,368	23,422	17	166.65	29.85	295.24	37
		1997	12,995	2,644	22,767	17	159.24	32.40	278.99	38
		1998	13,005	2,959	21,926	17	155.89	35.47	262.83	37
		1999	10,238	3,256	15,609	25	120.97	38.47	184.42	53
		2000	13,312	4,080	19,935	18	155.44	47.64	232.76	37
		2001	12,065	4,950	19,105	19	138.31	56.75	219.02	41
		2002	14,426	5,852	20,902	15	163.95	66.51	237.56	28
		2003	14,940	6,758	22,690	15	169.13	76.51	256.87	25
		2004	15,468	7,780	24,222	15	173.71	87.37	272.00	22
		2005	16,132	8,899	25,995	14	178.57	98.51	287.76	20
		2006	16,950	10,238	27,936	13	184.16	111.24	303.52	17
		2007	17,804	11,653	29,970	13	190.99	125.01	321.51	15
	Female	1992	6,986	1,013	13,129	22	96.28	13.96	180.94	37
		1993	9,295	1,121	16,779	14	125.54	15.14	226.61	18
		1994	6,619	1,244	11,614	22	88.20	16.57	154.75	37
		1995	8,803	1,381	15,752	16	115.38	18.10	206.47	18
		1996	8,669	1,553	15,359	17	111.78	20.03	198.04	17
		1997	8,626	1,755	15,112	17	108.26	22.02	189.67	18
		1998	8,754	1,992	14,760	17	107.38	24.43	181.04	16
		1999	6,987	2,222	10,652	19	84.59	26.90	128.96	40
		2000	9,188	2,816	13,759	13	110.01	33.72	164.74	15
		2001	8,384	3,440	13,277	17	98.82	40.54	156.49	26
		2002	10,026	4,067	14,527	12	117.04	47.48	169.59	13
		2003	10,295	4,657	15,636	11	119.46	54.04	181.43	12
		2004	10,454	5,258	16,369	11	120.01	60.36	187.92	11
		2005	10,551	5,820	17,002	9	119.63	65.99	192.77	13
		2006	10,562	6,380	17,408	9	117.73	71.11	194.03	15
		2007	10,378	6,793	17,471	9	114.12	74.69	192.10	15

Metropolitan Statistical Area	PWID Population	Year	Number	Min	Max	Rank	Rate	Min	Max	Rank
Seattle--Bellevue--Everett, WA	Young (15-29)	1992	4,845	702	9,105	24	105.80	15.34	198.83	37
		1993	6,442	777	11,628	15	140.92	17.00	254.37	20
		1994	4,566	858	8,010	23	100.42	18.86	176.18	38
		1995	6,019	944	10,771	14	131.61	20.64	235.50	23
		1996	5,855	1,049	10,372	15	126.00	22.57	223.23	24
		1997	5,733	1,166	10,044	14	119.93	24.40	210.11	29
		1998	5,707	1,299	9,622	15	116.67	26.55	196.71	33
		1999	4,453	1,416	6,789	23	89.80	28.56	136.91	49
		2000	5,708	1,749	8,547	17	114.09	34.97	170.85	35
		2001	5,059	2,076	8,012	20	101.20	41.52	160.25	46
		2002	5,858	2,376	8,488	17	118.39	48.03	171.53	35
		2003	5,804	2,626	8,815	18	118.93	53.80	180.63	37
		2004	5,667	2,850	8,875	19	116.91	58.80	183.06	36
		2005	5,481	3,024	8,833	22	112.79	62.22	181.75	38
		2006	5,241	3,166	8,638	24	106.37	64.25	175.32	42
		2007	4,905	3,210	8,256	25	99.25	64.96	167.08	46
	Old (30-64)	1992	13,406	1,943	25,194	25	133.36	19.33	250.64	44
		1993	17,326	2,090	31,274	15	167.03	20.15	301.51	27
		1994	12,127	2,278	21,277	28	114.34	21.48	200.62	46
		1995	16,042	2,516	28,706	15	148.00	23.21	264.83	33
		1996	15,859	2,841	28,097	15	143.62	25.73	254.45	33
		1997	15,923	3,239	27,896	15	140.32	28.55	245.83	33
		1998	16,331	3,716	27,535	15	140.74	32.03	237.29	31
		1999	13,154	4,184	20,055	22	111.81	35.56	170.47	47
		2000	17,401	5,333	26,057	15	146.05	44.76	218.71	23
		2001	15,897	6,522	25,174	17	130.22	53.43	206.21	32
		2002	18,919	7,675	27,412	14	152.37	61.81	220.76	17
		2003	19,161	8,668	29,101	12	152.42	68.95	231.49	14
		2004	18,910	9,511	29,611	13	148.10	74.49	231.91	14
		2005	18,058	9,961	29,099	13	138.98	76.66	223.95	14
		2006	16,263	9,824	26,804	15	122.76	74.15	202.32	21
		2007	13,132	8,595	22,106	20	97.46	63.79	164.05	34

Metropolitan Statistical Area	PWID Population	Year	Number	Min	Max	Rank	Rate	Min	Max	Rank
Springfield, MA	Total	1992	5,502	4,144	6,793	73	140.02	105.45	172.86	39
		1993	6,307	4,276	8,164	63	161.02	109.15	208.42	25
		1994	5,903	4,454	7,144	70	150.66	113.68	182.33	30
		1995	6,969	4,680	9,454	58	177.70	119.34	241.07	15
		1996	7,296	4,929	10,040	55	186.03	125.68	256.01	11
		1997	7,771	5,670	10,625	48	196.97	143.73	269.30	8
		1998	8,234	6,427	11,199	47	207.76	162.17	282.58	7
		1999	7,639	7,026	8,038	55	191.40	176.05	201.40	9
		2000	8,998	7,352	12,394	44	223.92	182.96	308.44	6
		2001	7,974	7,331	8,718	50	197.45	181.52	215.89	7
		2002	9,327	7,355	13,106	43	228.23	179.97	320.69	6
		2003	9,595	7,321	13,498	42	232.32	177.25	326.82	5
		2004	9,978	6,990	13,869	42	239.82	168.01	333.35	5
		2005	10,371	6,656	14,220	40	247.39	158.78	339.22	4
		2006	10,771	6,277	14,569	40	255.13	148.68	345.08	3
		2007	11,149	5,873	14,877	40	263.06	138.58	351.04	3
	Non-Hispanic White	1992	2,499	1,882	3,084	75	75.73	57.03	93.49	49
		1993	2,750	1,864	3,559	71	84.12	57.02	108.88	42
		1994	2,485	1,875	3,008	75	76.42	57.66	92.48	49
		1995	2,850	1,914	3,866	69	88.03	59.12	119.43	44
		1996	2,914	1,968	4,010	69	90.58	61.20	124.65	44
		1997	3,046	2,223	4,165	68	94.82	69.19	129.64	38
		1998	3,182	2,484	4,329	67	99.31	77.52	135.07	36
		1999	2,923	2,689	3,076	75	91.18	83.87	95.94	36
		2000	3,422	2,796	4,714	61	106.80	87.26	147.11	28
		2001	3,023	2,780	3,306	74	94.42	86.80	103.23	37
		2002	3,536	2,788	4,968	61	109.71	86.52	154.16	25
		2003	3,644	2,780	5,127	59	112.45	85.79	158.19	25
		2004	3,803	2,665	5,287	61	117.10	82.04	162.78	20
		2005	3,972	2,549	5,446	57	122.04	78.32	167.33	18
		2006	4,148	2,417	5,610	58	127.35	74.22	172.25	16
		2007	4,318	2,275	5,762	56	132.82	69.97	177.23	14

Metropolitan Statistical Area	PWID Population	Year	Number	Min	Max	Rank	Rate	Min	Max	Rank
Springfield, MA	Non-Hispanic Black	1992	843	635	1,040	83	360.81	271.73	445.42	35
		1993	950	644	1,230	76	405.49	274.87	524.86	22
		1994	844	637	1,022	81	357.91	270.06	433.13	28
		1995	919	617	1,247	74	390.14	262.02	529.28	15
		1996	869	587	1,195	72	366.01	247.27	503.69	16
		1997	823	600	1,125	72	338.92	247.31	463.37	19
		1998	771	601	1,048	74	313.26	244.52	426.07	18
		1999	633	582	666	82	253.80	233.45	267.06	26
		2000	668	546	920	76	262.51	214.49	361.60	21
		2001	541	497	591	82	209.59	192.68	229.16	35
		2002	595	469	836	76	227.15	179.12	319.18	24
		2003	599	457	843	76	225.28	171.88	316.91	24
		2004	639	448	889	75	237.83	166.62	330.59	20
		2005	722	463	990	73	264.50	169.76	362.67	16
		2006	869	507	1,176	70	315.00	183.57	426.05	13
		2007	1,120	590	1,495	62	402.59	212.08	537.23	11
	Hispanic	1992	2,016	1,518	2,488	33	626.96	472.16	773.98	4
		1993	2,439	1,653	3,157	31	727.24	492.97	941.32	3
		1994	2,371	1,789	2,870	29	680.47	513.44	823.48	4
		1995	2,870	1,927	3,893	28	788.69	529.67	1069.94	3
		1996	3,045	2,057	4,190	25	801.04	541.17	1102.36	3
		1997	3,256	2,376	4,452	24	815.86	595.33	1115.45	1
		1998	3,436	2,682	4,673	22	820.37	640.36	1115.81	1
		1999	3,153	2,900	3,318	23	719.08	661.42	756.65	3
		2000	3,655	2,986	5,034	18	797.33	651.48	1098.31	1
		2001	3,174	2,918	3,470	23	669.13	615.16	731.61	3
		2002	3,628	2,861	5,098	19	734.02	578.82	1031.40	1
		2003	3,643	2,779	5,124	18	710.68	542.22	999.76	1
		2004	3,698	2,591	5,140	19	696.80	488.16	968.55	1
		2005	3,759	2,413	5,155	19	683.64	438.76	937.38	1
		2006	3,834	2,234	5,186	18	669.82	390.34	905.97	1
		2007	3,920	2,065	5,232	19	661.90	348.69	883.26	1

Metropolitan Statistical Area	PWID Population	Year	Number	Min	Max	Rank	Rate	Min	Max	Rank
Springfield, MA	Male	1992	4,009	3,019	4,950	69	211.55	159.32	261.16	28
		1993	4,535	3,074	5,869	57	240.04	162.71	310.70	18
		1994	4,211	3,177	5,096	66	222.72	168.05	269.53	22
		1995	4,956	3,328	6,723	51	261.99	175.95	355.41	9
		1996	5,195	3,509	7,149	46	274.69	185.58	378.01	8
		1997	5,558	4,056	7,599	44	292.11	213.15	399.38	5
		1998	5,929	4,628	8,065	41	310.22	242.15	421.93	5
		1999	5,546	5,101	5,836	47	288.13	265.03	303.19	7
		2000	6,588	5,383	9,075	38	339.92	277.75	468.24	2
		2001	5,886	5,411	6,436	46	301.80	277.46	329.98	6
		2002	6,933	5,467	9,741	37	350.59	276.47	492.63	2
		2003	7,166	5,468	10,081	37	358.21	273.30	503.92	2
		2004	7,469	5,232	10,382	37	370.54	259.59	515.05	2
		2005	7,753	4,976	10,631	37	381.66	244.95	523.31	2
		2006	8,009	4,667	10,833	36	390.75	227.71	528.51	2
		2007	8,202	4,321	10,945	33	398.50	209.93	531.76	1
	Female	1992	1,454	1,095	1,795	80	71.48	53.83	88.24	48
		1993	1,747	1,184	2,262	74	86.15	58.40	111.52	35
		1994	1,686	1,272	2,041	78	83.16	62.75	100.64	40
		1995	2,024	1,359	2,746	68	99.72	66.97	135.28	26
		1996	2,131	1,439	2,932	66	104.92	70.88	144.39	21
		1997	2,260	1,649	3,090	63	110.64	80.73	151.27	15
		1998	2,367	1,847	3,219	61	115.35	90.04	156.89	14
		1999	2,158	1,985	2,271	69	104.44	96.06	109.89	21
		2000	2,489	2,034	3,429	59	119.66	97.78	164.84	13
		2001	2,156	1,982	2,358	71	103.27	94.94	112.91	23
		2002	2,466	1,945	3,466	64	116.92	92.20	164.29	14
		2003	2,487	1,897	3,498	63	116.77	89.09	164.27	14
		2004	2,546	1,784	3,539	61	118.71	83.16	165.00	12
		2005	2,623	1,684	3,597	59	121.43	77.93	166.50	11
		2006	2,726	1,588	3,687	56	125.49	73.13	169.73	11
		2007	2,855	1,504	3,809	54	130.95	68.98	174.74	9

Metropolitan Statistical Area	PWID Population	Year	Number	Min	Max	Rank	Rate	Min	Max	Rank
Springfield, MA	Young (15-29)	1992	1,653	1,245	2,040	68	113.63	85.57	140.27	32
		1993	1,924	1,304	2,490	51	135.55	91.88	175.45	22
		1994	1,840	1,389	2,227	59	132.15	99.71	159.92	22
		1995	2,231	1,498	3,027	45	162.93	109.42	221.03	9
		1996	2,406	1,626	3,312	44	178.63	120.68	245.82	7
		1997	2,644	1,930	3,616	36	197.81	144.34	270.44	3
		1998	2,891	2,256	3,932	34	217.11	169.47	295.30	2
		1999	2,762	2,540	2,906	43	208.27	191.57	219.15	6
		2000	3,340	2,729	4,601	35	251.78	205.72	346.82	2
		2001	3,026	2,782	3,309	42	227.22	208.89	248.44	6
		2002	3,598	2,838	5,056	33	264.87	208.87	372.18	3
		2003	3,737	2,851	5,257	33	268.30	204.70	377.44	3
		2004	3,890	2,725	5,407	34	272.99	191.25	379.46	3
		2005	4,009	2,573	5,497	34	275.17	176.60	377.30	2
		2006	4,082	2,379	5,521	33	274.38	159.90	371.12	3
		2007	4,090	2,155	5,458	34	271.38	142.96	362.13	5
	Old (30-64)	1992	3,958	2,981	4,887	74	159.94	120.45	197.44	37
		1993	4,455	3,020	5,767	65	178.36	120.90	230.86	24
		1994	4,104	3,097	4,967	75	162.50	122.61	196.65	34
		1995	4,779	3,210	6,484	63	187.26	125.76	254.04	20
		1996	4,945	3,341	6,805	61	192.07	129.76	264.32	15
		1997	5,212	3,803	7,126	57	199.81	145.80	273.19	9
		1998	5,468	4,268	7,437	55	207.76	162.17	282.59	8
		1999	5,022	4,620	5,285	60	188.47	173.36	198.32	9
		2000	5,854	4,783	8,064	46	217.50	177.72	299.61	7
		2001	5,128	4,714	5,607	56	189.47	174.18	207.16	8
		2002	5,919	4,667	8,316	45	216.93	171.06	304.81	7
		2003	5,993	4,572	8,431	44	218.92	167.03	307.97	6
		2004	6,115	4,284	8,499	43	223.52	156.59	310.69	6
		2005	6,209	3,985	8,514	42	227.03	145.70	311.29	6
		2006	6,267	3,652	8,476	39	229.22	133.58	310.03	6
		2007	6,259	3,297	8,352	37	229.19	120.73	305.83	6

Metropolitan Statistical Area	PWID Population	Year	Number	Min	Max	Rank	Rate	Min	Max	Rank
Stockton--Lodi, CA	Total	1992	7,355	5,576	10,254	58	235.41	178.46	328.21	10
		1993	7,789	6,121	9,220	52	247.49	194.51	292.98	5
		1994	7,294	6,678	7,711	60	230.15	210.70	243.29	7
		1995	7,819	6,495	9,325	48	244.42	203.04	291.50	4
		1996	7,906	5,457	9,324	47	244.23	168.59	288.04	4
		1997	8,316	5,382	9,661	46	251.34	162.67	291.98	3
		1998	8,833	5,564	10,511	44	260.67	164.18	310.16	4
		1999	9,348	5,653	11,469	45	267.61	161.85	328.35	3
		2000	9,915	5,522	12,451	40	274.23	152.71	344.35	2
		2001	10,899	5,127	13,893	39	287.19	135.11	366.10	3
		2002	10,773	4,313	15,586	40	273.82	109.63	396.17	2
		2003	11,145	3,436	17,453	38	273.75	84.39	428.71	2
		2004	11,679	3,155	19,474	38	278.21	75.16	463.92	2
		2005	12,234	2,872	21,659	35	283.57	66.57	502.02	2
		2006	12,645	2,577	23,704	34	289.48	58.99	542.65	2
		2007	13,053	2,314	25,752	33	295.81	52.44	583.59	2
	Non-Hispanic White	1992	3,674	2,785	5,123	63	203.47	154.24	283.68	11
		1993	3,819	3,001	4,520	58	214.88	168.89	254.38	4
		1994	3,534	3,235	3,736	64	201.70	184.65	213.22	7
		1995	3,762	3,125	4,487	57	217.03	180.28	258.83	4
		1996	3,790	2,616	4,470	58	220.13	151.96	259.62	4
		1997	3,977	2,574	4,620	49	229.69	148.66	266.83	4
		1998	4,210	2,652	5,010	48	240.93	151.75	286.67	3
		1999	4,430	2,679	5,435	47	249.60	150.95	306.24	2
		2000	4,651	2,590	5,840	41	257.12	143.19	322.87	2
		2001	5,027	2,365	6,409	41	271.82	127.88	346.50	2
		2002	4,846	1,940	7,011	41	260.57	104.32	376.99	2
		2003	4,836	1,491	7,574	42	258.27	79.62	404.46	2
		2004	4,824	1,303	8,043	43	256.29	69.24	427.36	2
		2005	4,732	1,111	8,377	43	251.33	59.00	444.93	2
		2006	4,489	915	8,415	53	241.92	49.30	453.51	3
		2007	4,154	737	8,196	59	226.51	40.16	446.87	4

Metropolitan Statistical Area	PWID Population	Year	Number	Min	Max	Rank	Rate	Min	Max	Rank
Stockton--Lodi, CA	Non-Hispanic Black	1992	1,142	866	1,593	73	669.93	507.86	934.01	6
		1993	1,276	1,003	1,510	64	713.61	560.86	844.77	6
		1994	1,236	1,132	1,307	66	668.49	611.98	706.66	4
		1995	1,350	1,122	1,610	63	699.39	580.98	834.11	4
		1996	1,376	950	1,622	62	685.20	473.00	808.12	4
		1997	1,447	936	1,681	59	685.07	443.38	795.83	2
		1998	1,530	964	1,821	57	691.53	435.56	822.83	2
		1999	1,611	975	1,977	53	690.26	417.46	846.92	2
		2000	1,706	950	2,142	47	694.48	386.75	872.07	2
		2001	1,884	886	2,401	44	708.30	333.22	902.92	2
		2002	1,889	756	2,734	39	667.67	267.32	966.01	2
		2003	2,010	620	3,148	34	672.76	207.40	1053.58	2
		2004	2,202	595	3,673	33	705.58	190.61	1176.56	3
		2005	2,460	578	4,356	28	750.86	176.26	1329.28	3
		2006	2,772	565	5,196	26	816.30	166.34	1530.24	3
		2007	3,190	566	6,294	25	911.15	161.53	1797.57	3
	Hispanic	1992	2,262	1,715	3,153	31	290.29	220.07	404.73	19
		1993	2,204	1,732	2,609	32	271.96	213.74	321.94	20
		1994	1,922	1,760	2,032	35	228.82	209.48	241.89	24
		1995	1,943	1,614	2,317	33	223.62	185.76	266.69	22
		1996	1,877	1,295	2,213	33	208.20	143.72	245.55	21
		1997	1,910	1,236	2,218	33	203.05	131.41	235.88	20
		1998	1,987	1,252	2,365	33	202.50	127.54	240.94	18
		1999	2,086	1,261	2,559	34	202.52	122.48	248.48	19
		2000	2,219	1,236	2,787	31	204.39	113.82	256.66	18
		2001	2,475	1,165	3,156	29	211.91	99.69	270.13	17
		2002	2,509	1,005	3,631	26	201.60	80.72	291.68	17
		2003	2,690	829	4,212	26	204.16	62.94	319.72	17
		2004	2,949	797	4,917	25	212.52	57.41	354.38	17
		2005	3,262	766	5,775	22	224.25	52.64	397.01	15
		2006	3,591	732	6,732	21	238.96	48.69	447.95	15
		2007	3,980	706	7,852	17	257.31	45.62	507.63	15

Metropolitan Statistical Area	PWID Population	Year	Number	Min	Max	Rank	Rate	Min	Max	Rank
Stockton--Lodi, CA	Male	1992	4,707	3,568	6,563	59	293.01	222.13	408.52	14
		1993	4,855	3,816	5,748	53	299.98	235.77	355.12	7
		1994	4,445	4,070	4,699	62	273.57	250.45	289.20	14
		1995	4,677	3,885	5,578	54	285.39	237.07	340.37	6
		1996	4,662	3,218	5,498	55	281.63	194.41	332.15	6
		1997	4,855	3,142	5,640	52	287.36	185.98	333.82	6
		1998	5,128	3,230	6,102	47	297.06	187.10	353.46	6
		1999	5,421	3,279	6,651	48	305.32	184.65	374.62	5
		2000	5,768	3,212	7,243	41	313.99	174.85	394.28	5
		2001	6,386	3,004	8,141	40	331.23	155.83	422.25	3
		2002	6,383	2,555	9,234	40	319.40	127.88	462.12	4
		2003	6,699	2,065	10,492	39	324.29	99.97	507.86	4
		2004	7,145	1,930	11,914	39	336.10	90.80	560.44	4
		2005	7,638	1,793	13,522	39	349.03	81.94	617.91	3
		2006	8,074	1,645	15,135	35	364.39	74.25	683.08	3
		2007	8,540	1,514	16,848	31	381.36	67.61	752.37	3
	Female	1992	2,704	2,050	3,769	57	178.12	135.03	248.34	5
		1993	2,981	2,343	3,528	45	195.01	153.27	230.85	4
		1994	2,889	2,645	3,054	52	187.09	171.27	197.77	2
		1995	3,187	2,648	3,801	43	204.30	169.71	243.65	3
		1996	3,298	2,276	3,889	41	208.46	143.90	245.86	3
		1997	3,529	2,284	4,099	40	217.93	141.04	253.16	2
		1998	3,791	2,388	4,511	37	228.06	143.64	271.36	2
		1999	4,034	2,440	4,949	38	234.85	142.03	288.15	2
		2000	4,276	2,381	5,369	36	240.39	133.87	301.86	2
		2001	4,667	2,195	5,949	36	249.97	117.60	318.65	2
		2002	4,549	1,821	6,582	34	234.99	94.08	339.99	2
		2003	4,608	1,421	7,217	33	229.80	70.84	359.87	2
		2004	4,691	1,267	7,822	33	226.41	61.16	377.53	2
		2005	4,733	1,111	8,379	32	222.62	52.26	394.12	2
		2006	4,668	951	8,750	35	216.85	44.19	406.51	2
		2007	4,550	807	8,977	35	209.34	37.11	412.99	2

Metropolitan Statistical Area	PWID Population	Year	Number	Min	Max	Rank	Rate	Min	Max	Rank
Stockton--Lodi, CA	Young (15-29)	1992	1,808	1,371	2,521	63	164.87	124.98	229.86	15
		1993	1,715	1,348	2,030	58	157.53	123.81	186.48	13
		1994	1,477	1,353	1,562	72	136.84	125.27	144.65	19
		1995	1,492	1,240	1,780	68	137.69	114.38	164.22	18
		1996	1,449	1,001	1,709	69	132.54	91.49	156.31	21
		1997	1,486	961	1,726	68	132.87	86.00	154.36	20
		1998	1,551	977	1,846	69	135.36	85.25	161.06	21
		1999	1,620	979	1,987	72	136.93	82.81	168.00	23
		2000	1,691	942	2,123	68	137.85	76.77	173.10	22
		2001	1,815	854	2,313	71	139.29	65.53	177.56	26
		2002	1,727	692	2,499	74	126.44	50.62	182.94	27
		2003	1,687	520	2,642	75	118.08	36.40	184.92	38
		2004	1,626	439	2,712	76	109.29	29.53	182.24	43
		2005	1,516	356	2,684	81	98.20	23.05	173.84	53
		2006	1,339	273	2,511	87	85.40	17.40	160.09	60
		2007	1,127	200	2,223	89	71.26	12.63	140.60	74
	Old (30-64)	1992	5,578	4,229	7,777	57	275.12	208.57	383.58	6
		1993	6,099	4,793	7,220	48	296.26	232.84	350.71	4
		1994	5,844	5,350	6,178	56	279.68	256.04	295.65	4
		1995	6,369	5,291	7,596	46	301.13	250.14	359.13	3
		1996	6,519	4,500	7,689	45	304.13	209.94	358.68	3
		1997	6,919	4,478	8,037	43	315.83	204.41	366.89	2
		1998	7,399	4,660	8,804	39	329.92	207.80	392.56	2
		1999	7,872	4,761	9,658	39	340.72	206.06	418.05	3
		2000	8,390	4,672	10,535	34	351.15	195.55	440.95	2
		2001	9,269	4,360	11,815	33	371.91	174.97	474.11	1
		2002	9,217	3,690	13,335	29	358.90	143.69	519.27	1
		2003	9,608	2,962	15,047	27	363.64	112.10	569.47	1
		2004	10,167	2,747	16,953	26	375.17	101.35	625.60	1
		2005	10,778	2,530	19,081	22	389.01	91.32	688.68	1
		2006	11,294	2,301	21,171	22	403.38	82.20	756.17	1
		2007	11,834	2,098	23,346	21	417.89	74.09	824.44	1

Metropolitan Statistical Area	PWID Population	Year	Number	Min	Max	Rank	Rate	Min	Max	Rank
Syracuse, NY	Total	1992	3,210	2,484	3,824	88	65.53	50.72	78.08	85
		1993	2,634	1,408	3,701	90	53.83	28.78	75.64	91
		1994	2,856	1,915	3,549	91	58.77	39.41	73.04	90
		1995	2,368	1,444	3,401	92	49.03	29.91	70.44	93
		1996	2,242	1,368	3,253	94	46.66	28.47	67.71	95
		1997	2,128	1,142	3,108	94	44.49	23.88	64.98	95
		1998	2,049	1,019	2,976	96	42.85	21.31	62.24	96
		1999	2,174	1,057	2,844	96	45.47	22.11	59.50	96
		2000	2,022	1,229	2,785	97	42.26	25.68	58.20	96
		2001	2,228	1,475	2,733	96	46.38	30.69	56.89	96
		2002	2,115	1,600	2,710	97	43.73	33.08	56.03	96
		2003	2,170	1,638	2,688	97	44.54	33.61	55.16	96
		2004	2,228	1,683	2,660	96	45.47	34.35	54.28	96
		2005	2,285	1,728	2,626	96	46.49	35.14	53.40	96
		2006	2,351	1,775	2,829	97	47.61	35.94	57.29	96
		2007	2,418	1,826	3,119	97	48.79	36.85	62.95	94
	Non-Hispanic White	1992	1,569	1,214	1,869	93	35.23	27.27	41.98	92
		1993	1,340	717	1,883	94	30.22	16.16	42.46	93
		1994	1,493	1,001	1,855	94	33.98	22.79	42.23	93
		1995	1,259	768	1,808	96	28.90	17.63	41.52	94
		1996	1,202	733	1,744	97	27.84	16.99	40.40	95
		1997	1,144	614	1,670	97	26.68	14.32	38.96	95
		1998	1,099	546	1,596	97	25.70	12.78	37.34	97
		1999	1,159	564	1,517	97	27.19	13.22	35.57	96
		2000	1,071	651	1,474	97	25.15	15.28	34.64	97
		2001	1,171	775	1,436	97	27.46	18.17	33.69	95
		2002	1,104	835	1,414	97	25.79	19.51	33.04	97
		2003	1,128	851	1,398	98	26.21	19.78	32.47	99
		2004	1,158	875	1,383	98	26.80	20.25	32.00	100
		2005	1,195	903	1,373	98	27.59	20.86	31.70	100
		2006	1,244	939	1,497	98	28.66	21.63	34.48	99
		2007	1,304	985	1,682	98	30.00	22.65	38.70	99

Metropolitan Statistical Area	PWID Population	Year	Number	Min	Max	Rank	Rate	Min	Max	Rank
Syracuse, NY	Non-Hispanic Black	1992	1,263	978	1,505	67	455.15	352.29	542.29	23
		1993	961	514	1,350	75	339.62	181.59	477.20	36
		1994	972	652	1,208	76	340.05	228.06	422.62	34
		1995	757	462	1,087	83	261.21	159.34	375.25	46
		1996	677	413	982	85	229.19	139.85	332.60	51
		1997	610	328	891	85	204.55	109.77	298.74	53
		1998	561	279	815	87	184.63	91.81	268.20	54
		1999	572	278	748	83	186.39	90.62	243.88	51
		2000	513	312	706	84	164.52	99.97	226.54	55
		2001	547	362	671	81	172.82	114.36	211.99	54
		2002	504	381	646	85	155.93	117.94	199.76	54
		2003	503	380	623	85	152.92	115.39	189.40	52
		2004	504	381	602	84	151.19	114.21	180.49	52
		2005	505	382	580	83	149.85	113.29	172.15	51
		2006	509	384	612	83	148.36	112.00	178.51	51
		2007	514	388	663	84	147.49	111.38	190.27	52
	Hispanic	1992	296	229	353	62	409.27	316.78	487.63	9
		1993	251	134	353	61	332.98	178.04	467.87	12
		1994	282	189	350	61	364.18	244.24	452.61	11
		1995	241	147	347	61	303.84	185.35	436.50	13
		1996	236	144	342	63	283.29	172.86	411.10	13
		1997	230	124	336	63	269.67	144.71	393.84	14
		1998	228	113	331	64	258.04	128.31	374.83	14
		1999	248	120	324	64	273.28	132.88	357.58	14
		2000	235	143	324	65	248.55	151.03	342.24	14
		2001	264	175	324	64	266.64	176.44	327.07	13
		2002	255	193	327	65	248.29	187.80	318.10	14
		2003	265	200	329	65	250.05	188.69	309.70	14
		2004	276	208	329	66	253.98	191.86	303.19	13
		2005	286	216	328	67	257.49	194.67	295.81	13
		2006	297	224	357	66	257.09	194.08	309.35	14
		2007	308	233	397	67	259.31	195.83	334.52	14

Metropolitan Statistical Area	PWID Population	Year	Number	Min	Max	Rank	Rate	Min	Max	Rank
Syracuse, NY	Male	1992	2,451	1,897	2,920	86	101.47	78.54	120.90	77
		1993	1,969	1,053	2,767	87	81.60	43.63	114.65	86
		1994	2,097	1,406	2,606	87	87.50	58.68	108.74	84
		1995	1,713	1,045	2,460	91	71.92	43.87	103.32	88
		1996	1,602	978	2,325	92	67.58	41.24	98.08	89
		1997	1,507	809	2,201	93	63.88	34.28	93.29	91
		1998	1,442	717	2,095	93	61.13	30.40	88.80	91
		1999	1,524	741	1,994	93	64.66	31.44	84.61	92
		2000	1,415	860	1,949	93	60.00	36.46	82.62	93
		2001	1,559	1,032	1,913	94	65.82	43.55	80.74	93
		2002	1,481	1,120	1,898	93	62.11	46.98	79.57	93
		2003	1,522	1,149	1,885	92	63.29	47.75	78.38	92
		2004	1,565	1,182	1,868	92	64.69	48.87	77.22	91
		2005	1,608	1,215	1,847	92	66.14	50.00	75.98	89
		2006	1,655	1,249	1,991	93	67.73	51.13	81.50	87
		2007	1,701	1,285	2,194	93	69.35	52.37	89.46	83
	Female	1992	760	589	906	94	30.63	23.70	36.49	96
		1993	679	363	954	94	27.38	14.64	38.47	96
		1994	787	528	978	95	31.95	21.43	39.71	94
		1995	687	419	987	96	28.06	17.11	40.30	97
		1996	675	412	980	96	27.75	16.93	40.27	96
		1997	658	353	962	96	27.18	14.58	39.69	97
		1998	645	321	937	98	26.62	13.24	38.67	99
		1999	690	336	903	97	28.48	13.85	37.27	98
		2000	643	391	886	98	26.52	16.11	36.51	99
		2001	706	467	866	97	29.00	19.19	35.57	97
		2002	665	503	852	98	27.13	20.52	34.75	99
		2003	675	509	836	99	27.35	20.64	33.87	99
		2004	684	517	817	99	27.58	20.83	32.92	99
		2005	692	524	796	99	27.86	21.06	32.01	99
		2006	704	531	847	99	28.20	21.29	33.94	99
		2007	717	541	925	99	28.63	21.62	36.94	99

Metropolitan Statistical Area	PWID Population	Year	Number	Min	Max	Rank	Rate	Min	Max	Rank
Syracuse, NY	Young (15-29)	1992	740	573	882	91	42.87	33.18	51.08	86
		1993	560	299	787	93	33.25	17.78	46.72	94
		1994	580	389	721	93	35.57	23.85	44.20	94
		1995	475	290	683	94	29.94	18.27	43.02	94
		1996	458	279	664	94	29.55	18.03	42.88	94
		1997	453	243	661	94	29.83	16.01	43.56	94
		1998	463	230	673	94	30.92	15.38	44.92	95
		1999	530	258	693	96	35.90	17.45	46.97	95
		2000	536	326	738	96	36.62	22.25	50.43	96
		2001	645	427	792	96	43.76	28.96	53.68	95
		2002	669	506	857	96	44.60	33.73	57.14	94
		2003	746	563	924	97	48.55	36.64	60.13	91
		2004	825	624	985	97	52.56	39.71	62.75	89
		2005	903	682	1,037	97	56.17	42.47	64.53	92
		2006	977	737	1,175	95	59.57	44.97	71.68	89
		2007	1,040	785	1,341	96	62.33	47.07	80.41	86
	Old (30-64)	1992	2,545	1,970	3,032	87	80.23	62.10	95.59	82
		1993	2,131	1,140	2,994	87	66.40	35.50	93.29	88
		1994	2,335	1,566	2,902	88	72.35	48.52	89.91	87
		1995	1,943	1,185	2,791	92	59.93	36.56	86.09	89
		1996	1,833	1,119	2,661	92	56.31	34.36	81.72	92
		1997	1,724	925	2,518	93	52.82	28.35	77.14	93
		1998	1,635	813	2,375	95	49.80	24.76	72.34	94
		1999	1,698	825	2,221	96	51.38	24.98	67.23	93
		2000	1,536	933	2,114	96	46.24	28.10	63.68	94
		2001	1,634	1,081	2,005	96	49.08	32.48	60.21	92
		2002	1,488	1,125	1,906	97	44.59	33.73	57.13	94
		2003	1,456	1,098	1,803	97	43.63	32.92	54.03	94
		2004	1,417	1,070	1,692	96	42.57	32.15	50.81	94
		2005	1,373	1,038	1,578	96	41.49	31.37	47.67	92
		2006	1,332	1,005	1,603	96	40.37	30.47	48.57	93
		2007	1,292	976	1,667	96	39.32	29.69	50.72	93

Metropolitan Statistical Area	PWID Population	Year	Number	Min	Max	Rank	Rate	Min	Max	Rank
Tacoma, WA	Total	1992	5,725	4,539	6,824	70	140.70	111.56	167.71	38
		1993	7,673	4,956	13,198	53	184.64	119.25	317.58	18
		1994	5,920	5,361	6,936	69	140.23	126.99	164.29	36
		1995	7,478	5,295	11,791	53	173.66	122.96	273.82	18
		1996	7,351	5,104	10,992	53	168.56	117.02	252.04	17
		1997	7,248	4,924	10,193	55	163.52	111.09	229.95	16
		1998	7,223	4,892	9,427	55	159.25	107.87	207.86	15
		1999	6,726	4,856	8,046	63	144.94	104.64	173.38	24
		2000	7,280	4,901	8,725	53	153.96	103.65	184.52	16
		2001	7,472	4,965	9,497	58	154.50	102.65	196.37	18
		2002	7,592	4,854	10,325	50	153.66	98.25	208.99	16
		2003	7,689	4,698	11,136	50	153.57	93.84	222.42	16
		2004	7,772	4,495	11,966	51	153.74	88.92	236.71	16
		2005	7,919	4,314	12,938	51	154.18	84.00	251.92	15
		2006	8,132	4,123	14,072	51	154.83	78.50	267.92	15
		2007	8,284	3,576	15,115	50	155.67	67.20	284.02	15
	Non-Hispanic White	1992	3,742	2,967	4,461	60	111.35	88.29	132.73	34
		1993	5,045	3,258	8,678	43	147.60	95.33	253.87	17
		1994	3,904	3,535	4,573	59	113.18	102.49	132.60	26
		1995	4,933	3,493	7,778	41	141.12	99.92	222.50	17
		1996	4,842	3,362	7,240	42	137.66	95.57	205.83	16
		1997	4,760	3,234	6,694	42	134.03	91.05	188.48	16
		1998	4,726	3,201	6,168	41	130.73	88.55	170.63	15
		1999	4,381	3,163	5,241	48	119.16	86.03	142.54	22
		2000	4,720	3,177	5,657	40	126.81	85.37	151.97	16
		2001	4,823	3,205	6,130	44	127.14	84.47	161.60	18
		2002	4,882	3,121	6,640	40	126.50	80.88	172.05	16
		2003	4,931	3,013	7,142	41	126.44	77.26	183.12	15
		2004	4,979	2,880	7,665	42	126.66	73.26	195.01	15
		2005	5,076	2,765	8,294	42	127.57	69.50	208.44	14
		2006	5,228	2,651	9,046	41	129.14	65.48	223.47	14
		2007	5,354	2,311	9,768	41	131.09	56.59	239.16	15

Metropolitan Statistical Area	PWID Population	Year	Number	Min	Max	Rank	Rate	Min	Max	Rank
Tacoma, WA	Non-Hispanic Black	1992	1,000	793	1,192	79	349.56	277.16	416.66	39
		1993	1,161	750	1,997	68	399.72	258.16	687.52	27
		1994	798	722	935	84	265.34	240.29	310.87	51
		1995	921	652	1,453	73	296.47	209.91	467.45	39
		1996	848	589	1,268	73	266.03	184.69	397.78	42
		1997	800	543	1,125	74	243.05	165.12	341.80	43
		1998	777	526	1,014	73	231.34	156.69	301.95	40
		1999	716	517	856	76	206.93	149.40	247.53	46
		2000	776	522	930	69	216.42	145.69	259.36	36
		2001	804	535	1,023	69	220.06	146.21	279.70	27
		2002	830	531	1,129	67	220.13	140.75	299.41	27
		2003	855	522	1,238	67	224.42	137.13	325.02	25
		2004	877	507	1,350	67	228.92	132.40	352.45	21
		2005	904	492	1,477	67	232.25	126.53	379.46	20
		2006	933	473	1,614	66	232.30	117.78	401.97	20
		2007	948	409	1,729	68	232.90	100.54	424.92	24
	Hispanic	1992	322	255	384	58	213.29	169.12	254.24	28
		1993	412	266	708	55	256.81	165.86	441.72	22
		1994	307	278	359	60	179.38	162.44	210.15	28
		1995	378	267	595	57	207.28	146.76	326.82	26
		1996	365	253	545	57	188.02	130.53	281.13	28
		1997	355	241	500	58	171.91	116.79	241.75	31
		1998	351	238	459	59	159.42	107.98	208.08	29
		1999	326	235	390	61	138.83	100.23	166.07	31
		2000	351	237	421	61	142.42	95.88	170.68	26
		2001	360	239	457	61	136.75	90.86	173.81	27
		2002	364	233	495	62	130.18	83.23	177.05	29
		2003	366	224	530	62	125.76	76.84	182.13	31
		2004	366	212	564	62	121.92	70.52	187.71	31
		2005	368	200	601	62	116.49	63.46	190.32	31
		2006	371	188	642	62	109.80	55.67	190.00	32
		2007	369	159	674	64	104.56	45.13	190.76	36

Metropolitan Statistical Area	PWID Population	Year	Number	Min	Max	Rank	Rate	Min	Max	Rank
Tacoma, WA	Male	1992	3,336	2,645	3,977	78	161.89	128.36	192.97	44
		1993	4,522	2,921	7,778	58	215.33	139.07	370.37	27
		1994	3,485	3,156	4,083	78	163.00	147.61	190.97	41
		1995	4,353	3,082	6,863	63	200.06	141.65	315.44	28
		1996	4,195	2,912	6,272	63	190.59	132.32	284.98	31
		1997	4,027	2,735	5,662	63	180.06	122.32	253.21	33
		1998	3,886	2,632	5,072	65	170.09	115.21	222.00	35
		1999	3,494	2,522	4,179	76	149.31	107.80	178.60	38
		2000	3,647	2,455	4,370	68	153.12	103.08	183.51	38
		2001	3,616	2,403	4,596	74	148.39	98.59	188.60	36
		2002	3,567	2,280	4,851	71	143.51	91.76	195.19	38
		2003	3,535	2,160	5,120	71	141.06	86.20	204.30	39
		2004	3,536	2,045	5,445	71	139.87	80.90	215.35	39
		2005	3,618	1,971	5,911	68	140.56	76.58	229.66	36
		2006	3,794	1,924	6,565	68	143.73	72.88	248.72	33
		2007	4,022	1,736	7,338	66	150.46	64.95	274.51	31
	Female	1992	2,349	1,862	2,800	64	116.97	92.74	139.42	22
		1993	3,126	2,019	5,376	43	152.06	98.21	261.55	11
		1994	2,435	2,206	2,853	62	116.89	105.85	136.95	21
		1995	3,149	2,229	4,965	44	147.81	104.65	233.06	11
		1996	3,200	2,221	4,784	45	148.11	102.82	221.46	7
		1997	3,281	2,229	4,613	43	149.37	101.48	210.05	7
		1998	3,409	2,309	4,449	41	151.45	102.58	197.68	7
		1999	3,308	2,389	3,957	45	143.78	103.80	171.99	7
		2000	3,720	2,504	4,458	39	158.49	106.69	189.94	6
		2001	3,944	2,620	5,013	41	164.37	109.21	208.92	6
		2002	4,108	2,627	5,588	38	167.32	106.99	227.58	5
		2003	4,227	2,583	6,122	35	169.03	103.29	244.81	4
		2004	4,292	2,482	6,608	36	169.86	98.24	261.52	4
		2005	4,334	2,361	7,081	36	169.16	92.16	276.38	4
		2006	4,339	2,200	7,509	36	166.11	84.22	287.43	4
		2007	4,223	1,823	7,704	36	159.42	68.82	290.86	4

Metropolitan Statistical Area	PWID Population	Year	Number	Min	Max	Rank	Rate	Min	Max	Rank
Tacoma, WA	Young (15-29)	1992	1,353	1,073	1,613	75	96.80	76.75	115.38	42
		1993	1,860	1,202	3,200	53	134.19	86.67	230.81	23
		1994	1,466	1,327	1,717	73	105.63	95.66	123.76	36
		1995	1,881	1,332	2,966	54	134.86	95.48	212.63	20
		1996	1,871	1,299	2,798	55	133.61	92.76	199.78	18
		1997	1,859	1,263	2,614	57	131.25	89.17	184.57	21
		1998	1,860	1,260	2,427	61	128.91	87.31	168.25	26
		1999	1,732	1,251	2,072	67	117.71	84.99	140.81	33
		2000	1,869	1,258	2,240	62	125.42	84.43	150.30	28
		2001	1,906	1,266	2,423	68	125.29	83.24	159.25	32
		2002	1,919	1,227	2,609	68	122.98	78.64	167.27	33
		2003	1,920	1,173	2,781	70	121.63	74.32	176.15	33
		2004	1,913	1,106	2,945	74	120.13	69.48	184.95	33
		2005	1,917	1,044	3,132	74	118.33	64.46	193.33	32
		2006	1,933	980	3,345	75	115.75	58.69	200.29	37
		2007	1,931	833	3,522	75	114.44	49.40	208.79	37
	Old (30-64)	1992	4,456	3,533	5,312	68	166.84	132.28	198.86	34
		1993	5,871	3,792	10,099	52	212.01	136.93	364.67	17
		1994	4,483	4,060	5,252	67	158.17	143.24	185.31	36
		1995	5,638	3,992	8,889	53	193.66	137.12	305.35	18
		1996	5,542	3,848	8,287	54	187.19	129.96	279.89	16
		1997	5,480	3,723	7,706	53	181.68	123.43	255.49	16
		1998	5,481	3,713	7,154	54	177.24	120.05	231.33	16
		1999	5,121	3,697	6,125	59	161.58	116.66	193.28	22
		2000	5,547	3,734	6,648	52	171.30	115.32	205.30	15
		2001	5,678	3,772	7,216	53	171.26	113.79	217.68	12
		2002	5,722	3,658	7,782	46	169.25	108.22	230.20	9
		2003	5,707	3,487	8,265	46	166.47	101.72	241.09	9
		2004	5,625	3,253	8,660	47	162.42	93.94	250.07	9
		2005	5,510	3,002	9,003	48	156.73	85.39	256.07	11
		2006	5,333	2,704	9,228	48	148.87	75.48	257.60	11
		2007	4,973	2,147	9,073	49	136.81	59.06	249.61	13

Metropolitan Statistical Area	PWID Population	Year	Number	Min	Max	Rank	Rate	Min	Max	Rank
Tampa--St. Petersburg--Clearwater, FL	Total	1992	15,708	12,157	17,834	31	121.06	93.69	137.44	44
		1993	12,805	4,647	17,512	33	97.71	35.46	133.62	52
		1994	15,416	12,535	17,240	31	116.07	94.39	129.81	45
		1995	12,850	5,208	17,054	31	95.01	38.50	126.09	51
		1996	12,929	5,461	16,842	32	94.02	39.71	122.48	52
		1997	13,251	5,741	16,756	31	94.08	40.76	118.97	51
		1998	13,871	6,030	16,713	30	96.17	41.80	115.87	50
		1999	17,158	16,524	17,568	20	116.55	112.24	119.34	40
		2000	15,202	6,548	18,931	25	101.02	43.51	125.80	47
		2001	18,890	16,294	20,547	20	122.77	105.90	133.54	34
		2002	16,187	6,230	22,921	23	102.86	39.59	145.66	43
		2003	16,348	6,050	25,363	20	101.69	37.63	157.77	44
		2004	16,564	5,890	28,353	21	100.19	35.63	171.50	45
		2005	16,770	5,698	31,506	21	98.59	33.50	185.23	44
		2006	16,869	5,457	34,675	20	96.97	31.37	199.34	47
		2007	16,763	5,125	37,540	20	95.31	29.14	213.45	46
	Non-Hispanic White	1992	10,552	8,166	11,980	20	100.33	77.65	113.90	38
		1993	8,649	3,139	11,829	24	82.13	29.81	112.33	44
		1994	10,462	8,507	11,701	18	98.77	80.31	110.46	38
		1995	8,758	3,549	11,623	24	81.90	33.19	108.69	51
		1996	8,848	3,737	11,525	24	82.10	34.68	106.95	49
		1997	9,106	3,945	11,515	23	83.20	36.04	105.20	47
		1998	9,576	4,163	11,538	21	86.08	37.42	103.72	43
		1999	11,908	11,468	12,193	14	105.84	101.93	108.38	29
		2000	10,615	4,572	13,219	18	93.15	40.12	116.00	38
		2001	13,284	11,459	14,449	13	115.09	99.28	125.18	24
		2002	11,478	4,418	16,254	16	98.20	37.80	139.06	32
		2003	11,704	4,331	18,159	15	98.90	36.60	153.44	32
		2004	11,990	4,264	20,524	15	99.44	35.36	170.22	34
		2005	12,289	4,176	23,088	14	100.08	34.01	188.03	34
		2006	12,531	4,054	25,759	14	100.96	32.66	207.53	34
		2007	12,638	3,864	28,303	14	101.87	31.14	228.13	34

Metropolitan Statistical Area	PWID Population	Year	Number	Min	Max	Rank	Rate	Min	Max	Rank
Tampa--St. Petersburg--Clearwater, FL	Non-Hispanic Black	1992	3,139	2,430	3,564	33	261.21	202.15	296.56	58
		1993	2,470	896	3,378	40	199.84	72.53	273.30	65
		1994	2,875	2,338	3,215	35	225.82	183.63	252.56	63
		1995	2,315	938	3,073	40	175.92	71.29	233.47	69
		1996	2,245	948	2,924	39	165.44	69.88	215.51	71
		1997	2,206	956	2,790	38	156.21	67.67	197.52	73
		1998	2,200	956	2,651	36	150.09	65.25	180.85	69
		1999	2,569	2,474	2,631	34	169.23	162.98	173.28	65
		2000	2,125	915	2,646	36	135.78	58.48	169.08	69
		2001	2,431	2,097	2,645	34	149.88	129.29	163.03	66
		2002	1,888	727	2,673	40	112.35	43.25	159.10	69
		2003	1,696	628	2,631	44	97.57	36.11	151.39	76
		2004	1,497	533	2,563	52	82.72	29.42	141.60	85
		2005	1,292	439	2,427	59	68.53	23.29	128.74	88
		2006	1,082	350	2,224	65	55.20	17.86	113.46	93
		2007	875	267	1,959	71	43.39	13.26	97.16	98
	Hispanic	1992	908	703	1,031	39	89.17	69.01	101.24	64
		1993	718	261	982	46	66.56	24.16	91.02	67
		1994	859	698	960	42	75.58	61.46	84.52	68
		1995	725	294	962	48	59.87	24.26	79.46	72
		1996	751	317	978	47	58.18	24.58	75.79	71
		1997	802	347	1,014	46	58.46	25.32	73.92	67
		1998	884	384	1,065	45	60.77	26.42	73.22	64
		1999	1,157	1,114	1,185	41	74.79	72.03	76.58	57
		2000	1,089	469	1,356	43	65.83	28.35	81.98	59
		2001	1,436	1,239	1,562	40	81.39	70.21	88.53	53
		2002	1,303	502	1,845	42	69.05	26.58	97.78	57
		2003	1,386	513	2,150	40	69.36	25.67	107.61	56
		2004	1,467	522	2,512	40	68.79	24.46	117.76	56
		2005	1,536	522	2,887	40	67.32	22.88	126.49	56
		2006	1,579	511	3,246	40	64.87	20.99	133.35	57
		2007	1,582	483	3,542	41	61.89	18.92	138.60	58

Metropolitan Statistical Area	PWID Population	Year	Number	Min	Max	Rank	Rate	Min	Max	Rank
Tampa--St. Petersburg--Clearwater, FL	Male	1992	10,047	7,776	11,407	33	158.50	122.67	179.95	45
		1993	8,147	2,957	11,142	35	127.12	46.14	173.85	53
		1994	9,733	7,915	10,885	32	149.80	121.81	167.54	44
		1995	8,033	3,255	10,660	34	121.36	49.18	161.06	53
		1996	7,986	3,373	10,403	33	118.53	50.07	154.40	53
		1997	8,074	3,498	10,210	33	116.86	50.63	147.77	52
		1998	8,327	3,620	10,033	32	117.67	51.15	141.78	52
		1999	10,137	9,763	10,380	26	140.25	135.07	143.61	40
		2000	8,835	3,806	11,003	29	119.54	51.49	148.87	52
		2001	10,798	9,314	11,745	21	142.85	123.22	155.38	38
		2002	9,103	3,504	12,891	28	117.72	45.31	166.71	49
		2003	9,053	3,350	14,045	27	114.53	42.38	177.69	54
		2004	9,044	3,216	15,481	27	111.11	39.51	190.19	55
		2005	9,045	3,074	16,993	27	107.91	36.67	202.74	56
		2006	9,011	2,915	18,522	27	105.03	33.98	215.90	56
		2007	8,894	2,719	19,919	26	102.37	31.29	229.24	56
	Female	1992	5,680	4,396	6,448	29	85.58	66.23	97.17	39
		1993	4,634	1,682	6,337	32	69.20	25.12	94.64	47
		1994	5,616	4,567	6,281	26	82.79	67.32	92.59	41
		1995	4,737	1,920	6,287	29	68.59	27.80	91.03	53
		1996	4,843	2,046	6,309	28	69.05	29.17	89.95	53
		1997	5,061	2,192	6,399	27	70.52	30.55	89.18	51
		1998	5,414	2,353	6,523	26	73.68	32.03	88.78	49
		1999	6,856	6,603	7,020	20	91.49	88.11	93.68	32
		2000	6,224	2,681	7,751	22	81.28	35.01	101.22	40
		2001	7,925	6,836	8,620	18	101.25	87.34	110.13	25
		2002	6,955	2,677	9,849	20	86.90	33.45	123.06	31
		2003	7,186	2,659	11,149	20	87.94	32.54	136.43	28
		2004	7,434	2,644	12,726	18	88.57	31.50	151.62	26
		2005	7,667	2,605	14,405	16	88.87	30.20	166.96	24
		2006	7,834	2,534	16,103	15	88.86	28.75	182.65	23
		2007	7,880	2,409	17,648	15	88.56	27.07	198.33	24

Metropolitan Statistical Area	PWID Population	Year	Number	Min	Max	Rank	Rate	Min	Max	Rank
Tampa--St. Petersburg--Clearwater, FL	Young (15-29)	1992	3,728	2,885	4,232	35	94.42	73.07	107.20	46
		1993	2,946	1,069	4,028	40	75.63	27.45	103.43	53
		1994	3,543	2,881	3,963	31	92.08	74.88	102.98	43
		1995	3,029	1,227	4,020	33	78.81	31.94	104.59	54
		1996	3,191	1,348	4,156	32	82.68	34.92	107.70	53
		1997	3,478	1,507	4,398	31	88.64	38.40	112.08	47
		1998	3,913	1,701	4,715	26	97.79	42.51	117.83	44
		1999	5,230	5,036	5,355	18	128.82	124.06	131.91	28
		2000	5,009	2,157	6,238	21	121.64	52.39	151.48	31
		2001	6,703	5,782	7,291	15	160.12	138.12	174.16	17
		2002	6,140	2,363	8,695	15	143.81	55.36	203.65	22
		2003	6,559	2,427	10,177	16	149.61	55.36	232.12	22
		2004	6,936	2,467	11,874	15	152.36	54.18	260.81	22
		2005	7,215	2,452	13,556	14	153.01	51.99	287.46	22
		2006	7,322	2,369	15,051	15	150.05	48.54	308.44	22
		2007	7,187	2,197	16,094	15	144.72	44.24	324.09	24
	Old (30-64)	1992	12,004	9,290	13,629	31	132.98	102.92	150.98	45
		1993	9,860	3,579	13,485	32	107.06	38.86	146.41	52
		1994	11,857	9,642	13,260	29	125.69	102.21	140.57	44
		1995	9,798	3,971	13,003	29	101.20	41.01	134.30	54
		1996	9,706	4,100	12,644	29	98.12	41.45	127.82	58
		1997	9,732	4,216	12,305	29	95.78	41.49	121.11	58
		1998	9,904	4,306	11,934	29	95.03	41.31	114.50	58
		1999	11,848	11,410	12,131	26	111.12	107.02	113.78	48
		2000	10,108	4,354	12,587	28	92.47	39.83	115.16	56
		2001	12,063	10,405	13,120	24	107.70	92.91	117.15	47
		2002	9,926	3,821	14,056	27	86.56	33.32	122.58	56
		2003	9,656	3,573	14,981	26	82.59	30.56	128.14	55
		2004	9,483	3,373	16,233	27	79.16	28.15	135.51	54
		2005	9,400	3,194	17,660	27	76.46	25.98	143.65	55
		2006	9,383	3,035	19,287	27	74.97	24.25	154.10	55
		2007	9,403	2,875	21,057	27	74.50	22.78	166.84	55

Metropolitan Statistical Area	PWID Population	Year	Number	Min	Max	Rank	Rate	Min	Max	Rank
Toledo, OH	Total	1992	2,670	2,099	3,015	93	66.35	52.15	74.92	84
		1993	2,267	1,189	2,940	95	56.39	29.57	73.14	86
		1994	2,582	2,124	2,991	94	64.24	52.84	74.42	83
		1995	2,203	1,180	3,051	95	54.67	29.27	75.70	88
		1996	2,176	1,176	3,121	95	53.89	29.12	77.30	88
		1997	2,113	1,171	3,196	95	52.15	28.91	78.90	89
		1998	2,055	1,167	3,295	95	50.57	28.71	81.09	90
		1999	2,303	1,649	3,389	94	56.60	40.53	83.29	85
		2000	2,020	1,207	3,496	98	49.56	29.61	85.75	92
		2001	2,324	1,635	3,612	95	56.75	39.93	88.21	86
		2002	2,133	1,284	3,749	96	51.94	31.25	91.29	89
		2003	2,256	1,321	3,886	95	54.79	32.08	94.38	84
		2004	2,415	1,333	4,020	95	58.54	32.32	97.46	79
		2005	2,568	1,243	4,152	95	62.18	30.11	100.55	75
		2006	2,721	1,156	4,272	93	66.02	28.04	103.63	72
		2007	2,865	1,071	4,658	93	69.47	25.98	112.96	70
	Non-Hispanic White	1992	1,313	1,032	1,483	96	38.74	30.45	43.74	87
		1993	1,165	611	1,511	97	34.51	18.10	44.77	87
		1994	1,371	1,128	1,588	96	40.77	33.54	47.24	84
		1995	1,200	643	1,662	97	35.71	19.12	49.45	85
		1996	1,209	653	1,734	96	36.04	19.47	51.69	86
		1997	1,192	661	1,803	96	35.54	19.70	53.76	85
		1998	1,175	667	1,883	95	35.01	19.87	56.14	86
		1999	1,332	954	1,960	95	39.79	28.49	58.55	82
		2000	1,182	706	2,045	96	35.40	21.15	61.24	85
		2001	1,377	969	2,140	96	41.17	28.97	63.99	83
		2002	1,282	771	2,253	96	38.35	23.07	67.40	85
		2003	1,379	807	2,376	96	41.26	24.16	71.09	84
		2004	1,507	832	2,509	96	45.12	24.91	75.13	82
		2005	1,642	795	2,654	95	49.27	23.86	79.67	78
		2006	1,790	760	2,809	92	53.99	22.93	84.75	73
		2007	1,945	727	3,162	88	58.85	22.01	95.69	67

Metropolitan Statistical Area	PWID Population	Year	Number	Min	Max	Rank	Rate	Min	Max	Rank
Toledo, OH	Non-Hispanic Black	1992	1,081	850	1,220	75	242.77	190.81	274.11	63
		1993	869	456	1,127	82	192.51	100.95	249.71	69
		1994	943	776	1,093	79	206.21	169.63	238.92	69
		1995	771	413	1,068	82	165.91	88.83	229.74	74
		1996	733	396	1,052	82	155.26	83.88	222.69	75
		1997	687	381	1,039	81	142.94	79.24	216.25	76
		1998	646	366	1,035	81	132.56	75.25	212.56	76
		1999	699	500	1,028	78	141.30	101.18	207.92	73
		2000	591	353	1,022	81	117.40	70.14	203.11	75
		2001	653	459	1,015	78	127.50	89.72	198.18	74
		2002	572	344	1,006	78	110.68	66.60	194.53	72
		2003	574	336	989	77	109.89	64.34	189.30	69
		2004	578	319	962	78	109.65	60.53	182.56	69
		2005	572	277	925	78	107.11	51.87	173.18	69
		2006	558	237	876	78	103.18	43.83	161.97	71
		2007	535	200	870	82	97.97	36.64	159.30	74
	Hispanic	1992	186	146	210	69	142.71	112.16	161.13	47
		1993	157	82	204	69	116.71	61.20	151.39	48
		1994	177	145	205	69	127.92	105.23	148.20	43
		1995	148	79	205	71	103.48	55.41	143.30	49
		1996	142	77	204	71	96.23	51.99	138.02	49
		1997	134	74	202	73	88.17	48.88	133.39	51
		1998	125	71	201	74	80.55	45.73	129.17	52
		1999	135	97	199	74	84.88	60.78	124.90	54
		2000	114	68	198	75	69.24	41.37	119.79	57
		2001	127	89	197	75	74.84	52.66	116.32	58
		2002	113	68	198	78	64.54	38.84	113.44	59
		2003	116	68	200	79	64.67	37.86	111.40	59
		2004	122	68	204	79	66.35	36.63	110.48	57
		2005	129	63	209	79	68.87	33.35	111.36	55
		2006	138	59	217	81	72.35	30.73	113.57	53
		2007	149	56	242	79	76.02	28.43	123.61	52

Metropolitan Statistical Area	PWID Population	Year	Number	Min	Max	Rank	Rate	Min	Max	Rank
Toledo, OH	Male	1992	1,717	1,350	1,939	95	87.65	68.89	98.96	86
		1993	1,399	734	1,815	96	71.40	37.44	92.61	91
		1994	1,542	1,268	1,786	96	78.65	64.70	91.12	87
		1995	1,285	688	1,779	97	65.29	34.96	90.41	93
		1996	1,250	675	1,793	97	63.35	34.22	90.86	93
		1997	1,204	668	1,822	97	60.82	33.72	92.02	93
		1998	1,171	665	1,878	98	58.95	33.46	94.52	95
		1999	1,319	944	1,940	97	66.23	47.43	97.45	88
		2000	1,167	697	2,019	99	58.50	34.95	101.20	94
		2001	1,358	956	2,111	97	67.66	47.61	105.17	88
		2002	1,263	760	2,219	99	62.67	37.71	110.16	91
		2003	1,352	792	2,329	97	66.89	39.16	115.23	87
		2004	1,464	808	2,437	95	72.22	39.87	120.25	79
		2005	1,571	761	2,540	93	77.32	37.45	125.03	76
		2006	1,674	711	2,627	92	82.41	35.00	129.36	71
		2007	1,763	659	2,867	92	86.72	32.43	141.01	67
	Female	1992	991	779	1,119	89	48.02	37.74	54.21	78
		1993	899	471	1,166	89	43.62	22.88	56.59	82
		1994	1,074	883	1,244	88	52.17	42.92	60.45	76
		1995	947	507	1,312	89	45.94	24.60	63.61	80
		1996	955	516	1,370	88	46.27	25.00	66.36	79
		1997	937	520	1,418	91	45.26	25.09	68.48	79
		1998	914	519	1,465	92	43.99	24.97	70.53	78
		1999	1,019	730	1,500	89	49.04	35.12	72.17	80
		2000	885	529	1,531	96	42.53	25.41	73.57	82
		2001	1,004	706	1,560	94	48.07	33.83	74.72	81
		2002	906	545	1,593	96	43.30	26.06	76.11	83
		2003	941	551	1,621	96	44.91	26.29	77.36	81
		2004	990	547	1,649	95	47.20	26.06	78.59	77
		2005	1,037	502	1,677	95	49.44	23.94	79.95	73
		2006	1,087	462	1,706	94	51.98	22.08	81.60	67
		2007	1,138	426	1,850	93	54.43	20.36	88.51	63

Metropolitan Statistical Area	PWID Population	Year	Number	Min	Max	Rank	Rate	Min	Max	Rank
Toledo, OH	Young (15-29)	1992	598	470	675	95	40.92	32.16	46.20	91
		1993	489	257	635	96	33.97	17.81	44.06	92
		1994	540	444	625	94	38.04	31.30	44.08	89
		1995	449	240	622	95	31.82	17.04	44.06	92
		1996	436	236	625	95	31.03	16.76	44.51	92
		1997	420	233	636	95	29.97	16.62	45.34	93
		1998	410	233	658	98	29.24	16.60	46.88	98
		1999	467	334	687	99	33.53	24.01	49.34	97
		2000	422	252	729	100	30.49	18.21	52.75	100
		2001	507	357	788	100	36.74	25.85	57.10	99
		2002	494	297	868	100	35.79	21.54	62.91	100
		2003	563	330	970	100	40.78	23.88	70.26	100
		2004	660	365	1,099	100	47.67	26.32	79.37	96
		2005	781	378	1,263	100	56.20	27.22	90.87	91
		2006	933	396	1,465	97	67.40	28.63	105.80	79
		2007	1,118	418	1,818	91	80.61	30.15	131.06	62
	Old (30-64)	1992	2,128	1,673	2,403	92	83.03	65.26	93.75	78
		1993	1,821	955	2,362	93	70.57	37.01	91.54	83
		1994	2,086	1,716	2,417	93	80.23	66.00	92.95	81
		1995	1,788	957	2,476	93	68.29	36.57	94.57	82
		1996	1,771	957	2,540	93	67.27	36.35	96.49	81
		1997	1,720	954	2,603	94	64.93	36.00	98.24	81
		1998	1,671	948	2,679	94	62.77	35.63	100.65	81
		1999	1,864	1,335	2,743	93	69.62	49.85	102.44	78
		2000	1,623	970	2,808	94	60.26	36.00	104.25	79
		2001	1,847	1,300	2,871	93	68.02	47.87	105.73	77
		2002	1,671	1,006	2,937	92	61.27	36.87	107.70	75
		2003	1,734	1,015	2,988	91	63.37	37.10	109.17	68
		2004	1,812	1,000	3,017	91	66.15	36.52	110.14	65
		2005	1,869	905	3,022	89	68.23	33.04	110.32	61
		2006	1,905	809	2,991	89	69.59	29.56	109.24	60
		2007	1,908	714	3,103	89	69.73	26.08	113.39	57

Metropolitan Statistical Area	PWID Population	Year	Number	Min	Max	Rank	Rate	Min	Max	Rank
Tucson, AZ	Total	1992	10,572	8,550	12,176	42	234.55	189.68	270.13	11
		1993	10,798	9,213	12,036	40	232.52	198.40	259.18	6
		1994	11,006	10,042	12,037	42	227.05	207.15	248.31	8
		1995	11,272	10,819	11,881	36	225.28	216.23	237.44	7
		1996	11,291	10,527	11,569	34	221.29	206.31	226.73	7
		1997	11,359	10,258	12,320	33	217.22	196.16	235.59	6
		1998	11,321	9,766	13,056	35	213.23	183.95	245.91	6
		1999	11,276	9,287	13,880	36	208.52	171.74	256.69	7
		2000	11,424	8,817	14,842	35	206.24	159.17	267.93	7
		2001	11,523	8,294	15,823	36	203.67	146.60	279.67	6
		2002	11,804	7,784	16,927	35	203.57	134.24	291.92	7
		2003	12,123	7,208	18,020	35	205.00	121.89	304.71	7
		2004	12,673	6,571	19,264	33	209.23	108.48	318.07	7
		2005	13,275	5,910	20,636	31	213.57	95.08	332.00	7
		2006	13,962	5,214	22,130	29	218.57	81.63	346.44	7
		2007	14,575	4,443	23,523	27	223.64	68.17	360.93	7
	Non-Hispanic White	1992	5,225	4,226	6,018	46	172.48	139.49	198.65	14
		1993	5,073	4,328	5,654	42	164.24	140.14	183.07	14
		1994	4,951	4,517	5,414	47	154.81	141.25	169.31	15
		1995	4,900	4,703	5,165	42	149.78	143.76	157.87	13
		1996	4,797	4,472	4,915	44	145.23	135.40	148.81	13
		1997	4,777	4,314	5,181	41	142.21	128.42	154.23	13
		1998	4,781	4,125	5,514	39	141.39	121.97	163.06	11
		1999	4,856	3,999	5,978	43	142.10	117.03	174.92	13
		2000	5,096	3,933	6,621	37	147.01	113.46	190.99	9
		2001	5,406	3,891	7,424	38	154.07	110.89	211.56	9
		2002	5,905	3,894	8,469	35	165.68	109.26	237.59	7
		2003	6,541	3,889	9,723	29	181.55	107.95	269.87	6
		2004	7,433	3,854	11,299	27	202.86	105.18	308.37	5
		2005	8,497	3,783	13,210	24	227.66	101.36	353.92	4
		2006	9,750	3,641	15,453	20	256.55	95.81	406.63	2
		2007	11,052	3,369	17,836	18	287.87	87.75	464.59	1

Metropolitan Statistical Area	PWID Population	Year	Number	Min	Max	Rank	Rate	Min	Max	Rank
Tucson, AZ	Non-Hispanic Black	1992	1,055	853	1,215	76	772.92	625.06	890.16	4
		1993	983	839	1,096	74	701.63	598.65	782.05	7
		1994	907	827	992	80	609.57	556.16	666.66	5
		1995	836	803	881	76	538.09	516.47	567.15	9
		1996	753	702	772	80	467.75	436.10	479.26	11
		1997	683	617	741	82	411.19	371.32	445.96	11
		1998	619	534	714	83	365.51	315.31	421.53	11
		1999	568	467	699	84	328.13	270.25	403.92	15
		2000	538	415	699	83	301.76	232.88	392.02	13
		2001	519	373	712	83	282.82	203.57	388.36	17
		2002	521	344	747	84	275.93	181.96	395.69	14
		2003	541	322	804	81	280.74	166.92	417.29	15
		2004	592	307	899	77	295.92	153.43	449.84	14
		2005	674	300	1,048	75	327.59	145.85	509.27	12
		2006	806	301	1,277	73	372.02	138.93	589.66	11
		2007	1,004	306	1,620	66	448.04	136.57	723.09	8
	Hispanic	1992	2,936	2,375	3,382	27	259.96	210.23	299.40	23
		1993	3,079	2,627	3,431	27	258.49	220.55	288.12	21
		1994	3,210	2,929	3,511	26	254.10	231.83	277.90	21
		1995	3,348	3,213	3,528	22	252.42	242.28	266.05	17
		1996	3,395	3,165	3,479	22	246.40	229.72	252.46	16
		1997	3,435	3,102	3,726	22	239.89	216.63	260.17	15
		1998	3,416	2,947	3,939	23	231.16	199.41	266.59	16
		1999	3,364	2,771	4,142	18	220.51	181.61	271.44	16
		2000	3,337	2,575	4,335	23	209.66	161.80	272.37	17
		2001	3,256	2,344	4,471	22	197.06	141.84	270.59	18
		2002	3,185	2,100	4,567	23	184.99	121.99	265.28	18
		2003	3,077	1,829	4,573	24	172.32	102.46	256.13	18
		2004	2,974	1,542	4,521	24	160.83	83.39	244.49	19
		2005	2,827	1,258	4,394	25	147.05	65.47	228.59	23
		2006	2,641	986	4,186	28	131.74	49.20	208.81	25
		2007	2,394	730	3,863	28	115.21	35.12	185.93	28

Metropolitan Statistical Area	PWID Population	Year	Number	Min	Max	Rank	Rate	Min	Max	Rank
Tucson, AZ	Male	1992	7,856	6,353	9,048	38	351.27	284.07	404.55	4
		1993	7,660	6,536	8,538	37	332.48	283.68	370.59	4
		1994	7,466	6,812	8,165	40	310.56	283.35	339.65	6
		1995	7,336	7,042	7,733	37	295.77	283.89	311.75	4
		1996	7,086	6,606	7,260	37	280.36	261.39	287.26	7
		1997	6,917	6,246	7,502	36	267.18	241.27	289.77	8
		1998	6,738	5,812	7,770	36	256.68	221.43	296.02	8
		1999	6,611	5,445	8,138	40	247.47	203.81	304.63	11
		2000	6,654	5,135	8,644	37	243.14	187.65	315.87	8
		2001	6,722	4,838	9,230	39	240.30	172.96	329.97	12
		2002	6,953	4,585	9,971	36	242.52	159.92	347.78	8
		2003	7,265	4,320	10,799	36	248.84	147.96	369.88	8
		2004	7,778	4,033	11,824	35	259.85	134.73	395.01	7
		2005	8,393	3,737	13,048	32	273.13	121.60	424.59	7
		2006	9,136	3,412	14,480	25	288.94	107.91	457.97	7
		2007	9,904	3,019	15,984	24	306.67	93.48	494.94	6
	Female	1992	2,928	2,368	3,372	49	128.93	104.26	148.48	19
		1993	3,300	2,815	3,678	41	141.02	120.32	157.18	14
		1994	3,661	3,341	4,004	41	149.84	136.71	163.88	9
		1995	4,030	3,868	4,247	34	159.70	153.28	168.32	6
		1996	4,283	3,993	4,388	33	166.32	155.07	170.42	6
		1997	4,517	4,079	4,899	33	171.05	154.47	185.52	5
		1998	4,663	4,023	5,378	32	173.73	149.87	200.35	5
		1999	4,756	3,917	5,855	35	173.85	143.18	214.00	5
		2000	4,878	3,765	6,337	31	174.05	134.32	226.11	5
		2001	4,923	3,543	6,760	33	172.11	123.88	236.33	5
		2002	4,984	3,286	7,147	27	170.01	112.11	243.80	4
		2003	4,992	2,968	7,421	28	166.74	99.14	247.84	5
		2004	5,018	2,602	7,627	27	163.79	84.92	248.99	5
		2005	4,973	2,214	7,730	28	158.23	70.44	245.98	5
		2006	4,859	1,815	7,702	31	150.63	56.25	238.75	6
		2007	4,617	1,407	7,451	34	140.42	42.80	226.63	6

Metropolitan Statistical Area	PWID Population	Year	Number	Min	Max	Rank	Rate	Min	Max	Rank
Tucson, AZ	Young (15-29)	1992	2,657	2,149	3,060	43	165.56	133.89	190.67	14
		1993	2,554	2,179	2,847	41	157.20	134.13	175.22	14
		1994	2,491	2,273	2,724	41	149.51	136.41	163.51	16
		1995	2,483	2,383	2,617	42	145.74	139.89	153.61	14
		1996	2,460	2,294	2,521	42	142.38	132.74	145.88	16
		1997	2,487	2,246	2,697	42	141.09	127.41	153.02	15
		1998	2,528	2,180	2,915	41	141.48	122.05	163.16	15
		1999	2,603	2,144	3,204	46	143.62	118.28	176.79	17
		2000	2,762	2,132	3,588	42	149.42	115.31	194.11	18
		2001	2,951	2,124	4,052	44	157.16	113.12	215.81	18
		2002	3,234	2,133	4,638	38	168.61	111.19	241.80	16
		2003	3,583	2,131	5,326	35	183.73	109.24	273.10	15
		2004	4,068	2,109	6,183	32	203.72	105.62	309.68	12
		2005	4,648	2,069	7,225	26	227.93	101.48	354.34	7
		2006	5,346	1,996	8,473	21	255.56	95.44	405.06	5
		2007	6,106	1,861	9,854	18	286.38	87.29	462.19	2
	Old (30-64)	1992	8,043	6,504	9,263	40	277.08	224.07	319.11	5
		1993	8,326	7,104	9,281	38	275.76	235.29	307.37	6
		1994	8,568	7,818	9,371	38	269.34	245.74	294.57	5
		1995	8,829	8,474	9,306	35	267.54	256.79	281.99	5
		1996	8,866	8,266	9,084	34	262.74	244.96	269.21	4
		1997	8,911	8,047	9,665	34	257.04	232.12	278.78	5
		1998	8,839	7,625	10,194	32	250.92	216.46	289.37	6
		1999	8,726	7,186	10,741	35	242.73	199.91	298.79	6
		2000	8,723	6,732	11,332	32	236.34	182.40	307.03	6
		2001	8,636	6,216	11,858	36	228.47	164.44	313.72	6
		2002	8,633	5,693	12,380	33	222.49	146.72	319.05	6
		2003	8,594	5,110	12,775	33	216.84	128.93	322.32	7
		2004	8,638	4,479	13,131	31	212.75	110.31	323.41	7
		2005	8,615	3,835	13,392	29	206.26	91.83	320.64	7
		2006	8,523	3,183	13,508	29	198.39	74.09	314.45	7
		2007	8,247	2,514	13,309	29	188.05	57.32	303.50	7

Metropolitan Statistical Area	PWID Population	Year	Number	Min	Max	Rank	Rate	Min	Max	Rank
Tulsa, OK	Total	1992	4,135	2,720	5,448	82	86.24	56.72	113.63	67
		1993	4,223	3,149	5,452	81	87.13	64.97	112.48	58
		1994	4,491	3,590	5,421	81	92.12	73.63	111.19	59
		1995	4,477	3,910	5,391	82	91.27	79.72	109.90	54
		1996	4,652	3,913	5,414	82	93.36	78.52	108.64	53
		1997	4,840	3,915	5,447	80	95.42	77.18	107.38	50
		1998	5,053	3,920	5,714	78	97.75	75.84	110.54	49
		1999	5,718	5,260	6,340	71	108.62	99.92	120.44	44
		2000	5,490	3,774	7,247	72	103.29	71.01	136.35	46
		2001	6,412	5,511	8,171	67	119.48	102.70	152.26	36
		2002	5,962	3,555	9,170	67	109.97	65.58	169.13	40
		2003	6,158	3,418	10,114	66	113.26	62.86	186.00	36
		2004	6,347	3,248	11,063	67	116.57	59.65	203.19	31
		2005	6,577	3,115	12,080	64	119.98	56.83	220.38	27
		2006	6,863	2,999	13,224	58	123.60	54.01	238.16	26
		2007	7,176	2,910	14,424	58	127.33	51.63	255.94	24
	Non-Hispanic White	1992	3,002	1,974	3,955	69	76.24	50.15	100.46	48
		1993	3,110	2,319	4,015	67	78.60	58.61	101.46	48
		1994	3,327	2,660	4,016	67	84.11	67.23	101.52	45
		1995	3,315	2,895	3,992	64	83.83	73.22	100.94	48
		1996	3,425	2,881	3,986	64	85.89	72.23	99.95	45
		1997	3,529	2,855	3,972	59	87.65	70.90	98.64	44
		1998	3,637	2,822	4,113	55	89.44	69.39	101.14	41
		1999	4,054	3,730	4,496	51	98.86	90.95	109.63	32
		2000	3,831	2,634	5,057	53	93.30	64.15	123.17	36
		2001	4,404	3,785	5,612	48	106.86	91.85	136.18	29
		2002	4,038	2,408	6,211	49	97.54	58.16	150.01	33
		2003	4,127	2,290	6,777	48	99.87	55.43	164.01	31
		2004	4,228	2,164	7,369	49	102.71	52.56	179.03	32
		2005	4,383	2,076	8,051	52	106.34	50.37	195.31	29
		2006	4,611	2,015	8,885	51	111.11	48.55	214.09	24
		2007	4,901	1,987	9,851	47	117.10	47.48	235.39	22

Metropolitan Statistical Area	PWID Population	Year	Number	Min	Max	Rank	Rate	Min	Max	Rank
Tulsa, OK	Non-Hispanic Black	1992	358	236	472	95	94.01	61.84	123.88	98
		1993	347	259	448	96	89.27	66.57	115.24	97
		1994	344	275	415	96	86.85	69.42	104.83	97
		1995	315	275	379	96	77.96	68.09	93.88	96
		1996	297	250	346	96	71.64	60.26	83.37	96
		1997	279	226	314	96	65.16	52.70	73.33	97
		1998	262	204	297	95	59.10	45.85	66.83	98
		1999	268	247	297	95	58.48	53.80	64.85	98
		2000	234	161	309	95	49.72	34.19	65.64	98
		2001	251	216	320	95	52.54	45.16	66.95	97
		2002	218	130	335	95	44.73	26.67	68.79	98
		2003	214	119	351	95	43.62	24.21	71.63	99
		2004	215	110	375	95	43.35	22.19	75.57	99
		2005	224	106	412	96	44.59	21.12	81.90	99
		2006	244	107	471	96	47.62	20.81	91.75	95
		2007	278	113	559	95	52.84	21.42	106.21	93
	Hispanic	1992	75	49	99	79	66.30	43.61	87.37	75
		1993	72	54	93	80	58.38	43.53	75.37	75
		1994	73	58	88	82	53.78	42.98	64.91	79
		1995	70	61	84	82	47.12	41.16	56.75	78
		1996	71	59	82	82	42.68	35.90	49.67	80
		1997	72	58	81	82	38.71	31.31	43.56	82
		1998	75	58	84	82	35.56	27.59	40.22	81
		1999	84	78	94	82	35.74	32.88	39.63	83
		2000	82	56	108	82	31.72	21.81	41.87	81
		2001	97	83	123	81	34.66	29.79	44.17	80
		2002	92	55	141	82	30.56	18.23	47.00	81
		2003	98	54	161	81	30.80	17.09	50.58	80
		2004	105	54	182	81	31.54	16.14	54.97	78
		2005	114	54	209	82	32.38	15.34	59.47	79
		2006	125	55	241	82	33.54	14.66	64.63	76
		2007	140	57	281	83	35.13	14.25	70.62	75

Metropolitan Statistical Area	PWID Population	Year	Number	Min	Max	Rank	Rate	Min	Max	Rank
Tulsa, OK	Male	1992	2,663	1,752	3,509	82	112.90	74.26	148.76	65
		1993	2,666	1,988	3,441	82	111.64	83.25	144.12	62
		1994	2,781	2,223	3,357	81	115.67	92.45	139.61	61
		1995	2,723	2,379	3,279	82	112.40	98.18	135.35	59
		1996	2,783	2,341	3,239	82	112.99	95.03	131.49	57
		1997	2,850	2,305	3,207	83	113.67	91.94	127.92	55
		1998	2,931	2,274	3,315	83	114.66	88.96	129.66	54
		1999	3,270	3,008	3,626	80	125.56	115.51	139.23	49
		2000	3,096	2,129	4,087	81	117.76	80.97	155.46	53
		2001	3,568	3,066	4,546	76	134.08	115.25	170.87	46
		2002	3,273	1,952	5,034	77	121.52	72.47	186.90	46
		2003	3,334	1,851	5,476	75	123.50	68.54	202.82	44
		2004	3,388	1,734	5,905	75	125.12	64.03	218.09	42
		2005	3,459	1,638	6,353	71	126.86	60.09	233.01	43
		2006	3,552	1,552	6,844	72	128.71	56.24	248.00	40
		2007	3,649	1,480	7,336	73	130.12	52.76	261.55	37
	Female	1992	1,585	1,042	2,088	75	65.05	42.79	85.71	59
		1993	1,643	1,225	2,120	75	66.79	49.80	86.21	51
		1994	1,777	1,420	2,145	75	71.92	57.49	86.81	51
		1995	1,805	1,577	2,174	75	72.72	63.52	87.57	47
		1996	1,914	1,610	2,228	75	75.95	63.88	88.38	45
		1997	2,033	1,645	2,288	69	79.26	64.11	89.20	45
		1998	2,168	1,682	2,451	65	82.96	64.36	93.81	40
		1999	2,504	2,303	2,776	60	94.12	86.59	104.37	29
		2000	2,452	1,686	3,237	60	91.29	62.76	120.51	28
		2001	2,917	2,507	3,717	56	107.82	92.67	137.40	19
		2002	2,758	1,645	4,242	52	101.10	60.29	155.49	20
		2003	2,891	1,605	4,748	51	105.62	58.62	173.45	18
		2004	3,017	1,544	5,258	48	110.23	56.41	192.13	18
		2005	3,156	1,495	5,796	46	114.54	54.26	210.38	15
		2006	3,314	1,448	6,386	45	118.65	51.85	228.62	14
		2007	3,475	1,409	6,985	42	122.74	49.76	246.71	12

Metropolitan Statistical Area	PWID Population	Year	Number	Min	Max	Rank	Rate	Min	Max	Rank
Tulsa, OK	Young (15-29)	1992	1,590	1,046	2,095	69	103.12	67.83	135.88	38
		1993	1,613	1,202	2,082	61	104.79	78.14	135.28	37
		1994	1,710	1,367	2,064	64	111.65	89.24	134.77	31
		1995	1,705	1,490	2,054	57	110.83	96.81	133.46	31
		1996	1,777	1,495	2,068	60	113.61	95.55	132.21	34
		1997	1,857	1,502	2,090	58	116.24	94.02	130.82	34
		1998	1,949	1,512	2,204	57	119.39	92.63	135.01	31
		1999	2,216	2,038	2,457	54	133.50	122.81	148.03	27
		2000	2,135	1,468	2,819	56	128.43	88.30	169.54	25
		2001	2,498	2,147	3,183	49	149.81	128.76	190.91	19
		2002	2,319	1,383	3,567	53	138.25	82.45	212.63	25
		2003	2,384	1,323	3,914	56	142.14	78.89	233.43	24
		2004	2,432	1,245	4,240	58	145.25	74.33	253.18	23
		2005	2,482	1,176	4,558	60	147.33	69.78	270.60	25
		2006	2,533	1,107	4,881	59	148.08	64.71	285.33	23
		2007	2,571	1,042	5,167	57	146.85	59.54	295.18	23
	Old (30-64)	1992	2,677	1,761	3,528	85	82.30	54.14	108.45	79
		1993	2,691	2,007	3,474	83	81.36	60.67	105.03	75
		1994	2,832	2,264	3,419	85	84.71	67.71	102.24	77
		1995	2,807	2,452	3,380	83	83.38	72.83	100.40	69
		1996	2,911	2,448	3,387	83	85.13	71.60	99.07	69
		1997	3,030	2,450	3,409	83	87.18	70.52	98.11	60
		1998	3,168	2,458	3,583	80	89.58	69.50	101.30	59
		1999	3,593	3,305	3,984	76	99.68	91.71	110.54	54
		2000	3,455	2,375	4,561	79	94.58	65.03	124.86	55
		2001	4,034	3,467	5,140	74	109.05	93.73	138.97	45
		2002	3,740	2,230	5,752	74	99.89	59.57	153.63	46
		2003	3,837	2,130	6,302	69	102.04	56.64	167.58	43
		2004	3,910	2,001	6,815	66	103.71	53.08	180.78	39
		2005	3,981	1,886	7,313	64	104.85	49.67	192.59	33
		2006	4,050	1,770	7,803	63	105.40	46.06	203.10	31
		2007	4,085	1,656	8,212	59	105.15	42.63	211.35	27

Metropolitan Statistical Area	PWID Population	Year	Number	Min	Max	Rank	Rate	Min	Max	Rank
Ventura, CA	Total	1992	4,540	2,261	5,800	80	100.40	50.00	128.26	54
		1993	5,536	2,166	8,887	71	122.31	47.86	196.36	41
		1994	4,334	2,103	5,841	82	95.04	46.11	128.09	54
		1995	5,331	2,028	8,634	72	116.63	44.36	188.89	41
		1996	5,243	1,978	8,506	73	113.98	43.00	184.90	44
		1997	5,224	1,954	8,477	73	111.33	41.63	180.66	45
		1998	5,209	1,952	8,436	75	108.94	40.82	176.42	46
		1999	4,142	1,955	6,315	85	84.75	40.01	129.20	60
		2000	5,187	2,059	8,291	76	103.94	41.25	166.14	45
		2001	4,140	2,152	6,393	85	81.73	42.49	126.22	59
		2002	5,026	2,273	7,688	78	97.32	44.01	148.85	47
		2003	4,916	2,381	7,331	80	94.02	45.53	140.21	50
		2004	4,793	2,483	6,959	80	90.85	47.07	131.90	53
		2005	4,633	2,571	6,514	82	87.62	48.61	123.18	56
		2006	4,489	2,663	6,098	85	84.25	49.98	114.45	56
		2007	4,316	2,743	5,934	85	80.81	51.35	111.09	60
	Non-Hispanic White	1992	1,984	988	2,534	85	67.91	33.82	86.76	56
		1993	2,152	842	3,454	75	74.84	29.28	120.15	52
		1994	1,567	760	2,112	92	54.96	26.66	74.07	65
		1995	1,870	711	3,029	83	66.38	25.25	107.51	55
		1996	1,849	697	2,999	84	66.08	24.93	107.20	55
		1997	1,901	711	3,084	84	67.33	25.18	109.26	55
		1998	1,988	745	3,219	83	69.85	26.17	113.12	56
		1999	1,669	788	2,544	87	57.98	27.37	88.38	64
		2000	2,204	875	3,522	80	75.87	30.11	121.28	53
		2001	1,838	956	2,839	87	63.05	32.78	97.37	61
		2002	2,298	1,039	3,515	81	78.09	35.32	119.44	47
		2003	2,268	1,098	3,382	81	76.93	37.26	114.73	49
		2004	2,173	1,126	3,155	82	73.83	38.25	107.19	51
		2005	1,996	1,108	2,806	86	68.34	37.92	96.08	56
		2006	1,758	1,043	2,388	93	60.48	35.88	82.16	66
		2007	1,448	920	1,991	96	50.30	31.97	69.15	78

Metropolitan Statistical Area	PWID Population	Year	Number	Min	Max	Rank	Rate	Min	Max	Rank
Ventura, CA	Non-Hispanic Black	1992	164	82	210	100	162.48	80.92	207.56	84
		1993	178	69	285	100	176.86	69.20	283.94	77
		1994	125	61	168	100	123.16	59.75	165.99	93
		1995	140	53	226	100	138.74	52.77	224.71	83
		1996	127	48	206	100	125.81	47.46	204.09	84
		1997	118	44	192	100	116.85	43.70	189.62	83
		1998	112	42	182	100	110.85	41.54	179.51	83
		1999	86	41	131	100	84.21	39.75	128.37	92
		2000	106	42	170	99	102.28	40.59	163.49	83
		2001	85	44	131	99	81.58	42.42	125.99	89
		2002	106	48	162	99	98.28	44.44	150.31	79
		2003	108	52	161	99	99.94	48.40	149.05	73
		2004	112	58	162	99	102.71	53.22	149.12	72
		2005	118	65	166	99	107.14	59.45	150.63	68
		2006	128	76	173	99	113.82	67.52	154.61	65
		2007	141	90	194	99	129.11	82.06	177.50	59
	Hispanic	1992	2,140	1,066	2,734	32	174.52	86.92	222.95	37
		1993	2,746	1,075	4,409	29	216.60	84.75	347.74	29
		1994	2,220	1,077	2,992	31	168.75	81.87	227.44	31
		1995	2,777	1,056	4,497	29	205.16	78.03	332.28	27
		1996	2,744	1,035	4,451	29	196.68	74.19	319.06	26
		1997	2,722	1,018	4,417	27	187.46	70.10	304.18	22
		1998	2,684	1,006	4,347	26	177.79	66.62	287.93	21
		1999	2,102	992	3,204	33	133.79	63.16	203.96	32
		2000	2,586	1,026	4,134	26	158.10	62.75	252.72	23
		2001	2,029	1,055	3,133	36	120.39	62.59	185.92	33
		2002	2,429	1,098	3,715	28	139.55	63.11	213.44	24
		2003	2,356	1,141	3,514	29	131.80	63.82	196.55	26
		2004	2,299	1,191	3,338	31	125.78	65.17	182.62	28
		2005	2,250	1,248	3,163	31	121.25	67.28	170.46	28
		2006	2,238	1,327	3,040	32	118.15	70.10	160.50	28
		2007	2,243	1,425	3,083	32	116.13	73.80	159.65	27

Metropolitan Statistical Area	PWID Population	Year	Number	Min	Max	Rank	Rate	Min	Max	Rank
Ventura, CA	Male	1992	2,698	1,343	3,446	81	116.30	57.92	148.57	64
		1993	3,270	1,279	5,249	78	140.99	55.17	226.35	46
		1994	2,559	1,241	3,449	84	109.67	53.21	147.81	68
		1995	3,161	1,202	5,120	81	135.43	51.51	219.34	45
		1996	3,135	1,183	5,086	81	133.71	50.44	216.90	46
		1997	3,160	1,182	5,128	79	132.38	49.50	214.82	46
		1998	3,194	1,197	5,173	77	131.62	49.32	213.15	46
		1999	2,578	1,217	3,930	85	104.07	49.12	158.64	63
		2000	3,279	1,301	5,242	74	129.64	51.45	207.22	45
		2001	2,658	1,382	4,104	84	103.64	53.89	160.06	62
		2002	3,274	1,481	5,007	76	124.91	56.49	191.06	44
		2003	3,244	1,571	4,837	76	122.39	59.27	182.52	45
		2004	3,198	1,657	4,643	77	119.51	61.93	173.53	47
		2005	3,118	1,730	4,384	81	116.04	64.39	163.14	49
		2006	3,038	1,802	4,127	82	111.96	66.42	152.09	53
		2007	2,927	1,860	4,025	81	107.86	68.55	148.29	54
	Female	1992	1,824	908	2,330	71	82.82	41.25	105.80	41
		1993	2,261	885	3,630	63	102.47	40.09	164.50	28
		1994	1,785	866	2,406	74	80.15	38.88	108.02	42
		1995	2,197	836	3,558	65	98.22	37.36	159.08	27
		1996	2,148	810	3,485	65	95.25	35.93	154.52	29
		1997	2,116	791	3,434	67	91.78	34.32	148.93	28
		1998	2,076	778	3,362	69	88.15	33.03	142.76	34
		1999	1,618	764	2,466	84	67.12	31.68	102.32	57
		2000	1,979	785	3,163	74	80.40	31.91	128.52	41
		2001	1,539	800	2,377	85	61.53	31.99	95.03	60
		2002	1,818	822	2,781	78	71.48	32.33	109.33	47
		2003	1,730	838	2,580	81	67.10	32.49	100.06	51
		2004	1,641	851	2,383	83	63.13	32.71	91.67	56
		2005	1,547	858	2,175	85	59.48	33.00	83.62	61
		2006	1,466	869	1,991	86	56.05	33.26	76.15	63
		2007	1,384	879	1,902	88	52.66	33.47	72.40	66

Metropolitan Statistical Area	PWID Population	Year	Number	Min	Max	Rank	Rate	Min	Max	Rank
Ventura, CA	Young (15-29)	1992	1,326	660	1,693	76	88.65	44.15	113.25	52
		1993	1,564	612	2,511	65	106.99	41.87	171.77	32
		1994	1,214	589	1,636	77	84.08	40.79	113.33	53
		1995	1,509	574	2,443	67	105.90	40.28	171.52	38
		1996	1,521	574	2,468	68	107.17	40.43	173.85	36
		1997	1,569	587	2,545	67	109.24	40.85	177.27	37
		1998	1,625	609	2,632	67	111.73	41.86	180.94	35
		1999	1,343	634	2,047	81	90.68	42.80	138.23	47
		2000	1,738	690	2,777	66	115.02	45.65	183.85	34
		2001	1,420	738	2,193	83	92.65	48.17	143.09	49
		2002	1,742	788	2,664	72	110.41	49.93	168.88	42
		2003	1,690	819	2,521	74	105.39	51.04	157.17	47
		2004	1,600	829	2,322	77	97.90	50.72	142.14	53
		2005	1,459	810	2,052	85	88.08	48.87	123.84	56
		2006	1,289	765	1,751	89	75.92	45.04	103.13	68
		2007	1,082	688	1,488	92	63.18	40.15	86.86	85
	Old (30-64)	1992	3,283	1,635	4,193	82	108.46	54.02	138.56	55
		1993	4,032	1,578	6,473	69	131.59	51.49	211.25	42
		1994	3,162	1,534	4,262	82	101.45	49.22	136.73	61
		1995	3,878	1,475	6,281	75	123.27	46.88	199.64	42
		1996	3,790	1,430	6,149	76	119.16	44.95	193.30	43
		1997	3,741	1,399	6,071	76	114.88	42.96	186.42	43
		1998	3,688	1,382	5,972	76	110.85	41.54	179.52	45
		1999	2,895	1,367	4,414	84	84.99	40.12	129.56	62
		2000	3,579	1,421	5,721	78	102.86	40.82	164.41	47
		2001	2,823	1,468	4,360	83	79.92	41.55	123.43	62
		2002	3,397	1,536	5,196	78	94.70	42.83	144.85	49
		2003	3,307	1,601	4,931	78	91.23	44.18	136.05	48
		2004	3,227	1,672	4,685	78	88.60	45.91	128.65	49
		2005	3,143	1,744	4,419	78	86.56	48.03	121.70	48
		2006	3,092	1,835	4,201	77	85.18	50.54	115.72	47
		2007	3,041	1,933	4,181	74	83.82	53.27	115.24	44

Metropolitan Statistical Area	PWID Population	Year	Number	Min	Max	Rank	Rate	Min	Max	Rank
Washington, DC--MD--VA--WV	Total	1992	27,585	7,075	39,577	9	89.80	23.03	128.84	61
		1993	24,989	7,095	38,651	13	80.52	22.86	124.54	66
		1994	28,306	7,125	40,050	8	90.18	22.70	127.60	63
		1995	26,170	7,142	42,000	11	82.59	22.54	132.55	64
		1996	26,843	7,184	44,184	10	83.51	22.35	137.46	63
		1997	27,481	7,220	46,374	9	84.36	22.16	142.36	60
		1998	28,232	7,367	48,778	9	85.39	22.28	147.54	58
		1999	31,016	7,555	51,495	8	91.98	22.40	152.71	56
		2000	30,281	7,910	54,860	8	87.88	22.96	159.22	57
		2001	33,272	8,286	58,414	8	94.40	23.51	165.73	54
		2002	31,979	9,755	61,959	8	89.14	27.19	172.71	54
		2003	32,858	11,239	65,413	8	90.26	30.87	179.68	52
		2004	33,904	13,316	69,103	7	91.75	36.04	187.00	51
		2005	34,980	15,460	72,918	7	93.21	41.20	194.31	49
		2006	35,940	17,717	76,517	7	94.95	46.81	202.15	48
		2007	36,886	18,395	80,157	7	96.63	48.19	209.99	44
	Non-Hispanic White	1992	14,015	3,595	20,107	11	72.39	18.57	103.86	51
		1993	11,474	3,258	17,747	17	59.40	16.87	91.88	60
		1994	11,894	2,994	16,829	13	61.57	15.50	87.12	60
		1995	10,219	2,789	16,400	18	53.03	14.47	85.10	65
		1996	9,909	2,652	16,310	17	51.34	13.74	84.51	66
		1997	9,764	2,565	16,477	18	50.58	13.29	85.35	68
		1998	9,834	2,566	16,990	18	50.85	13.27	87.85	69
		1999	10,781	2,626	17,899	18	55.33	13.48	91.86	68
		2000	10,681	2,790	19,351	17	54.25	14.17	98.28	68
		2001	12,094	3,012	21,232	17	60.49	15.06	106.19	63
		2002	12,138	3,703	23,517	13	60.20	18.36	116.64	64
		2003	13,167	4,504	26,213	11	64.95	22.21	129.29	60
		2004	14,463	5,681	29,479	9	70.91	27.85	144.54	55
		2005	15,975	7,060	33,301	7	77.86	34.41	162.31	47
		2006	17,620	8,686	37,513	6	86.09	42.44	183.30	44
		2007	19,418	9,684	42,198	6	94.92	47.34	206.28	41

Metropolitan Statistical Area	PWID Population	Year	Number	Min	Max	Rank	Rate	Min	Max	Rank
Washington, DC--MD--VA--WV	Non-Hispanic Black	1992	9,761	2,504	14,004	9	126.91	32.55	182.08	94
		1993	10,565	3,000	16,341	8	135.21	38.39	209.13	88
		1994	13,594	3,422	19,235	7	171.16	43.08	242.17	79
		1995	13,731	3,747	22,037	7	170.34	46.48	273.37	72
		1996	14,928	3,995	24,572	7	181.56	48.59	298.85	66
		1997	15,810	4,154	26,680	6	188.96	49.64	318.88	54
		1998	16,466	4,297	28,448	5	192.86	50.33	333.21	51
		1999	18,007	4,386	29,897	4	206.27	50.25	342.47	47
		2000	17,200	4,493	31,162	3	192.95	50.40	349.56	43
		2001	18,170	4,525	31,900	3	200.02	49.81	351.17	40
		2002	16,483	5,028	31,937	3	178.17	54.35	345.21	42
		2003	15,674	5,361	31,203	3	167.05	57.14	332.56	46
		2004	14,656	5,756	29,871	3	153.73	60.38	313.34	49
		2005	13,409	5,927	27,953	4	138.39	61.17	288.49	55
		2006	11,964	5,898	25,472	8	121.92	60.10	259.57	62
		2007	10,469	5,221	22,751	8	105.80	52.76	229.91	69
	Hispanic	1992	440	113	631	57	23.46	6.02	33.66	97
		1993	353	100	546	59	17.64	5.01	27.29	99
		1994	374	94	529	56	17.61	4.43	24.92	99
		1995	339	92	543	58	15.07	4.11	24.19	99
		1996	354	95	583	58	14.78	3.96	24.33	99
		1997	382	100	644	57	14.95	3.93	25.23	97
		1998	424	111	733	57	15.56	4.06	26.88	95
		1999	514	125	854	54	17.72	4.32	29.42	93
		2000	561	146	1,016	55	18.11	4.73	32.82	92
		2001	692	172	1,215	53	21.08	5.25	37.00	91
		2002	748	228	1,449	52	21.59	6.58	41.82	89
		2003	858	294	1,708	51	23.54	8.05	46.86	88
		2004	977	384	1,991	51	25.59	10.05	52.17	86
		2005	1,093	483	2,278	49	27.32	12.08	56.96	82
		2006	1,190	587	2,533	46	28.58	14.09	60.84	82
		2007	1,258	628	2,734	46	29.16	14.54	63.38	81

Metropolitan Statistical Area	PWID Population	Year	Number	Min	Max	Rank	Rate	Min	Max	Rank
Washington, DC--MD--VA--WV	Male	1992	16,500	4,232	23,673	12	109.00	27.96	156.38	71
		1993	15,549	4,415	24,051	15	101.74	28.88	157.36	67
		1994	18,114	4,559	25,629	9	117.24	29.51	165.88	60
		1995	17,064	4,657	27,386	11	109.52	29.89	175.77	61
		1996	17,702	4,738	29,137	9	112.07	30.00	184.47	58
		1997	18,216	4,786	30,739	9	113.93	29.93	192.26	54
		1998	18,716	4,884	32,336	9	115.48	30.14	199.52	53
		1999	20,478	4,988	33,999	8	123.99	30.20	205.86	51
		2000	19,846	5,184	35,955	9	117.55	30.71	212.97	54
		2001	21,592	5,377	37,908	8	125.12	31.16	219.66	51
		2002	20,513	6,257	39,745	9	116.91	35.66	226.52	52
		2003	20,818	7,121	41,443	8	116.97	40.01	232.87	49
		2004	21,218	8,334	43,246	8	117.49	46.15	239.47	48
		2005	21,650	9,568	45,130	7	117.86	52.09	245.69	47
		2006	22,048	10,869	46,941	7	118.88	58.60	253.10	48
		2007	22,506	11,224	48,908	7	120.22	59.95	261.25	45
	Female	1992	10,880	2,791	15,610	8	69.83	17.91	100.19	52
		1993	9,366	2,659	14,487	13	59.46	16.88	91.97	61
		1994	10,213	2,571	14,450	11	64.08	16.13	90.67	60
		1995	9,201	2,511	14,766	14	57.13	15.59	91.69	62
		1996	9,298	2,489	15,305	15	56.88	15.22	93.62	62
		1997	9,471	2,488	15,982	12	57.10	15.00	96.36	62
		1998	9,758	2,547	16,860	11	57.90	15.11	100.03	60
		1999	10,821	2,636	17,965	9	62.89	15.32	104.42	58
		2000	10,712	2,798	19,407	8	60.96	15.92	110.44	59
		2001	11,969	2,981	21,014	9	66.54	16.57	116.82	55
		2002	11,712	3,573	22,692	8	63.90	19.49	123.81	57
		2003	12,246	4,189	24,380	8	65.81	22.51	131.02	54
		2004	12,833	5,040	26,156	7	67.92	26.68	138.43	51
		2005	13,399	5,922	27,930	6	69.94	30.91	145.79	45
		2006	13,862	6,834	29,513	6	71.80	35.40	152.88	42
		2007	14,234	7,099	30,932	6	73.18	36.50	159.03	39

Metropolitan Statistical Area	PWID Population	Year	Number	Min	Max	Rank	Rate	Min	Max	Rank
Washington, DC--MD--VA--WV	Young (15-29)	1992	6,510	1,670	9,341	16	64.10	16.44	91.97	69
		1993	5,066	1,438	7,836	20	50.68	14.39	78.38	74
		1994	5,178	1,303	7,326	20	52.54	13.22	74.34	73
		1995	4,528	1,236	7,267	22	46.50	12.69	74.62	79
		1996	4,586	1,227	7,549	22	47.22	12.64	77.72	77
		1997	4,811	1,264	8,119	19	49.50	13.01	83.54	74
		1998	5,222	1,363	9,022	18	53.34	13.92	92.16	72
		1999	6,203	1,511	10,299	14	62.73	15.28	104.15	66
		2000	6,654	1,738	12,056	11	66.45	17.36	120.38	68
		2001	8,106	2,019	14,231	9	79.82	19.88	140.14	64
		2002	8,654	2,640	16,767	8	84.54	25.79	163.80	58
		2003	9,836	3,364	19,581	8	95.04	32.51	189.21	51
		2004	11,119	4,367	22,663	8	105.63	41.49	215.30	48
		2005	12,393	5,477	25,833	7	115.14	50.89	240.03	36
		2006	13,510	6,660	28,764	6	124.02	61.14	264.04	31
		2007	14,407	7,185	31,307	6	130.69	65.17	284.00	29
	Old (30-64)	1992	21,406	5,490	30,712	8	104.11	26.70	149.36	61
		1993	20,068	5,697	31,039	14	95.38	27.08	147.53	62
		1994	23,212	5,843	32,842	7	107.80	27.13	152.52	56
		1995	21,702	5,922	34,829	12	98.89	26.98	158.70	57
		1996	22,340	5,979	36,771	9	99.60	26.66	163.94	56
		1997	22,802	5,990	38,478	9	99.77	26.21	168.36	55
		1998	23,210	6,057	40,101	9	99.74	26.03	172.32	54
		1999	25,107	6,116	41,685	8	105.35	25.66	174.91	52
		2000	23,979	6,264	43,443	7	98.11	25.63	177.75	52
		2001	25,598	6,375	44,941	8	102.02	25.41	179.11	51
		2002	23,731	7,239	45,979	8	92.56	28.23	179.34	52
		2003	23,349	7,986	46,481	8	89.61	30.65	178.39	52
		2004	22,905	8,996	46,685	8	86.67	34.04	176.65	52
		2005	22,323	9,866	46,533	8	83.41	36.86	173.86	51
		2006	21,548	10,623	45,878	8	79.93	39.41	170.18	51
		2007	20,709	10,328	45,003	8	76.28	38.04	165.77	52

Metropolitan Statistical Area	PWID Population	Year	Number	Min	Max	Rank	Rate	Min	Max	Rank
West Palm Beach--Boca Raton, FL	Total	1992	9,290	5,175	12,601	48	173.03	96.39	234.69	23
		1993	7,267	1,815	11,603	57	131.87	32.94	210.56	36
		1994	8,903	6,036	10,605	51	156.72	106.25	186.69	26
		1995	7,086	2,164	10,024	57	121.44	37.09	171.79	38
		1996	7,019	2,322	9,946	60	117.35	38.83	166.28	41
		1997	7,000	2,476	9,937	60	113.25	40.05	160.76	43
		1998	7,018	2,629	9,910	58	110.18	41.27	155.58	43
		1999	8,393	6,608	9,848	51	128.18	100.91	150.39	33
		2000	6,742	2,850	9,633	59	100.51	42.49	143.61	48
		2001	7,550	5,420	9,400	55	109.91	78.90	136.84	45
		2002	5,909	2,702	9,167	68	83.58	38.22	129.66	60
		2003	5,363	2,614	8,876	74	74.01	36.08	122.49	66
		2004	4,927	2,541	8,610	79	65.92	34.00	115.19	71
		2005	4,610	2,408	8,246	84	60.31	31.50	107.89	78
		2006	4,223	2,232	7,717	86	54.90	29.01	100.32	89
		2007	3,822	2,022	7,120	88	49.79	26.34	92.75	93
	Non-Hispanic White	1992	5,942	3,310	8,059	39	147.71	82.28	200.34	21
		1993	4,897	1,223	7,819	46	120.20	30.02	191.92	28
		1994	6,173	4,185	7,353	36	148.81	100.89	177.26	18
		1995	4,973	1,519	7,035	40	118.32	36.14	167.39	26
		1996	4,925	1,630	6,979	41	115.91	38.35	164.25	25
		1997	4,868	1,722	6,911	39	112.31	39.72	159.43	23
		1998	4,807	1,801	6,788	38	108.95	40.81	153.83	25
		1999	5,641	4,441	6,618	37	126.12	99.29	147.97	21
		2000	4,441	1,878	6,346	43	98.25	41.54	140.38	32
		2001	4,886	3,508	6,083	42	107.02	76.83	133.24	28
		2002	3,779	1,728	5,863	56	81.52	37.28	126.47	44
		2003	3,422	1,668	5,663	66	73.09	35.63	120.96	54
		2004	3,177	1,638	5,551	72	66.71	34.40	116.57	59
		2005	3,048	1,592	5,452	75	63.43	33.14	113.48	64
		2006	2,907	1,537	5,313	79	61.08	32.28	111.60	65
		2007	2,777	1,469	5,173	80	59.27	31.36	110.40	66

Metropolitan Statistical Area	PWID Population	Year	Number	Min	Max	Rank	Rate	Min	Max	Rank
West Palm Beach--Boca Raton, FL	Non-Hispanic Black	1992	1,787	995	2,424	61	247.34	137.79	335.48	61
		1993	1,245	311	1,988	65	163.99	40.96	261.84	82
		1994	1,413	958	1,683	63	176.45	119.62	210.18	76
		1995	1,077	329	1,524	67	128.12	39.13	181.25	88
		1996	1,050	347	1,487	67	119.68	39.60	169.59	87
		1997	1,051	372	1,492	65	113.69	40.21	161.39	85
		1998	1,072	402	1,514	65	110.40	41.35	155.88	84
		1999	1,312	1,033	1,540	61	129.04	101.59	151.40	78
		2000	1,077	455	1,539	64	102.38	43.29	146.29	82
		2001	1,222	878	1,522	62	112.22	80.56	139.71	79
		2002	955	436	1,481	64	84.47	38.62	131.04	87
		2003	845	412	1,399	68	72.05	35.13	119.25	89
		2004	735	379	1,285	72	59.91	30.90	104.69	92
		2005	628	328	1,123	77	49.39	25.80	88.36	95
		2006	503	266	919	85	38.59	20.39	70.52	100
		2007	378	200	704	91	28.71	15.19	53.48	100
	Hispanic	1992	469	261	636	55	87.18	48.57	118.25	65
		1993	376	94	601	57	64.64	16.15	103.21	68
		1994	486	329	578	54	77.30	52.40	92.07	67
		1995	414	126	586	56	60.94	18.61	86.21	71
		1996	444	147	629	56	60.45	20.00	85.66	68
		1997	480	170	682	54	60.70	21.46	86.16	65
		1998	520	195	734	54	61.38	22.99	86.67	62
		1999	664	523	779	52	72.87	57.37	85.50	60
		2000	560	237	800	56	57.15	24.16	81.65	63
		2001	642	461	799	54	61.13	43.88	76.10	62
		2002	498	228	773	57	44.20	20.21	68.58	67
		2003	433	211	716	60	35.93	17.52	59.47	75
		2004	364	188	636	63	28.29	14.59	49.43	81
		2005	296	155	529	66	21.81	11.39	39.01	91
		2006	222	117	406	71	15.75	8.32	28.78	96
		2007	154	82	287	77	10.63	5.63	19.81	99

Metropolitan Statistical Area	PWID Population	Year	Number	Min	Max	Rank	Rate	Min	Max	Rank
West Palm Beach--Boca Raton, FL	Male	1992	5,883	3,277	7,979	49	222.55	123.98	301.86	25
		1993	4,669	1,166	7,454	55	172.01	42.96	274.65	36
		1994	5,749	3,898	6,849	47	205.59	139.38	244.90	28
		1995	4,565	1,394	6,458	55	158.96	48.55	224.87	40
		1996	4,484	1,484	6,354	59	152.30	50.39	215.80	42
		1997	4,413	1,561	6,264	59	144.98	51.27	205.81	44
		1998	4,350	1,629	6,142	58	138.65	51.94	195.78	44
		1999	5,103	4,017	5,987	53	158.15	124.50	185.55	36
		2000	4,018	1,699	5,741	63	121.51	51.37	173.63	49
		2001	4,414	3,169	5,496	61	130.17	93.44	162.05	48
		2002	3,399	1,554	5,273	73	97.23	44.46	150.83	65
		2003	3,050	1,487	5,048	81	84.86	41.37	140.44	69
		2004	2,790	1,439	4,875	85	75.09	38.73	131.22	78
		2005	2,621	1,369	4,688	86	68.88	35.98	123.22	85
		2006	2,436	1,288	4,452	87	63.50	33.56	116.03	93
		2007	2,263	1,197	4,216	88	59.01	31.22	109.92	96
	Female	1992	3,409	1,899	4,623	43	125.05	69.66	169.61	21
		1993	2,601	650	4,154	54	93.02	23.23	148.52	32
		1994	3,158	2,141	3,762	45	109.50	74.23	130.43	22
		1995	2,524	771	3,570	55	85.17	26.02	120.49	37
		1996	2,537	839	3,594	56	83.52	27.63	118.34	39
		1997	2,587	915	3,672	56	82.44	29.16	117.03	41
		1998	2,665	998	3,762	54	82.43	30.88	116.39	43
		1999	3,281	2,583	3,850	46	98.79	77.78	115.91	25
		2000	2,713	1,147	3,876	55	79.75	33.72	113.96	42
		2001	3,118	2,238	3,882	48	89.65	64.36	111.61	32
		2002	2,492	1,140	3,866	62	69.74	31.89	108.19	49
		2003	2,294	1,118	3,796	65	62.81	30.62	103.96	58
		2004	2,118	1,092	3,700	68	56.32	29.04	98.41	62
		2005	1,968	1,028	3,521	79	51.27	26.78	91.72	67
		2006	1,767	934	3,228	81	45.82	24.21	83.72	79
		2007	1,540	815	2,869	85	40.10	21.21	74.69	85

Metropolitan Statistical Area	PWID Population	Year	Number	Min	Max	Rank	Rate	Min	Max	Rank
West Palm Beach--Boca Raton, FL	Young (15-29)	1992	2,022	1,127	2,743	54	129.79	72.30	176.04	26
		1993	1,578	394	2,520	64	101.14	25.26	161.49	43
		1994	1,993	1,351	2,374	52	127.08	86.15	151.38	24
		1995	1,679	513	2,375	59	106.38	32.49	150.49	37
		1996	1,796	594	2,545	59	112.35	37.17	159.19	35
		1997	1,960	693	2,782	55	119.77	42.36	170.02	31
		1998	2,164	811	3,055	53	129.07	48.35	182.25	25
		1999	2,851	2,245	3,345	40	166.46	131.05	195.30	14
		2000	2,511	1,062	3,588	47	144.12	60.93	205.92	19
		2001	3,054	2,192	3,802	41	170.96	122.72	212.84	14
		2002	2,563	1,172	3,975	48	137.86	63.04	213.86	26
		2003	2,454	1,197	4,062	53	127.20	62.01	210.52	26
		2004	2,338	1,206	4,085	61	115.28	59.45	201.44	39
		2005	2,222	1,161	3,974	68	106.15	55.46	189.90	45
		2006	2,020	1,068	3,691	74	95.31	50.37	174.17	51
		2007	1,765	934	3,288	79	83.08	43.95	154.75	56
	Old (30-64)	1992	7,359	4,099	9,981	46	193.10	107.57	261.91	24
		1993	5,734	1,432	9,156	53	145.16	36.26	231.77	36
		1994	6,947	4,710	8,275	49	168.93	114.52	201.22	28
		1995	5,429	1,658	7,680	56	127.55	38.96	180.44	40
		1996	5,243	1,735	7,429	57	119.64	39.58	169.52	42
		1997	5,061	1,790	7,185	60	111.37	39.38	158.09	45
		1998	4,876	1,826	6,885	61	103.90	38.92	146.70	49
		1999	5,565	4,381	6,530	54	115.10	90.61	135.04	42
		2000	4,241	1,793	6,060	66	85.42	36.11	122.05	59
		2001	4,490	3,223	5,590	67	88.33	63.41	109.97	57
		2002	3,318	1,517	5,147	79	63.67	29.11	98.77	71
		2003	2,850	1,389	4,717	81	53.61	26.13	88.73	81
		2004	2,495	1,287	4,359	86	45.80	23.62	80.03	91
		2005	2,248	1,174	4,021	87	40.49	21.15	72.44	95
		2006	2,014	1,064	3,680	87	36.13	19.09	66.02	95
		2007	1,817	961	3,385	91	32.73	17.32	60.97	95

Metropolitan Statistical Area	PWID Population	Year	Number	Min	Max	Rank	Rate	Min	Max	Rank
Wichita, KS	Total	1992	1,686	1,040	2,512	97	52.23	32.21	77.81	95
		1993	1,809	1,097	2,457	97	55.35	33.57	75.18	88
		1994	1,722	1,158	2,401	97	52.29	35.17	72.91	94
		1995	1,883	1,223	2,349	96	56.62	36.76	70.64	84
		1996	1,934	1,296	2,398	96	57.42	38.47	71.19	83
		1997	1,986	1,374	2,485	96	58.11	40.18	72.70	82
		1998	2,066	1,526	2,586	94	59.27	43.78	74.21	80
		1999	1,946	1,666	2,258	98	55.35	47.38	64.22	88
		2000	2,232	1,797	2,737	93	63.16	50.84	77.45	73
		2001	2,188	1,934	2,605	97	61.44	54.30	73.13	79
		2002	2,403	2,034	2,854	94	66.71	56.47	79.23	70
		2003	2,479	2,121	3,086	94	68.54	58.64	85.33	70
		2004	2,552	2,173	3,345	94	70.18	59.76	92.01	67
		2005	2,625	2,230	3,615	93	71.67	60.88	98.69	66
		2006	2,710	2,282	3,928	94	73.29	61.70	106.22	67
		2007	2,801	2,174	4,261	94	74.79	58.05	113.75	65
	Non-Hispanic White	1992	1,142	704	1,701	98	41.60	25.65	61.97	85
		1993	1,219	739	1,655	96	44.07	26.73	59.87	77
		1994	1,164	783	1,624	97	42.07	28.29	58.66	83
		1995	1,287	836	1,606	95	46.32	30.07	57.79	73
		1996	1,342	899	1,664	94	47.98	32.15	59.49	69
		1997	1,402	970	1,754	92	49.71	34.37	62.19	69
		1998	1,484	1,096	1,858	91	52.03	38.43	65.15	67
		1999	1,422	1,217	1,650	92	49.82	42.64	57.81	72
		2000	1,655	1,332	2,029	89	58.21	46.86	71.38	65
		2001	1,640	1,450	1,952	92	57.51	50.83	68.46	67
		2002	1,812	1,534	2,152	89	63.17	53.47	75.02	61
		2003	1,871	1,601	2,329	91	65.19	55.77	81.16	59
		2004	1,914	1,630	2,509	92	66.61	56.72	87.33	60
		2005	1,939	1,647	2,671	87	67.22	57.11	92.57	60
		2006	1,951	1,642	2,828	87	67.32	56.66	97.56	60
		2007	1,937	1,503	2,946	89	66.28	51.44	100.80	63

Metropolitan Statistical Area	PWID Population	Year	Number	Min	Max	Rank	Rate	Min	Max	Rank
Wichita, KS	Non-Hispanic Black	1992	412	254	614	94	171.48	105.75	255.48	83
		1993	426	258	579	94	174.30	105.71	236.77	78
		1994	375	252	523	95	151.00	101.55	210.55	85
		1995	366	237	456	95	145.17	94.25	181.11	81
		1996	326	218	404	95	127.00	85.10	157.46	83
		1997	284	196	355	95	109.03	75.39	136.40	87
		1998	248	183	310	96	92.00	67.95	115.18	92
		1999	195	167	226	97	71.08	60.84	82.47	94
		2000	189	152	232	96	67.83	54.60	83.18	93
		2001	160	141	190	97	56.56	49.99	67.32	95
		2002	157	133	186	98	54.26	45.93	64.45	93
		2003	151	129	188	98	52.28	44.72	65.08	93
		2004	153	131	201	98	52.34	44.57	68.62	95
		2005	167	142	230	98	56.71	48.18	78.10	92
		2006	198	166	286	98	65.53	55.16	94.97	91
		2007	255	198	388	98	83.45	64.77	126.92	79
	Hispanic	1992	49	30	73	86	35.57	21.94	53.00	93
		1993	56	34	76	84	37.72	22.88	51.24	88
		1994	55	37	77	86	34.89	23.47	48.65	92
		1995	62	40	77	84	36.27	23.55	45.25	87
		1996	64	43	80	84	34.91	23.39	43.28	87
		1997	66	46	82	84	33.13	22.91	41.45	86
		1998	68	50	85	83	31.32	23.13	39.21	86
		1999	63	54	73	87	27.13	23.22	31.48	89
		2000	71	57	88	84	28.55	22.98	35.01	84
		2001	69	61	82	88	26.44	23.36	31.47	87
		2002	75	64	90	86	27.55	23.32	32.72	84
		2003	78	67	97	87	27.38	23.43	34.09	84
		2004	82	70	107	87	27.69	23.58	36.31	83
		2005	87	74	120	86	28.41	24.13	39.12	80
		2006	96	81	139	85	29.86	25.13	43.27	80
		2007	108	84	164	84	32.03	24.86	48.71	76

Metropolitan Statistical Area	PWID Population	Year	Number	Min	Max	Rank	Rate	Min	Max	Rank
Wichita, KS	Male	1992	1,178	726	1,755	98	72.85	44.93	108.53	95
		1993	1,214	736	1,649	98	74.11	44.95	100.68	88
		1994	1,112	748	1,551	98	67.29	45.25	93.82	95
		1995	1,174	763	1,465	98	70.36	45.68	87.78	89
		1996	1,169	783	1,449	98	69.15	46.34	85.74	88
		1997	1,168	808	1,461	98	68.07	47.07	85.16	87
		1998	1,188	877	1,487	97	67.82	50.09	84.91	86
		1999	1,099	941	1,276	99	62.14	53.19	72.10	96
		2000	1,245	1,002	1,527	98	69.96	56.31	85.79	82
		2001	1,212	1,071	1,442	99	67.51	59.66	80.35	89
		2002	1,327	1,124	1,577	96	73.12	61.89	86.84	79
		2003	1,373	1,174	1,709	96	75.40	64.50	93.86	76
		2004	1,423	1,212	1,865	97	77.68	66.15	101.84	74
		2005	1,480	1,257	2,038	96	80.21	68.14	110.46	73
		2006	1,551	1,305	2,247	96	83.03	69.89	120.33	69
		2007	1,632	1,266	2,482	94	86.36	67.03	131.35	68
	Female	1992	500	308	744	99	31.00	19.12	46.19	95
		1993	588	357	799	96	36.07	21.87	48.99	86
		1994	606	408	845	97	36.98	24.87	51.56	87
		1995	710	461	885	95	42.85	27.82	53.45	83
		1996	771	517	956	95	45.97	30.80	57.00	80
		1997	830	574	1,038	95	48.75	33.71	60.99	74
		1998	895	661	1,120	94	51.61	38.12	64.62	69
		1999	866	741	1,005	96	49.58	42.44	57.53	77
		2000	1,011	814	1,240	92	57.66	46.41	70.71	63
		2001	1,001	884	1,191	95	56.65	50.07	67.44	67
		2002	1,099	931	1,306	93	61.55	52.10	73.10	59
		2003	1,125	962	1,400	92	62.63	53.58	77.97	59
		2004	1,138	969	1,492	92	63.05	53.69	82.67	57
		2005	1,139	967	1,568	92	62.63	53.21	86.25	53
		2006	1,132	953	1,640	93	61.82	52.04	89.60	56
		2007	1,113	864	1,692	94	59.95	46.53	91.18	53

Metropolitan Statistical Area	PWID Population	Year	Number	Min	Max	Rank	Rate	Min	Max	Rank
Wichita, KS	Young (15-29)	1992	600	370	895	94	56.09	34.59	83.56	75
		1993	588	357	799	92	55.14	33.44	74.90	68
		1994	532	358	742	95	50.02	33.64	69.75	77
		1995	573	372	715	93	53.68	34.86	66.98	66
		1996	597	400	740	93	55.14	36.94	68.36	67
		1997	635	439	795	93	57.62	39.85	72.09	67
		1998	693	512	868	92	61.47	45.40	76.96	66
		1999	689	590	799	93	60.62	51.89	70.33	69
		2000	831	669	1,019	89	73.13	58.87	89.68	61
		2001	848	750	1,010	93	74.60	65.93	88.80	66
		2002	955	808	1,134	91	83.10	70.34	98.70	61
		2003	988	845	1,230	93	86.29	73.82	107.42	60
		2004	992	845	1,300	94	86.33	73.52	113.19	59
		2005	959	815	1,320	95	83.09	70.59	114.43	66
		2006	887	747	1,285	99	75.92	63.91	110.03	67
		2007	771	599	1,173	99	65.15	50.57	99.09	83
	Old (30-64)	1992	1,102	679	1,641	98	51.05	31.48	76.06	97
		1993	1,232	747	1,673	98	55.95	33.93	76.00	93
		1994	1,198	806	1,671	98	53.75	36.15	74.95	95
		1995	1,320	857	1,647	97	58.46	37.96	72.94	92
		1996	1,351	905	1,675	97	59.11	39.61	73.29	88
		1997	1,370	948	1,714	98	59.17	40.92	74.03	87
		1998	1,398	1,033	1,751	98	59.32	43.82	74.27	85
		1999	1,287	1,102	1,493	98	54.09	46.30	62.76	89
		2000	1,441	1,160	1,767	97	60.06	48.35	73.66	80
		2001	1,381	1,221	1,644	98	56.96	50.33	67.79	85
		2002	1,492	1,263	1,772	96	60.83	51.49	72.24	76
		2003	1,530	1,309	1,905	94	61.92	52.97	77.08	75
		2004	1,589	1,353	2,083	94	63.87	54.38	83.73	68
		2005	1,676	1,424	2,308	93	66.82	56.76	92.02	62
		2006	1,807	1,521	2,620	91	71.45	60.14	103.55	58
		2007	1,981	1,537	3,013	88	77.34	60.02	117.62	49

Metropolitan Statistical Area	PWID Population	Year	Number	Min	Max	Rank	Rate	Min	Max	Rank
Wilmington--Newark, DE--MD	Total	1992	5,426	3,331	6,846	74	151.77	93.16	191.47	32
		1993	4,425	816	7,230	80	122.54	22.59	200.22	40
		1994	5,823	3,607	7,583	71	159.44	98.77	207.61	23
		1995	5,163	2,483	7,988	75	138.98	66.83	215.00	34
		1996	5,518	3,278	8,359	70	146.60	87.09	222.06	28
		1997	5,859	4,018	8,730	67	153.75	105.45	229.11	21
		1998	6,125	4,213	8,783	66	158.83	109.24	227.75	16
		1999	6,692	4,376	8,852	64	171.16	111.93	226.40	13
		2000	6,589	4,551	8,881	63	166.15	114.77	223.93	10
		2001	6,899	4,721	8,891	63	171.85	117.60	221.47	13
		2002	6,818	4,821	9,243	57	167.99	118.77	227.72	9
		2003	6,982	4,932	9,621	54	169.78	119.93	233.96	8
		2004	7,093	5,035	10,045	54	170.38	120.94	241.27	8
		2005	7,207	5,140	10,477	53	171.00	121.96	248.59	9
		2006	7,324	5,243	10,949	53	171.91	123.06	256.99	9
		2007	7,415	5,204	11,386	55	172.82	121.28	265.38	9
	Non-Hispanic White	1992	2,644	1,623	3,336	74	92.01	56.48	116.09	41
		1993	2,203	406	3,599	74	76.49	14.10	124.99	51
		1994	2,975	1,843	3,873	72	103.07	63.85	134.20	34
		1995	2,714	1,305	4,198	72	93.33	44.88	144.39	38
		1996	2,988	1,775	4,526	67	102.37	60.81	155.06	31
		1997	3,268	2,242	4,871	66	111.79	76.68	166.59	25
		1998	3,518	2,419	5,044	60	120.02	82.55	172.10	20
		1999	3,948	2,582	5,222	53	134.19	87.75	177.49	16
		2000	3,983	2,751	5,368	51	134.66	93.01	181.48	13
		2001	4,259	2,914	5,489	53	143.32	98.08	184.70	13
		2002	4,281	3,027	5,803	45	143.68	101.58	194.77	11
		2003	4,438	3,135	6,116	45	148.20	104.68	204.22	8
		2004	4,542	3,224	6,432	44	151.14	107.29	214.03	9
		2005	4,622	3,297	6,720	45	153.36	109.38	222.95	9
		2006	4,675	3,346	6,989	46	155.09	111.02	231.84	9
		2007	4,676	3,281	7,180	52	155.52	109.14	238.82	11

Metropolitan Statistical Area	PWID Population	Year	Number	Min	Max	Rank	Rate	Min	Max	Rank
Wilmington--Newark, DE--MD	Non-Hispanic Black	1992	2,061	1,265	2,601	54	385.38	236.55	486.20	33
		1993	1,649	304	2,695	55	298.40	55.01	487.59	45
		1994	2,092	1,296	2,724	51	365.31	226.30	475.67	25
		1995	1,761	847	2,724	53	294.24	141.48	455.19	41
		1996	1,764	1,048	2,672	51	284.05	168.74	430.26	34
		1997	1,739	1,193	2,591	51	269.98	185.18	402.31	33
		1998	1,679	1,155	2,407	53	251.98	173.30	361.31	35
		1999	1,692	1,106	2,238	52	246.10	160.93	325.52	29
		2000	1,543	1,066	2,080	55	218.22	150.73	294.10	35
		2001	1,511	1,034	1,947	55	207.72	142.15	267.70	38
		2002	1,419	1,003	1,923	56	190.08	134.39	257.67	38
		2003	1,410	996	1,943	56	183.72	129.77	253.16	38
		2004	1,429	1,014	2,023	56	180.81	128.34	256.04	37
		2005	1,496	1,067	2,174	48	183.44	130.83	266.68	38
		2006	1,625	1,163	2,429	47	192.70	137.95	288.08	36
		2007	1,827	1,282	2,806	42	211.22	148.24	324.36	32
	Hispanic	1992	125	77	158	74	132.04	81.05	166.58	51
		1993	130	24	212	73	126.95	23.40	207.44	45
		1994	208	129	271	68	186.24	115.37	242.51	27
		1995	217	104	335	64	176.94	85.08	273.71	31
		1996	263	156	399	61	197.32	117.22	298.88	24
		1997	309	212	461	60	212.87	146.01	317.21	18
		1998	350	241	502	60	224.46	154.38	321.86	17
		1999	406	266	537	57	239.78	156.80	317.16	15
		2000	420	290	566	58	229.73	158.68	309.62	16
		2001	458	313	590	57	238.28	163.07	307.09	15
		2002	470	332	637	58	231.44	163.63	313.73	16
		2003	501	354	691	57	235.35	166.25	324.32	15
		2004	535	379	757	57	239.61	170.08	339.30	15
		2005	577	412	839	57	246.64	175.91	358.55	14
		2006	635	455	950	57	257.82	184.56	385.42	13
		2007	713	500	1,095	56	278.23	195.26	427.26	13

Metropolitan Statistical Area	PWID Population	Year	Number	Min	Max	Rank	Rate	Min	Max	Rank
Wilmington--Newark, DE--MD	Male	1992	3,557	2,183	4,488	75	202.11	124.06	254.98	33
		1993	2,928	540	4,784	81	164.71	30.36	269.14	38
		1994	3,883	2,405	5,056	71	215.69	133.61	280.85	24
		1995	3,463	1,665	5,357	73	189.08	90.92	292.51	32
		1996	3,716	2,207	5,628	68	200.43	119.07	303.60	26
		1997	3,955	2,713	5,893	65	210.95	144.69	314.36	20
		1998	4,139	2,847	5,935	62	218.34	150.17	313.08	12
		1999	4,521	2,956	5,979	60	235.24	153.83	311.15	14
		2000	4,444	3,069	5,989	55	228.01	157.49	307.30	11
		2001	4,639	3,175	5,979	58	235.39	161.09	303.36	13
		2002	4,566	3,228	6,190	53	229.21	162.05	310.71	9
		2003	4,651	3,285	6,409	53	230.52	162.84	317.66	9
		2004	4,695	3,333	6,649	53	229.84	163.15	325.47	11
		2005	4,735	3,377	6,884	52	229.27	163.52	333.30	11
		2006	4,772	3,416	7,134	54	228.55	163.61	341.67	11
		2007	4,786	3,359	7,349	54	227.72	159.81	349.69	11
	Female	1992	1,960	1,203	2,473	67	107.95	66.26	136.20	29
		1993	1,562	288	2,553	78	85.22	15.71	139.25	37
		1994	2,019	1,251	2,629	68	109.02	67.53	141.96	23
		1995	1,766	849	2,732	76	93.74	45.07	145.01	31
		1996	1,869	1,110	2,831	76	97.84	58.12	148.20	26
		1997	1,973	1,353	2,939	72	101.90	69.89	151.85	21
		1998	2,057	1,415	2,950	71	104.91	72.15	150.43	17
		1999	2,248	1,470	2,974	68	113.08	73.95	149.57	16
		2000	2,220	1,533	2,992	66	110.08	76.03	148.36	14
		2001	2,336	1,599	3,011	66	114.31	78.22	147.31	16
		2002	2,324	1,643	3,151	65	112.46	79.51	152.45	15
		2003	2,399	1,695	3,306	64	114.54	80.91	157.84	15
		2004	2,459	1,746	3,483	64	115.99	82.33	164.25	13
		2005	2,522	1,799	3,667	63	117.36	83.70	170.61	14
		2006	2,588	1,853	3,869	59	119.11	85.27	178.07	13
		2007	2,645	1,856	4,062	57	120.83	84.80	185.55	13

Metropolitan Statistical Area	PWID Population	Year	Number	Min	Max	Rank	Rate	Min	Max	Rank
Wilmington--Newark, DE--MD	Young (15-29)	1992	1,466	900	1,850	73	119.38	73.27	150.61	29
		1993	1,253	231	2,048	76	103.53	19.08	169.16	39
		1994	1,757	1,088	2,288	63	146.35	90.66	190.57	17
		1995	1,676	806	2,593	60	139.58	67.12	215.93	17
		1996	1,937	1,151	2,934	54	161.08	95.69	243.99	11
		1997	2,223	1,525	3,313	48	184.30	126.41	274.65	6
		1998	2,503	1,721	3,589	43	206.45	141.99	296.03	4
		1999	2,921	1,910	3,864	39	239.71	156.75	317.06	2
		2000	3,040	2,100	4,097	38	248.07	171.35	334.34	3
		2001	3,321	2,272	4,279	36	270.85	185.35	349.06	2
		2002	3,372	2,384	4,571	35	274.15	193.83	371.63	2
		2003	3,487	2,463	4,805	36	279.92	197.73	385.74	2
		2004	3,506	2,489	4,965	36	278.06	197.38	393.75	2
		2005	3,442	2,454	5,003	39	269.05	191.89	391.13	4
		2006	3,280	2,348	4,904	41	253.09	181.18	378.36	6
		2007	3,000	2,105	4,606	48	229.94	161.37	353.10	7
	Old (30-64)	1992	4,059	2,492	5,121	73	172.95	106.16	218.20	32
		1993	3,225	595	5,270	81	134.37	24.77	219.57	40
		1994	4,116	2,550	5,359	74	167.87	103.99	218.58	29
		1995	3,523	1,694	5,450	79	140.11	67.37	216.74	36
		1996	3,620	2,151	5,483	78	141.31	83.95	214.04	35
		1997	3,684	2,527	5,490	77	141.47	97.03	210.81	32
		1998	3,686	2,535	5,286	77	139.41	95.88	199.90	32
		1999	3,855	2,521	5,099	74	143.25	93.67	189.47	31
		2000	3,643	2,517	4,910	76	132.95	91.84	179.19	33
		2001	3,682	2,520	4,745	78	132.04	90.36	170.16	29
		2002	3,543	2,505	4,802	77	125.23	88.54	169.76	31
		2003	3,572	2,523	4,922	75	124.61	88.02	171.72	28
		2004	3,624	2,573	5,132	73	124.87	88.64	176.83	26
		2005	3,737	2,665	5,432	69	127.29	90.78	185.05	22
		2006	3,920	2,806	5,860	65	132.23	94.66	197.68	16
		2007	4,166	2,924	6,397	58	139.52	97.91	214.25	11

Metropolitan Statistical Area	PWID Population	Year	Number	Min	Max	Rank	Rate	Min	Max	Rank
Youngstown--Warren, OH	Total	1992	1,187	530	1,704	100	30.95	13.83	44.46	100
		1993	1,160	668	1,807	100	30.24	17.42	47.12	100
		1994	1,451	822	1,908	99	37.85	21.43	49.79	99
		1995	1,395	821	2,013	99	36.45	21.45	52.59	99
		1996	1,526	898	2,126	99	39.86	23.46	55.54	96
		1997	1,655	981	2,239	99	43.37	25.71	58.67	96
		1998	1,809	1,066	2,363	98	47.44	27.97	61.97	94
		1999	2,229	1,803	2,487	95	58.66	47.43	65.45	83
		2000	2,148	1,253	2,693	95	56.75	33.09	71.13	81
		2001	2,667	2,259	2,987	93	70.68	59.87	79.16	71
		2002	2,491	1,430	3,360	93	66.20	38.02	89.30	71
		2003	2,650	1,520	3,735	92	70.56	40.48	99.45	67
		2004	2,794	1,599	4,117	92	74.60	42.68	109.91	66
		2005	2,937	1,672	4,493	92	78.67	44.79	120.36	61
		2006	3,083	1,746	4,883	92	82.80	46.90	131.17	58
		2007	3,210	1,800	5,246	91	86.87	48.72	141.97	55
	Non-Hispanic White	1992	648	289	931	100	18.99	8.48	27.28	100
		1993	612	353	953	100	17.96	10.35	27.98	100
		1994	753	426	990	100	22.16	12.55	29.15	100
		1995	723	426	1,043	100	21.36	12.57	30.82	100
		1996	801	471	1,116	100	23.70	13.95	33.03	100
		1997	889	527	1,203	98	26.46	15.68	35.78	98
		1998	1,003	591	1,310	98	29.93	17.65	39.10	93
		1999	1,282	1,037	1,430	96	38.51	31.14	42.97	86
		2000	1,285	749	1,611	95	38.88	22.67	48.73	82
		2001	1,661	1,406	1,860	91	50.45	42.73	56.50	73
		2002	1,612	926	2,174	93	49.15	28.23	66.30	74
		2003	1,777	1,020	2,505	92	54.33	31.17	76.57	68
		2004	1,935	1,107	2,850	91	59.33	33.94	87.41	65
		2005	2,089	1,189	3,196	84	64.34	36.63	98.44	62
		2006	2,240	1,269	3,549	83	69.32	39.26	109.81	57
		2007	2,370	1,329	3,873	82	73.98	41.49	120.91	53

Metropolitan Statistical Area	PWID Population	Year	Number	Min	Max	Rank	Rate	Min	Max	Rank
Youngstown--Warren, OH	Non-Hispanic Black	1992	448	200	643	91	128.57	57.43	184.65	93
		1993	457	263	712	92	129.89	74.83	202.37	91
		1994	580	329	763	87	161.99	91.73	213.09	81
		1995	552	325	796	87	152.10	89.52	219.43	78
		1996	584	344	814	87	159.17	93.69	221.80	73
		1997	601	356	813	86	162.53	96.36	219.84	68
		1998	612	361	800	84	163.40	96.33	213.45	64
		1999	692	560	772	79	182.31	147.42	203.42	57
		2000	604	352	757	79	157.30	91.71	197.15	59
		2001	672	569	753	75	175.44	148.60	196.48	53
		2002	561	322	756	81	146.45	84.10	197.56	57
		2003	533	306	751	82	139.44	80.00	196.52	58
		2004	505	289	744	83	132.27	75.68	194.88	60
		2005	484	275	740	85	126.79	72.18	193.97	61
		2006	473	268	749	86	123.09	69.71	194.98	61
		2007	472	264	771	86	123.20	69.10	201.35	62
	Hispanic	1992	34	15	48	89	67.79	30.28	97.36	73
		1993	33	19	51	89	63.71	36.70	99.26	72
		1994	41	23	55	87	78.15	44.26	102.80	65
		1995	41	24	59	87	74.20	43.67	107.05	59
		1996	46	27	64	87	79.89	47.02	111.32	56
		1997	51	30	69	88	86.40	51.22	116.86	52
		1998	57	34	75	87	92.90	54.77	121.35	46
		1999	72	58	80	85	112.91	91.30	125.98	38
		2000	70	41	88	85	103.08	60.10	129.19	42
		2001	87	74	98	83	124.57	105.51	139.51	32
		2002	80	46	108	83	113.05	64.92	152.50	34
		2003	83	47	117	85	114.02	65.41	160.69	33
		2004	82	47	121	87	111.40	63.74	164.13	34
		2005	80	45	122	88	105.27	59.93	161.05	36
		2006	75	43	119	88	96.58	54.70	152.98	40
		2007	68	38	111	89	84.68	47.49	138.39	45

Metropolitan Statistical Area	PWID Population	Year	Number	Min	Max	Rank	Rate	Min	Max	Rank
Youngstown--Warren, OH	Male	1992	670	299	962	100	36.01	16.09	51.72	100
		1993	681	392	1,061	100	36.53	21.05	56.92	100
		1994	876	496	1,152	99	46.81	26.51	61.57	99
		1995	856	504	1,236	99	45.68	26.89	65.90	99
		1996	944	556	1,315	99	50.23	29.57	69.99	99
		1997	1,025	608	1,386	99	54.57	32.35	73.80	96
		1998	1,113	656	1,454	99	59.19	34.90	77.32	94
		1999	1,357	1,097	1,514	96	72.13	58.32	80.48	86
		2000	1,287	750	1,613	97	68.52	39.95	85.88	85
		2001	1,566	1,326	1,754	93	83.59	70.80	93.62	76
		2002	1,429	821	1,928	94	76.53	43.95	103.23	76
		2003	1,483	851	2,090	94	79.49	45.61	112.04	74
		2004	1,524	872	2,245	93	81.85	46.83	120.59	72
		2005	1,561	889	2,388	94	84.05	47.85	128.59	67
		2006	1,599	906	2,533	94	86.28	48.86	136.67	67
		2007	1,630	914	2,664	95	88.57	49.67	144.75	66
	Female	1992	527	235	756	98	26.68	11.92	38.32	99
		1993	489	282	761	100	24.81	14.29	38.65	99
		1994	590	334	776	98	30.06	17.02	39.54	97
		1995	555	327	801	98	28.44	16.74	41.03	96
		1996	603	355	840	98	30.94	18.21	43.11	93
		1997	657	389	889	97	33.88	20.09	45.83	91
		1998	728	429	951	96	37.68	22.21	49.21	91
		1999	917	741	1,023	94	47.77	38.62	53.30	81
		2000	908	529	1,138	95	47.58	27.74	59.64	74
		2001	1,162	984	1,301	89	61.13	51.78	68.46	61
		2002	1,119	643	1,510	91	59.08	33.93	79.69	60
		2003	1,228	704	1,730	88	64.97	37.27	91.56	56
		2004	1,331	762	1,961	87	70.66	40.43	104.10	47
		2005	1,433	816	2,193	87	76.38	43.49	116.86	39
		2006	1,532	868	2,427	85	81.97	46.42	129.84	31
		2007	1,614	905	2,638	83	87.06	48.83	142.29	25

Metropolitan Statistical Area	PWID Population	Year	Number	Min	Max	Rank	Rate	Min	Max	Rank
Youngstown--Warren, OH	Young (15-29)	1992	327	146	469	100	28.11	12.56	40.38	99
		1993	242	140	378	100	21.16	12.19	32.96	100
		1994	254	144	334	100	22.44	12.71	29.52	100
		1995	226	133	327	100	20.16	11.87	29.09	99
		1996	251	148	349	100	22.46	13.22	31.29	99
		1997	296	176	401	100	26.75	15.86	36.18	98
		1998	372	219	486	100	33.85	19.96	44.22	92
		1999	546	441	609	94	50.30	40.67	56.12	81
		2000	634	370	795	94	59.28	34.56	74.31	73
		2001	945	800	1,058	91	89.22	75.57	99.92	55
		2002	1,037	596	1,400	88	98.37	56.49	132.70	49
		2003	1,260	723	1,775	86	119.00	68.27	167.71	36
		2004	1,463	837	2,155	84	137.60	78.73	202.73	26
		2005	1,632	929	2,496	79	153.10	87.16	234.22	21
		2006	1,747	989	2,767	78	163.00	92.32	258.21	19
		2007	1,774	995	2,899	78	166.16	93.19	271.56	19
	Old (30-64)	1992	889	397	1,277	100	33.30	14.87	47.83	100
		1993	940	542	1,465	100	34.96	20.14	54.47	100
		1994	1,222	692	1,607	97	45.26	25.63	59.53	98
		1995	1,194	703	1,722	98	44.12	25.97	63.66	96
		1996	1,305	768	1,819	98	48.12	28.33	67.06	94
		1997	1,397	828	1,890	97	51.57	30.57	69.75	94
		1998	1,486	876	1,941	96	54.78	32.29	71.55	91
		1999	1,756	1,420	1,960	94	64.68	52.30	72.17	82
		2000	1,594	929	1,998	95	58.69	34.22	73.57	83
		2001	1,828	1,548	2,047	94	67.35	57.04	75.42	78
		2002	1,549	889	2,089	95	57.19	32.84	77.15	80
		2003	1,475	846	2,079	96	54.69	31.38	77.08	78
		2004	1,388	794	2,045	97	51.74	29.60	76.22	82
		2005	1,316	749	2,013	98	49.33	28.08	75.47	86
		2006	1,280	725	2,028	97	48.30	27.35	76.51	86
		2007	1,292	725	2,112	96	49.18	27.58	80.38	78
